# Lung Surfactant Protein B Peptide Mimics Interact with the Human ACE2 Receptor

**DOI:** 10.3390/ijms241310837

**Published:** 2023-06-29

**Authors:** Alan J. Waring, Grace C.-L. Jung, Shantanu K. Sharma, Frans J. Walther

**Affiliations:** 1 Department of Medicine, David Geffen School of Medicine, University of California Los Angeles, Los Angeles, CA 90095, USA; awaring@ucla.edu (A.J.W.); cljung@mednet.ucla.edu (G.C.-L.J.); 2 The Lundquist Institute for Biomedical Innovation at Harbor-UCLA Medical Center, Torrance, CA 90502, USA; 3 Materials and Process Simulation Center, California Institute of Technology, Pasadena, CA 91125, USA; ssharma@alumni.caltech.edu; 4 Department of Pediatrics, David Geffen School of Medicine, University of California Los Angeles, Los Angeles, CA 90095, USA

**Keywords:** lung surfactant, surfactant protein B peptide mimics, COVID-19, ACE2 receptor protein, SARS-CoV-2 binding, surface plasmon resonance

## Abstract

Lung surfactant is a complex mixture of phospholipids and surfactant proteins that is produced in alveolar type 2 cells. It prevents lung collapse by reducing surface tension and is involved in innate immunity. Exogenous animal-derived and, more recently, synthetic lung surfactant has shown clinical efficacy in surfactant-deficient premature infants and in critically ill patients with acute respiratory distress syndrome (ARDS), such as those with severe COVID-19 disease. COVID-19 pneumonia is initiated by the binding of the viral receptor-binding domain (RBD) of SARS-CoV-2 to the cellular receptor angiotensin-converting enzyme 2 (ACE2). Inflammation and tissue damage then lead to loss and dysfunction of surface activity that can be relieved by treatment with an exogenous lung surfactant. Surfactant protein B (SP-B) is pivotal for surfactant activity and has anti-inflammatory effects. Here, we study the binding of two synthetic SP-B peptide mimics, Super Mini-B (SMB) and B-YL, to a recombinant human ACE2 receptor protein construct using molecular docking and surface plasmon resonance (SPR) to evaluate their potential as antiviral drugs. The SPR measurements confirmed that both the SMB and B-YL peptides bind to the rhACE2 receptor with affinities like that of the viral RBD–ACE2 complex. These findings suggest that synthetic lung surfactant peptide mimics can act as competitive inhibitors of the binding of viral RBD to the ACE2 receptor.

## 1. Introduction

The COVID-19 pandemic has resulted in hundreds of millions of infected patients, with disease severity ranging from mild flu-like symptoms to severe acute respiratory distress syndrome (ARDS) and death. In humans, infection with the SARS-CoV-2 virus can lead to an acute viral infection with a mean incubation time of around 5 days. Clinical symptoms include fever, cough, fatigue, muscle pain, and dyspnea. A subsequent rapid worsening of respiratory problems may require invasive mechanical ventilation with oxygen supplementation and an administration of antivirals, monoclonal antibody therapy, and anti-coagulants [1,2].

In humans, membrane-bound protein angiotensin-converting Enzyme 2 (ACE2) is expressed in the epithelium of the lungs, small intestines, heart, liver, and kidneys. In the lungs, ACE2 is mainly present on the membranes of alveolar epithelial Type 2 cells. Binding of the receptor-binding domain (RBD) of the SARS-CoV-2 Spike 1 (S1) protein to ACE2 plays an essential role in viral entry and results in damage to the alveoli. Following infection, SARS-CoV-2 replicates in the cells of the respiratory and intestinal epithelium, leading to tissue damage and associated clinical symptoms. The destruction of alveolar Type 2 cells by SARS-CoV-2 results in a loss of active lung surfactant and native immunity because these cells produce and secrete lung surfactant [1,3].

**Figure 1:**
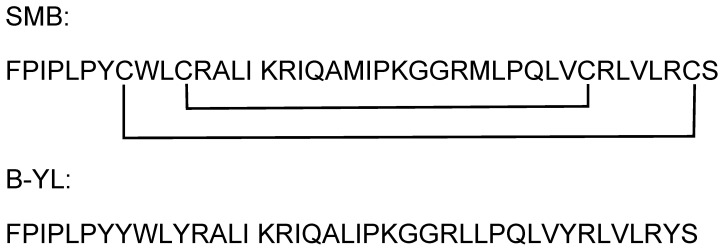
Amino acid sequences for the synthetic lung surfactant protein B peptide constructs Super Mini-B (SMB) and B-YL.

Mammalian lung surfactant consists of about 80% phospholipids, 10% neutral lipids, and 10% proteins. Two of the four surfactant proteins, the hydrophilic surfactant proteins A and D (SP-A and SP-D), are important for native immunity of the lung, whereas the two hydrophobic surfactant proteins, B and C (SP-B and SP-C), are surface active, reduce surface tension at the air–liquid interface in the alveoli, and prevent alveolar collapse. The absence of SP-B in the lung is lethal in humans and mammals. Preterm infants with neonatal respiratory distress syndrome (RDS) due to lung immaturity and surfactant deficiency benefit from treatment with the intratracheal instillation of an exogenous lung surfactant. The clinical surfactant, produced by extracting animal lungs and lavage material, consists of surfactant lipids and SP-B and SP-C and has led to a sharp reduction in morbidity and mortality of preterm infants. In a small clinical study, surfactant treatment reduced mortality and duration of mechanical ventilation in ARDS patients with COVID-19-associated ARDS [4]. The anti-inflammatory activity of SP-B and phospholipids of exogenous surfactant may also provide lung-protective support in these cases [5,6]. Recently, the availability of highly functional SP-B and SP-C peptide mimics has led to the development of advanced synthetic lung surfactants [7]. Two of these surfactant formulations consist of 3% of the SP-B peptide mimics, Super Mini-B (SMB) [8] or B-YL [9] (Figure 1), mixed in three surfactant phospholipids (DPPC, POPC, and POPG 5:3:2 by weight).

Thus, a key element in the infection of host cells by SARS-CoV is the binding of the viral S1 protein to the ACE2 receptor on the host cellular membrane. This initial interaction of the viral RBD with the host membrane receptor is a major determinant of the membrane fusion of the virus with the host lung cells [10,11]. Recently, there have been several peptide-based approaches that target these initial steps involved in the interaction RBD of the viral S1 protein with the ACE2 receptor and aim to prevent viral entry [12,13,14,15,16]. In this study, we characterize the interaction of the synthetic lung SP-B peptide mimics, SMB and B-YL, with the ACE2 receptor.

## 2. Results

### 2.1. Determination of the Potential SMB and B-YL Peptides to Form Complexes with Human ACE2 Receptor Using Molecular Docking

**Figure 2:**
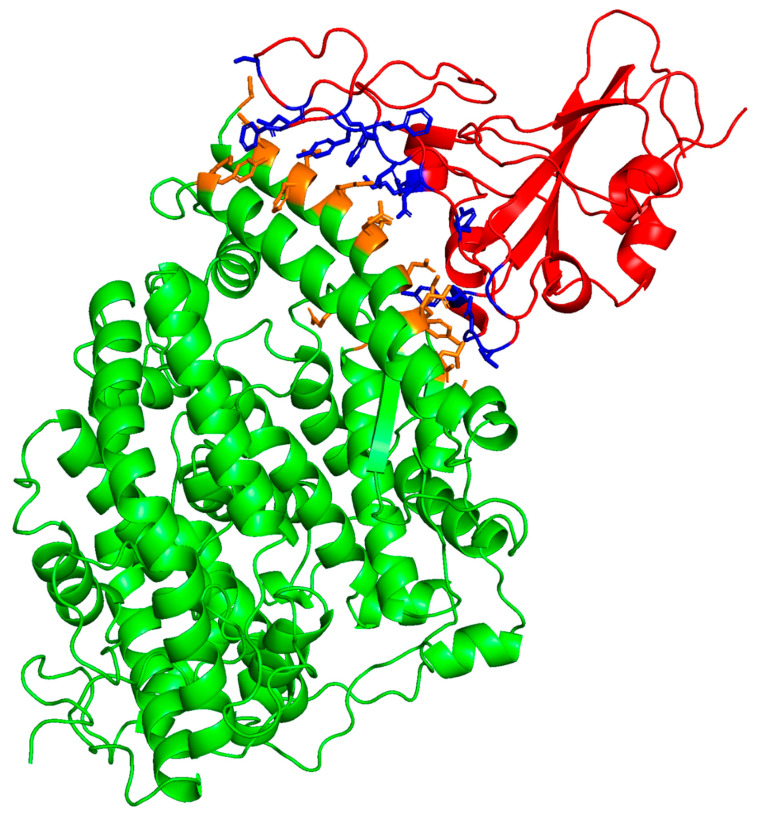
Molecular illustration of human ACE2-viral RBD crystal structure (PDB accession code: 6m0j.pdb). ACE2 receptor is shown in green highlight with binding domain interface highlighted in orange. Viral RBD construct highlighted in red with binding domain interface in blue. Contact cutoff defined at 5.5 angstroms.

The molecular coordinates of the crystal structure of the ACE2–SARS-CoV-2 spike protein RBD complex (PDB accession code: 6m0j) were used as the initial template for the prediction of the degree of interaction of the ACE2–RBD interfacial domain (Figure 2). The binding interface contact residue pairs are listed in Table 1. This complex coordinate set was used to determine binding affinity by contacts-based prediction methodology [17]. Both the affinity or binding-free energy (ΔG) and dissociation constant (K_D_) were predicted from this structural information. The structurally derived binding metrics compare favorably with experimentally determined binding measurements of ACE2-SARS-CoV-2 interactions using SPR (Table 2). Residue pair interactions include both residue side chain ion pairs and hydrophobic interactions of aromatic residue side chains that stabilize the receptor–viral protein complex. The correlations of these in-silico predictions with experimental binding observations suggest that the use of this structure-based approach to the prediction of the potential binding of peptides and proteins to the viral–receptor interface is a reasonable approach for the estimation of possible experimental residue-specific molecular interactions.

**Figure 3:**
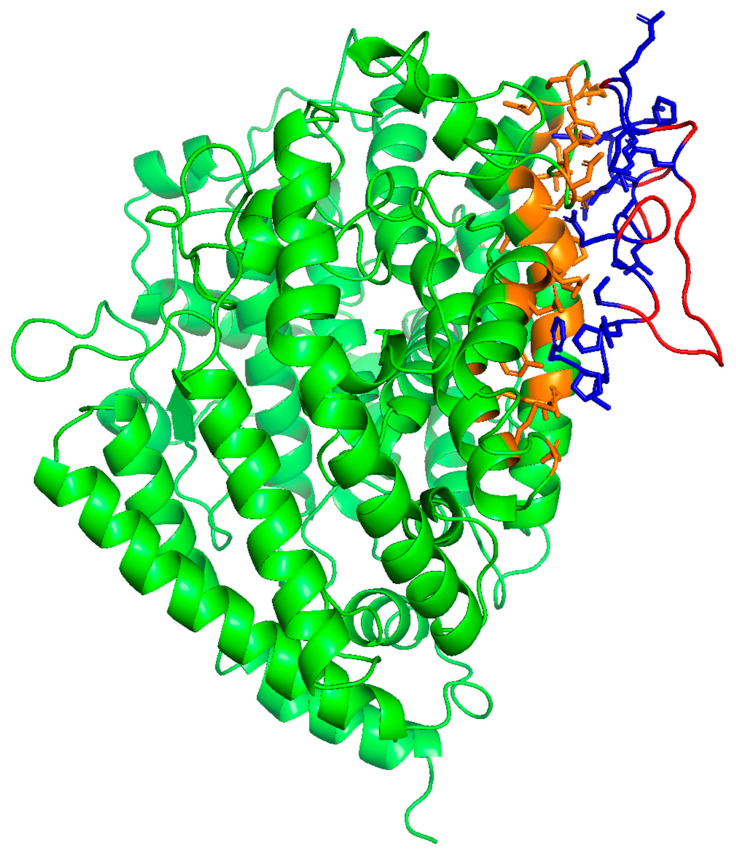
Molecular illustration of molecular dynamic-refined complex of ACE2 receptor with SMB peptide interacting with viral binding domain (Supplementary Materials File S1; MolecularArchive accession code: ma-r9084). ACE2 human receptor highlighted in green with binding domain in orange. The SMB peptide is highlighted in red with binding domain-interactive residues shown in blue. Interactive residue contact cutoff defined at 5.5 angstroms.

The potential SMB and B-YL peptide interactions with the ACE2 receptor protein were estimated from the docking of the peptide into the crystal structure of each of the molecular complex coordinates (PDB accession code: 6m0j) using integrative modeling of the potential SMB-ACE2 and B-YL-ACE2 biomolecular complexes [18]. As shown in Figure 3, the SMB and B-YL peptides can be docked into the ACE2 viral binding site of the protein constructs. Analysis of the complex structures with contact-based prediction methodology indicates that binding affinities are like that of the native biomolecular complex (Table 2), and that the interactive residues of the ACE2 construct are in the same N-terminal helical domain as observed for the ACE2–RBD crystal structure.

**Figure 4:**
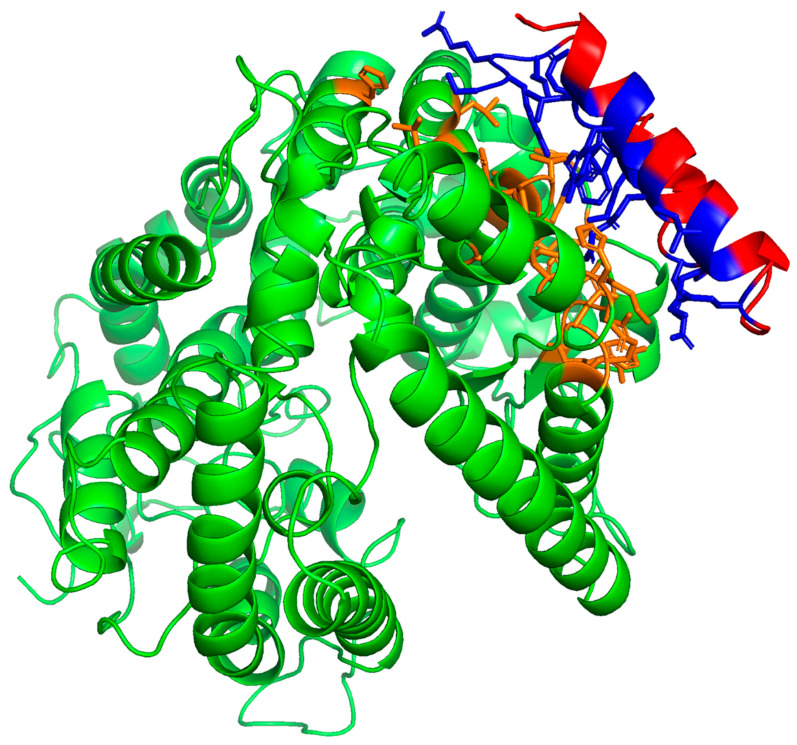
Molecular illustration of molecular dynamic-refined complex of ACE2 receptor with B-YL peptide interacting with viral binding domain (Supplementary Materials File S2; MolecularArchive accession code: ma-amyf9). ACE2 human receptor highlighted in green with binding domain in orange. The B-YL peptide is highlighted in red with binding domain-interactive residues shown in blue. Interactive residue contact cutoff defined at 5.5 angstroms.

A structural analysis of the interface between the ACE2 receptor and the SMB and B-YL peptides indicates that the ACE2 N-terminal helix interacts with the N-terminal helix of the SMB and B-YL peptides. The interactive residues of the helical domains are listed in Table 1 and illustrated in Figure 3 and Figure 4. These amino acid side chain interactions include charged residues that form a salt bridge between glutamic acid residues of an ACE2 helix with an arginine residue of the N-terminal helix of the SMB and B-YL peptides. There are also hydrophobic residue side chain interactions that stabilize the protein–peptide complex that include the aromatic side chains of tyrosine residue pairs, as well as the leucine and alanine of the ACE2 N-terminal helix interacting with the tyrosine side chains of the SMB and B-YL peptide helical domains.

### 2.2. Surface Plasmon Resonance (SPR) Measurements of SMB and B-YL Peptide Binding to ACE-2 Domain

To better characterize the potential degree and type of interaction of the lung ACE2 receptor protein and lung surfactant peptides SMB and B-YL, we used SPR to test the binding of these peptides to the recombinant human ACE2 receptor construct (Supplementary Materials File S3). The SPR binding study was accomplished by immobilizing the ligand (ACE2) on the SPR sensor surface and flowing the solute containing the SMB or B-YL peptide past the sensor-linked molecule. The binding of the solute to the sensor surface ligand results in an evanescent sensor response and is measured in response units (RU) that are proportional to the bound mass. This technique has been shown to be very useful in the study of ligand binding in protein–protein interactions [19], such as those in the current study.

**Figure 5:**
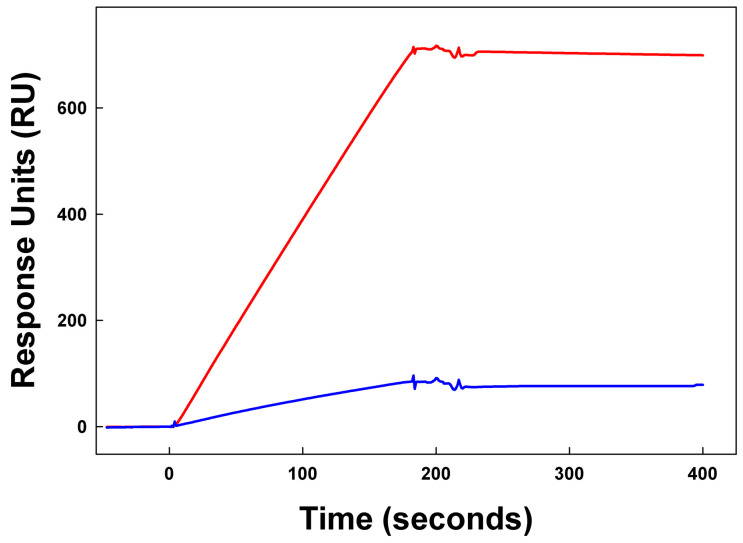
Surface plasmon resonance (SPR) sensor grams of SMB (red) and B-YL (blue) peptide binding to the rhACE2 receptor protein (Supplementary Materials File S3). Solutions of 1 µM of recombinant protein in HBS–EP buffer were then flowed over the respective chip-linked peptides. The SPR responses are in relative response units (RU) on the Y axis.

Representative sensor grams for the interaction of the lung surfactant SMB and B-YL peptides tested in the present study with the ACE-2 receptor protein are shown in Figure 5. The kinetic analysis of these traces to determine relative binding (affinity) of the peptides to the expressed protein construct is summarized in Table 2 and Table 3. The ACE2 construct (Figure 5) had the highest binding affinity for the SMB peptide and lowest dissociation constant (K_D_). B-YL peptide had a lower affinity with corresponding higher K_D_ values (Table 3).

## 3. Discussion

Although the development of modern vaccines has been an important step in helping control the spread of the COVID-19 virus [20], an alternative therapeutic approach to viral infection intervention can be found in the development of peptides that bind the host ACE-2 receptor COVID-19-binding domain interface in the lungs, thereby blocking viral entry into the host [12,21]. The peptide-based ACE-2 interaction blockers fall into two categories. One class consists of short peptides that are six amino acid residues in length. Typically, these short peptide amino acid sequences have hydrophobic residues that are dominated by tyrosine interspersed with polar cationic residues of lysine and arginine [13,14]. These short amino acid sequences have little secondary conformation, even when bonded in a complex with the receptor protein. The second class of peptide viral fusion blockers have amino acid sequences that span from approximately 20–60 residues in length [12,16,22]. These blocking inhibitors have considerable alpha helical propensity and assume a tertiary fold of a helix–hairpin-like structure. The amino acids in the longer peptide sequences have a sequential distribution that resembles an amphipathic helix. Polar-charged residues include arginine and lysine, and hydrophobic residues include aromatic amino acids such as tyrosine, phenylalanine, and tryptophan. These amino acid sequences also contain residues such as asparagine and glutamine, which provide additional hydrogen bonding between the peptide and the target receptor sequence and help stabilize the potential receptor–viral protein complex.

In the present study we examined the potential interaction of two hydrophobic lung surfactant peptide mimics of surfactant protein B, named SMB and B-YL, with the ACE-2–SARS-CoV-2 Receptor-Binding Domain. The rationale for the selection of these specific peptide mimics was in part based on the amino acid sequence of the peptides (Figure 1), which are composed of multiple tyrosine residues and cationic polar amino acid residues arginine and lysine. Additionally, both the SMB and B-YL sequences have a distribution of polar and non-polar residues that help enhance the amphipathic alpha helical secondary structure by providing the peptide a helix hairpin character and a tertiary Saposin fold [23,24]. These primary and secondary features of the SMB and B-YL peptides suggest that they resemble many of the known peptide inhibitors of the ACE-2–COVID-19 spike protein complex. Docking of the SMB and B-YL peptides into the ACE-2 RBD binding interface allows for the prediction of a high propensity for interactions between the peptide and the receptor–viral protein interface. Experimental in-vitro SPR measurements of the binding of the SMB and B-YL peptides to the recombinant human ACE-2 receptor protein confirm the binding of the peptides to this target domain. These results suggest that lung surfactant protein B peptide mimics have potential for use in the therapeutic intervention of COVID-19 infection in the lung. We speculate that the observed experimental difference binding affinity of the SMB peptide compared to the B-YL construct for the ACE-1 receptor may be related to the greater rigidity of the disulfide-linked structure of the helix hairpin mimic conformation [23,24].

This study was performed in an aqueous solution, but, since SP-B is a lipid-associated protein, it is expected that these peptides require binding to lipid membranes to adopt a functional structure. This raises the question whether the interaction of SMB and B-YL with ACE2 can be affected by the presence of surfactant membranes. A recent paper by Rozak et al. [25] suggests that lipids do not interfere with viral binding to ACE2, and the same could be true for surfactant lipids and peptide binding.

While the present findings, demonstrating the interaction of synthetic surfactant B peptides with the human ACE2 receptor, are very encouraging regarding the possible blocking of the binding site to the COVID-19 spike protein, more detailed studies of the peptides in various synthetic lipid formulations will be required to optimize this system for therapeutic applications.

## 4. Materials and Methods

### 4.1. Protein–Peptide Docking and Prediction of Binding Affinities 

The potential interactions of the lung SP-B peptide mimics and the components of the S1 Protein-ACE2 receptor protein complex were initially determined by downloading the molecular coordinates of the SMB and B-YL peptides from the ModelArchive website (https://www.modelachive.org accessed on 25 May 2023; ID: ma-scodz for SMB and ma-vilb7 for B-YL) and the S/RBD–ACE2 crystal structure from the Protein Data Bank (https://www.rcsb.org accessed on 25 May 2023; ID: 6M0J). Protein–peptide docking and analysis were carried out using the HADDOCK 2.4 server (V 2.4-2021.05; https://www.bonvinlab.org/education/HADDOCK24/ accessed on 25 May 2023) [18,26]. The docked structures were then analyzed with Prodigy (https://wenmr.science.uu.nl/prodigy/ accessed on 25 May 2023) [17,27] to estimate the binding-free energy and the affinity constants for the residue-specific interactions of the peptide–protein complex.

### 4.2. Protein and Peptide Constructs

HBS–EP buffer (10 mM Hepes, pH 7.4, 150 mM NaCl, 3 mM EDTA, 0.005% surfactant P20, pH 7.4) was obtained from Biacore (Uppsala, Sweden). Organic solvents used for sample synthesis and purification were HPLC grade or better (Fisher Scientific, Waltham, MA, USA). The synthetic SMB and B-YL lung surfactant peptides, of which amino acid sequences are shown in Figure 1, were synthesized using a standard Fmoc protocol and HPLC purified as described previously [8,23].

SMB and B-YL peptides were assembled using H-Ser(OtBu)-HMPB NovaPEG serine resin (Novabiochem, MilliporeSigma, Burlington, MA, USA) possessing a substitution of 0.62 mmole/g. Peptide synthesis was carried out at a 0.25 mmol scale on a Liberty Microwave Peptide (CEM Corporation, Matthews, NC, USA) synthesizer employing the standard Fmoc protocol [24]. Both SMB and B-YL peptides were double-coupled from the C-terminus to Residue 29, followed by triple-coupling for all remaining amino acid residues to the N-terminal phenylalanine to insure optimal yield. Peptides were cleaved from the resin for one hour using the phenol:thioanisole:ethanedithiol:water:trifluoracetic acid (0.75:0.25:0.5:0.5: 10, *v*:*v*) cleavage–deprotection mixture. The crude peptide was then purified to greater than 95% by a Jasco preparative HPLC (Easton, MD, USA) using a VYDAC diphenyl or C8 (1” by 12” width by length) column at 20 mL/min. The peptides were eluted from the column with a 0 to 100% ACN (water to acetonitrile) with 0.1% TFA as an ion-pairing agent added to both aqueous and organic phases with a linear gradient in 1 h. The purified product solution was then lyophilized, and the mass confirmed the resulting peptide powder by Maldi TOF mass spectrometry.

In the case of SMB, the Saposin protein-like peptide folded [24] and directed disulfide formation between Cys-8 and Cys-40, as well as between Cys-11 and Cys-34, and was facilitated by dissolution in TFE with the oxidant dimethylsulfoxide (DMSO) and 50 mM of PBS pH 7.5 (TFE:DMSO:PBS, 3:1.5:5.5, *v*:*v*) to mediate oxidation of the thiols to form disulfides [28]. The peptide–aqueous–organic solvent solution was stirred for 48 h at 25 °C to ensure complete disulfide formation before the concentration of the peptide by Speed–Vac^®^. Purification of the oxidized SMB by HPLC was performed as described above for the crude product and the oxidized peptides disulfide-linked molecular mass confirmed by Maldi–TOF mass spectrometry. Peptide concentrations were determined by the UV spectrometry method of Anthis and Clore [29].

Expressed viral spike and human ACE2 receptor protein constructs were supplied by R&D Systems (https://www.bio-techne.com accessed on 25 May 2023). The recombinant human ACE2 receptor construct included residues Glutamine 18 to Serine 740 of the full-length protein with a C-terminal 10 Histidine tag.

### 4.3. Surface Plasmon Resonance (SPR) Measurements of the Binding of SMB and B-YL Surfactant Peptides to Lung Receptor ACE-2 Domain

The binding affinity of the SMB and B-YL lung surfactant peptides to the recombinant human ACE-2 receptor construct was measured with SPR spectroscopy using a Biacore T100 system (GE Healthcare Bio-Sciences Corp, Piscataway, NJ, USA). Due to limited aqueous solution solubility, the SMB and B-YL peptides were dissolved in DMSO at 1 mg/mL since this solvent enhances the dominant alpha helical conformation of these peptides (Supplementary Materials File S4). The organic-solvated peptides were then diluted into 1–2000 dilutions in the HBS–EP buffer for injection. Binding measurements were then made by flowing a running buffer solution of the test sample in the HBS–EP buffer over the chip-associated ACE2 at a flow rate of 30 µL/min for 5 min to determine the binding affinity at 37 °C. The binding of SMB and B-YL peptides dissolved in the analyte was determined from their sensor grams, in which the arbitrary response units (RU) are recorded as a function of time. The binding associated with control medium containing no peptide was subtracted from final affinity traces. The mean “on” and “off” rate constants (k_on_ and k_off_) and the dissociation equilibrium constant (K_D_ = k_off_/k_on_) were calculated using Biacore Insight Evaluation Software version 5, based on curve-fitting measurements.

## Figures and Tables

**Table 1:** Predicted contact residue pairs for the ACE2-SMB and ACE2-B-YL interface docked complexes. ACE2 protein residues are labeled “A,” RBD protein and SMB and B-YL peptide residues are designated “B.”

ACE2-RBD	ACE2–B-YL	ACE2-SMB
Contact Residues	Contact Residues	Contact Residues
LEU 45 A TYR 446 B	ARG 340 A ARG 27 B	PRO 218 A ALA 13 B
TYR 83 A ASN 487 B	VAL 370 A LEU 5 B	THR 214 A PRO 4 B
TYR 83 A PHE 486 B	ARG 376 A ARG 12 B	GLU 466 A ARG 12 B
ARG 357 A THR 500 B	VAL 370 A TRP 9 B	LEU 219 A LYS 16 B
HIS 34 A LEU 455 B	ALA 367 A TYR 40 B	LYS 217 A PRO 4 B
LYS 31 A LEU 455 B	HIS 17 A TYR 8 B	THR 591 A ARG 12 B
ASP 355 A ASN 501 B	LEU 538 A ARG 39 B	LYS 441 A PRO 2 B
LYS 31 A PHE 490 B	LEU 543 A SER 41 B	ASP 592 A TRP 9 B
ALA 386 A TYR 505 B	ASP 338 A ARG 27 B	GLN 581 A MET 21 B
GLN 24 A TYR 489 B	PRO 372 A TYR 8 B	TRP 589 A CYS 8 B
ARG 393 A TYR 505 B	ARG 376 A LEU 28 B	LYS 459 A PHE 1 B
LEU 45 A GLN 498 B	ALA 369 A GLN 31 B	THR 214 A TYR 7 B
LYS 353 A GLN 498 B	ASN 16 A TYR 8 B	GLU 466 A PRO 4 B
ASP 30 A LYS 417 B	THR 542 A ARG 39 B	VAL 587 A GLY 25 B
LYS 353 A GLY 502 B	LEU 538 A SER 41 B	MET 463 A PHE 1 B
ASP 355 A GLY 502 B	GLN 371 A LEU 5 B	LEU 219 A GLN 19 B
GLY 354 A THR 500 B	PHE 339 A GLN 31 B	PHE 586 A ARG 27 B
LYS 31 A GLN 493 B	VAL 370 A GLN 31 B	LYS 441 A PHE 1 B
ASN 330 A THR 500 B	HIS 555 A SER 41 B	THR 591 A TRP 9 B
ASP 30 A LEU 455 B	HIS 17 A LYS 16 B	SER 585 A GLY 26 B
TYR 41 A GLN 498 B	GLY 337 A GLN 31 B	ASN 582 A MET 21 B
THR 27 A TYR 473 B	ASN 305 A TYR 34 B	ASP 592 A GLN 31 B
GLU 35 A GLN 493 B	GLN 539 A ARG 39 B	TYR 437 A ILE 3 B
SER 19 A ALA 475 B	VAL 370 A TYR 40 B	PHE 586 A MET 28 B
GLN 42 A TYR 449 B	VAL 370 A ARG 35 B	ASN 590 A GLN 31 B
GLY 354 A VAL 503 B	LYS 336 A ARG 39 B	VAL 587 A PRO 23 B
TYR 41 A ASN 501 B	LYS 536 A ARG 39 B	ARG 583 A MET 21 B
THR 27 A ALA 475 B	LYS 535 A ARG 39 B	TRP 589 A MET 28 B
GLN 24 A SER 477 B	LYS 336 A TRP 9 B	SER 585 A LYS 24 B
ASP 38 A GLN 498 B	GLU 20 A LEU 28 B	PHE 586 A GLY 26 B
HIS 34 A TYR 453 B	LEU 538 A LEU 38 B	VAL 587 A MET 21 B
GLN 24 A PHE 486 B	PRO 304 A LEU 38 B	HIS 222 A MET 21 B
ASP 38 A TYR 449 B	LYS 336 A GLN 31 B	THR 214 A LEU 14 B
LYS 353 A PHE 497 B	GLU 21 A LYS 16 B	TRP 589 A ARG 12 B
TYR 41 A THR 500 B	GLN 371 A TYR 8 B	HIS 222 A GLN 19 B
HIS 34 A LYS 417 B	HIS 17 A ARG 12 B	GLU 462 A PHE 1 B
GLY 354 A GLY 502 B	LEU 538 A TYR 40 B	GLU 210 A ILE 3 B
TYR 83 A TYR 489 B	ALA 369 A TRP 9 B	LYS 217 A LEU 5 B
LEU 79 A PHE 486 B	THR 542 A SER 41 B	PHE 575 A GLN 19 B
LYS 353 A ASN 501 B	ARG 376 A GLN 31 B	ASN 590 A TRP 9 B
LYS 31 A TYR 489 B	TYR 24 A GLY 25 B	GLN 221 A LYS 16 B
ASP 24 A GLY 496 B	GLN 539 A SER 41 B	LYS 217 A TYR 7 B
GLN 24 A ASN 487 B	GLU 20 A ARG 12 B	PRO 218 A LEU 14 B
GLY 354 A TYR 505 B	LYS 336 A GLY 25 B	GLU 466 A TRP 9 B
GLN 42 A GLY 447 B	GLY 337 A LEU 28 B	GLU 210 A ILE 3 B
PHE 28 A ASN 487 B	HIS 17 A ILE 15 B	LYS 217 A LEU 5 B
ASP 355 A THR 500 B	VAL 370 A TYR 8 B	PHE 575 A GLN 19 B
GLY 354 A ASN 501 B	TYR 24 A ARG 27 B	VAL 587 A LYS 16 B
GLN 42 A GLY 446 B	MET 366 A TYR 34 B	HIS 222 A LYS 16 B
LEU 45 A THR 500 B	GLY 302 A LEU 38 B	GLN 221 A LEU 14 B
GLN 24 A GLY 476 B	GLU 20 A LYS 24 B	LYS 217 A CYS 8 B
LYS 353 A TYR 495 B	HIS 17 A GLN 19 B	GLU 462 A SER 41 B
LYS 31 A GLU 484 B	PRO 304 A TYR 34 B	GLU 466 A SER 41 B
GLU 37 A TYR 505 B	GLN 371 A SER 41 B	GLU 210 A PRO 4 B
LYS 31 A PHE 456 B	LYS 336 A LEU 28 B	LYS 441 A ILE 3 B
HIS 34 A GLN 493 B	THR 542 A TYR 40 B	PRO 218 A GLN 19 B
PHE 28 A TYR 489 B	THR 307 A TYR 34 B	THR 591 A SER 41 B
LYS 353 A GLY 496 B	GLN 363 A TYR 34 B	MET 445 A PRO 2 B
GLN 42 A GLN 498 B	MET 366 A GLN 31 B	GLN 221 A ARG 12 B
THR 27 A TYR 489 B	ARG 376 A TRP 9 B	TYR 437 A PRO 4 B
GLN 24 A ALA 475 B	MET 366 A TYR 40 B	THR 242 A ARG 27 B
ASP 30 A PHE 456 B	VAL 370 A SER 41 B	GLU 466 A TYR 7 B
MET 82 A PHE 486 B	PRO 372 A ARG 12 B	VAL 587 A GLY 26 B
LYS 353 A TYR 505 B	PHE 373 A ARG 12 B	VAL 587 A ARG 12 B
	GLN 371 A TRP 9 B	LYS 579 A MET 21 B
	LYS 336 A LYS 24 B	SER 585 A GLY 25 B
	TYR 368 A TYR 40 B	GLU 466 A CYS 8 B
	GLU 21 A ARG 12 B	GLN 221 A LYS 24 B
	ASN 16 A ARG 12 B	LYS 579 A ALA 20 B
		THR 242 A GLY 26 B
		PHE 213 A PRO 4 B
		THR 591 A CYS 8 B
		GLU 580 A MET 21 B
		MET 445 A PHE 1 B
		ILE 467 A PHE 1 B
		GLY 588 A ARG 12 B
		VAL 587 A LYS 24 B
		ARG 583 A ILE 22 B
		GLU 210 A PHE 1 B
		ASP 592 A MET 28 B
		PHE 586 A GLY 25 B
		LYS 217 A ARG 12 B
		ASN 590 A MET 28 B
		PRO 218 A ILE 15 B
		TYR 437 A PHE 1 B
		GLY 588 A LYS 24 B
		LEU 578 A MET 21 B
		ASN 590 A ARG 12 B
		ARG 465 A ARG 12 B
		ARG 583 A ALA 20 B
		THR 591 A MET 28 B
		GLU 450 A PRO 2 B

**Table 2:** Comparison of predicted binding parameters and SPR experimentally derived binding measurements.

Protein-Protein Interaction	ΔG (kcal mol^−1^)	K_D_ (M)
Binding-Free Energies and Affinities of the binding of ACE2–SARS-CoV-2 RBD from Experimental SPR Measurements compared with that using structure-based prediction mythology		
ACE2–RBD (PDB: 6m0j.pdb)	−11.90	1.90 × 10^−9^
ACE2–RBD (SPR Data) *	−11.82	4.67 × 10^−9^
Binding-Free Energies and Affinities Predicted from Docking of Molecular Complexes (HADDOCK)		
ACE2–SMB	−13.5	3.10 × 10^−10^
ACE2–B-YL	−11.1	5.20 × 10^−9^
Experimentally Determined Binding-Free Energies and Affinities from SPR experimental measurements		
ACE2–SMB-expt.data-SPR	−11.4	9.87 × 10^−9^
ACE2–B-YL-expt.data-SPR	−12.6	1.27 × 10^−9^

* RBD(wt)-ACE2 SPR binding data [10]. ΔG° = –RT ln1/K_D_ = RT ln K_D,_ where ΔG° is the standard Gibbs free energy change, R is the universal gas constant, T is the absolute temperature (K), and K_D_ is the equilibrium dissociation constant.

**Table 3:** Binding data of SMB and B-YL surfactant peptides with rhACE2 receptor protein derived from experimentally determined peptide–protein interaction data using SPR.

	ka (1/Ms)	kd (1/s)	Rmax (RU)	RI (RU)	Conc of Analyte	KA (1/M)	KD (M)	Req (RU)	Kobs (1/s)
0.5 µg/mL SMB to hACE2	6.2 × 10^3^	6.12 × 10^−5^	6.48 × 10^3^	5.89	1.05 × 10^−7^	1.01 × 10^8^	9.87 × 10^−9^	5.93 × 10^3^	7.12 × 10^−4^
0.5 µg/mL B-YL to hACE2	2.19 × 10^4^	2.79 × 10^−5^	258	2.12	1.05 × 10^−7^	7.85 × 10^8^	1.27 × 10^−9^	255	2.33 × 10^−3^

SPR metrics derived from the time course of binding of the ACE2 to the SMB and B-YL peptides. Association and dissociation kinetic rate constants (k_on_, k_off_) and equilibrium dissociation constants (K_D_) calculated from SPR kinetic measurements for the hACE2 were attached to the Biacore sensor chip, while SMB and B-YL peptides were flowed in the anylate. Peptide was dissolved in HBS–EP buffer and flowed past the recombinant protein constructs on the CSM sensor chip with a Biacore system (Methods). Kinetic rate constants and equilibrium dissociation constants were determined from curve-fitting analysis of SPR traces. Ka = Association constant; kd = Dissociation constant; RI = Bulk refractive index contribution; Rmax = Maximum binding capacity; KA = Equilibrium association constant; KD = Equilibrium dissociation constant/affinity; Req = Response at equilibrium; Kobs = “Observed constant” (kobs = ka × concentration+ kd).

## Data Availability

Data is contained within the Supplementary Materials.

## Supplementary Materials

The supporting information can be downloaded at: https://www.mdpi.com/article/10.3390/ijms241310837/s1. References [30,31] are cited in the Supplementary Material.

## References

[1] Walther F.J., Waring A.J. (2021). Synthetic lung surfactant treatment for COVID-19 pneumonia. Coronaviruses.

[2] National Institutes of Health (2023). Coronavirus Disease 2019 (COVID-19) Treatment Guidelines.

[3] Gerard L., Lecocq M., Bouzin C., Hoton D., Schmit G., Pereira J.P., Montiel V., Plante-Bordeneuve T., Laterre P.F., Pilette C. (2021). Increased Angiotensin-Converting Enzyme 2 and Loss of Alveolar Type II Cells in COVID-19-related acute respiratory distress syndrome. Am. J. Respir. Crit. Care Med..

[4] Piva S., DiBlasi R.M., Slee A.E., Jobe A.H., Roccaro A.M., Filippini M., Latronico N., Bertoni M., Marshall J.C., Portman M.A. (2021). Surfactant therapy for COVID-19 related ARDS: A retrospective case-control pilot study. Respir. Res..

[5] Numata M., Kandasamy P., Voelker D.R. (2023). The anti-inflammatory and antiviral properties of anionic pulmonary surfactant phospholipids. Immunol. Rev..

[6] Cattel F., Giordano S., Bertiond C., Lupia T., Corcione S., Scaldaferri M., Angelone L., De Rosa F.G. (2021). Use of exogenous pulmonary surfactant in acute respiratory distress syndrome (ARDS): Role in SARS-CoV-2-related lung injury. Respir. Physiol. Neurobiol..

[7] Walther F.J., Gordon L.M., Waring A.J. (2019). Advances in synthetic lung surfactant protein technology. Expert Rev. Respir. Med..

[8] Walther F.J., Waring A.J., Hernandez-Juviel J.M., Gordon L.M., Wang Z., Jung C.-L., Ruchala P., Clark A.P., Smith W.M., Sharma S. (2010). Critical structural and functional roles for the N-terminal insertion sequence in surfactant protein B analogs. PLoS ONE.

[9] Walther F.J., Gupta M., Gordon L.M., Waring A.J. (2018). A sulfur-free peptide mimic of surfactant protein B (B-YL) exhibits high in vitro and in vivo surface activities. Gates Open Res..

[10] Lan J., Ge J., Yu J., Shan S., Zhou H., Fan S., Zhang Q., Shi X., Wang Q., Zhang L. (2020). Structure of the SARS-CoV-2 spike receptor-binding domain bound to the ACE2 receptor. Nature.

[11] Benton D.J., Wrobel A.G., Xu P., Roustan C., Martin S.R., Rosenthal P.B., Skehel J.J., Gamblin S.J. (2020). Receptor binding and priming of the spike protein of SARS-CoV-2 for membrane fusion. Nature.

[12] Cao L., Goreshnik I., Coventry B., Case J.B., Miller L., Kozodoy L., Chen R.E., Carter L., Walls A.C., Park Y.J. (2020). De novo design of picomolar SARS-CoV-2 miniprotein inhibitors. Science.

[13] Peter E.K., Schug A. (2021). The inhibitory effect of a coronavirus spike protein fragment with ACE2. Biophys. J..

[14] Struck A.W., Axmann M., Pfefferle S., Drosten C., Meyer B. (2012). A hexapeptide of the receptor-binding domain of SARS corona virus spike protein blocks viral entry into host cells via the human receptor ACE2. Antiviral Res..

[15] VanPatten S., He M., Altiti A., FCheng K., Ghanem M.H., Al-Abed Y. (2020). Evidence supporting the use of peptides and peptidomimetics as potential SARS-CoV-2 (COVID-19) therapeutics. Future Med. Chem..

[16] Sadremomtaz A., Al-Dahmani Z.M., Ruiz-Moreno A.J., Monti A., Wang C., Azad T., Bell J.C., Doti N., Velasco-Velázquez M.A., de Jong D. (2022). Synthetic peptides that antagonize the angiotensin-converting enzyme-2 (ACE-2) interaction with SARS-CoV-2 receptor binding spike protein. J. Med. Chem..

[17] Xue L.C., Rodrigues J.P., Kastritis P.L., Bonvin A.M., Vangone A. (2016). PRODIGY: A web server for predicting the binding affinity of protein-protein complexes. Bioinformatics.

[18] van Zundert G.C.P., Rodrigues J.P.G.L.M., Trellet M., Schmitz C., Kastritis P.L., Karaca E., Melquiond A.S.J., van Dijk M., de Vries S.J., Bonvin A.M.J.J. (2016). The HADDOCK2.2 web server: User-friendly integrative modeling of biomolecular complexes. J. Mol. Biol..

[19] Patching S.G. (2014). Surface plasmon resonance spectroscopy for characterisation of membrane protein-ligand interactions and its potential for drug discovery. Biochim. Biophys. Acta.

[20] Graña C., Ghosn L., Evrenoglou T., Jarde A., Minozzi S., Bergman H., Buckley B.S., Probyn K., Villanueva G., Henschke N. (2022). Efficacy and safety of COVID-19 vaccines. Cochrane Database Syst. Rev..

[21] Day C.J., Bailly B., Guillon P., Dirr L., Jen F.E., Spillings B.L., Mak J., von Itzstein M., Haselhorst T., Jennings M.P. (2021). Multidisciplinary approaches identify compounds that bind to human ACE2 or SARS-CoV-2 spike protein as candidates to block SARS-CoV-2-ACE2 receptor interactions. mBio.

[22] Das A., Vishvakarma V., Dey A., Dey S., Gupta A., Das M., Vishwakarma K.K., Roy D.S., Yadav S., Kesarwani S. (2021). Biophysical properties of the isolated spike protein binding helix of human ACE2. Biophys. J..

[23] Walther F.J., Sharma S., Gordon L.M., Waring A.J. (2021). Structural and functional stability of the sulfur-free surfactant protein B peptide mimic B-YL in synthetic surfactant lipids. BMC Pulm. Med..

[24] Waring A.J., Whitelegge J.P., Sharma S.K., Gordon L.M., Walther F.J. (2022). Emulation of the structure of the Saposin protein fold by a lung surfactant peptide construct of surfactant Protein B. PLoS ONE.

[25] Rozak H., Nihonyanagi S., Myalitsin A., Roy S., Ahmed M., Tahara T., Rzeznicka I.I. (2023). Adsorption of SARS-CoV-2 Spike (N501Y) RBD to Human Angiotensin-Converting Enzyme 2 at a Lipid/Water Interface. J. Phys. Chem. B.

[26] Honorato R.V., Koukos P.I., Jiménez-García B., Tsaregorodtsev A., Verlato M., Giachetti A., Rosato A., Bonvin A.M.J.J. (2021). Structural biology in the clouds: The WeNMR-EOSC ecosystem. Front. Mol. Biosci..

[27] Vangone A., Bonvin A.M. (2015). Contacts-based prediction of binding affinity in protein-protein complexes. Elife.

[28] Tam J.P., Dong X.C., Wu C.R. (1999). Solvent assistance in regiospecific disulfide formation in dimethylsulfoxide. Lett. Peptide Sci..

[29] Anthis N.J., Clore G.M. (2013). Sequence-specific determination of protein and peptide concentrations by absorbance at 205 nm. Protein Sci..

[30] Kauppinen J.K., Moffatt D.J., Mantsch H.H., Cameron D.G. (1981). Fourier self-deconvolution: A method for resolving intrinsically overlapped bands. Appl. Spectr..

[31] Byler D.M., Susi H. (1986). Examination of the secondary structure of protein by deconvolved FTIR spectra. Biopolymers.

