# Supplementary material for: Lung Surfactant Protein B Peptide Mimics Interact with the Human ACE2 Receptor
Source: Int J Mol Sci. 2023 Jun 29;24(13):10837. doi: 10.3390/ijms241310837 (PMC10341807; doi:10.3390/ijms241310837)
Supplement: Supplementary file 1 [file ijms-24-10837-s001.zip › File S1_SMB_ACE2_complex.pdf]

S1. Supplement file – ACE2 - SMB Haddock Docked Molecular Coordinates and Prodigy Binding Analysis of the predicted complex.

Haddock Webserver (version 2.4)

https://wenmr.science.uu.nl/haddock2.4/submit/1

Haddock Predicted Molecular Coordinates of the ACE2 – B-YL Complex based on docking the B-YL helical domains and N-terminal insertion sequences with the ACE2 contact residues of the Covid Receptor Binding Domain.

Summary

HADDOCK clustered **150** structures in **19** cluster(s), which represents **75 %** of the water-refined models HADDOCK generated. Note that currently the maximum number of models considered for clustering is 200.

The top cluster detailed below is the most reliable according to HADDOCK. Its Z-score indicates how many standard deviations from the average this cluster is located in terms of score (the more negative the better).

Best Docked Complex Structure

HADDOCK score	64.7 ± 1.8
Cluster size	10
RMSD from the overall lowest-energy structure	4.3 ± 0.1
Van der Waals energy	-44.1 ± 8.0
Electrostatic energy	-233.7 ± 49.2
Desolvation energy	0.7 ± 2.4
Restraints violation energy	255.3 ± 31.2
Buried Surface Area	1346.4 ± 57.7
Z-Score	-1.9

ACE2 (Chain A) SMB (Chain B)

ATOM	1	N	SER	A	19	-37.272	11.016	15.120	1.00	15.00	A	N
ATOM	2	CA	SER	A	19	-37.419	12.463	15.125	1.00	15.00	A	C
ATOM	3	CB	SER	A	19	-38.508	12.875	16.115	1.00	15.00	A	C
ATOM	4	OG	SER	A	19	-39.647	12.047	15.964	1.00	15.00	A	O
ATOM	5	C	SER	A	19	-36.098	13.154	15.459	1.00	15.00	A	C
ATOM	6	O	SER	A	19	-35.807	14.240	14.949	1.00	15.00	A	O
ATOM	7	N	THR	A	20	-35.296	12.516	16.303	1.00	15.00	A	N
ATOM	8	CA	THR	A	20	-34.010	13.069	16.699	1.00	15.00	A	C
ATOM	9	CB	THR	A	20	-33.424	12.331	17.918	1.00	15.00	A	C
ATOM	10	OG1	THR	A	20	-33.248	10.945	17.601	1.00	15.00	A	O
ATOM	11	CG2	THR	A	20	-34.349	12.460	19.120	1.00	15.00	A	C
ATOM	12	C	THR	A	20	-33.019	12.988	15.544	1.00	15.00	A	C
ATOM	13	O	THR	A	20	-33.233	12.245	14.582	1.00	15.00	A	O
ATOM	14	N	ILE	A	21	-31.935	13.743	15.648	1.00	15.00	A	N
ATOM	15	CA	ILE	A	21	-30.910	13.770	14.613	1.00	15.00	A	C
ATOM	16	CB	ILE	A	21	-29.745	14.702	15.004	1.00	15.00	A	C
ATOM	17	CG1	ILE	A	21	-30.259	16.129	15.218	1.00	15.00	A	C
ATOM	18	CG2	ILE	A	21	-28.643	14.679	13.954	1.00	15.00	A	C
ATOM	19	CD1	ILE	A	21	-30.936	16.737	14.006	1.00	15.00	A	C
ATOM	20	C	ILE	A	21	-30.385	12.368	14.302	1.00	15.00	A	C
ATOM	21	O	ILE	A	21	-30.218	12.009	13.138	1.00	15.00	A	O
ATOM	22	N	GLU	A	22	-30.160	11.569	15.340	1.00	15.00	A	N
ATOM	23	CA	GLU	A	22	-29.655	10.212	15.160	1.00	15.00	A	C
ATOM	24	CB	GLU	A	22	-29.337	9.557	16.503	1.00	15.00	A	C
ATOM	25	CG	GLU	A	22	-28.501	8.295	16.378	1.00	15.00	A	C
ATOM	26	CD	GLU	A	22	-28.459	7.491	17.658	1.00	15.00	A	C
ATOM	27	OE1	GLU	A	22	-27.586	7.767	18.504	1.00	15.00	A	O
ATOM	28	OE2	GLU	A	22	-29.297	6.574	17.810	1.00	15.00	A	O
ATOM	29	C	GLU	A	22	-30.637	9.351	14.368	1.00	15.00	A	C
ATOM	30	O	GLU	A	22	-30.239	8.609	13.471	1.00	15.00	A	O
ATOM	31	N	GLU	A	23	-31.919	9.452	14.700	1.00	15.00	A	N
ATOM	32	CA	GLU	A	23	-32.947	8.681	14.009	1.00	15.00	A	C
ATOM	33	CB	GLU	A	23	-34.302	8.858	14.689	1.00	15.00	A	C
ATOM	34	CG	GLU	A	23	-34.363	8.300	16.099	1.00	15.00	A	C
ATOM	35	CD	GLU	A	23	-35.582	8.788	16.850	1.00	15.00	A	C
ATOM	36	OE1	GLU	A	23	-36.467	7.968	17.141	1.00	15.00	A	O
ATOM	37	OE2	GLU	A	23	-35.649	10.003	17.141	1.00	15.00	A	O
ATOM	38	C	GLU	A	23	-33.037	9.103	12.549	1.00	15.00	A	C
ATOM	39	O	GLU	A	23	-33.101	8.264	11.650	1.00	15.00	A	O
ATOM	40	N	GLN	A	24	-33.015	10.409	12.324	1.00	15.00	A	N
ATOM	41	CA	GLN	A	24	-33.090	10.953	10.977	1.00	15.00	A	C
ATOM	42	CB	GLN	A	24	-33.278	12.467	11.013	1.00	15.00	A	C
ATOM	43	CG	GLN	A	24	-34.487	12.923	11.811	1.00	15.00	A	C
ATOM	44	CD	GLN	A	24	-35.063	14.223	11.295	1.00	15.00	A	C
ATOM	45	OE1	GLN	A	24	-35.051	14.483	10.090	1.00	15.00	A	O
ATOM	46	NE2	GLN	A	24	-35.563	15.051	12.200	1.00	15.00	A	N
ATOM	47	C	GLN	A	24	-31.847	10.587	10.173	1.00	15.00	A	C
ATOM	48	O	GLN	A	24	-31.921	10.368	8.961	1.00	15.00	A	O
ATOM	49	N	ALA	A	25	-30.708	10.521	10.854	1.00	15.00	A	N
ATOM	50	CA	ALA	A	25	-29.450	10.164	10.214	1.00	15.00	A	C
ATOM	51	CB	ALA	A	25	-28.293	10.359	11.178	1.00	15.00	A	C
ATOM	52	C	ALA	A	25	-29.497	8.722	9.728	1.00	15.00	A	C
ATOM	53	O	ALA	A	25	-28.979	8.400	8.660	1.00	15.00	A	O
ATOM	54	N	LYS	A	26	-30.137	7.862	10.517	1.00	15.00	A	N
ATOM	55	CA	LYS	A	26	-30.274	6.453	10.169	1.00	15.00	A	C
ATOM	56	CB	LYS	A	26	-31.025	5.699	11.270	1.00	15.00	A	C
ATOM	57	CG	LYS	A	26	-30.233	5.534	12.557	1.00	15.00	A	C
ATOM	58	CD	LYS	A	26	-31.148	5.391	13.763	1.00	15.00	A	C
ATOM	59	CE	LYS	A	26	-30.346	5.407	15.054	1.00	15.00	A	C
ATOM	60	NZ	LYS	A	26	-31.186	5.725	16.242	1.00	15.00	A	N

ATOM	61	C	LYS	A	26	-31.004	6.306	8.840	1.00	15.00	A	C
ATOM	62	O	LYS	A	26	-30.552	5.589	7.948	1.00	15.00	A	O
ATOM	63	N	THR	A	27	-32.124	7.007	8.709	1.00	15.00	A	N
ATOM	64	CA	THR	A	27	-32.910	6.965	7.485	1.00	15.00	A	C
ATOM	65	CB	THR	A	27	-34.276	7.648	7.677	1.00	15.00	A	C
ATOM	66	OG1	THR	A	27	-34.123	8.808	8.510	1.00	15.00	A	O
ATOM	67	CG2	THR	A	27	-35.266	6.691	8.323	1.00	15.00	A	C
ATOM	68	C	THR	A	27	-32.156	7.622	6.328	1.00	15.00	A	C
ATOM	69	O	THR	A	27	-32.378	7.305	5.164	1.00	15.00	A	O
ATOM	70	N	PHE	A	28	-31.258	8.543	6.656	1.00	15.00	A	N
ATOM	71	CA	PHE	A	28	-30.467	9.217	5.639	1.00	15.00	A	C
ATOM	72	CB	PHE	A	28	-29.788	10.463	6.216	1.00	15.00	A	C
ATOM	73	CG	PHE	A	28	-28.870	11.154	5.249	1.00	15.00	A	C
ATOM	74	CD1	PHE	A	28	-29.382	11.882	4.187	1.00	15.00	A	C
ATOM	75	CD2	PHE	A	28	-27.496	11.070	5.398	1.00	15.00	A	C
ATOM	76	CE1	PHE	A	28	-28.541	12.512	3.292	1.00	15.00	A	C
ATOM	77	CE2	PHE	A	28	-26.649	11.699	4.507	1.00	15.00	A	C
ATOM	78	CZ	PHE	A	28	-27.172	12.420	3.452	1.00	15.00	A	C
ATOM	79	C	PHE	A	28	-29.428	8.252	5.083	1.00	15.00	A	C
ATOM	80	O	PHE	A	28	-29.240	8.147	3.870	1.00	15.00	A	O
ATOM	81	N	LEU	A	29	-28.771	7.533	5.987	1.00	15.00	A	N
ATOM	82	CA	LEU	A	29	-27.761	6.559	5.605	1.00	15.00	A	C
ATOM	83	CB	LEU	A	29	-27.009	6.047	6.835	1.00	15.00	A	C
ATOM	84	CG	LEU	A	29	-26.178	7.083	7.595	1.00	15.00	A	C
ATOM	85	CD1	LEU	A	29	-25.598	6.477	8.862	1.00	15.00	A	C
ATOM	86	CD2	LEU	A	29	-25.073	7.643	6.711	1.00	15.00	A	C
ATOM	87	C	LEU	A	29	-28.400	5.402	4.850	1.00	15.00	A	C
ATOM	88	O	LEU	A	29	-27.754	4.763	4.022	1.00	15.00	A	O
ATOM	89	N	ASP	A	30	-29.668	5.139	5.153	1.00	15.00	A	N
ATOM	90	CA	ASP	A	30	-30.422	4.077	4.491	1.00	15.00	A	C
ATOM	91	CB	ASP	A	30	-31.830	3.964	5.111	1.00	15.00	A	C
ATOM	92	CG	ASP	A	30	-32.977	4.010	4.110	1.00	15.00	A	C
ATOM	93	OD1	ASP	A	30	-33.140	3.047	3.342	1.00	15.00	A	O
ATOM	94	OD2	ASP	A	30	-33.746	4.996	4.117	1.00	15.00	A	O
ATOM	95	C	ASP	A	30	-30.492	4.352	2.993	1.00	15.00	A	C
ATOM	96	O	ASP	A	30	-30.142	3.500	2.171	1.00	15.00	A	O
ATOM	97	N	LYS	A	31	-30.902	5.569	2.656	1.00	15.00	A	N
ATOM	98	CA	LYS	A	31	-31.015	5.988	1.272	1.00	15.00	A	C
ATOM	99	CB	LYS	A	31	-31.661	7.375	1.203	1.00	15.00	A	C
ATOM	100	CG	LYS	A	31	-31.970	7.859	-0.203	1.00	15.00	A	C
ATOM	101	CD	LYS	A	31	-32.773	9.149	-0.174	1.00	15.00	A	C
ATOM	102	CE	LYS	A	31	-33.150	9.608	-1.574	1.00	15.00	A	C
ATOM	103	NZ	LYS	A	31	-33.728	8.507	-2.382	1.00	15.00	A	N
ATOM	104	C	LYS	A	31	-29.642	6.008	0.608	1.00	15.00	A	C
ATOM	105	O	LYS	A	31	-29.471	5.504	-0.501	1.00	15.00	A	O
ATOM	106	N	PHE	A	32	-28.662	6.568	1.310	1.00	15.00	A	N
ATOM	107	CA	PHE	A	32	-27.303	6.661	0.791	1.00	15.00	A	C
ATOM	108	CB	PHE	A	32	-26.400	7.447	1.747	1.00	15.00	A	C
ATOM	109	CG	PHE	A	32	-24.960	7.477	1.317	1.00	15.00	A	C
ATOM	110	CD1	PHE	A	32	-24.554	8.281	0.267	1.00	15.00	A	C
ATOM	111	CD2	PHE	A	32	-24.014	6.692	1.956	1.00	15.00	A	C
ATOM	112	CE1	PHE	A	32	-23.234	8.304	-0.140	1.00	15.00	A	C
ATOM	113	CE2	PHE	A	32	-22.692	6.712	1.555	1.00	15.00	A	C
ATOM	114	CZ	PHE	A	32	-22.301	7.518	0.505	1.00	15.00	A	C
ATOM	115	C	PHE	A	32	-26.700	5.286	0.511	1.00	15.00	A	C
ATOM	116	O	PHE	A	32	-26.120	5.065	-0.549	1.00	15.00	A	O
ATOM	117	N	ASN	A	33	-26.844	4.367	1.461	1.00	15.00	A	N
ATOM	118	CA	ASN	A	33	-26.297	3.018	1.318	1.00	15.00	A	C
ATOM	119	CB	ASN	A	33	-26.602	2.165	2.556	1.00	15.00	A	C
ATOM	120	CG	ASN	A	33	-25.479	2.169	3.578	1.00	15.00	A	C
ATOM	121	OD1	ASN	A	33	-24.560	1.352	3.513	1.00	15.00	A	O
ATOM	122	ND2	ASN	A	33	-25.551	3.081	4.536	1.00	15.00	A	N
ATOM	123	C	ASN	A	33	-26.836	2.329	0.073	1.00	15.00	A	C
ATOM	124	O	ASN	A	33	-26.077	1.758	-0.711	1.00	15.00	A	O
ATOM	125	N	HIS	A	34	-28.145	2.405	-0.112	1.00	15.00	A	N
ATOM	126	CA	HIS	A	34	-28.794	1.782	-1.255	1.00	15.00	A	C
ATOM	127	CB	HIS	A	34	-30.309	1.764	-1.050	1.00	15.00	A	C
ATOM	128	CG	HIS	A	34	-31.010	0.642	-1.751	1.00	15.00	A	C
ATOM	129	ND1	HIS	A	34	-31.520	-0.466	-1.110	1.00	15.00	A	N
ATOM	130	CD2	HIS	A	34	-31.293	0.475	-3.066	1.00	15.00	A	C
ATOM	131	CE1	HIS	A	34	-32.083	-1.251	-2.035	1.00	15.00	A	C

ATOM	132	NE2	HIS	A	34	-31.972	-0.725	-3.240	1.00	15.00	A	N
ATOM	133	C	HIS	A	34	-28.436	2.504	-2.553	1.00	15.00	A	C
ATOM	134	O	HIS	A	34	-28.161	1.870	-3.573	1.00	15.00	A	O
ATOM	135	N	GLU	A	35	-28.434	3.832	-2.504	1.00	15.00	A	N
ATOM	136	CA	GLU	A	35	-28.111	4.649	-3.668	1.00	15.00	A	C
ATOM	137	CB	GLU	A	35	-28.329	6.133	-3.351	1.00	15.00	A	C
ATOM	138	CG	GLU	A	35	-29.368	6.827	-4.222	1.00	15.00	A	C
ATOM	139	CD	GLU	A	35	-30.796	6.469	-3.854	1.00	15.00	A	C
ATOM	140	OE1	GLU	A	35	-31.194	5.309	-4.086	1.00	15.00	A	O
ATOM	141	OE2	GLU	A	35	-31.530	7.352	-3.364	1.00	15.00	A	O
ATOM	142	C	GLU	A	35	-26.670	4.419	-4.130	1.00	15.00	A	C
ATOM	143	O	GLU	A	35	-26.409	4.237	-5.323	1.00	15.00	A	O
ATOM	144	N	ALA	A	36	-25.747	4.407	-3.175	1.00	15.00	A	N
ATOM	145	CA	ALA	A	36	-24.329	4.218	-3.465	1.00	15.00	A	C
ATOM	146	CB	ALA	A	36	-23.494	4.490	-2.223	1.00	15.00	A	C
ATOM	147	C	ALA	A	36	-24.023	2.829	-4.019	1.00	15.00	A	C
ATOM	148	O	ALA	A	36	-23.199	2.692	-4.922	1.00	15.00	A	O
ATOM	149	N	GLU	A	37	-24.692	1.811	-3.481	1.00	15.00	A	N
ATOM	150	CA	GLU	A	37	-24.487	0.424	-3.909	1.00	15.00	A	C
ATOM	151	CB	GLU	A	37	-25.451	-0.509	-3.162	1.00	15.00	A	C
ATOM	152	CG	GLU	A	37	-25.259	-1.992	-3.450	1.00	15.00	A	C
ATOM	153	CD	GLU	A	37	-24.363	-2.690	-2.443	1.00	15.00	A	C
ATOM	154	OE1	GLU	A	37	-23.175	-2.916	-2.753	1.00	15.00	A	O
ATOM	155	OE2	GLU	A	37	-24.845	-3.031	-1.342	1.00	15.00	A	O
ATOM	156	C	GLU	A	37	-24.646	0.257	-5.425	1.00	15.00	A	C
ATOM	157	O	GLU	A	37	-23.941	-0.537	-6.051	1.00	15.00	A	O
ATOM	158	N	ASP	A	38	-25.563	1.013	-6.015	1.00	15.00	A	N
ATOM	159	CA	ASP	A	38	-25.798	0.932	-7.455	1.00	15.00	A	C
ATOM	160	CB	ASP	A	38	-27.236	1.319	-7.796	1.00	15.00	A	C
ATOM	161	CG	ASP	A	38	-27.672	0.835	-9.167	1.00	15.00	A	C
ATOM	162	OD1	ASP	A	38	-28.268	-0.260	-9.245	1.00	15.00	A	O
ATOM	163	OD2	ASP	A	38	-27.423	1.541	-10.163	1.00	15.00	A	O
ATOM	164	C	ASP	A	38	-24.831	1.819	-8.225	1.00	15.00	A	C
ATOM	165	O	ASP	A	38	-24.126	1.352	-9.118	1.00	15.00	A	O
ATOM	166	N	LEU	A	39	-24.787	3.095	-7.851	1.00	15.00	A	N
ATOM	167	CA	LEU	A	39	-23.924	4.072	-8.512	1.00	15.00	A	C
ATOM	168	CB	LEU	A	39	-24.083	5.451	-7.872	1.00	15.00	A	C
ATOM	169	CG	LEU	A	39	-25.337	6.229	-8.274	1.00	15.00	A	C
ATOM	170	CD1	LEU	A	39	-25.752	7.185	-7.169	1.00	15.00	A	C
ATOM	171	CD2	LEU	A	39	-25.102	6.984	-9.574	1.00	15.00	A	C
ATOM	172	C	LEU	A	39	-22.459	3.645	-8.527	1.00	15.00	A	C
ATOM	173	O	LEU	A	39	-21.775	3.787	-9.544	1.00	15.00	A	O
ATOM	174	N	PHE	A	40	-21.981	3.114	-7.407	1.00	15.00	A	N
ATOM	175	CA	PHE	A	40	-20.600	2.664	-7.314	1.00	15.00	A	C
ATOM	176	CB	PHE	A	40	-20.239	2.284	-5.877	1.00	15.00	A	C
ATOM	177	CG	PHE	A	40	-18.799	1.897	-5.693	1.00	15.00	A	C
ATOM	178	CD1	PHE	A	40	-17.792	2.839	-5.822	1.00	15.00	A	C
ATOM	179	CD2	PHE	A	40	-18.453	0.590	-5.391	1.00	15.00	A	C
ATOM	180	CE1	PHE	A	40	-16.467	2.487	-5.653	1.00	15.00	A	C
ATOM	181	CE2	PHE	A	40	-17.131	0.229	-5.221	1.00	15.00	A	C
ATOM	182	CZ	PHE	A	40	-16.135	1.179	-5.352	1.00	15.00	A	C
ATOM	183	C	PHE	A	40	-20.362	1.487	-8.251	1.00	15.00	A	C
ATOM	184	O	PHE	A	40	-19.329	1.414	-8.918	1.00	15.00	A	O
ATOM	185	N	TYR	A	41	-21.333	0.580	-8.311	1.00	15.00	A	N
ATOM	186	CA	TYR	A	41	-21.238	-0.589	-9.175	1.00	15.00	A	C
ATOM	187	CB	TYR	A	41	-22.461	-1.497	-8.992	1.00	15.00	A	C
ATOM	188	CG	TYR	A	41	-22.395	-2.790	-9.778	1.00	15.00	A	C
ATOM	189	CD1	TYR	A	41	-21.437	-3.750	-9.489	1.00	15.00	A	C
ATOM	190	CD2	TYR	A	41	-23.289	-3.049	-10.810	1.00	15.00	A	C
ATOM	191	CE1	TYR	A	41	-21.366	-4.931	-10.201	1.00	15.00	A	C
ATOM	192	CE2	TYR	A	41	-23.227	-4.230	-11.528	1.00	15.00	A	C
ATOM	193	CZ	TYR	A	41	-22.261	-5.167	-11.219	1.00	15.00	A	C
ATOM	194	OH	TYR	A	41	-22.193	-6.343	-11.929	1.00	15.00	A	O
ATOM	195	C	TYR	A	41	-21.117	-0.157	-10.631	1.00	15.00	A	C
ATOM	196	O	TYR	A	41	-20.276	-0.665	-11.371	1.00	15.00	A	O
ATOM	197	N	GLN	A	42	-21.949	0.802	-11.023	1.00	15.00	A	N
ATOM	198	CA	GLN	A	42	-21.943	1.320	-12.386	1.00	15.00	A	C
ATOM	199	CB	GLN	A	42	-23.067	2.345	-12.570	1.00	15.00	A	C
ATOM	200	CG	GLN	A	42	-24.440	1.839	-12.154	1.00	15.00	A	C
ATOM	201	CD	GLN	A	42	-25.087	0.941	-13.190	1.00	15.00	A	C
ATOM	202	OE1	GLN	A	42	-24.410	0.329	-14.016	1.00	15.00	A	O

ATOM	203	NE2	GLN	A	42	-26.405	0.837	-13.135	1.00	15.00	A	N
ATOM	204	C	GLN	A	42	-20.595	1.956	-12.717	1.00	15.00	A	C
ATOM	205	O	GLN	A	42	-20.002	1.676	-13.761	1.00	15.00	A	O
ATOM	206	N	SER	A	43	-20.109	2.799	-11.811	1.00	15.00	A	N
ATOM	207	CA	SER	A	43	-18.831	3.472	-11.997	1.00	15.00	A	C
ATOM	208	CB	SER	A	43	-18.579	4.466	-10.857	1.00	15.00	A	C
ATOM	209	OG	SER	A	43	-17.471	5.304	-11.139	1.00	15.00	A	O
ATOM	210	C	SER	A	43	-17.691	2.458	-12.084	1.00	15.00	A	C
ATOM	211	O	SER	A	43	-16.804	2.576	-12.934	1.00	15.00	A	O
ATOM	212	N	SER	A	44	-17.727	1.459	-11.212	1.00	15.00	A	N
ATOM	213	CA	SER	A	44	-16.703	0.426	-11.192	1.00	15.00	A	C
ATOM	214	CB	SER	A	44	-16.832	-0.428	-9.933	1.00	15.00	A	C
ATOM	215	OG	SER	A	44	-16.840	0.391	-8.777	1.00	15.00	A	O
ATOM	216	C	SER	A	44	-16.770	-0.439	-12.447	1.00	15.00	A	C
ATOM	217	O	SER	A	44	-15.739	-0.833	-12.993	1.00	15.00	A	O
ATOM	218	N	LEU	A	45	-17.986	-0.720	-12.909	1.00	15.00	A	N
ATOM	219	CA	LEU	A	45	-18.184	-1.523	-14.108	1.00	15.00	A	C
ATOM	220	CB	LEU	A	45	-19.675	-1.760	-14.356	1.00	15.00	A	C
ATOM	221	CG	LEU	A	45	-20.027	-2.793	-15.429	1.00	15.00	A	C
ATOM	222	CD1	LEU	A	45	-19.639	-4.193	-14.975	1.00	15.00	A	C
ATOM	223	CD2	LEU	A	45	-21.508	-2.728	-15.769	1.00	15.00	A	C
ATOM	224	C	LEU	A	45	-17.553	-0.829	-15.308	1.00	15.00	A	C
ATOM	225	O	LEU	A	45	-16.859	-1.456	-16.108	1.00	15.00	A	O
ATOM	226	N	ALA	A	46	-17.789	0.475	-15.414	1.00	15.00	A	N
ATOM	227	CA	ALA	A	46	-17.230	1.267	-16.502	1.00	15.00	A	C
ATOM	228	CB	ALA	A	46	-17.787	2.681	-16.470	1.00	15.00	A	C
ATOM	229	C	ALA	A	46	-15.708	1.287	-16.403	1.00	15.00	A	C
ATOM	230	O	ALA	A	46	-15.006	1.199	-17.412	1.00	15.00	A	O
ATOM	231	N	SER	A	47	-15.210	1.393	-15.175	1.00	15.00	A	N
ATOM	232	CA	SER	A	47	-13.777	1.406	-14.928	1.00	15.00	A	C
ATOM	233	CB	SER	A	47	-13.494	1.676	-13.445	1.00	15.00	A	C
ATOM	234	OG	SER	A	47	-12.113	1.898	-13.212	1.00	15.00	A	O
ATOM	235	C	SER	A	47	-13.157	0.079	-15.361	1.00	15.00	A	C
ATOM	236	O	SER	A	47	-12.098	0.056	-15.989	1.00	15.00	A	O
ATOM	237	N	TRP	A	48	-13.831	-1.023	-15.033	1.00	15.00	A	N
ATOM	238	CA	TRP	A	48	-13.353	-2.350	-15.402	1.00	15.00	A	C
ATOM	239	CB	TRP	A	48	-14.258	-3.445	-14.816	1.00	15.00	A	C
ATOM	240	CG	TRP	A	48	-13.909	-4.831	-15.284	1.00	15.00	A	C
ATOM	241	CD1	TRP	A	48	-14.347	-5.448	-16.423	1.00	15.00	A	C
ATOM	242	CD2	TRP	A	48	-13.046	-5.770	-14.630	1.00	15.00	A	C
ATOM	243	NE1	TRP	A	48	-13.806	-6.705	-16.521	1.00	15.00	A	N
ATOM	244	CE2	TRP	A	48	-13.004	-6.928	-15.433	1.00	15.00	A	C
ATOM	245	CE3	TRP	A	48	-12.303	-5.744	-13.447	1.00	15.00	A	C
ATOM	246	CZ2	TRP	A	48	-12.248	-8.046	-15.090	1.00	15.00	A	C
ATOM	247	CZ3	TRP	A	48	-11.555	-6.855	-13.109	1.00	15.00	A	C
ATOM	248	CH2	TRP	A	48	-11.532	-7.989	-13.927	1.00	15.00	A	C
ATOM	249	C	TRP	A	48	-13.283	-2.483	-16.916	1.00	15.00	A	C
ATOM	250	O	TRP	A	48	-12.285	-2.957	-17.458	1.00	15.00	A	O
ATOM	251	N	ASN	A	49	-14.346	-2.049	-17.589	1.00	15.00	A	N
ATOM	252	CA	ASN	A	49	-14.417	-2.116	-19.046	1.00	15.00	A	C
ATOM	253	CB	ASN	A	49	-15.700	-1.464	-19.570	1.00	15.00	A	C
ATOM	254	CG	ASN	A	49	-16.928	-2.336	-19.376	1.00	15.00	A	C
ATOM	255	OD1	ASN	A	49	-16.831	-3.559	-19.287	1.00	15.00	A	O
ATOM	256	ND2	ASN	A	49	-18.095	-1.712	-19.319	1.00	15.00	A	N
ATOM	257	C	ASN	A	49	-13.197	-1.470	-19.681	1.00	15.00	A	C
ATOM	258	O	ASN	A	49	-12.523	-2.080	-20.511	1.00	15.00	A	O
ATOM	259	N	TYR	A	50	-12.901	-0.244	-19.267	1.00	15.00	A	N
ATOM	260	CA	TYR	A	50	-11.751	0.481	-19.789	1.00	15.00	A	C
ATOM	261	CB	TYR	A	50	-11.765	1.937	-19.306	1.00	15.00	A	C
ATOM	262	CG	TYR	A	50	-10.438	2.648	-19.469	1.00	15.00	A	C
ATOM	263	CD1	TYR	A	50	-9.948	2.970	-20.728	1.00	15.00	A	C
ATOM	264	CD2	TYR	A	50	-9.668	2.985	-18.362	1.00	15.00	A	C
ATOM	265	CE1	TYR	A	50	-8.732	3.606	-20.877	1.00	15.00	A	C
ATOM	266	CE2	TYR	A	50	-8.453	3.624	-18.502	1.00	15.00	A	C
ATOM	267	CZ	TYR	A	50	-7.989	3.931	-19.763	1.00	15.00	A	C
ATOM	268	OH	TYR	A	50	-6.776	4.565	-19.910	1.00	15.00	A	O
ATOM	269	C	TYR	A	50	-10.443	-0.195	-19.382	1.00	15.00	A	C
ATOM	270	O	TYR	A	50	-9.504	-0.278	-20.169	1.00	15.00	A	O
ATOM	271	N	ASN	A	51	-10.397	-0.693	-18.155	1.00	15.00	A	N
ATOM	272	CA	ASN	A	51	-9.199	-1.345	-17.638	1.00	15.00	A	C
ATOM	273	CB	ASN	A	51	-9.314	-1.607	-16.136	1.00	15.00	A	C

ATOM	274	CG	ASN	A	51	-8.719	-0.486	-15.304	1.00	15.00	A	C
ATOM	275	OD1	ASN	A	51	-7.539	-0.509	-14.966	1.00	15.00	A	O
ATOM	276	ND2	ASN	A	51	-9.533	0.504	-14.970	1.00	15.00	A	N
ATOM	277	C	ASN	A	51	-8.859	-2.628	-18.390	1.00	15.00	A	C
ATOM	278	O	ASN	A	51	-7.690	-2.998	-18.496	1.00	15.00	A	O
ATOM	279	N	THR	A	52	-9.875	-3.304	-18.912	1.00	15.00	A	N
ATOM	280	CA	THR	A	52	-9.658	-4.541	-19.655	1.00	15.00	A	C
ATOM	281	CB	THR	A	52	-10.691	-5.624	-19.284	1.00	15.00	A	C
ATOM	282	OG1	THR	A	52	-12.019	-5.088	-19.366	1.00	15.00	A	O
ATOM	283	CG2	THR	A	52	-10.440	-6.135	-17.875	1.00	15.00	A	C
ATOM	284	C	THR	A	52	-9.675	-4.308	-21.166	1.00	15.00	A	C
ATOM	285	O	THR	A	52	-9.422	-5.223	-21.950	1.00	15.00	A	O
ATOM	286	N	ASN	A	53	-9.971	-3.079	-21.565	1.00	15.00	A	N
ATOM	287	CA	ASN	A	53	-10.029	-2.719	-22.977	1.00	15.00	A	C
ATOM	288	CB	ASN	A	53	-11.360	-3.165	-23.593	1.00	15.00	A	C
ATOM	289	CG	ASN	A	53	-11.422	-2.956	-25.095	1.00	15.00	A	C
ATOM	290	OD1	ASN	A	53	-10.399	-2.917	-25.775	1.00	15.00	A	O
ATOM	291	ND2	ASN	A	53	-12.630	-2.836	-25.626	1.00	15.00	A	N
ATOM	292	C	ASN	A	53	-9.840	-1.216	-23.136	1.00	15.00	A	C
ATOM	293	O	ASN	A	53	-10.805	-0.448	-23.120	1.00	15.00	A	O
ATOM	294	N	ILE	A	54	-8.587	-0.806	-23.264	1.00	15.00	A	N
ATOM	295	CA	ILE	A	54	-8.238	0.602	-23.403	1.00	15.00	A	C
ATOM	296	CB	ILE	A	54	-6.726	0.838	-23.186	1.00	15.00	A	C
ATOM	297	CG1	ILE	A	54	-6.291	0.259	-21.835	1.00	15.00	A	C
ATOM	298	CG2	ILE	A	54	-6.397	2.324	-23.260	1.00	15.00	A	C
ATOM	299	CD1	ILE	A	54	-4.793	0.266	-21.614	1.00	15.00	A	C
ATOM	300	C	ILE	A	54	-8.664	1.174	-24.754	1.00	15.00	A	C
ATOM	301	O	ILE	A	54	-7.976	1.010	-25.762	1.00	15.00	A	O
ATOM	302	N	THR	A	55	-9.815	1.829	-24.764	1.00	15.00	A	N
ATOM	303	CA	THR	A	55	-10.340	2.457	-25.965	1.00	15.00	A	C
ATOM	304	CB	THR	A	55	-11.480	1.625	-26.592	1.00	15.00	A	C
ATOM	305	OG1	THR	A	55	-12.473	1.333	-25.599	1.00	15.00	A	O
ATOM	306	CG2	THR	A	55	-10.950	0.325	-27.180	1.00	15.00	A	C
ATOM	307	C	THR	A	55	-10.870	3.841	-25.607	1.00	15.00	A	C
ATOM	308	O	THR	A	55	-11.313	4.056	-24.479	1.00	15.00	A	O
ATOM	309	N	GLU	A	56	-10.822	4.772	-26.561	1.00	15.00	A	N
ATOM	310	CA	GLU	A	56	-11.299	6.138	-26.334	1.00	15.00	A	C
ATOM	311	CB	GLU	A	56	-11.198	6.956	-27.626	1.00	15.00	A	C
ATOM	312	CG	GLU	A	56	-11.426	8.448	-27.440	1.00	15.00	A	C
ATOM	313	CD	GLU	A	56	-10.517	9.041	-26.383	1.00	15.00	A	C
ATOM	314	OE1	GLU	A	56	-9.283	8.980	-26.562	1.00	15.00	A	O
ATOM	315	OE2	GLU	A	56	-11.038	9.559	-25.375	1.00	15.00	A	O
ATOM	316	C	GLU	A	56	-12.738	6.137	-25.820	1.00	15.00	A	C
ATOM	317	O	GLU	A	56	-13.085	6.867	-24.890	1.00	15.00	A	O
ATOM	318	N	GLU	A	57	-13.564	5.289	-26.419	1.00	15.00	A	N
ATOM	319	CA	GLU	A	57	-14.961	5.171	-26.028	1.00	15.00	A	C
ATOM	320	CB	GLU	A	57	-15.664	4.150	-26.923	1.00	15.00	A	C
ATOM	321	CG	GLU	A	57	-17.118	3.894	-26.566	1.00	15.00	A	C
ATOM	322	CD	GLU	A	57	-17.709	2.750	-27.360	1.00	15.00	A	C
ATOM	323	OE1	GLU	A	57	-18.851	2.889	-27.838	1.00	15.00	A	O
ATOM	324	OE2	GLU	A	57	-17.021	1.716	-27.508	1.00	15.00	A	O
ATOM	325	C	GLU	A	57	-15.074	4.760	-24.562	1.00	15.00	A	C
ATOM	326	O	GLU	A	57	-15.891	5.301	-23.813	1.00	15.00	A	O
ATOM	327	N	ASN	A	58	-14.231	3.818	-24.151	1.00	15.00	A	N
ATOM	328	CA	ASN	A	58	-14.236	3.336	-22.777	1.00	15.00	A	C
ATOM	329	CB	ASN	A	58	-13.396	2.068	-22.624	1.00	15.00	A	C
ATOM	330	CG	ASN	A	58	-14.242	0.810	-22.602	1.00	15.00	A	C
ATOM	331	OD1	ASN	A	58	-15.410	0.837	-22.220	1.00	15.00	A	O
ATOM	332	ND2	ASN	A	58	-13.651	-0.306	-22.997	1.00	15.00	A	N
ATOM	333	C	ASN	A	58	-13.754	4.411	-21.817	1.00	15.00	A	C
ATOM	334	O	ASN	A	58	-14.251	4.513	-20.698	1.00	15.00	A	O
ATOM	335	N	VAL	A	59	-12.789	5.214	-22.263	1.00	15.00	A	N
ATOM	336	CA	VAL	A	59	-12.260	6.301	-21.444	1.00	15.00	A	C
ATOM	337	CB	VAL	A	59	-11.182	7.119	-22.194	1.00	15.00	A	C
ATOM	338	CG1	VAL	A	59	-10.649	8.243	-21.317	1.00	15.00	A	C
ATOM	339	CG2	VAL	A	59	-10.045	6.226	-22.658	1.00	15.00	A	C
ATOM	340	C	VAL	A	59	-13.401	7.232	-21.061	1.00	15.00	A	C
ATOM	341	O	VAL	A	59	-13.644	7.489	-19.882	1.00	15.00	A	O
ATOM	342	N	GLN	A	60	-14.121	7.703	-22.073	1.00	15.00	A	N
ATOM	343	CA	GLN	A	60	-15.249	8.595	-21.861	1.00	15.00	A	C
ATOM	344	CB	GLN	A	60	-15.811	9.094	-23.193	1.00	15.00	A	C

ATOM	345	CG	GLN	A	60	-15.251	10.439	-23.625	1.00	15.00	A	C
ATOM	346	CD	GLN	A	60	-15.636	11.555	-22.673	1.00	15.00	A	C
ATOM	347	OE1	GLN	A	60	-14.955	11.804	-21.680	1.00	15.00	A	O
ATOM	348	NE2	GLN	A	60	-16.732	12.239	-22.972	1.00	15.00	A	N
ATOM	349	C	GLN	A	60	-16.339	7.922	-21.035	1.00	15.00	A	C
ATOM	350	O	GLN	A	60	-16.949	8.552	-20.176	1.00	15.00	A	O
ATOM	351	N	ASN	A	61	-16.572	6.638	-21.291	1.00	15.00	A	N
ATOM	352	CA	ASN	A	61	-17.584	5.884	-20.552	1.00	15.00	A	C
ATOM	353	CB	ASN	A	61	-17.734	4.467	-21.109	1.00	15.00	A	C
ATOM	354	CG	ASN	A	61	-18.908	4.333	-22.061	1.00	15.00	A	C
ATOM	355	OD1	ASN	A	61	-20.064	4.370	-21.647	1.00	15.00	A	O
ATOM	356	ND2	ASN	A	61	-18.619	4.168	-23.342	1.00	15.00	A	N
ATOM	357	C	ASN	A	61	-17.234	5.831	-19.069	1.00	15.00	A	C
ATOM	358	O	ASN	A	61	-18.096	6.004	-18.207	1.00	15.00	A	O
ATOM	359	N	MET	A	62	-15.957	5.603	-18.786	1.00	15.00	A	N
ATOM	360	CA	MET	A	62	-15.470	5.540	-17.415	1.00	15.00	A	C
ATOM	361	CB	MET	A	62	-14.016	5.057	-17.395	1.00	15.00	A	C
ATOM	362	CG	MET	A	62	-13.355	5.095	-16.027	1.00	15.00	A	C
ATOM	363	SD	MET	A	62	-11.668	4.464	-16.061	1.00	15.00	A	S
ATOM	364	CE	MET	A	62	-11.111	4.894	-14.415	1.00	15.00	A	C
ATOM	365	C	MET	A	62	-15.585	6.907	-16.751	1.00	15.00	A	C
ATOM	366	O	MET	A	62	-16.004	7.015	-15.596	1.00	15.00	A	O
ATOM	367	N	ASN	A	63	-15.226	7.948	-17.496	1.00	15.00	A	N
ATOM	368	CA	ASN	A	63	-15.292	9.318	-16.991	1.00	15.00	A	C
ATOM	369	CB	ASN	A	63	-14.692	10.309	-17.999	1.00	15.00	A	C
ATOM	370	CG	ASN	A	63	-13.202	10.116	-18.223	1.00	15.00	A	C
ATOM	371	OD1	ASN	A	63	-12.494	9.584	-17.369	1.00	15.00	A	O
ATOM	372	ND2	ASN	A	63	-12.714	10.556	-19.375	1.00	15.00	A	N
ATOM	373	C	ASN	A	63	-16.734	9.706	-16.685	1.00	15.00	A	C
ATOM	374	O	ASN	A	63	-17.030	10.243	-15.620	1.00	15.00	A	O
ATOM	375	N	ASN	A	64	-17.628	9.392	-17.619	1.00	15.00	A	N
ATOM	376	CA	ASN	A	64	-19.050	9.702	-17.478	1.00	15.00	A	C
ATOM	377	CB	ASN	A	64	-19.825	9.261	-18.725	1.00	15.00	A	C
ATOM	378	CG	ASN	A	64	-19.712	10.242	-19.876	1.00	15.00	A	C
ATOM	379	OD1	ASN	A	64	-19.665	11.453	-19.678	1.00	15.00	A	O
ATOM	380	ND2	ASN	A	64	-19.667	9.723	-21.095	1.00	15.00	A	N
ATOM	381	C	ASN	A	64	-19.653	9.048	-16.240	1.00	15.00	A	C
ATOM	382	O	ASN	A	64	-20.588	9.577	-15.636	1.00	15.00	A	O
ATOM	383	N	ALA	A	65	-19.129	7.887	-15.873	1.00	15.00	A	N
ATOM	384	CA	ALA	A	65	-19.614	7.171	-14.703	1.00	15.00	A	C
ATOM	385	CB	ALA	A	65	-19.325	5.684	-14.833	1.00	15.00	A	C
ATOM	386	C	ALA	A	65	-18.985	7.732	-13.433	1.00	15.00	A	C
ATOM	387	O	ALA	A	65	-19.670	7.946	-12.430	1.00	15.00	A	O
ATOM	388	N	GLY	A	66	-17.681	7.987	-13.495	1.00	15.00	A	N
ATOM	389	CA	GLY	A	66	-16.961	8.516	-12.352	1.00	15.00	A	C
ATOM	390	C	GLY	A	66	-17.436	9.897	-11.943	1.00	15.00	A	C
ATOM	391	O	GLY	A	66	-17.551	10.189	-10.751	1.00	15.00	A	O
ATOM	392	N	ASP	A	67	-17.713	10.745	-12.928	1.00	15.00	A	N
ATOM	393	CA	ASP	A	67	-18.182	12.103	-12.662	1.00	15.00	A	C
ATOM	394	CB	ASP	A	67	-18.332	12.898	-13.962	1.00	15.00	A	C
ATOM	395	CG	ASP	A	67	-17.224	13.918	-14.152	1.00	15.00	A	C
ATOM	396	OD1	ASP	A	67	-16.450	14.140	-13.197	1.00	15.00	A	O
ATOM	397	OD2	ASP	A	67	-17.133	14.497	-15.255	1.00	15.00	A	O
ATOM	398	C	ASP	A	67	-19.499	12.086	-11.897	1.00	15.00	A	C
ATOM	399	O	ASP	A	67	-19.689	12.847	-10.946	1.00	15.00	A	O
ATOM	400	N	LYS	A	68	-20.400	11.196	-12.302	1.00	15.00	A	N
ATOM	401	CA	LYS	A	68	-21.696	11.070	-11.645	1.00	15.00	A	C
ATOM	402	CB	LYS	A	68	-22.592	10.081	-12.394	1.00	15.00	A	C
ATOM	403	CG	LYS	A	68	-23.067	10.576	-13.749	1.00	15.00	A	C
ATOM	404	CD	LYS	A	68	-24.009	9.578	-14.401	1.00	15.00	A	C
ATOM	405	CE	LYS	A	68	-24.435	10.046	-15.783	1.00	15.00	A	C
ATOM	406	NZ	LYS	A	68	-25.426	9.124	-16.401	1.00	15.00	A	N
ATOM	407	C	LYS	A	68	-21.521	10.624	-10.199	1.00	15.00	A	C
ATOM	408	O	LYS	A	68	-22.190	11.128	-9.297	1.00	15.00	A	O
ATOM	409	N	TRP	A	69	-20.607	9.684	-9.991	1.00	15.00	A	N
ATOM	410	CA	TRP	A	69	-20.321	9.168	-8.660	1.00	15.00	A	C
ATOM	411	CB	TRP	A	69	-19.355	7.981	-8.762	1.00	15.00	A	C
ATOM	412	CG	TRP	A	69	-18.889	7.435	-7.446	1.00	15.00	A	C
ATOM	413	CD1	TRP	A	69	-17.601	7.380	-6.998	1.00	15.00	A	C
ATOM	414	CD2	TRP	A	69	-19.698	6.861	-6.411	1.00	15.00	A	C
ATOM	415	NE1	TRP	A	69	-17.557	6.810	-5.751	1.00	15.00	A	N

ATOM	416	CE2	TRP	A	69	-18.831	6.483	-5.367	1.00	15.00	A	C
ATOM	417	CE3	TRP	A	69	-21.069	6.632	-6.263	1.00	15.00	A	C
ATOM	418	CZ2	TRP	A	69	-19.289	5.889	-4.194	1.00	15.00	A	C
ATOM	419	CZ3	TRP	A	69	-21.520	6.042	-5.099	1.00	15.00	A	C
ATOM	420	CH2	TRP	A	69	-20.634	5.679	-4.079	1.00	15.00	A	C
ATOM	421	C	TRP	A	69	-19.738	10.273	-7.781	1.00	15.00	A	C
ATOM	422	O	TRP	A	69	-20.141	10.445	-6.628	1.00	15.00	A	O
ATOM	423	N	SER	A	70	-18.807	11.033	-8.347	1.00	15.00	A	N
ATOM	424	CA	SER	A	70	-18.169	12.131	-7.633	1.00	15.00	A	C
ATOM	425	CB	SER	A	70	-17.040	12.722	-8.480	1.00	15.00	A	C
ATOM	426	OG	SER	A	70	-16.132	11.709	-8.881	1.00	15.00	A	O
ATOM	427	C	SER	A	70	-19.188	13.209	-7.263	1.00	15.00	A	C
ATOM	428	O	SER	A	70	-19.144	13.772	-6.165	1.00	15.00	A	O
ATOM	429	N	ALA	A	71	-20.107	13.491	-8.183	1.00	15.00	A	N
ATOM	430	CA	ALA	A	71	-21.145	14.485	-7.946	1.00	15.00	A	C
ATOM	431	CB	ALA	A	71	-21.911	14.777	-9.227	1.00	15.00	A	C
ATOM	432	C	ALA	A	71	-22.092	14.006	-6.851	1.00	15.00	A	C
ATOM	433	O	ALA	A	71	-22.464	14.771	-5.960	1.00	15.00	A	O
ATOM	434	N	PHE	A	72	-22.459	12.729	-6.919	1.00	15.00	A	N
ATOM	435	CA	PHE	A	72	-23.351	12.127	-5.935	1.00	15.00	A	C
ATOM	436	CB	PHE	A	72	-23.624	10.660	-6.288	1.00	15.00	A	C
ATOM	437	CG	PHE	A	72	-24.392	9.905	-5.239	1.00	15.00	A	C
ATOM	438	CD1	PHE	A	72	-25.723	10.195	-4.989	1.00	15.00	A	C
ATOM	439	CD2	PHE	A	72	-23.780	8.901	-4.504	1.00	15.00	A	C
ATOM	440	CE1	PHE	A	72	-26.430	9.500	-4.027	1.00	15.00	A	C
ATOM	441	CE2	PHE	A	72	-24.481	8.203	-3.540	1.00	15.00	A	C
ATOM	442	CZ	PHE	A	72	-25.807	8.502	-3.301	1.00	15.00	A	C
ATOM	443	C	PHE	A	72	-22.758	12.232	-4.534	1.00	15.00	A	C
ATOM	444	O	PHE	A	72	-23.445	12.616	-3.586	1.00	15.00	A	O
ATOM	445	N	LEU	A	73	-21.478	11.899	-4.415	1.00	15.00	A	N
ATOM	446	CA	LEU	A	73	-20.787	11.960	-3.134	1.00	15.00	A	C
ATOM	447	CB	LEU	A	73	-19.362	11.420	-3.260	1.00	15.00	A	C
ATOM	448	CG	LEU	A	73	-19.224	9.914	-3.486	1.00	15.00	A	C
ATOM	449	CD1	LEU	A	73	-17.761	9.541	-3.661	1.00	15.00	A	C
ATOM	450	CD2	LEU	A	73	-19.841	9.139	-2.330	1.00	15.00	A	C
ATOM	451	C	LEU	A	73	-20.752	13.387	-2.608	1.00	15.00	A	C
ATOM	452	O	LEU	A	73	-20.909	13.621	-1.412	1.00	15.00	A	O
ATOM	453	N	LYS	A	74	-20.553	14.339	-3.512	1.00	15.00	A	N
ATOM	454	CA	LYS	A	74	-20.500	15.744	-3.139	1.00	15.00	A	C
ATOM	455	CB	LYS	A	74	-20.108	16.612	-4.337	1.00	15.00	A	C
ATOM	456	CG	LYS	A	74	-19.812	18.062	-3.983	1.00	15.00	A	C
ATOM	457	CD	LYS	A	74	-18.986	18.745	-5.063	1.00	15.00	A	C
ATOM	458	CE	LYS	A	74	-17.580	18.166	-5.141	1.00	15.00	A	C
ATOM	459	NZ	LYS	A	74	-16.879	18.227	-3.828	1.00	15.00	A	N
ATOM	460	C	LYS	A	74	-21.830	16.200	-2.550	1.00	15.00	A	C
ATOM	461	O	LYS	A	74	-21.862	16.838	-1.500	1.00	15.00	A	O
ATOM	462	N	GLU	A	75	-22.920	15.852	-3.224	1.00	15.00	A	N
ATOM	463	CA	GLU	A	75	-24.255	16.215	-2.764	1.00	15.00	A	C
ATOM	464	CB	GLU	A	75	-25.303	15.796	-3.803	1.00	15.00	A	C
ATOM	465	CG	GLU	A	75	-26.747	16.052	-3.392	1.00	15.00	A	C
ATOM	466	CD	GLU	A	75	-27.000	17.487	-2.967	1.00	15.00	A	C
ATOM	467	OE1	GLU	A	75	-26.257	18.383	-3.416	1.00	15.00	A	O
ATOM	468	OE2	GLU	A	75	-27.943	17.712	-2.182	1.00	15.00	A	O
ATOM	469	C	GLU	A	75	-24.549	15.566	-1.414	1.00	15.00	A	C
ATOM	470	O	GLU	A	75	-24.983	16.230	-0.470	1.00	15.00	A	O
ATOM	471	N	GLN	A	76	-24.277	14.268	-1.320	1.00	15.00	A	N
ATOM	472	CA	GLN	A	76	-24.512	13.526	-0.088	1.00	15.00	A	C
ATOM	473	CB	GLN	A	76	-24.213	12.039	-0.275	1.00	15.00	A	C
ATOM	474	CG	GLN	A	76	-25.205	11.313	-1.167	1.00	15.00	A	C
ATOM	475	CD	GLN	A	76	-26.644	11.510	-0.734	1.00	15.00	A	C
ATOM	476	OE1	GLN	A	76	-27.153	10.791	0.121	1.00	15.00	A	O
ATOM	477	NE2	GLN	A	76	-27.314	12.480	-1.336	1.00	15.00	A	N
ATOM	478	C	GLN	A	76	-23.693	14.090	1.067	1.00	15.00	A	C
ATOM	479	O	GLN	A	76	-24.150	14.101	2.206	1.00	15.00	A	O
ATOM	480	N	SER	A	77	-22.489	14.559	0.764	1.00	15.00	A	N
ATOM	481	CA	SER	A	77	-21.617	15.132	1.778	1.00	15.00	A	C
ATOM	482	CB	SER	A	77	-20.265	15.522	1.170	1.00	15.00	A	C
ATOM	483	OG	SER	A	77	-19.289	15.738	2.175	1.00	15.00	A	O
ATOM	484	C	SER	A	77	-22.281	16.347	2.421	1.00	15.00	A	C
ATOM	485	O	SER	A	77	-22.345	16.454	3.646	1.00	15.00	A	O
ATOM	486	N	THR	A	78	-22.800	17.240	1.586	1.00	15.00	A	N

ATOM	487	CA	THR	A	78	-23.464	18.445	2.062	1.00	15.00	A	C
ATOM	488	CB	THR	A	78	-23.889	19.336	0.880	1.00	15.00	A	C
ATOM	489	OG1	THR	A	78	-22.953	19.170	-0.194	1.00	15.00	A	O
ATOM	490	CG2	THR	A	78	-23.913	20.799	1.299	1.00	15.00	A	C
ATOM	491	C	THR	A	78	-24.695	18.093	2.897	1.00	15.00	A	C
ATOM	492	O	THR	A	78	-24.910	18.655	3.972	1.00	15.00	A	O
ATOM	493	N	LEU	A	79	-25.487	17.147	2.402	1.00	15.00	A	N
ATOM	494	CA	LEU	A	79	-26.692	16.712	3.099	1.00	15.00	A	C
ATOM	495	CB	LEU	A	79	-27.505	15.758	2.221	1.00	15.00	A	C
ATOM	496	CG	LEU	A	79	-28.053	16.332	0.913	1.00	15.00	A	C
ATOM	497	CD1	LEU	A	79	-28.700	15.235	0.082	1.00	15.00	A	C
ATOM	498	CD2	LEU	A	79	-29.048	17.448	1.189	1.00	15.00	A	C
ATOM	499	C	LEU	A	79	-26.346	16.034	4.423	1.00	15.00	A	C
ATOM	500	O	LEU	A	79	-27.069	16.168	5.409	1.00	15.00	A	O
ATOM	501	N	ALA	A	80	-25.235	15.309	4.438	1.00	15.00	A	N
ATOM	502	CA	ALA	A	80	-24.789	14.614	5.638	1.00	15.00	A	C
ATOM	503	CB	ALA	A	80	-23.641	13.670	5.317	1.00	15.00	A	C
ATOM	504	C	ALA	A	80	-24.372	15.601	6.718	1.00	15.00	A	C
ATOM	505	O	ALA	A	80	-24.624	15.379	7.901	1.00	15.00	A	O
ATOM	506	N	GLN	A	81	-23.756	16.703	6.301	1.00	15.00	A	N
ATOM	507	CA	GLN	A	81	-23.289	17.732	7.230	1.00	15.00	A	C
ATOM	508	CB	GLN	A	81	-22.379	18.735	6.516	1.00	15.00	A	C
ATOM	509	CG	GLN	A	81	-21.096	18.122	5.974	1.00	15.00	A	C
ATOM	510	CD	GLN	A	81	-20.372	19.026	4.994	1.00	15.00	A	C
ATOM	511	OE1	GLN	A	81	-20.361	20.246	5.141	1.00	15.00	A	O
ATOM	512	NE2	GLN	A	81	-19.767	18.430	3.979	1.00	15.00	A	N
ATOM	513	C	GLN	A	81	-24.445	18.452	7.924	1.00	15.00	A	C
ATOM	514	O	GLN	A	81	-24.231	19.266	8.821	1.00	15.00	A	O
ATOM	515	N	MET	A	82	-25.670	18.145	7.511	1.00	15.00	A	N
ATOM	516	CA	MET	A	82	-26.852	18.758	8.106	1.00	15.00	A	C
ATOM	517	CB	MET	A	82	-28.032	18.729	7.129	1.00	15.00	A	C
ATOM	518	CG	MET	A	82	-27.816	19.560	5.875	1.00	15.00	A	C
ATOM	519	SD	MET	A	82	-29.235	19.528	4.762	1.00	15.00	A	S
ATOM	520	CE	MET	A	82	-28.613	20.511	3.399	1.00	15.00	A	C
ATOM	521	C	MET	A	82	-27.226	18.059	9.410	1.00	15.00	A	C
ATOM	522	O	MET	A	82	-28.087	18.526	10.154	1.00	15.00	A	O
ATOM	523	N	TYR	A	83	-26.574	16.933	9.676	1.00	15.00	A	N
ATOM	524	CA	TYR	A	83	-26.823	16.168	10.890	1.00	15.00	A	C
ATOM	525	CB	TYR	A	83	-27.124	14.704	10.548	1.00	15.00	A	C
ATOM	526	CG	TYR	A	83	-28.351	14.509	9.680	1.00	15.00	A	C
ATOM	527	CD1	TYR	A	83	-28.234	14.333	8.306	1.00	15.00	A	C
ATOM	528	CD2	TYR	A	83	-29.625	14.501	10.233	1.00	15.00	A	C
ATOM	529	CE1	TYR	A	83	-29.351	14.156	7.510	1.00	15.00	A	C
ATOM	530	CE2	TYR	A	83	-30.747	14.323	9.445	1.00	15.00	A	C
ATOM	531	CZ	TYR	A	83	-30.605	14.152	8.084	1.00	15.00	A	C
ATOM	532	OH	TYR	A	83	-31.721	13.972	7.291	1.00	15.00	A	O
ATOM	533	C	TYR	A	83	-25.619	16.262	11.829	1.00	15.00	A	C
ATOM	534	O	TYR	A	83	-24.608	15.594	11.619	1.00	15.00	A	O
ATOM	535	N	PRO	A	84	-25.709	17.105	12.872	1.00	15.00	A	N
ATOM	536	CA	PRO	A	84	-24.619	17.299	13.838	1.00	15.00	A	C
ATOM	537	CB	PRO	A	84	-25.185	18.317	14.836	1.00	15.00	A	C
ATOM	538	CG	PRO	A	84	-26.662	18.269	14.642	1.00	15.00	A	C
ATOM	539	CD	PRO	A	84	-26.881	17.932	13.196	1.00	15.00	A	C
ATOM	540	C	PRO	A	84	-24.219	16.010	14.557	1.00	15.00	A	C
ATOM	541	O	PRO	A	84	-25.054	15.326	15.149	1.00	15.00	A	O
ATOM	542	N	LEU	A	85	-22.924	15.702	14.524	1.00	15.00	A	N
ATOM	543	CA	LEU	A	85	-22.391	14.496	15.158	1.00	15.00	A	C
ATOM	544	CB	LEU	A	85	-20.958	14.230	14.696	1.00	15.00	A	C
ATOM	545	CG	LEU	A	85	-20.785	13.721	13.267	1.00	15.00	A	C
ATOM	546	CD1	LEU	A	85	-19.316	13.471	12.974	1.00	15.00	A	C
ATOM	547	CD2	LEU	A	85	-21.600	12.457	13.043	1.00	15.00	A	C
ATOM	548	C	LEU	A	85	-22.428	14.565	16.681	1.00	15.00	A	C
ATOM	549	O	LEU	A	85	-22.082	13.596	17.360	1.00	15.00	A	O
ATOM	550	N	GLN	A	86	-22.830	15.706	17.220	1.00	15.00	A	N
ATOM	551	CA	GLN	A	86	-22.907	15.870	18.663	1.00	15.00	A	C
ATOM	552	CB	GLN	A	86	-22.937	17.351	19.048	1.00	15.00	A	C
ATOM	553	CG	GLN	A	86	-22.867	17.592	20.548	1.00	15.00	A	C
ATOM	554	CD	GLN	A	86	-23.753	18.733	21.003	1.00	15.00	A	C
ATOM	555	OE1	GLN	A	86	-23.918	19.728	20.301	1.00	15.00	A	O
ATOM	556	NE2	GLN	A	86	-24.342	18.587	22.180	1.00	15.00	A	N
ATOM	557	C	GLN	A	86	-24.131	15.151	19.220	1.00	15.00	A	C

ATOM	558	O	GLN	A	86	-24.109	14.651	20.343	1.00	15.00	A	O
ATOM	559	N	GLU	A	87	-25.184	15.077	18.413	1.00	15.00	A	N
ATOM	560	CA	GLU	A	87	-26.425	14.427	18.822	1.00	15.00	A	C
ATOM	561	CB	GLU	A	87	-27.626	15.169	18.239	1.00	15.00	A	C
ATOM	562	CG	GLU	A	87	-27.790	16.589	18.751	1.00	15.00	A	C
ATOM	563	CD	GLU	A	87	-28.962	17.298	18.107	1.00	15.00	A	C
ATOM	564	OE1	GLU	A	87	-28.729	18.133	17.210	1.00	15.00	A	O
ATOM	565	OE2	GLU	A	87	-30.117	17.005	18.486	1.00	15.00	A	O
ATOM	566	C	GLU	A	87	-26.460	12.964	18.384	1.00	15.00	A	C
ATOM	567	O	GLU	A	87	-27.517	12.329	18.376	1.00	15.00	A	O
ATOM	568	N	ILE	A	88	-25.302	12.433	18.017	1.00	15.00	A	N
ATOM	569	CA	ILE	A	88	-25.209	11.047	17.578	1.00	15.00	A	C
ATOM	570	CB	ILE	A	88	-24.446	10.906	16.242	1.00	15.00	A	C
ATOM	571	CG1	ILE	A	88	-24.903	11.958	15.224	1.00	15.00	A	C
ATOM	572	CG2	ILE	A	88	-24.617	9.502	15.677	1.00	15.00	A	C
ATOM	573	CD1	ILE	A	88	-26.325	11.782	14.738	1.00	15.00	A	C
ATOM	574	C	ILE	A	88	-24.520	10.195	18.639	1.00	15.00	A	C
ATOM	575	O	ILE	A	88	-23.321	10.354	18.899	1.00	15.00	A	O
ATOM	576	N	GLN	A	89	-25.282	9.301	19.248	1.00	15.00	A	N
ATOM	577	CA	GLN	A	89	-24.762	8.415	20.278	1.00	15.00	A	C
ATOM	578	CB	GLN	A	89	-25.850	8.097	21.316	1.00	15.00	A	C
ATOM	579	CG	GLN	A	89	-26.200	9.250	22.251	1.00	15.00	A	C
ATOM	580	CD	GLN	A	89	-26.909	10.403	21.559	1.00	15.00	A	C
ATOM	581	OE1	GLN	A	89	-26.740	11.560	21.936	1.00	15.00	A	O
ATOM	582	NE2	GLN	A	89	-27.716	10.098	20.549	1.00	15.00	A	N
ATOM	583	C	GLN	A	89	-24.265	7.128	19.635	1.00	15.00	A	C
ATOM	584	O	GLN	A	89	-23.193	6.624	19.970	1.00	15.00	A	O
ATOM	585	N	ASN	A	90	-25.064	6.610	18.708	1.00	15.00	A	N
ATOM	586	CA	ASN	A	90	-24.737	5.385	17.981	1.00	15.00	A	C
ATOM	587	CB	ASN	A	90	-25.856	5.061	16.988	1.00	15.00	A	C
ATOM	588	CG	ASN	A	90	-25.799	3.638	16.468	1.00	15.00	A	C
ATOM	589	OD1	ASN	A	90	-24.853	3.248	15.786	1.00	15.00	A	O
ATOM	590	ND2	ASN	A	90	-26.826	2.859	16.773	1.00	15.00	A	N
ATOM	591	C	ASN	A	90	-23.409	5.531	17.239	1.00	15.00	A	C
ATOM	592	O	ASN	A	90	-23.286	6.338	16.318	1.00	15.00	A	O
ATOM	593	N	LEU	A	91	-22.424	4.735	17.640	1.00	15.00	A	N
ATOM	594	CA	LEU	A	91	-21.092	4.781	17.042	1.00	15.00	A	C
ATOM	595	CB	LEU	A	91	-20.107	3.922	17.838	1.00	15.00	A	C
ATOM	596	CG	LEU	A	91	-19.843	4.358	19.282	1.00	15.00	A	C
ATOM	597	CD1	LEU	A	91	-18.978	3.334	19.999	1.00	15.00	A	C
ATOM	598	CD2	LEU	A	91	-19.189	5.733	19.320	1.00	15.00	A	C
ATOM	599	C	LEU	A	91	-21.078	4.380	15.567	1.00	15.00	A	C
ATOM	600	O	LEU	A	91	-20.515	5.092	14.737	1.00	15.00	A	O
ATOM	601	N	THR	A	92	-21.702	3.254	15.239	1.00	15.00	A	N
ATOM	602	CA	THR	A	92	-21.731	2.767	13.863	1.00	15.00	A	C
ATOM	603	CB	THR	A	92	-22.421	1.398	13.778	1.00	15.00	A	C
ATOM	604	OG1	THR	A	92	-22.939	1.058	15.074	1.00	15.00	A	O
ATOM	605	CG2	THR	A	92	-21.423	0.334	13.345	1.00	15.00	A	C
ATOM	606	C	THR	A	92	-22.404	3.764	12.916	1.00	15.00	A	C
ATOM	607	O	THR	A	92	-22.073	3.832	11.731	1.00	15.00	A	O
ATOM	608	N	VAL	A	93	-23.348	4.533	13.440	1.00	15.00	A	N
ATOM	609	CA	VAL	A	93	-24.038	5.539	12.643	1.00	15.00	A	C
ATOM	610	CB	VAL	A	93	-25.414	5.895	13.253	1.00	15.00	A	C
ATOM	611	CG1	VAL	A	93	-26.024	7.111	12.569	1.00	15.00	A	C
ATOM	612	CG2	VAL	A	93	-26.357	4.705	13.151	1.00	15.00	A	C
ATOM	613	C	VAL	A	93	-23.167	6.789	12.547	1.00	15.00	A	C
ATOM	614	O	VAL	A	93	-23.038	7.400	11.484	1.00	15.00	A	O
ATOM	615	N	LYS	A	94	-22.550	7.135	13.670	1.00	15.00	A	N
ATOM	616	CA	LYS	A	94	-21.673	8.292	13.761	1.00	15.00	A	C
ATOM	617	CB	LYS	A	94	-21.164	8.415	15.198	1.00	15.00	A	C
ATOM	618	CG	LYS	A	94	-20.452	9.714	15.518	1.00	15.00	A	C
ATOM	619	CD	LYS	A	94	-20.096	9.771	16.992	1.00	15.00	A	C
ATOM	620	CE	LYS	A	94	-19.728	11.178	17.421	1.00	15.00	A	C
ATOM	621	NZ	LYS	A	94	-19.756	11.321	18.900	1.00	15.00	A	N
ATOM	622	C	LYS	A	94	-20.491	8.157	12.808	1.00	15.00	A	C
ATOM	623	O	LYS	A	94	-20.135	9.105	12.109	1.00	15.00	A	O
ATOM	624	N	LEU	A	95	-19.889	6.973	12.790	1.00	15.00	A	N
ATOM	625	CA	LEU	A	95	-18.744	6.701	11.929	1.00	15.00	A	C
ATOM	626	CB	LEU	A	95	-18.214	5.287	12.171	1.00	15.00	A	C
ATOM	627	CG	LEU	A	95	-17.571	5.038	13.537	1.00	15.00	A	C
ATOM	628	CD1	LEU	A	95	-17.283	3.558	13.730	1.00	15.00	A	C

ATOM	629	CD2	LEU	A	95	-16.299	5.858	13.689	1.00	15.00	A	C
ATOM	630	C	LEU	A	95	-19.097	6.888	10.460	1.00	15.00	A	C
ATOM	631	O	LEU	A	95	-18.338	7.495	9.703	1.00	15.00	A	O
ATOM	632	N	GLN	A	96	-20.257	6.373	10.067	1.00	15.00	A	N
ATOM	633	CA	GLN	A	96	-20.717	6.487	8.690	1.00	15.00	A	C
ATOM	634	CB	GLN	A	96	-22.003	5.687	8.479	1.00	15.00	A	C
ATOM	635	CG	GLN	A	96	-21.790	4.187	8.373	1.00	15.00	A	C
ATOM	636	CD	GLN	A	96	-23.093	3.422	8.262	1.00	15.00	A	C
ATOM	637	OE1	GLN	A	96	-23.599	3.183	7.165	1.00	15.00	A	O
ATOM	638	NE2	GLN	A	96	-23.643	3.032	9.401	1.00	15.00	A	N
ATOM	639	C	GLN	A	96	-20.933	7.948	8.316	1.00	15.00	A	C
ATOM	640	O	GLN	A	96	-20.475	8.406	7.270	1.00	15.00	A	O
ATOM	641	N	LEU	A	97	-21.620	8.679	9.187	1.00	15.00	A	N
ATOM	642	CA	LEU	A	97	-21.884	10.092	8.954	1.00	15.00	A	C
ATOM	643	CB	LEU	A	97	-22.732	10.674	10.085	1.00	15.00	A	C
ATOM	644	CG	LEU	A	97	-24.246	10.665	9.882	1.00	15.00	A	C
ATOM	645	CD1	LEU	A	97	-24.940	11.218	11.115	1.00	15.00	A	C
ATOM	646	CD2	LEU	A	97	-24.626	11.474	8.649	1.00	15.00	A	C
ATOM	647	C	LEU	A	97	-20.581	10.872	8.841	1.00	15.00	A	C
ATOM	648	O	LEU	A	97	-20.402	11.661	7.919	1.00	15.00	A	O
ATOM	649	N	GLN	A	98	-19.669	10.619	9.773	1.00	15.00	A	N
ATOM	650	CA	GLN	A	98	-18.377	11.298	9.806	1.00	15.00	A	C
ATOM	651	CB	GLN	A	98	-17.554	10.802	10.996	1.00	15.00	A	C
ATOM	652	CG	GLN	A	98	-16.520	11.796	11.497	1.00	15.00	A	C
ATOM	653	CD	GLN	A	98	-15.852	11.345	12.782	1.00	15.00	A	C
ATOM	654	OE1	GLN	A	98	-15.531	12.157	13.646	1.00	15.00	A	O
ATOM	655	NE2	GLN	A	98	-15.633	10.046	12.915	1.00	15.00	A	N
ATOM	656	C	GLN	A	98	-17.595	11.096	8.511	1.00	15.00	A	C
ATOM	657	O	GLN	A	98	-16.989	12.032	7.989	1.00	15.00	A	O
ATOM	658	N	ALA	A	99	-17.631	9.877	7.985	1.00	15.00	A	N
ATOM	659	CA	ALA	A	99	-16.915	9.549	6.756	1.00	15.00	A	C
ATOM	660	CB	ALA	A	99	-16.869	8.042	6.553	1.00	15.00	A	C
ATOM	661	C	ALA	A	99	-17.528	10.237	5.539	1.00	15.00	A	C
ATOM	662	O	ALA	A	99	-16.883	10.373	4.503	1.00	15.00	A	O
ATOM	663	N	LEU	A	100	-18.772	10.672	5.665	1.00	15.00	A	N
ATOM	664	CA	LEU	A	100	-19.452	11.339	4.565	1.00	15.00	A	C
ATOM	665	CB	LEU	A	100	-20.875	10.793	4.408	1.00	15.00	A	C
ATOM	666	CG	LEU	A	100	-21.623	11.200	3.135	1.00	15.00	A	C
ATOM	667	CD1	LEU	A	100	-20.835	10.804	1.895	1.00	15.00	A	C
ATOM	668	CD2	LEU	A	100	-23.010	10.578	3.111	1.00	15.00	A	C
ATOM	669	C	LEU	A	100	-19.478	12.854	4.760	1.00	15.00	A	C
ATOM	670	O	LEU	A	100	-19.712	13.608	3.816	1.00	15.00	A	O
ATOM	671	N	GLN	A	101	-19.221	13.300	5.981	1.00	15.00	A	N
ATOM	672	CA	GLN	A	101	-19.237	14.726	6.287	1.00	15.00	A	C
ATOM	673	CB	GLN	A	101	-19.609	14.972	7.748	1.00	15.00	A	C
ATOM	674	CG	GLN	A	101	-21.056	14.651	8.073	1.00	15.00	A	C
ATOM	675	CD	GLN	A	101	-21.421	14.969	9.506	1.00	15.00	A	C
ATOM	676	OE1	GLN	A	101	-20.572	14.973	10.393	1.00	15.00	A	O
ATOM	677	NE2	GLN	A	101	-22.693	15.231	9.741	1.00	15.00	A	N
ATOM	678	C	GLN	A	101	-17.917	15.408	5.955	1.00	15.00	A	C
ATOM	679	O	GLN	A	101	-17.861	16.632	5.847	1.00	15.00	A	O
ATOM	680	N	GLN	A	102	-16.860	14.622	5.797	1.00	15.00	A	N
ATOM	681	CA	GLN	A	102	-15.549	15.175	5.480	1.00	15.00	A	C
ATOM	682	CB	GLN	A	102	-14.432	14.139	5.660	1.00	15.00	A	C
ATOM	683	CG	GLN	A	102	-14.696	12.789	5.010	1.00	15.00	A	C
ATOM	684	CD	GLN	A	102	-13.545	11.819	5.204	1.00	15.00	A	C
ATOM	685	OE1	GLN	A	102	-13.744	10.610	5.306	1.00	15.00	A	O
ATOM	686	NE2	GLN	A	102	-12.328	12.341	5.255	1.00	15.00	A	N
ATOM	687	C	GLN	A	102	-15.516	15.796	4.085	1.00	15.00	A	C
ATOM	688	O	GLN	A	102	-15.250	15.122	3.091	1.00	15.00	A	O
ATOM	689	N	ASN	A	103	-15.802	17.091	4.021	1.00	15.00	A	N
ATOM	690	CA	ASN	A	103	-15.801	17.809	2.754	1.00	15.00	A	C
ATOM	691	CB	ASN	A	103	-16.422	19.210	2.887	1.00	15.00	A	C
ATOM	692	CG	ASN	A	103	-15.758	20.092	3.931	1.00	15.00	A	C
ATOM	693	OD1	ASN	A	103	-14.542	20.255	3.945	1.00	15.00	A	O
ATOM	694	ND2	ASN	A	103	-16.561	20.669	4.816	1.00	15.00	A	N
ATOM	695	C	ASN	A	103	-14.401	17.862	2.143	1.00	15.00	A	C
ATOM	696	O	ASN	A	103	-14.252	17.995	0.925	1.00	15.00	A	O
ATOM	697	N	GLY	A	104	-13.383	17.748	2.994	1.00	15.00	A	N
ATOM	698	CA	GLY	A	104	-12.011	17.764	2.533	1.00	15.00	A	C
ATOM	699	C	GLY	A	104	-11.650	19.061	1.856	1.00	15.00	A	C

ATOM	700	O	GLY	A	104	-11.517	20.095	2.513	1.00	15.00	A	O
ATOM	701	N	SER	A	105	-11.518	19.012	0.538	1.00	15.00	A	N
ATOM	702	CA	SER	A	105	-11.171	20.195	-0.227	1.00	15.00	A	C
ATOM	703	CB	SER	A	105	-10.560	19.826	-1.588	1.00	15.00	A	C
ATOM	704	OG	SER	A	105	-9.395	20.594	-1.857	1.00	15.00	A	O
ATOM	705	C	SER	A	105	-12.383	21.117	-0.396	1.00	15.00	A	C
ATOM	706	O	SER	A	105	-12.229	22.280	-0.755	1.00	15.00	A	O
ATOM	707	N	SER	A	106	-13.593	20.608	-0.114	1.00	15.00	A	N
ATOM	708	CA	SER	A	106	-14.803	21.417	-0.249	1.00	15.00	A	C
ATOM	709	CB	SER	A	106	-16.065	20.552	-0.361	1.00	15.00	A	C
ATOM	710	OG	SER	A	106	-15.946	19.602	-1.412	1.00	15.00	A	O
ATOM	711	C	SER	A	106	-14.940	22.441	0.877	1.00	15.00	A	C
ATOM	712	O	SER	A	106	-15.979	23.070	1.023	1.00	15.00	A	O
ATOM	713	N	VAL	A	107	-13.897	22.580	1.682	1.00	15.00	A	N
ATOM	714	CA	VAL	A	107	-13.892	23.555	2.762	1.00	15.00	A	C
ATOM	715	CB	VAL	A	107	-13.194	23.014	4.032	1.00	15.00	A	C
ATOM	716	CG1	VAL	A	107	-11.680	23.052	3.894	1.00	15.00	A	C
ATOM	717	CG2	VAL	A	107	-13.656	23.771	5.270	1.00	15.00	A	C
ATOM	718	C	VAL	A	107	-13.221	24.857	2.299	1.00	15.00	A	C
ATOM	719	O	VAL	A	107	-13.234	25.867	3.003	1.00	15.00	A	O
ATOM	720	N	LEU	A	108	-12.646	24.833	1.103	1.00	15.00	A	N
ATOM	721	CA	LEU	A	108	-11.981	26.009	0.561	1.00	15.00	A	C
ATOM	722	CB	LEU	A	108	-10.625	25.636	-0.045	1.00	15.00	A	C
ATOM	723	CG	LEU	A	108	-9.579	25.075	0.919	1.00	15.00	A	C
ATOM	724	CD1	LEU	A	108	-8.339	24.632	0.158	1.00	15.00	A	C
ATOM	725	CD2	LEU	A	108	-9.219	26.104	1.981	1.00	15.00	A	C
ATOM	726	C	LEU	A	108	-12.842	26.700	-0.489	1.00	15.00	A	C
ATOM	727	O	LEU	A	108	-13.855	26.160	-0.939	1.00	15.00	A	O
ATOM	728	N	SER	A	109	-12.428	27.898	-0.880	1.00	15.00	A	N
ATOM	729	CA	SER	A	109	-13.141	28.658	-1.886	1.00	15.00	A	C
ATOM	730	CB	SER	A	109	-12.592	30.087	-1.935	1.00	15.00	A	C
ATOM	731	OG	SER	A	109	-11.221	30.121	-1.561	1.00	15.00	A	O
ATOM	732	C	SER	A	109	-12.965	27.975	-3.234	1.00	15.00	A	C
ATOM	733	O	SER	A	109	-11.953	27.302	-3.455	1.00	15.00	A	O
ATOM	734	N	GLU	A	110	-13.939	28.134	-4.126	1.00	15.00	A	N
ATOM	735	CA	GLU	A	110	-13.872	27.517	-5.447	1.00	15.00	A	C
ATOM	736	CB	GLU	A	110	-15.007	28.015	-6.329	1.00	15.00	A	C
ATOM	737	CG	GLU	A	110	-16.390	27.837	-5.735	1.00	15.00	A	C
ATOM	738	CD	GLU	A	110	-17.427	28.609	-6.515	1.00	15.00	A	C
ATOM	739	OE1	GLU	A	110	-17.274	28.709	-7.752	1.00	15.00	A	O
ATOM	740	OE2	GLU	A	110	-18.373	29.128	-5.892	1.00	15.00	A	O
ATOM	741	C	GLU	A	110	-12.545	27.839	-6.115	1.00	15.00	A	C
ATOM	742	O	GLU	A	110	-11.946	26.991	-6.774	1.00	15.00	A	O
ATOM	743	N	ASP	A	111	-12.095	29.076	-5.926	1.00	15.00	A	N
ATOM	744	CA	ASP	A	111	-10.832	29.537	-6.489	1.00	15.00	A	C
ATOM	745	CB	ASP	A	111	-10.555	30.986	-6.081	1.00	15.00	A	C
ATOM	746	CG	ASP	A	111	-9.093	31.365	-6.243	1.00	15.00	A	C
ATOM	747	OD1	ASP	A	111	-8.603	31.382	-7.396	1.00	15.00	A	O
ATOM	748	OD2	ASP	A	111	-8.431	31.629	-5.220	1.00	15.00	A	O
ATOM	749	C	ASP	A	111	-9.673	28.645	-6.058	1.00	15.00	A	C
ATOM	750	O	ASP	A	111	-9.034	28.001	-6.892	1.00	15.00	A	O
ATOM	751	N	LYS	A	112	-9.430	28.582	-4.752	1.00	15.00	A	N
ATOM	752	CA	LYS	A	112	-8.339	27.776	-4.221	1.00	15.00	A	C
ATOM	753	CB	LYS	A	112	-8.123	28.034	-2.730	1.00	15.00	A	C
ATOM	754	CG	LYS	A	112	-7.670	29.452	-2.422	1.00	15.00	A	C
ATOM	755	CD	LYS	A	112	-7.133	29.578	-1.007	1.00	15.00	A	C
ATOM	756	CE	LYS	A	112	-6.645	30.993	-0.734	1.00	15.00	A	C
ATOM	757	NZ	LYS	A	112	-5.921	31.088	0.560	1.00	15.00	A	N
ATOM	758	C	LYS	A	112	-8.531	26.292	-4.513	1.00	15.00	A	C
ATOM	759	O	LYS	A	112	-7.565	25.575	-4.755	1.00	15.00	A	O
ATOM	760	N	SER	A	113	-9.781	25.843	-4.506	1.00	15.00	A	N
ATOM	761	CA	SER	A	113	-10.092	24.447	-4.790	1.00	15.00	A	C
ATOM	762	CB	SER	A	113	-11.592	24.184	-4.626	1.00	15.00	A	C
ATOM	763	OG	SER	A	113	-12.035	24.543	-3.330	1.00	15.00	A	O
ATOM	764	C	SER	A	113	-9.656	24.094	-6.203	1.00	15.00	A	C
ATOM	765	O	SER	A	113	-9.041	23.055	-6.436	1.00	15.00	A	O
ATOM	766	N	LYS	A	114	-9.967	24.974	-7.148	1.00	15.00	A	N
ATOM	767	CA	LYS	A	114	-9.595	24.753	-8.535	1.00	15.00	A	C
ATOM	768	CB	LYS	A	114	-10.259	25.786	-9.446	1.00	15.00	A	C
ATOM	769	CG	LYS	A	114	-11.765	25.640	-9.569	1.00	15.00	A	C
ATOM	770	CD	LYS	A	114	-12.367	26.742	-10.433	1.00	15.00	A	C

ATOM	771	CE	LYS	A	114	-12.118	28.122	-9.839	1.00	15.00	A	C
ATOM	772	NZ	LYS	A	114	-12.584	29.212	-10.739	1.00	15.00	A	N
ATOM	773	C	LYS	A	114	-8.089	24.853	-8.691	1.00	15.00	A	C
ATOM	774	O	LYS	A	114	-7.468	24.048	-9.386	1.00	15.00	A	O
ATOM	775	N	ARG	A	115	-7.511	25.842	-8.024	1.00	15.00	A	N
ATOM	776	CA	ARG	A	115	-6.080	26.079	-8.078	1.00	15.00	A	C
ATOM	777	CB	ARG	A	115	-5.714	27.316	-7.269	1.00	15.00	A	C
ATOM	778	CG	ARG	A	115	-4.400	27.971	-7.663	1.00	15.00	A	C
ATOM	779	CD	ARG	A	115	-4.175	29.225	-6.839	1.00	15.00	A	C
ATOM	780	NE	ARG	A	115	-5.268	30.185	-7.008	1.00	15.00	A	N
ATOM	781	CZ	ARG	A	115	-5.102	31.506	-6.986	1.00	15.00	A	C
ATOM	782	NH1	ARG	A	115	-3.897	32.019	-6.783	1.00	15.00	A	N
ATOM	783	NH2	ARG	A	115	-6.138	32.310	-7.219	1.00	15.00	A	N
ATOM	784	C	ARG	A	115	-5.313	24.867	-7.576	1.00	15.00	A	C
ATOM	785	O	ARG	A	115	-4.387	24.400	-8.241	1.00	15.00	A	O
ATOM	786	N	LEU	A	116	-5.728	24.340	-6.425	1.00	15.00	A	N
ATOM	787	CA	LEU	A	116	-5.089	23.165	-5.837	1.00	15.00	A	C
ATOM	788	CB	LEU	A	116	-5.724	22.818	-4.478	1.00	15.00	A	C
ATOM	789	CG	LEU	A	116	-5.146	21.601	-3.739	1.00	15.00	A	C
ATOM	790	CD1	LEU	A	116	-3.669	21.801	-3.436	1.00	15.00	A	C
ATOM	791	CD2	LEU	A	116	-5.924	21.317	-2.463	1.00	15.00	A	C
ATOM	792	C	LEU	A	116	-5.188	21.974	-6.788	1.00	15.00	A	C
ATOM	793	O	LEU	A	116	-4.200	21.272	-7.021	1.00	15.00	A	O
ATOM	794	N	ASN	A	117	-6.381	21.772	-7.354	1.00	15.00	A	N
ATOM	795	CA	ASN	A	117	-6.614	20.677	-8.294	1.00	15.00	A	C
ATOM	796	CB	ASN	A	117	-8.076	20.647	-8.743	1.00	15.00	A	C
ATOM	797	CG	ASN	A	117	-8.942	19.806	-7.829	1.00	15.00	A	C
ATOM	798	OD1	ASN	A	117	-8.506	18.778	-7.319	1.00	15.00	A	O
ATOM	799	ND2	ASN	A	117	-10.172	20.242	-7.603	1.00	15.00	A	N
ATOM	800	C	ASN	A	117	-5.697	20.801	-9.504	1.00	15.00	A	C
ATOM	801	O	ASN	A	117	-5.192	19.804	-10.027	1.00	15.00	A	O
ATOM	802	N	THR	A	118	-5.478	22.035	-9.936	1.00	15.00	A	N
ATOM	803	CA	THR	A	118	-4.612	22.308	-11.070	1.00	15.00	A	C
ATOM	804	CB	THR	A	118	-4.670	23.798	-11.463	1.00	15.00	A	C
ATOM	805	OG1	THR	A	118	-6.039	24.188	-11.634	1.00	15.00	A	O
ATOM	806	CG2	THR	A	118	-3.916	24.047	-12.760	1.00	15.00	A	C
ATOM	807	C	THR	A	118	-3.173	21.911	-10.738	1.00	15.00	A	C
ATOM	808	O	THR	A	118	-2.504	21.246	-11.530	1.00	15.00	A	O
ATOM	809	N	ILE	A	119	-2.714	22.303	-9.550	1.00	15.00	A	N
ATOM	810	CA	ILE	A	119	-1.361	21.984	-9.104	1.00	15.00	A	C
ATOM	811	CB	ILE	A	119	-1.066	22.577	-7.710	1.00	15.00	A	C
ATOM	812	CG1	ILE	A	119	-1.211	24.099	-7.735	1.00	15.00	A	C
ATOM	813	CG2	ILE	A	119	0.329	22.183	-7.242	1.00	15.00	A	C
ATOM	814	CD1	ILE	A	119	-1.279	24.726	-6.361	1.00	15.00	A	C
ATOM	815	C	ILE	A	119	-1.165	20.472	-9.054	1.00	15.00	A	C
ATOM	816	O	ILE	A	119	-0.175	19.948	-9.567	1.00	15.00	A	O
ATOM	817	N	LEU	A	120	-2.127	19.779	-8.447	1.00	15.00	A	N
ATOM	818	CA	LEU	A	120	-2.075	18.326	-8.329	1.00	15.00	A	C
ATOM	819	CB	LEU	A	120	-3.313	17.806	-7.594	1.00	15.00	A	C
ATOM	820	CG	LEU	A	120	-3.472	18.245	-6.136	1.00	15.00	A	C
ATOM	821	CD1	LEU	A	120	-4.818	17.797	-5.588	1.00	15.00	A	C
ATOM	822	CD2	LEU	A	120	-2.337	17.698	-5.285	1.00	15.00	A	C
ATOM	823	C	LEU	A	120	-1.975	17.677	-9.703	1.00	15.00	A	C
ATOM	824	O	LEU	A	120	-1.134	16.806	-9.929	1.00	15.00	A	O
ATOM	825	N	ASN	A	121	-2.829	18.122	-10.619	1.00	15.00	A	N
ATOM	826	CA	ASN	A	121	-2.839	17.594	-11.978	1.00	15.00	A	C
ATOM	827	CB	ASN	A	121	-3.957	18.236	-12.805	1.00	15.00	A	C
ATOM	828	CG	ASN	A	121	-5.277	17.495	-12.693	1.00	15.00	A	C
ATOM	829	OD1	ASN	A	121	-5.562	16.588	-13.471	1.00	15.00	A	O
ATOM	830	ND2	ASN	A	121	-6.093	17.879	-11.724	1.00	15.00	A	N
ATOM	831	C	ASN	A	121	-1.496	17.823	-12.656	1.00	15.00	A	C
ATOM	832	O	ASN	A	121	-0.912	16.898	-13.216	1.00	15.00	A	O
ATOM	833	N	THR	A	122	-1.001	19.054	-12.569	1.00	15.00	A	N
ATOM	834	CA	THR	A	122	0.274	19.419	-13.176	1.00	15.00	A	C
ATOM	835	CB	THR	A	122	0.581	20.919	-12.994	1.00	15.00	A	C
ATOM	836	OG1	THR	A	122	-0.582	21.687	-13.333	1.00	15.00	A	O
ATOM	837	CG2	THR	A	122	1.729	21.343	-13.898	1.00	15.00	A	C
ATOM	838	C	THR	A	122	1.426	18.576	-12.621	1.00	15.00	A	C
ATOM	839	O	THR	A	122	2.280	18.106	-13.377	1.00	15.00	A	O
ATOM	840	N	MET	A	123	1.442	18.374	-11.306	1.00	15.00	A	N
ATOM	841	CA	MET	A	123	2.489	17.577	-10.673	1.00	15.00	A	C

ATOM	842	CB	MET	A	123	2.366	17.607	-9.146	1.00	15.00	A	C
ATOM	843	CG	MET	A	123	2.734	18.942	-8.517	1.00	15.00	A	C
ATOM	844	SD	MET	A	123	2.955	18.845	-6.729	1.00	15.00	A	S
ATOM	845	CE	MET	A	123	1.337	18.280	-6.211	1.00	15.00	A	C
ATOM	846	C	MET	A	123	2.440	16.140	-11.178	1.00	15.00	A	C
ATOM	847	O	MET	A	123	3.474	15.527	-11.447	1.00	15.00	A	O
ATOM	848	N	SER	A	124	1.228	15.622	-11.328	1.00	15.00	A	N
ATOM	849	CA	SER	A	124	1.027	14.267	-11.810	1.00	15.00	A	C
ATOM	850	CB	SER	A	124	-0.449	13.872	-11.683	1.00	15.00	A	C
ATOM	851	OG	SER	A	124	-0.629	12.478	-11.859	1.00	15.00	A	O
ATOM	852	C	SER	A	124	1.497	14.127	-13.259	1.00	15.00	A	C
ATOM	853	O	SER	A	124	2.218	13.184	-13.596	1.00	15.00	A	O
ATOM	854	N	THR	A	125	1.106	15.080	-14.107	1.00	15.00	A	N
ATOM	855	CA	THR	A	125	1.482	15.051	-15.515	1.00	15.00	A	C
ATOM	856	CB	THR	A	125	0.785	16.160	-16.330	1.00	15.00	A	C
ATOM	857	OG1	THR	A	125	0.625	17.333	-15.525	1.00	15.00	A	O
ATOM	858	CG2	THR	A	125	-0.578	15.692	-16.815	1.00	15.00	A	C
ATOM	859	C	THR	A	125	2.994	15.136	-15.714	1.00	15.00	A	C
ATOM	860	O	THR	A	125	3.559	14.392	-16.513	1.00	15.00	A	O
ATOM	861	N	ILE	A	126	3.648	16.030	-14.979	1.00	15.00	A	N
ATOM	862	CA	ILE	A	126	5.095	16.198	-15.097	1.00	15.00	A	C
ATOM	863	CB	ILE	A	126	5.606	17.398	-14.266	1.00	15.00	A	C
ATOM	864	CG1	ILE	A	126	5.042	18.712	-14.816	1.00	15.00	A	C
ATOM	865	CG2	ILE	A	126	7.128	17.442	-14.248	1.00	15.00	A	C
ATOM	866	CD1	ILE	A	126	5.461	19.936	-14.029	1.00	15.00	A	C
ATOM	867	C	ILE	A	126	5.829	14.925	-14.680	1.00	15.00	A	C
ATOM	868	O	ILE	A	126	6.805	14.523	-15.308	1.00	15.00	A	O
ATOM	869	N	TYR	A	127	5.330	14.279	-13.636	1.00	15.00	A	N
ATOM	870	CA	TYR	A	127	5.939	13.057	-13.126	1.00	15.00	A	C
ATOM	871	CB	TYR	A	127	5.322	12.687	-11.773	1.00	15.00	A	C
ATOM	872	CG	TYR	A	127	5.912	11.446	-11.143	1.00	15.00	A	C
ATOM	873	CD1	TYR	A	127	7.088	11.511	-10.411	1.00	15.00	A	C
ATOM	874	CD2	TYR	A	127	5.294	10.209	-11.283	1.00	15.00	A	C
ATOM	875	CE1	TYR	A	127	7.634	10.380	-9.838	1.00	15.00	A	C
ATOM	876	CE2	TYR	A	127	5.833	9.073	-10.712	1.00	15.00	A	C
ATOM	877	CZ	TYR	A	127	7.004	9.165	-9.992	1.00	15.00	A	C
ATOM	878	OH	TYR	A	127	7.544	8.035	-9.418	1.00	15.00	A	O
ATOM	879	C	TYR	A	127	5.799	11.889	-14.105	1.00	15.00	A	C
ATOM	880	O	TYR	A	127	6.647	10.997	-14.146	1.00	15.00	A	O
ATOM	881	N	SER	A	128	4.740	11.900	-14.899	1.00	15.00	A	N
ATOM	882	CA	SER	A	128	4.496	10.822	-15.847	1.00	15.00	A	C
ATOM	883	CB	SER	A	128	2.993	10.576	-15.994	1.00	15.00	A	C
ATOM	884	OG	SER	A	128	2.270	11.792	-15.904	1.00	15.00	A	O
ATOM	885	C	SER	A	128	5.136	11.091	-17.205	1.00	15.00	A	C
ATOM	886	O	SER	A	128	5.705	10.192	-17.822	1.00	15.00	A	O
ATOM	887	N	THR	A	129	5.052	12.330	-17.666	1.00	15.00	A	N
ATOM	888	CA	THR	A	129	5.619	12.687	-18.956	1.00	15.00	A	C
ATOM	889	CB	THR	A	129	4.799	13.775	-19.679	1.00	15.00	A	C
ATOM	890	OG1	THR	A	129	4.652	14.923	-18.837	1.00	15.00	A	O
ATOM	891	CG2	THR	A	129	3.423	13.247	-20.058	1.00	15.00	A	C
ATOM	892	C	THR	A	129	7.081	13.111	-18.844	1.00	15.00	A	C
ATOM	893	O	THR	A	129	7.710	13.453	-19.845	1.00	15.00	A	O
ATOM	894	N	GLY	A	130	7.617	13.088	-17.630	1.00	15.00	A	N
ATOM	895	CA	GLY	A	130	9.004	13.457	-17.422	1.00	15.00	A	C
ATOM	896	C	GLY	A	130	9.949	12.558	-18.194	1.00	15.00	A	C
ATOM	897	O	GLY	A	130	9.823	11.333	-18.154	1.00	15.00	A	O
ATOM	898	N	LYS	A	131	10.887	13.161	-18.907	1.00	15.00	A	N
ATOM	899	CA	LYS	A	131	11.846	12.408	-19.701	1.00	15.00	A	C
ATOM	900	CB	LYS	A	131	11.309	12.180	-21.120	1.00	15.00	A	C
ATOM	901	CG	LYS	A	131	10.967	13.457	-21.875	1.00	15.00	A	C
ATOM	902	CD	LYS	A	131	10.391	13.163	-23.253	1.00	15.00	A	C
ATOM	903	CE	LYS	A	131	8.977	12.611	-23.162	1.00	15.00	A	C
ATOM	904	NZ	LYS	A	131	8.083	13.503	-22.375	1.00	15.00	A	N
ATOM	905	C	LYS	A	131	13.174	13.146	-19.757	1.00	15.00	A	C
ATOM	906	O	LYS	A	131	13.207	14.360	-19.934	1.00	15.00	A	O
ATOM	907	N	VAL	A	132	14.262	12.414	-19.603	1.00	15.00	A	N
ATOM	908	CA	VAL	A	132	15.582	13.017	-19.635	1.00	15.00	A	C
ATOM	909	CB	VAL	A	132	16.352	12.819	-18.313	1.00	15.00	A	C
ATOM	910	CG1	VAL	A	132	15.661	13.574	-17.187	1.00	15.00	A	C
ATOM	911	CG2	VAL	A	132	16.475	11.344	-17.968	1.00	15.00	A	C
ATOM	912	C	VAL	A	132	16.394	12.496	-20.812	1.00	15.00	A	C

ATOM	913	O	VAL	A	132	16.504	11.287	-21.027	1.00	15.00	A	O
ATOM	914	N	CYS	A	133	16.928	13.415	-21.592	1.00	15.00	A	N
ATOM	915	CA	CYS	A	133	17.724	13.061	-22.753	1.00	15.00	A	C
ATOM	916	C	CYS	A	133	19.210	13.100	-22.415	1.00	15.00	A	C
ATOM	917	O	CYS	A	133	19.624	13.788	-21.477	1.00	15.00	A	O
ATOM	918	CB	CYS	A	133	17.412	14.018	-23.907	1.00	15.00	A	C
ATOM	919	SG	CYS	A	133	15.743	14.758	-23.825	1.00	15.00	A	S
ATOM	920	N	ASN	A	134	20.003	12.348	-23.164	1.00	15.00	A	N
ATOM	921	CA	ASN	A	134	21.445	12.307	-22.952	1.00	15.00	A	C
ATOM	922	CB	ASN	A	134	22.048	10.995	-23.475	1.00	15.00	A	C
ATOM	923	CG	ASN	A	134	21.820	10.781	-24.958	1.00	15.00	A	C
ATOM	924	OD1	ASN	A	134	20.722	10.979	-25.460	1.00	15.00	A	O
ATOM	925	ND2	ASN	A	134	22.865	10.400	-25.672	1.00	15.00	A	N
ATOM	926	C	ASN	A	134	22.104	13.506	-23.627	1.00	15.00	A	C
ATOM	927	O	ASN	A	134	21.590	14.023	-24.617	1.00	15.00	A	O
ATOM	928	N	PRO	A	135	23.254	13.962	-23.106	1.00	15.00	A	N
ATOM	929	CA	PRO	A	135	23.963	15.125	-23.657	1.00	15.00	A	C
ATOM	930	CB	PRO	A	135	25.089	15.367	-22.647	1.00	15.00	A	C
ATOM	931	CG	PRO	A	135	25.309	14.041	-22.004	1.00	15.00	A	C
ATOM	932	CD	PRO	A	135	23.960	13.382	-21.949	1.00	15.00	A	C
ATOM	933	C	PRO	A	135	24.543	14.870	-25.048	1.00	15.00	A	C
ATOM	934	O	PRO	A	135	24.888	15.810	-25.763	1.00	15.00	A	O
ATOM	935	N	ASP	A	136	24.638	13.603	-25.426	1.00	15.00	A	N
ATOM	936	CA	ASP	A	136	25.190	13.230	-26.724	1.00	15.00	A	C
ATOM	937	CB	ASP	A	136	25.830	11.836	-26.670	1.00	15.00	A	C
ATOM	938	CG	ASP	A	136	26.135	11.373	-25.258	1.00	15.00	A	C
ATOM	939	OD1	ASP	A	136	27.274	11.574	-24.796	1.00	15.00	A	O
ATOM	940	OD2	ASP	A	136	25.223	10.815	-24.605	1.00	15.00	A	O
ATOM	941	C	ASP	A	136	24.138	13.278	-27.830	1.00	15.00	A	C
ATOM	942	O	ASP	A	136	24.451	13.600	-28.976	1.00	15.00	A	O
ATOM	943	N	ASN	A	137	22.892	12.964	-27.495	1.00	15.00	A	N
ATOM	944	CA	ASN	A	137	21.820	12.967	-28.486	1.00	15.00	A	C
ATOM	945	CB	ASN	A	137	21.535	11.550	-28.996	1.00	15.00	A	C
ATOM	946	CG	ASN	A	137	20.551	11.527	-30.152	1.00	15.00	A	C
ATOM	947	OD1	ASN	A	137	20.324	12.542	-30.814	1.00	15.00	A	O
ATOM	948	ND2	ASN	A	137	19.972	10.368	-30.417	1.00	15.00	A	N
ATOM	949	C	ASN	A	137	20.552	13.616	-27.938	1.00	15.00	A	C
ATOM	950	O	ASN	A	137	19.864	13.052	-27.092	1.00	15.00	A	O
ATOM	951	N	PRO	A	138	20.217	14.813	-28.440	1.00	15.00	A	N
ATOM	952	CA	PRO	A	138	19.040	15.570	-27.994	1.00	15.00	A	C
ATOM	953	CB	PRO	A	138	19.123	16.880	-28.791	1.00	15.00	A	C
ATOM	954	CG	PRO	A	138	20.534	16.954	-29.266	1.00	15.00	A	C
ATOM	955	CD	PRO	A	138	20.969	15.535	-29.478	1.00	15.00	A	C
ATOM	956	C	PRO	A	138	17.707	14.869	-28.271	1.00	15.00	A	C
ATOM	957	O	PRO	A	138	16.690	15.223	-27.680	1.00	15.00	A	O
ATOM	958	N	GLN	A	139	17.703	13.883	-29.163	1.00	15.00	A	N
ATOM	959	CA	GLN	A	139	16.465	13.175	-29.489	1.00	15.00	A	C
ATOM	960	CB	GLN	A	139	16.349	12.897	-30.993	1.00	15.00	A	C
ATOM	961	CG	GLN	A	139	17.392	11.937	-31.538	1.00	15.00	A	C
ATOM	962	CD	GLN	A	139	17.087	11.457	-32.944	1.00	15.00	A	C
ATOM	963	OE1	GLN	A	139	15.931	11.306	-33.327	1.00	15.00	A	O
ATOM	964	NE2	GLN	A	139	18.130	11.215	-33.724	1.00	15.00	A	N
ATOM	965	C	GLN	A	139	16.299	11.885	-28.685	1.00	15.00	A	C
ATOM	966	O	GLN	A	139	15.258	11.232	-28.757	1.00	15.00	A	O
ATOM	967	N	GLU	A	140	17.313	11.524	-27.909	1.00	15.00	A	N
ATOM	968	CA	GLU	A	140	17.257	10.307	-27.109	1.00	15.00	A	C
ATOM	969	CB	GLU	A	140	18.595	9.567	-27.149	1.00	15.00	A	C
ATOM	970	CG	GLU	A	140	18.509	8.159	-27.713	1.00	15.00	A	C
ATOM	971	CD	GLU	A	140	19.854	7.635	-28.174	1.00	15.00	A	C
ATOM	972	OE1	GLU	A	140	20.415	6.751	-27.496	1.00	15.00	A	O
ATOM	973	OE2	GLU	A	140	20.349	8.110	-29.219	1.00	15.00	A	O
ATOM	974	C	GLU	A	140	16.847	10.615	-25.673	1.00	15.00	A	C
ATOM	975	O	GLU	A	140	17.681	10.699	-24.772	1.00	15.00	A	O
ATOM	976	N	CYS	A	141	15.553	10.804	-25.469	1.00	15.00	A	N
ATOM	977	CA	CYS	A	141	15.027	11.108	-24.148	1.00	15.00	A	C
ATOM	978	C	CYS	A	141	14.354	9.886	-23.533	1.00	15.00	A	C
ATOM	979	O	CYS	A	141	13.442	9.305	-24.123	1.00	15.00	A	O
ATOM	980	CB	CYS	A	141	14.043	12.273	-24.230	1.00	15.00	A	C
ATOM	981	SG	CYS	A	141	14.659	13.701	-25.179	1.00	15.00	A	S
ATOM	982	N	LEU	A	142	14.800	9.504	-22.345	1.00	15.00	A	N
ATOM	983	CA	LEU	A	142	14.247	8.349	-21.655	1.00	15.00	A	C

ATOM	984	CB	LEU	A	142	15.369	7.451	-21.125	1.00	15.00	A	C
ATOM	985	CG	LEU	A	142	16.253	6.775	-22.175	1.00	15.00	A	C
ATOM	986	CD1	LEU	A	142	17.420	6.063	-21.510	1.00	15.00	A	C
ATOM	987	CD2	LEU	A	142	15.439	5.802	-23.016	1.00	15.00	A	C
ATOM	988	C	LEU	A	142	13.339	8.773	-20.507	1.00	15.00	A	C
ATOM	989	O	LEU	A	142	13.674	9.671	-19.733	1.00	15.00	A	O
ATOM	990	N	LEU	A	143	12.184	8.128	-20.415	1.00	15.00	A	N
ATOM	991	CA	LEU	A	143	11.225	8.410	-19.356	1.00	15.00	A	C
ATOM	992	CB	LEU	A	143	9.817	8.009	-19.809	1.00	15.00	A	C
ATOM	993	CG	LEU	A	143	9.284	8.682	-21.074	1.00	15.00	A	C
ATOM	994	CD1	LEU	A	143	8.520	7.681	-21.927	1.00	15.00	A	C
ATOM	995	CD2	LEU	A	143	8.392	9.859	-20.714	1.00	15.00	A	C
ATOM	996	C	LEU	A	143	11.595	7.602	-18.120	1.00	15.00	A	C
ATOM	997	O	LEU	A	143	12.457	6.721	-18.190	1.00	15.00	A	O
ATOM	998	N	LEU	A	144	10.959	7.899	-16.992	1.00	15.00	A	N
ATOM	999	CA	LEU	A	144	11.226	7.167	-15.760	1.00	15.00	A	C
ATOM	1000	CB	LEU	A	144	10.400	7.726	-14.599	1.00	15.00	A	C
ATOM	1001	CG	LEU	A	144	10.521	6.978	-13.267	1.00	15.00	A	C
ATOM	1002	CD1	LEU	A	144	11.883	7.214	-12.633	1.00	15.00	A	C
ATOM	1003	CD2	LEU	A	144	9.404	7.379	-12.317	1.00	15.00	A	C
ATOM	1004	C	LEU	A	144	10.906	5.692	-15.959	1.00	15.00	A	C
ATOM	1005	O	LEU	A	144	11.753	4.825	-15.754	1.00	15.00	A	O
ATOM	1006	N	GLU	A	145	9.686	5.426	-16.391	1.00	15.00	A	N
ATOM	1007	CA	GLU	A	145	9.238	4.068	-16.630	1.00	15.00	A	C
ATOM	1008	CB	GLU	A	145	7.864	3.852	-15.985	1.00	15.00	A	C
ATOM	1009	CG	GLU	A	145	7.294	2.449	-16.153	1.00	15.00	A	C
ATOM	1010	CD	GLU	A	145	8.200	1.371	-15.590	1.00	15.00	A	C
ATOM	1011	OE1	GLU	A	145	9.063	1.697	-14.749	1.00	15.00	A	O
ATOM	1012	OE2	GLU	A	145	8.053	0.203	-15.994	1.00	15.00	A	O
ATOM	1013	C	GLU	A	145	9.165	3.796	-18.129	1.00	15.00	A	C
ATOM	1014	O	GLU	A	145	8.494	4.519	-18.864	1.00	15.00	A	O
ATOM	1015	N	PRO	A	146	9.896	2.782	-18.611	1.00	15.00	A	N
ATOM	1016	CA	PRO	A	146	10.773	1.955	-17.793	1.00	15.00	A	C
ATOM	1017	CB	PRO	A	146	10.474	0.576	-18.377	1.00	15.00	A	C
ATOM	1018	CG	PRO	A	146	10.235	0.827	-19.839	1.00	15.00	A	C
ATOM	1019	CD	PRO	A	146	9.865	2.289	-19.993	1.00	15.00	A	C
ATOM	1020	C	PRO	A	146	12.240	2.293	-18.047	1.00	15.00	A	C
ATOM	1021	O	PRO	A	146	13.135	1.549	-17.657	1.00	15.00	A	O
ATOM	1022	N	GLY	A	147	12.472	3.427	-18.700	1.00	15.00	A	N
ATOM	1023	CA	GLY	A	147	13.821	3.842	-19.041	1.00	15.00	A	C
ATOM	1024	C	GLY	A	147	14.726	4.039	-17.842	1.00	15.00	A	C
ATOM	1025	O	GLY	A	147	15.644	3.255	-17.611	1.00	15.00	A	O
ATOM	1026	N	LEU	A	148	14.466	5.085	-17.077	1.00	15.00	A	N
ATOM	1027	CA	LEU	A	148	15.277	5.399	-15.909	1.00	15.00	A	C
ATOM	1028	CB	LEU	A	148	14.876	6.749	-15.322	1.00	15.00	A	C
ATOM	1029	CG	LEU	A	148	15.093	7.960	-16.231	1.00	15.00	A	C
ATOM	1030	CD1	LEU	A	148	14.726	9.244	-15.507	1.00	15.00	A	C
ATOM	1031	CD2	LEU	A	148	16.531	8.008	-16.725	1.00	15.00	A	C
ATOM	1032	C	LEU	A	148	15.210	4.307	-14.847	1.00	15.00	A	C
ATOM	1033	O	LEU	A	148	16.201	4.033	-14.169	1.00	15.00	A	O
ATOM	1034	N	ASN	A	149	14.047	3.684	-14.710	1.00	15.00	A	N
ATOM	1035	CA	ASN	A	149	13.861	2.624	-13.727	1.00	15.00	A	C
ATOM	1036	CB	ASN	A	149	12.397	2.190	-13.632	1.00	15.00	A	C
ATOM	1037	CG	ASN	A	149	11.581	3.095	-12.728	1.00	15.00	A	C
ATOM	1038	OD1	ASN	A	149	12.126	3.791	-11.869	1.00	15.00	A	O
ATOM	1039	ND2	ASN	A	149	10.272	3.088	-12.903	1.00	15.00	A	N
ATOM	1040	C	ASN	A	149	14.762	1.432	-14.013	1.00	15.00	A	C
ATOM	1041	O	ASN	A	149	15.369	0.879	-13.099	1.00	15.00	A	O
ATOM	1042	N	GLU	A	150	14.858	1.054	-15.284	1.00	15.00	A	N
ATOM	1043	CA	GLU	A	150	15.697	-0.068	-15.689	1.00	15.00	A	C
ATOM	1044	CB	GLU	A	150	15.517	-0.346	-17.187	1.00	15.00	A	C
ATOM	1045	CG	GLU	A	150	16.093	-1.670	-17.669	1.00	15.00	A	C
ATOM	1046	CD	GLU	A	150	17.583	-1.598	-17.944	1.00	15.00	A	C
ATOM	1047	OE1	GLU	A	150	18.285	-2.599	-17.691	1.00	15.00	A	O
ATOM	1048	OE2	GLU	A	150	18.049	-0.535	-18.402	1.00	15.00	A	O
ATOM	1049	C	GLU	A	150	17.158	0.228	-15.366	1.00	15.00	A	C
ATOM	1050	O	GLU	A	150	17.838	-0.572	-14.723	1.00	15.00	A	O
ATOM	1051	N	ILE	A	151	17.621	1.399	-15.785	1.00	15.00	A	N
ATOM	1052	CA	ILE	A	151	18.999	1.816	-15.546	1.00	15.00	A	C
ATOM	1053	CB	ILE	A	151	19.274	3.218	-16.135	1.00	15.00	A	C
ATOM	1054	CG1	ILE	A	151	19.032	3.216	-17.648	1.00	15.00	A	C

ATOM	1055	CG2	ILE	A	151	20.694	3.672	-15.819	1.00	15.00	A	C
ATOM	1056	CD1	ILE	A	151	19.154	4.579	-18.293	1.00	15.00	A	C
ATOM	1057	C	ILE	A	151	19.324	1.816	-14.050	1.00	15.00	A	C
ATOM	1058	O	ILE	A	151	20.391	1.370	-13.630	1.00	15.00	A	O
ATOM	1059	N	MET	A	152	18.385	2.286	-13.244	1.00	15.00	A	N
ATOM	1060	CA	MET	A	152	18.586	2.349	-11.803	1.00	15.00	A	C
ATOM	1061	CB	MET	A	152	17.721	3.444	-11.178	1.00	15.00	A	C
ATOM	1062	CG	MET	A	152	18.110	4.852	-11.600	1.00	15.00	A	C
ATOM	1063	SD	MET	A	152	19.790	5.291	-11.114	1.00	15.00	A	S
ATOM	1064	CE	MET	A	152	19.563	5.605	-9.368	1.00	15.00	A	C
ATOM	1065	C	MET	A	152	18.327	1.007	-11.123	1.00	15.00	A	C
ATOM	1066	O	MET	A	152	18.379	0.909	-9.898	1.00	15.00	A	O
ATOM	1067	N	ALA	A	153	18.065	-0.027	-11.909	1.00	15.00	A	N
ATOM	1068	CA	ALA	A	153	17.801	-1.346	-11.355	1.00	15.00	A	C
ATOM	1069	CB	ALA	A	153	16.369	-1.773	-11.642	1.00	15.00	A	C
ATOM	1070	C	ALA	A	153	18.783	-2.398	-11.864	1.00	15.00	A	C
ATOM	1071	O	ALA	A	153	19.329	-3.173	-11.078	1.00	15.00	A	O
ATOM	1072	N	ASN	A	154	19.023	-2.409	-13.169	1.00	15.00	A	N
ATOM	1073	CA	ASN	A	154	19.908	-3.399	-13.775	1.00	15.00	A	C
ATOM	1074	CB	ASN	A	154	19.288	-3.977	-15.048	1.00	15.00	A	C
ATOM	1075	CG	ASN	A	154	18.038	-4.792	-14.768	1.00	15.00	A	C
ATOM	1076	OD1	ASN	A	154	16.933	-4.257	-14.692	1.00	15.00	A	O
ATOM	1077	ND2	ASN	A	154	18.203	-6.098	-14.609	1.00	15.00	A	N
ATOM	1078	C	ASN	A	154	21.320	-2.884	-14.048	1.00	15.00	A	C
ATOM	1079	O	ASN	A	154	22.283	-3.647	-13.952	1.00	15.00	A	O
ATOM	1080	N	SER	A	155	21.455	-1.607	-14.400	1.00	15.00	A	N
ATOM	1081	CA	SER	A	155	22.775	-1.043	-14.678	1.00	15.00	A	C
ATOM	1082	CB	SER	A	155	22.686	0.369	-15.260	1.00	15.00	A	C
ATOM	1083	OG	SER	A	155	23.968	0.974	-15.321	1.00	15.00	A	O
ATOM	1084	C	SER	A	155	23.649	-1.059	-13.430	1.00	15.00	A	C
ATOM	1085	O	SER	A	155	23.176	-0.798	-12.320	1.00	15.00	A	O
ATOM	1086	N	LEU	A	156	24.926	-1.359	-13.619	1.00	15.00	A	N
ATOM	1087	CA	LEU	A	156	25.865	-1.431	-12.512	1.00	15.00	A	C
ATOM	1088	CB	LEU	A	156	26.452	-2.842	-12.407	1.00	15.00	A	C
ATOM	1089	CG	LEU	A	156	25.463	-3.968	-12.097	1.00	15.00	A	C
ATOM	1090	CD1	LEU	A	156	26.111	-5.324	-12.328	1.00	15.00	A	C
ATOM	1091	CD2	LEU	A	156	24.952	-3.856	-10.668	1.00	15.00	A	C
ATOM	1092	C	LEU	A	156	26.987	-0.411	-12.657	1.00	15.00	A	C
ATOM	1093	O	LEU	A	156	27.980	-0.469	-11.929	1.00	15.00	A	O
ATOM	1094	N	ASP	A	157	26.840	0.523	-13.589	1.00	15.00	A	N
ATOM	1095	CA	ASP	A	157	27.868	1.539	-13.787	1.00	15.00	A	C
ATOM	1096	CB	ASP	A	157	27.959	2.015	-15.236	1.00	15.00	A	C
ATOM	1097	CG	ASP	A	157	29.043	3.064	-15.406	1.00	15.00	A	C
ATOM	1098	OD1	ASP	A	157	30.236	2.702	-15.386	1.00	15.00	A	O
ATOM	1099	OD2	ASP	A	157	28.702	4.258	-15.522	1.00	15.00	A	O
ATOM	1100	C	ASP	A	157	27.651	2.715	-12.846	1.00	15.00	A	C
ATOM	1101	O	ASP	A	157	26.526	3.179	-12.661	1.00	15.00	A	O
ATOM	1102	N	TYR	A	158	28.735	3.192	-12.258	1.00	15.00	A	N
ATOM	1103	CA	TYR	A	158	28.674	4.297	-11.313	1.00	15.00	A	C
ATOM	1104	CB	TYR	A	158	30.012	4.452	-10.582	1.00	15.00	A	C
ATOM	1105	CG	TYR	A	158	29.991	5.480	-9.472	1.00	15.00	A	C
ATOM	1106	CD1	TYR	A	158	30.377	6.795	-9.706	1.00	15.00	A	C
ATOM	1107	CD2	TYR	A	158	29.579	5.138	-8.191	1.00	15.00	A	C
ATOM	1108	CE1	TYR	A	158	30.352	7.738	-8.697	1.00	15.00	A	C
ATOM	1109	CE2	TYR	A	158	29.552	6.074	-7.176	1.00	15.00	A	C
ATOM	1110	CZ	TYR	A	158	29.938	7.372	-7.434	1.00	15.00	A	C
ATOM	1111	OH	TYR	A	158	29.909	8.307	-6.426	1.00	15.00	A	O
ATOM	1112	C	TYR	A	158	28.280	5.611	-11.982	1.00	15.00	A	C
ATOM	1113	O	TYR	A	158	27.547	6.410	-11.400	1.00	15.00	A	O
ATOM	1114	N	ASN	A	159	28.744	5.821	-13.205	1.00	15.00	A	N
ATOM	1115	CA	ASN	A	159	28.461	7.058	-13.926	1.00	15.00	A	C
ATOM	1116	CB	ASN	A	159	29.502	7.303	-15.021	1.00	15.00	A	C
ATOM	1117	CG	ASN	A	159	30.829	7.789	-14.470	1.00	15.00	A	C
ATOM	1118	OD1	ASN	A	159	30.877	8.528	-13.489	1.00	15.00	A	O
ATOM	1119	ND2	ASN	A	159	31.918	7.368	-15.094	1.00	15.00	A	N
ATOM	1120	C	ASN	A	159	27.058	7.062	-14.515	1.00	15.00	A	C
ATOM	1121	O	ASN	A	159	26.316	8.033	-14.362	1.00	15.00	A	O
ATOM	1122	N	GLU	A	160	26.701	5.973	-15.185	1.00	15.00	A	N
ATOM	1123	CA	GLU	A	160	25.385	5.842	-15.801	1.00	15.00	A	C
ATOM	1124	CB	GLU	A	160	25.262	4.493	-16.511	1.00	15.00	A	C
ATOM	1125	CG	GLU	A	160	24.047	4.369	-17.414	1.00	15.00	A	C

ATOM	1126	CD	GLU	A	160	24.048	3.080	-18.210	1.00	15.00	A	C
ATOM	1127	OE1	GLU	A	160	23.723	3.123	-19.413	1.00	15.00	A	O
ATOM	1128	OE2	GLU	A	160	24.378	2.023	-17.627	1.00	15.00	A	O
ATOM	1129	C	GLU	A	160	24.272	6.012	-14.768	1.00	15.00	A	C
ATOM	1130	O	GLU	A	160	23.325	6.774	-14.978	1.00	15.00	A	O
ATOM	1131	N	ARG	A	161	24.404	5.318	-13.641	1.00	15.00	A	N
ATOM	1132	CA	ARG	A	161	23.412	5.405	-12.574	1.00	15.00	A	C
ATOM	1133	CB	ARG	A	161	23.680	4.362	-11.488	1.00	15.00	A	C
ATOM	1134	CG	ARG	A	161	23.513	2.925	-11.958	1.00	15.00	A	C
ATOM	1135	CD	ARG	A	161	23.728	1.941	-10.820	1.00	15.00	A	C
ATOM	1136	NE	ARG	A	161	22.724	2.105	-9.770	1.00	15.00	A	N
ATOM	1137	CZ	ARG	A	161	21.678	1.298	-9.604	1.00	15.00	A	C
ATOM	1138	NH1	ARG	A	161	21.492	0.257	-10.410	1.00	15.00	A	N
ATOM	1139	NH2	ARG	A	161	20.804	1.550	-8.638	1.00	15.00	A	N
ATOM	1140	C	ARG	A	161	23.383	6.809	-11.978	1.00	15.00	A	C
ATOM	1141	O	ARG	A	161	22.325	7.316	-11.608	1.00	15.00	A	O
ATOM	1142	N	LEU	A	162	24.553	7.437	-11.899	1.00	15.00	A	N
ATOM	1143	CA	LEU	A	162	24.663	8.790	-11.368	1.00	15.00	A	C
ATOM	1144	CB	LEU	A	162	26.138	9.200	-11.270	1.00	15.00	A	C
ATOM	1145	CG	LEU	A	162	26.426	10.661	-10.920	1.00	15.00	A	C
ATOM	1146	CD1	LEU	A	162	25.947	10.984	-9.514	1.00	15.00	A	C
ATOM	1147	CD2	LEU	A	162	27.911	10.962	-11.064	1.00	15.00	A	C
ATOM	1148	C	LEU	A	162	23.904	9.762	-12.265	1.00	15.00	A	C
ATOM	1149	O	LEU	A	162	23.188	10.641	-11.783	1.00	15.00	A	O
ATOM	1150	N	TRP	A	163	24.059	9.578	-13.572	1.00	15.00	A	N
ATOM	1151	CA	TRP	A	163	23.395	10.418	-14.558	1.00	15.00	A	C
ATOM	1152	CB	TRP	A	163	23.814	10.010	-15.977	1.00	15.00	A	C
ATOM	1153	CG	TRP	A	163	23.009	10.664	-17.063	1.00	15.00	A	C
ATOM	1154	CD1	TRP	A	163	23.119	11.952	-17.501	1.00	15.00	A	C
ATOM	1155	CD2	TRP	A	163	21.969	10.061	-17.848	1.00	15.00	A	C
ATOM	1156	NE1	TRP	A	163	22.213	12.189	-18.507	1.00	15.00	A	N
ATOM	1157	CE2	TRP	A	163	21.496	11.045	-18.738	1.00	15.00	A	C
ATOM	1158	CE3	TRP	A	163	21.393	8.787	-17.883	1.00	15.00	A	C
ATOM	1159	CZ2	TRP	A	163	20.475	10.794	-19.653	1.00	15.00	A	C
ATOM	1160	CZ3	TRP	A	163	20.380	8.541	-18.792	1.00	15.00	A	C
ATOM	1161	CH2	TRP	A	163	19.932	9.539	-19.664	1.00	15.00	A	C
ATOM	1162	C	TRP	A	163	21.880	10.336	-14.415	1.00	15.00	A	C
ATOM	1163	O	TRP	A	163	21.205	11.359	-14.331	1.00	15.00	A	O
ATOM	1164	N	ALA	A	164	21.357	9.114	-14.381	1.00	15.00	A	N
ATOM	1165	CA	ALA	A	164	19.919	8.895	-14.255	1.00	15.00	A	C
ATOM	1166	CB	ALA	A	164	19.598	7.413	-14.373	1.00	15.00	A	C
ATOM	1167	C	ALA	A	164	19.387	9.457	-12.940	1.00	15.00	A	C
ATOM	1168	O	ALA	A	164	18.317	10.070	-12.899	1.00	15.00	A	O
ATOM	1169	N	TRP	A	165	20.149	9.251	-11.873	1.00	15.00	A	N
ATOM	1170	CA	TRP	A	165	19.774	9.731	-10.551	1.00	15.00	A	C
ATOM	1171	CB	TRP	A	165	20.813	9.279	-9.517	1.00	15.00	A	C
ATOM	1172	CG	TRP	A	165	20.405	9.514	-8.093	1.00	15.00	A	C
ATOM	1173	CD1	TRP	A	165	19.727	8.648	-7.287	1.00	15.00	A	C
ATOM	1174	CD2	TRP	A	165	20.653	10.686	-7.301	1.00	15.00	A	C
ATOM	1175	NE1	TRP	A	165	19.529	9.206	-6.050	1.00	15.00	A	N
ATOM	1176	CE2	TRP	A	165	20.090	10.457	-6.032	1.00	15.00	A	C
ATOM	1177	CE3	TRP	A	165	21.296	11.905	-7.543	1.00	15.00	A	C
ATOM	1178	CZ2	TRP	A	165	20.146	11.401	-5.011	1.00	15.00	A	C
ATOM	1179	CZ3	TRP	A	165	21.352	12.840	-6.526	1.00	15.00	A	C
ATOM	1180	CH2	TRP	A	165	20.781	12.583	-5.275	1.00	15.00	A	C
ATOM	1181	C	TRP	A	165	19.672	11.251	-10.534	1.00	15.00	A	C
ATOM	1182	O	TRP	A	165	18.717	11.815	-9.989	1.00	15.00	A	O
ATOM	1183	N	GLU	A	166	20.658	11.903	-11.132	1.00	15.00	A	N
ATOM	1184	CA	GLU	A	166	20.698	13.354	-11.172	1.00	15.00	A	C
ATOM	1185	CB	GLU	A	166	22.109	13.860	-11.487	1.00	15.00	A	C
ATOM	1186	CG	GLU	A	166	22.271	15.372	-11.390	1.00	15.00	A	C
ATOM	1187	CD	GLU	A	166	21.783	15.931	-10.067	1.00	15.00	A	C
ATOM	1188	OE1	GLU	A	166	21.859	15.212	-9.049	1.00	15.00	A	O
ATOM	1189	OE2	GLU	A	166	21.318	17.088	-10.037	1.00	15.00	A	O
ATOM	1190	C	GLU	A	166	19.689	13.936	-12.153	1.00	15.00	A	C
ATOM	1191	O	GLU	A	166	18.953	14.854	-11.800	1.00	15.00	A	O
ATOM	1192	N	SER	A	167	19.625	13.384	-13.363	1.00	15.00	A	N
ATOM	1193	CA	SER	A	167	18.725	13.900	-14.393	1.00	15.00	A	C
ATOM	1194	CB	SER	A	167	18.892	13.151	-15.719	1.00	15.00	A	C
ATOM	1195	OG	SER	A	167	18.749	11.752	-15.554	1.00	15.00	A	O
ATOM	1196	C	SER	A	167	17.261	13.954	-13.948	1.00	15.00	A	C

```
ATOM   1197  O   SER A 167      16.576  14.955 -14.175  1.00 15.00      A    O
ATOM   1198  N   TRP A 168      16.796  12.897 -13.288  1.00 15.00      A    N
ATOM   1199  CA  TRP A 168      15.417  12.831 -12.818  1.00 15.00      A    C
ATOM   1200  CB  TRP A 168      15.139  11.465 -12.172  1.00 15.00      A    C
ATOM   1201  CG  TRP A 168      13.711  11.258 -11.764  1.00 15.00      A    C
ATOM   1202  CD1 TRP A 168      13.252  10.972 -10.512  1.00 15.00      A    C
ATOM   1203  CD2 TRP A 168      12.557  11.320 -12.611  1.00 15.00      A    C
ATOM   1204  NE1 TRP A 168      11.881  10.850 -10.529  1.00 15.00      A    N
ATOM   1205  CE2 TRP A 168      11.433  11.059 -11.807  1.00 15.00      A    C
ATOM   1206  CE3 TRP A 168      12.365  11.571 -13.974  1.00 15.00      A    C
ATOM   1207  CZ2 TRP A 168      10.135  11.041 -12.320  1.00 15.00      A    C
ATOM   1208  CZ3 TRP A 168      11.078  11.553 -14.480  1.00 15.00      A    C
ATOM   1209  CH2 TRP A 168       9.979  11.290 -13.655  1.00 15.00      A    C
ATOM   1210  C   TRP A 168      15.093  13.960 -11.835  1.00 15.00      A    C
ATOM   1211  O   TRP A 168      13.948  14.408 -11.737  1.00 15.00      A    O
ATOM   1212  N   ARG A 169      16.099  14.443 -11.128  1.00 15.00      A    N
ATOM   1213  CA  ARG A 169      15.883  15.490 -10.141  1.00 15.00      A    C
ATOM   1214  CB  ARG A 169      16.587  15.167  -8.820  1.00 15.00      A    C
ATOM   1215  CG  ARG A 169      15.752  14.306  -7.880  1.00 15.00      A    C
ATOM   1216  CD  ARG A 169      16.612  13.405  -7.003  1.00 15.00      A    C
ATOM   1217  NE  ARG A 169      17.054  12.220  -7.728  1.00 15.00      A    N
ATOM   1218  CZ  ARG A 169      16.622  10.980  -7.464  1.00 15.00      A    C
ATOM   1219  NH1 ARG A 169      15.736  10.767  -6.498  1.00 15.00      A    N
ATOM   1220  NH2 ARG A 169      17.048   9.953  -8.180  1.00 15.00      A    N
ATOM   1221  C   ARG A 169      16.247  16.882 -10.652  1.00 15.00      A    C
ATOM   1222  O   ARG A 169      15.619  17.865 -10.267  1.00 15.00      A    O
ATOM   1223  N   SER A 170      17.242  16.966 -11.526  1.00 15.00      A    N
ATOM   1224  CA  SER A 170      17.672  18.251 -12.078  1.00 15.00      A    C
ATOM   1225  CB  SER A 170      19.036  18.134 -12.777  1.00 15.00      A    C
ATOM   1226  OG  SER A 170      19.209  16.857 -13.358  1.00 15.00      A    O
ATOM   1227  C   SER A 170      16.631  18.822 -13.037  1.00 15.00      A    C
ATOM   1228  O   SER A 170      16.440  20.035 -13.114  1.00 15.00      A    O
ATOM   1229  N   GLU A 171      15.957  17.931 -13.746  1.00 15.00      A    N
ATOM   1230  CA  GLU A 171      14.940  18.323 -14.710  1.00 15.00      A    C
ATOM   1231  CB  GLU A 171      14.952  17.353 -15.897  1.00 15.00      A    C
ATOM   1232  CG  GLU A 171      14.902  18.018 -17.265  1.00 15.00      A    C
ATOM   1233  CD  GLU A 171      13.530  18.563 -17.604  1.00 15.00      A    C
ATOM   1234  OE1 GLU A 171      13.387  19.802 -17.694  1.00 15.00      A    O
ATOM   1235  OE2 GLU A 171      12.593  17.754 -17.768  1.00 15.00      A    O
ATOM   1236  C   GLU A 171      13.557  18.364 -14.065  1.00 15.00      A    C
ATOM   1237  O   GLU A 171      13.101  19.418 -13.619  1.00 15.00      A    O
ATOM   1238  N   VAL A 172      12.915  17.204 -13.999  1.00 15.00      A    N
ATOM   1239  CA  VAL A 172      11.580  17.070 -13.424  1.00 15.00      A    C
ATOM   1240  CB  VAL A 172      11.120  15.596 -13.490  1.00 15.00      A    C
ATOM   1241  CG1 VAL A 172       9.733  15.416 -12.890  1.00 15.00      A    C
ATOM   1242  CG2 VAL A 172      11.153  15.097 -14.925  1.00 15.00      A    C
ATOM   1243  C   VAL A 172      11.521  17.559 -11.973  1.00 15.00      A    C
ATOM   1244  O   VAL A 172      10.581  18.252 -11.581  1.00 15.00      A    O
ATOM   1245  N   GLY A 173      12.534  17.202 -11.190  1.00 15.00      A    N
ATOM   1246  CA  GLY A 173      12.577  17.589  -9.786  1.00 15.00      A    C
ATOM   1247  C   GLY A 173      12.440  19.086  -9.543  1.00 15.00      A    C
ATOM   1248  O   GLY A 173      11.671  19.506  -8.678  1.00 15.00      A    O
ATOM   1249  N   LYS A 174      13.172  19.894 -10.304  1.00 15.00      A    N
ATOM   1250  CA  LYS A 174      13.126  21.347 -10.140  1.00 15.00      A    C
ATOM   1251  CB  LYS A 174      14.262  22.034 -10.909  1.00 15.00      A    C
ATOM   1252  CG  LYS A 174      15.655  21.709 -10.386  1.00 15.00      A    C
ATOM   1253  CD  LYS A 174      16.693  22.687 -10.918  1.00 15.00      A    C
ATOM   1254  CE  LYS A 174      18.106  22.198 -10.641  1.00 15.00      A    C
ATOM   1255  NZ  LYS A 174      18.912  23.190  -9.874  1.00 15.00      A    N
ATOM   1256  C   LYS A 174      11.770  21.928 -10.545  1.00 15.00      A    C
ATOM   1257  O   LYS A 174      11.307  22.914  -9.965  1.00 15.00      A    O
ATOM   1258  N   GLN A 175      11.130  21.305 -11.528  1.00 15.00      A    N
ATOM   1259  CA  GLN A 175       9.829  21.768 -12.005  1.00 15.00      A    C
ATOM   1260  CB  GLN A 175       9.433  21.045 -13.293  1.00 15.00      A    C
ATOM   1261  CG  GLN A 175      10.413  21.180 -14.444  1.00 15.00      A    C
ATOM   1262  CD  GLN A 175      10.039  20.265 -15.591  1.00 15.00      A    C
ATOM   1263  OE1 GLN A 175       8.864  19.976 -15.806  1.00 15.00      A    O
ATOM   1264  NE2 GLN A 175      11.028  19.802 -16.328  1.00 15.00      A    N
ATOM   1265  C   GLN A 175       8.738  21.545 -10.958  1.00 15.00      A    C
ATOM   1266  O   GLN A 175       7.719  22.238 -10.952  1.00 15.00      A    O
ATOM   1267  N   LEU A 176       8.968  20.593 -10.062  1.00 15.00      A    N
```

ATOM	1268	CA	LEU	A	176	7.995	20.253	-9.035	1.00	15.00	A	C
ATOM	1269	CB	LEU	A	176	8.114	18.771	-8.662	1.00	15.00	A	C
ATOM	1270	CG	LEU	A	176	7.763	17.777	-9.771	1.00	15.00	A	C
ATOM	1271	CD1	LEU	A	176	8.148	16.363	-9.373	1.00	15.00	A	C
ATOM	1272	CD2	LEU	A	176	6.283	17.856	-10.108	1.00	15.00	A	C
ATOM	1273	C	LEU	A	176	8.138	21.121	-7.790	1.00	15.00	A	C
ATOM	1274	O	LEU	A	176	7.269	21.090	-6.920	1.00	15.00	A	O
ATOM	1275	N	ARG	A	177	9.219	21.905	-7.709	1.00	15.00	A	N
ATOM	1276	CA	ARG	A	177	9.454	22.757	-6.539	1.00	15.00	A	C
ATOM	1277	CB	ARG	A	177	10.883	23.342	-6.473	1.00	15.00	A	C
ATOM	1278	CG	ARG	A	177	11.037	24.545	-5.535	1.00	15.00	A	C
ATOM	1279	CD	ARG	A	177	10.857	24.178	-4.065	1.00	15.00	A	C
ATOM	1280	NE	ARG	A	177	12.094	23.640	-3.473	1.00	15.00	A	N
ATOM	1281	CZ	ARG	A	177	12.650	24.097	-2.346	1.00	15.00	A	C
ATOM	1282	NH1	ARG	A	177	12.095	25.084	-1.679	1.00	15.00	A	N
ATOM	1283	NH2	ARG	A	177	13.744	23.526	-1.870	1.00	15.00	A	N
ATOM	1284	C	ARG	A	177	8.379	23.834	-6.351	1.00	15.00	A	C
ATOM	1285	O	ARG	A	177	7.706	23.850	-5.319	1.00	15.00	A	O
ATOM	1286	N	PRO	A	178	8.209	24.764	-7.321	1.00	15.00	A	N
ATOM	1287	CA	PRO	A	178	7.207	25.827	-7.209	1.00	15.00	A	C
ATOM	1288	CB	PRO	A	178	7.271	26.554	-8.563	1.00	15.00	A	C
ATOM	1289	CG	PRO	A	178	8.073	25.678	-9.464	1.00	15.00	A	C
ATOM	1290	CD	PRO	A	178	8.978	24.878	-8.575	1.00	15.00	A	C
ATOM	1291	C	PRO	A	178	5.810	25.270	-6.966	1.00	15.00	A	C
ATOM	1292	O	PRO	A	178	5.026	25.853	-6.218	1.00	15.00	A	O
ATOM	1293	N	LEU	A	179	5.521	24.131	-7.594	1.00	15.00	A	N
ATOM	1294	CA	LEU	A	179	4.221	23.488	-7.460	1.00	15.00	A	C
ATOM	1295	CB	LEU	A	179	4.072	22.370	-8.496	1.00	15.00	A	C
ATOM	1296	CG	LEU	A	179	4.066	22.794	-9.966	1.00	15.00	A	C
ATOM	1297	CD1	LEU	A	179	3.998	21.575	-10.875	1.00	15.00	A	C
ATOM	1298	CD2	LEU	A	179	2.904	23.735	-10.246	1.00	15.00	A	C
ATOM	1299	C	LEU	A	179	4.016	22.926	-6.057	1.00	15.00	A	C
ATOM	1300	O	LEU	A	179	2.973	23.144	-5.440	1.00	15.00	A	O
ATOM	1301	N	TYR	A	180	5.018	22.216	-5.548	1.00	15.00	A	N
ATOM	1302	CA	TYR	A	180	4.927	21.615	-4.221	1.00	15.00	A	C
ATOM	1303	CB	TYR	A	180	6.149	20.738	-3.928	1.00	15.00	A	C
ATOM	1304	CG	TYR	A	180	5.901	19.678	-2.876	1.00	15.00	A	C
ATOM	1305	CD1	TYR	A	180	5.264	18.488	-3.203	1.00	15.00	A	C
ATOM	1306	CD2	TYR	A	180	6.296	19.867	-1.556	1.00	15.00	A	C
ATOM	1307	CE1	TYR	A	180	5.027	17.518	-2.249	1.00	15.00	A	C
ATOM	1308	CE2	TYR	A	180	6.064	18.901	-0.594	1.00	15.00	A	C
ATOM	1309	CZ	TYR	A	180	5.429	17.728	-0.946	1.00	15.00	A	C
ATOM	1310	OH	TYR	A	180	5.190	16.763	0.011	1.00	15.00	A	O
ATOM	1311	C	TYR	A	180	4.763	22.677	-3.142	1.00	15.00	A	C
ATOM	1312	O	TYR	A	180	4.077	22.460	-2.142	1.00	15.00	A	O
ATOM	1313	N	GLU	A	181	5.371	23.834	-3.363	1.00	15.00	A	N
ATOM	1314	CA	GLU	A	181	5.298	24.925	-2.402	1.00	15.00	A	C
ATOM	1315	CB	GLU	A	181	6.259	26.054	-2.769	1.00	15.00	A	C
ATOM	1316	CG	GLU	A	181	7.718	25.617	-2.802	1.00	15.00	A	C
ATOM	1317	CD	GLU	A	181	8.686	26.773	-2.687	1.00	15.00	A	C
ATOM	1318	OE1	GLU	A	181	8.385	27.858	-3.224	1.00	15.00	A	O
ATOM	1319	OE2	GLU	A	181	9.743	26.607	-2.037	1.00	15.00	A	O
ATOM	1320	C	GLU	A	181	3.869	25.430	-2.223	1.00	15.00	A	C
ATOM	1321	O	GLU	A	181	3.377	25.520	-1.096	1.00	15.00	A	O
ATOM	1322	N	GLU	A	182	3.194	25.743	-3.327	1.00	15.00	A	N
ATOM	1323	CA	GLU	A	182	1.818	26.222	-3.249	1.00	15.00	A	C
ATOM	1324	CB	GLU	A	182	1.339	26.793	-4.584	1.00	15.00	A	C
ATOM	1325	CG	GLU	A	182	0.172	27.758	-4.438	1.00	15.00	A	C
ATOM	1326	CD	GLU	A	182	-0.348	28.272	-5.763	1.00	15.00	A	C
ATOM	1327	OE1	GLU	A	182	0.473	28.629	-6.630	1.00	15.00	A	O
ATOM	1328	OE2	GLU	A	182	-1.582	28.331	-5.929	1.00	15.00	A	O
ATOM	1329	C	GLU	A	182	0.902	25.095	-2.784	1.00	15.00	A	C
ATOM	1330	O	GLU	A	182	-0.089	25.326	-2.086	1.00	15.00	A	O
ATOM	1331	N	TYR	A	183	1.258	23.871	-3.164	1.00	15.00	A	N
ATOM	1332	CA	TYR	A	183	0.502	22.690	-2.777	1.00	15.00	A	C
ATOM	1333	CB	TYR	A	183	1.134	21.436	-3.404	1.00	15.00	A	C
ATOM	1334	CG	TYR	A	183	0.972	20.156	-2.608	1.00	15.00	A	C
ATOM	1335	CD1	TYR	A	183	1.985	19.708	-1.768	1.00	15.00	A	C
ATOM	1336	CD2	TYR	A	183	-0.186	19.396	-2.698	1.00	15.00	A	C
ATOM	1337	CE1	TYR	A	183	1.848	18.543	-1.040	1.00	15.00	A	C
ATOM	1338	CE2	TYR	A	183	-0.331	18.228	-1.973	1.00	15.00	A	C

ATOM	1339	CZ	TYR	A	183	0.689	17.806	-1.145	1.00	15.00	A	C
ATOM	1340	OH	TYR	A	183	0.548	16.648	-0.416	1.00	15.00	A	O
ATOM	1341	C	TYR	A	183	0.444	22.577	-1.255	1.00	15.00	A	C
ATOM	1342	O	TYR	A	183	-0.616	22.324	-0.684	1.00	15.00	A	O
ATOM	1343	N	VAL	A	184	1.586	22.790	-0.606	1.00	15.00	A	N
ATOM	1344	CA	VAL	A	184	1.673	22.714	0.849	1.00	15.00	A	C
ATOM	1345	CB	VAL	A	184	3.135	22.852	1.341	1.00	15.00	A	C
ATOM	1346	CG1	VAL	A	184	3.197	23.007	2.855	1.00	15.00	A	C
ATOM	1347	CG2	VAL	A	184	3.956	21.650	0.903	1.00	15.00	A	C
ATOM	1348	C	VAL	A	184	0.806	23.787	1.505	1.00	15.00	A	C
ATOM	1349	O	VAL	A	184	0.114	23.524	2.489	1.00	15.00	A	O
ATOM	1350	N	VAL	A	185	0.829	24.986	0.936	1.00	15.00	A	N
ATOM	1351	CA	VAL	A	185	0.055	26.102	1.469	1.00	15.00	A	C
ATOM	1352	CB	VAL	A	185	0.384	27.423	0.738	1.00	15.00	A	C
ATOM	1353	CG1	VAL	A	185	-0.447	28.573	1.294	1.00	15.00	A	C
ATOM	1354	CG2	VAL	A	185	1.868	27.738	0.848	1.00	15.00	A	C
ATOM	1355	C	VAL	A	185	-1.445	25.829	1.387	1.00	15.00	A	C
ATOM	1356	O	VAL	A	185	-2.144	25.856	2.401	1.00	15.00	A	O
ATOM	1357	N	LEU	A	186	-1.923	25.532	0.186	1.00	15.00	A	N
ATOM	1358	CA	LEU	A	186	-3.342	25.272	-0.033	1.00	15.00	A	C
ATOM	1359	CB	LEU	A	186	-3.645	25.136	-1.525	1.00	15.00	A	C
ATOM	1360	CG	LEU	A	186	-3.331	26.365	-2.382	1.00	15.00	A	C
ATOM	1361	CD1	LEU	A	186	-3.596	26.082	-3.851	1.00	15.00	A	C
ATOM	1362	CD2	LEU	A	186	-4.134	27.569	-1.914	1.00	15.00	A	C
ATOM	1363	C	LEU	A	186	-3.839	24.057	0.747	1.00	15.00	A	C
ATOM	1364	O	LEU	A	186	-4.952	24.063	1.273	1.00	15.00	A	O
ATOM	1365	N	LYS	A	187	-3.015	23.018	0.830	1.00	15.00	A	N
ATOM	1366	CA	LYS	A	187	-3.391	21.814	1.564	1.00	15.00	A	C
ATOM	1367	CB	LYS	A	187	-2.421	20.669	1.276	1.00	15.00	A	C
ATOM	1368	CG	LYS	A	187	-2.659	19.973	-0.053	1.00	15.00	A	C
ATOM	1369	CD	LYS	A	187	-3.927	19.135	-0.025	1.00	15.00	A	C
ATOM	1370	CE	LYS	A	187	-3.999	18.209	-1.228	1.00	15.00	A	C
ATOM	1371	NZ	LYS	A	187	-4.758	16.969	-0.927	1.00	15.00	A	N
ATOM	1372	C	LYS	A	187	-3.450	22.095	3.059	1.00	15.00	A	C
ATOM	1373	O	LYS	A	187	-4.291	21.547	3.773	1.00	15.00	A	O
ATOM	1374	N	ASN	A	188	-2.554	22.956	3.522	1.00	15.00	A	N
ATOM	1375	CA	ASN	A	188	-2.504	23.330	4.927	1.00	15.00	A	C
ATOM	1376	CB	ASN	A	188	-1.257	24.160	5.216	1.00	15.00	A	C
ATOM	1377	CG	ASN	A	188	-0.407	23.564	6.313	1.00	15.00	A	C
ATOM	1378	OD1	ASN	A	188	-0.916	22.966	7.261	1.00	15.00	A	O
ATOM	1379	ND2	ASN	A	188	0.899	23.716	6.187	1.00	15.00	A	N
ATOM	1380	C	ASN	A	188	-3.744	24.123	5.297	1.00	15.00	A	C
ATOM	1381	O	ASN	A	188	-4.330	23.918	6.362	1.00	15.00	A	O
ATOM	1382	N	GLU	A	189	-4.135	25.034	4.408	1.00	15.00	A	N
ATOM	1383	CA	GLU	A	189	-5.329	25.839	4.616	1.00	15.00	A	C
ATOM	1384	CB	GLU	A	189	-5.549	26.785	3.436	1.00	15.00	A	C
ATOM	1385	CG	GLU	A	189	-4.563	27.935	3.358	1.00	15.00	A	C
ATOM	1386	CD	GLU	A	189	-5.040	29.027	2.428	1.00	15.00	A	C
ATOM	1387	OE1	GLU	A	189	-4.280	29.416	1.512	1.00	15.00	A	O
ATOM	1388	OE2	GLU	A	189	-6.185	29.503	2.596	1.00	15.00	A	O
ATOM	1389	C	GLU	A	189	-6.521	24.908	4.741	1.00	15.00	A	C
ATOM	1390	O	GLU	A	189	-7.360	25.053	5.632	1.00	15.00	A	O
ATOM	1391	N	MET	A	190	-6.561	23.935	3.838	1.00	15.00	A	N
ATOM	1392	CA	MET	A	190	-7.612	22.929	3.816	1.00	15.00	A	C
ATOM	1393	CB	MET	A	190	-7.356	21.949	2.664	1.00	15.00	A	C
ATOM	1394	CG	MET	A	190	-8.139	20.653	2.749	1.00	15.00	A	C
ATOM	1395	SD	MET	A	190	-7.161	19.217	2.255	1.00	15.00	A	S
ATOM	1396	CE	MET	A	190	-8.213	17.893	2.843	1.00	15.00	A	C
ATOM	1397	C	MET	A	190	-7.674	22.179	5.148	1.00	15.00	A	C
ATOM	1398	O	MET	A	190	-8.748	22.016	5.730	1.00	15.00	A	O
ATOM	1399	N	ALA	A	191	-6.514	21.740	5.629	1.00	15.00	A	N
ATOM	1400	CA	ALA	A	191	-6.420	21.013	6.891	1.00	15.00	A	C
ATOM	1401	CB	ALA	A	191	-5.006	20.494	7.098	1.00	15.00	A	C
ATOM	1402	C	ALA	A	191	-6.846	21.882	8.071	1.00	15.00	A	C
ATOM	1403	O	ALA	A	191	-7.600	21.441	8.933	1.00	15.00	A	O
ATOM	1404	N	ARG	A	192	-6.375	23.127	8.100	1.00	15.00	A	N
ATOM	1405	CA	ARG	A	192	-6.725	24.050	9.181	1.00	15.00	A	C
ATOM	1406	CB	ARG	A	192	-5.942	25.367	9.077	1.00	15.00	A	C
ATOM	1407	CG	ARG	A	192	-4.728	25.433	9.998	1.00	15.00	A	C
ATOM	1408	CD	ARG	A	192	-3.416	25.497	9.220	1.00	15.00	A	C
ATOM	1409	NE	ARG	A	192	-2.360	24.721	9.878	1.00	15.00	A	N

ATOM	1410	CZ	ARG	A	192	-1.055	24.904	9.682	1.00	15.00	A	C
ATOM	1411	NH1	ARG	A	192	-0.622	25.844	8.841	1.00	15.00	A	N
ATOM	1412	NH2	ARG	A	192	-0.185	24.127	10.308	1.00	15.00	A	N
ATOM	1413	C	ARG	A	192	-8.226	24.317	9.203	1.00	15.00	A	C
ATOM	1414	O	ARG	A	192	-8.844	24.363	10.267	1.00	15.00	A	O
ATOM	1415	N	ALA	A	193	-8.814	24.458	8.020	1.00	15.00	A	N
ATOM	1416	CA	ALA	A	193	-10.250	24.701	7.897	1.00	15.00	A	C
ATOM	1417	CB	ALA	A	193	-10.594	25.197	6.498	1.00	15.00	A	C
ATOM	1418	C	ALA	A	193	-11.052	23.445	8.264	1.00	15.00	A	C
ATOM	1419	O	ALA	A	193	-12.260	23.507	8.476	1.00	15.00	A	O
ATOM	1420	N	ASN	A	194	-10.361	22.309	8.336	1.00	15.00	A	N
ATOM	1421	CA	ASN	A	194	-10.987	21.042	8.699	1.00	15.00	A	C
ATOM	1422	CB	ASN	A	194	-10.403	19.890	7.862	1.00	15.00	A	C
ATOM	1423	CG	ASN	A	194	-11.202	19.581	6.606	1.00	15.00	A	C
ATOM	1424	OD1	ASN	A	194	-11.997	18.644	6.574	1.00	15.00	A	O
ATOM	1425	ND2	ASN	A	194	-10.985	20.359	5.559	1.00	15.00	A	N
ATOM	1426	C	ASN	A	194	-10.755	20.772	10.183	1.00	15.00	A	C
ATOM	1427	O	ASN	A	194	-11.061	19.689	10.679	1.00	15.00	A	O
ATOM	1428	N	HIS	A	195	-10.188	21.772	10.872	1.00	15.00	A	N
ATOM	1429	CA	HIS	A	195	-9.905	21.696	12.313	1.00	15.00	A	C
ATOM	1430	CB	HIS	A	195	-11.153	21.321	13.130	1.00	15.00	A	C
ATOM	1431	CG	HIS	A	195	-12.278	22.311	13.023	1.00	15.00	A	C
ATOM	1432	ND1	HIS	A	195	-13.584	21.901	12.877	1.00	15.00	A	N
ATOM	1433	CD2	HIS	A	195	-12.238	23.667	13.036	1.00	15.00	A	C
ATOM	1434	CE1	HIS	A	195	-14.305	23.003	12.802	1.00	15.00	A	C
ATOM	1435	NE2	HIS	A	195	-13.535	24.095	12.894	1.00	15.00	A	N
ATOM	1436	C	HIS	A	195	-8.703	20.806	12.665	1.00	15.00	A	C
ATOM	1437	O	HIS	A	195	-8.655	20.212	13.744	1.00	15.00	A	O
ATOM	1438	N	TYR	A	196	-7.725	20.730	11.768	1.00	15.00	A	N
ATOM	1439	CA	TYR	A	196	-6.521	19.931	12.009	1.00	15.00	A	C
ATOM	1440	CB	TYR	A	196	-6.240	19.017	10.816	1.00	15.00	A	C
ATOM	1441	CG	TYR	A	196	-7.190	17.844	10.717	1.00	15.00	A	C
ATOM	1442	CD1	TYR	A	196	-8.427	17.972	10.100	1.00	15.00	A	C
ATOM	1443	CD2	TYR	A	196	-6.852	16.610	11.250	1.00	15.00	A	C
ATOM	1444	CE1	TYR	A	196	-9.301	16.903	10.017	1.00	15.00	A	C
ATOM	1445	CE2	TYR	A	196	-7.716	15.536	11.170	1.00	15.00	A	C
ATOM	1446	CZ	TYR	A	196	-8.938	15.688	10.555	1.00	15.00	A	C
ATOM	1447	OH	TYR	A	196	-9.799	14.618	10.477	1.00	15.00	A	O
ATOM	1448	C	TYR	A	196	-5.321	20.840	12.278	1.00	15.00	A	C
ATOM	1449	O	TYR	A	196	-5.347	22.018	11.916	1.00	15.00	A	O
ATOM	1450	N	GLU	A	197	-4.271	20.304	12.904	1.00	15.00	A	N
ATOM	1451	CA	GLU	A	197	-3.091	21.115	13.208	1.00	15.00	A	C
ATOM	1452	CB	GLU	A	197	-2.280	20.578	14.401	1.00	15.00	A	C
ATOM	1453	CG	GLU	A	197	-1.449	19.335	14.120	1.00	15.00	A	C
ATOM	1454	CD	GLU	A	197	-0.408	19.064	15.191	1.00	15.00	A	C
ATOM	1455	OE1	GLU	A	197	-0.434	19.739	16.240	1.00	15.00	A	O
ATOM	1456	OE2	GLU	A	197	0.449	18.178	14.979	1.00	15.00	A	O
ATOM	1457	C	GLU	A	197	-2.220	21.339	11.971	1.00	15.00	A	C
ATOM	1458	O	GLU	A	197	-1.749	22.453	11.727	1.00	15.00	A	O
ATOM	1459	N	ASP	A	198	-2.012	20.285	11.189	1.00	15.00	A	N
ATOM	1460	CA	ASP	A	198	-1.218	20.372	9.963	1.00	15.00	A	C
ATOM	1461	CB	ASP	A	198	0.281	20.137	10.228	1.00	15.00	A	C
ATOM	1462	CG	ASP	A	198	0.674	18.681	10.405	1.00	15.00	A	C
ATOM	1463	OD1	ASP	A	198	1.113	18.058	9.421	1.00	15.00	A	O
ATOM	1464	OD2	ASP	A	198	0.570	18.164	11.531	1.00	15.00	A	O
ATOM	1465	C	ASP	A	198	-1.775	19.418	8.909	1.00	15.00	A	C
ATOM	1466	O	ASP	A	198	-2.684	18.643	9.202	1.00	15.00	A	O
ATOM	1467	N	TYR	A	199	-1.259	19.478	7.681	1.00	15.00	A	N
ATOM	1468	CA	TYR	A	199	-1.754	18.597	6.621	1.00	15.00	A	C
ATOM	1469	CB	TYR	A	199	-1.224	18.975	5.234	1.00	15.00	A	C
ATOM	1470	CG	TYR	A	199	-1.831	18.137	4.124	1.00	15.00	A	C
ATOM	1471	CD1	TYR	A	199	-3.202	17.905	4.075	1.00	15.00	A	C
ATOM	1472	CD2	TYR	A	199	-1.038	17.565	3.140	1.00	15.00	A	C
ATOM	1473	CE1	TYR	A	199	-3.762	17.129	3.081	1.00	15.00	A	C
ATOM	1474	CE2	TYR	A	199	-1.591	16.789	2.138	1.00	15.00	A	C
ATOM	1475	CZ	TYR	A	199	-2.952	16.573	2.116	1.00	15.00	A	C
ATOM	1476	OH	TYR	A	199	-3.509	15.800	1.122	1.00	15.00	A	O
ATOM	1477	C	TYR	A	199	-1.473	17.133	6.938	1.00	15.00	A	C
ATOM	1478	O	TYR	A	199	-2.280	16.254	6.632	1.00	15.00	A	O
ATOM	1479	N	GLY	A	200	-0.336	16.878	7.565	1.00	15.00	A	N
ATOM	1480	CA	GLY	A	200	0.014	15.528	7.934	1.00	15.00	A	C

ATOM	1481	C	GLY	A	200	-0.924	15.026	9.006	1.00	15.00	A	C
ATOM	1482	O	GLY	A	200	-1.217	13.836	9.077	1.00	15.00	A	O
ATOM	1483	N	ASP	A	201	-1.405	15.953	9.832	1.00	15.00	A	N
ATOM	1484	CA	ASP	A	201	-2.344	15.627	10.903	1.00	15.00	A	C
ATOM	1485	CB	ASP	A	201	-2.675	16.878	11.725	1.00	15.00	A	C
ATOM	1486	CG	ASP	A	201	-3.478	16.574	12.974	1.00	15.00	A	C
ATOM	1487	OD1	ASP	A	201	-3.262	15.504	13.583	1.00	15.00	A	O
ATOM	1488	OD2	ASP	A	201	-4.325	17.413	13.355	1.00	15.00	A	O
ATOM	1489	C	ASP	A	201	-3.612	15.052	10.299	1.00	15.00	A	C
ATOM	1490	O	ASP	A	201	-4.227	14.143	10.852	1.00	15.00	A	O
ATOM	1491	N	TYR	A	202	-3.975	15.579	9.136	1.00	15.00	A	N
ATOM	1492	CA	TYR	A	202	-5.148	15.122	8.412	1.00	15.00	A	C
ATOM	1493	CB	TYR	A	202	-5.404	16.037	7.209	1.00	15.00	A	C
ATOM	1494	CG	TYR	A	202	-6.616	15.668	6.383	1.00	15.00	A	C
ATOM	1495	CD1	TYR	A	202	-6.475	15.030	5.158	1.00	15.00	A	C
ATOM	1496	CD2	TYR	A	202	-7.899	15.962	6.826	1.00	15.00	A	C
ATOM	1497	CE1	TYR	A	202	-7.577	14.694	4.397	1.00	15.00	A	C
ATOM	1498	CE2	TYR	A	202	-9.008	15.628	6.072	1.00	15.00	A	C
ATOM	1499	CZ	TYR	A	202	-8.841	14.995	4.858	1.00	15.00	A	C
ATOM	1500	OH	TYR	A	202	-9.942	14.664	4.099	1.00	15.00	A	O
ATOM	1501	C	TYR	A	202	-4.946	13.677	7.957	1.00	15.00	A	C
ATOM	1502	O	TYR	A	202	-5.886	12.885	7.922	1.00	15.00	A	O
ATOM	1503	N	TRP	A	203	-3.704	13.338	7.627	1.00	15.00	A	N
ATOM	1504	CA	TRP	A	203	-3.366	11.987	7.191	1.00	15.00	A	C
ATOM	1505	CB	TRP	A	203	-2.021	11.974	6.459	1.00	15.00	A	C
ATOM	1506	CG	TRP	A	203	-2.090	12.416	5.028	1.00	15.00	A	C
ATOM	1507	CD1	TRP	A	203	-3.209	12.526	4.252	1.00	15.00	A	C
ATOM	1508	CD2	TRP	A	203	-0.987	12.797	4.196	1.00	15.00	A	C
ATOM	1509	NE1	TRP	A	203	-2.869	12.955	2.991	1.00	15.00	A	N
ATOM	1510	CE2	TRP	A	203	-1.511	13.127	2.932	1.00	15.00	A	C
ATOM	1511	CE3	TRP	A	203	0.392	12.893	4.401	1.00	15.00	A	C
ATOM	1512	CZ2	TRP	A	203	-0.704	13.544	1.876	1.00	15.00	A	C
ATOM	1513	CZ3	TRP	A	203	1.191	13.307	3.352	1.00	15.00	A	C
ATOM	1514	CH2	TRP	A	203	0.641	13.628	2.106	1.00	15.00	A	C
ATOM	1515	C	TRP	A	203	-3.309	11.049	8.392	1.00	15.00	A	C
ATOM	1516	O	TRP	A	203	-3.564	9.853	8.280	1.00	15.00	A	O
ATOM	1517	N	ARG	A	204	-2.994	11.612	9.552	1.00	15.00	A	N
ATOM	1518	CA	ARG	A	204	-2.900	10.840	10.785	1.00	15.00	A	C
ATOM	1519	CB	ARG	A	204	-2.011	11.570	11.809	1.00	15.00	A	C
ATOM	1520	CG	ARG	A	204	-0.616	11.911	11.297	1.00	15.00	A	C
ATOM	1521	CD	ARG	A	204	0.295	12.454	12.396	1.00	15.00	A	C
ATOM	1522	NE	ARG	A	204	-0.095	13.789	12.864	1.00	15.00	A	N
ATOM	1523	CZ	ARG	A	204	0.470	14.932	12.458	1.00	15.00	A	C
ATOM	1524	NH1	ARG	A	204	1.453	14.926	11.561	1.00	15.00	A	N
ATOM	1525	NH2	ARG	A	204	0.067	16.083	12.973	1.00	15.00	A	N
ATOM	1526	C	ARG	A	204	-4.289	10.589	11.375	1.00	15.00	A	C
ATOM	1527	O	ARG	A	204	-4.421	9.979	12.435	1.00	15.00	A	O
ATOM	1528	N	GLY	A	205	-5.319	11.044	10.665	1.00	15.00	A	N
ATOM	1529	CA	GLY	A	205	-6.686	10.892	11.135	1.00	15.00	A	C
ATOM	1530	C	GLY	A	205	-7.292	9.524	10.884	1.00	15.00	A	C
ATOM	1531	O	GLY	A	205	-8.259	9.150	11.541	1.00	15.00	A	O
ATOM	1532	N	ASP	A	206	-6.726	8.768	9.948	1.00	15.00	A	N
ATOM	1533	CA	ASP	A	206	-7.250	7.436	9.626	1.00	15.00	A	C
ATOM	1534	CB	ASP	A	206	-6.598	6.886	8.354	1.00	15.00	A	C
ATOM	1535	CG	ASP	A	206	-7.181	5.551	7.917	1.00	15.00	A	C
ATOM	1536	OD1	ASP	A	206	-8.388	5.505	7.576	1.00	15.00	A	O
ATOM	1537	OD2	ASP	A	206	-6.431	4.551	7.902	1.00	15.00	A	O
ATOM	1538	C	ASP	A	206	-7.060	6.462	10.788	1.00	15.00	A	C
ATOM	1539	O	ASP	A	206	-7.800	5.490	10.935	1.00	15.00	A	O
ATOM	1540	N	TYR	A	207	-6.077	6.747	11.629	1.00	15.00	A	N
ATOM	1541	CA	TYR	A	207	-5.782	5.899	12.774	1.00	15.00	A	C
ATOM	1542	CB	TYR	A	207	-4.266	5.806	12.990	1.00	15.00	A	C
ATOM	1543	CG	TYR	A	207	-3.473	5.303	11.799	1.00	15.00	A	C
ATOM	1544	CD1	TYR	A	207	-3.155	6.146	10.740	1.00	15.00	A	C
ATOM	1545	CD2	TYR	A	207	-3.025	3.989	11.744	1.00	15.00	A	C
ATOM	1546	CE1	TYR	A	207	-2.416	5.695	9.661	1.00	15.00	A	C
ATOM	1547	CE2	TYR	A	207	-2.287	3.529	10.668	1.00	15.00	A	C
ATOM	1548	CZ	TYR	A	207	-1.985	4.385	9.629	1.00	15.00	A	C
ATOM	1549	OH	TYR	A	207	-1.242	3.931	8.560	1.00	15.00	A	O
ATOM	1550	C	TYR	A	207	-6.432	6.451	14.041	1.00	15.00	A	C
ATOM	1551	O	TYR	A	207	-6.288	5.876	15.121	1.00	15.00	A	O

ATOM	1552	N	GLU	A	208	-7.157	7.554	13.904	1.00	15.00	A	N
ATOM	1553	CA	GLU	A	208	-7.794	8.190	15.048	1.00	15.00	A	C
ATOM	1554	CB	GLU	A	208	-8.073	9.667	14.775	1.00	15.00	A	C
ATOM	1555	CG	GLU	A	208	-8.632	10.408	15.976	1.00	15.00	A	C
ATOM	1556	CD	GLU	A	208	-8.734	11.901	15.763	1.00	15.00	A	C
ATOM	1557	OE1	GLU	A	208	-8.745	12.344	14.596	1.00	15.00	A	O
ATOM	1558	OE2	GLU	A	208	-8.801	12.639	16.766	1.00	15.00	A	O
ATOM	1559	C	GLU	A	208	-9.068	7.474	15.484	1.00	15.00	A	C
ATOM	1560	O	GLU	A	208	-9.908	7.104	14.662	1.00	15.00	A	O
ATOM	1561	N	VAL	A	209	-9.196	7.285	16.789	1.00	15.00	A	N
ATOM	1562	CA	VAL	A	209	-10.353	6.628	17.376	1.00	15.00	A	C
ATOM	1563	CB	VAL	A	209	-10.019	5.186	17.825	1.00	15.00	A	C
ATOM	1564	CG1	VAL	A	209	-11.250	4.504	18.399	1.00	15.00	A	C
ATOM	1565	CG2	VAL	A	209	-9.442	4.370	16.677	1.00	15.00	A	C
ATOM	1566	C	VAL	A	209	-10.811	7.425	18.594	1.00	15.00	A	C
ATOM	1567	O	VAL	A	209	-10.043	7.605	19.536	1.00	15.00	A	O
ATOM	1568	N	ASN	A	210	-12.047	7.916	18.561	1.00	15.00	A	N
ATOM	1569	CA	ASN	A	210	-12.589	8.698	19.673	1.00	15.00	A	C
ATOM	1570	CB	ASN	A	210	-12.677	10.186	19.312	1.00	15.00	A	C
ATOM	1571	CG	ASN	A	210	-11.337	10.883	19.212	1.00	15.00	A	C
ATOM	1572	OD1	ASN	A	210	-10.703	11.186	20.220	1.00	15.00	A	O
ATOM	1573	ND2	ASN	A	210	-10.916	11.169	17.992	1.00	15.00	A	N
ATOM	1574	C	ASN	A	210	-13.982	8.222	20.065	1.00	15.00	A	C
ATOM	1575	O	ASN	A	210	-14.958	8.493	19.358	1.00	15.00	A	O
ATOM	1576	N	GLY	A	211	-14.079	7.507	21.174	1.00	15.00	A	N
ATOM	1577	CA	GLY	A	211	-15.375	7.050	21.640	1.00	15.00	A	C
ATOM	1578	C	GLY	A	211	-15.412	5.582	22.007	1.00	15.00	A	C
ATOM	1579	O	GLY	A	211	-16.490	4.993	22.103	1.00	15.00	A	O
ATOM	1580	N	VAL	A	212	-14.250	4.979	22.205	1.00	15.00	A	N
ATOM	1581	CA	VAL	A	212	-14.181	3.572	22.567	1.00	15.00	A	C
ATOM	1582	CB	VAL	A	212	-13.690	2.689	21.398	1.00	15.00	A	C
ATOM	1583	CG1	VAL	A	212	-14.143	1.249	21.594	1.00	15.00	A	C
ATOM	1584	CG2	VAL	A	212	-14.188	3.226	20.063	1.00	15.00	A	C
ATOM	1585	C	VAL	A	212	-13.269	3.389	23.774	1.00	15.00	A	C
ATOM	1586	O	VAL	A	212	-12.045	3.347	23.638	1.00	15.00	A	O
ATOM	1587	N	ASP	A	213	-13.884	3.287	24.947	1.00	15.00	A	N
ATOM	1588	CA	ASP	A	213	-13.166	3.130	26.213	1.00	15.00	A	C
ATOM	1589	CB	ASP	A	213	-14.144	2.837	27.354	1.00	15.00	A	C
ATOM	1590	CG	ASP	A	213	-14.383	4.036	28.248	1.00	15.00	A	C
ATOM	1591	OD1	ASP	A	213	-13.570	4.262	29.168	1.00	15.00	A	O
ATOM	1592	OD2	ASP	A	213	-15.390	4.744	28.034	1.00	15.00	A	O
ATOM	1593	C	ASP	A	213	-12.095	2.045	26.163	1.00	15.00	A	C
ATOM	1594	O	ASP	A	213	-12.395	0.858	26.020	1.00	15.00	A	O
ATOM	1595	N	GLY	A	214	-10.840	2.467	26.265	1.00	15.00	A	N
ATOM	1596	CA	GLY	A	214	-9.735	1.529	26.256	1.00	15.00	A	C
ATOM	1597	C	GLY	A	214	-9.343	1.071	24.866	1.00	15.00	A	C
ATOM	1598	O	GLY	A	214	-8.643	0.064	24.717	1.00	15.00	A	O
ATOM	1599	N	TYR	A	215	-9.814	1.784	23.852	1.00	15.00	A	N
ATOM	1600	CA	TYR	A	215	-9.499	1.457	22.465	1.00	15.00	A	C
ATOM	1601	CB	TYR	A	215	-10.616	0.619	21.833	1.00	15.00	A	C
ATOM	1602	CG	TYR	A	215	-10.693	-0.798	22.350	1.00	15.00	A	C
ATOM	1603	CD1	TYR	A	215	-9.937	-1.809	21.773	1.00	15.00	A	C
ATOM	1604	CD2	TYR	A	215	-11.517	-1.124	23.419	1.00	15.00	A	C
ATOM	1605	CE1	TYR	A	215	-9.998	-3.104	22.248	1.00	15.00	A	C
ATOM	1606	CE2	TYR	A	215	-11.586	-2.416	23.899	1.00	15.00	A	C
ATOM	1607	CZ	TYR	A	215	-10.825	-3.403	23.311	1.00	15.00	A	C
ATOM	1608	OH	TYR	A	215	-10.884	-4.686	23.798	1.00	15.00	A	O
ATOM	1609	C	TYR	A	215	-9.272	2.721	21.642	1.00	15.00	A	C
ATOM	1610	O	TYR	A	215	-9.025	2.649	20.436	1.00	15.00	A	O
ATOM	1611	N	ASP	A	216	-9.362	3.872	22.300	1.00	15.00	A	N
ATOM	1612	CA	ASP	A	216	-9.171	5.158	21.638	1.00	15.00	A	C
ATOM	1613	CB	ASP	A	216	-9.552	6.318	22.565	1.00	15.00	A	C
ATOM	1614	CG	ASP	A	216	-11.034	6.368	22.884	1.00	15.00	A	C
ATOM	1615	OD1	ASP	A	216	-11.857	6.283	21.945	1.00	15.00	A	O
ATOM	1616	OD2	ASP	A	216	-11.378	6.493	24.073	1.00	15.00	A	O
ATOM	1617	C	ASP	A	216	-7.732	5.329	21.168	1.00	15.00	A	C
ATOM	1618	O	ASP	A	216	-6.813	4.696	21.695	1.00	15.00	A	O
ATOM	1619	N	TYR	A	217	-7.542	6.194	20.185	1.00	15.00	A	N
ATOM	1620	CA	TYR	A	217	-6.222	6.454	19.632	1.00	15.00	A	C
ATOM	1621	CB	TYR	A	217	-5.898	5.433	18.535	1.00	15.00	A	C
ATOM	1622	CG	TYR	A	217	-4.430	5.341	18.174	1.00	15.00	A	C

ATOM	1623	CD1	TYR	A	217	-3.918	6.031	17.083	1.00	15.00	A	C
ATOM	1624	CD2	TYR	A	217	-3.559	4.558	18.920	1.00	15.00	A	C
ATOM	1625	CE1	TYR	A	217	-2.583	5.944	16.743	1.00	15.00	A	C
ATOM	1626	CE2	TYR	A	217	-2.219	4.467	18.589	1.00	15.00	A	C
ATOM	1627	CZ	TYR	A	217	-1.737	5.161	17.498	1.00	15.00	A	C
ATOM	1628	OH	TYR	A	217	-0.407	5.075	17.161	1.00	15.00	A	O
ATOM	1629	C	TYR	A	217	-6.174	7.866	19.066	1.00	15.00	A	C
ATOM	1630	O	TYR	A	217	-7.057	8.266	18.309	1.00	15.00	A	O
ATOM	1631	N	SER	A	218	-5.153	8.621	19.439	1.00	15.00	A	N
ATOM	1632	CA	SER	A	218	-5.014	9.988	18.968	1.00	15.00	A	C
ATOM	1633	CB	SER	A	218	-4.485	10.883	20.092	1.00	15.00	A	C
ATOM	1634	OG	SER	A	218	-3.184	10.483	20.491	1.00	15.00	A	O
ATOM	1635	C	SER	A	218	-4.085	10.052	17.760	1.00	15.00	A	C
ATOM	1636	O	SER	A	218	-3.229	9.186	17.577	1.00	15.00	A	O
ATOM	1637	N	ARG	A	219	-4.254	11.084	16.944	1.00	15.00	A	N
ATOM	1638	CA	ARG	A	219	-3.428	11.268	15.754	1.00	15.00	A	C
ATOM	1639	CB	ARG	A	219	-4.011	12.368	14.869	1.00	15.00	A	C
ATOM	1640	CG	ARG	A	219	-5.492	12.192	14.590	1.00	15.00	A	C
ATOM	1641	CD	ARG	A	219	-5.991	13.161	13.535	1.00	15.00	A	C
ATOM	1642	NE	ARG	A	219	-5.782	14.555	13.910	1.00	15.00	A	N
ATOM	1643	CZ	ARG	A	219	-6.691	15.313	14.521	1.00	15.00	A	C
ATOM	1644	NH1	ARG	A	219	-7.882	14.818	14.843	1.00	15.00	A	N
ATOM	1645	NH2	ARG	A	219	-6.419	16.586	14.766	1.00	15.00	A	N
ATOM	1646	C	ARG	A	219	-1.987	11.595	16.134	1.00	15.00	A	C
ATOM	1647	O	ARG	A	219	-1.066	11.413	15.337	1.00	15.00	A	O
ATOM	1648	N	GLY	A	220	-1.802	12.072	17.359	1.00	15.00	A	N
ATOM	1649	CA	GLY	A	220	-0.475	12.408	17.836	1.00	15.00	A	C
ATOM	1650	C	GLY	A	220	0.302	11.176	18.250	1.00	15.00	A	C
ATOM	1651	O	GLY	A	220	1.503	11.081	17.994	1.00	15.00	A	O
ATOM	1652	N	GLN	A	221	-0.401	10.221	18.861	1.00	15.00	A	N
ATOM	1653	CA	GLN	A	221	0.208	8.975	19.323	1.00	15.00	A	C
ATOM	1654	CB	GLN	A	221	-0.853	8.080	19.974	1.00	15.00	A	C
ATOM	1655	CG	GLN	A	221	-0.315	6.771	20.532	1.00	15.00	A	C
ATOM	1656	CD	GLN	A	221	0.285	6.918	21.915	1.00	15.00	A	C
ATOM	1657	OE1	GLN	A	221	-0.202	7.695	22.737	1.00	15.00	A	O
ATOM	1658	NE2	GLN	A	221	1.346	6.173	22.182	1.00	15.00	A	N
ATOM	1659	C	GLN	A	221	0.879	8.233	18.170	1.00	15.00	A	C
ATOM	1660	O	GLN	A	221	1.894	7.563	18.358	1.00	15.00	A	O
ATOM	1661	N	LEU	A	222	0.314	8.377	16.973	1.00	15.00	A	N
ATOM	1662	CA	LEU	A	222	0.852	7.726	15.784	1.00	15.00	A	C
ATOM	1663	CB	LEU	A	222	0.026	8.086	14.545	1.00	15.00	A	C
ATOM	1664	CG	LEU	A	222	0.494	7.468	13.223	1.00	15.00	A	C
ATOM	1665	CD1	LEU	A	222	0.269	5.963	13.218	1.00	15.00	A	C
ATOM	1666	CD2	LEU	A	222	-0.202	8.124	12.042	1.00	15.00	A	C
ATOM	1667	C	LEU	A	222	2.312	8.105	15.567	1.00	15.00	A	C
ATOM	1668	O	LEU	A	222	3.116	7.288	15.127	1.00	15.00	A	O
ATOM	1669	N	ILE	A	223	2.652	9.344	15.893	1.00	15.00	A	N
ATOM	1670	CA	ILE	A	223	4.015	9.821	15.733	1.00	15.00	A	C
ATOM	1671	CB	ILE	A	223	4.107	11.351	15.923	1.00	15.00	A	C
ATOM	1672	CG1	ILE	A	223	3.070	12.062	15.048	1.00	15.00	A	C
ATOM	1673	CG2	ILE	A	223	5.510	11.847	15.597	1.00	15.00	A	C
ATOM	1674	CD1	ILE	A	223	2.908	13.535	15.360	1.00	15.00	A	C
ATOM	1675	C	ILE	A	223	4.920	9.136	16.749	1.00	15.00	A	C
ATOM	1676	O	ILE	A	223	5.934	8.541	16.391	1.00	15.00	A	O
ATOM	1677	N	GLU	A	224	4.512	9.196	18.011	1.00	15.00	A	N
ATOM	1678	CA	GLU	A	224	5.271	8.601	19.102	1.00	15.00	A	C
ATOM	1679	CB	GLU	A	224	4.572	8.864	20.436	1.00	15.00	A	C
ATOM	1680	CG	GLU	A	224	4.359	10.341	20.727	1.00	15.00	A	C
ATOM	1681	CD	GLU	A	224	3.571	10.586	21.997	1.00	15.00	A	C
ATOM	1682	OE1	GLU	A	224	2.343	10.797	21.901	1.00	15.00	A	O
ATOM	1683	OE2	GLU	A	224	4.183	10.575	23.086	1.00	15.00	A	O
ATOM	1684	C	GLU	A	224	5.490	7.106	18.890	1.00	15.00	A	C
ATOM	1685	O	GLU	A	224	6.625	6.632	18.919	1.00	15.00	A	O
ATOM	1686	N	ASP	A	225	4.407	6.376	18.647	1.00	15.00	A	N
ATOM	1687	CA	ASP	A	225	4.478	4.931	18.431	1.00	15.00	A	C
ATOM	1688	CB	ASP	A	225	3.093	4.356	18.121	1.00	15.00	A	C
ATOM	1689	CG	ASP	A	225	2.173	4.327	19.325	1.00	15.00	A	C
ATOM	1690	OD1	ASP	A	225	2.655	4.520	20.457	1.00	15.00	A	O
ATOM	1691	OD2	ASP	A	225	0.960	4.105	19.140	1.00	15.00	A	O
ATOM	1692	C	ASP	A	225	5.442	4.579	17.304	1.00	15.00	A	C
ATOM	1693	O	ASP	A	225	6.266	3.670	17.437	1.00	15.00	A	O

ATOM	1694	N	VAL	A	226	5.343	5.311	16.201	1.00	15.00	A	N
ATOM	1695	CA	VAL	A	226	6.202	5.078	15.046	1.00	15.00	A	C
ATOM	1696	CB	VAL	A	226	5.770	5.928	13.825	1.00	15.00	A	C
ATOM	1697	CG1	VAL	A	226	6.879	6.012	12.785	1.00	15.00	A	C
ATOM	1698	CG2	VAL	A	226	4.510	5.346	13.200	1.00	15.00	A	C
ATOM	1699	C	VAL	A	226	7.671	5.333	15.380	1.00	15.00	A	C
ATOM	1700	O	VAL	A	226	8.529	4.496	15.101	1.00	15.00	A	O
ATOM	1701	N	GLU	A	227	7.952	6.470	16.002	1.00	15.00	A	N
ATOM	1702	CA	GLU	A	227	9.322	6.825	16.354	1.00	15.00	A	C
ATOM	1703	CB	GLU	A	227	9.430	8.303	16.740	1.00	15.00	A	C
ATOM	1704	CG	GLU	A	227	8.812	9.234	15.704	1.00	15.00	A	C
ATOM	1705	CD	GLU	A	227	9.277	10.671	15.814	1.00	15.00	A	C
ATOM	1706	OE1	GLU	A	227	9.136	11.274	16.893	1.00	15.00	A	O
ATOM	1707	OE2	GLU	A	227	9.773	11.206	14.800	1.00	15.00	A	O
ATOM	1708	C	GLU	A	227	9.898	5.905	17.432	1.00	15.00	A	C
ATOM	1709	O	GLU	A	227	11.086	5.592	17.415	1.00	15.00	A	O
ATOM	1710	N	HIS	A	228	9.050	5.456	18.356	1.00	15.00	A	N
ATOM	1711	CA	HIS	A	228	9.491	4.559	19.424	1.00	15.00	A	C
ATOM	1712	CB	HIS	A	228	8.393	4.358	20.475	1.00	15.00	A	C
ATOM	1713	CG	HIS	A	228	8.146	5.535	21.367	1.00	15.00	A	C
ATOM	1714	ND1	HIS	A	228	7.171	5.499	22.335	1.00	15.00	A	N
ATOM	1715	CD2	HIS	A	228	8.746	6.751	21.385	1.00	15.00	A	C
ATOM	1716	CE1	HIS	A	228	7.191	6.683	22.915	1.00	15.00	A	C
ATOM	1717	NE2	HIS	A	228	8.127	7.472	22.374	1.00	15.00	A	N
ATOM	1718	C	HIS	A	228	9.896	3.203	18.862	1.00	15.00	A	C
ATOM	1719	O	HIS	A	228	10.991	2.714	19.128	1.00	15.00	A	O
ATOM	1720	N	THR	A	229	9.009	2.609	18.069	1.00	15.00	A	N
ATOM	1721	CA	THR	A	229	9.266	1.300	17.480	1.00	15.00	A	C
ATOM	1722	CB	THR	A	229	8.023	0.736	16.760	1.00	15.00	A	C
ATOM	1723	OG1	THR	A	229	7.412	1.755	15.953	1.00	15.00	A	O
ATOM	1724	CG2	THR	A	229	7.008	0.218	17.769	1.00	15.00	A	C
ATOM	1725	C	THR	A	229	10.456	1.330	16.521	1.00	15.00	A	C
ATOM	1726	O	THR	A	229	11.158	0.331	16.357	1.00	15.00	A	O
ATOM	1727	N	PHE	A	230	10.686	2.484	15.902	1.00	15.00	A	N
ATOM	1728	CA	PHE	A	230	11.790	2.645	14.966	1.00	15.00	A	C
ATOM	1729	CB	PHE	A	230	11.727	4.015	14.287	1.00	15.00	A	C
ATOM	1730	CG	PHE	A	230	12.733	4.193	13.186	1.00	15.00	A	C
ATOM	1731	CD1	PHE	A	230	12.571	3.547	11.972	1.00	15.00	A	C
ATOM	1732	CD2	PHE	A	230	13.842	5.003	13.367	1.00	15.00	A	C
ATOM	1733	CE1	PHE	A	230	13.496	3.705	10.959	1.00	15.00	A	C
ATOM	1734	CE2	PHE	A	230	14.770	5.166	12.359	1.00	15.00	A	C
ATOM	1735	CZ	PHE	A	230	14.598	4.516	11.152	1.00	15.00	A	C
ATOM	1736	C	PHE	A	230	13.132	2.453	15.669	1.00	15.00	A	C
ATOM	1737	O	PHE	A	230	14.093	1.959	15.072	1.00	15.00	A	O
ATOM	1738	N	GLU	A	231	13.184	2.832	16.942	1.00	15.00	A	N
ATOM	1739	CA	GLU	A	231	14.399	2.698	17.738	1.00	15.00	A	C
ATOM	1740	CB	GLU	A	231	14.204	3.309	19.130	1.00	15.00	A	C
ATOM	1741	CG	GLU	A	231	14.039	4.819	19.136	1.00	15.00	A	C
ATOM	1742	CD	GLU	A	231	15.142	5.523	18.376	1.00	15.00	A	C
ATOM	1743	OE1	GLU	A	231	16.320	5.416	18.777	1.00	15.00	A	O
ATOM	1744	OE2	GLU	A	231	14.848	6.181	17.359	1.00	15.00	A	O
ATOM	1745	C	GLU	A	231	14.797	1.231	17.873	1.00	15.00	A	C
ATOM	1746	O	GLU	A	231	15.980	0.898	17.920	1.00	15.00	A	O
ATOM	1747	N	GLU	A	232	13.798	0.359	17.912	1.00	15.00	A	N
ATOM	1748	CA	GLU	A	232	14.033	-1.072	18.047	1.00	15.00	A	C
ATOM	1749	CB	GLU	A	232	12.780	-1.768	18.584	1.00	15.00	A	C
ATOM	1750	CG	GLU	A	232	12.379	-1.318	19.982	1.00	15.00	A	C
ATOM	1751	CD	GLU	A	232	10.969	-1.734	20.354	1.00	15.00	A	C
ATOM	1752	OE1	GLU	A	232	10.770	-2.913	20.719	1.00	15.00	A	O
ATOM	1753	OE2	GLU	A	232	10.057	-0.883	20.275	1.00	15.00	A	O
ATOM	1754	C	GLU	A	232	14.480	-1.694	16.722	1.00	15.00	A	C
ATOM	1755	O	GLU	A	232	15.140	-2.734	16.702	1.00	15.00	A	O
ATOM	1756	N	ILE	A	233	14.136	-1.043	15.616	1.00	15.00	A	N
ATOM	1757	CA	ILE	A	233	14.509	-1.534	14.292	1.00	15.00	A	C
ATOM	1758	CB	ILE	A	233	13.522	-1.044	13.206	1.00	15.00	A	C
ATOM	1759	CG1	ILE	A	233	12.079	-1.312	13.638	1.00	15.00	A	C
ATOM	1760	CG2	ILE	A	233	13.807	-1.723	11.872	1.00	15.00	A	C
ATOM	1761	CD1	ILE	A	233	11.042	-0.657	12.752	1.00	15.00	A	C
ATOM	1762	C	ILE	A	233	15.925	-1.088	13.928	1.00	15.00	A	C
ATOM	1763	O	ILE	A	233	16.594	-1.712	13.100	1.00	15.00	A	O
ATOM	1764	N	LYS	A	234	16.375	-0.010	14.567	1.00	15.00	A	N

ATOM	1765	CA	LYS	A	234	17.707	0.549	14.325	1.00	15.00	A	C
ATOM	1766	CB	LYS	A	234	18.017	1.703	15.280	1.00	15.00	A	C
ATOM	1767	CG	LYS	A	234	17.539	3.058	14.798	1.00	15.00	A	C
ATOM	1768	CD	LYS	A	234	17.803	4.124	15.843	1.00	15.00	A	C
ATOM	1769	CE	LYS	A	234	17.390	5.497	15.347	1.00	15.00	A	C
ATOM	1770	NZ	LYS	A	234	17.298	6.474	16.461	1.00	15.00	A	N
ATOM	1771	C	LYS	A	234	18.830	-0.494	14.347	1.00	15.00	A	C
ATOM	1772	O	LYS	A	234	19.536	-0.641	13.353	1.00	15.00	A	O
ATOM	1773	N	PRO	A	235	19.006	-1.249	15.459	1.00	15.00	A	N
ATOM	1774	CA	PRO	A	235	20.069	-2.263	15.565	1.00	15.00	A	C
ATOM	1775	CB	PRO	A	235	19.740	-2.987	16.874	1.00	15.00	A	C
ATOM	1776	CG	PRO	A	235	19.006	-1.977	17.681	1.00	15.00	A	C
ATOM	1777	CD	PRO	A	235	18.204	-1.173	16.699	1.00	15.00	A	C
ATOM	1778	C	PRO	A	235	20.057	-3.252	14.400	1.00	15.00	A	C
ATOM	1779	O	PRO	A	235	21.108	-3.697	13.937	1.00	15.00	A	O
ATOM	1780	N	LEU	A	236	18.863	-3.585	13.927	1.00	15.00	A	N
ATOM	1781	CA	LEU	A	236	18.711	-4.515	12.818	1.00	15.00	A	C
ATOM	1782	CB	LEU	A	236	17.262	-5.007	12.740	1.00	15.00	A	C
ATOM	1783	CG	LEU	A	236	16.903	-5.915	11.561	1.00	15.00	A	C
ATOM	1784	CD1	LEU	A	236	17.666	-7.229	11.633	1.00	15.00	A	C
ATOM	1785	CD2	LEU	A	236	15.403	-6.167	11.522	1.00	15.00	A	C
ATOM	1786	C	LEU	A	236	19.119	-3.851	11.505	1.00	15.00	A	C
ATOM	1787	O	LEU	A	236	19.829	-4.440	10.688	1.00	15.00	A	O
ATOM	1788	N	TYR	A	237	18.684	-2.611	11.323	1.00	15.00	A	N
ATOM	1789	CA	TYR	A	237	18.993	-1.862	10.114	1.00	15.00	A	C
ATOM	1790	CB	TYR	A	237	18.140	-0.595	10.024	1.00	15.00	A	C
ATOM	1791	CG	TYR	A	237	18.412	0.233	8.788	1.00	15.00	A	C
ATOM	1792	CD1	TYR	A	237	18.177	-0.283	7.520	1.00	15.00	A	C
ATOM	1793	CD2	TYR	A	237	18.907	1.527	8.889	1.00	15.00	A	C
ATOM	1794	CE1	TYR	A	237	18.426	0.467	6.388	1.00	15.00	A	C
ATOM	1795	CE2	TYR	A	237	19.159	2.285	7.762	1.00	15.00	A	C
ATOM	1796	CZ	TYR	A	237	18.918	1.750	6.514	1.00	15.00	A	C
ATOM	1797	OH	TYR	A	237	19.161	2.500	5.389	1.00	15.00	A	O
ATOM	1798	C	TYR	A	237	20.474	-1.508	10.038	1.00	15.00	A	C
ATOM	1799	O	TYR	A	237	21.088	-1.623	8.978	1.00	15.00	A	O
ATOM	1800	N	GLU	A	238	21.042	-1.087	11.163	1.00	15.00	A	N
ATOM	1801	CA	GLU	A	238	22.452	-0.712	11.224	1.00	15.00	A	C
ATOM	1802	CB	GLU	A	238	22.836	-0.247	12.629	1.00	15.00	A	C
ATOM	1803	CG	GLU	A	238	22.285	1.122	12.996	1.00	15.00	A	C
ATOM	1804	CD	GLU	A	238	22.846	1.645	14.301	1.00	15.00	A	C
ATOM	1805	OE1	GLU	A	238	24.083	1.799	14.395	1.00	15.00	A	O
ATOM	1806	OE2	GLU	A	238	22.050	1.905	15.228	1.00	15.00	A	O
ATOM	1807	C	GLU	A	238	23.355	-1.854	10.773	1.00	15.00	A	C
ATOM	1808	O	GLU	A	238	24.287	-1.648	9.994	1.00	15.00	A	O
ATOM	1809	N	HIS	A	239	23.068	-3.061	11.248	1.00	15.00	A	N
ATOM	1810	CA	HIS	A	239	23.860	-4.226	10.877	1.00	15.00	A	C
ATOM	1811	CB	HIS	A	239	23.567	-5.419	11.785	1.00	15.00	A	C
ATOM	1812	CG	HIS	A	239	24.377	-5.401	13.044	1.00	15.00	A	C
ATOM	1813	ND1	HIS	A	239	25.606	-6.013	13.112	1.00	15.00	A	N
ATOM	1814	CD2	HIS	A	239	24.108	-4.809	14.233	1.00	15.00	A	C
ATOM	1815	CE1	HIS	A	239	26.061	-5.780	14.329	1.00	15.00	A	C
ATOM	1816	NE2	HIS	A	239	25.187	-5.056	15.043	1.00	15.00	A	N
ATOM	1817	C	HIS	A	239	23.665	-4.579	9.408	1.00	15.00	A	C
ATOM	1818	O	HIS	A	239	24.602	-5.007	8.731	1.00	15.00	A	O
ATOM	1819	N	LEU	A	240	22.448	-4.389	8.915	1.00	15.00	A	N
ATOM	1820	CA	LEU	A	240	22.148	-4.658	7.517	1.00	15.00	A	C
ATOM	1821	CB	LEU	A	240	20.642	-4.550	7.262	1.00	15.00	A	C
ATOM	1822	CG	LEU	A	240	20.176	-4.810	5.827	1.00	15.00	A	C
ATOM	1823	CD1	LEU	A	240	20.359	-6.274	5.454	1.00	15.00	A	C
ATOM	1824	CD2	LEU	A	240	18.727	-4.383	5.647	1.00	15.00	A	C
ATOM	1825	C	LEU	A	240	22.903	-3.660	6.645	1.00	15.00	A	C
ATOM	1826	O	LEU	A	240	23.544	-4.034	5.664	1.00	15.00	A	O
ATOM	1827	N	HIS	A	241	22.838	-2.391	7.041	1.00	15.00	A	N
ATOM	1828	CA	HIS	A	241	23.510	-1.307	6.332	1.00	15.00	A	C
ATOM	1829	CB	HIS	A	241	23.251	0.016	7.068	1.00	15.00	A	C
ATOM	1830	CG	HIS	A	241	23.725	1.251	6.358	1.00	15.00	A	C
ATOM	1831	ND1	HIS	A	241	25.037	1.663	6.426	1.00	15.00	A	N
ATOM	1832	CD2	HIS	A	241	23.019	2.139	5.616	1.00	15.00	A	C
ATOM	1833	CE1	HIS	A	241	25.100	2.785	5.734	1.00	15.00	A	C
ATOM	1834	NE2	HIS	A	241	23.905	3.110	5.226	1.00	15.00	A	N
ATOM	1835	C	HIS	A	241	25.010	-1.575	6.263	1.00	15.00	A	C

ATOM	1836	O	HIS	A	241	25.628	-1.447	5.205	1.00	15.00	A	O
ATOM	1837	N	ALA	A	242	25.578	-1.974	7.399	1.00	15.00	A	N
ATOM	1838	CA	ALA	A	242	27.004	-2.262	7.491	1.00	15.00	A	C
ATOM	1839	CB	ALA	A	242	27.391	-2.583	8.925	1.00	15.00	A	C
ATOM	1840	C	ALA	A	242	27.413	-3.391	6.550	1.00	15.00	A	C
ATOM	1841	O	ALA	A	242	28.411	-3.279	5.833	1.00	15.00	A	O
ATOM	1842	N	TYR	A	243	26.628	-4.465	6.537	1.00	15.00	A	N
ATOM	1843	CA	TYR	A	243	26.921	-5.603	5.681	1.00	15.00	A	C
ATOM	1844	CB	TYR	A	243	26.005	-6.791	5.995	1.00	15.00	A	C
ATOM	1845	CG	TYR	A	243	26.268	-8.004	5.127	1.00	15.00	A	C
ATOM	1846	CD1	TYR	A	243	27.367	-8.824	5.355	1.00	15.00	A	C
ATOM	1847	CD2	TYR	A	243	25.422	-8.322	4.073	1.00	15.00	A	C
ATOM	1848	CE1	TYR	A	243	27.614	-9.925	4.556	1.00	15.00	A	C
ATOM	1849	CE2	TYR	A	243	25.660	-9.421	3.271	1.00	15.00	A	C
ATOM	1850	CZ	TYR	A	243	26.757	-10.219	3.515	1.00	15.00	A	C
ATOM	1851	OH	TYR	A	243	27.004	-11.314	2.713	1.00	15.00	A	O
ATOM	1852	C	TYR	A	243	26.813	-5.207	4.213	1.00	15.00	A	C
ATOM	1853	O	TYR	A	243	27.672	-5.559	3.401	1.00	15.00	A	O
ATOM	1854	N	VAL	A	244	25.755	-4.476	3.875	1.00	15.00	A	N
ATOM	1855	CA	VAL	A	244	25.563	-4.017	2.505	1.00	15.00	A	C
ATOM	1856	CB	VAL	A	244	24.221	-3.265	2.320	1.00	15.00	A	C
ATOM	1857	CG1	VAL	A	244	24.127	-2.664	0.927	1.00	15.00	A	C
ATOM	1858	CG2	VAL	A	244	23.052	-4.208	2.550	1.00	15.00	A	C
ATOM	1859	C	VAL	A	244	26.735	-3.124	2.098	1.00	15.00	A	C
ATOM	1860	O	VAL	A	244	27.257	-3.234	0.986	1.00	15.00	A	O
ATOM	1861	N	ARG	A	245	27.156	-2.262	3.020	1.00	15.00	A	N
ATOM	1862	CA	ARG	A	245	28.275	-1.373	2.778	1.00	15.00	A	C
ATOM	1863	CB	ARG	A	245	28.493	-0.438	3.967	1.00	15.00	A	C
ATOM	1864	CG	ARG	A	245	29.831	0.283	3.947	1.00	15.00	A	C
ATOM	1865	CD	ARG	A	245	30.223	0.761	5.330	1.00	15.00	A	C
ATOM	1866	NE	ARG	A	245	29.593	2.035	5.701	1.00	15.00	A	N
ATOM	1867	CZ	ARG	A	245	29.202	2.306	6.943	1.00	15.00	A	C
ATOM	1868	NH1	ARG	A	245	29.385	1.401	7.891	1.00	15.00	A	N
ATOM	1869	NH2	ARG	A	245	28.640	3.465	7.241	1.00	15.00	A	N
ATOM	1870	C	ARG	A	245	29.544	-2.165	2.479	1.00	15.00	A	C
ATOM	1871	O	ARG	A	245	30.259	-1.852	1.538	1.00	15.00	A	O
ATOM	1872	N	ALA	A	246	29.808	-3.193	3.278	1.00	15.00	A	N
ATOM	1873	CA	ALA	A	246	30.990	-4.031	3.085	1.00	15.00	A	C
ATOM	1874	CB	ALA	A	246	31.131	-5.036	4.219	1.00	15.00	A	C
ATOM	1875	C	ALA	A	246	30.962	-4.751	1.734	1.00	15.00	A	C
ATOM	1876	O	ALA	A	246	31.979	-4.843	1.047	1.00	15.00	A	O
ATOM	1877	N	LYS	A	247	29.793	-5.251	1.352	1.00	15.00	A	N
ATOM	1878	CA	LYS	A	247	29.645	-5.963	0.086	1.00	15.00	A	C
ATOM	1879	CB	LYS	A	247	28.299	-6.689	0.016	1.00	15.00	A	C
ATOM	1880	CG	LYS	A	247	28.101	-7.750	1.091	1.00	15.00	A	C
ATOM	1881	CD	LYS	A	247	28.860	-9.034	0.780	1.00	15.00	A	C
ATOM	1882	CE	LYS	A	247	28.121	-9.883	-0.243	1.00	15.00	A	C
ATOM	1883	NZ	LYS	A	247	28.483	-11.322	-0.151	1.00	15.00	A	N
ATOM	1884	C	LYS	A	247	29.808	-5.022	-1.104	1.00	15.00	A	C
ATOM	1885	O	LYS	A	247	30.494	-5.349	-2.071	1.00	15.00	A	O
ATOM	1886	N	LEU	A	248	29.180	-3.856	-1.024	1.00	15.00	A	N
ATOM	1887	CA	LEU	A	248	29.259	-2.871	-2.098	1.00	15.00	A	C
ATOM	1888	CB	LEU	A	248	28.184	-1.794	-1.928	1.00	15.00	A	C
ATOM	1889	CG	LEU	A	248	26.742	-2.218	-2.205	1.00	15.00	A	C
ATOM	1890	CD1	LEU	A	248	25.796	-1.043	-2.010	1.00	15.00	A	C
ATOM	1891	CD2	LEU	A	248	26.607	-2.785	-3.611	1.00	15.00	A	C
ATOM	1892	C	LEU	A	248	30.643	-2.228	-2.167	1.00	15.00	A	C
ATOM	1893	O	LEU	A	248	31.124	-1.889	-3.248	1.00	15.00	A	O
ATOM	1894	N	MET	A	249	31.281	-2.073	-1.011	1.00	15.00	A	N
ATOM	1895	CA	MET	A	249	32.612	-1.471	-0.932	1.00	15.00	A	C
ATOM	1896	CB	MET	A	249	33.040	-1.311	0.528	1.00	15.00	A	C
ATOM	1897	CG	MET	A	249	34.291	-0.471	0.735	1.00	15.00	A	C
ATOM	1898	SD	MET	A	249	34.843	-0.469	2.452	1.00	15.00	A	S
ATOM	1899	CE	MET	A	249	35.329	-2.182	2.654	1.00	15.00	A	C
ATOM	1900	C	MET	A	249	33.639	-2.309	-1.685	1.00	15.00	A	C
ATOM	1901	O	MET	A	249	34.636	-1.789	-2.179	1.00	15.00	A	O
ATOM	1902	N	ASN	A	250	33.385	-3.607	-1.776	1.00	15.00	A	N
ATOM	1903	CA	ASN	A	250	34.289	-4.512	-2.475	1.00	15.00	A	C
ATOM	1904	CB	ASN	A	250	34.048	-5.961	-2.046	1.00	15.00	A	C
ATOM	1905	CG	ASN	A	250	34.659	-6.280	-0.694	1.00	15.00	A	C
ATOM	1906	OD1	ASN	A	250	35.722	-5.774	-0.342	1.00	15.00	A	O

ATOM	1907	ND2	ASN	A	250	33.986	-7.118	0.078	1.00	15.00	A	N
ATOM	1908	C	ASN	A	250	34.140	-4.368	-3.985	1.00	15.00	A	C
ATOM	1909	O	ASN	A	250	35.036	-4.727	-4.747	1.00	15.00	A	O
ATOM	1910	N	ALA	A	251	33.001	-3.833	-4.411	1.00	15.00	A	N
ATOM	1911	CA	ALA	A	251	32.732	-3.630	-5.828	1.00	15.00	A	C
ATOM	1912	CB	ALA	A	251	31.265	-3.894	-6.134	1.00	15.00	A	C
ATOM	1913	C	ALA	A	251	33.122	-2.215	-6.243	1.00	15.00	A	C
ATOM	1914	O	ALA	A	251	33.600	-1.987	-7.353	1.00	15.00	A	O
ATOM	1915	N	TYR	A	252	32.915	-1.267	-5.337	1.00	15.00	A	N
ATOM	1916	CA	TYR	A	252	33.251	0.130	-5.587	1.00	15.00	A	C
ATOM	1917	CB	TYR	A	252	31.979	0.986	-5.657	1.00	15.00	A	C
ATOM	1918	CG	TYR	A	252	30.909	0.474	-6.597	1.00	15.00	A	C
ATOM	1919	CD1	TYR	A	252	30.974	0.722	-7.962	1.00	15.00	A	C
ATOM	1920	CD2	TYR	A	252	29.824	-0.249	-6.114	1.00	15.00	A	C
ATOM	1921	CE1	TYR	A	252	29.990	0.266	-8.819	1.00	15.00	A	C
ATOM	1922	CE2	TYR	A	252	28.838	-0.711	-6.964	1.00	15.00	A	C
ATOM	1923	CZ	TYR	A	252	28.924	-0.450	-8.315	1.00	15.00	A	C
ATOM	1924	OH	TYR	A	252	27.940	-0.905	-9.163	1.00	15.00	A	O
ATOM	1925	C	TYR	A	252	34.150	0.659	-4.469	1.00	15.00	A	C
ATOM	1926	O	TYR	A	252	33.733	1.512	-3.683	1.00	15.00	A	O
ATOM	1927	N	PRO	A	253	35.406	0.180	-4.391	1.00	15.00	A	N
ATOM	1928	CA	PRO	A	253	36.346	0.590	-3.336	1.00	15.00	A	C
ATOM	1929	CB	PRO	A	253	37.578	-0.287	-3.588	1.00	15.00	A	C
ATOM	1930	CG	PRO	A	253	37.483	-0.673	-5.023	1.00	15.00	A	C
ATOM	1931	CD	PRO	A	253	36.017	-0.786	-5.324	1.00	15.00	A	C
ATOM	1932	C	PRO	A	253	36.722	2.065	-3.415	1.00	15.00	A	C
ATOM	1933	O	PRO	A	253	37.114	2.670	-2.420	1.00	15.00	A	O
ATOM	1934	N	SER	A	254	36.595	2.642	-4.596	1.00	15.00	A	N
ATOM	1935	CA	SER	A	254	36.934	4.037	-4.799	1.00	15.00	A	C
ATOM	1936	CB	SER	A	254	37.690	4.176	-6.120	1.00	15.00	A	C
ATOM	1937	OG	SER	A	254	37.325	3.133	-7.015	1.00	15.00	A	O
ATOM	1938	C	SER	A	254	35.693	4.924	-4.809	1.00	15.00	A	C
ATOM	1939	O	SER	A	254	35.763	6.089	-5.192	1.00	15.00	A	O
ATOM	1940	N	TYR	A	255	34.555	4.380	-4.387	1.00	15.00	A	N
ATOM	1941	CA	TYR	A	255	33.317	5.150	-4.385	1.00	15.00	A	C
ATOM	1942	CB	TYR	A	255	32.424	4.729	-5.556	1.00	15.00	A	C
ATOM	1943	CG	TYR	A	255	33.040	4.958	-6.919	1.00	15.00	A	C
ATOM	1944	CD1	TYR	A	255	33.359	6.238	-7.354	1.00	15.00	A	C
ATOM	1945	CD2	TYR	A	255	33.299	3.893	-7.774	1.00	15.00	A	C
ATOM	1946	CE1	TYR	A	255	33.918	6.452	-8.598	1.00	15.00	A	C
ATOM	1947	CE2	TYR	A	255	33.860	4.097	-9.020	1.00	15.00	A	C
ATOM	1948	CZ	TYR	A	255	34.166	5.379	-9.427	1.00	15.00	A	C
ATOM	1949	OH	TYR	A	255	34.722	5.589	-10.667	1.00	15.00	A	O
ATOM	1950	C	TYR	A	255	32.540	5.036	-3.075	1.00	15.00	A	C
ATOM	1951	O	TYR	A	255	31.578	5.774	-2.860	1.00	15.00	A	O
ATOM	1952	N	ILE	A	256	32.943	4.120	-2.204	1.00	15.00	A	N
ATOM	1953	CA	ILE	A	256	32.246	3.933	-0.936	1.00	15.00	A	C
ATOM	1954	CB	ILE	A	256	31.414	2.629	-0.931	1.00	15.00	A	C
ATOM	1955	CG1	ILE	A	256	30.423	2.633	-2.100	1.00	15.00	A	C
ATOM	1956	CG2	ILE	A	256	30.674	2.458	0.390	1.00	15.00	A	C
ATOM	1957	CD1	ILE	A	256	29.647	1.348	-2.259	1.00	15.00	A	C
ATOM	1958	C	ILE	A	256	33.213	3.939	0.243	1.00	15.00	A	C
ATOM	1959	O	ILE	A	256	34.296	3.362	0.176	1.00	15.00	A	O
ATOM	1960	N	SER	A	257	32.811	4.606	1.316	1.00	15.00	A	N
ATOM	1961	CA	SER	A	257	33.618	4.691	2.518	1.00	15.00	A	C
ATOM	1962	CB	SER	A	257	33.480	6.087	3.139	1.00	15.00	A	C
ATOM	1963	OG	SER	A	257	34.086	6.157	4.420	1.00	15.00	A	O
ATOM	1964	C	SER	A	257	33.176	3.625	3.515	1.00	15.00	A	C
ATOM	1965	O	SER	A	257	31.978	3.396	3.694	1.00	15.00	A	O
ATOM	1966	N	PRO	A	258	34.137	2.961	4.179	1.00	15.00	A	N
ATOM	1967	CA	PRO	A	258	33.847	1.912	5.166	1.00	15.00	A	C
ATOM	1968	CB	PRO	A	258	35.233	1.375	5.540	1.00	15.00	A	C
ATOM	1969	CG	PRO	A	258	36.169	2.486	5.212	1.00	15.00	A	C
ATOM	1970	CD	PRO	A	258	35.586	3.169	4.009	1.00	15.00	A	C
ATOM	1971	C	PRO	A	258	33.143	2.459	6.408	1.00	15.00	A	C
ATOM	1972	O	PRO	A	258	32.624	1.697	7.224	1.00	15.00	A	O
ATOM	1973	N	ILE	A	259	33.136	3.780	6.548	1.00	15.00	A	N
ATOM	1974	CA	ILE	A	259	32.493	4.425	7.688	1.00	15.00	A	C
ATOM	1975	CB	ILE	A	259	33.521	5.123	8.609	1.00	15.00	A	C
ATOM	1976	CG1	ILE	A	259	34.252	6.244	7.862	1.00	15.00	A	C
ATOM	1977	CG2	ILE	A	259	34.516	4.115	9.168	1.00	15.00	A	C

ATOM	1978	CD1	ILE	A	259	35.038	7.172	8.764	1.00	15.00	A	C
ATOM	1979	C	ILE	A	259	31.451	5.445	7.229	1.00	15.00	A	C
ATOM	1980	O	ILE	A	259	30.598	5.871	8.007	1.00	15.00	A	O
ATOM	1981	N	GLY	A	260	31.513	5.817	5.956	1.00	15.00	A	N
ATOM	1982	CA	GLY	A	260	30.586	6.795	5.420	1.00	15.00	A	C
ATOM	1983	C	GLY	A	260	29.252	6.198	5.017	1.00	15.00	A	C
ATOM	1984	O	GLY	A	260	29.075	4.978	5.028	1.00	15.00	A	O
ATOM	1985	N	CYS	A	261	28.310	7.065	4.672	1.00	15.00	A	N
ATOM	1986	CA	CYS	A	261	26.981	6.638	4.259	1.00	15.00	A	C
ATOM	1987	CB	CYS	A	261	26.039	7.841	4.224	1.00	15.00	A	C
ATOM	1988	SG	CYS	A	261	25.878	8.701	5.803	1.00	15.00	A	S
ATOM	1989	C	CYS	A	261	27.017	5.957	2.891	1.00	15.00	A	C
ATOM	1990	O	CYS	A	261	28.050	5.941	2.219	1.00	15.00	A	O
ATOM	1991	N	LEU	A	262	25.886	5.399	2.482	1.00	15.00	A	N
ATOM	1992	CA	LEU	A	262	25.791	4.716	1.200	1.00	15.00	A	C
ATOM	1993	CB	LEU	A	262	24.882	3.489	1.309	1.00	15.00	A	C
ATOM	1994	CG	LEU	A	262	25.453	2.288	2.066	1.00	15.00	A	C
ATOM	1995	CD1	LEU	A	262	24.440	1.153	2.109	1.00	15.00	A	C
ATOM	1996	CD2	LEU	A	262	26.753	1.823	1.429	1.00	15.00	A	C
ATOM	1997	C	LEU	A	262	25.269	5.657	0.121	1.00	15.00	A	C
ATOM	1998	O	LEU	A	262	24.403	6.491	0.387	1.00	15.00	A	O
ATOM	1999	N	PRO	A	263	25.805	5.546	-1.106	1.00	15.00	A	N
ATOM	2000	CA	PRO	A	263	25.385	6.381	-2.238	1.00	15.00	A	C
ATOM	2001	CB	PRO	A	263	26.261	5.891	-3.397	1.00	15.00	A	C
ATOM	2002	CG	PRO	A	263	27.418	5.221	-2.741	1.00	15.00	A	C
ATOM	2003	CD	PRO	A	263	26.875	4.610	-1.484	1.00	15.00	A	C
ATOM	2004	C	PRO	A	263	23.909	6.178	-2.574	1.00	15.00	A	C
ATOM	2005	O	PRO	A	263	23.448	5.048	-2.746	1.00	15.00	A	O
ATOM	2006	N	ALA	A	264	23.181	7.283	-2.676	1.00	15.00	A	N
ATOM	2007	CA	ALA	A	264	21.751	7.256	-2.971	1.00	15.00	A	C
ATOM	2008	CB	ALA	A	264	21.187	8.668	-2.955	1.00	15.00	A	C
ATOM	2009	C	ALA	A	264	21.420	6.565	-4.294	1.00	15.00	A	C
ATOM	2010	O	ALA	A	264	20.351	5.974	-4.439	1.00	15.00	A	O
ATOM	2011	N	HIS	A	265	22.334	6.631	-5.252	1.00	15.00	A	N
ATOM	2012	CA	HIS	A	265	22.110	6.021	-6.559	1.00	15.00	A	C
ATOM	2013	CB	HIS	A	265	22.795	6.826	-7.671	1.00	15.00	A	C
ATOM	2014	CG	HIS	A	265	24.290	6.922	-7.565	1.00	15.00	A	C
ATOM	2015	ND1	HIS	A	265	24.903	7.422	-6.439	1.00	15.00	A	N
ATOM	2016	CD2	HIS	A	265	25.236	6.590	-8.477	1.00	15.00	A	C
ATOM	2017	CE1	HIS	A	265	26.200	7.387	-6.690	1.00	15.00	A	C
ATOM	2018	NE2	HIS	A	265	26.445	6.891	-7.910	1.00	15.00	A	N
ATOM	2019	C	HIS	A	265	22.539	4.555	-6.602	1.00	15.00	A	C
ATOM	2020	O	HIS	A	265	22.395	3.889	-7.627	1.00	15.00	A	O
ATOM	2021	N	LEU	A	266	23.038	4.047	-5.486	1.00	15.00	A	N
ATOM	2022	CA	LEU	A	266	23.493	2.663	-5.416	1.00	15.00	A	C
ATOM	2023	CB	LEU	A	266	24.994	2.604	-5.111	1.00	15.00	A	C
ATOM	2024	CG	LEU	A	266	25.933	3.122	-6.204	1.00	15.00	A	C
ATOM	2025	CD1	LEU	A	266	27.385	2.964	-5.780	1.00	15.00	A	C
ATOM	2026	CD2	LEU	A	266	25.682	2.399	-7.519	1.00	15.00	A	C
ATOM	2027	C	LEU	A	266	22.716	1.881	-4.365	1.00	15.00	A	C
ATOM	2028	O	LEU	A	266	23.216	0.909	-3.804	1.00	15.00	A	O
ATOM	2029	N	LEU	A	267	21.484	2.304	-4.111	1.00	15.00	A	N
ATOM	2030	CA	LEU	A	267	20.646	1.647	-3.115	1.00	15.00	A	C
ATOM	2031	CB	LEU	A	267	19.741	2.662	-2.420	1.00	15.00	A	C
ATOM	2032	CG	LEU	A	267	20.451	3.770	-1.641	1.00	15.00	A	C
ATOM	2033	CD1	LEU	A	267	19.442	4.764	-1.096	1.00	15.00	A	C
ATOM	2034	CD2	LEU	A	267	21.292	3.184	-0.517	1.00	15.00	A	C
ATOM	2035	C	LEU	A	267	19.817	0.515	-3.711	1.00	15.00	A	C
ATOM	2036	O	LEU	A	267	19.381	-0.383	-2.999	1.00	15.00	A	O
ATOM	2037	N	GLY	A	268	19.593	0.556	-5.014	1.00	15.00	A	N
ATOM	2038	CA	GLY	A	268	18.816	-0.491	-5.646	1.00	15.00	A	C
ATOM	2039	C	GLY	A	268	17.785	0.049	-6.609	1.00	15.00	A	C
ATOM	2040	O	GLY	A	268	17.456	-0.603	-7.597	1.00	15.00	A	O
ATOM	2041	N	ASP	A	269	17.269	1.235	-6.326	1.00	15.00	A	N
ATOM	2042	CA	ASP	A	269	16.276	1.854	-7.195	1.00	15.00	A	C
ATOM	2043	CB	ASP	A	269	14.832	1.656	-6.699	1.00	15.00	A	C
ATOM	2044	CG	ASP	A	269	14.537	2.293	-5.357	1.00	15.00	A	C
ATOM	2045	OD1	ASP	A	269	14.195	3.493	-5.324	1.00	15.00	A	O
ATOM	2046	OD2	ASP	A	269	14.632	1.594	-4.331	1.00	15.00	A	O
ATOM	2047	C	ASP	A	269	16.610	3.316	-7.488	1.00	15.00	A	C
ATOM	2048	O	ASP	A	269	17.774	3.715	-7.416	1.00	15.00	A	O

ATOM	2049	N	MET	A	270	15.593	4.104	-7.813	1.00	15.00	A	N
ATOM	2050	CA	MET	A	270	15.779	5.510	-8.157	1.00	15.00	A	C
ATOM	2051	CB	MET	A	270	14.732	5.930	-9.199	1.00	15.00	A	C
ATOM	2052	CG	MET	A	270	14.677	7.423	-9.486	1.00	15.00	A	C
ATOM	2053	SD	MET	A	270	16.129	8.024	-10.370	1.00	15.00	A	S
ATOM	2054	CE	MET	A	270	15.727	7.543	-12.047	1.00	15.00	A	C
ATOM	2055	C	MET	A	270	15.719	6.424	-6.931	1.00	15.00	A	C
ATOM	2056	O	MET	A	270	16.247	7.542	-6.948	1.00	15.00	A	O
ATOM	2057	N	TRP	A	271	15.084	5.960	-5.867	1.00	15.00	A	N
ATOM	2058	CA	TRP	A	271	14.963	6.762	-4.655	1.00	15.00	A	C
ATOM	2059	CB	TRP	A	271	13.486	6.991	-4.303	1.00	15.00	A	C
ATOM	2060	CG	TRP	A	271	12.652	7.502	-5.442	1.00	15.00	A	C
ATOM	2061	CD1	TRP	A	271	12.427	8.806	-5.777	1.00	15.00	A	C
ATOM	2062	CD2	TRP	A	271	11.925	6.714	-6.394	1.00	15.00	A	C
ATOM	2063	NE1	TRP	A	271	11.607	8.876	-6.878	1.00	15.00	A	N
ATOM	2064	CE2	TRP	A	271	11.286	7.607	-7.276	1.00	15.00	A	C
ATOM	2065	CE3	TRP	A	271	11.755	5.340	-6.587	1.00	15.00	A	C
ATOM	2066	CZ2	TRP	A	271	10.491	7.172	-8.332	1.00	15.00	A	C
ATOM	2067	CZ3	TRP	A	271	10.965	4.910	-7.635	1.00	15.00	A	C
ATOM	2068	CH2	TRP	A	271	10.344	5.822	-8.495	1.00	15.00	A	C
ATOM	2069	C	TRP	A	271	15.673	6.090	-3.483	1.00	15.00	A	C
ATOM	2070	O	TRP	A	271	16.136	6.758	-2.548	1.00	15.00	A	O
ATOM	2071	N	GLY	A	272	15.760	4.771	-3.544	1.00	15.00	A	N
ATOM	2072	CA	GLY	A	272	16.387	4.014	-2.485	1.00	15.00	A	C
ATOM	2073	C	GLY	A	272	15.344	3.538	-1.502	1.00	15.00	A	C
ATOM	2074	O	GLY	A	272	15.457	3.771	-0.301	1.00	15.00	A	O
ATOM	2075	N	ARG	A	273	14.326	2.870	-2.026	1.00	15.00	A	N
ATOM	2076	CA	ARG	A	273	13.226	2.372	-1.225	1.00	15.00	A	C
ATOM	2077	CB	ARG	A	273	11.895	2.799	-1.860	1.00	15.00	A	C
ATOM	2078	CG	ARG	A	273	10.764	3.029	-0.868	1.00	15.00	A	C
ATOM	2079	CD	ARG	A	273	9.655	1.999	-0.996	1.00	15.00	A	C
ATOM	2080	NE	ARG	A	273	8.820	2.191	-2.181	1.00	15.00	A	N
ATOM	2081	CZ	ARG	A	273	7.509	1.970	-2.195	1.00	15.00	A	C
ATOM	2082	NH1	ARG	A	273	6.893	1.577	-1.084	1.00	15.00	A	N
ATOM	2083	NH2	ARG	A	273	6.821	2.098	-3.322	1.00	15.00	A	N
ATOM	2084	C	ARG	A	273	13.282	0.853	-1.114	1.00	15.00	A	C
ATOM	2085	O	ARG	A	273	12.726	0.274	-0.181	1.00	15.00	A	O
ATOM	2086	N	PHE	A	274	13.910	0.209	-2.092	1.00	15.00	A	N
ATOM	2087	CA	PHE	A	274	14.051	-1.243	-2.088	1.00	15.00	A	C
ATOM	2088	CB	PHE	A	274	13.034	-1.927	-3.009	1.00	15.00	A	C
ATOM	2089	CG	PHE	A	274	11.613	-1.835	-2.528	1.00	15.00	A	C
ATOM	2090	CD1	PHE	A	274	11.262	-2.282	-1.264	1.00	15.00	A	C
ATOM	2091	CD2	PHE	A	274	10.629	-1.296	-3.339	1.00	15.00	A	C
ATOM	2092	CE1	PHE	A	274	9.956	-2.191	-0.818	1.00	15.00	A	C
ATOM	2093	CE2	PHE	A	274	9.321	-1.204	-2.900	1.00	15.00	A	C
ATOM	2094	CZ	PHE	A	274	8.984	-1.651	-1.638	1.00	15.00	A	C
ATOM	2095	C	PHE	A	274	15.468	-1.648	-2.471	1.00	15.00	A	C
ATOM	2096	O	PHE	A	274	15.929	-1.379	-3.580	1.00	15.00	A	O
ATOM	2097	N	TRP	A	275	16.154	-2.305	-1.548	1.00	15.00	A	N
ATOM	2098	CA	TRP	A	275	17.523	-2.747	-1.777	1.00	15.00	A	C
ATOM	2099	CB	TRP	A	275	18.312	-2.747	-0.461	1.00	15.00	A	C
ATOM	2100	CG	TRP	A	275	18.526	-1.394	0.156	1.00	15.00	A	C
ATOM	2101	CD1	TRP	A	275	18.005	-0.197	-0.251	1.00	15.00	A	C
ATOM	2102	CD2	TRP	A	275	19.329	-1.106	1.307	1.00	15.00	A	C
ATOM	2103	NE1	TRP	A	275	18.438	0.814	0.575	1.00	15.00	A	N
ATOM	2104	CE2	TRP	A	275	19.251	0.280	1.539	1.00	15.00	A	C
ATOM	2105	CE3	TRP	A	275	20.107	-1.890	2.164	1.00	15.00	A	C
ATOM	2106	CZ2	TRP	A	275	19.922	0.898	2.592	1.00	15.00	A	C
ATOM	2107	CZ3	TRP	A	275	20.771	-1.275	3.208	1.00	15.00	A	C
ATOM	2108	CH2	TRP	A	275	20.675	0.105	3.414	1.00	15.00	A	C
ATOM	2109	C	TRP	A	275	17.534	-4.152	-2.368	1.00	15.00	A	C
ATOM	2110	O	TRP	A	275	18.578	-4.795	-2.447	1.00	15.00	A	O
ATOM	2111	N	THR	A	276	16.363	-4.623	-2.783	1.00	15.00	A	N
ATOM	2112	CA	THR	A	276	16.223	-5.954	-3.361	1.00	15.00	A	C
ATOM	2113	CB	THR	A	276	14.761	-6.225	-3.757	1.00	15.00	A	C
ATOM	2114	OG1	THR	A	276	13.891	-5.548	-2.837	1.00	15.00	A	O
ATOM	2115	CG2	THR	A	276	14.467	-7.719	-3.725	1.00	15.00	A	C
ATOM	2116	C	THR	A	276	17.118	-6.154	-4.585	1.00	15.00	A	C
ATOM	2117	O	THR	A	276	17.577	-7.266	-4.850	1.00	15.00	A	O
ATOM	2118	N	ASN	A	277	17.391	-5.073	-5.308	1.00	15.00	A	N
ATOM	2119	CA	ASN	A	277	18.221	-5.153	-6.508	1.00	15.00	A	C

```
ATOM   2120  CB  ASN A 277      17.958  -3.976  -7.448  1.00 15.00      A    C
ATOM   2121  CG  ASN A 277      16.586  -4.040  -8.090  1.00 15.00      A    C
ATOM   2122  OD1 ASN A 277      16.024  -5.117  -8.283  1.00 15.00      A    O
ATOM   2123  ND2 ASN A 277      16.035  -2.884  -8.422  1.00 15.00      A    N
ATOM   2124  C   ASN A 277      19.701  -5.245  -6.166  1.00 15.00      A    C
ATOM   2125  O   ASN A 277      20.544  -5.415  -7.045  1.00 15.00      A    O
ATOM   2126  N   LEU A 278      20.015  -5.136  -4.884  1.00 15.00      A    N
ATOM   2127  CA  LEU A 278      21.393  -5.221  -4.432  1.00 15.00      A    C
ATOM   2128  CB  LEU A 278      21.637  -4.302  -3.233  1.00 15.00      A    C
ATOM   2129  CG  LEU A 278      21.637  -2.798  -3.508  1.00 15.00      A    C
ATOM   2130  CD1 LEU A 278      22.046  -2.037  -2.258  1.00 15.00      A    C
ATOM   2131  CD2 LEU A 278      22.560  -2.460  -4.670  1.00 15.00      A    C
ATOM   2132  C   LEU A 278      21.738  -6.651  -4.051  1.00 15.00      A    C
ATOM   2133  O   LEU A 278      22.889  -6.954  -3.745  1.00 15.00      A    O
ATOM   2134  N   TYR A 279      20.734  -7.527  -4.084  1.00 15.00      A    N
ATOM   2135  CA  TYR A 279      20.911  -8.936  -3.726  1.00 15.00      A    C
ATOM   2136  CB  TYR A 279      19.586  -9.693  -3.876  1.00 15.00      A    C
ATOM   2137  CG  TYR A 279      19.655 -11.166  -3.530  1.00 15.00      A    C
ATOM   2138  CD1 TYR A 279      19.742 -11.590  -2.209  1.00 15.00      A    C
ATOM   2139  CD2 TYR A 279      19.626 -12.134  -4.528  1.00 15.00      A    C
ATOM   2140  CE1 TYR A 279      19.798 -12.936  -1.893  1.00 15.00      A    C
ATOM   2141  CE2 TYR A 279      19.683 -13.480  -4.221  1.00 15.00      A    C
ATOM   2142  CZ  TYR A 279      19.768 -13.876  -2.903  1.00 15.00      A    C
ATOM   2143  OH  TYR A 279      19.820 -15.218  -2.596  1.00 15.00      A    O
ATOM   2144  C   TYR A 279      22.000  -9.599  -4.566  1.00 15.00      A    C
ATOM   2145  O   TYR A 279      22.656 -10.538  -4.120  1.00 15.00      A    O
ATOM   2146  N   SER A 280      22.199  -9.089  -5.769  1.00 15.00      A    N
ATOM   2147  CA  SER A 280      23.200  -9.620  -6.678  1.00 15.00      A    C
ATOM   2148  CB  SER A 280      23.051  -8.912  -8.022  1.00 15.00      A    C
ATOM   2149  OG  SER A 280      22.202  -7.781  -7.880  1.00 15.00      A    O
ATOM   2150  C   SER A 280      24.612  -9.412  -6.134  1.00 15.00      A    C
ATOM   2151  O   SER A 280      25.521 -10.185  -6.430  1.00 15.00      A    O
ATOM   2152  N   LEU A 281      24.786  -8.376  -5.322  1.00 15.00      A    N
ATOM   2153  CA  LEU A 281      26.092  -8.065  -4.761  1.00 15.00      A    C
ATOM   2154  CB  LEU A 281      26.489  -6.625  -5.102  1.00 15.00      A    C
ATOM   2155  CG  LEU A 281      26.568  -6.282  -6.593  1.00 15.00      A    C
ATOM   2156  CD1 LEU A 281      26.838  -4.798  -6.791  1.00 15.00      A    C
ATOM   2157  CD2 LEU A 281      27.636  -7.118  -7.282  1.00 15.00      A    C
ATOM   2158  C   LEU A 281      26.145  -8.285  -3.251  1.00 15.00      A    C
ATOM   2159  O   LEU A 281      27.223  -8.445  -2.685  1.00 15.00      A    O
ATOM   2160  N   THR A 282      24.988  -8.299  -2.600  1.00 15.00      A    N
ATOM   2161  CA  THR A 282      24.934  -8.485  -1.153  1.00 15.00      A    C
ATOM   2162  CB  THR A 282      23.872  -7.579  -0.500  1.00 15.00      A    C
ATOM   2163  OG1 THR A 282      22.642  -7.684  -1.223  1.00 15.00      A    O
ATOM   2164  CG2 THR A 282      24.334  -6.130  -0.508  1.00 15.00      A    C
ATOM   2165  C   THR A 282      24.675  -9.939  -0.765  1.00 15.00      A    C
ATOM   2166  O   THR A 282      24.643 -10.273   0.418  1.00 15.00      A    O
ATOM   2167  N   VAL A 283      24.505 -10.797  -1.765  1.00 15.00      A    N
ATOM   2168  CA  VAL A 283      24.247 -12.215  -1.532  1.00 15.00      A    C
ATOM   2169  CB  VAL A 283      24.047 -12.989  -2.858  1.00 15.00      A    C
ATOM   2170  CG1 VAL A 283      25.259 -12.852  -3.773  1.00 15.00      A    C
ATOM   2171  CG2 VAL A 283      23.722 -14.453  -2.597  1.00 15.00      A    C
ATOM   2172  C   VAL A 283      25.360 -12.867  -0.706  1.00 15.00      A    C
ATOM   2173  O   VAL A 283      26.537 -12.803  -1.067  1.00 15.00      A    O
ATOM   2174  N   PRO A 284      25.003 -13.473   0.435  1.00 15.00      A    N
ATOM   2175  CA  PRO A 284      25.967 -14.144   1.308  1.00 15.00      A    C
ATOM   2176  CB  PRO A 284      25.132 -14.564   2.526  1.00 15.00      A    C
ATOM   2177  CG  PRO A 284      23.873 -13.770   2.435  1.00 15.00      A    C
ATOM   2178  CD  PRO A 284      23.636 -13.537   0.974  1.00 15.00      A    C
ATOM   2179  C   PRO A 284      26.557 -15.378   0.636  1.00 15.00      A    C
ATOM   2180  O   PRO A 284      27.751 -15.427   0.340  1.00 15.00      A    O
ATOM   2181  N   PHE A 285      25.707 -16.359   0.378  1.00 15.00      A    N
ATOM   2182  CA  PHE A 285      26.134 -17.597  -0.249  1.00 15.00      A    C
ATOM   2183  CB  PHE A 285      25.643 -18.796   0.566  1.00 15.00      A    C
ATOM   2184  CG  PHE A 285      25.934 -18.678   2.037  1.00 15.00      A    C
ATOM   2185  CD1 PHE A 285      27.205 -18.928   2.528  1.00 15.00      A    C
ATOM   2186  CD2 PHE A 285      24.937 -18.311   2.928  1.00 15.00      A    C
ATOM   2187  CE1 PHE A 285      27.477 -18.815   3.879  1.00 15.00      A    C
ATOM   2188  CE2 PHE A 285      25.202 -18.197   4.280  1.00 15.00      A    C
ATOM   2189  CZ  PHE A 285      26.474 -18.449   4.756  1.00 15.00      A    C
ATOM   2190  C   PHE A 285      25.618 -17.666  -1.680  1.00 15.00      A    C
```

ATOM	2191	O	PHE	A	285	24.521	-18.163	-1.934	1.00	15.00	A	O
ATOM	2192	N	GLY	A	286	26.417	-17.160	-2.611	1.00	15.00	A	N
ATOM	2193	CA	GLY	A	286	26.027	-17.148	-4.012	1.00	15.00	A	C
ATOM	2194	C	GLY	A	286	26.104	-18.512	-4.669	1.00	15.00	A	C
ATOM	2195	O	GLY	A	286	25.724	-18.671	-5.825	1.00	15.00	A	O
ATOM	2196	N	GLN	A	287	26.588	-19.500	-3.932	1.00	15.00	A	N
ATOM	2197	CA	GLN	A	287	26.712	-20.850	-4.460	1.00	15.00	A	C
ATOM	2198	CB	GLN	A	287	28.068	-21.447	-4.089	1.00	15.00	A	C
ATOM	2199	CG	GLN	A	287	29.238	-20.677	-4.679	1.00	15.00	A	C
ATOM	2200	CD	GLN	A	287	30.331	-21.583	-5.203	1.00	15.00	A	C
ATOM	2201	OE1	GLN	A	287	31.316	-21.848	-4.521	1.00	15.00	A	O
ATOM	2202	NE2	GLN	A	287	30.156	-22.074	-6.421	1.00	15.00	A	N
ATOM	2203	C	GLN	A	287	25.566	-21.736	-3.983	1.00	15.00	A	C
ATOM	2204	O	GLN	A	287	25.642	-22.963	-4.040	1.00	15.00	A	O
ATOM	2205	N	LYS	A	288	24.499	-21.101	-3.522	1.00	15.00	A	N
ATOM	2206	CA	LYS	A	288	23.330	-21.818	-3.041	1.00	15.00	A	C
ATOM	2207	CB	LYS	A	288	22.948	-21.355	-1.636	1.00	15.00	A	C
ATOM	2208	CG	LYS	A	288	23.806	-21.952	-0.538	1.00	15.00	A	C
ATOM	2209	CD	LYS	A	288	23.526	-23.437	-0.360	1.00	15.00	A	C
ATOM	2210	CE	LYS	A	288	24.559	-24.072	0.551	1.00	15.00	A	C
ATOM	2211	NZ	LYS	A	288	24.133	-25.395	1.074	1.00	15.00	A	N
ATOM	2212	C	LYS	A	288	22.156	-21.634	-3.991	1.00	15.00	A	C
ATOM	2213	O	LYS	A	288	21.749	-20.506	-4.271	1.00	15.00	A	O
ATOM	2214	N	PRO	A	289	21.609	-22.745	-4.505	1.00	15.00	A	N
ATOM	2215	CA	PRO	A	289	20.472	-22.719	-5.429	1.00	15.00	A	C
ATOM	2216	CB	PRO	A	289	20.178	-24.203	-5.693	1.00	15.00	A	C
ATOM	2217	CG	PRO	A	289	20.860	-24.939	-4.590	1.00	15.00	A	C
ATOM	2218	CD	PRO	A	289	22.059	-24.117	-4.226	1.00	15.00	A	C
ATOM	2219	C	PRO	A	289	19.252	-22.041	-4.812	1.00	15.00	A	C
ATOM	2220	O	PRO	A	289	18.846	-22.364	-3.694	1.00	15.00	A	O
ATOM	2221	N	ASN	A	290	18.684	-21.089	-5.539	1.00	15.00	A	N
ATOM	2222	CA	ASN	A	290	17.509	-20.371	-5.066	1.00	15.00	A	C
ATOM	2223	CB	ASN	A	290	17.362	-19.031	-5.791	1.00	15.00	A	C
ATOM	2224	CG	ASN	A	290	17.035	-17.890	-4.844	1.00	15.00	A	C
ATOM	2225	OD1	ASN	A	290	15.918	-17.375	-4.828	1.00	15.00	A	O
ATOM	2226	ND2	ASN	A	290	18.011	-17.491	-4.041	1.00	15.00	A	N
ATOM	2227	C	ASN	A	290	16.261	-21.227	-5.248	1.00	15.00	A	C
ATOM	2228	O	ASN	A	290	16.284	-22.218	-5.978	1.00	15.00	A	O
ATOM	2229	N	ILE	A	291	15.179	-20.845	-4.592	1.00	15.00	A	N
ATOM	2230	CA	ILE	A	291	13.939	-21.600	-4.675	1.00	15.00	A	C
ATOM	2231	CB	ILE	A	291	13.220	-21.668	-3.311	1.00	15.00	A	C
ATOM	2232	CG1	ILE	A	291	14.222	-21.952	-2.187	1.00	15.00	A	C
ATOM	2233	CG2	ILE	A	291	12.134	-22.734	-3.338	1.00	15.00	A	C
ATOM	2234	CD1	ILE	A	291	13.645	-21.802	-0.796	1.00	15.00	A	C
ATOM	2235	C	ILE	A	291	12.992	-21.005	-5.711	1.00	15.00	A	C
ATOM	2236	O	ILE	A	291	12.323	-20.008	-5.448	1.00	15.00	A	O
ATOM	2237	N	ASP	A	292	12.964	-21.609	-6.890	1.00	15.00	A	N
ATOM	2238	CA	ASP	A	292	12.080	-21.169	-7.963	1.00	15.00	A	C
ATOM	2239	CB	ASP	A	292	12.855	-20.463	-9.083	1.00	15.00	A	C
ATOM	2240	CG	ASP	A	292	11.948	-19.883	-10.161	1.00	15.00	A	C
ATOM	2241	OD1	ASP	A	292	10.707	-19.952	-10.013	1.00	15.00	A	O
ATOM	2242	OD2	ASP	A	292	12.469	-19.359	-11.160	1.00	15.00	A	O
ATOM	2243	C	ASP	A	292	11.322	-22.370	-8.512	1.00	15.00	A	C
ATOM	2244	O	ASP	A	292	11.914	-23.413	-8.795	1.00	15.00	A	O
ATOM	2245	N	VAL	A	293	10.013	-22.229	-8.639	1.00	15.00	A	N
ATOM	2246	CA	VAL	A	293	9.175	-23.307	-9.138	1.00	15.00	A	C
ATOM	2247	CB	VAL	A	293	7.998	-23.608	-8.184	1.00	15.00	A	C
ATOM	2248	CG1	VAL	A	293	8.512	-24.054	-6.824	1.00	15.00	A	C
ATOM	2249	CG2	VAL	A	293	7.087	-22.396	-8.045	1.00	15.00	A	C
ATOM	2250	C	VAL	A	293	8.638	-23.002	-10.532	1.00	15.00	A	C
ATOM	2251	O	VAL	A	293	7.817	-23.752	-11.059	1.00	15.00	A	O
ATOM	2252	N	THR	A	294	9.109	-21.908	-11.124	1.00	15.00	A	N
ATOM	2253	CA	THR	A	294	8.672	-21.501	-12.457	1.00	15.00	A	C
ATOM	2254	CB	THR	A	294	9.429	-20.245	-12.936	1.00	15.00	A	C
ATOM	2255	OG1	THR	A	294	9.221	-19.181	-11.996	1.00	15.00	A	O
ATOM	2256	CG2	THR	A	294	8.942	-19.806	-14.309	1.00	15.00	A	C
ATOM	2257	C	THR	A	294	8.835	-22.635	-13.471	1.00	15.00	A	C
ATOM	2258	O	THR	A	294	7.881	-22.993	-14.168	1.00	15.00	A	O
ATOM	2259	N	ASP	A	295	10.031	-23.213	-13.530	1.00	15.00	A	N
ATOM	2260	CA	ASP	A	295	10.308	-24.313	-14.453	1.00	15.00	A	C
ATOM	2261	CB	ASP	A	295	11.767	-24.766	-14.347	1.00	15.00	A	C

```
ATOM   2262  CG  ASP A 295      12.724 -23.867 -15.104  1.00 15.00       A    C
ATOM   2263  OD1 ASP A 295      12.370 -23.418 -16.215  1.00 15.00       A    O
ATOM   2264  OD2 ASP A 295      13.831 -23.613 -14.587  1.00 15.00       A    O
ATOM   2265  C   ASP A 295       9.379 -25.491 -14.182  1.00 15.00       A    C
ATOM   2266  O   ASP A 295       8.778 -26.047 -15.100  1.00 15.00       A    O
ATOM   2267  N   ALA A 296       9.238 -25.838 -12.904  1.00 15.00       A    N
ATOM   2268  CA  ALA A 296       8.382 -26.949 -12.494  1.00 15.00       A    C
ATOM   2269  CB  ALA A 296       8.480 -27.170 -10.993  1.00 15.00       A    C
ATOM   2270  C   ALA A 296       6.933 -26.708 -12.909  1.00 15.00       A    C
ATOM   2271  O   ALA A 296       6.212 -27.643 -13.260  1.00 15.00       A    O
ATOM   2272  N   MET A 297       6.513 -25.449 -12.865  1.00 15.00       A    N
ATOM   2273  CA  MET A 297       5.160 -25.081 -13.255  1.00 15.00       A    C
ATOM   2274  CB  MET A 297       4.873 -23.617 -12.910  1.00 15.00       A    C
ATOM   2275  CG  MET A 297       4.680 -23.354 -11.425  1.00 15.00       A    C
ATOM   2276  SD  MET A 297       4.722 -21.599 -11.008  1.00 15.00       A    S
ATOM   2277  CE  MET A 297       3.297 -21.002 -11.912  1.00 15.00       A    C
ATOM   2278  C   MET A 297       4.963 -25.325 -14.746  1.00 15.00       A    C
ATOM   2279  O   MET A 297       3.922 -25.818 -15.172  1.00 15.00       A    O
ATOM   2280  N   VAL A 298       5.980 -24.992 -15.532  1.00 15.00       A    N
ATOM   2281  CA  VAL A 298       5.923 -25.188 -16.975  1.00 15.00       A    C
ATOM   2282  CB  VAL A 298       7.115 -24.514 -17.694  1.00 15.00       A    C
ATOM   2283  CG1 VAL A 298       7.040 -24.739 -19.198  1.00 15.00       A    C
ATOM   2284  CG2 VAL A 298       7.154 -23.025 -17.383  1.00 15.00       A    C
ATOM   2285  C   VAL A 298       5.902 -26.682 -17.299  1.00 15.00       A    C
ATOM   2286  O   VAL A 298       5.141 -27.128 -18.158  1.00 15.00       A    O
ATOM   2287  N   ASP A 299       6.725 -27.444 -16.582  1.00 15.00       A    N
ATOM   2288  CA  ASP A 299       6.815 -28.894 -16.769  1.00 15.00       A    C
ATOM   2289  CB  ASP A 299       7.841 -29.496 -15.803  1.00 15.00       A    C
ATOM   2290  CG  ASP A 299       9.229 -29.625 -16.401  1.00 15.00       A    C
ATOM   2291  OD1 ASP A 299       9.334 -29.846 -17.625  1.00 15.00       A    O
ATOM   2292  OD2 ASP A 299      10.212 -29.518 -15.638  1.00 15.00       A    O
ATOM   2293  C   ASP A 299       5.467 -29.571 -16.547  1.00 15.00       A    C
ATOM   2294  O   ASP A 299       5.077 -30.468 -17.293  1.00 15.00       A    O
ATOM   2295  N   GLN A 300       4.753 -29.132 -15.517  1.00 15.00       A    N
ATOM   2296  CA  GLN A 300       3.450 -29.702 -15.192  1.00 15.00       A    C
ATOM   2297  CB  GLN A 300       3.161 -29.574 -13.698  1.00 15.00       A    C
ATOM   2298  CG  GLN A 300       4.115 -30.336 -12.797  1.00 15.00       A    C
ATOM   2299  CD  GLN A 300       3.766 -30.174 -11.331  1.00 15.00       A    C
ATOM   2300  OE1 GLN A 300       2.601 -29.987 -10.973  1.00 15.00       A    O
ATOM   2301  NE2 GLN A 300       4.773 -30.237 -10.475  1.00 15.00       A    N
ATOM   2302  C   GLN A 300       2.328 -29.042 -15.991  1.00 15.00       A    C
ATOM   2303  O   GLN A 300       1.149 -29.310 -15.746  1.00 15.00       A    O
ATOM   2304  N   ALA A 301       2.706 -28.178 -16.931  1.00 15.00       A    N
ATOM   2305  CA  ALA A 301       1.751 -27.468 -17.783  1.00 15.00       A    C
ATOM   2306  CB  ALA A 301       1.011 -28.432 -18.704  1.00 15.00       A    C
ATOM   2307  C   ALA A 301       0.777 -26.612 -16.972  1.00 15.00       A    C
ATOM   2308  O   ALA A 301      -0.439 -26.690 -17.153  1.00 15.00       A    O
ATOM   2309  N   TRP A 302       1.318 -25.797 -16.077  1.00 15.00       A    N
ATOM   2310  CA  TRP A 302       0.501 -24.921 -15.246  1.00 15.00       A    C
ATOM   2311  CB  TRP A 302       1.289 -24.455 -14.015  1.00 15.00       A    C
ATOM   2312  CG  TRP A 302       1.319 -25.444 -12.887  1.00 15.00       A    C
ATOM   2313  CD1 TRP A 302       1.658 -26.762 -12.956  1.00 15.00       A    C
ATOM   2314  CD2 TRP A 302       1.004 -25.184 -11.514  1.00 15.00       A    C
ATOM   2315  NE1 TRP A 302       1.570 -27.342 -11.714  1.00 15.00       A    N
ATOM   2316  CE2 TRP A 302       1.170 -26.394 -10.812  1.00 15.00       A    C
ATOM   2317  CE3 TRP A 302       0.597 -24.047 -10.812  1.00 15.00       A    C
ATOM   2318  CZ2 TRP A 302       0.943 -26.498  -9.442  1.00 15.00       A    C
ATOM   2319  CZ3 TRP A 302       0.373 -24.152  -9.453  1.00 15.00       A    C
ATOM   2320  CH2 TRP A 302       0.546 -25.369  -8.783  1.00 15.00       A    C
ATOM   2321  C   TRP A 302       0.029 -23.703 -16.035  1.00 15.00       A    C
ATOM   2322  O   TRP A 302       0.814 -23.066 -16.738  1.00 15.00       A    O
ATOM   2323  N   ASP A 303      -1.252 -23.386 -15.917  1.00 15.00       A    N
ATOM   2324  CA  ASP A 303      -1.822 -22.231 -16.601  1.00 15.00       A    C
ATOM   2325  CB  ASP A 303      -2.909 -22.662 -17.601  1.00 15.00       A    C
ATOM   2326  CG  ASP A 303      -4.316 -22.514 -17.055  1.00 15.00       A    C
ATOM   2327  OD1 ASP A 303      -4.663 -23.238 -16.097  1.00 15.00       A    O
ATOM   2328  OD2 ASP A 303      -5.065 -21.652 -17.564  1.00 15.00       A    O
ATOM   2329  C   ASP A 303      -2.375 -21.241 -15.575  1.00 15.00       A    C
ATOM   2330  O   ASP A 303      -2.296 -21.488 -14.368  1.00 15.00       A    O
ATOM   2331  N   ALA A 304      -2.936 -20.135 -16.055  1.00 15.00       A    N
ATOM   2332  CA  ALA A 304      -3.495 -19.107 -15.183  1.00 15.00       A    C
```

```
ATOM   2333  CB  ALA A 304      -4.039 -17.957 -16.013  1.00 15.00       A    C
ATOM   2334  C   ALA A 304      -4.588 -19.681 -14.289  1.00 15.00       A    C
ATOM   2335  O   ALA A 304      -4.618 -19.422 -13.083  1.00 15.00       A    O
ATOM   2336  N   GLN A 305      -5.467 -20.478 -14.888  1.00 15.00       A    N
ATOM   2337  CA  GLN A 305      -6.560 -21.107 -14.161  1.00 15.00       A    C
ATOM   2338  CB  GLN A 305      -7.403 -21.948 -15.125  1.00 15.00       A    C
ATOM   2339  CG  GLN A 305      -8.477 -22.803 -14.473  1.00 15.00       A    C
ATOM   2340  CD  GLN A 305      -9.717 -22.019 -14.095  1.00 15.00       A    C
ATOM   2341  OE1 GLN A 305      -9.641 -20.954 -13.487  1.00 15.00       A    O
ATOM   2342  NE2 GLN A 305     -10.873 -22.539 -14.470  1.00 15.00       A    N
ATOM   2343  C   GLN A 305      -5.999 -21.978 -13.043  1.00 15.00       A    C
ATOM   2344  O   GLN A 305      -6.439 -21.895 -11.895  1.00 15.00       A    O
ATOM   2345  N   ARG A 306      -5.013 -22.801 -13.386  1.00 15.00       A    N
ATOM   2346  CA  ARG A 306      -4.368 -23.680 -12.419  1.00 15.00       A    C
ATOM   2347  CB  ARG A 306      -3.295 -24.535 -13.099  1.00 15.00       A    C
ATOM   2348  CG  ARG A 306      -2.570 -25.485 -12.159  1.00 15.00       A    C
ATOM   2349  CD  ARG A 306      -3.443 -26.673 -11.790  1.00 15.00       A    C
ATOM   2350  NE  ARG A 306      -2.940 -27.384 -10.614  1.00 15.00       A    N
ATOM   2351  CZ  ARG A 306      -2.095 -28.413 -10.660  1.00 15.00       A    C
ATOM   2352  NH1 ARG A 306      -1.642 -28.863 -11.829  1.00 15.00       A    N
ATOM   2353  NH2 ARG A 306      -1.710 -28.994  -9.529  1.00 15.00       A    N
ATOM   2354  C   ARG A 306      -3.748 -22.878 -11.278  1.00 15.00       A    C
ATOM   2355  O   ARG A 306      -3.883 -23.245 -10.112  1.00 15.00       A    O
ATOM   2356  N   ILE A 307      -3.080 -21.781 -11.619  1.00 15.00       A    N
ATOM   2357  CA  ILE A 307      -2.442 -20.932 -10.619  1.00 15.00       A    C
ATOM   2358  CB  ILE A 307      -1.670 -19.761 -11.278  1.00 15.00       A    C
ATOM   2359  CG1 ILE A 307      -0.490 -20.298 -12.094  1.00 15.00       A    C
ATOM   2360  CG2 ILE A 307      -1.186 -18.765 -10.232  1.00 15.00       A    C
ATOM   2361  CD1 ILE A 307       0.156 -19.269 -12.998  1.00 15.00       A    C
ATOM   2362  C   ILE A 307      -3.474 -20.388  -9.625  1.00 15.00       A    C
ATOM   2363  O   ILE A 307      -3.304 -20.504  -8.411  1.00 15.00       A    O
ATOM   2364  N   PHE A 308      -4.556 -19.829 -10.150  1.00 15.00       A    N
ATOM   2365  CA  PHE A 308      -5.613 -19.255  -9.314  1.00 15.00       A    C
ATOM   2366  CB  PHE A 308      -6.528 -18.354 -10.141  1.00 15.00       A    C
ATOM   2367  CG  PHE A 308      -6.007 -16.955 -10.303  1.00 15.00       A    C
ATOM   2368  CD1 PHE A 308      -5.111 -16.645 -11.313  1.00 15.00       A    C
ATOM   2369  CD2 PHE A 308      -6.411 -15.950  -9.440  1.00 15.00       A    C
ATOM   2370  CE1 PHE A 308      -4.629 -15.360 -11.458  1.00 15.00       A    C
ATOM   2371  CE2 PHE A 308      -5.933 -14.662  -9.580  1.00 15.00       A    C
ATOM   2372  CZ  PHE A 308      -5.039 -14.365 -10.591  1.00 15.00       A    C
ATOM   2373  C   PHE A 308      -6.421 -20.315  -8.557  1.00 15.00       A    C
ATOM   2374  O   PHE A 308      -6.933 -20.058  -7.465  1.00 15.00       A    O
ATOM   2375  N   LYS A 309      -6.540 -21.496  -9.145  1.00 15.00       A    N
ATOM   2376  CA  LYS A 309      -7.270 -22.596  -8.522  1.00 15.00       A    C
ATOM   2377  CB  LYS A 309      -7.499 -23.728  -9.522  1.00 15.00       A    C
ATOM   2378  CG  LYS A 309      -8.835 -23.639 -10.234  1.00 15.00       A    C
ATOM   2379  CD  LYS A 309      -9.981 -23.643  -9.240  1.00 15.00       A    C
ATOM   2380  CE  LYS A 309     -11.226 -23.040  -9.856  1.00 15.00       A    C
ATOM   2381  NZ  LYS A 309     -12.338 -22.939  -8.879  1.00 15.00       A    N
ATOM   2382  C   LYS A 309      -6.542 -23.109  -7.285  1.00 15.00       A    C
ATOM   2383  O   LYS A 309      -7.158 -23.476  -6.283  1.00 15.00       A    O
ATOM   2384  N   GLU A 310      -5.230 -23.118  -7.356  1.00 15.00       A    N
ATOM   2385  CA  GLU A 310      -4.418 -23.564  -6.241  1.00 15.00       A    C
ATOM   2386  CB  GLU A 310      -2.968 -23.690  -6.681  1.00 15.00       A    C
ATOM   2387  CG  GLU A 310      -2.711 -24.833  -7.647  1.00 15.00       A    C
ATOM   2388  CD  GLU A 310      -3.222 -26.158  -7.132  1.00 15.00       A    C
ATOM   2389  OE1 GLU A 310      -3.253 -26.350  -5.904  1.00 15.00       A    O
ATOM   2390  OE2 GLU A 310      -3.598 -27.022  -7.955  1.00 15.00       A    O
ATOM   2391  C   GLU A 310      -4.522 -22.563  -5.105  1.00 15.00       A    C
ATOM   2392  O   GLU A 310      -4.564 -22.930  -3.929  1.00 15.00       A    O
ATOM   2393  N   ALA A 311      -4.583 -21.294  -5.479  1.00 15.00       A    N
ATOM   2394  CA  ALA A 311      -4.700 -20.219  -4.514  1.00 15.00       A    C
ATOM   2395  CB  ALA A 311      -4.550 -18.869  -5.196  1.00 15.00       A    C
ATOM   2396  C   ALA A 311      -6.035 -20.316  -3.799  1.00 15.00       A    C
ATOM   2397  O   ALA A 311      -6.106 -20.161  -2.581  1.00 15.00       A    O
ATOM   2398  N   GLU A 312      -7.088 -20.586  -4.569  1.00 15.00       A    N
ATOM   2399  CA  GLU A 312      -8.429 -20.732  -4.020  1.00 15.00       A    C
ATOM   2400  CB  GLU A 312      -9.416 -21.011  -5.156  1.00 15.00       A    C
ATOM   2401  CG  GLU A 312     -10.859 -21.181  -4.714  1.00 15.00       A    C
ATOM   2402  CD  GLU A 312     -11.750 -21.679  -5.835  1.00 15.00       A    C
ATOM   2403  OE1 GLU A 312     -11.565 -22.831  -6.286  1.00 15.00       A    O
```

ATOM	2404	OE2	GLU	A	312	-12.638	-20.921	-6.279	1.00	15.00	A	O
ATOM	2405	C	GLU	A	312	-8.446	-21.879	-3.013	1.00	15.00	A	C
ATOM	2406	O	GLU	A	312	-8.991	-21.760	-1.913	1.00	15.00	A	O
ATOM	2407	N	LYS	A	313	-7.822	-22.984	-3.403	1.00	15.00	A	N
ATOM	2408	CA	LYS	A	313	-7.726	-24.168	-2.558	1.00	15.00	A	C
ATOM	2409	CB	LYS	A	313	-6.961	-25.258	-3.307	1.00	15.00	A	C
ATOM	2410	CG	LYS	A	313	-6.919	-26.610	-2.613	1.00	15.00	A	C
ATOM	2411	CD	LYS	A	313	-5.950	-27.547	-3.317	1.00	15.00	A	C
ATOM	2412	CE	LYS	A	313	-6.262	-27.641	-4.804	1.00	15.00	A	C
ATOM	2413	NZ	LYS	A	313	-5.104	-28.140	-5.590	1.00	15.00	A	N
ATOM	2414	C	LYS	A	313	-7.004	-23.837	-1.254	1.00	15.00	A	C
ATOM	2415	O	LYS	A	313	-7.350	-24.355	-0.190	1.00	15.00	A	O
ATOM	2416	N	PHE	A	314	-6.002	-22.970	-1.353	1.00	15.00	A	N
ATOM	2417	CA	PHE	A	314	-5.222	-22.547	-0.196	1.00	15.00	A	C
ATOM	2418	CB	PHE	A	314	-4.086	-21.607	-0.628	1.00	15.00	A	C
ATOM	2419	CG	PHE	A	314	-3.478	-20.791	0.483	1.00	15.00	A	C
ATOM	2420	CD1	PHE	A	314	-2.632	-21.374	1.413	1.00	15.00	A	C
ATOM	2421	CD2	PHE	A	314	-3.748	-19.435	0.590	1.00	15.00	A	C
ATOM	2422	CE1	PHE	A	314	-2.069	-20.622	2.427	1.00	15.00	A	C
ATOM	2423	CE2	PHE	A	314	-3.190	-18.678	1.602	1.00	15.00	A	C
ATOM	2424	CZ	PHE	A	314	-2.348	-19.272	2.522	1.00	15.00	A	C
ATOM	2425	C	PHE	A	314	-6.116	-21.877	0.844	1.00	15.00	A	C
ATOM	2426	O	PHE	A	314	-6.043	-22.192	2.031	1.00	15.00	A	O
ATOM	2427	N	PHE	A	315	-6.974	-20.973	0.389	1.00	15.00	A	N
ATOM	2428	CA	PHE	A	315	-7.879	-20.265	1.287	1.00	15.00	A	C
ATOM	2429	CB	PHE	A	315	-8.573	-19.107	0.566	1.00	15.00	A	C
ATOM	2430	CG	PHE	A	315	-7.675	-17.928	0.331	1.00	15.00	A	C
ATOM	2431	CD1	PHE	A	315	-7.593	-16.907	1.263	1.00	15.00	A	C
ATOM	2432	CD2	PHE	A	315	-6.906	-17.844	-0.815	1.00	15.00	A	C
ATOM	2433	CE1	PHE	A	315	-6.762	-15.825	1.055	1.00	15.00	A	C
ATOM	2434	CE2	PHE	A	315	-6.073	-16.765	-1.032	1.00	15.00	A	C
ATOM	2435	CZ	PHE	A	315	-6.000	-15.754	-0.095	1.00	15.00	A	C
ATOM	2436	C	PHE	A	315	-8.906	-21.214	1.892	1.00	15.00	A	C
ATOM	2437	O	PHE	A	315	-9.203	-21.144	3.083	1.00	15.00	A	O
ATOM	2438	N	VAL	A	316	-9.427	-22.113	1.072	1.00	15.00	A	N
ATOM	2439	CA	VAL	A	316	-10.419	-23.080	1.526	1.00	15.00	A	C
ATOM	2440	CB	VAL	A	316	-10.948	-23.942	0.356	1.00	15.00	A	C
ATOM	2441	CG1	VAL	A	316	-11.934	-24.992	0.848	1.00	15.00	A	C
ATOM	2442	CG2	VAL	A	316	-11.599	-23.061	-0.699	1.00	15.00	A	C
ATOM	2443	C	VAL	A	316	-9.861	-23.981	2.633	1.00	15.00	A	C
ATOM	2444	O	VAL	A	316	-10.569	-24.327	3.578	1.00	15.00	A	O
ATOM	2445	N	SER	A	317	-8.579	-24.323	2.529	1.00	15.00	A	N
ATOM	2446	CA	SER	A	317	-7.934	-25.195	3.510	1.00	15.00	A	C
ATOM	2447	CB	SER	A	317	-6.516	-25.574	3.066	1.00	15.00	A	C
ATOM	2448	OG	SER	A	317	-5.591	-24.524	3.307	1.00	15.00	A	O
ATOM	2449	C	SER	A	317	-7.917	-24.597	4.919	1.00	15.00	A	C
ATOM	2450	O	SER	A	317	-7.862	-25.328	5.906	1.00	15.00	A	O
ATOM	2451	N	VAL	A	318	-7.975	-23.272	5.019	1.00	15.00	A	N
ATOM	2452	CA	VAL	A	318	-7.949	-22.622	6.325	1.00	15.00	A	C
ATOM	2453	CB	VAL	A	318	-7.065	-21.352	6.351	1.00	15.00	A	C
ATOM	2454	CG1	VAL	A	318	-5.665	-21.664	5.846	1.00	15.00	A	C
ATOM	2455	CG2	VAL	A	318	-7.685	-20.220	5.552	1.00	15.00	A	C
ATOM	2456	C	VAL	A	318	-9.352	-22.324	6.855	1.00	15.00	A	C
ATOM	2457	O	VAL	A	318	-9.509	-21.694	7.901	1.00	15.00	A	O
ATOM	2458	N	GLY	A	319	-10.368	-22.783	6.132	1.00	15.00	A	N
ATOM	2459	CA	GLY	A	319	-11.737	-22.569	6.563	1.00	15.00	A	C
ATOM	2460	C	GLY	A	319	-12.398	-21.376	5.901	1.00	15.00	A	C
ATOM	2461	O	GLY	A	319	-13.474	-20.948	6.317	1.00	15.00	A	O
ATOM	2462	N	LEU	A	320	-11.762	-20.836	4.873	1.00	15.00	A	N
ATOM	2463	CA	LEU	A	320	-12.312	-19.693	4.161	1.00	15.00	A	C
ATOM	2464	CB	LEU	A	320	-11.203	-18.708	3.777	1.00	15.00	A	C
ATOM	2465	CG	LEU	A	320	-10.527	-17.974	4.940	1.00	15.00	A	C
ATOM	2466	CD1	LEU	A	320	-9.369	-17.125	4.441	1.00	15.00	A	C
ATOM	2467	CD2	LEU	A	320	-11.533	-17.118	5.695	1.00	15.00	A	C
ATOM	2468	C	LEU	A	320	-13.086	-20.155	2.930	1.00	15.00	A	C
ATOM	2469	O	LEU	A	320	-12.756	-21.182	2.335	1.00	15.00	A	O
ATOM	2470	N	PRO	A	321	-14.139	-19.417	2.543	1.00	15.00	A	N
ATOM	2471	CA	PRO	A	321	-14.964	-19.766	1.382	1.00	15.00	A	C
ATOM	2472	CB	PRO	A	321	-16.088	-18.723	1.410	1.00	15.00	A	C
ATOM	2473	CG	PRO	A	321	-15.521	-17.580	2.176	1.00	15.00	A	C
ATOM	2474	CD	PRO	A	321	-14.611	-18.189	3.204	1.00	15.00	A	C

```
ATOM   2475  C   PRO A 321     -14.199 -19.677   0.065  1.00 15.00      A    C
ATOM   2476  O   PRO A 321     -13.161 -19.017  -0.029  1.00 15.00      A    O
ATOM   2477  N   ASN A 322     -14.728 -20.346  -0.950  1.00 15.00      A    N
ATOM   2478  CA  ASN A 322     -14.120 -20.349  -2.273  1.00 15.00      A    C
ATOM   2479  CB  ASN A 322     -14.510 -21.610  -3.063  1.00 15.00      A    C
ATOM   2480  CG  ASN A 322     -16.002 -21.920  -3.046  1.00 15.00      A    C
ATOM   2481  OD1 ASN A 322     -16.834 -21.058  -2.772  1.00 15.00      A    O
ATOM   2482  ND2 ASN A 322     -16.348 -23.164  -3.339  1.00 15.00      A    N
ATOM   2483  C   ASN A 322     -14.514 -19.094  -3.043  1.00 15.00      A    C
ATOM   2484  O   ASN A 322     -15.193 -18.214  -2.508  1.00 15.00      A    O
ATOM   2485  N   MET A 323     -14.089 -19.010  -4.293  1.00 15.00      A    N
ATOM   2486  CA  MET A 323     -14.409 -17.859  -5.119  1.00 15.00      A    C
ATOM   2487  CB  MET A 323     -13.349 -17.663  -6.203  1.00 15.00      A    C
ATOM   2488  CG  MET A 323     -11.947 -17.383  -5.677  1.00 15.00      A    C
ATOM   2489  SD  MET A 323     -11.741 -15.696  -5.064  1.00 15.00      A    S
ATOM   2490  CE  MET A 323     -12.283 -15.875  -3.366  1.00 15.00      A    C
ATOM   2491  C   MET A 323     -15.785 -18.032  -5.746  1.00 15.00      A    C
ATOM   2492  O   MET A 323     -16.162 -19.138  -6.135  1.00 15.00      A    O
ATOM   2493  N   THR A 324     -16.534 -16.942  -5.831  1.00 15.00      A    N
ATOM   2494  CA  THR A 324     -17.870 -16.976  -6.404  1.00 15.00      A    C
ATOM   2495  CB  THR A 324     -18.581 -15.623  -6.211  1.00 15.00      A    C
ATOM   2496  OG1 THR A 324     -17.620 -14.562  -6.307  1.00 15.00      A    O
ATOM   2497  CG2 THR A 324     -19.257 -15.566  -4.850  1.00 15.00      A    C
ATOM   2498  C   THR A 324     -17.823 -17.322  -7.892  1.00 15.00      A    C
ATOM   2499  O   THR A 324     -16.803 -17.115  -8.553  1.00 15.00      A    O
ATOM   2500  N   GLN A 325     -18.927 -17.852  -8.414  1.00 15.00      A    N
ATOM   2501  CA  GLN A 325     -19.006 -18.223  -9.823  1.00 15.00      A    C
ATOM   2502  CB  GLN A 325     -20.391 -18.784 -10.156  1.00 15.00      A    C
ATOM   2503  CG  GLN A 325     -20.490 -19.396 -11.544  1.00 15.00      A    C
ATOM   2504  CD  GLN A 325     -21.915 -19.723 -11.947  1.00 15.00      A    C
ATOM   2505  OE1 GLN A 325     -22.750 -20.067 -11.107  1.00 15.00      A    O
ATOM   2506  NE2 GLN A 325     -22.206 -19.602 -13.232  1.00 15.00      A    N
ATOM   2507  C   GLN A 325     -18.695 -17.022 -10.711  1.00 15.00      A    C
ATOM   2508  O   GLN A 325     -17.869 -17.110 -11.619  1.00 15.00      A    O
ATOM   2509  N   GLY A 326     -19.326 -15.890 -10.397  1.00 15.00      A    N
ATOM   2510  CA  GLY A 326     -19.122 -14.671 -11.161  1.00 15.00      A    C
ATOM   2511  C   GLY A 326     -17.672 -14.233 -11.197  1.00 15.00      A    C
ATOM   2512  O   GLY A 326     -17.251 -13.541 -12.120  1.00 15.00      A    O
ATOM   2513  N   PHE A 327     -16.901 -14.641 -10.199  1.00 15.00      A    N
ATOM   2514  CA  PHE A 327     -15.494 -14.289 -10.142  1.00 15.00      A    C
ATOM   2515  CB  PHE A 327     -14.899 -14.658  -8.776  1.00 15.00      A    C
ATOM   2516  CG  PHE A 327     -13.398 -14.572  -8.717  1.00 15.00      A    C
ATOM   2517  CD1 PHE A 327     -12.614 -15.691  -8.954  1.00 15.00      A    C
ATOM   2518  CD2 PHE A 327     -12.772 -13.372  -8.431  1.00 15.00      A    C
ATOM   2519  CE1 PHE A 327     -11.236 -15.614  -8.906  1.00 15.00      A    C
ATOM   2520  CE2 PHE A 327     -11.395 -13.289  -8.380  1.00 15.00      A    C
ATOM   2521  CZ  PHE A 327     -10.626 -14.410  -8.618  1.00 15.00      A    C
ATOM   2522  C   PHE A 327     -14.738 -15.012 -11.246  1.00 15.00      A    C
ATOM   2523  O   PHE A 327     -13.906 -14.426 -11.930  1.00 15.00      A    O
ATOM   2524  N   TRP A 328     -15.062 -16.280 -11.433  1.00 15.00      A    N
ATOM   2525  CA  TRP A 328     -14.400 -17.095 -12.439  1.00 15.00      A    C
ATOM   2526  CB  TRP A 328     -14.571 -18.588 -12.119  1.00 15.00      A    C
ATOM   2527  CG  TRP A 328     -13.825 -19.024 -10.887  1.00 15.00      A    C
ATOM   2528  CD1 TRP A 328     -14.360 -19.483  -9.714  1.00 15.00      A    C
ATOM   2529  CD2 TRP A 328     -12.403 -19.036 -10.708  1.00 15.00      A    C
ATOM   2530  NE1 TRP A 328     -13.355 -19.782  -8.820  1.00 15.00      A    N
ATOM   2531  CE2 TRP A 328     -12.147 -19.511  -9.406  1.00 15.00      A    C
ATOM   2532  CE3 TRP A 328     -11.320 -18.690 -11.522  1.00 15.00      A    C
ATOM   2533  CZ2 TRP A 328     -10.857 -19.648  -8.902  1.00 15.00      A    C
ATOM   2534  CZ3 TRP A 328     -10.042 -18.827 -11.017  1.00 15.00      A    C
ATOM   2535  CH2 TRP A 328      -9.821 -19.302  -9.721  1.00 15.00      A    C
ATOM   2536  C   TRP A 328     -14.886 -16.775 -13.853  1.00 15.00      A    C
ATOM   2537  O   TRP A 328     -14.253 -17.161 -14.833  1.00 15.00      A    O
ATOM   2538  N   GLU A 329     -15.989 -16.045 -13.955  1.00 15.00      A    N
ATOM   2539  CA  GLU A 329     -16.556 -15.697 -15.253  1.00 15.00      A    C
ATOM   2540  CB  GLU A 329     -18.061 -15.998 -15.270  1.00 15.00      A    C
ATOM   2541  CG  GLU A 329     -18.414 -17.456 -15.010  1.00 15.00      A    C
ATOM   2542  CD  GLU A 329     -19.841 -17.643 -14.530  1.00 15.00      A    C
ATOM   2543  OE1 GLU A 329     -20.547 -18.519 -15.077  1.00 15.00      A    O
ATOM   2544  OE2 GLU A 329     -20.256 -16.924 -13.596  1.00 15.00      A    O
ATOM   2545  C   GLU A 329     -16.321 -14.232 -15.634  1.00 15.00      A    C
```

ATOM	2546	O	GLU	A	329	-16.271	-13.897	-16.817	1.00	15.00	A	O
ATOM	2547	N	ASN	A	330	-16.151	-13.362	-14.642	1.00	15.00	A	N
ATOM	2548	CA	ASN	A	330	-15.977	-11.932	-14.910	1.00	15.00	A	C
ATOM	2549	CB	ASN	A	330	-17.011	-11.120	-14.124	1.00	15.00	A	C
ATOM	2550	CG	ASN	A	330	-18.390	-11.152	-14.748	1.00	15.00	A	C
ATOM	2551	OD1	ASN	A	330	-18.531	-11.139	-15.969	1.00	15.00	A	O
ATOM	2552	ND2	ASN	A	330	-19.420	-11.185	-13.914	1.00	15.00	A	N
ATOM	2553	C	ASN	A	330	-14.575	-11.407	-14.611	1.00	15.00	A	C
ATOM	2554	O	ASN	A	330	-14.369	-10.192	-14.550	1.00	15.00	A	O
ATOM	2555	N	SER	A	331	-13.611	-12.296	-14.441	1.00	15.00	A	N
ATOM	2556	CA	SER	A	331	-12.246	-11.872	-14.145	1.00	15.00	A	C
ATOM	2557	CB	SER	A	331	-11.712	-12.563	-12.891	1.00	15.00	A	C
ATOM	2558	OG	SER	A	331	-12.446	-12.178	-11.744	1.00	15.00	A	O
ATOM	2559	C	SER	A	331	-11.302	-12.109	-15.317	1.00	15.00	A	C
ATOM	2560	O	SER	A	331	-11.546	-12.964	-16.166	1.00	15.00	A	O
ATOM	2561	N	MET	A	332	-10.224	-11.339	-15.353	1.00	15.00	A	N
ATOM	2562	CA	MET	A	332	-9.221	-11.464	-16.399	1.00	15.00	A	C
ATOM	2563	CB	MET	A	332	-9.037	-10.136	-17.141	1.00	15.00	A	C
ATOM	2564	CG	MET	A	332	-8.079	-10.211	-18.322	1.00	15.00	A	C
ATOM	2565	SD	MET	A	332	-8.210	-8.793	-19.431	1.00	15.00	A	S
ATOM	2566	CE	MET	A	332	-9.700	-9.204	-20.336	1.00	15.00	A	C
ATOM	2567	C	MET	A	332	-7.912	-11.914	-15.771	1.00	15.00	A	C
ATOM	2568	O	MET	A	332	-7.148	-11.101	-15.254	1.00	15.00	A	O
ATOM	2569	N	LEU	A	333	-7.678	-13.217	-15.798	1.00	15.00	A	N
ATOM	2570	CA	LEU	A	333	-6.481	-13.796	-15.210	1.00	15.00	A	C
ATOM	2571	CB	LEU	A	333	-6.862	-15.067	-14.450	1.00	15.00	A	C
ATOM	2572	CG	LEU	A	333	-8.222	-15.036	-13.745	1.00	15.00	A	C
ATOM	2573	CD1	LEU	A	333	-8.639	-16.431	-13.315	1.00	15.00	A	C
ATOM	2574	CD2	LEU	A	333	-8.195	-14.089	-12.555	1.00	15.00	A	C
ATOM	2575	C	LEU	A	333	-5.457	-14.121	-16.289	1.00	15.00	A	C
ATOM	2576	O	LEU	A	333	-4.437	-14.754	-16.027	1.00	15.00	A	O
ATOM	2577	N	THR	A	334	-5.737	-13.666	-17.498	1.00	15.00	A	N
ATOM	2578	CA	THR	A	334	-4.869	-13.903	-18.637	1.00	15.00	A	C
ATOM	2579	CB	THR	A	334	-5.415	-15.056	-19.508	1.00	15.00	A	C
ATOM	2580	OG1	THR	A	334	-6.631	-15.565	-18.938	1.00	15.00	A	O
ATOM	2581	CG2	THR	A	334	-4.397	-16.179	-19.618	1.00	15.00	A	C
ATOM	2582	C	THR	A	334	-4.794	-12.647	-19.497	1.00	15.00	A	C
ATOM	2583	O	THR	A	334	-5.509	-11.679	-19.248	1.00	15.00	A	O
ATOM	2584	N	ASP	A	335	-3.931	-12.667	-20.503	1.00	15.00	A	N
ATOM	2585	CA	ASP	A	335	-3.783	-11.533	-21.406	1.00	15.00	A	C
ATOM	2586	CB	ASP	A	335	-2.476	-11.650	-22.193	1.00	15.00	A	C
ATOM	2587	CG	ASP	A	335	-1.467	-10.586	-21.817	1.00	15.00	A	C
ATOM	2588	OD1	ASP	A	335	-1.436	-9.532	-22.481	1.00	15.00	A	O
ATOM	2589	OD2	ASP	A	335	-0.693	-10.818	-20.858	1.00	15.00	A	O
ATOM	2590	C	ASP	A	335	-4.948	-11.468	-22.385	1.00	15.00	A	C
ATOM	2591	O	ASP	A	335	-5.480	-12.502	-22.795	1.00	15.00	A	O
ATOM	2592	N	PRO	A	336	-5.364	-10.249	-22.760	1.00	15.00	A	N
ATOM	2593	CA	PRO	A	336	-6.458	-10.035	-23.714	1.00	15.00	A	C
ATOM	2594	CB	PRO	A	336	-6.925	-8.617	-23.383	1.00	15.00	A	C
ATOM	2595	CG	PRO	A	336	-5.709	-7.924	-22.862	1.00	15.00	A	C
ATOM	2596	CD	PRO	A	336	-4.818	-8.976	-22.251	1.00	15.00	A	C
ATOM	2597	C	PRO	A	336	-5.960	-10.106	-25.159	1.00	15.00	A	C
ATOM	2598	O	PRO	A	336	-6.714	-9.888	-26.112	1.00	15.00	A	O
ATOM	2599	N	GLY	A	337	-4.678	-10.403	-25.309	1.00	15.00	A	N
ATOM	2600	CA	GLY	A	337	-4.086	-10.506	-26.622	1.00	15.00	A	C
ATOM	2601	C	GLY	A	337	-3.518	-9.186	-27.090	1.00	15.00	A	C
ATOM	2602	O	GLY	A	337	-3.459	-8.223	-26.329	1.00	15.00	A	O
ATOM	2603	N	ASN	A	338	-3.109	-9.129	-28.347	1.00	15.00	A	N
ATOM	2604	CA	ASN	A	338	-2.541	-7.905	-28.899	1.00	15.00	A	C
ATOM	2605	CB	ASN	A	338	-1.403	-8.217	-29.877	1.00	15.00	A	C
ATOM	2606	CG	ASN	A	338	-0.514	-7.015	-30.158	1.00	15.00	A	C
ATOM	2607	OD1	ASN	A	338	-0.348	-6.130	-29.315	1.00	15.00	A	O
ATOM	2608	ND2	ASN	A	338	0.069	-6.975	-31.346	1.00	15.00	A	N
ATOM	2609	C	ASN	A	338	-3.613	-7.051	-29.570	1.00	15.00	A	C
ATOM	2610	O	ASN	A	338	-3.325	-5.977	-30.094	1.00	15.00	A	O
ATOM	2611	N	VAL	A	339	-4.851	-7.529	-29.541	1.00	15.00	A	N
ATOM	2612	CA	VAL	A	339	-5.962	-6.801	-30.144	1.00	15.00	A	C
ATOM	2613	CB	VAL	A	339	-7.137	-7.740	-30.494	1.00	15.00	A	C
ATOM	2614	CG1	VAL	A	339	-8.168	-7.017	-31.348	1.00	15.00	A	C
ATOM	2615	CG2	VAL	A	339	-6.632	-8.987	-31.206	1.00	15.00	A	C
ATOM	2616	C	VAL	A	339	-6.441	-5.716	-29.185	1.00	15.00	A	C

ATOM	2617	O	VAL	A	339	-6.325	-4.524	-29.468	1.00	15.00	A	O
ATOM	2618	N	GLN	A	340	-6.960	-6.139	-28.043	1.00	15.00	A	N
ATOM	2619	CA	GLN	A	340	-7.435	-5.210	-27.032	1.00	15.00	A	C
ATOM	2620	CB	GLN	A	340	-8.765	-5.676	-26.429	1.00	15.00	A	C
ATOM	2621	CG	GLN	A	340	-8.895	-7.183	-26.264	1.00	15.00	A	C
ATOM	2622	CD	GLN	A	340	-10.048	-7.578	-25.360	1.00	15.00	A	C
ATOM	2623	OE1	GLN	A	340	-10.740	-8.561	-25.613	1.00	15.00	A	O
ATOM	2624	NE2	GLN	A	340	-10.251	-6.828	-24.287	1.00	15.00	A	N
ATOM	2625	C	GLN	A	340	-6.383	-5.065	-25.946	1.00	15.00	A	C
ATOM	2626	O	GLN	A	340	-5.868	-6.059	-25.441	1.00	15.00	A	O
ATOM	2627	N	LYS	A	341	-6.044	-3.835	-25.606	1.00	15.00	A	N
ATOM	2628	CA	LYS	A	341	-5.042	-3.592	-24.582	1.00	15.00	A	C
ATOM	2629	CB	LYS	A	341	-4.169	-2.386	-24.938	1.00	15.00	A	C
ATOM	2630	CG	LYS	A	341	-3.298	-2.606	-26.166	1.00	15.00	A	C
ATOM	2631	CD	LYS	A	341	-2.539	-3.923	-26.070	1.00	15.00	A	C
ATOM	2632	CE	LYS	A	341	-2.041	-4.380	-27.430	1.00	15.00	A	C
ATOM	2633	NZ	LYS	A	341	-0.713	-3.800	-27.769	1.00	15.00	A	N
ATOM	2634	C	LYS	A	341	-5.687	-3.422	-23.216	1.00	15.00	A	C
ATOM	2635	O	LYS	A	341	-6.754	-2.825	-23.098	1.00	15.00	A	O
ATOM	2636	N	ALA	A	342	-5.038	-3.955	-22.194	1.00	15.00	A	N
ATOM	2637	CA	ALA	A	342	-5.551	-3.869	-20.838	1.00	15.00	A	C
ATOM	2638	CB	ALA	A	342	-6.176	-5.196	-20.433	1.00	15.00	A	C
ATOM	2639	C	ALA	A	342	-4.435	-3.504	-19.874	1.00	15.00	A	C
ATOM	2640	O	ALA	A	342	-3.256	-3.533	-20.232	1.00	15.00	A	O
ATOM	2641	N	VAL	A	343	-4.811	-3.141	-18.660	1.00	15.00	A	N
ATOM	2642	CA	VAL	A	343	-3.843	-2.788	-17.637	1.00	15.00	A	C
ATOM	2643	CB	VAL	A	343	-4.454	-1.831	-16.590	1.00	15.00	A	C
ATOM	2644	CG1	VAL	A	343	-3.411	-1.413	-15.563	1.00	15.00	A	C
ATOM	2645	CG2	VAL	A	343	-5.054	-0.608	-17.270	1.00	15.00	A	C
ATOM	2646	C	VAL	A	343	-3.352	-4.056	-16.945	1.00	15.00	A	C
ATOM	2647	O	VAL	A	343	-4.056	-4.634	-16.119	1.00	15.00	A	O
ATOM	2648	N	CYS	A	344	-2.148	-4.494	-17.292	1.00	15.00	A	N
ATOM	2649	CA	CYS	A	344	-1.572	-5.701	-16.705	1.00	15.00	A	C
ATOM	2650	C	CYS	A	344	-1.025	-5.463	-15.297	1.00	15.00	A	C
ATOM	2651	O	CYS	A	344	0.042	-5.962	-14.941	1.00	15.00	A	O
ATOM	2652	CB	CYS	A	344	-0.489	-6.290	-17.612	1.00	15.00	A	C
ATOM	2653	SG	CYS	A	344	-1.131	-7.145	-19.086	1.00	15.00	A	S
ATOM	2654	N	HIS	A	345	-1.766	-4.713	-14.497	1.00	15.00	A	N
ATOM	2655	CA	HIS	A	345	-1.367	-4.426	-13.130	1.00	15.00	A	C
ATOM	2656	CB	HIS	A	345	-1.394	-2.915	-12.856	1.00	15.00	A	C
ATOM	2657	CG	HIS	A	345	-0.899	-2.528	-11.492	1.00	15.00	A	C
ATOM	2658	ND1	HIS	A	345	-1.675	-1.785	-10.634	1.00	15.00	A	N
ATOM	2659	CD2	HIS	A	345	0.286	-2.807	-10.890	1.00	15.00	A	C
ATOM	2660	CE1	HIS	A	345	-0.958	-1.630	-9.537	1.00	15.00	A	C
ATOM	2661	NE2	HIS	A	345	0.233	-2.231	-9.646	1.00	15.00	A	N
ATOM	2662	C	HIS	A	345	-2.298	-5.168	-12.179	1.00	15.00	A	C
ATOM	2663	O	HIS	A	345	-3.502	-4.911	-12.159	1.00	15.00	A	O
ATOM	2664	N	PRO	A	346	-1.750	-6.123	-11.409	1.00	15.00	A	N
ATOM	2665	CA	PRO	A	346	-2.528	-6.926	-10.457	1.00	15.00	A	C
ATOM	2666	CB	PRO	A	346	-1.455	-7.693	-9.680	1.00	15.00	A	C
ATOM	2667	CG	PRO	A	346	-0.299	-7.766	-10.615	1.00	15.00	A	C
ATOM	2668	CD	PRO	A	346	-0.322	-6.486	-11.398	1.00	15.00	A	C
ATOM	2669	C	PRO	A	346	-3.338	-6.058	-9.506	1.00	15.00	A	C
ATOM	2670	O	PRO	A	346	-2.777	-5.280	-8.734	1.00	15.00	A	O
ATOM	2671	N	THR	A	347	-4.657	-6.174	-9.590	1.00	15.00	A	N
ATOM	2672	CA	THR	A	347	-5.541	-5.402	-8.736	1.00	15.00	A	C
ATOM	2673	CB	THR	A	347	-5.942	-4.070	-9.411	1.00	15.00	A	C
ATOM	2674	OG1	THR	A	347	-6.016	-4.247	-10.836	1.00	15.00	A	O
ATOM	2675	CG2	THR	A	347	-4.947	-2.966	-9.083	1.00	15.00	A	C
ATOM	2676	C	THR	A	347	-6.814	-6.173	-8.389	1.00	15.00	A	C
ATOM	2677	O	THR	A	347	-7.392	-6.860	-9.242	1.00	15.00	A	O
ATOM	2678	N	ALA	A	348	-7.220	-6.087	-7.127	1.00	15.00	A	N
ATOM	2679	CA	ALA	A	348	-8.449	-6.716	-6.664	1.00	15.00	A	C
ATOM	2680	CB	ALA	A	348	-8.377	-6.969	-5.167	1.00	15.00	A	C
ATOM	2681	C	ALA	A	348	-9.600	-5.773	-6.991	1.00	15.00	A	C
ATOM	2682	O	ALA	A	348	-9.474	-4.561	-6.812	1.00	15.00	A	O
ATOM	2683	N	TRP	A	349	-10.713	-6.304	-7.470	1.00	15.00	A	N
ATOM	2684	CA	TRP	A	349	-11.827	-5.453	-7.853	1.00	15.00	A	C
ATOM	2685	CB	TRP	A	349	-12.002	-5.456	-9.377	1.00	15.00	A	C
ATOM	2686	CG	TRP	A	349	-10.967	-4.670	-10.121	1.00	15.00	A	C
ATOM	2687	CD1	TRP	A	349	-9.652	-4.992	-10.284	1.00	15.00	A	C

ATOM	2688	CD2	TRP	A	349	-11.166	-3.435	-10.816	1.00	15.00	A	C
ATOM	2689	NE1	TRP	A	349	-9.015	-4.030	-11.025	1.00	15.00	A	N
ATOM	2690	CE2	TRP	A	349	-9.924	-3.063	-11.368	1.00	15.00	A	C
ATOM	2691	CE3	TRP	A	349	-12.273	-2.606	-11.023	1.00	15.00	A	C
ATOM	2692	CZ2	TRP	A	349	-9.759	-1.899	-12.112	1.00	15.00	A	C
ATOM	2693	CZ3	TRP	A	349	-12.107	-1.452	-11.762	1.00	15.00	A	C
ATOM	2694	CH2	TRP	A	349	-10.860	-1.110	-12.298	1.00	15.00	A	C
ATOM	2695	C	TRP	A	349	-13.155	-5.815	-7.200	1.00	15.00	A	C
ATOM	2696	O	TRP	A	349	-13.777	-6.820	-7.551	1.00	15.00	A	O
ATOM	2697	N	ASP	A	350	-13.572	-4.998	-6.244	1.00	15.00	A	N
ATOM	2698	CA	ASP	A	350	-14.873	-5.163	-5.609	1.00	15.00	A	C
ATOM	2699	CB	ASP	A	350	-14.819	-5.008	-4.085	1.00	15.00	A	C
ATOM	2700	CG	ASP	A	350	-16.214	-4.923	-3.470	1.00	15.00	A	C
ATOM	2701	OD1	ASP	A	350	-17.147	-5.575	-3.999	1.00	15.00	A	O
ATOM	2702	OD2	ASP	A	350	-16.386	-4.202	-2.465	1.00	15.00	A	O
ATOM	2703	C	ASP	A	350	-15.775	-4.096	-6.199	1.00	15.00	A	C
ATOM	2704	O	ASP	A	350	-15.501	-2.901	-6.071	1.00	15.00	A	O
ATOM	2705	N	LEU	A	351	-16.819	-4.519	-6.880	1.00	15.00	A	N
ATOM	2706	CA	LEU	A	351	-17.729	-3.585	-7.515	1.00	15.00	A	C
ATOM	2707	CB	LEU	A	351	-18.091	-4.068	-8.925	1.00	15.00	A	C
ATOM	2708	CG	LEU	A	351	-16.980	-4.029	-9.984	1.00	15.00	A	C
ATOM	2709	CD1	LEU	A	351	-16.025	-5.202	-9.823	1.00	15.00	A	C
ATOM	2710	CD2	LEU	A	351	-17.580	-4.019	-11.381	1.00	15.00	A	C
ATOM	2711	C	LEU	A	351	-18.990	-3.404	-6.690	1.00	15.00	A	C
ATOM	2712	O	LEU	A	351	-19.892	-2.658	-7.074	1.00	15.00	A	O
ATOM	2713	N	GLY	A	352	-19.044	-4.068	-5.547	1.00	15.00	A	N
ATOM	2714	CA	GLY	A	352	-20.216	-3.979	-4.709	1.00	15.00	A	C
ATOM	2715	C	GLY	A	352	-21.304	-4.908	-5.205	1.00	15.00	A	C
ATOM	2716	O	GLY	A	352	-21.112	-5.624	-6.192	1.00	15.00	A	O
ATOM	2717	N	LYS	A	353	-22.441	-4.914	-4.514	1.00	15.00	A	N
ATOM	2718	CA	LYS	A	353	-23.580	-5.767	-4.872	1.00	15.00	A	C
ATOM	2719	CB	LYS	A	353	-24.127	-5.435	-6.267	1.00	15.00	A	C
ATOM	2720	CG	LYS	A	353	-24.913	-4.136	-6.341	1.00	15.00	A	C
ATOM	2721	CD	LYS	A	353	-25.589	-3.970	-7.693	1.00	15.00	A	C
ATOM	2722	CE	LYS	A	353	-26.402	-2.686	-7.748	1.00	15.00	A	C
ATOM	2723	NZ	LYS	A	353	-27.119	-2.533	-9.043	1.00	15.00	A	N
ATOM	2724	C	LYS	A	353	-23.271	-7.263	-4.765	1.00	15.00	A	C
ATOM	2725	O	LYS	A	353	-24.168	-8.095	-4.929	1.00	15.00	A	O
ATOM	2726	N	GLY	A	354	-22.017	-7.601	-4.490	1.00	15.00	A	N
ATOM	2727	CA	GLY	A	354	-21.624	-8.988	-4.380	1.00	15.00	A	C
ATOM	2728	C	GLY	A	354	-20.792	-9.444	-5.564	1.00	15.00	A	C
ATOM	2729	O	GLY	A	354	-20.581	-10.640	-5.755	1.00	15.00	A	O
ATOM	2730	N	ASP	A	355	-20.330	-8.492	-6.364	1.00	15.00	A	N
ATOM	2731	CA	ASP	A	355	-19.513	-8.803	-7.534	1.00	15.00	A	C
ATOM	2732	CB	ASP	A	355	-19.966	-7.982	-8.747	1.00	15.00	A	C
ATOM	2733	CG	ASP	A	355	-19.292	-8.403	-10.039	1.00	15.00	A	C
ATOM	2734	OD1	ASP	A	355	-19.066	-9.615	-10.231	1.00	15.00	A	O
ATOM	2735	OD2	ASP	A	355	-18.997	-7.517	-10.873	1.00	15.00	A	O
ATOM	2736	C	ASP	A	355	-18.031	-8.564	-7.241	1.00	15.00	A	C
ATOM	2737	O	ASP	A	355	-17.598	-7.428	-7.030	1.00	15.00	A	O
ATOM	2738	N	PHE	A	356	-17.266	-9.651	-7.208	1.00	15.00	A	N
ATOM	2739	CA	PHE	A	356	-15.833	-9.592	-6.933	1.00	15.00	A	C
ATOM	2740	CB	PHE	A	356	-15.501	-10.379	-5.661	1.00	15.00	A	C
ATOM	2741	CG	PHE	A	356	-16.318	-9.989	-4.464	1.00	15.00	A	C
ATOM	2742	CD1	PHE	A	356	-15.923	-8.941	-3.651	1.00	15.00	A	C
ATOM	2743	CD2	PHE	A	356	-17.482	-10.672	-4.152	1.00	15.00	A	C
ATOM	2744	CE1	PHE	A	356	-16.674	-8.579	-2.548	1.00	15.00	A	C
ATOM	2745	CE2	PHE	A	356	-18.239	-10.316	-3.050	1.00	15.00	A	C
ATOM	2746	CZ	PHE	A	356	-17.835	-9.266	-2.246	1.00	15.00	A	C
ATOM	2747	C	PHE	A	356	-15.041	-10.160	-8.106	1.00	15.00	A	C
ATOM	2748	O	PHE	A	356	-15.324	-11.259	-8.580	1.00	15.00	A	O
ATOM	2749	N	ARG	A	357	-14.046	-9.412	-8.564	1.00	15.00	A	N
ATOM	2750	CA	ARG	A	357	-13.223	-9.835	-9.696	1.00	15.00	A	C
ATOM	2751	CB	ARG	A	357	-13.759	-9.202	-10.990	1.00	15.00	A	C
ATOM	2752	CG	ARG	A	357	-15.206	-9.555	-11.298	1.00	15.00	A	C
ATOM	2753	CD	ARG	A	357	-16.000	-8.332	-11.704	1.00	15.00	A	C
ATOM	2754	NE	ARG	A	357	-15.912	-8.093	-13.144	1.00	15.00	A	N
ATOM	2755	CZ	ARG	A	357	-16.908	-7.615	-13.881	1.00	15.00	A	C
ATOM	2756	NH1	ARG	A	357	-18.070	-7.316	-13.328	1.00	15.00	A	N
ATOM	2757	NH2	ARG	A	357	-16.736	-7.431	-15.180	1.00	15.00	A	N
ATOM	2758	C	ARG	A	357	-11.760	-9.448	-9.500	1.00	15.00	A	C

ATOM	2759	O	ARG	A	357	-11.440	-8.661	-8.615	1.00	15.00	A	O
ATOM	2760	N	ILE	A	358	-10.883	-10.009	-10.329	1.00	15.00	A	N
ATOM	2761	CA	ILE	A	358	-9.453	-9.710	-10.269	1.00	15.00	A	C
ATOM	2762	CB	ILE	A	358	-8.660	-10.871	-9.632	1.00	15.00	A	C
ATOM	2763	CG1	ILE	A	358	-8.810	-10.853	-8.115	1.00	15.00	A	C
ATOM	2764	CG2	ILE	A	358	-7.189	-10.843	-10.025	1.00	15.00	A	C
ATOM	2765	CD1	ILE	A	358	-8.115	-12.001	-7.417	1.00	15.00	A	C
ATOM	2766	C	ILE	A	358	-8.906	-9.425	-11.666	1.00	15.00	A	C
ATOM	2767	O	ILE	A	358	-9.286	-10.080	-12.638	1.00	15.00	A	O
ATOM	2768	N	LEU	A	359	-8.017	-8.446	-11.760	1.00	15.00	A	N
ATOM	2769	CA	LEU	A	359	-7.415	-8.081	-13.029	1.00	15.00	A	C
ATOM	2770	CB	LEU	A	359	-7.696	-6.613	-13.326	1.00	15.00	A	C
ATOM	2771	CG	LEU	A	359	-8.111	-6.274	-14.756	1.00	15.00	A	C
ATOM	2772	CD1	LEU	A	359	-8.468	-4.802	-14.858	1.00	15.00	A	C
ATOM	2773	CD2	LEU	A	359	-7.008	-6.629	-15.741	1.00	15.00	A	C
ATOM	2774	C	LEU	A	359	-5.912	-8.306	-12.972	1.00	15.00	A	C
ATOM	2775	O	LEU	A	359	-5.210	-7.664	-12.187	1.00	15.00	A	O
ATOM	2776	N	MET	A	360	-5.420	-9.221	-13.791	1.00	15.00	A	N
ATOM	2777	CA	MET	A	360	-4.001	-9.519	-13.825	1.00	15.00	A	C
ATOM	2778	CB	MET	A	360	-3.572	-10.212	-12.529	1.00	15.00	A	C
ATOM	2779	CG	MET	A	360	-2.079	-10.488	-12.433	1.00	15.00	A	C
ATOM	2780	SD	MET	A	360	-1.689	-11.807	-11.266	1.00	15.00	A	S
ATOM	2781	CE	MET	A	360	-2.565	-11.251	-9.807	1.00	15.00	A	C
ATOM	2782	C	MET	A	360	-3.670	-10.420	-14.997	1.00	15.00	A	C
ATOM	2783	O	MET	A	360	-4.300	-11.454	-15.188	1.00	15.00	A	O
ATOM	2784	N	CYS	A	361	-2.687	-10.023	-15.784	1.00	15.00	A	N
ATOM	2785	CA	CYS	A	361	-2.249	-10.826	-16.914	1.00	15.00	A	C
ATOM	2786	C	CYS	A	361	-1.244	-11.854	-16.405	1.00	15.00	A	C
ATOM	2787	O	CYS	A	361	-0.065	-11.819	-16.757	1.00	15.00	A	O
ATOM	2788	CB	CYS	A	361	-1.604	-9.934	-17.978	1.00	15.00	A	C
ATOM	2789	SG	CYS	A	361	-2.535	-8.410	-18.342	1.00	15.00	A	S
ATOM	2790	N	THR	A	362	-1.732	-12.738	-15.544	1.00	15.00	A	N
ATOM	2791	CA	THR	A	362	-0.926	-13.778	-14.918	1.00	15.00	A	C
ATOM	2792	CB	THR	A	362	-1.822	-14.672	-14.045	1.00	15.00	A	C
ATOM	2793	OG1	THR	A	362	-2.768	-13.836	-13.363	1.00	15.00	A	O
ATOM	2794	CG2	THR	A	362	-0.996	-15.426	-13.016	1.00	15.00	A	C
ATOM	2795	C	THR	A	362	-0.140	-14.638	-15.912	1.00	15.00	A	C
ATOM	2796	O	THR	A	362	-0.673	-15.108	-16.924	1.00	15.00	A	O
ATOM	2797	N	LYS	A	363	1.142	-14.815	-15.615	1.00	15.00	A	N
ATOM	2798	CA	LYS	A	363	2.035	-15.621	-16.431	1.00	15.00	A	C
ATOM	2799	CB	LYS	A	363	3.328	-14.847	-16.713	1.00	15.00	A	C
ATOM	2800	CG	LYS	A	363	3.120	-13.416	-17.174	1.00	15.00	A	C
ATOM	2801	CD	LYS	A	363	2.569	-13.379	-18.585	1.00	15.00	A	C
ATOM	2802	CE	LYS	A	363	2.120	-11.983	-18.965	1.00	15.00	A	C
ATOM	2803	NZ	LYS	A	363	1.408	-11.991	-20.264	1.00	15.00	A	N
ATOM	2804	C	LYS	A	363	2.380	-16.901	-15.679	1.00	15.00	A	C
ATOM	2805	O	LYS	A	363	1.986	-17.076	-14.526	1.00	15.00	A	O
ATOM	2806	N	VAL	A	364	3.123	-17.787	-16.323	1.00	15.00	A	N
ATOM	2807	CA	VAL	A	364	3.528	-19.038	-15.699	1.00	15.00	A	C
ATOM	2808	CB	VAL	A	364	3.647	-20.175	-16.739	1.00	15.00	A	C
ATOM	2809	CG1	VAL	A	364	3.806	-21.525	-16.054	1.00	15.00	A	C
ATOM	2810	CG2	VAL	A	364	2.432	-20.184	-17.658	1.00	15.00	A	C
ATOM	2811	C	VAL	A	364	4.863	-18.824	-14.988	1.00	15.00	A	C
ATOM	2812	O	VAL	A	364	5.903	-19.331	-15.410	1.00	15.00	A	O
ATOM	2813	N	THR	A	365	4.819	-18.036	-13.925	1.00	15.00	A	N
ATOM	2814	CA	THR	A	365	6.005	-17.707	-13.153	1.00	15.00	A	C
ATOM	2815	CB	THR	A	365	6.431	-16.259	-13.463	1.00	15.00	A	C
ATOM	2816	OG1	THR	A	365	5.335	-15.579	-14.103	1.00	15.00	A	O
ATOM	2817	CG2	THR	A	365	7.634	-16.248	-14.395	1.00	15.00	A	C
ATOM	2818	C	THR	A	365	5.732	-17.835	-11.655	1.00	15.00	A	C
ATOM	2819	O	THR	A	365	4.588	-17.694	-11.212	1.00	15.00	A	O
ATOM	2820	N	MET	A	366	6.783	-18.107	-10.881	1.00	15.00	A	N
ATOM	2821	CA	MET	A	366	6.655	-18.243	-9.431	1.00	15.00	A	C
ATOM	2822	CB	MET	A	366	7.988	-18.654	-8.797	1.00	15.00	A	C
ATOM	2823	CG	MET	A	366	7.957	-18.739	-7.277	1.00	15.00	A	C
ATOM	2824	SD	MET	A	366	9.202	-19.860	-6.613	1.00	15.00	A	S
ATOM	2825	CE	MET	A	366	8.957	-19.635	-4.853	1.00	15.00	A	C
ATOM	2826	C	MET	A	366	6.136	-16.953	-8.802	1.00	15.00	A	C
ATOM	2827	O	MET	A	366	5.293	-16.986	-7.904	1.00	15.00	A	O
ATOM	2828	N	ASP	A	367	6.637	-15.821	-9.285	1.00	15.00	A	N
ATOM	2829	CA	ASP	A	367	6.215	-14.518	-8.780	1.00	15.00	A	C

```
ATOM   2830  CB  ASP A 367       6.989 -13.384  -9.457  1.00 15.00       A    C
ATOM   2831  CG  ASP A 367       8.435 -13.310  -9.004  1.00 15.00       A    C
ATOM   2832  OD1 ASP A 367       8.672 -13.130  -7.791  1.00 15.00       A    O
ATOM   2833  OD2 ASP A 367       9.332 -13.439  -9.863  1.00 15.00       A    O
ATOM   2834  C   ASP A 367       4.714 -14.322  -8.972  1.00 15.00       A    C
ATOM   2835  O   ASP A 367       4.007 -13.925  -8.044  1.00 15.00       A    O
ATOM   2836  N   ASP A 368       4.228 -14.625 -10.173  1.00 15.00       A    N
ATOM   2837  CA  ASP A 368       2.804 -14.494 -10.486  1.00 15.00       A    C
ATOM   2838  CB  ASP A 368       2.532 -14.827 -11.952  1.00 15.00       A    C
ATOM   2839  CG  ASP A 368       2.935 -13.714 -12.891  1.00 15.00       A    C
ATOM   2840  OD1 ASP A 368       4.139 -13.388 -12.933  1.00 15.00       A    O
ATOM   2841  OD2 ASP A 368       2.053 -13.186 -13.602  1.00 15.00       A    O
ATOM   2842  C   ASP A 368       1.982 -15.412  -9.597  1.00 15.00       A    C
ATOM   2843  O   ASP A 368       0.895 -15.052  -9.143  1.00 15.00       A    O
ATOM   2844  N   PHE A 369       2.518 -16.599  -9.347  1.00 15.00       A    N
ATOM   2845  CA  PHE A 369       1.861 -17.578  -8.497  1.00 15.00       A    C
ATOM   2846  CB  PHE A 369       2.698 -18.862  -8.444  1.00 15.00       A    C
ATOM   2847  CG  PHE A 369       2.222 -19.880  -7.450  1.00 15.00       A    C
ATOM   2848  CD1 PHE A 369       1.079 -20.623  -7.689  1.00 15.00       A    C
ATOM   2849  CD2 PHE A 369       2.924 -20.095  -6.275  1.00 15.00       A    C
ATOM   2850  CE1 PHE A 369       0.643 -21.562  -6.774  1.00 15.00       A    C
ATOM   2851  CE2 PHE A 369       2.495 -21.031  -5.355  1.00 15.00       A    C
ATOM   2852  CZ  PHE A 369       1.352 -21.766  -5.605  1.00 15.00       A    C
ATOM   2853  C   PHE A 369       1.661 -17.000  -7.096  1.00 15.00       A    C
ATOM   2854  O   PHE A 369       0.614 -17.186  -6.472  1.00 15.00       A    O
ATOM   2855  N   LEU A 370       2.667 -16.277  -6.617  1.00 15.00       A    N
ATOM   2856  CA  LEU A 370       2.606 -15.656  -5.301  1.00 15.00       A    C
ATOM   2857  CB  LEU A 370       4.004 -15.249  -4.828  1.00 15.00       A    C
ATOM   2858  CG  LEU A 370       4.999 -16.390  -4.600  1.00 15.00       A    C
ATOM   2859  CD1 LEU A 370       6.354 -15.842  -4.181  1.00 15.00       A    C
ATOM   2860  CD2 LEU A 370       4.469 -17.367  -3.562  1.00 15.00       A    C
ATOM   2861  C   LEU A 370       1.682 -14.442  -5.323  1.00 15.00       A    C
ATOM   2862  O   LEU A 370       0.887 -14.241  -4.406  1.00 15.00       A    O
ATOM   2863  N   THR A 371       1.778 -13.651  -6.386  1.00 15.00       A    N
ATOM   2864  CA  THR A 371       0.951 -12.458  -6.537  1.00 15.00       A    C
ATOM   2865  CB  THR A 371       1.333 -11.666  -7.804  1.00 15.00       A    C
ATOM   2866  OG1 THR A 371       2.757 -11.504  -7.847  1.00 15.00       A    O
ATOM   2867  CG2 THR A 371       0.683 -10.290  -7.791  1.00 15.00       A    C
ATOM   2868  C   THR A 371      -0.536 -12.819  -6.571  1.00 15.00       A    C
ATOM   2869  O   THR A 371      -1.379 -12.072  -6.074  1.00 15.00       A    O
ATOM   2870  N   ALA A 372      -0.851 -13.976  -7.150  1.00 15.00       A    N
ATOM   2871  CA  ALA A 372      -2.231 -14.442  -7.225  1.00 15.00       A    C
ATOM   2872  CB  ALA A 372      -2.308 -15.752  -7.993  1.00 15.00       A    C
ATOM   2873  C   ALA A 372      -2.811 -14.608  -5.823  1.00 15.00       A    C
ATOM   2874  O   ALA A 372      -3.993 -14.353  -5.591  1.00 15.00       A    O
ATOM   2875  N   HIS A 373      -1.959 -15.018  -4.889  1.00 15.00       A    N
ATOM   2876  CA  HIS A 373      -2.367 -15.213  -3.503  1.00 15.00       A    C
ATOM   2877  CB  HIS A 373      -1.317 -16.030  -2.744  1.00 15.00       A    C
ATOM   2878  CG  HIS A 373      -1.360 -17.498  -3.035  1.00 15.00       A    C
ATOM   2879  ND1 HIS A 373      -0.711 -18.098  -4.095  1.00 15.00       A    N
ATOM   2880  CD2 HIS A 373      -1.993 -18.498  -2.374  1.00 15.00       A    C
ATOM   2881  CE1 HIS A 373      -0.967 -19.412  -4.044  1.00 15.00       A    C
ATOM   2882  NE2 HIS A 373      -1.740 -19.705  -3.016  1.00 15.00       A    N
ATOM   2883  C   HIS A 373      -2.586 -13.869  -2.816  1.00 15.00       A    C
ATOM   2884  O   HIS A 373      -3.500 -13.717  -2.008  1.00 15.00       A    O
ATOM   2885  N   HIS A 374      -1.742 -12.899  -3.155  1.00 15.00       A    N
ATOM   2886  CA  HIS A 374      -1.835 -11.556  -2.590  1.00 15.00       A    C
ATOM   2887  CB  HIS A 374      -0.662 -10.701  -3.095  1.00 15.00       A    C
ATOM   2888  CG  HIS A 374      -0.460  -9.396  -2.375  1.00 15.00       A    C
ATOM   2889  ND1 HIS A 374       0.448  -9.280  -1.348  1.00 15.00       A    N
ATOM   2890  CD2 HIS A 374      -1.038  -8.186  -2.596  1.00 15.00       A    C
ATOM   2891  CE1 HIS A 374       0.409  -8.012  -0.970  1.00 15.00       A    C
ATOM   2892  NE2 HIS A 374      -0.473  -7.315  -1.696  1.00 15.00       A    N
ATOM   2893  C   HIS A 374      -3.163 -10.918  -2.990  1.00 15.00       A    C
ATOM   2894  O   HIS A 374      -3.923 -10.449  -2.139  1.00 15.00       A    O
ATOM   2895  N   GLU A 375      -3.448 -10.946  -4.287  1.00 15.00       A    N
ATOM   2896  CA  GLU A 375      -4.678 -10.372  -4.822  1.00 15.00       A    C
ATOM   2897  CB  GLU A 375      -4.669 -10.399  -6.350  1.00 15.00       A    C
ATOM   2898  CG  GLU A 375      -3.638  -9.471  -6.967  1.00 15.00       A    C
ATOM   2899  CD  GLU A 375      -3.611  -8.117  -6.291  1.00 15.00       A    C
ATOM   2900  OE1 GLU A 375      -4.611  -7.379  -6.405  1.00 15.00       A    O
```

ATOM	2901	OE2	GLU	A	375	-2.597	-7.801	-5.643	1.00	15.00	A	O
ATOM	2902	C	GLU	A	375	-5.914	-11.080	-4.282	1.00	15.00	A	C
ATOM	2903	O	GLU	A	375	-6.894	-10.436	-3.906	1.00	15.00	A	O
ATOM	2904	N	MET	A	376	-5.870	-12.407	-4.237	1.00	15.00	A	N
ATOM	2905	CA	MET	A	376	-6.997	-13.176	-3.727	1.00	15.00	A	C
ATOM	2906	CB	MET	A	376	-6.833	-14.671	-4.004	1.00	15.00	A	C
ATOM	2907	CG	MET	A	376	-8.096	-15.481	-3.750	1.00	15.00	A	C
ATOM	2908	SD	MET	A	376	-7.967	-17.184	-4.326	1.00	15.00	A	S
ATOM	2909	CE	MET	A	376	-7.948	-16.943	-6.101	1.00	15.00	A	C
ATOM	2910	C	MET	A	376	-7.189	-12.912	-2.237	1.00	15.00	A	C
ATOM	2911	O	MET	A	376	-8.299	-13.019	-1.713	1.00	15.00	A	O
ATOM	2912	N	GLY	A	377	-6.096	-12.559	-1.563	1.00	15.00	A	N
ATOM	2913	CA	GLY	A	377	-6.156	-12.250	-0.150	1.00	15.00	A	C
ATOM	2914	C	GLY	A	377	-7.018	-11.033	0.098	1.00	15.00	A	C
ATOM	2915	O	GLY	A	377	-7.773	-10.981	1.070	1.00	15.00	A	O
ATOM	2916	N	HIS	A	378	-6.910	-10.057	-0.798	1.00	15.00	A	N
ATOM	2917	CA	HIS	A	378	-7.701	-8.839	-0.700	1.00	15.00	A	C
ATOM	2918	CB	HIS	A	378	-7.236	-7.799	-1.721	1.00	15.00	A	C
ATOM	2919	CG	HIS	A	378	-5.935	-7.146	-1.375	1.00	15.00	A	C
ATOM	2920	ND1	HIS	A	378	-5.690	-6.666	-0.110	1.00	15.00	A	N
ATOM	2921	CD2	HIS	A	378	-4.852	-6.913	-2.159	1.00	15.00	A	C
ATOM	2922	CE1	HIS	A	378	-4.472	-6.157	-0.149	1.00	15.00	A	C
ATOM	2923	NE2	HIS	A	378	-3.932	-6.280	-1.365	1.00	15.00	A	N
ATOM	2924	C	HIS	A	378	-9.171	-9.163	-0.916	1.00	15.00	A	C
ATOM	2925	O	HIS	A	378	-10.020	-8.762	-0.129	1.00	15.00	A	O
ATOM	2926	N	ILE	A	379	-9.453	-9.929	-1.970	1.00	15.00	A	N
ATOM	2927	CA	ILE	A	379	-10.823	-10.330	-2.290	1.00	15.00	A	C
ATOM	2928	CB	ILE	A	379	-10.873	-11.215	-3.559	1.00	15.00	A	C
ATOM	2929	CG1	ILE	A	379	-10.437	-10.413	-4.789	1.00	15.00	A	C
ATOM	2930	CG2	ILE	A	379	-12.261	-11.810	-3.771	1.00	15.00	A	C
ATOM	2931	CD1	ILE	A	379	-11.353	-9.254	-5.128	1.00	15.00	A	C
ATOM	2932	C	ILE	A	379	-11.453	-11.070	-1.110	1.00	15.00	A	C
ATOM	2933	O	ILE	A	379	-12.642	-10.918	-0.828	1.00	15.00	A	O
ATOM	2934	N	GLN	A	380	-10.642	-11.856	-0.410	1.00	15.00	A	N
ATOM	2935	CA	GLN	A	380	-11.114	-12.600	0.748	1.00	15.00	A	C
ATOM	2936	CB	GLN	A	380	-10.027	-13.546	1.258	1.00	15.00	A	C
ATOM	2937	CG	GLN	A	380	-10.550	-14.643	2.169	1.00	15.00	A	C
ATOM	2938	CD	GLN	A	380	-11.467	-15.617	1.452	1.00	15.00	A	C
ATOM	2939	OE1	GLN	A	380	-12.447	-16.090	2.019	1.00	15.00	A	O
ATOM	2940	NE2	GLN	A	380	-11.145	-15.937	0.207	1.00	15.00	A	N
ATOM	2941	C	GLN	A	380	-11.542	-11.633	1.851	1.00	15.00	A	C
ATOM	2942	O	GLN	A	380	-12.507	-11.881	2.574	1.00	15.00	A	O
ATOM	2943	N	TYR	A	381	-10.815	-10.527	1.960	1.00	15.00	A	N
ATOM	2944	CA	TYR	A	381	-11.114	-9.496	2.943	1.00	15.00	A	C
ATOM	2945	CB	TYR	A	381	-9.961	-8.481	2.979	1.00	15.00	A	C
ATOM	2946	CG	TYR	A	381	-9.819	-7.672	4.257	1.00	15.00	A	C
ATOM	2947	CD1	TYR	A	381	-10.853	-7.570	5.180	1.00	15.00	A	C
ATOM	2948	CD2	TYR	A	381	-8.633	-7.003	4.533	1.00	15.00	A	C
ATOM	2949	CE1	TYR	A	381	-10.708	-6.825	6.339	1.00	15.00	A	C
ATOM	2950	CE2	TYR	A	381	-8.478	-6.259	5.687	1.00	15.00	A	C
ATOM	2951	CZ	TYR	A	381	-9.517	-6.172	6.586	1.00	15.00	A	C
ATOM	2952	OH	TYR	A	381	-9.363	-5.431	7.738	1.00	15.00	A	O
ATOM	2953	C	TYR	A	381	-12.406	-8.798	2.528	1.00	15.00	A	C
ATOM	2954	O	TYR	A	381	-13.323	-8.630	3.333	1.00	15.00	A	O
ATOM	2955	N	ASP	A	382	-12.467	-8.427	1.251	1.00	15.00	A	N
ATOM	2956	CA	ASP	A	382	-13.631	-7.751	0.680	1.00	15.00	A	C
ATOM	2957	CB	ASP	A	382	-13.434	-7.502	-0.824	1.00	15.00	A	C
ATOM	2958	CG	ASP	A	382	-12.145	-6.773	-1.159	1.00	15.00	A	C
ATOM	2959	OD1	ASP	A	382	-11.701	-5.935	-0.345	1.00	15.00	A	O
ATOM	2960	OD2	ASP	A	382	-11.566	-7.050	-2.233	1.00	15.00	A	O
ATOM	2961	C	ASP	A	382	-14.898	-8.570	0.898	1.00	15.00	A	C
ATOM	2962	O	ASP	A	382	-15.915	-8.053	1.360	1.00	15.00	A	O
ATOM	2963	N	MET	A	383	-14.820	-9.858	0.580	1.00	15.00	A	N
ATOM	2964	CA	MET	A	383	-15.954	-10.763	0.734	1.00	15.00	A	C
ATOM	2965	CB	MET	A	383	-15.625	-12.143	0.160	1.00	15.00	A	C
ATOM	2966	CG	MET	A	383	-15.630	-12.200	-1.359	1.00	15.00	A	C
ATOM	2967	SD	MET	A	383	-14.981	-13.752	-2.012	1.00	15.00	A	S
ATOM	2968	CE	MET	A	383	-16.103	-14.938	-1.275	1.00	15.00	A	C
ATOM	2969	C	MET	A	383	-16.379	-10.893	2.193	1.00	15.00	A	C
ATOM	2970	O	MET	A	383	-17.561	-11.063	2.491	1.00	15.00	A	O
ATOM	2971	N	ALA	A	384	-15.414	-10.796	3.097	1.00	15.00	A	N

```
ATOM   2972  CA  ALA A 384      -15.687 -10.914   4.522  1.00 15.00       A    C
ATOM   2973  CB  ALA A 384      -14.399 -11.134   5.297  1.00 15.00       A    C
ATOM   2974  C   ALA A 384      -16.446  -9.703   5.058  1.00 15.00       A    C
ATOM   2975  O   ALA A 384      -17.315  -9.840   5.922  1.00 15.00       A    O
ATOM   2976  N   TYR A 385      -16.130  -8.518   4.547  1.00 15.00       A    N
ATOM   2977  CA  TYR A 385      -16.806  -7.308   5.000  1.00 15.00       A    C
ATOM   2978  CB  TYR A 385      -15.833  -6.155   5.307  1.00 15.00       A    C
ATOM   2979  CG  TYR A 385      -15.020  -5.612   4.143  1.00 15.00       A    C
ATOM   2980  CD1 TYR A 385      -15.631  -5.029   3.039  1.00 15.00       A    C
ATOM   2981  CD2 TYR A 385      -13.632  -5.651   4.171  1.00 15.00       A    C
ATOM   2982  CE1 TYR A 385      -14.886  -4.504   2.001  1.00 15.00       A    C
ATOM   2983  CE2 TYR A 385      -12.878  -5.133   3.134  1.00 15.00       A    C
ATOM   2984  CZ  TYR A 385      -13.511  -4.559   2.053  1.00 15.00       A    C
ATOM   2985  OH  TYR A 385      -12.768  -4.026   1.027  1.00 15.00       A    O
ATOM   2986  C   TYR A 385      -17.944  -6.885   4.072  1.00 15.00       A    C
ATOM   2987  O   TYR A 385      -18.455  -5.770   4.169  1.00 15.00       A    O
ATOM   2988  N   ALA A 386      -18.361  -7.793   3.196  1.00 15.00       A    N
ATOM   2989  CA  ALA A 386      -19.444  -7.511   2.258  1.00 15.00       A    C
ATOM   2990  CB  ALA A 386      -19.531  -8.600   1.199  1.00 15.00       A    C
ATOM   2991  C   ALA A 386      -20.779  -7.361   2.985  1.00 15.00       A    C
ATOM   2992  O   ALA A 386      -21.730  -6.791   2.452  1.00 15.00       A    O
ATOM   2993  N   ALA A 387      -20.837  -7.874   4.209  1.00 15.00       A    N
ATOM   2994  CA  ALA A 387      -22.047  -7.796   5.018  1.00 15.00       A    C
ATOM   2995  CB  ALA A 387      -22.115  -8.972   5.981  1.00 15.00       A    C
ATOM   2996  C   ALA A 387      -22.106  -6.475   5.782  1.00 15.00       A    C
ATOM   2997  O   ALA A 387      -23.099  -6.170   6.442  1.00 15.00       A    O
ATOM   2998  N   GLN A 388      -21.034  -5.700   5.689  1.00 15.00       A    N
ATOM   2999  CA  GLN A 388      -20.956  -4.411   6.361  1.00 15.00       A    C
ATOM   3000  CB  GLN A 388      -19.490  -4.056   6.645  1.00 15.00       A    C
ATOM   3001  CG  GLN A 388      -18.795  -4.943   7.669  1.00 15.00       A    C
ATOM   3002  CD  GLN A 388      -19.170  -4.599   9.097  1.00 15.00       A    C
ATOM   3003  OE1 GLN A 388      -19.615  -3.490   9.389  1.00 15.00       A    O
ATOM   3004  NE2 GLN A 388      -18.962  -5.539  10.007  1.00 15.00       A    N
ATOM   3005  C   GLN A 388      -21.566  -3.326   5.477  1.00 15.00       A    C
ATOM   3006  O   GLN A 388      -21.621  -3.479   4.253  1.00 15.00       A    O
ATOM   3007  N   PRO A 389      -22.058  -2.229   6.080  1.00 15.00       A    N
ATOM   3008  CA  PRO A 389      -22.633  -1.111   5.326  1.00 15.00       A    C
ATOM   3009  CB  PRO A 389      -23.027  -0.094   6.404  1.00 15.00       A    C
ATOM   3010  CG  PRO A 389      -22.303  -0.519   7.635  1.00 15.00       A    C
ATOM   3011  CD  PRO A 389      -22.116  -2.004   7.530  1.00 15.00       A    C
ATOM   3012  C   PRO A 389      -21.599  -0.514   4.372  1.00 15.00       A    C
ATOM   3013  O   PRO A 389      -20.396  -0.646   4.593  1.00 15.00       A    O
ATOM   3014  N   PHE A 390      -22.074   0.160   3.333  1.00 15.00       A    N
ATOM   3015  CA  PHE A 390      -21.205   0.744   2.312  1.00 15.00       A    C
ATOM   3016  CB  PHE A 390      -22.024   1.553   1.302  1.00 15.00       A    C
ATOM   3017  CG  PHE A 390      -21.268   1.891   0.049  1.00 15.00       A    C
ATOM   3018  CD1 PHE A 390      -20.649   3.122  -0.093  1.00 15.00       A    C
ATOM   3019  CD2 PHE A 390      -21.173   0.973  -0.984  1.00 15.00       A    C
ATOM   3020  CE1 PHE A 390      -19.949   3.432  -1.243  1.00 15.00       A    C
ATOM   3021  CE2 PHE A 390      -20.476   1.277  -2.137  1.00 15.00       A    C
ATOM   3022  CZ  PHE A 390      -19.862   2.508  -2.266  1.00 15.00       A    C
ATOM   3023  C   PHE A 390      -20.068   1.595   2.883  1.00 15.00       A    C
ATOM   3024  O   PHE A 390      -18.908   1.406   2.523  1.00 15.00       A    O
ATOM   3025  N   LEU A 391      -20.396   2.508   3.787  1.00 15.00       A    N
ATOM   3026  CA  LEU A 391      -19.393   3.397   4.376  1.00 15.00       A    C
ATOM   3027  CB  LEU A 391      -20.057   4.593   5.058  1.00 15.00       A    C
ATOM   3028  CG  LEU A 391      -20.612   5.668   4.122  1.00 15.00       A    C
ATOM   3029  CD1 LEU A 391      -21.539   6.608   4.874  1.00 15.00       A    C
ATOM   3030  CD2 LEU A 391      -19.479   6.444   3.466  1.00 15.00       A    C
ATOM   3031  C   LEU A 391      -18.441   2.684   5.336  1.00 15.00       A    C
ATOM   3032  O   LEU A 391      -17.475   3.278   5.816  1.00 15.00       A    O
ATOM   3033  N   LEU A 392      -18.703   1.416   5.611  1.00 15.00       A    N
ATOM   3034  CA  LEU A 392      -17.854   0.649   6.510  1.00 15.00       A    C
ATOM   3035  CB  LEU A 392      -18.635   0.171   7.737  1.00 15.00       A    C
ATOM   3036  CG  LEU A 392      -19.104   1.268   8.701  1.00 15.00       A    C
ATOM   3037  CD1 LEU A 392      -19.883   0.670   9.860  1.00 15.00       A    C
ATOM   3038  CD2 LEU A 392      -17.922   2.079   9.212  1.00 15.00       A    C
ATOM   3039  C   LEU A 392      -17.199  -0.519   5.782  1.00 15.00       A    C
ATOM   3040  O   LEU A 392      -16.562  -1.374   6.399  1.00 15.00       A    O
ATOM   3041  N   ARG A 393      -17.353  -0.541   4.462  1.00 15.00       A    N
ATOM   3042  CA  ARG A 393      -16.769  -1.593   3.642  1.00 15.00       A    C
```

ATOM	3043	CB	ARG	A	393	-17.610	-1.858	2.391	1.00	15.00	A	C
ATOM	3044	CG	ARG	A	393	-18.906	-2.604	2.652	1.00	15.00	A	C
ATOM	3045	CD	ARG	A	393	-19.614	-2.941	1.351	1.00	15.00	A	C
ATOM	3046	NE	ARG	A	393	-21.050	-3.151	1.539	1.00	15.00	A	N
ATOM	3047	CZ	ARG	A	393	-21.939	-3.121	0.544	1.00	15.00	A	C
ATOM	3048	NH1	ARG	A	393	-21.534	-2.895	-0.699	1.00	15.00	A	N
ATOM	3049	NH2	ARG	A	393	-23.232	-3.310	0.789	1.00	15.00	A	N
ATOM	3050	C	ARG	A	393	-15.349	-1.221	3.246	1.00	15.00	A	C
ATOM	3051	O	ARG	A	393	-15.121	-0.617	2.198	1.00	15.00	A	O
ATOM	3052	N	ASN	A	394	-14.407	-1.562	4.109	1.00	15.00	A	N
ATOM	3053	CA	ASN	A	394	-13.000	-1.277	3.878	1.00	15.00	A	C
ATOM	3054	CB	ASN	A	394	-12.764	0.239	3.869	1.00	15.00	A	C
ATOM	3055	CG	ASN	A	394	-11.609	0.652	2.977	1.00	15.00	A	C
ATOM	3056	OD1	ASN	A	394	-10.474	0.783	3.431	1.00	15.00	A	O
ATOM	3057	ND2	ASN	A	394	-11.892	0.859	1.701	1.00	15.00	A	N
ATOM	3058	C	ASN	A	394	-12.185	-1.918	4.990	1.00	15.00	A	C
ATOM	3059	O	ASN	A	394	-12.741	-2.628	5.827	1.00	15.00	A	O
ATOM	3060	N	GLY	A	395	-10.886	-1.672	5.002	1.00	15.00	A	N
ATOM	3061	CA	GLY	A	395	-10.045	-2.227	6.039	1.00	15.00	A	C
ATOM	3062	C	GLY	A	395	-10.027	-1.320	7.249	1.00	15.00	A	C
ATOM	3063	O	GLY	A	395	-10.493	-0.182	7.168	1.00	15.00	A	O
ATOM	3064	N	ALA	A	396	-9.499	-1.813	8.365	1.00	15.00	A	N
ATOM	3065	CA	ALA	A	396	-9.431	-1.025	9.594	1.00	15.00	A	C
ATOM	3066	CB	ALA	A	396	-8.765	-1.817	10.705	1.00	15.00	A	C
ATOM	3067	C	ALA	A	396	-8.716	0.305	9.365	1.00	15.00	A	C
ATOM	3068	O	ALA	A	396	-9.162	1.347	9.842	1.00	15.00	A	O
ATOM	3069	N	ASN	A	397	-7.612	0.259	8.628	1.00	15.00	A	N
ATOM	3070	CA	ASN	A	397	-6.847	1.459	8.316	1.00	15.00	A	C
ATOM	3071	CB	ASN	A	397	-5.904	1.875	9.460	1.00	15.00	A	C
ATOM	3072	CG	ASN	A	397	-4.812	0.871	9.786	1.00	15.00	A	C
ATOM	3073	OD1	ASN	A	397	-4.322	0.149	8.920	1.00	15.00	A	O
ATOM	3074	ND2	ASN	A	397	-4.416	0.827	11.049	1.00	15.00	A	N
ATOM	3075	C	ASN	A	397	-6.106	1.311	6.989	1.00	15.00	A	C
ATOM	3076	O	ASN	A	397	-6.097	0.227	6.390	1.00	15.00	A	O
ATOM	3077	N	GLU	A	398	-5.481	2.396	6.545	1.00	15.00	A	N
ATOM	3078	CA	GLU	A	398	-4.749	2.431	5.280	1.00	15.00	A	C
ATOM	3079	CB	GLU	A	398	-4.248	3.845	4.970	1.00	15.00	A	C
ATOM	3080	CG	GLU	A	398	-3.588	4.558	6.139	1.00	15.00	A	C
ATOM	3081	CD	GLU	A	398	-2.529	5.547	5.696	1.00	15.00	A	C
ATOM	3082	OE1	GLU	A	398	-2.763	6.282	4.709	1.00	15.00	A	O
ATOM	3083	OE2	GLU	A	398	-1.451	5.579	6.329	1.00	15.00	A	O
ATOM	3084	C	GLU	A	398	-3.606	1.412	5.167	1.00	15.00	A	C
ATOM	3085	O	GLU	A	398	-3.061	1.214	4.077	1.00	15.00	A	O
ATOM	3086	N	GLY	A	399	-3.240	0.768	6.269	1.00	15.00	A	N
ATOM	3087	CA	GLY	A	399	-2.164	-0.207	6.225	1.00	15.00	A	C
ATOM	3088	C	GLY	A	399	-2.603	-1.605	6.630	1.00	15.00	A	C
ATOM	3089	O	GLY	A	399	-1.776	-2.507	6.755	1.00	15.00	A	O
ATOM	3090	N	PHE	A	400	-3.903	-1.798	6.810	1.00	15.00	A	N
ATOM	3091	CA	PHE	A	400	-4.432	-3.095	7.222	1.00	15.00	A	C
ATOM	3092	CB	PHE	A	400	-5.766	-2.923	7.954	1.00	15.00	A	C
ATOM	3093	CG	PHE	A	400	-5.743	-3.355	9.394	1.00	15.00	A	C
ATOM	3094	CD1	PHE	A	400	-5.062	-2.612	10.345	1.00	15.00	A	C
ATOM	3095	CD2	PHE	A	400	-6.412	-4.498	9.799	1.00	15.00	A	C
ATOM	3096	CE1	PHE	A	400	-5.047	-3.000	11.672	1.00	15.00	A	C
ATOM	3097	CE2	PHE	A	400	-6.401	-4.893	11.124	1.00	15.00	A	C
ATOM	3098	CZ	PHE	A	400	-5.718	-4.143	12.062	1.00	15.00	A	C
ATOM	3099	C	PHE	A	400	-4.600	-4.059	6.049	1.00	15.00	A	C
ATOM	3100	O	PHE	A	400	-4.207	-5.222	6.133	1.00	15.00	A	O
ATOM	3101	N	HIS	A	401	-5.174	-3.566	4.955	1.00	15.00	A	N
ATOM	3102	CA	HIS	A	401	-5.425	-4.389	3.768	1.00	15.00	A	C
ATOM	3103	CB	HIS	A	401	-6.056	-3.557	2.651	1.00	15.00	A	C
ATOM	3104	CG	HIS	A	401	-7.459	-3.961	2.323	1.00	15.00	A	C
ATOM	3105	ND1	HIS	A	401	-7.803	-4.776	1.266	1.00	15.00	A	N
ATOM	3106	CD2	HIS	A	401	-8.620	-3.646	2.945	1.00	15.00	A	C
ATOM	3107	CE1	HIS	A	401	-9.136	-4.925	1.279	1.00	15.00	A	C
ATOM	3108	NE2	HIS	A	401	-9.676	-4.257	2.284	1.00	15.00	A	N
ATOM	3109	C	HIS	A	401	-4.172	-5.090	3.251	1.00	15.00	A	C
ATOM	3110	O	HIS	A	401	-4.140	-6.314	3.139	1.00	15.00	A	O
ATOM	3111	N	GLU	A	402	-3.143	-4.308	2.952	1.00	15.00	A	N
ATOM	3112	CA	GLU	A	402	-1.890	-4.844	2.426	1.00	15.00	A	C
ATOM	3113	CB	GLU	A	402	-0.918	-3.708	2.114	1.00	15.00	A	C

ATOM	3114	CG	GLU	A	402	-1.184	-3.013	0.791	1.00	15.00	A	C
ATOM	3115	CD	GLU	A	402	-0.911	-3.922	-0.389	1.00	15.00	A	C
ATOM	3116	OE1	GLU	A	402	-1.869	-4.522	-0.913	1.00	15.00	A	O
ATOM	3117	OE2	GLU	A	402	0.266	-4.050	-0.775	1.00	15.00	A	O
ATOM	3118	C	GLU	A	402	-1.246	-5.867	3.365	1.00	15.00	A	C
ATOM	3119	O	GLU	A	402	-0.546	-6.775	2.919	1.00	15.00	A	O
ATOM	3120	N	ALA	A	403	-1.503	-5.730	4.661	1.00	15.00	A	N
ATOM	3121	CA	ALA	A	403	-0.942	-6.643	5.650	1.00	15.00	A	C
ATOM	3122	CB	ALA	A	403	-1.165	-6.107	7.055	1.00	15.00	A	C
ATOM	3123	C	ALA	A	403	-1.543	-8.039	5.503	1.00	15.00	A	C
ATOM	3124	O	ALA	A	403	-0.862	-9.046	5.700	1.00	15.00	A	O
ATOM	3125	N	VAL	A	404	-2.818	-8.086	5.136	1.00	15.00	A	N
ATOM	3126	CA	VAL	A	404	-3.522	-9.350	4.951	1.00	15.00	A	C
ATOM	3127	CB	VAL	A	404	-5.053	-9.131	4.890	1.00	15.00	A	C
ATOM	3128	CG1	VAL	A	404	-5.799	-10.457	4.828	1.00	15.00	A	C
ATOM	3129	CG2	VAL	A	404	-5.522	-8.314	6.084	1.00	15.00	A	C
ATOM	3130	C	VAL	A	404	-3.050	-10.037	3.669	1.00	15.00	A	C
ATOM	3131	O	VAL	A	404	-3.161	-11.254	3.521	1.00	15.00	A	O
ATOM	3132	N	GLY	A	405	-2.497	-9.252	2.756	1.00	15.00	A	N
ATOM	3133	CA	GLY	A	405	-2.022	-9.799	1.502	1.00	15.00	A	C
ATOM	3134	C	GLY	A	405	-0.633	-10.401	1.607	1.00	15.00	A	C
ATOM	3135	O	GLY	A	405	-0.402	-11.525	1.157	1.00	15.00	A	O
ATOM	3136	N	GLU	A	406	0.284	-9.665	2.229	1.00	15.00	A	N
ATOM	3137	CA	GLU	A	406	1.668	-10.114	2.371	1.00	15.00	A	C
ATOM	3138	CB	GLU	A	406	2.554	-9.014	2.955	1.00	15.00	A	C
ATOM	3139	CG	GLU	A	406	2.843	-7.878	1.982	1.00	15.00	A	C
ATOM	3140	CD	GLU	A	406	4.329	-7.670	1.732	1.00	15.00	A	C
ATOM	3141	OE1	GLU	A	406	5.147	-8.355	2.382	1.00	15.00	A	O
ATOM	3142	OE2	GLU	A	406	4.681	-6.802	0.901	1.00	15.00	A	O
ATOM	3143	C	GLU	A	406	1.807	-11.422	3.152	1.00	15.00	A	C
ATOM	3144	O	GLU	A	406	2.666	-12.245	2.835	1.00	15.00	A	O
ATOM	3145	N	ILE	A	407	0.964	-11.626	4.164	1.00	15.00	A	N
ATOM	3146	CA	ILE	A	407	1.024	-12.855	4.958	1.00	15.00	A	C
ATOM	3147	CB	ILE	A	407	0.093	-12.829	6.188	1.00	15.00	A	C
ATOM	3148	CG1	ILE	A	407	-1.353	-12.533	5.780	1.00	15.00	A	C
ATOM	3149	CG2	ILE	A	407	0.588	-11.817	7.209	1.00	15.00	A	C
ATOM	3150	CD1	ILE	A	407	-2.348	-12.642	6.916	1.00	15.00	A	C
ATOM	3151	C	ILE	A	407	0.733	-14.091	4.104	1.00	15.00	A	C
ATOM	3152	O	ILE	A	407	1.318	-15.155	4.316	1.00	15.00	A	O
ATOM	3153	N	MET	A	408	-0.155	-13.935	3.123	1.00	15.00	A	N
ATOM	3154	CA	MET	A	408	-0.512	-15.035	2.233	1.00	15.00	A	C
ATOM	3155	CB	MET	A	408	-1.670	-14.637	1.311	1.00	15.00	A	C
ATOM	3156	CG	MET	A	408	-2.903	-14.122	2.033	1.00	15.00	A	C
ATOM	3157	SD	MET	A	408	-3.598	-15.327	3.177	1.00	15.00	A	S
ATOM	3158	CE	MET	A	408	-4.884	-14.347	3.946	1.00	15.00	A	C
ATOM	3159	C	MET	A	408	0.690	-15.427	1.391	1.00	15.00	A	C
ATOM	3160	O	MET	A	408	1.017	-16.608	1.256	1.00	15.00	A	O
ATOM	3161	N	SER	A	409	1.356	-14.418	0.846	1.00	15.00	A	N
ATOM	3162	CA	SER	A	409	2.529	-14.626	0.016	1.00	15.00	A	C
ATOM	3163	CB	SER	A	409	2.961	-13.295	-0.605	1.00	15.00	A	C
ATOM	3164	OG	SER	A	409	1.846	-12.631	-1.181	1.00	15.00	A	O
ATOM	3165	C	SER	A	409	3.671	-15.246	0.822	1.00	15.00	A	C
ATOM	3166	O	SER	A	409	4.492	-15.985	0.281	1.00	15.00	A	O
ATOM	3167	N	LEU	A	410	3.703	-14.953	2.120	1.00	15.00	A	N
ATOM	3168	CA	LEU	A	410	4.738	-15.482	3.003	1.00	15.00	A	C
ATOM	3169	CB	LEU	A	410	4.644	-14.851	4.398	1.00	15.00	A	C
ATOM	3170	CG	LEU	A	410	5.012	-13.369	4.512	1.00	15.00	A	C
ATOM	3171	CD1	LEU	A	410	4.743	-12.859	5.920	1.00	15.00	A	C
ATOM	3172	CD2	LEU	A	410	6.467	-13.143	4.129	1.00	15.00	A	C
ATOM	3173	C	LEU	A	410	4.662	-17.004	3.114	1.00	15.00	A	C
ATOM	3174	O	LEU	A	410	5.601	-17.707	2.746	1.00	15.00	A	O
ATOM	3175	N	SER	A	411	3.535	-17.507	3.606	1.00	15.00	A	N
ATOM	3176	CA	SER	A	411	3.349	-18.944	3.782	1.00	15.00	A	C
ATOM	3177	CB	SER	A	411	2.053	-19.230	4.542	1.00	15.00	A	C
ATOM	3178	OG	SER	A	411	2.126	-18.723	5.864	1.00	15.00	A	O
ATOM	3179	C	SER	A	411	3.385	-19.714	2.462	1.00	15.00	A	C
ATOM	3180	O	SER	A	411	3.917	-20.822	2.400	1.00	15.00	A	O
ATOM	3181	N	ALA	A	412	2.852	-19.112	1.406	1.00	15.00	A	N
ATOM	3182	CA	ALA	A	412	2.809	-19.758	0.095	1.00	15.00	A	C
ATOM	3183	CB	ALA	A	412	1.829	-19.039	-0.820	1.00	15.00	A	C
ATOM	3184	C	ALA	A	412	4.186	-19.852	-0.562	1.00	15.00	A	C

ATOM	3185	O	ALA	A	412	4.358	-20.558	-1.554	1.00	15.00	A	O
ATOM	3186	N	ALA	A	413	5.166	-19.148	-0.011	1.00	15.00	A	N
ATOM	3187	CA	ALA	A	413	6.513	-19.161	-0.569	1.00	15.00	A	C
ATOM	3188	CB	ALA	A	413	7.064	-17.747	-0.662	1.00	15.00	A	C
ATOM	3189	C	ALA	A	413	7.451	-20.044	0.245	1.00	15.00	A	C
ATOM	3190	O	ALA	A	413	8.651	-20.104	-0.023	1.00	15.00	A	O
ATOM	3191	N	THR	A	414	6.905	-20.739	1.230	1.00	15.00	A	N
ATOM	3192	CA	THR	A	414	7.715	-21.601	2.072	1.00	15.00	A	C
ATOM	3193	CB	THR	A	414	7.075	-21.830	3.457	1.00	15.00	A	C
ATOM	3194	OG1	THR	A	414	5.808	-22.491	3.318	1.00	15.00	A	O
ATOM	3195	CG2	THR	A	414	6.887	-20.508	4.184	1.00	15.00	A	C
ATOM	3196	C	THR	A	414	7.970	-22.944	1.397	1.00	15.00	A	C
ATOM	3197	O	THR	A	414	7.104	-23.468	0.690	1.00	15.00	A	O
ATOM	3198	N	PRO	A	415	9.172	-23.514	1.598	1.00	15.00	A	N
ATOM	3199	CA	PRO	A	415	9.539	-24.810	1.018	1.00	15.00	A	C
ATOM	3200	CB	PRO	A	415	10.909	-25.102	1.636	1.00	15.00	A	C
ATOM	3201	CG	PRO	A	415	11.454	-23.762	1.990	1.00	15.00	A	C
ATOM	3202	CD	PRO	A	415	10.270	-22.931	2.389	1.00	15.00	A	C
ATOM	3203	C	PRO	A	415	8.542	-25.895	1.411	1.00	15.00	A	C
ATOM	3204	O	PRO	A	415	8.289	-26.823	0.647	1.00	15.00	A	O
ATOM	3205	N	LYS	A	416	7.966	-25.752	2.602	1.00	15.00	A	N
ATOM	3206	CA	LYS	A	416	6.987	-26.705	3.107	1.00	15.00	A	C
ATOM	3207	CB	LYS	A	416	6.581	-26.347	4.542	1.00	15.00	A	C
ATOM	3208	CG	LYS	A	416	5.743	-27.413	5.236	1.00	15.00	A	C
ATOM	3209	CD	LYS	A	416	4.586	-26.804	6.019	1.00	15.00	A	C
ATOM	3210	CE	LYS	A	416	5.065	-26.049	7.249	1.00	15.00	A	C
ATOM	3211	NZ	LYS	A	416	3.926	-25.494	8.034	1.00	15.00	A	N
ATOM	3212	C	LYS	A	416	5.753	-26.725	2.210	1.00	15.00	A	C
ATOM	3213	O	LYS	A	416	5.344	-27.780	1.722	1.00	15.00	A	O
ATOM	3214	N	HIS	A	417	5.179	-25.548	1.982	1.00	15.00	A	N
ATOM	3215	CA	HIS	A	417	3.993	-25.417	1.144	1.00	15.00	A	C
ATOM	3216	CB	HIS	A	417	3.498	-23.964	1.162	1.00	15.00	A	C
ATOM	3217	CG	HIS	A	417	2.190	-23.737	0.462	1.00	15.00	A	C
ATOM	3218	ND1	HIS	A	417	0.966	-23.729	1.095	1.00	15.00	A	N
ATOM	3219	CD2	HIS	A	417	1.931	-23.492	-0.847	1.00	15.00	A	C
ATOM	3220	CE1	HIS	A	417	0.025	-23.489	0.173	1.00	15.00	A	C
ATOM	3221	NE2	HIS	A	417	0.560	-23.337	-1.024	1.00	15.00	A	N
ATOM	3222	C	HIS	A	417	4.298	-25.864	-0.283	1.00	15.00	A	C
ATOM	3223	O	HIS	A	417	3.527	-26.606	-0.892	1.00	15.00	A	O
ATOM	3224	N	LEU	A	418	5.445	-25.433	-0.795	1.00	15.00	A	N
ATOM	3225	CA	LEU	A	418	5.865	-25.774	-2.151	1.00	15.00	A	C
ATOM	3226	CB	LEU	A	418	7.166	-25.055	-2.505	1.00	15.00	A	C
ATOM	3227	CG	LEU	A	418	7.090	-23.527	-2.555	1.00	15.00	A	C
ATOM	3228	CD1	LEU	A	418	8.482	-22.924	-2.644	1.00	15.00	A	C
ATOM	3229	CD2	LEU	A	418	6.230	-23.070	-3.723	1.00	15.00	A	C
ATOM	3230	C	LEU	A	418	6.014	-27.282	-2.341	1.00	15.00	A	C
ATOM	3231	O	LEU	A	418	5.698	-27.818	-3.406	1.00	15.00	A	O
ATOM	3232	N	LYS	A	419	6.490	-27.964	-1.309	1.00	15.00	A	N
ATOM	3233	CA	LYS	A	419	6.666	-29.409	-1.372	1.00	15.00	A	C
ATOM	3234	CB	LYS	A	419	7.613	-29.890	-0.268	1.00	15.00	A	C
ATOM	3235	CG	LYS	A	419	9.080	-29.596	-0.539	1.00	15.00	A	C
ATOM	3236	CD	LYS	A	419	9.933	-29.831	0.697	1.00	15.00	A	C
ATOM	3237	CE	LYS	A	419	11.402	-29.566	0.407	1.00	15.00	A	C
ATOM	3238	NZ	LYS	A	419	12.261	-29.794	1.599	1.00	15.00	A	N
ATOM	3239	C	LYS	A	419	5.322	-30.119	-1.261	1.00	15.00	A	C
ATOM	3240	O	LYS	A	419	5.110	-31.168	-1.865	1.00	15.00	A	O
ATOM	3241	N	SER	A	420	4.413	-29.525	-0.500	1.00	15.00	A	N
ATOM	3242	CA	SER	A	420	3.090	-30.095	-0.298	1.00	15.00	A	C
ATOM	3243	CB	SER	A	420	2.409	-29.449	0.912	1.00	15.00	A	C
ATOM	3244	OG	SER	A	420	1.211	-30.126	1.250	1.00	15.00	A	O
ATOM	3245	C	SER	A	420	2.212	-29.957	-1.542	1.00	15.00	A	C
ATOM	3246	O	SER	A	420	1.358	-30.804	-1.801	1.00	15.00	A	O
ATOM	3247	N	ILE	A	421	2.416	-28.888	-2.307	1.00	15.00	A	N
ATOM	3248	CA	ILE	A	421	1.625	-28.662	-3.516	1.00	15.00	A	C
ATOM	3249	CB	ILE	A	421	1.413	-27.163	-3.819	1.00	15.00	A	C
ATOM	3250	CG1	ILE	A	421	2.754	-26.436	-3.957	1.00	15.00	A	C
ATOM	3251	CG2	ILE	A	421	0.552	-26.523	-2.739	1.00	15.00	A	C
ATOM	3252	CD1	ILE	A	421	2.628	-24.944	-4.175	1.00	15.00	A	C
ATOM	3253	C	ILE	A	421	2.218	-29.378	-4.728	1.00	15.00	A	C
ATOM	3254	O	ILE	A	421	1.645	-29.355	-5.817	1.00	15.00	A	O
ATOM	3255	N	GLY	A	422	3.369	-30.010	-4.529	1.00	15.00	A	N

ATOM	3256	CA	GLY	A	422	4.013	-30.745	-5.601	1.00	15.00	A	C
ATOM	3257	C	GLY	A	422	4.777	-29.865	-6.571	1.00	15.00	A	C
ATOM	3258	O	GLY	A	422	5.004	-30.251	-7.716	1.00	15.00	A	O
ATOM	3259	N	LEU	A	423	5.178	-28.685	-6.124	1.00	15.00	A	N
ATOM	3260	CA	LEU	A	423	5.927	-27.774	-6.979	1.00	15.00	A	C
ATOM	3261	CB	LEU	A	423	5.438	-26.332	-6.820	1.00	15.00	A	C
ATOM	3262	CG	LEU	A	423	4.253	-25.928	-7.700	1.00	15.00	A	C
ATOM	3263	CD1	LEU	A	423	3.922	-24.456	-7.510	1.00	15.00	A	C
ATOM	3264	CD2	LEU	A	423	4.545	-26.229	-9.162	1.00	15.00	A	C
ATOM	3265	C	LEU	A	423	7.421	-27.870	-6.702	1.00	15.00	A	C
ATOM	3266	O	LEU	A	423	8.246	-27.602	-7.573	1.00	15.00	A	O
ATOM	3267	N	LEU	A	424	7.760	-28.262	-5.485	1.00	15.00	A	N
ATOM	3268	CA	LEU	A	424	9.148	-28.400	-5.085	1.00	15.00	A	C
ATOM	3269	CB	LEU	A	424	9.451	-27.449	-3.923	1.00	15.00	A	C
ATOM	3270	CG	LEU	A	424	10.914	-27.327	-3.495	1.00	15.00	A	C
ATOM	3271	CD1	LEU	A	424	11.755	-26.735	-4.617	1.00	15.00	A	C
ATOM	3272	CD2	LEU	A	424	11.029	-26.483	-2.235	1.00	15.00	A	C
ATOM	3273	C	LEU	A	424	9.422	-29.836	-4.668	1.00	15.00	A	C
ATOM	3274	O	LEU	A	424	8.650	-30.422	-3.908	1.00	15.00	A	O
ATOM	3275	N	SER	A	425	10.508	-30.404	-5.178	1.00	15.00	A	N
ATOM	3276	CA	SER	A	425	10.875	-31.772	-4.847	1.00	15.00	A	C
ATOM	3277	CB	SER	A	425	12.102	-32.214	-5.650	1.00	15.00	A	C
ATOM	3278	OG	SER	A	425	11.910	-33.507	-6.197	1.00	15.00	A	O
ATOM	3279	C	SER	A	425	11.134	-31.903	-3.348	1.00	15.00	A	C
ATOM	3280	O	SER	A	425	11.778	-31.042	-2.742	1.00	15.00	A	O
ATOM	3281	N	PRO	A	426	10.614	-32.972	-2.729	1.00	15.00	A	N
ATOM	3282	CA	PRO	A	426	10.780	-33.223	-1.291	1.00	15.00	A	C
ATOM	3283	CB	PRO	A	426	10.052	-34.557	-1.076	1.00	15.00	A	C
ATOM	3284	CG	PRO	A	426	9.114	-34.667	-2.228	1.00	15.00	A	C
ATOM	3285	CD	PRO	A	426	9.817	-34.022	-3.384	1.00	15.00	A	C
ATOM	3286	C	PRO	A	426	12.249	-33.364	-0.906	1.00	15.00	A	C
ATOM	3287	O	PRO	A	426	12.647	-33.045	0.215	1.00	15.00	A	O
ATOM	3288	N	ASP	A	427	13.042	-33.812	-1.864	1.00	15.00	A	N
ATOM	3289	CA	ASP	A	427	14.471	-34.029	-1.688	1.00	15.00	A	C
ATOM	3290	CB	ASP	A	427	15.005	-34.858	-2.864	1.00	15.00	A	C
ATOM	3291	CG	ASP	A	427	13.899	-35.610	-3.591	1.00	15.00	A	C
ATOM	3292	OD1	ASP	A	427	13.907	-36.856	-3.564	1.00	15.00	A	O
ATOM	3293	OD2	ASP	A	427	13.004	-34.943	-4.174	1.00	15.00	A	O
ATOM	3294	C	ASP	A	427	15.236	-32.713	-1.575	1.00	15.00	A	C
ATOM	3295	O	ASP	A	427	16.431	-32.708	-1.276	1.00	15.00	A	O
ATOM	3296	N	PHE	A	428	14.553	-31.598	-1.834	1.00	15.00	A	N
ATOM	3297	CA	PHE	A	428	15.174	-30.284	-1.727	1.00	15.00	A	C
ATOM	3298	CB	PHE	A	428	14.202	-29.182	-2.161	1.00	15.00	A	C
ATOM	3299	CG	PHE	A	428	14.816	-27.810	-2.205	1.00	15.00	A	C
ATOM	3300	CD1	PHE	A	428	14.611	-26.907	-1.173	1.00	15.00	A	C
ATOM	3301	CD2	PHE	A	428	15.600	-27.424	-3.279	1.00	15.00	A	C
ATOM	3302	CE1	PHE	A	428	15.177	-25.646	-1.212	1.00	15.00	A	C
ATOM	3303	CE2	PHE	A	428	16.168	-26.165	-3.325	1.00	15.00	A	C
ATOM	3304	CZ	PHE	A	428	15.956	-25.275	-2.290	1.00	15.00	A	C
ATOM	3305	C	PHE	A	428	15.629	-30.047	-0.291	1.00	15.00	A	C
ATOM	3306	O	PHE	A	428	14.805	-29.923	0.619	1.00	15.00	A	O
ATOM	3307	N	GLN	A	429	16.937	-30.001	-0.101	1.00	15.00	A	N
ATOM	3308	CA	GLN	A	429	17.520	-29.801	1.215	1.00	15.00	A	C
ATOM	3309	CB	GLN	A	429	18.991	-30.237	1.204	1.00	15.00	A	C
ATOM	3310	CG	GLN	A	429	19.709	-30.106	2.541	1.00	15.00	A	C
ATOM	3311	CD	GLN	A	429	19.189	-31.067	3.596	1.00	15.00	A	C
ATOM	3312	OE1	GLN	A	429	18.009	-31.410	3.625	1.00	15.00	A	O
ATOM	3313	NE2	GLN	A	429	20.074	-31.508	4.475	1.00	15.00	A	N
ATOM	3314	C	GLN	A	429	17.394	-28.353	1.679	1.00	15.00	A	C
ATOM	3315	O	GLN	A	429	17.596	-27.421	0.901	1.00	15.00	A	O
ATOM	3316	N	GLU	A	430	17.048	-28.184	2.948	1.00	15.00	A	N
ATOM	3317	CA	GLU	A	430	16.906	-26.867	3.553	1.00	15.00	A	C
ATOM	3318	CB	GLU	A	430	15.513	-26.727	4.189	1.00	15.00	A	C
ATOM	3319	CG	GLU	A	430	14.365	-26.820	3.189	1.00	15.00	A	C
ATOM	3320	CD	GLU	A	430	13.004	-27.031	3.834	1.00	15.00	A	C
ATOM	3321	OE1	GLU	A	430	12.616	-26.227	4.702	1.00	15.00	A	O
ATOM	3322	OE2	GLU	A	430	12.306	-27.991	3.447	1.00	15.00	A	O
ATOM	3323	C	GLU	A	430	17.992	-26.695	4.612	1.00	15.00	A	C
ATOM	3324	O	GLU	A	430	17.926	-27.305	5.679	1.00	15.00	A	O
ATOM	3325	N	ASP	A	431	19.007	-25.898	4.307	1.00	15.00	A	N
ATOM	3326	CA	ASP	A	431	20.103	-25.682	5.243	1.00	15.00	A	C

ATOM	3327	CB	ASP	A	431	21.434	-26.219	4.693	1.00	15.00	A	C
ATOM	3328	CG	ASP	A	431	21.873	-25.562	3.395	1.00	15.00	A	C
ATOM	3329	OD1	ASP	A	431	22.067	-24.330	3.375	1.00	15.00	A	O
ATOM	3330	OD2	ASP	A	431	22.052	-26.283	2.391	1.00	15.00	A	O
ATOM	3331	C	ASP	A	431	20.237	-24.221	5.654	1.00	15.00	A	C
ATOM	3332	O	ASP	A	431	19.559	-23.343	5.113	1.00	15.00	A	O
ATOM	3333	N	ASN	A	432	21.127	-23.984	6.616	1.00	15.00	A	N
ATOM	3334	CA	ASN	A	432	21.395	-22.647	7.146	1.00	15.00	A	C
ATOM	3335	CB	ASN	A	432	22.605	-22.686	8.089	1.00	15.00	A	C
ATOM	3336	CG	ASN	A	432	22.256	-23.032	9.524	1.00	15.00	A	C
ATOM	3337	OD1	ASN	A	432	21.280	-23.726	9.794	1.00	15.00	A	O
ATOM	3338	ND2	ASN	A	432	23.070	-22.560	10.458	1.00	15.00	A	N
ATOM	3339	C	ASN	A	432	21.671	-21.635	6.041	1.00	15.00	A	C
ATOM	3340	O	ASN	A	432	21.108	-20.542	6.038	1.00	15.00	A	O
ATOM	3341	N	GLU	A	433	22.528	-22.011	5.100	1.00	15.00	A	N
ATOM	3342	CA	GLU	A	433	22.903	-21.128	4.002	1.00	15.00	A	C
ATOM	3343	CB	GLU	A	433	23.937	-21.798	3.099	1.00	15.00	A	C
ATOM	3344	CG	GLU	A	433	25.296	-22.015	3.744	1.00	15.00	A	C
ATOM	3345	CD	GLU	A	433	26.240	-22.773	2.833	1.00	15.00	A	C
ATOM	3346	OE1	GLU	A	433	26.787	-22.155	1.895	1.00	15.00	A	O
ATOM	3347	OE2	GLU	A	433	26.403	-23.998	3.031	1.00	15.00	A	O
ATOM	3348	C	GLU	A	433	21.691	-20.705	3.181	1.00	15.00	A	C
ATOM	3349	O	GLU	A	433	21.471	-19.513	2.952	1.00	15.00	A	O
ATOM	3350	N	THR	A	434	20.902	-21.681	2.752	1.00	15.00	A	N
ATOM	3351	CA	THR	A	434	19.720	-21.406	1.950	1.00	15.00	A	C
ATOM	3352	CB	THR	A	434	19.029	-22.703	1.495	1.00	15.00	A	C
ATOM	3353	OG1	THR	A	434	19.233	-23.726	2.479	1.00	15.00	A	O
ATOM	3354	CG2	THR	A	434	19.606	-23.167	0.166	1.00	15.00	A	C
ATOM	3355	C	THR	A	434	18.722	-20.501	2.675	1.00	15.00	A	C
ATOM	3356	O	THR	A	434	18.113	-19.620	2.062	1.00	15.00	A	O
ATOM	3357	N	GLU	A	435	18.570	-20.701	3.978	1.00	15.00	A	N
ATOM	3358	CA	GLU	A	435	17.643	-19.895	4.760	1.00	15.00	A	C
ATOM	3359	CB	GLU	A	435	17.295	-20.579	6.078	1.00	15.00	A	C
ATOM	3360	CG	GLU	A	435	16.666	-21.948	5.876	1.00	15.00	A	C
ATOM	3361	CD	GLU	A	435	15.864	-22.422	7.064	1.00	15.00	A	C
ATOM	3362	OE1	GLU	A	435	15.488	-21.587	7.908	1.00	15.00	A	O
ATOM	3363	OE2	GLU	A	435	15.584	-23.634	7.138	1.00	15.00	A	O
ATOM	3364	C	GLU	A	435	18.167	-18.476	4.969	1.00	15.00	A	C
ATOM	3365	O	GLU	A	435	17.401	-17.511	4.927	1.00	15.00	A	O
ATOM	3366	N	ILE	A	436	19.474	-18.349	5.179	1.00	15.00	A	N
ATOM	3367	CA	ILE	A	436	20.092	-17.039	5.364	1.00	15.00	A	C
ATOM	3368	CB	ILE	A	436	21.587	-17.154	5.752	1.00	15.00	A	C
ATOM	3369	CG1	ILE	A	436	21.732	-17.705	7.176	1.00	15.00	A	C
ATOM	3370	CG2	ILE	A	436	22.303	-15.815	5.619	1.00	15.00	A	C
ATOM	3371	CD1	ILE	A	436	21.033	-16.877	8.236	1.00	15.00	A	C
ATOM	3372	C	ILE	A	436	19.937	-16.196	4.097	1.00	15.00	A	C
ATOM	3373	O	ILE	A	436	19.625	-15.006	4.166	1.00	15.00	A	O
ATOM	3374	N	ASN	A	437	20.130	-16.829	2.939	1.00	15.00	A	N
ATOM	3375	CA	ASN	A	437	19.999	-16.142	1.653	1.00	15.00	A	C
ATOM	3376	CB	ASN	A	437	20.279	-17.101	0.491	1.00	15.00	A	C
ATOM	3377	CG	ASN	A	437	21.750	-17.244	0.151	1.00	15.00	A	C
ATOM	3378	OD1	ASN	A	437	22.600	-16.501	0.642	1.00	15.00	A	O
ATOM	3379	ND2	ASN	A	437	22.057	-18.200	-0.709	1.00	15.00	A	N
ATOM	3380	C	ASN	A	437	18.595	-15.577	1.497	1.00	15.00	A	C
ATOM	3381	O	ASN	A	437	18.407	-14.454	1.020	1.00	15.00	A	O
ATOM	3382	N	PHE	A	438	17.613	-16.368	1.910	1.00	15.00	A	N
ATOM	3383	CA	PHE	A	438	16.217	-15.973	1.832	1.00	15.00	A	C
ATOM	3384	CB	PHE	A	438	15.321	-17.145	2.250	1.00	15.00	A	C
ATOM	3385	CG	PHE	A	438	13.860	-16.810	2.337	1.00	15.00	A	C
ATOM	3386	CD1	PHE	A	438	13.129	-16.531	1.195	1.00	15.00	A	C
ATOM	3387	CD2	PHE	A	438	13.217	-16.777	3.564	1.00	15.00	A	C
ATOM	3388	CE1	PHE	A	438	11.784	-16.225	1.273	1.00	15.00	A	C
ATOM	3389	CE2	PHE	A	438	11.872	-16.472	3.651	1.00	15.00	A	C
ATOM	3390	CZ	PHE	A	438	11.155	-16.195	2.503	1.00	15.00	A	C
ATOM	3391	C	PHE	A	438	15.951	-14.756	2.712	1.00	15.00	A	C
ATOM	3392	O	PHE	A	438	15.392	-13.757	2.255	1.00	15.00	A	O
ATOM	3393	N	LEU	A	439	16.375	-14.839	3.968	1.00	15.00	A	N
ATOM	3394	CA	LEU	A	439	16.183	-13.750	4.919	1.00	15.00	A	C
ATOM	3395	CB	LEU	A	439	16.674	-14.154	6.310	1.00	15.00	A	C
ATOM	3396	CG	LEU	A	439	15.806	-15.165	7.062	1.00	15.00	A	C
ATOM	3397	CD1	LEU	A	439	16.487	-15.596	8.350	1.00	15.00	A	C

```
ATOM   3398  CD2 LEU A 439      14.433 -14.578   7.352  1.00 15.00       A    C
ATOM   3399  C   LEU A 439      16.878 -12.472   4.459  1.00 15.00       A    C
ATOM   3400  O   LEU A 439      16.368 -11.371   4.666  1.00 15.00       A    O
ATOM   3401  N   LEU A 440      18.038 -12.627   3.830  1.00 15.00       A    N
ATOM   3402  CA  LEU A 440      18.804 -11.489   3.332  1.00 15.00       A    C
ATOM   3403  CB  LEU A 440      20.132 -11.971   2.728  1.00 15.00       A    C
ATOM   3404  CG  LEU A 440      21.305 -10.980   2.729  1.00 15.00       A    C
ATOM   3405  CD1 LEU A 440      21.231 -10.035   1.540  1.00 15.00       A    C
ATOM   3406  CD2 LEU A 440      21.371 -10.207   4.037  1.00 15.00       A    C
ATOM   3407  C   LEU A 440      17.992 -10.715   2.296  1.00 15.00       A    C
ATOM   3408  O   LEU A 440      17.858  -9.493   2.384  1.00 15.00       A    O
ATOM   3409  N   LYS A 441      17.428 -11.440   1.336  1.00 15.00       A    N
ATOM   3410  CA  LYS A 441      16.621 -10.832   0.283  1.00 15.00       A    C
ATOM   3411  CB  LYS A 441      16.207 -11.896  -0.739  1.00 15.00       A    C
ATOM   3412  CG  LYS A 441      15.796 -11.344  -2.096  1.00 15.00       A    C
ATOM   3413  CD  LYS A 441      15.587 -12.466  -3.103  1.00 15.00       A    C
ATOM   3414  CE  LYS A 441      15.216 -11.925  -4.475  1.00 15.00       A    C
ATOM   3415  NZ  LYS A 441      15.004 -13.018  -5.466  1.00 15.00       A    N
ATOM   3416  C   LYS A 441      15.386 -10.159   0.880  1.00 15.00       A    C
ATOM   3417  O   LYS A 441      14.948  -9.101   0.416  1.00 15.00       A    O
ATOM   3418  N   GLN A 442      14.837 -10.780   1.918  1.00 15.00       A    N
ATOM   3419  CA  GLN A 442      13.663 -10.252   2.601  1.00 15.00       A    C
ATOM   3420  CB  GLN A 442      13.134 -11.271   3.616  1.00 15.00       A    C
ATOM   3421  CG  GLN A 442      12.513 -12.509   2.992  1.00 15.00       A    C
ATOM   3422  CD  GLN A 442      11.185 -12.217   2.325  1.00 15.00       A    C
ATOM   3423  OE1 GLN A 442      11.134 -11.830   1.160  1.00 15.00       A    O
ATOM   3424  NE2 GLN A 442      10.099 -12.408   3.058  1.00 15.00       A    N
ATOM   3425  C   GLN A 442      13.988  -8.940   3.309  1.00 15.00       A    C
ATOM   3426  O   GLN A 442      13.242  -7.967   3.206  1.00 15.00       A    O
ATOM   3427  N   ALA A 443      15.115  -8.917   4.014  1.00 15.00       A    N
ATOM   3428  CA  ALA A 443      15.544  -7.732   4.752  1.00 15.00       A    C
ATOM   3429  CB  ALA A 443      16.790  -8.034   5.567  1.00 15.00       A    C
ATOM   3430  C   ALA A 443      15.785  -6.542   3.829  1.00 15.00       A    C
ATOM   3431  O   ALA A 443      15.448  -5.409   4.167  1.00 15.00       A    O
ATOM   3432  N   LEU A 444      16.357  -6.809   2.662  1.00 15.00       A    N
ATOM   3433  CA  LEU A 444      16.647  -5.760   1.687  1.00 15.00       A    C
ATOM   3434  CB  LEU A 444      17.394  -6.346   0.488  1.00 15.00       A    C
ATOM   3435  CG  LEU A 444      18.762  -6.966   0.772  1.00 15.00       A    C
ATOM   3436  CD1 LEU A 444      19.311  -7.630  -0.481  1.00 15.00       A    C
ATOM   3437  CD2 LEU A 444      19.733  -5.917   1.293  1.00 15.00       A    C
ATOM   3438  C   LEU A 444      15.371  -5.077   1.205  1.00 15.00       A    C
ATOM   3439  O   LEU A 444      15.377  -3.897   0.852  1.00 15.00       A    O
ATOM   3440  N   THR A 445      14.280  -5.822   1.199  1.00 15.00       A    N
ATOM   3441  CA  THR A 445      13.002  -5.301   0.747  1.00 15.00       A    C
ATOM   3442  CB  THR A 445      12.202  -6.410   0.034  1.00 15.00       A    C
ATOM   3443  OG1 THR A 445      13.088  -7.165  -0.808  1.00 15.00       A    O
ATOM   3444  CG2 THR A 445      11.086  -5.817  -0.811  1.00 15.00       A    C
ATOM   3445  C   THR A 445      12.171  -4.753   1.907  1.00 15.00       A    C
ATOM   3446  O   THR A 445      11.668  -3.633   1.853  1.00 15.00       A    O
ATOM   3447  N   ILE A 446      12.056  -5.541   2.962  1.00 15.00       A    N
ATOM   3448  CA  ILE A 446      11.257  -5.168   4.121  1.00 15.00       A    C
ATOM   3449  CB  ILE A 446      10.840  -6.422   4.918  1.00 15.00       A    C
ATOM   3450  CG1 ILE A 446       9.953  -7.322   4.051  1.00 15.00       A    C
ATOM   3451  CG2 ILE A 446      10.122  -6.040   6.202  1.00 15.00       A    C
ATOM   3452  CD1 ILE A 446       9.896  -8.762   4.512  1.00 15.00       A    C
ATOM   3453  C   ILE A 446      11.962  -4.168   5.040  1.00 15.00       A    C
ATOM   3454  O   ILE A 446      11.516  -3.030   5.188  1.00 15.00       A    O
ATOM   3455  N   VAL A 447      13.070  -4.589   5.639  1.00 15.00       A    N
ATOM   3456  CA  VAL A 447      13.812  -3.742   6.573  1.00 15.00       A    C
ATOM   3457  CB  VAL A 447      14.963  -4.513   7.257  1.00 15.00       A    C
ATOM   3458  CG1 VAL A 447      15.649  -3.649   8.307  1.00 15.00       A    C
ATOM   3459  CG2 VAL A 447      14.444  -5.798   7.881  1.00 15.00       A    C
ATOM   3460  C   VAL A 447      14.355  -2.474   5.913  1.00 15.00       A    C
ATOM   3461  O   VAL A 447      14.351  -1.406   6.521  1.00 15.00       A    O
ATOM   3462  N   GLY A 448      14.799  -2.591   4.669  1.00 15.00       A    N
ATOM   3463  CA  GLY A 448      15.353  -1.446   3.962  1.00 15.00       A    C
ATOM   3464  C   GLY A 448      14.346  -0.339   3.691  1.00 15.00       A    C
ATOM   3465  O   GLY A 448      14.712   0.835   3.620  1.00 15.00       A    O
ATOM   3466  N   THR A 449      13.079  -0.699   3.562  1.00 15.00       A    N
ATOM   3467  CA  THR A 449      12.038   0.279   3.273  1.00 15.00       A    C
ATOM   3468  CB  THR A 449      10.873  -0.364   2.481  1.00 15.00       A    C
```

ATOM	3469	OG1	THR	A	449	10.514	0.478	1.381	1.00	15.00	A	O
ATOM	3470	CG2	THR	A	449	9.651	-0.630	3.352	1.00	15.00	A	C
ATOM	3471	C	THR	A	449	11.531	1.000	4.531	1.00	15.00	A	C
ATOM	3472	O	THR	A	449	10.945	2.085	4.443	1.00	15.00	A	O
ATOM	3473	N	LEU	A	450	11.785	0.413	5.695	1.00	15.00	A	N
ATOM	3474	CA	LEU	A	450	11.338	0.984	6.966	1.00	15.00	A	C
ATOM	3475	CB	LEU	A	450	11.590	0.024	8.133	1.00	15.00	A	C
ATOM	3476	CG	LEU	A	450	10.855	-1.318	8.063	1.00	15.00	A	C
ATOM	3477	CD1	LEU	A	450	11.355	-2.264	9.142	1.00	15.00	A	C
ATOM	3478	CD2	LEU	A	450	9.352	-1.112	8.184	1.00	15.00	A	C
ATOM	3479	C	LEU	A	450	11.904	2.387	7.247	1.00	15.00	A	C
ATOM	3480	O	LEU	A	450	11.134	3.328	7.459	1.00	15.00	A	O
ATOM	3481	N	PRO	A	451	13.245	2.565	7.254	1.00	15.00	A	N
ATOM	3482	CA	PRO	A	451	13.861	3.873	7.519	1.00	15.00	A	C
ATOM	3483	CB	PRO	A	451	15.367	3.590	7.473	1.00	15.00	A	C
ATOM	3484	CG	PRO	A	451	15.476	2.122	7.679	1.00	15.00	A	C
ATOM	3485	CD	PRO	A	451	14.267	1.533	7.022	1.00	15.00	A	C
ATOM	3486	C	PRO	A	451	13.485	4.905	6.464	1.00	15.00	A	C
ATOM	3487	O	PRO	A	451	13.250	6.072	6.783	1.00	15.00	A	O
ATOM	3488	N	PHE	A	452	13.423	4.468	5.210	1.00	15.00	A	N
ATOM	3489	CA	PHE	A	452	13.066	5.354	4.111	1.00	15.00	A	C
ATOM	3490	CB	PHE	A	452	13.157	4.621	2.769	1.00	15.00	A	C
ATOM	3491	CG	PHE	A	452	12.907	5.501	1.574	1.00	15.00	A	C
ATOM	3492	CD1	PHE	A	452	11.640	5.603	1.022	1.00	15.00	A	C
ATOM	3493	CD2	PHE	A	452	13.938	6.227	1.004	1.00	15.00	A	C
ATOM	3494	CE1	PHE	A	452	11.406	6.410	-0.075	1.00	15.00	A	C
ATOM	3495	CE2	PHE	A	452	13.712	7.036	-0.093	1.00	15.00	A	C
ATOM	3496	CZ	PHE	A	452	12.444	7.127	-0.634	1.00	15.00	A	C
ATOM	3497	C	PHE	A	452	11.661	5.909	4.312	1.00	15.00	A	C
ATOM	3498	O	PHE	A	452	11.433	7.109	4.162	1.00	15.00	A	O
ATOM	3499	N	THR	A	453	10.735	5.028	4.669	1.00	15.00	A	N
ATOM	3500	CA	THR	A	453	9.351	5.415	4.893	1.00	15.00	A	C
ATOM	3501	CB	THR	A	453	8.467	4.178	5.158	1.00	15.00	A	C
ATOM	3502	OG1	THR	A	453	8.513	3.305	4.021	1.00	15.00	A	O
ATOM	3503	CG2	THR	A	453	7.027	4.584	5.424	1.00	15.00	A	C
ATOM	3504	C	THR	A	453	9.231	6.401	6.055	1.00	15.00	A	C
ATOM	3505	O	THR	A	453	8.626	7.466	5.916	1.00	15.00	A	O
ATOM	3506	N	TYR	A	454	9.828	6.050	7.190	1.00	15.00	A	N
ATOM	3507	CA	TYR	A	454	9.782	6.903	8.371	1.00	15.00	A	C
ATOM	3508	CB	TYR	A	454	10.483	6.233	9.560	1.00	15.00	A	C
ATOM	3509	CG	TYR	A	454	10.843	7.195	10.671	1.00	15.00	A	C
ATOM	3510	CD1	TYR	A	454	9.857	7.803	11.437	1.00	15.00	A	C
ATOM	3511	CD2	TYR	A	454	12.169	7.511	10.938	1.00	15.00	A	C
ATOM	3512	CE1	TYR	A	454	10.181	8.701	12.436	1.00	15.00	A	C
ATOM	3513	CE2	TYR	A	454	12.502	8.403	11.938	1.00	15.00	A	C
ATOM	3514	CZ	TYR	A	454	11.506	8.996	12.682	1.00	15.00	A	C
ATOM	3515	OH	TYR	A	454	11.837	9.895	13.669	1.00	15.00	A	O
ATOM	3516	C	TYR	A	454	10.391	8.279	8.100	1.00	15.00	A	C
ATOM	3517	O	TYR	A	454	9.796	9.304	8.429	1.00	15.00	A	O
ATOM	3518	N	MET	A	455	11.570	8.291	7.493	1.00	15.00	A	N
ATOM	3519	CA	MET	A	455	12.271	9.536	7.197	1.00	15.00	A	C
ATOM	3520	CB	MET	A	455	13.672	9.239	6.653	1.00	15.00	A	C
ATOM	3521	CG	MET	A	455	14.488	10.464	6.259	1.00	15.00	A	C
ATOM	3522	SD	MET	A	455	14.333	10.890	4.510	1.00	15.00	A	S
ATOM	3523	CE	MET	A	455	14.219	9.270	3.755	1.00	15.00	A	C
ATOM	3524	C	MET	A	455	11.487	10.420	6.230	1.00	15.00	A	C
ATOM	3525	O	MET	A	455	11.339	11.622	6.463	1.00	15.00	A	O
ATOM	3526	N	LEU	A	456	10.970	9.818	5.164	1.00	15.00	A	N
ATOM	3527	CA	LEU	A	456	10.217	10.560	4.161	1.00	15.00	A	C
ATOM	3528	CB	LEU	A	456	9.861	9.666	2.970	1.00	15.00	A	C
ATOM	3529	CG	LEU	A	456	9.323	10.380	1.727	1.00	15.00	A	C
ATOM	3530	CD1	LEU	A	456	10.071	9.926	0.484	1.00	15.00	A	C
ATOM	3531	CD2	LEU	A	456	7.830	10.138	1.570	1.00	15.00	A	C
ATOM	3532	C	LEU	A	456	8.961	11.189	4.754	1.00	15.00	A	C
ATOM	3533	O	LEU	A	456	8.691	12.372	4.539	1.00	15.00	A	O
ATOM	3534	N	GLU	A	457	8.205	10.403	5.510	1.00	15.00	A	N
ATOM	3535	CA	GLU	A	457	6.978	10.898	6.119	1.00	15.00	A	C
ATOM	3536	CB	GLU	A	457	6.133	9.756	6.685	1.00	15.00	A	C
ATOM	3537	CG	GLU	A	457	4.644	10.065	6.734	1.00	15.00	A	C
ATOM	3538	CD	GLU	A	457	3.990	10.058	5.363	1.00	15.00	A	C
ATOM	3539	OE1	GLU	A	457	2.939	9.404	5.208	1.00	15.00	A	O

ATOM	3540	OE2	GLU	A	457	4.524	10.693	4.432	1.00	15.00	A	O
ATOM	3541	C	GLU	A	457	7.272	11.943	7.191	1.00	15.00	A	C
ATOM	3542	O	GLU	A	457	6.567	12.949	7.297	1.00	15.00	A	O
ATOM	3543	N	LYS	A	458	8.322	11.709	7.977	1.00	15.00	A	N
ATOM	3544	CA	LYS	A	458	8.713	12.642	9.028	1.00	15.00	A	C
ATOM	3545	CB	LYS	A	458	9.946	12.128	9.778	1.00	15.00	A	C
ATOM	3546	CG	LYS	A	458	10.022	12.561	11.235	1.00	15.00	A	C
ATOM	3547	CD	LYS	A	458	11.367	13.195	11.560	1.00	15.00	A	C
ATOM	3548	CE	LYS	A	458	11.675	13.136	13.051	1.00	15.00	A	C
ATOM	3549	NZ	LYS	A	458	10.491	13.470	13.890	1.00	15.00	A	N
ATOM	3550	C	LYS	A	458	9.006	14.010	8.426	1.00	15.00	A	C
ATOM	3551	O	LYS	A	458	8.586	15.035	8.962	1.00	15.00	A	O
ATOM	3552	N	TRP	A	459	9.718	14.010	7.301	1.00	15.00	A	N
ATOM	3553	CA	TRP	A	459	10.064	15.243	6.603	1.00	15.00	A	C
ATOM	3554	CB	TRP	A	459	10.870	14.922	5.336	1.00	15.00	A	C
ATOM	3555	CG	TRP	A	459	11.333	16.130	4.573	1.00	15.00	A	C
ATOM	3556	CD1	TRP	A	459	12.454	16.871	4.811	1.00	15.00	A	C
ATOM	3557	CD2	TRP	A	459	10.691	16.731	3.439	1.00	15.00	A	C
ATOM	3558	NE1	TRP	A	459	12.547	17.897	3.902	1.00	15.00	A	N
ATOM	3559	CE2	TRP	A	459	11.477	17.833	3.050	1.00	15.00	A	C
ATOM	3560	CE3	TRP	A	459	9.527	16.446	2.719	1.00	15.00	A	C
ATOM	3561	CZ2	TRP	A	459	11.138	18.647	1.972	1.00	15.00	A	C
ATOM	3562	CZ3	TRP	A	459	9.192	17.256	1.651	1.00	15.00	A	C
ATOM	3563	CH2	TRP	A	459	9.994	18.343	1.286	1.00	15.00	A	C
ATOM	3564	C	TRP	A	459	8.802	16.017	6.240	1.00	15.00	A	C
ATOM	3565	O	TRP	A	459	8.699	17.216	6.498	1.00	15.00	A	O
ATOM	3566	N	ARG	A	460	7.836	15.310	5.669	1.00	15.00	A	N
ATOM	3567	CA	ARG	A	460	6.576	15.915	5.260	1.00	15.00	A	C
ATOM	3568	CB	ARG	A	460	5.726	14.904	4.497	1.00	15.00	A	C
ATOM	3569	CG	ARG	A	460	6.457	14.258	3.337	1.00	15.00	A	C
ATOM	3570	CD	ARG	A	460	5.763	12.990	2.889	1.00	15.00	A	C
ATOM	3571	NE	ARG	A	460	4.990	13.195	1.670	1.00	15.00	A	N
ATOM	3572	CZ	ARG	A	460	4.019	12.382	1.265	1.00	15.00	A	C
ATOM	3573	NH1	ARG	A	460	3.691	11.315	1.984	1.00	15.00	A	N
ATOM	3574	NH2	ARG	A	460	3.362	12.643	0.144	1.00	15.00	A	N
ATOM	3575	C	ARG	A	460	5.808	16.459	6.455	1.00	15.00	A	C
ATOM	3576	O	ARG	A	460	5.302	17.576	6.411	1.00	15.00	A	O
ATOM	3577	N	TRP	A	461	5.732	15.670	7.523	1.00	15.00	A	N
ATOM	3578	CA	TRP	A	461	5.026	16.082	8.734	1.00	15.00	A	C
ATOM	3579	CB	TRP	A	461	5.114	14.996	9.809	1.00	15.00	A	C
ATOM	3580	CG	TRP	A	461	4.207	13.824	9.581	1.00	15.00	A	C
ATOM	3581	CD1	TRP	A	461	3.166	13.745	8.700	1.00	15.00	A	C
ATOM	3582	CD2	TRP	A	461	4.260	12.562	10.254	1.00	15.00	A	C
ATOM	3583	NE1	TRP	A	461	2.566	12.512	8.788	1.00	15.00	A	N
ATOM	3584	CE2	TRP	A	461	3.222	11.766	9.732	1.00	15.00	A	C
ATOM	3585	CE3	TRP	A	461	5.087	12.028	11.248	1.00	15.00	A	C
ATOM	3586	CZ2	TRP	A	461	2.989	10.464	10.171	1.00	15.00	A	C
ATOM	3587	CZ3	TRP	A	461	4.855	10.737	11.682	1.00	15.00	A	C
ATOM	3588	CH2	TRP	A	461	3.815	9.969	11.144	1.00	15.00	A	C
ATOM	3589	C	TRP	A	461	5.596	17.388	9.278	1.00	15.00	A	C
ATOM	3590	O	TRP	A	461	4.855	18.323	9.585	1.00	15.00	A	O
ATOM	3591	N	MET	A	462	6.917	17.447	9.376	1.00	15.00	A	N
ATOM	3592	CA	MET	A	462	7.594	18.635	9.878	1.00	15.00	A	C
ATOM	3593	CB	MET	A	462	9.087	18.357	10.082	1.00	15.00	A	C
ATOM	3594	CG	MET	A	462	9.372	17.264	11.104	1.00	15.00	A	C
ATOM	3595	SD	MET	A	462	11.125	16.864	11.258	1.00	15.00	A	S
ATOM	3596	CE	MET	A	462	11.524	16.478	9.561	1.00	15.00	A	C
ATOM	3597	C	MET	A	462	7.380	19.813	8.930	1.00	15.00	A	C
ATOM	3598	O	MET	A	462	7.145	20.942	9.365	1.00	15.00	A	O
ATOM	3599	N	VAL	A	463	7.440	19.538	7.632	1.00	15.00	A	N
ATOM	3600	CA	VAL	A	463	7.240	20.568	6.618	1.00	15.00	A	C
ATOM	3601	CB	VAL	A	463	7.546	20.030	5.196	1.00	15.00	A	C
ATOM	3602	CG1	VAL	A	463	6.858	20.861	4.120	1.00	15.00	A	C
ATOM	3603	CG2	VAL	A	463	9.046	20.003	4.954	1.00	15.00	A	C
ATOM	3604	C	VAL	A	463	5.813	21.119	6.677	1.00	15.00	A	C
ATOM	3605	O	VAL	A	463	5.604	22.330	6.602	1.00	15.00	A	O
ATOM	3606	N	PHE	A	464	4.842	20.226	6.839	1.00	15.00	A	N
ATOM	3607	CA	PHE	A	464	3.439	20.617	6.897	1.00	15.00	A	C
ATOM	3608	CB	PHE	A	464	2.516	19.397	6.844	1.00	15.00	A	C
ATOM	3609	CG	PHE	A	464	2.462	18.744	5.492	1.00	15.00	A	C
ATOM	3610	CD1	PHE	A	464	2.495	19.511	4.339	1.00	15.00	A	C

ATOM	3611	CD2	PHE	A	464	2.382	17.366	5.373	1.00	15.00	A	C
ATOM	3612	CE1	PHE	A	464	2.450	18.918	3.092	1.00	15.00	A	C
ATOM	3613	CE2	PHE	A	464	2.336	16.766	4.129	1.00	15.00	A	C
ATOM	3614	CZ	PHE	A	464	2.371	17.543	2.987	1.00	15.00	A	C
ATOM	3615	C	PHE	A	464	3.128	21.486	8.110	1.00	15.00	A	C
ATOM	3616	O	PHE	A	464	2.339	22.425	8.020	1.00	15.00	A	O
ATOM	3617	N	LYS	A	465	3.738	21.178	9.243	1.00	15.00	A	N
ATOM	3618	CA	LYS	A	465	3.505	21.964	10.444	1.00	15.00	A	C
ATOM	3619	CB	LYS	A	465	3.960	21.215	11.691	1.00	15.00	A	C
ATOM	3620	CG	LYS	A	465	3.478	21.848	12.988	1.00	15.00	A	C
ATOM	3621	CD	LYS	A	465	3.575	20.878	14.150	1.00	15.00	A	C
ATOM	3622	CE	LYS	A	465	3.032	19.511	13.770	1.00	15.00	A	C
ATOM	3623	NZ	LYS	A	465	3.002	18.589	14.929	1.00	15.00	A	N
ATOM	3624	C	LYS	A	465	4.210	23.310	10.350	1.00	15.00	A	C
ATOM	3625	O	LYS	A	465	3.651	24.343	10.724	1.00	15.00	A	O
ATOM	3626	N	GLY	A	466	5.427	23.288	9.828	1.00	15.00	A	N
ATOM	3627	CA	GLY	A	466	6.205	24.503	9.695	1.00	15.00	A	C
ATOM	3628	C	GLY	A	466	7.523	24.406	10.434	1.00	15.00	A	C
ATOM	3629	O	GLY	A	466	8.162	25.415	10.719	1.00	15.00	A	O
ATOM	3630	N	GLU	A	467	7.927	23.177	10.731	1.00	15.00	A	N
ATOM	3631	CA	GLU	A	467	9.169	22.920	11.446	1.00	15.00	A	C
ATOM	3632	CB	GLU	A	467	9.104	21.546	12.128	1.00	15.00	A	C
ATOM	3633	CG	GLU	A	467	7.961	21.417	13.136	1.00	15.00	A	C
ATOM	3634	CD	GLU	A	467	7.587	19.978	13.459	1.00	15.00	A	C
ATOM	3635	OE1	GLU	A	467	6.426	19.737	13.865	1.00	15.00	A	O
ATOM	3636	OE2	GLU	A	467	8.444	19.087	13.308	1.00	15.00	A	O
ATOM	3637	C	GLU	A	467	10.372	23.017	10.503	1.00	15.00	A	C
ATOM	3638	O	GLU	A	467	11.518	23.124	10.943	1.00	15.00	A	O
ATOM	3639	N	ILE	A	468	10.104	22.988	9.200	1.00	15.00	A	N
ATOM	3640	CA	ILE	A	468	11.160	23.085	8.197	1.00	15.00	A	C
ATOM	3641	CB	ILE	A	468	11.258	21.810	7.329	1.00	15.00	A	C
ATOM	3642	CG1	ILE	A	468	11.599	20.597	8.201	1.00	15.00	A	C
ATOM	3643	CG2	ILE	A	468	12.300	21.984	6.230	1.00	15.00	A	C
ATOM	3644	CD1	ILE	A	468	11.748	19.306	7.426	1.00	15.00	A	C
ATOM	3645	C	ILE	A	468	10.946	24.305	7.298	1.00	15.00	A	C
ATOM	3646	O	ILE	A	468	9.935	24.402	6.598	1.00	15.00	A	O
ATOM	3647	N	PRO	A	469	11.892	25.258	7.325	1.00	15.00	A	N
ATOM	3648	CA	PRO	A	469	11.824	26.479	6.513	1.00	15.00	A	C
ATOM	3649	CB	PRO	A	469	13.014	27.315	7.006	1.00	15.00	A	C
ATOM	3650	CG	PRO	A	469	13.440	26.679	8.286	1.00	15.00	A	C
ATOM	3651	CD	PRO	A	469	13.103	25.226	8.155	1.00	15.00	A	C
ATOM	3652	C	PRO	A	469	11.976	26.177	5.022	1.00	15.00	A	C
ATOM	3653	O	PRO	A	469	12.629	25.205	4.642	1.00	15.00	A	O
ATOM	3654	N	LYS	A	470	11.384	27.027	4.187	1.00	15.00	A	N
ATOM	3655	CA	LYS	A	470	11.433	26.857	2.732	1.00	15.00	A	C
ATOM	3656	CB	LYS	A	470	10.597	27.939	2.050	1.00	15.00	A	C
ATOM	3657	CG	LYS	A	470	9.128	27.585	1.875	1.00	15.00	A	C
ATOM	3658	CD	LYS	A	470	8.366	28.702	1.174	1.00	15.00	A	C
ATOM	3659	CE	LYS	A	470	9.109	29.195	-0.060	1.00	15.00	A	C
ATOM	3660	NZ	LYS	A	470	8.191	29.458	-1.198	1.00	15.00	A	N
ATOM	3661	C	LYS	A	470	12.863	26.915	2.206	1.00	15.00	A	C
ATOM	3662	O	LYS	A	470	13.199	26.273	1.210	1.00	15.00	A	O
ATOM	3663	N	ASP	A	471	13.684	27.697	2.888	1.00	15.00	A	N
ATOM	3664	CA	ASP	A	471	15.088	27.882	2.535	1.00	15.00	A	C
ATOM	3665	CB	ASP	A	471	15.612	29.122	3.260	1.00	15.00	A	C
ATOM	3666	CG	ASP	A	471	15.162	29.146	4.710	1.00	15.00	A	C
ATOM	3667	OD1	ASP	A	471	14.009	29.569	4.961	1.00	15.00	A	O
ATOM	3668	OD2	ASP	A	471	15.934	28.713	5.594	1.00	15.00	A	O
ATOM	3669	C	ASP	A	471	15.933	26.685	2.954	1.00	15.00	A	C
ATOM	3670	O	ASP	A	471	17.124	26.623	2.655	1.00	15.00	A	O
ATOM	3671	N	GLN	A	472	15.321	25.738	3.652	1.00	15.00	A	N
ATOM	3672	CA	GLN	A	472	16.037	24.560	4.117	1.00	15.00	A	C
ATOM	3673	CB	GLN	A	472	16.347	24.679	5.611	1.00	15.00	A	C
ATOM	3674	CG	GLN	A	472	17.419	25.703	5.944	1.00	15.00	A	C
ATOM	3675	CD	GLN	A	472	17.630	25.852	7.435	1.00	15.00	A	C
ATOM	3676	OE1	GLN	A	472	18.457	25.154	8.033	1.00	15.00	A	O
ATOM	3677	NE2	GLN	A	472	16.895	26.768	8.044	1.00	15.00	A	N
ATOM	3678	C	GLN	A	472	15.247	23.287	3.849	1.00	15.00	A	C
ATOM	3679	O	GLN	A	472	15.350	22.313	4.596	1.00	15.00	A	O
ATOM	3680	N	TRP	A	473	14.476	23.291	2.772	1.00	15.00	A	N
ATOM	3681	CA	TRP	A	473	13.666	22.137	2.411	1.00	15.00	A	C

ATOM	3682	CB	TRP	A	473	12.668	22.505	1.312	1.00	15.00	A	C
ATOM	3683	CG	TRP	A	473	11.300	22.833	1.820	1.00	15.00	A	C
ATOM	3684	CD1	TRP	A	473	10.955	23.152	3.100	1.00	15.00	A	C
ATOM	3685	CD2	TRP	A	473	10.091	22.871	1.057	1.00	15.00	A	C
ATOM	3686	NE1	TRP	A	473	9.607	23.391	3.182	1.00	15.00	A	N
ATOM	3687	CE2	TRP	A	473	9.051	23.224	1.939	1.00	15.00	A	C
ATOM	3688	CE3	TRP	A	473	9.786	22.642	-0.289	1.00	15.00	A	C
ATOM	3689	CZ2	TRP	A	473	7.730	23.353	1.520	1.00	15.00	A	C
ATOM	3690	CZ3	TRP	A	473	8.475	22.771	-0.703	1.00	15.00	A	C
ATOM	3691	CH2	TRP	A	473	7.462	23.123	0.198	1.00	15.00	A	C
ATOM	3692	C	TRP	A	473	14.545	20.989	1.941	1.00	15.00	A	C
ATOM	3693	O	TRP	A	473	14.457	19.869	2.453	1.00	15.00	A	O
ATOM	3694	N	MET	A	474	15.406	21.281	0.978	1.00	15.00	A	N
ATOM	3695	CA	MET	A	474	16.301	20.277	0.427	1.00	15.00	A	C
ATOM	3696	CB	MET	A	474	16.880	20.730	-0.914	1.00	15.00	A	C
ATOM	3697	CG	MET	A	474	15.873	20.751	-2.052	1.00	15.00	A	C
ATOM	3698	SD	MET	A	474	14.845	19.268	-2.111	1.00	15.00	A	S
ATOM	3699	CE	MET	A	474	16.087	17.991	-1.927	1.00	15.00	A	C
ATOM	3700	C	MET	A	474	17.420	19.960	1.400	1.00	15.00	A	C
ATOM	3701	O	MET	A	474	17.828	18.806	1.539	1.00	15.00	A	O
ATOM	3702	N	LYS	A	475	17.908	20.993	2.069	1.00	15.00	A	N
ATOM	3703	CA	LYS	A	475	18.977	20.843	3.040	1.00	15.00	A	C
ATOM	3704	CB	LYS	A	475	19.321	22.200	3.655	1.00	15.00	A	C
ATOM	3705	CG	LYS	A	475	20.528	22.175	4.573	1.00	15.00	A	C
ATOM	3706	CD	LYS	A	475	20.537	23.380	5.494	1.00	15.00	A	C
ATOM	3707	CE	LYS	A	475	21.386	23.117	6.721	1.00	15.00	A	C
ATOM	3708	NZ	LYS	A	475	21.008	23.995	7.858	1.00	15.00	A	N
ATOM	3709	C	LYS	A	475	18.590	19.852	4.135	1.00	15.00	A	C
ATOM	3710	O	LYS	A	475	19.278	18.853	4.346	1.00	15.00	A	O
ATOM	3711	N	LYS	A	476	17.469	20.116	4.801	1.00	15.00	A	N
ATOM	3712	CA	LYS	A	476	16.993	19.255	5.881	1.00	15.00	A	C
ATOM	3713	CB	LYS	A	476	15.732	19.839	6.524	1.00	15.00	A	C
ATOM	3714	CG	LYS	A	476	15.884	20.160	8.002	1.00	15.00	A	C
ATOM	3715	CD	LYS	A	476	16.136	18.897	8.810	1.00	15.00	A	C
ATOM	3716	CE	LYS	A	476	16.626	19.215	10.212	1.00	15.00	A	C
ATOM	3717	NZ	LYS	A	476	17.048	17.984	10.934	1.00	15.00	A	N
ATOM	3718	C	LYS	A	476	16.729	17.835	5.389	1.00	15.00	A	C
ATOM	3719	O	LYS	A	476	16.928	16.863	6.123	1.00	15.00	A	O
ATOM	3720	N	TRP	A	477	16.288	17.728	4.143	1.00	15.00	A	N
ATOM	3721	CA	TRP	A	477	15.999	16.439	3.532	1.00	15.00	A	C
ATOM	3722	CB	TRP	A	477	15.443	16.643	2.119	1.00	15.00	A	C
ATOM	3723	CG	TRP	A	477	15.333	15.387	1.309	1.00	15.00	A	C
ATOM	3724	CD1	TRP	A	477	16.105	15.033	0.242	1.00	15.00	A	C
ATOM	3725	CD2	TRP	A	477	14.394	14.322	1.495	1.00	15.00	A	C
ATOM	3726	NE1	TRP	A	477	15.705	13.816	-0.250	1.00	15.00	A	N
ATOM	3727	CE2	TRP	A	477	14.656	13.357	0.503	1.00	15.00	A	C
ATOM	3728	CE3	TRP	A	477	13.358	14.092	2.404	1.00	15.00	A	C
ATOM	3729	CZ2	TRP	A	477	13.919	12.181	0.396	1.00	15.00	A	C
ATOM	3730	CZ3	TRP	A	477	12.628	12.925	2.295	1.00	15.00	A	C
ATOM	3731	CH2	TRP	A	477	12.912	11.984	1.300	1.00	15.00	A	C
ATOM	3732	C	TRP	A	477	17.248	15.563	3.493	1.00	15.00	A	C
ATOM	3733	O	TRP	A	477	17.219	14.402	3.906	1.00	15.00	A	O
ATOM	3734	N	TRP	A	478	18.350	16.127	3.020	1.00	15.00	A	N
ATOM	3735	CA	TRP	A	478	19.593	15.379	2.927	1.00	15.00	A	C
ATOM	3736	CB	TRP	A	478	20.505	15.907	1.823	1.00	15.00	A	C
ATOM	3737	CG	TRP	A	478	19.983	15.526	0.472	1.00	15.00	A	C
ATOM	3738	CD1	TRP	A	478	19.436	16.351	-0.469	1.00	15.00	A	C
ATOM	3739	CD2	TRP	A	478	19.926	14.203	-0.073	1.00	15.00	A	C
ATOM	3740	NE1	TRP	A	478	19.040	15.618	-1.563	1.00	15.00	A	N
ATOM	3741	CE2	TRP	A	478	19.336	14.299	-1.345	1.00	15.00	A	C
ATOM	3742	CE3	TRP	A	478	20.323	12.945	0.392	1.00	15.00	A	C
ATOM	3743	CZ2	TRP	A	478	19.125	13.187	-2.155	1.00	15.00	A	C
ATOM	3744	CZ3	TRP	A	478	20.115	11.842	-0.413	1.00	15.00	A	C
ATOM	3745	CH2	TRP	A	478	19.523	11.970	-1.673	1.00	15.00	A	C
ATOM	3746	C	TRP	A	478	20.280	15.227	4.277	1.00	15.00	A	C
ATOM	3747	O	TRP	A	478	21.052	14.287	4.488	1.00	15.00	A	O
ATOM	3748	N	GLU	A	479	19.982	16.139	5.198	1.00	15.00	A	N
ATOM	3749	CA	GLU	A	479	20.533	16.060	6.542	1.00	15.00	A	C
ATOM	3750	CB	GLU	A	479	20.078	17.254	7.390	1.00	15.00	A	C
ATOM	3751	CG	GLU	A	479	20.773	18.573	7.086	1.00	15.00	A	C
ATOM	3752	CD	GLU	A	479	20.201	19.720	7.902	1.00	15.00	A	C

ATOM	3753	OE1	GLU	A	479	19.015	19.648	8.278	1.00	15.00	A	O
ATOM	3754	OE2	GLU	A	479	20.932	20.696	8.168	1.00	15.00	A	O
ATOM	3755	C	GLU	A	479	20.023	14.777	7.189	1.00	15.00	A	C
ATOM	3756	O	GLU	A	479	20.795	13.969	7.702	1.00	15.00	A	O
ATOM	3757	N	MET	A	480	18.710	14.581	7.105	1.00	15.00	A	N
ATOM	3758	CA	MET	A	480	18.064	13.408	7.681	1.00	15.00	A	C
ATOM	3759	CB	MET	A	480	16.563	13.640	7.819	1.00	15.00	A	C
ATOM	3760	CG	MET	A	480	16.169	14.410	9.067	1.00	15.00	A	C
ATOM	3761	SD	MET	A	480	14.646	15.350	8.844	1.00	15.00	A	S
ATOM	3762	CE	MET	A	480	13.631	14.145	7.987	1.00	15.00	A	C
ATOM	3763	C	MET	A	480	18.325	12.151	6.860	1.00	15.00	A	C
ATOM	3764	O	MET	A	480	18.306	11.042	7.392	1.00	15.00	A	O
ATOM	3765	N	LYS	A	481	18.558	12.319	5.562	1.00	15.00	A	N
ATOM	3766	CA	LYS	A	481	18.834	11.183	4.690	1.00	15.00	A	C
ATOM	3767	CB	LYS	A	481	18.941	11.626	3.230	1.00	15.00	A	C
ATOM	3768	CG	LYS	A	481	17.705	11.332	2.391	1.00	15.00	A	C
ATOM	3769	CD	LYS	A	481	17.687	9.890	1.902	1.00	15.00	A	C
ATOM	3770	CE	LYS	A	481	16.654	9.694	0.802	1.00	15.00	A	C
ATOM	3771	NZ	LYS	A	481	16.695	8.318	0.227	1.00	15.00	A	N
ATOM	3772	C	LYS	A	481	20.111	10.475	5.123	1.00	15.00	A	C
ATOM	3773	O	LYS	A	481	20.163	9.247	5.176	1.00	15.00	A	O
ATOM	3774	N	ARG	A	482	21.133	11.259	5.443	1.00	15.00	A	N
ATOM	3775	CA	ARG	A	482	22.408	10.709	5.887	1.00	15.00	A	C
ATOM	3776	CB	ARG	A	482	23.514	11.764	5.780	1.00	15.00	A	C
ATOM	3777	CG	ARG	A	482	23.708	12.407	4.414	1.00	15.00	A	C
ATOM	3778	CD	ARG	A	482	24.698	13.557	4.534	1.00	15.00	A	C
ATOM	3779	NE	ARG	A	482	25.034	14.192	3.260	1.00	15.00	A	N
ATOM	3780	CZ	ARG	A	482	26.171	14.858	3.052	1.00	15.00	A	C
ATOM	3781	NH1	ARG	A	482	27.075	14.959	4.024	1.00	15.00	A	N
ATOM	3782	NH2	ARG	A	482	26.389	15.453	1.889	1.00	15.00	A	N
ATOM	3783	C	ARG	A	482	22.308	10.276	7.348	1.00	15.00	A	C
ATOM	3784	O	ARG	A	482	22.922	9.300	7.764	1.00	15.00	A	O
ATOM	3785	N	GLU	A	483	21.518	11.020	8.107	1.00	15.00	A	N
ATOM	3786	CA	GLU	A	483	21.336	10.781	9.535	1.00	15.00	A	C
ATOM	3787	CB	GLU	A	483	20.697	12.028	10.161	1.00	15.00	A	C
ATOM	3788	CG	GLU	A	483	20.256	11.896	11.608	1.00	15.00	A	C
ATOM	3789	CD	GLU	A	483	19.475	13.114	12.066	1.00	15.00	A	C
ATOM	3790	OE1	GLU	A	483	18.293	13.246	11.673	1.00	15.00	A	O
ATOM	3791	OE2	GLU	A	483	20.044	13.946	12.800	1.00	15.00	A	O
ATOM	3792	C	GLU	A	483	20.506	9.533	9.850	1.00	15.00	A	C
ATOM	3793	O	GLU	A	483	21.018	8.556	10.400	1.00	15.00	A	O
ATOM	3794	N	ILE	A	484	19.234	9.568	9.483	1.00	15.00	A	N
ATOM	3795	CA	ILE	A	484	18.309	8.478	9.771	1.00	15.00	A	C
ATOM	3796	CB	ILE	A	484	16.844	8.962	9.666	1.00	15.00	A	C
ATOM	3797	CG1	ILE	A	484	16.623	10.193	10.553	1.00	15.00	A	C
ATOM	3798	CG2	ILE	A	484	15.873	7.851	10.040	1.00	15.00	A	C
ATOM	3799	CD1	ILE	A	484	15.298	10.888	10.322	1.00	15.00	A	C
ATOM	3800	C	ILE	A	484	18.498	7.255	8.872	1.00	15.00	A	C
ATOM	3801	O	ILE	A	484	18.539	6.124	9.356	1.00	15.00	A	O
ATOM	3802	N	VAL	A	485	18.616	7.478	7.572	1.00	15.00	A	N
ATOM	3803	CA	VAL	A	485	18.739	6.372	6.624	1.00	15.00	A	C
ATOM	3804	CB	VAL	A	485	18.014	6.681	5.295	1.00	15.00	A	C
ATOM	3805	CG1	VAL	A	485	17.563	5.398	4.613	1.00	15.00	A	C
ATOM	3806	CG2	VAL	A	485	16.829	7.604	5.528	1.00	15.00	A	C
ATOM	3807	C	VAL	A	485	20.188	5.979	6.337	1.00	15.00	A	C
ATOM	3808	O	VAL	A	485	20.456	4.864	5.886	1.00	15.00	A	O
ATOM	3809	N	GLY	A	486	21.119	6.882	6.597	1.00	15.00	A	N
ATOM	3810	CA	GLY	A	486	22.514	6.588	6.328	1.00	15.00	A	C
ATOM	3811	C	GLY	A	486	22.789	6.567	4.838	1.00	15.00	A	C
ATOM	3812	O	GLY	A	486	23.615	5.790	4.349	1.00	15.00	A	O
ATOM	3813	N	VAL	A	487	22.080	7.422	4.115	1.00	15.00	A	N
ATOM	3814	CA	VAL	A	487	22.219	7.523	2.672	1.00	15.00	A	C
ATOM	3815	CB	VAL	A	487	20.864	7.289	1.972	1.00	15.00	A	C
ATOM	3816	CG1	VAL	A	487	20.978	7.525	0.475	1.00	15.00	A	C
ATOM	3817	CG2	VAL	A	487	20.354	5.885	2.255	1.00	15.00	A	C
ATOM	3818	C	VAL	A	487	22.745	8.905	2.307	1.00	15.00	A	C
ATOM	3819	O	VAL	A	487	22.187	9.915	2.730	1.00	15.00	A	O
ATOM	3820	N	VAL	A	488	23.803	8.945	1.515	1.00	15.00	A	N
ATOM	3821	CA	VAL	A	488	24.406	10.207	1.124	1.00	15.00	A	C
ATOM	3822	CB	VAL	A	488	25.907	10.249	1.491	1.00	15.00	A	C
ATOM	3823	CG1	VAL	A	488	26.706	9.218	0.706	1.00	15.00	A	C

ATOM	3824	CG2	VAL	A	488	26.478	11.644	1.310	1.00	15.00	A	C
ATOM	3825	C	VAL	A	488	24.203	10.498	-0.365	1.00	15.00	A	C
ATOM	3826	O	VAL	A	488	24.211	9.587	-1.201	1.00	15.00	A	O
ATOM	3827	N	GLU	A	489	24.005	11.769	-0.683	1.00	15.00	A	N
ATOM	3828	CA	GLU	A	489	23.798	12.197	-2.053	1.00	15.00	A	C
ATOM	3829	CB	GLU	A	489	22.930	13.467	-2.099	1.00	15.00	A	C
ATOM	3830	CG	GLU	A	489	23.651	14.783	-1.819	1.00	15.00	A	C
ATOM	3831	CD	GLU	A	489	24.111	14.967	-0.380	1.00	15.00	A	C
ATOM	3832	OE1	GLU	A	489	24.781	15.982	-0.105	1.00	15.00	A	O
ATOM	3833	OE2	GLU	A	489	23.823	14.105	0.480	1.00	15.00	A	O
ATOM	3834	C	GLU	A	489	25.134	12.406	-2.767	1.00	15.00	A	C
ATOM	3835	O	GLU	A	489	26.075	12.957	-2.198	1.00	15.00	A	O
ATOM	3836	N	PRO	A	490	25.245	11.930	-4.015	1.00	15.00	A	N
ATOM	3837	CA	PRO	A	490	26.473	12.068	-4.803	1.00	15.00	A	C
ATOM	3838	CB	PRO	A	490	26.277	11.050	-5.925	1.00	15.00	A	C
ATOM	3839	CG	PRO	A	490	24.799	10.967	-6.103	1.00	15.00	A	C
ATOM	3840	CD	PRO	A	490	24.194	11.197	-4.746	1.00	15.00	A	C
ATOM	3841	C	PRO	A	490	26.645	13.473	-5.381	1.00	15.00	A	C
ATOM	3842	O	PRO	A	490	27.760	13.902	-5.671	1.00	15.00	A	O
ATOM	3843	N	VAL	A	491	25.537	14.183	-5.544	1.00	15.00	A	N
ATOM	3844	CA	VAL	A	491	25.567	15.531	-6.092	1.00	15.00	A	C
ATOM	3845	CB	VAL	A	491	24.803	15.612	-7.435	1.00	15.00	A	C
ATOM	3846	CG1	VAL	A	491	24.902	17.007	-8.037	1.00	15.00	A	C
ATOM	3847	CG2	VAL	A	491	25.318	14.570	-8.417	1.00	15.00	A	C
ATOM	3848	C	VAL	A	491	24.941	16.515	-5.109	1.00	15.00	A	C
ATOM	3849	O	VAL	A	491	23.801	16.325	-4.683	1.00	15.00	A	O
ATOM	3850	N	PRO	A	492	25.683	17.570	-4.727	1.00	15.00	A	N
ATOM	3851	CA	PRO	A	492	25.187	18.592	-3.799	1.00	15.00	A	C
ATOM	3852	CB	PRO	A	492	26.360	19.574	-3.668	1.00	15.00	A	C
ATOM	3853	CG	PRO	A	492	27.233	19.301	-4.847	1.00	15.00	A	C
ATOM	3854	CD	PRO	A	492	27.059	17.845	-5.168	1.00	15.00	A	C
ATOM	3855	C	PRO	A	492	23.960	19.301	-4.368	1.00	15.00	A	C
ATOM	3856	O	PRO	A	492	24.054	20.043	-5.346	1.00	15.00	A	O
ATOM	3857	N	HIS	A	493	22.808	19.052	-3.766	1.00	15.00	A	N
ATOM	3858	CA	HIS	A	493	21.565	19.651	-4.228	1.00	15.00	A	C
ATOM	3859	CB	HIS	A	493	20.418	18.641	-4.164	1.00	15.00	A	C
ATOM	3860	CG	HIS	A	493	20.397	17.675	-5.310	1.00	15.00	A	C
ATOM	3861	ND1	HIS	A	493	19.376	16.776	-5.519	1.00	15.00	A	N
ATOM	3862	CD2	HIS	A	493	21.277	17.477	-6.320	1.00	15.00	A	C
ATOM	3863	CE1	HIS	A	493	19.628	16.069	-6.600	1.00	15.00	A	C
ATOM	3864	NE2	HIS	A	493	20.772	16.475	-7.103	1.00	15.00	A	N
ATOM	3865	C	HIS	A	493	21.213	20.906	-3.443	1.00	15.00	A	C
ATOM	3866	O	HIS	A	493	21.234	20.907	-2.213	1.00	15.00	A	O
ATOM	3867	N	ASP	A	494	20.895	21.971	-4.167	1.00	15.00	A	N
ATOM	3868	CA	ASP	A	494	20.525	23.241	-3.557	1.00	15.00	A	C
ATOM	3869	CB	ASP	A	494	21.021	24.409	-4.415	1.00	15.00	A	C
ATOM	3870	CG	ASP	A	494	20.208	24.588	-5.679	1.00	15.00	A	C
ATOM	3871	OD1	ASP	A	494	20.243	23.694	-6.551	1.00	15.00	A	O
ATOM	3872	OD2	ASP	A	494	19.515	25.613	-5.798	1.00	15.00	A	O
ATOM	3873	C	ASP	A	494	19.010	23.317	-3.375	1.00	15.00	A	C
ATOM	3874	O	ASP	A	494	18.297	22.344	-3.635	1.00	15.00	A	O
ATOM	3875	N	GLU	A	495	18.513	24.477	-2.963	1.00	15.00	A	N
ATOM	3876	CA	GLU	A	495	17.085	24.650	-2.723	1.00	15.00	A	C
ATOM	3877	CB	GLU	A	495	16.809	25.766	-1.712	1.00	15.00	A	C
ATOM	3878	CG	GLU	A	495	17.276	25.452	-0.295	1.00	15.00	A	C
ATOM	3879	CD	GLU	A	495	16.625	24.209	0.292	1.00	15.00	A	C
ATOM	3880	OE1	GLU	A	495	15.416	23.992	0.060	1.00	15.00	A	O
ATOM	3881	OE2	GLU	A	495	17.315	23.443	0.993	1.00	15.00	A	O
ATOM	3882	C	GLU	A	495	16.245	24.818	-3.995	1.00	15.00	A	C
ATOM	3883	O	GLU	A	495	15.037	25.006	-3.912	1.00	15.00	A	O
ATOM	3884	N	THR	A	496	16.858	24.764	-5.173	1.00	15.00	A	N
ATOM	3885	CA	THR	A	496	16.076	24.873	-6.404	1.00	15.00	A	C
ATOM	3886	CB	THR	A	496	16.872	25.443	-7.597	1.00	15.00	A	C
ATOM	3887	OG1	THR	A	496	18.039	24.650	-7.851	1.00	15.00	A	O
ATOM	3888	CG2	THR	A	496	17.269	26.891	-7.352	1.00	15.00	A	C
ATOM	3889	C	THR	A	496	15.507	23.508	-6.779	1.00	15.00	A	C
ATOM	3890	O	THR	A	496	14.695	23.383	-7.696	1.00	15.00	A	O
ATOM	3891	N	TYR	A	497	15.952	22.487	-6.053	1.00	15.00	A	N
ATOM	3892	CA	TYR	A	497	15.514	21.123	-6.285	1.00	15.00	A	C
ATOM	3893	CB	TYR	A	497	16.630	20.140	-5.916	1.00	15.00	A	C
ATOM	3894	CG	TYR	A	497	17.712	19.997	-6.954	1.00	15.00	A	C

```
ATOM   3895  CD1 TYR A 497      18.882  20.738  -6.872  1.00 15.00       A    C
ATOM   3896  CD2 TYR A 497      17.564  19.122  -8.017  1.00 15.00       A    C
ATOM   3897  CE1 TYR A 497      19.874  20.613  -7.824  1.00 15.00       A    C
ATOM   3898  CE2 TYR A 497      18.550  18.987  -8.972  1.00 15.00       A    C
ATOM   3899  CZ  TYR A 497      19.703  19.735  -8.871  1.00 15.00       A    C
ATOM   3900  OH  TYR A 497      20.678  19.621  -9.831  1.00 15.00       A    O
ATOM   3901  C   TYR A 497      14.283  20.782  -5.461  1.00 15.00       A    C
ATOM   3902  O   TYR A 497      13.793  21.590  -4.665  1.00 15.00       A    O
ATOM   3903  N   CYS A 498      13.806  19.568  -5.662  1.00 15.00       A    N
ATOM   3904  CA  CYS A 498      12.661  19.036  -4.950  1.00 15.00       A    C
ATOM   3905  CB  CYS A 498      11.349  19.599  -5.501  1.00 15.00       A    C
ATOM   3906  SG  CYS A 498      10.092  19.902  -4.237  1.00 15.00       A    S
ATOM   3907  C   CYS A 498      12.699  17.519  -5.067  1.00 15.00       A    C
ATOM   3908  O   CYS A 498      11.784  16.892  -5.597  1.00 15.00       A    O
ATOM   3909  N   ASP A 499      13.796  16.954  -4.574  1.00 15.00       A    N
ATOM   3910  CA  ASP A 499      14.040  15.511  -4.614  1.00 15.00       A    C
ATOM   3911  CB  ASP A 499      15.359  15.139  -3.923  1.00 15.00       A    C
ATOM   3912  CG  ASP A 499      16.557  15.881  -4.485  1.00 15.00       A    C
ATOM   3913  OD1 ASP A 499      16.465  16.421  -5.603  1.00 15.00       A    O
ATOM   3914  OD2 ASP A 499      17.599  15.936  -3.802  1.00 15.00       A    O
ATOM   3915  C   ASP A 499      12.885  14.669  -4.068  1.00 15.00       A    C
ATOM   3916  O   ASP A 499      12.483  13.704  -4.710  1.00 15.00       A    O
ATOM   3917  N   PRO A 500      12.331  14.988  -2.876  1.00 15.00       A    N
ATOM   3918  CA  PRO A 500      11.214  14.220  -2.314  1.00 15.00       A    C
ATOM   3919  CB  PRO A 500      10.839  14.990  -1.045  1.00 15.00       A    C
ATOM   3920  CG  PRO A 500      12.062  15.757  -0.686  1.00 15.00       A    C
ATOM   3921  CD  PRO A 500      12.749  16.083  -1.978  1.00 15.00       A    C
ATOM   3922  C   PRO A 500      10.019  14.178  -3.261  1.00 15.00       A    C
ATOM   3923  O   PRO A 500       9.305  13.181  -3.331  1.00 15.00       A    O
ATOM   3924  N   ALA A 501       9.831  15.252  -4.018  1.00 15.00       A    N
ATOM   3925  CA  ALA A 501       8.714  15.352  -4.947  1.00 15.00       A    C
ATOM   3926  CB  ALA A 501       8.470  16.800  -5.339  1.00 15.00       A    C
ATOM   3927  C   ALA A 501       8.916  14.484  -6.185  1.00 15.00       A    C
ATOM   3928  O   ALA A 501       7.975  14.246  -6.939  1.00 15.00       A    O
ATOM   3929  N   SER A 502      10.131  13.981  -6.381  1.00 15.00       A    N
ATOM   3930  CA  SER A 502      10.419  13.146  -7.538  1.00 15.00       A    C
ATOM   3931  CB  SER A 502      11.914  13.163  -7.890  1.00 15.00       A    C
ATOM   3932  OG  SER A 502      12.660  12.256  -7.093  1.00 15.00       A    O
ATOM   3933  C   SER A 502       9.902  11.720  -7.334  1.00 15.00       A    C
ATOM   3934  O   SER A 502      10.187  10.823  -8.131  1.00 15.00       A    O
ATOM   3935  N   LEU A 503       9.151  11.520  -6.253  1.00 15.00       A    N
ATOM   3936  CA  LEU A 503       8.568  10.225  -5.935  1.00 15.00       A    C
ATOM   3937  CB  LEU A 503       8.920   9.808  -4.502  1.00 15.00       A    C
ATOM   3938  CG  LEU A 503       8.207   8.565  -3.956  1.00 15.00       A    C
ATOM   3939  CD1 LEU A 503       8.585   7.323  -4.750  1.00 15.00       A    C
ATOM   3940  CD2 LEU A 503       8.520   8.374  -2.481  1.00 15.00       A    C
ATOM   3941  C   LEU A 503       7.053  10.278  -6.113  1.00 15.00       A    C
ATOM   3942  O   LEU A 503       6.400  11.211  -5.644  1.00 15.00       A    O
ATOM   3943  N   PHE A 504       6.515   9.264  -6.785  1.00 15.00       A    N
ATOM   3944  CA  PHE A 504       5.083   9.150  -7.070  1.00 15.00       A    C
ATOM   3945  CB  PHE A 504       4.764   7.722  -7.538  1.00 15.00       A    C
ATOM   3946  CG  PHE A 504       3.307   7.458  -7.793  1.00 15.00       A    C
ATOM   3947  CD1 PHE A 504       2.714   7.860  -8.978  1.00 15.00       A    C
ATOM   3948  CD2 PHE A 504       2.530   6.806  -6.847  1.00 15.00       A    C
ATOM   3949  CE1 PHE A 504       1.375   7.618  -9.217  1.00 15.00       A    C
ATOM   3950  CE2 PHE A 504       1.190   6.561  -7.079  1.00 15.00       A    C
ATOM   3951  CZ  PHE A 504       0.612   6.968  -8.266  1.00 15.00       A    C
ATOM   3952  C   PHE A 504       4.186   9.516  -5.886  1.00 15.00       A    C
ATOM   3953  O   PHE A 504       3.307  10.366  -6.006  1.00 15.00       A    O
ATOM   3954  N   HIS A 505       4.429   8.885  -4.747  1.00 15.00       A    N
ATOM   3955  CA  HIS A 505       3.617   9.098  -3.547  1.00 15.00       A    C
ATOM   3956  CB  HIS A 505       4.020   8.112  -2.448  1.00 15.00       A    C
ATOM   3957  CG  HIS A 505       3.812   6.677  -2.813  1.00 15.00       A    C
ATOM   3958  ND1 HIS A 505       4.774   5.888  -3.405  1.00 15.00       A    N
ATOM   3959  CD2 HIS A 505       2.720   5.887  -2.665  1.00 15.00       A    C
ATOM   3960  CE1 HIS A 505       4.249   4.672  -3.594  1.00 15.00       A    C
ATOM   3961  NE2 HIS A 505       3.005   4.619  -3.161  1.00 15.00       A    N
ATOM   3962  C   HIS A 505       3.680  10.523  -3.007  1.00 15.00       A    C
ATOM   3963  O   HIS A 505       2.813  10.936  -2.238  1.00 15.00       A    O
ATOM   3964  N   VAL A 506       4.685  11.277  -3.408  1.00 15.00       A    N
ATOM   3965  CA  VAL A 506       4.838  12.635  -2.917  1.00 15.00       A    C
```

```
ATOM   3966  CB  VAL A 506       6.314  12.974  -2.634  1.00 15.00       A    C
ATOM   3967  CG1 VAL A 506       6.437  14.323  -1.945  1.00 15.00       A    C
ATOM   3968  CG2 VAL A 506       6.944  11.885  -1.778  1.00 15.00       A    C
ATOM   3969  C   VAL A 506       4.220  13.663  -3.863  1.00 15.00       A    C
ATOM   3970  O   VAL A 506       3.524  14.576  -3.422  1.00 15.00       A    O
ATOM   3971  N   SER A 507       4.450  13.499  -5.160  1.00 15.00       A    N
ATOM   3972  CA  SER A 507       3.920  14.432  -6.149  1.00 15.00       A    C
ATOM   3973  CB  SER A 507       4.781  14.411  -7.411  1.00 15.00       A    C
ATOM   3974  OG  SER A 507       5.528  13.207  -7.492  1.00 15.00       A    O
ATOM   3975  C   SER A 507       2.460  14.145  -6.497  1.00 15.00       A    C
ATOM   3976  O   SER A 507       1.782  14.981  -7.093  1.00 15.00       A    O
ATOM   3977  N   ASN A 508       1.972  12.968  -6.123  1.00 15.00       A    N
ATOM   3978  CA  ASN A 508       0.592  12.597  -6.421  1.00 15.00       A    C
ATOM   3979  CB  ASN A 508       0.509  11.220  -7.083  1.00 15.00       A    C
ATOM   3980  CG  ASN A 508       0.972  11.246  -8.529  1.00 15.00       A    C
ATOM   3981  OD1 ASN A 508       0.172  11.401  -9.450  1.00 15.00       A    O
ATOM   3982  ND2 ASN A 508       2.272  11.099  -8.738  1.00 15.00       A    N
ATOM   3983  C   ASN A 508      -0.314  12.686  -5.198  1.00 15.00       A    C
ATOM   3984  O   ASN A 508      -1.452  12.226  -5.232  1.00 15.00       A    O
ATOM   3985  N   ASP A 509       0.212  13.275  -4.121  1.00 15.00       A    N
ATOM   3986  CA  ASP A 509      -0.546  13.473  -2.878  1.00 15.00       A    C
ATOM   3987  CB  ASP A 509      -1.716  14.440  -3.119  1.00 15.00       A    C
ATOM   3988  CG  ASP A 509      -2.421  14.885  -1.854  1.00 15.00       A    C
ATOM   3989  OD1 ASP A 509      -1.739  15.333  -0.906  1.00 15.00       A    O
ATOM   3990  OD2 ASP A 509      -3.669  14.837  -1.819  1.00 15.00       A    O
ATOM   3991  C   ASP A 509      -1.030  12.159  -2.248  1.00 15.00       A    C
ATOM   3992  O   ASP A 509      -2.214  11.830  -2.291  1.00 15.00       A    O
ATOM   3993  N   TYR A 510      -0.102  11.408  -1.666  1.00 15.00       A    N
ATOM   3994  CA  TYR A 510      -0.430  10.142  -1.019  1.00 15.00       A    C
ATOM   3995  CB  TYR A 510      -0.133   8.960  -1.950  1.00 15.00       A    C
ATOM   3996  CG  TYR A 510      -1.184   8.693  -3.002  1.00 15.00       A    C
ATOM   3997  CD1 TYR A 510      -2.410   8.131  -2.665  1.00 15.00       A    C
ATOM   3998  CD2 TYR A 510      -0.945   8.989  -4.336  1.00 15.00       A    C
ATOM   3999  CE1 TYR A 510      -3.366   7.873  -3.629  1.00 15.00       A    C
ATOM   4000  CE2 TYR A 510      -1.895   8.738  -5.306  1.00 15.00       A    C
ATOM   4001  CZ  TYR A 510      -3.102   8.180  -4.948  1.00 15.00       A    C
ATOM   4002  OH  TYR A 510      -4.049   7.923  -5.913  1.00 15.00       A    O
ATOM   4003  C   TYR A 510       0.383   9.971   0.258  1.00 15.00       A    C
ATOM   4004  O   TYR A 510       1.509  10.466   0.354  1.00 15.00       A    O
ATOM   4005  N   SER A 511      -0.188   9.283   1.237  1.00 15.00       A    N
ATOM   4006  CA  SER A 511       0.502   9.024   2.490  1.00 15.00       A    C
ATOM   4007  CB  SER A 511      -0.508   8.642   3.573  1.00 15.00       A    C
ATOM   4008  OG  SER A 511      -1.581   7.898   3.017  1.00 15.00       A    O
ATOM   4009  C   SER A 511       1.518   7.904   2.285  1.00 15.00       A    C
ATOM   4010  O   SER A 511       1.287   6.983   1.495  1.00 15.00       A    O
ATOM   4011  N   PHE A 512       2.635   7.980   2.983  1.00 15.00       A    N
ATOM   4012  CA  PHE A 512       3.679   6.980   2.846  1.00 15.00       A    C
ATOM   4013  CB  PHE A 512       5.009   7.656   2.507  1.00 15.00       A    C
ATOM   4014  CG  PHE A 512       5.940   6.800   1.699  1.00 15.00       A    C
ATOM   4015  CD1 PHE A 512       5.608   6.415   0.411  1.00 15.00       A    C
ATOM   4016  CD2 PHE A 512       7.149   6.382   2.226  1.00 15.00       A    C
ATOM   4017  CE1 PHE A 512       6.464   5.628  -0.336  1.00 15.00       A    C
ATOM   4018  CE2 PHE A 512       8.010   5.596   1.486  1.00 15.00       A    C
ATOM   4019  CZ  PHE A 512       7.667   5.218   0.203  1.00 15.00       A    C
ATOM   4020  C   PHE A 512       3.810   6.147   4.116  1.00 15.00       A    C
ATOM   4021  O   PHE A 512       4.236   4.993   4.067  1.00 15.00       A    O
ATOM   4022  N   ILE A 513       3.413   6.733   5.243  1.00 15.00       A    N
ATOM   4023  CA  ILE A 513       3.486   6.071   6.548  1.00 15.00       A    C
ATOM   4024  CB  ILE A 513       2.918   6.973   7.673  1.00 15.00       A    C
ATOM   4025  CG1 ILE A 513       3.290   6.434   9.058  1.00 15.00       A    C
ATOM   4026  CG2 ILE A 513       1.409   7.146   7.544  1.00 15.00       A    C
ATOM   4027  CD1 ILE A 513       4.777   6.461   9.345  1.00 15.00       A    C
ATOM   4028  C   ILE A 513       2.790   4.705   6.560  1.00 15.00       A    C
ATOM   4029  O   ILE A 513       3.176   3.813   7.318  1.00 15.00       A    O
ATOM   4030  N   ARG A 514       1.789   4.542   5.697  1.00 15.00       A    N
ATOM   4031  CA  ARG A 514       1.028   3.295   5.597  1.00 15.00       A    C
ATOM   4032  CB  ARG A 514      -0.033   3.411   4.501  1.00 15.00       A    C
ATOM   4033  CG  ARG A 514       0.525   3.389   3.086  1.00 15.00       A    C
ATOM   4034  CD  ARG A 514      -0.329   4.213   2.143  1.00 15.00       A    C
ATOM   4035  NE  ARG A 514      -1.732   3.805   2.165  1.00 15.00       A    N
ATOM   4036  CZ  ARG A 514      -2.722   4.558   1.696  1.00 15.00       A    C
```

ATOM	4037	NH1	ARG	A	514	-2.459	5.753	1.180	1.00	15.00	A	N
ATOM	4038	NH2	ARG	A	514	-3.974	4.116	1.745	1.00	15.00	A	N
ATOM	4039	C	ARG	A	514	1.923	2.091	5.313	1.00	15.00	A	C
ATOM	4040	O	ARG	A	514	1.558	0.954	5.609	1.00	15.00	A	O
ATOM	4041	N	TYR	A	515	3.093	2.340	4.740	1.00	15.00	A	N
ATOM	4042	CA	TYR	A	515	4.019	1.269	4.417	1.00	15.00	A	C
ATOM	4043	CB	TYR	A	515	4.948	1.682	3.274	1.00	15.00	A	C
ATOM	4044	CG	TYR	A	515	4.217	1.891	1.965	1.00	15.00	A	C
ATOM	4045	CD1	TYR	A	515	3.762	0.807	1.227	1.00	15.00	A	C
ATOM	4046	CD2	TYR	A	515	3.970	3.168	1.473	1.00	15.00	A	C
ATOM	4047	CE1	TYR	A	515	3.084	0.985	0.038	1.00	15.00	A	C
ATOM	4048	CE2	TYR	A	515	3.292	3.355	0.281	1.00	15.00	A	C
ATOM	4049	CZ	TYR	A	515	2.851	2.259	-0.431	1.00	15.00	A	C
ATOM	4050	OH	TYR	A	515	2.172	2.432	-1.613	1.00	15.00	A	O
ATOM	4051	C	TYR	A	515	4.801	0.836	5.648	1.00	15.00	A	C
ATOM	4052	O	TYR	A	515	5.319	-0.278	5.707	1.00	15.00	A	O
ATOM	4053	N	TYR	A	516	4.869	1.713	6.638	1.00	15.00	A	N
ATOM	4054	CA	TYR	A	516	5.572	1.406	7.876	1.00	15.00	A	C
ATOM	4055	CB	TYR	A	516	6.034	2.696	8.568	1.00	15.00	A	C
ATOM	4056	CG	TYR	A	516	6.769	2.472	9.873	1.00	15.00	A	C
ATOM	4057	CD1	TYR	A	516	6.128	2.654	11.092	1.00	15.00	A	C
ATOM	4058	CD2	TYR	A	516	8.102	2.075	9.888	1.00	15.00	A	C
ATOM	4059	CE1	TYR	A	516	6.790	2.448	12.286	1.00	15.00	A	C
ATOM	4060	CE2	TYR	A	516	8.772	1.867	11.080	1.00	15.00	A	C
ATOM	4061	CZ	TYR	A	516	8.110	2.055	12.276	1.00	15.00	A	C
ATOM	4062	OH	TYR	A	516	8.768	1.844	13.464	1.00	15.00	A	O
ATOM	4063	C	TYR	A	516	4.653	0.605	8.794	1.00	15.00	A	C
ATOM	4064	O	TYR	A	516	5.065	-0.390	9.394	1.00	15.00	A	O
ATOM	4065	N	THR	A	517	3.402	1.038	8.873	1.00	15.00	A	N
ATOM	4066	CA	THR	A	517	2.405	0.381	9.702	1.00	15.00	A	C
ATOM	4067	CB	THR	A	517	1.128	1.243	9.767	1.00	15.00	A	C
ATOM	4068	OG1	THR	A	517	1.096	2.115	8.627	1.00	15.00	A	O
ATOM	4069	CG2	THR	A	517	1.123	2.082	11.034	1.00	15.00	A	C
ATOM	4070	C	THR	A	517	2.064	-1.008	9.157	1.00	15.00	A	C
ATOM	4071	O	THR	A	517	2.046	-1.993	9.899	1.00	15.00	A	O
ATOM	4072	N	ARG	A	518	1.821	-1.070	7.852	1.00	15.00	A	N
ATOM	4073	CA	ARG	A	518	1.475	-2.315	7.173	1.00	15.00	A	C
ATOM	4074	CB	ARG	A	518	1.319	-2.061	5.676	1.00	15.00	A	C
ATOM	4075	CG	ARG	A	518	1.122	-3.309	4.843	1.00	15.00	A	C
ATOM	4076	CD	ARG	A	518	2.105	-3.340	3.689	1.00	15.00	A	C
ATOM	4077	NE	ARG	A	518	3.299	-4.131	3.995	1.00	15.00	A	N
ATOM	4078	CZ	ARG	A	518	4.167	-4.548	3.083	1.00	15.00	A	C
ATOM	4079	NH1	ARG	A	518	3.998	-4.270	1.790	1.00	15.00	A	N
ATOM	4080	NH2	ARG	A	518	5.213	-5.245	3.470	1.00	15.00	A	N
ATOM	4081	C	ARG	A	518	2.513	-3.405	7.411	1.00	15.00	A	C
ATOM	4082	O	ARG	A	518	2.161	-4.546	7.713	1.00	15.00	A	O
ATOM	4083	N	THR	A	519	3.785	-3.050	7.257	1.00	15.00	A	N
ATOM	4084	CA	THR	A	519	4.877	-3.994	7.457	1.00	15.00	A	C
ATOM	4085	CB	THR	A	519	6.243	-3.322	7.204	1.00	15.00	A	C
ATOM	4086	OG1	THR	A	519	6.262	-2.770	5.882	1.00	15.00	A	O
ATOM	4087	CG2	THR	A	519	7.372	-4.329	7.341	1.00	15.00	A	C
ATOM	4088	C	THR	A	519	4.844	-4.617	8.857	1.00	15.00	A	C
ATOM	4089	O	THR	A	519	4.975	-5.833	9.009	1.00	15.00	A	O
ATOM	4090	N	LEU	A	520	4.653	-3.784	9.875	1.00	15.00	A	N
ATOM	4091	CA	LEU	A	520	4.607	-4.266	11.252	1.00	15.00	A	C
ATOM	4092	CB	LEU	A	520	4.735	-3.107	12.244	1.00	15.00	A	C
ATOM	4093	CG	LEU	A	520	6.163	-2.687	12.630	1.00	15.00	A	C
ATOM	4094	CD1	LEU	A	520	7.013	-2.402	11.401	1.00	15.00	A	C
ATOM	4095	CD2	LEU	A	520	6.134	-1.474	13.543	1.00	15.00	A	C
ATOM	4096	C	LEU	A	520	3.342	-5.082	11.521	1.00	15.00	A	C
ATOM	4097	O	LEU	A	520	3.392	-6.110	12.201	1.00	15.00	A	O
ATOM	4098	N	TYR	A	521	2.216	-4.631	10.971	1.00	15.00	A	N
ATOM	4099	CA	TYR	A	521	0.944	-5.329	11.144	1.00	15.00	A	C
ATOM	4100	CB	TYR	A	521	-0.197	-4.573	10.451	1.00	15.00	A	C
ATOM	4101	CG	TYR	A	521	-0.577	-3.253	11.085	1.00	15.00	A	C
ATOM	4102	CD1	TYR	A	521	-0.441	-3.042	12.451	1.00	15.00	A	C
ATOM	4103	CD2	TYR	A	521	-1.079	-2.217	10.309	1.00	15.00	A	C
ATOM	4104	CE1	TYR	A	521	-0.794	-1.835	13.025	1.00	15.00	A	C
ATOM	4105	CE2	TYR	A	521	-1.432	-1.009	10.872	1.00	15.00	A	C
ATOM	4106	CZ	TYR	A	521	-1.288	-0.823	12.230	1.00	15.00	A	C
ATOM	4107	OH	TYR	A	521	-1.641	0.381	12.793	1.00	15.00	A	O

ATOM	4108	C	TYR	A	521	1.021	-6.737	10.566	1.00	15.00	A	C
ATOM	4109	O	TYR	A	521	0.586	-7.701	11.194	1.00	15.00	A	O
ATOM	4110	N	GLN	A	522	1.590	-6.838	9.370	1.00	15.00	A	N
ATOM	4111	CA	GLN	A	522	1.722	-8.112	8.674	1.00	15.00	A	C
ATOM	4112	CB	GLN	A	522	2.351	-7.904	7.286	1.00	15.00	A	C
ATOM	4113	CG	GLN	A	522	3.803	-8.343	7.154	1.00	15.00	A	C
ATOM	4114	CD	GLN	A	522	4.497	-7.729	5.960	1.00	15.00	A	C
ATOM	4115	OE1	GLN	A	522	4.271	-6.567	5.627	1.00	15.00	A	O
ATOM	4116	NE2	GLN	A	522	5.345	-8.508	5.310	1.00	15.00	A	N
ATOM	4117	C	GLN	A	522	2.507	-9.136	9.495	1.00	15.00	A	C
ATOM	4118	O	GLN	A	522	2.113	-10.299	9.590	1.00	15.00	A	O
ATOM	4119	N	PHE	A	523	3.600	-8.697	10.108	1.00	15.00	A	N
ATOM	4120	CA	PHE	A	523	4.423	-9.592	10.904	1.00	15.00	A	C
ATOM	4121	CB	PHE	A	523	5.794	-8.984	11.193	1.00	15.00	A	C
ATOM	4122	CG	PHE	A	523	6.709	-9.017	10.001	1.00	15.00	A	C
ATOM	4123	CD1	PHE	A	523	7.055	-10.222	9.413	1.00	15.00	A	C
ATOM	4124	CD2	PHE	A	523	7.215	-7.847	9.464	1.00	15.00	A	C
ATOM	4125	CE1	PHE	A	523	7.888	-10.260	8.312	1.00	15.00	A	C
ATOM	4126	CE2	PHE	A	523	8.050	-7.878	8.364	1.00	15.00	A	C
ATOM	4127	CZ	PHE	A	523	8.387	-9.085	7.787	1.00	15.00	A	C
ATOM	4128	C	PHE	A	523	3.701	-9.990	12.177	1.00	15.00	A	C
ATOM	4129	O	PHE	A	523	3.837	-11.116	12.655	1.00	15.00	A	O
ATOM	4130	N	GLN	A	524	2.917	-9.062	12.710	1.00	15.00	A	N
ATOM	4131	CA	GLN	A	524	2.142	-9.321	13.909	1.00	15.00	A	C
ATOM	4132	CB	GLN	A	524	1.484	-8.032	14.414	1.00	15.00	A	C
ATOM	4133	CG	GLN	A	524	2.396	-7.147	15.248	1.00	15.00	A	C
ATOM	4134	CD	GLN	A	524	1.704	-5.886	15.732	1.00	15.00	A	C
ATOM	4135	OE1	GLN	A	524	1.789	-4.832	15.103	1.00	15.00	A	O
ATOM	4136	NE2	GLN	A	524	1.011	-5.984	16.857	1.00	15.00	A	N
ATOM	4137	C	GLN	A	524	1.076	-10.369	13.604	1.00	15.00	A	C
ATOM	4138	O	GLN	A	524	0.891	-11.322	14.362	1.00	15.00	A	O
ATOM	4139	N	PHE	A	525	0.392	-10.188	12.475	1.00	15.00	A	N
ATOM	4140	CA	PHE	A	525	-0.655	-11.110	12.047	1.00	15.00	A	C
ATOM	4141	CB	PHE	A	525	-1.293	-10.646	10.732	1.00	15.00	A	C
ATOM	4142	CG	PHE	A	525	-2.159	-9.425	10.848	1.00	15.00	A	C
ATOM	4143	CD1	PHE	A	525	-2.943	-9.213	11.969	1.00	15.00	A	C
ATOM	4144	CD2	PHE	A	525	-2.193	-8.490	9.827	1.00	15.00	A	C
ATOM	4145	CE1	PHE	A	525	-3.743	-8.091	12.071	1.00	15.00	A	C
ATOM	4146	CE2	PHE	A	525	-2.989	-7.366	9.922	1.00	15.00	A	C
ATOM	4147	CZ	PHE	A	525	-3.765	-7.166	11.046	1.00	15.00	A	C
ATOM	4148	C	PHE	A	525	-0.104	-12.517	11.865	1.00	15.00	A	C
ATOM	4149	O	PHE	A	525	-0.620	-13.471	12.447	1.00	15.00	A	O
ATOM	4150	N	GLN	A	526	0.951	-12.630	11.062	1.00	15.00	A	N
ATOM	4151	CA	GLN	A	526	1.573	-13.919	10.785	1.00	15.00	A	C
ATOM	4152	CB	GLN	A	526	2.787	-13.754	9.865	1.00	15.00	A	C
ATOM	4153	CG	GLN	A	526	3.368	-15.065	9.346	1.00	15.00	A	C
ATOM	4154	CD	GLN	A	526	2.438	-15.802	8.396	1.00	15.00	A	C
ATOM	4155	OE1	GLN	A	526	1.216	-15.703	8.491	1.00	15.00	A	O
ATOM	4156	NE2	GLN	A	526	3.015	-16.551	7.469	1.00	15.00	A	N
ATOM	4157	C	GLN	A	526	1.959	-14.651	12.067	1.00	15.00	A	C
ATOM	4158	O	GLN	A	526	1.633	-15.823	12.238	1.00	15.00	A	O
ATOM	4159	N	GLU	A	527	2.625	-13.949	12.978	1.00	15.00	A	N
ATOM	4160	CA	GLU	A	527	3.052	-14.549	14.238	1.00	15.00	A	C
ATOM	4161	CB	GLU	A	527	3.839	-13.542	15.079	1.00	15.00	A	C
ATOM	4162	CG	GLU	A	527	4.628	-14.171	16.216	1.00	15.00	A	C
ATOM	4163	CD	GLU	A	527	5.280	-13.140	17.111	1.00	15.00	A	C
ATOM	4164	OE1	GLU	A	527	4.551	-12.411	17.811	1.00	15.00	A	O
ATOM	4165	OE2	GLU	A	527	6.529	-13.055	17.124	1.00	15.00	A	O
ATOM	4166	C	GLU	A	527	1.863	-15.094	15.030	1.00	15.00	A	C
ATOM	4167	O	GLU	A	527	1.888	-16.234	15.501	1.00	15.00	A	O
ATOM	4168	N	ALA	A	528	0.813	-14.286	15.144	1.00	15.00	A	N
ATOM	4169	CA	ALA	A	528	-0.384	-14.680	15.880	1.00	15.00	A	C
ATOM	4170	CB	ALA	A	528	-1.360	-13.520	15.969	1.00	15.00	A	C
ATOM	4171	C	ALA	A	528	-1.054	-15.896	15.246	1.00	15.00	A	C
ATOM	4172	O	ALA	A	528	-1.542	-16.786	15.947	1.00	15.00	A	O
ATOM	4173	N	LEU	A	529	-1.074	-15.932	13.921	1.00	15.00	A	N
ATOM	4174	CA	LEU	A	529	-1.679	-17.043	13.201	1.00	15.00	A	C
ATOM	4175	CB	LEU	A	529	-1.886	-16.693	11.726	1.00	15.00	A	C
ATOM	4176	CG	LEU	A	529	-2.745	-15.458	11.438	1.00	15.00	A	C
ATOM	4177	CD1	LEU	A	529	-2.844	-15.210	9.941	1.00	15.00	A	C
ATOM	4178	CD2	LEU	A	529	-4.129	-15.597	12.056	1.00	15.00	A	C

ATOM	4179	C	LEU	A	529	-0.832	-18.303	13.344	1.00	15.00	A	C
ATOM	4180	O	LEU	A	529	-1.362	-19.392	13.563	1.00	15.00	A	O
ATOM	4181	N	CYS	A	530	0.484	-18.144	13.237	1.00	15.00	A	N
ATOM	4182	CA	CYS	A	530	1.411	-19.266	13.364	1.00	15.00	A	C
ATOM	4183	C	CYS	A	530	1.295	-19.920	14.736	1.00	15.00	A	C
ATOM	4184	O	CYS	A	530	1.395	-21.142	14.861	1.00	15.00	A	O
ATOM	4185	CB	CYS	A	530	2.849	-18.810	13.117	1.00	15.00	A	C
ATOM	4186	SG	CYS	A	530	3.163	-18.219	11.425	1.00	15.00	A	S
ATOM	4187	N	GLN	A	531	1.082	-19.105	15.766	1.00	15.00	A	N
ATOM	4188	CA	GLN	A	531	0.933	-19.622	17.120	1.00	15.00	A	C
ATOM	4189	CB	GLN	A	531	0.876	-18.477	18.139	1.00	15.00	A	C
ATOM	4190	CG	GLN	A	531	0.939	-18.929	19.592	1.00	15.00	A	C
ATOM	4191	CD	GLN	A	531	1.057	-17.768	20.563	1.00	15.00	A	C
ATOM	4192	OE1	GLN	A	531	2.154	-17.337	20.902	1.00	15.00	A	O
ATOM	4193	NE2	GLN	A	531	-0.076	-17.262	21.024	1.00	15.00	A	N
ATOM	4194	C	GLN	A	531	-0.322	-20.488	17.213	1.00	15.00	A	C
ATOM	4195	O	GLN	A	531	-0.326	-21.528	17.871	1.00	15.00	A	O
ATOM	4196	N	ALA	A	532	-1.378	-20.061	16.526	1.00	15.00	A	N
ATOM	4197	CA	ALA	A	532	-2.641	-20.790	16.524	1.00	15.00	A	C
ATOM	4198	CB	ALA	A	532	-3.768	-19.897	16.025	1.00	15.00	A	C
ATOM	4199	C	ALA	A	532	-2.551	-22.061	15.683	1.00	15.00	A	C
ATOM	4200	O	ALA	A	532	-3.057	-23.113	16.080	1.00	15.00	A	O
ATOM	4201	N	ALA	A	533	-1.881	-21.959	14.535	1.00	15.00	A	N
ATOM	4202	CA	ALA	A	533	-1.719	-23.087	13.618	1.00	15.00	A	C
ATOM	4203	CB	ALA	A	533	-1.255	-22.595	12.256	1.00	15.00	A	C
ATOM	4204	C	ALA	A	533	-0.744	-24.126	14.163	1.00	15.00	A	C
ATOM	4205	O	ALA	A	533	-0.569	-25.192	13.572	1.00	15.00	A	O
ATOM	4206	N	LYS	A	534	-0.108	-23.796	15.285	1.00	15.00	A	N
ATOM	4207	CA	LYS	A	534	0.848	-24.686	15.939	1.00	15.00	A	C
ATOM	4208	CB	LYS	A	534	0.194	-26.013	16.335	1.00	15.00	A	C
ATOM	4209	CG	LYS	A	534	-1.087	-25.842	17.134	1.00	15.00	A	C
ATOM	4210	CD	LYS	A	534	-1.953	-27.088	17.080	1.00	15.00	A	C
ATOM	4211	CE	LYS	A	534	-3.379	-26.774	17.504	1.00	15.00	A	C
ATOM	4212	NZ	LYS	A	534	-3.418	-25.981	18.764	1.00	15.00	A	N
ATOM	4213	C	LYS	A	534	2.078	-24.911	15.070	1.00	15.00	A	C
ATOM	4214	O	LYS	A	534	2.348	-26.023	14.613	1.00	15.00	A	O
ATOM	4215	N	HIS	A	535	2.810	-23.838	14.826	1.00	15.00	A	N
ATOM	4216	CA	HIS	A	535	4.010	-23.913	14.021	1.00	15.00	A	C
ATOM	4217	CB	HIS	A	535	4.099	-22.739	13.040	1.00	15.00	A	C
ATOM	4218	CG	HIS	A	535	5.416	-22.670	12.333	1.00	15.00	A	C
ATOM	4219	ND1	HIS	A	535	6.301	-21.648	12.571	1.00	15.00	A	N
ATOM	4220	CD2	HIS	A	535	5.968	-23.544	11.455	1.00	15.00	A	C
ATOM	4221	CE1	HIS	A	535	7.367	-21.921	11.850	1.00	15.00	A	C
ATOM	4222	NE2	HIS	A	535	7.211	-23.057	11.159	1.00	15.00	A	N
ATOM	4223	C	HIS	A	535	5.256	-23.974	14.896	1.00	15.00	A	C
ATOM	4224	O	HIS	A	535	5.560	-23.034	15.631	1.00	15.00	A	O
ATOM	4225	N	GLU	A	536	5.967	-25.084	14.801	1.00	15.00	A	N
ATOM	4226	CA	GLU	A	536	7.189	-25.280	15.559	1.00	15.00	A	C
ATOM	4227	CB	GLU	A	536	7.295	-26.737	16.019	1.00	15.00	A	C
ATOM	4228	CG	GLU	A	536	6.111	-27.230	16.836	1.00	15.00	A	C
ATOM	4229	CD	GLU	A	536	6.043	-26.599	18.213	1.00	15.00	A	C
ATOM	4230	OE1	GLU	A	536	7.114	-26.281	18.775	1.00	15.00	A	O
ATOM	4231	OE2	GLU	A	536	4.921	-26.434	18.736	1.00	15.00	A	O
ATOM	4232	C	GLU	A	536	8.392	-24.935	14.690	1.00	15.00	A	C
ATOM	4233	O	GLU	A	536	8.580	-25.517	13.617	1.00	15.00	A	O
ATOM	4234	N	GLY	A	537	9.187	-23.974	15.133	1.00	15.00	A	N
ATOM	4235	CA	GLY	A	537	10.363	-23.590	14.383	1.00	15.00	A	C
ATOM	4236	C	GLY	A	537	10.447	-22.098	14.135	1.00	15.00	A	C
ATOM	4237	O	GLY	A	537	9.766	-21.317	14.798	1.00	15.00	A	O
ATOM	4238	N	PRO	A	538	11.289	-21.674	13.180	1.00	15.00	A	N
ATOM	4239	CA	PRO	A	538	11.468	-20.261	12.841	1.00	15.00	A	C
ATOM	4240	CB	PRO	A	538	12.721	-20.251	11.952	1.00	15.00	A	C
ATOM	4241	CG	PRO	A	538	13.285	-21.632	12.028	1.00	15.00	A	C
ATOM	4242	CD	PRO	A	538	12.136	-22.536	12.352	1.00	15.00	A	C
ATOM	4243	C	PRO	A	538	10.273	-19.711	12.071	1.00	15.00	A	C
ATOM	4244	O	PRO	A	538	9.771	-20.352	11.145	1.00	15.00	A	O
ATOM	4245	N	LEU	A	539	9.848	-18.508	12.445	1.00	15.00	A	N
ATOM	4246	CA	LEU	A	539	8.697	-17.845	11.833	1.00	15.00	A	C
ATOM	4247	CB	LEU	A	539	8.535	-16.435	12.408	1.00	15.00	A	C
ATOM	4248	CG	LEU	A	539	7.156	-15.792	12.246	1.00	15.00	A	C
ATOM	4249	CD1	LEU	A	539	6.104	-16.587	13.003	1.00	15.00	A	C

```
ATOM   4250  CD2 LEU A 539       7.179 -14.347  12.718  1.00 15.00       A    C
ATOM   4251  C   LEU A 539       8.763 -17.783  10.304  1.00 15.00       A    C
ATOM   4252  O   LEU A 539       7.730 -17.831   9.636  1.00 15.00       A    O
ATOM   4253  N   HIS A 540       9.965 -17.694   9.747  1.00 15.00       A    N
ATOM   4254  CA  HIS A 540      10.120 -17.608   8.295  1.00 15.00       A    C
ATOM   4255  CB  HIS A 540      11.506 -17.091   7.886  1.00 15.00       A    C
ATOM   4256  CG  HIS A 540      12.658 -17.941   8.325  1.00 15.00       A    C
ATOM   4257  ND1 HIS A 540      13.425 -17.601   9.415  1.00 15.00       A    N
ATOM   4258  CD2 HIS A 540      13.144 -19.079   7.777  1.00 15.00       A    C
ATOM   4259  CE1 HIS A 540      14.357 -18.531   9.502  1.00 15.00       A    C
ATOM   4260  NE2 HIS A 540      14.223 -19.442   8.535  1.00 15.00       A    N
ATOM   4261  C   HIS A 540       9.750 -18.905   7.567  1.00 15.00       A    C
ATOM   4262  O   HIS A 540       9.539 -18.904   6.357  1.00 15.00       A    O
ATOM   4263  N   LYS A 541       9.663 -20.006   8.307  1.00 15.00       A    N
ATOM   4264  CA  LYS A 541       9.303 -21.293   7.717  1.00 15.00       A    C
ATOM   4265  CB  LYS A 541      10.245 -22.395   8.209  1.00 15.00       A    C
ATOM   4266  CG  LYS A 541      11.693 -22.227   7.790  1.00 15.00       A    C
ATOM   4267  CD  LYS A 541      12.558 -23.319   8.395  1.00 15.00       A    C
ATOM   4268  CE  LYS A 541      12.492 -24.596   7.574  1.00 15.00       A    C
ATOM   4269  NZ  LYS A 541      13.375 -24.527   6.386  1.00 15.00       A    N
ATOM   4270  C   LYS A 541       7.869 -21.656   8.084  1.00 15.00       A    C
ATOM   4271  O   LYS A 541       7.452 -22.811   7.955  1.00 15.00       A    O
ATOM   4272  N   CYS A 542       7.124 -20.666   8.551  1.00 15.00       A    N
ATOM   4273  CA  CYS A 542       5.743 -20.871   8.957  1.00 15.00       A    C
ATOM   4274  C   CYS A 542       4.789 -20.988   7.775  1.00 15.00       A    C
ATOM   4275  O   CYS A 542       5.010 -20.414   6.711  1.00 15.00       A    O
ATOM   4276  CB  CYS A 542       5.270 -19.761   9.894  1.00 15.00       A    C
ATOM   4277  SG  CYS A 542       3.524 -19.919  10.391  1.00 15.00       A    S
ATOM   4278  N   ASP A 543       3.714 -21.725   7.998  1.00 15.00       A    N
ATOM   4279  CA  ASP A 543       2.683 -21.945   6.999  1.00 15.00       A    C
ATOM   4280  CB  ASP A 543       3.066 -23.104   6.080  1.00 15.00       A    C
ATOM   4281  CG  ASP A 543       1.919 -23.562   5.209  1.00 15.00       A    C
ATOM   4282  OD1 ASP A 543       1.128 -22.706   4.766  1.00 15.00       A    O
ATOM   4283  OD2 ASP A 543       1.807 -24.779   4.979  1.00 15.00       A    O
ATOM   4284  C   ASP A 543       1.375 -22.237   7.723  1.00 15.00       A    C
ATOM   4285  O   ASP A 543       1.328 -23.115   8.587  1.00 15.00       A    O
ATOM   4286  N   ILE A 544       0.330 -21.496   7.378  1.00 15.00       A    N
ATOM   4287  CA  ILE A 544      -0.968 -21.641   8.029  1.00 15.00       A    C
ATOM   4288  CB  ILE A 544      -1.620 -20.266   8.289  1.00 15.00       A    C
ATOM   4289  CG1 ILE A 544      -1.693 -19.452   6.990  1.00 15.00       A    C
ATOM   4290  CG2 ILE A 544      -0.847 -19.508   9.360  1.00 15.00       A    C
ATOM   4291  CD1 ILE A 544      -2.423 -18.133   7.128  1.00 15.00       A    C
ATOM   4292  C   ILE A 544      -1.940 -22.520   7.245  1.00 15.00       A    C
ATOM   4293  O   ILE A 544      -3.106 -22.643   7.624  1.00 15.00       A    O
ATOM   4294  N   SER A 545      -1.472 -23.121   6.157  1.00 15.00       A    N
ATOM   4295  CA  SER A 545      -2.319 -23.988   5.345  1.00 15.00       A    C
ATOM   4296  CB  SER A 545      -1.538 -24.531   4.146  1.00 15.00       A    C
ATOM   4297  OG  SER A 545      -0.915 -23.482   3.428  1.00 15.00       A    O
ATOM   4298  C   SER A 545      -2.878 -25.147   6.170  1.00 15.00       A    C
ATOM   4299  O   SER A 545      -2.174 -25.718   7.008  1.00 15.00       A    O
ATOM   4300  N   ASN A 546      -4.154 -25.464   5.940  1.00 15.00       A    N
ATOM   4301  CA  ASN A 546      -4.844 -26.560   6.632  1.00 15.00       A    C
ATOM   4302  CB  ASN A 546      -4.070 -27.885   6.542  1.00 15.00       A    C
ATOM   4303  CG  ASN A 546      -4.170 -28.544   5.180  1.00 15.00       A    C
ATOM   4304  OD1 ASN A 546      -5.179 -28.424   4.488  1.00 15.00       A    O
ATOM   4305  ND2 ASN A 546      -3.120 -29.248   4.783  1.00 15.00       A    N
ATOM   4306  C   ASN A 546      -5.205 -26.238   8.083  1.00 15.00       A    C
ATOM   4307  O   ASN A 546      -5.704 -27.102   8.803  1.00 15.00       A    O
ATOM   4308  N   SER A 547      -4.961 -25.008   8.517  1.00 15.00       A    N
ATOM   4309  CA  SER A 547      -5.283 -24.618   9.883  1.00 15.00       A    C
ATOM   4310  CB  SER A 547      -4.122 -23.874  10.546  1.00 15.00       A    C
ATOM   4311  OG  SER A 547      -4.475 -23.427  11.849  1.00 15.00       A    O
ATOM   4312  C   SER A 547      -6.555 -23.782   9.939  1.00 15.00       A    C
ATOM   4313  O   SER A 547      -6.580 -22.626   9.502  1.00 15.00       A    O
ATOM   4314  N   THR A 548      -7.607 -24.374  10.477  1.00 15.00       A    N
ATOM   4315  CA  THR A 548      -8.887 -23.703  10.610  1.00 15.00       A    C
ATOM   4316  CB  THR A 548      -9.985 -24.725  10.946  1.00 15.00       A    C
ATOM   4317  OG1 THR A 548      -9.369 -25.971  11.317  1.00 15.00       A    O
ATOM   4318  CG2 THR A 548     -10.887 -24.947   9.742  1.00 15.00       A    C
ATOM   4319  C   THR A 548      -8.825 -22.657  11.719  1.00 15.00       A    C
ATOM   4320  O   THR A 548      -9.492 -21.623  11.657  1.00 15.00       A    O
```

ATOM	4321	N	GLU	A	549	-8.014	-22.943	12.729	1.00	15.00	A	N
ATOM	4322	CA	GLU	A	549	-7.833	-22.052	13.868	1.00	15.00	A	C
ATOM	4323	CB	GLU	A	549	-6.848	-22.657	14.879	1.00	15.00	A	C
ATOM	4324	CG	GLU	A	549	-7.062	-24.136	15.176	1.00	15.00	A	C
ATOM	4325	CD	GLU	A	549	-6.511	-25.034	14.085	1.00	15.00	A	C
ATOM	4326	OE1	GLU	A	549	-5.290	-25.276	14.075	1.00	15.00	A	O
ATOM	4327	OE2	GLU	A	549	-7.302	-25.475	13.222	1.00	15.00	A	O
ATOM	4328	C	GLU	A	549	-7.326	-20.694	13.403	1.00	15.00	A	C
ATOM	4329	O	GLU	A	549	-7.855	-19.651	13.797	1.00	15.00	A	O
ATOM	4330	N	ALA	A	550	-6.302	-20.718	12.556	1.00	15.00	A	N
ATOM	4331	CA	ALA	A	550	-5.722	-19.495	12.020	1.00	15.00	A	C
ATOM	4332	CB	ALA	A	550	-4.475	-19.812	11.210	1.00	15.00	A	C
ATOM	4333	C	ALA	A	550	-6.742	-18.756	11.162	1.00	15.00	A	C
ATOM	4334	O	ALA	A	550	-6.917	-17.543	11.294	1.00	15.00	A	O
ATOM	4335	N	GLY	A	551	-7.422	-19.502	10.296	1.00	15.00	A	N
ATOM	4336	CA	GLY	A	551	-8.423	-18.916	9.424	1.00	15.00	A	C
ATOM	4337	C	GLY	A	551	-9.544	-18.252	10.200	1.00	15.00	A	C
ATOM	4338	O	GLY	A	551	-9.931	-17.123	9.898	1.00	15.00	A	O
ATOM	4339	N	GLN	A	552	-10.053	-18.948	11.211	1.00	15.00	A	N
ATOM	4340	CA	GLN	A	552	-11.131	-18.424	12.040	1.00	15.00	A	C
ATOM	4341	CB	GLN	A	552	-11.618	-19.489	13.029	1.00	15.00	A	C
ATOM	4342	CG	GLN	A	552	-12.706	-19.010	13.980	1.00	15.00	A	C
ATOM	4343	CD	GLN	A	552	-13.991	-18.641	13.265	1.00	15.00	A	C
ATOM	4344	OE1	GLN	A	552	-14.891	-19.464	13.119	1.00	15.00	A	O
ATOM	4345	NE2	GLN	A	552	-14.082	-17.401	12.809	1.00	15.00	A	N
ATOM	4346	C	GLN	A	552	-10.687	-17.171	12.791	1.00	15.00	A	C
ATOM	4347	O	GLN	A	552	-11.433	-16.193	12.885	1.00	15.00	A	O
ATOM	4348	N	LYS	A	553	-9.467	-17.204	13.317	1.00	15.00	A	N
ATOM	4349	CA	LYS	A	553	-8.923	-16.075	14.059	1.00	15.00	A	C
ATOM	4350	CB	LYS	A	553	-7.568	-16.438	14.672	1.00	15.00	A	C
ATOM	4351	CG	LYS	A	553	-7.071	-15.455	15.721	1.00	15.00	A	C
ATOM	4352	CD	LYS	A	553	-6.603	-16.180	16.973	1.00	15.00	A	C
ATOM	4353	CE	LYS	A	553	-5.947	-15.227	17.960	1.00	15.00	A	C
ATOM	4354	NZ	LYS	A	553	-6.857	-14.119	18.365	1.00	15.00	A	N
ATOM	4355	C	LYS	A	553	-8.795	-14.849	13.162	1.00	15.00	A	C
ATOM	4356	O	LYS	A	553	-9.123	-13.734	13.569	1.00	15.00	A	O
ATOM	4357	N	LEU	A	554	-8.329	-15.067	11.939	1.00	15.00	A	N
ATOM	4358	CA	LEU	A	554	-8.162	-13.985	10.979	1.00	15.00	A	C
ATOM	4359	CB	LEU	A	554	-7.330	-14.458	9.784	1.00	15.00	A	C
ATOM	4360	CG	LEU	A	554	-6.929	-13.383	8.770	1.00	15.00	A	C
ATOM	4361	CD1	LEU	A	554	-5.952	-12.395	9.390	1.00	15.00	A	C
ATOM	4362	CD2	LEU	A	554	-6.336	-14.018	7.522	1.00	15.00	A	C
ATOM	4363	C	LEU	A	554	-9.517	-13.465	10.505	1.00	15.00	A	C
ATOM	4364	O	LEU	A	554	-9.732	-12.253	10.416	1.00	15.00	A	O
ATOM	4365	N	PHE	A	555	-10.433	-14.389	10.223	1.00	15.00	A	N
ATOM	4366	CA	PHE	A	555	-11.772	-14.040	9.754	1.00	15.00	A	C
ATOM	4367	CB	PHE	A	555	-12.590	-15.304	9.467	1.00	15.00	A	C
ATOM	4368	CG	PHE	A	555	-13.951	-15.041	8.882	1.00	15.00	A	C
ATOM	4369	CD1	PHE	A	555	-14.086	-14.599	7.575	1.00	15.00	A	C
ATOM	4370	CD2	PHE	A	555	-15.095	-15.238	9.639	1.00	15.00	A	C
ATOM	4371	CE1	PHE	A	555	-15.335	-14.359	7.035	1.00	15.00	A	C
ATOM	4372	CE2	PHE	A	555	-16.347	-14.998	9.106	1.00	15.00	A	C
ATOM	4373	CZ	PHE	A	555	-16.468	-14.558	7.801	1.00	15.00	A	C
ATOM	4374	C	PHE	A	555	-12.501	-13.150	10.756	1.00	15.00	A	C
ATOM	4375	O	PHE	A	555	-13.319	-12.313	10.373	1.00	15.00	A	O
ATOM	4376	N	ASN	A	556	-12.187	-13.320	12.035	1.00	15.00	A	N
ATOM	4377	CA	ASN	A	556	-12.810	-12.526	13.091	1.00	15.00	A	C
ATOM	4378	CB	ASN	A	556	-12.341	-12.976	14.475	1.00	15.00	A	C
ATOM	4379	CG	ASN	A	556	-13.088	-14.196	14.970	1.00	15.00	A	C
ATOM	4380	OD1	ASN	A	556	-14.239	-14.424	14.606	1.00	15.00	A	O
ATOM	4381	ND2	ASN	A	556	-12.437	-14.988	15.806	1.00	15.00	A	N
ATOM	4382	C	ASN	A	556	-12.545	-11.039	12.899	1.00	15.00	A	C
ATOM	4383	O	ASN	A	556	-13.342	-10.199	13.313	1.00	15.00	A	O
ATOM	4384	N	MET	A	557	-11.423	-10.719	12.270	1.00	15.00	A	N
ATOM	4385	CA	MET	A	557	-11.067	-9.333	12.007	1.00	15.00	A	C
ATOM	4386	CB	MET	A	557	-9.550	-9.138	12.105	1.00	15.00	A	C
ATOM	4387	CG	MET	A	557	-9.071	-7.727	11.784	1.00	15.00	A	C
ATOM	4388	SD	MET	A	557	-8.672	-7.493	10.039	1.00	15.00	A	S
ATOM	4389	CE	MET	A	557	-7.212	-8.518	9.884	1.00	15.00	A	C
ATOM	4390	C	MET	A	557	-11.569	-8.924	10.628	1.00	15.00	A	C
ATOM	4391	O	MET	A	557	-12.065	-7.813	10.437	1.00	15.00	A	O

ATOM	4392	N	LEU	A	558	-11.465	-9.847	9.678	1.00	15.00	A	N
ATOM	4393	CA	LEU	A	558	-11.893	-9.598	8.306	1.00	15.00	A	C
ATOM	4394	CB	LEU	A	558	-11.568	-10.799	7.411	1.00	15.00	A	C
ATOM	4395	CG	LEU	A	558	-10.123	-11.307	7.430	1.00	15.00	A	C
ATOM	4396	CD1	LEU	A	558	-9.966	-12.501	6.501	1.00	15.00	A	C
ATOM	4397	CD2	LEU	A	558	-9.149	-10.203	7.052	1.00	15.00	A	C
ATOM	4398	C	LEU	A	558	-13.386	-9.281	8.223	1.00	15.00	A	C
ATOM	4399	O	LEU	A	558	-13.811	-8.482	7.394	1.00	15.00	A	O
ATOM	4400	N	ARG	A	559	-14.176	-9.899	9.095	1.00	15.00	A	N
ATOM	4401	CA	ARG	A	559	-15.621	-9.686	9.101	1.00	15.00	A	C
ATOM	4402	CB	ARG	A	559	-16.345	-10.862	9.763	1.00	15.00	A	C
ATOM	4403	CG	ARG	A	559	-16.078	-11.000	11.254	1.00	15.00	A	C
ATOM	4404	CD	ARG	A	559	-17.346	-11.364	12.007	1.00	15.00	A	C
ATOM	4405	NE	ARG	A	559	-18.449	-10.464	11.667	1.00	15.00	A	N
ATOM	4406	CZ	ARG	A	559	-18.910	-9.504	12.470	1.00	15.00	A	C
ATOM	4407	NH1	ARG	A	559	-18.365	-9.313	13.669	1.00	15.00	A	N
ATOM	4408	NH2	ARG	A	559	-19.910	-8.726	12.066	1.00	15.00	A	N
ATOM	4409	C	ARG	A	559	-16.009	-8.370	9.777	1.00	15.00	A	C
ATOM	4410	O	ARG	A	559	-17.191	-8.013	9.830	1.00	15.00	A	O
ATOM	4411	N	LEU	A	560	-15.022	-7.661	10.305	1.00	15.00	A	N
ATOM	4412	CA	LEU	A	560	-15.270	-6.389	10.968	1.00	15.00	A	C
ATOM	4413	CB	LEU	A	560	-14.308	-6.192	12.145	1.00	15.00	A	C
ATOM	4414	CG	LEU	A	560	-14.449	-7.161	13.320	1.00	15.00	A	C
ATOM	4415	CD1	LEU	A	560	-13.372	-6.891	14.360	1.00	15.00	A	C
ATOM	4416	CD2	LEU	A	560	-15.832	-7.055	13.942	1.00	15.00	A	C
ATOM	4417	C	LEU	A	560	-15.110	-5.239	9.987	1.00	15.00	A	C
ATOM	4418	O	LEU	A	560	-15.941	-4.332	9.931	1.00	15.00	A	O
ATOM	4419	N	GLY	A	561	-14.048	-5.291	9.199	1.00	15.00	A	N
ATOM	4420	CA	GLY	A	561	-13.785	-4.232	8.252	1.00	15.00	A	C
ATOM	4421	C	GLY	A	561	-13.281	-3.006	8.978	1.00	15.00	A	C
ATOM	4422	O	GLY	A	561	-12.190	-3.026	9.546	1.00	15.00	A	O
ATOM	4423	N	LYS	A	562	-14.075	-1.947	8.988	1.00	15.00	A	N
ATOM	4424	CA	LYS	A	562	-13.686	-0.734	9.687	1.00	15.00	A	C
ATOM	4425	CB	LYS	A	562	-13.145	0.343	8.741	1.00	15.00	A	C
ATOM	4426	CG	LYS	A	562	-14.164	0.977	7.807	1.00	15.00	A	C
ATOM	4427	CD	LYS	A	562	-13.563	2.186	7.102	1.00	15.00	A	C
ATOM	4428	CE	LYS	A	562	-13.111	3.242	8.105	1.00	15.00	A	C
ATOM	4429	NZ	LYS	A	562	-12.034	4.118	7.562	1.00	15.00	A	N
ATOM	4430	C	LYS	A	562	-14.808	-0.209	10.574	1.00	15.00	A	C
ATOM	4431	O	LYS	A	562	-14.846	0.974	10.914	1.00	15.00	A	O
ATOM	4432	N	SER	A	563	-15.725	-1.099	10.943	1.00	15.00	A	N
ATOM	4433	CA	SER	A	563	-16.832	-0.729	11.814	1.00	15.00	A	C
ATOM	4434	CB	SER	A	563	-17.936	-1.790	11.757	1.00	15.00	A	C
ATOM	4435	OG	SER	A	563	-17.391	-3.100	11.771	1.00	15.00	A	O
ATOM	4436	C	SER	A	563	-16.308	-0.579	13.234	1.00	15.00	A	C
ATOM	4437	O	SER	A	563	-16.700	0.326	13.971	1.00	15.00	A	O
ATOM	4438	N	GLU	A	564	-15.404	-1.479	13.591	1.00	15.00	A	N
ATOM	4439	CA	GLU	A	564	-14.772	-1.478	14.894	1.00	15.00	A	C
ATOM	4440	CB	GLU	A	564	-14.558	-2.920	15.366	1.00	15.00	A	C
ATOM	4441	CG	GLU	A	564	-15.841	-3.707	15.575	1.00	15.00	A	C
ATOM	4442	CD	GLU	A	564	-16.563	-3.334	16.855	1.00	15.00	A	C
ATOM	4443	OE1	GLU	A	564	-16.120	-2.385	17.540	1.00	15.00	A	O
ATOM	4444	OE2	GLU	A	564	-17.571	-3.998	17.177	1.00	15.00	A	O
ATOM	4445	C	GLU	A	564	-13.424	-0.775	14.785	1.00	15.00	A	C
ATOM	4446	O	GLU	A	564	-12.843	-0.709	13.698	1.00	15.00	A	O
ATOM	4447	N	PRO	A	565	-12.906	-0.236	15.897	1.00	15.00	A	N
ATOM	4448	CA	PRO	A	565	-11.615	0.455	15.906	1.00	15.00	A	C
ATOM	4449	CB	PRO	A	565	-11.448	0.890	17.366	1.00	15.00	A	C
ATOM	4450	CG	PRO	A	565	-12.821	0.851	17.941	1.00	15.00	A	C
ATOM	4451	CD	PRO	A	565	-13.540	-0.250	17.224	1.00	15.00	A	C
ATOM	4452	C	PRO	A	565	-10.478	-0.484	15.513	1.00	15.00	A	C
ATOM	4453	O	PRO	A	565	-10.482	-1.661	15.881	1.00	15.00	A	O
ATOM	4454	N	TRP	A	566	-9.500	0.042	14.777	1.00	15.00	A	N
ATOM	4455	CA	TRP	A	566	-8.355	-0.755	14.341	1.00	15.00	A	C
ATOM	4456	CB	TRP	A	566	-7.398	0.064	13.460	1.00	15.00	A	C
ATOM	4457	CG	TRP	A	566	-6.612	1.116	14.187	1.00	15.00	A	C
ATOM	4458	CD1	TRP	A	566	-6.978	2.412	14.406	1.00	15.00	A	C
ATOM	4459	CD2	TRP	A	566	-5.316	0.960	14.782	1.00	15.00	A	C
ATOM	4460	NE1	TRP	A	566	-5.995	3.071	15.102	1.00	15.00	A	N
ATOM	4461	CE2	TRP	A	566	-4.964	2.201	15.345	1.00	15.00	A	C
ATOM	4462	CE3	TRP	A	566	-4.422	-0.110	14.894	1.00	15.00	A	C

```
ATOM   4463  CZ2 TRP A 566      -3.757   2.401  16.010  1.00 15.00      A    C
ATOM   4464  CZ3 TRP A 566      -3.226   0.091  15.555  1.00 15.00      A    C
ATOM   4465  CH2 TRP A 566      -2.903   1.337  16.104  1.00 15.00      A    C
ATOM   4466  C   TRP A 566      -7.625  -1.372  15.532  1.00 15.00      A    C
ATOM   4467  O   TRP A 566      -7.018  -2.436  15.420  1.00 15.00      A    O
ATOM   4468  N   THR A 567      -7.709  -0.696  16.673  1.00 15.00      A    N
ATOM   4469  CA  THR A 567      -7.085  -1.160  17.900  1.00 15.00      A    C
ATOM   4470  CB  THR A 567      -7.302  -0.122  19.016  1.00 15.00      A    C
ATOM   4471  OG1 THR A 567      -8.562   0.534  18.800  1.00 15.00      A    O
ATOM   4472  CG2 THR A 567      -6.196   0.923  18.994  1.00 15.00      A    C
ATOM   4473  C   THR A 567      -7.687  -2.502  18.321  1.00 15.00      A    C
ATOM   4474  O   THR A 567      -6.972  -3.424  18.716  1.00 15.00      A    O
ATOM   4475  N   LEU A 568      -9.009  -2.609  18.208  1.00 15.00      A    N
ATOM   4476  CA  LEU A 568      -9.713  -3.836  18.557  1.00 15.00      A    C
ATOM   4477  CB  LEU A 568     -11.209  -3.561  18.749  1.00 15.00      A    C
ATOM   4478  CG  LEU A 568     -12.103  -4.781  19.001  1.00 15.00      A    C
ATOM   4479  CD1 LEU A 568     -11.698  -5.503  20.278  1.00 15.00      A    C
ATOM   4480  CD2 LEU A 568     -13.565  -4.370  19.058  1.00 15.00      A    C
ATOM   4481  C   LEU A 568      -9.504  -4.881  17.469  1.00 15.00      A    C
ATOM   4482  O   LEU A 568      -9.356  -6.069  17.751  1.00 15.00      A    O
ATOM   4483  N   ALA A 569      -9.478  -4.421  16.222  1.00 15.00      A    N
ATOM   4484  CA  ALA A 569      -9.273  -5.306  15.082  1.00 15.00      A    C
ATOM   4485  CB  ALA A 569      -9.321  -4.517  13.782  1.00 15.00      A    C
ATOM   4486  C   ALA A 569      -7.943  -6.041  15.215  1.00 15.00      A    C
ATOM   4487  O   ALA A 569      -7.827  -7.215  14.860  1.00 15.00      A    O
ATOM   4488  N   LEU A 570      -6.947  -5.342  15.744  1.00 15.00      A    N
ATOM   4489  CA  LEU A 570      -5.629  -5.919  15.943  1.00 15.00      A    C
ATOM   4490  CB  LEU A 570      -4.601  -4.812  16.189  1.00 15.00      A    C
ATOM   4491  CG  LEU A 570      -3.137  -5.182  15.947  1.00 15.00      A    C
ATOM   4492  CD1 LEU A 570      -2.928  -5.631  14.510  1.00 15.00      A    C
ATOM   4493  CD2 LEU A 570      -2.232  -4.007  16.274  1.00 15.00      A    C
ATOM   4494  C   LEU A 570      -5.652  -6.894  17.117  1.00 15.00      A    C
ATOM   4495  O   LEU A 570      -5.088  -7.990  17.039  1.00 15.00      A    O
ATOM   4496  N   GLU A 571      -6.329  -6.496  18.192  1.00 15.00      A    N
ATOM   4497  CA  GLU A 571      -6.447  -7.320  19.392  1.00 15.00      A    C
ATOM   4498  CB  GLU A 571      -7.224  -6.567  20.475  1.00 15.00      A    C
ATOM   4499  CG  GLU A 571      -7.341  -7.305  21.799  1.00 15.00      A    C
ATOM   4500  CD  GLU A 571      -7.529  -6.363  22.970  1.00 15.00      A    C
ATOM   4501  OE1 GLU A 571      -6.549  -6.125  23.706  1.00 15.00      A    O
ATOM   4502  OE2 GLU A 571      -8.652  -5.842  23.150  1.00 15.00      A    O
ATOM   4503  C   GLU A 571      -7.127  -8.649  19.071  1.00 15.00      A    C
ATOM   4504  O   GLU A 571      -6.750  -9.699  19.595  1.00 15.00      A    O
ATOM   4505  N   ASN A 572      -8.107  -8.596  18.181  1.00 15.00      A    N
ATOM   4506  CA  ASN A 572      -8.843  -9.785  17.768  1.00 15.00      A    C
ATOM   4507  CB  ASN A 572      -9.918  -9.419  16.742  1.00 15.00      A    C
ATOM   4508  CG  ASN A 572     -11.313  -9.406  17.332  1.00 15.00      A    C
ATOM   4509  OD1 ASN A 572     -11.498  -9.156  18.519  1.00 15.00      A    O
ATOM   4510  ND2 ASN A 572     -12.308  -9.678  16.503  1.00 15.00      A    N
ATOM   4511  C   ASN A 572      -7.917 -10.847  17.186  1.00 15.00      A    C
ATOM   4512  O   ASN A 572      -8.194 -12.046  17.276  1.00 15.00      A    O
ATOM   4513  N   VAL A 573      -6.808 -10.415  16.602  1.00 15.00      A    N
ATOM   4514  CA  VAL A 573      -5.864 -11.343  16.001  1.00 15.00      A    C
ATOM   4515  CB  VAL A 573      -5.453 -10.918  14.574  1.00 15.00      A    C
ATOM   4516  CG1 VAL A 573      -4.508 -11.943  13.959  1.00 15.00      A    C
ATOM   4517  CG2 VAL A 573      -6.678 -10.731  13.694  1.00 15.00      A    C
ATOM   4518  C   VAL A 573      -4.609 -11.541  16.849  1.00 15.00      A    C
ATOM   4519  O   VAL A 573      -4.358 -12.639  17.343  1.00 15.00      A    O
ATOM   4520  N   VAL A 574      -3.850 -10.469  17.044  1.00 15.00      A    N
ATOM   4521  CA  VAL A 574      -2.585 -10.550  17.775  1.00 15.00      A    C
ATOM   4522  CB  VAL A 574      -1.553  -9.528  17.246  1.00 15.00      A    C
ATOM   4523  CG1 VAL A 574      -1.505  -9.568  15.728  1.00 15.00      A    C
ATOM   4524  CG2 VAL A 574      -1.869  -8.121  17.728  1.00 15.00      A    C
ATOM   4525  C   VAL A 574      -2.724 -10.428  19.292  1.00 15.00      A    C
ATOM   4526  O   VAL A 574      -1.795 -10.761  20.026  1.00 15.00      A    O
ATOM   4527  N   GLY A 575      -3.868  -9.957  19.764  1.00 15.00      A    N
ATOM   4528  CA  GLY A 575      -4.067  -9.810  21.197  1.00 15.00      A    C
ATOM   4529  C   GLY A 575      -3.435  -8.541  21.738  1.00 15.00      A    C
ATOM   4530  O   GLY A 575      -3.246  -8.394  22.943  1.00 15.00      A    O
ATOM   4531  N   ALA A 576      -3.098  -7.631  20.836  1.00 15.00      A    N
ATOM   4532  CA  ALA A 576      -2.494  -6.364  21.207  1.00 15.00      A    C
ATOM   4533  CB  ALA A 576      -1.018  -6.342  20.836  1.00 15.00      A    C
```

ATOM	4534	C	ALA	A	576	-3.237	-5.232	20.518	1.00	15.00	A	C
ATOM	4535	O	ALA	A	576	-3.499	-5.296	19.318	1.00	15.00	A	O
ATOM	4536	N	LYS	A	577	-3.578	-4.204	21.281	1.00	15.00	A	N
ATOM	4537	CA	LYS	A	577	-4.314	-3.062	20.751	1.00	15.00	A	C
ATOM	4538	CB	LYS	A	577	-5.034	-2.323	21.881	1.00	15.00	A	C
ATOM	4539	CG	LYS	A	577	-5.921	-3.218	22.725	1.00	15.00	A	C
ATOM	4540	CD	LYS	A	577	-6.637	-2.444	23.818	1.00	15.00	A	C
ATOM	4541	CE	LYS	A	577	-7.398	-3.387	24.736	1.00	15.00	A	C
ATOM	4542	NZ	LYS	A	577	-8.457	-2.688	25.506	1.00	15.00	A	N
ATOM	4543	C	LYS	A	577	-3.428	-2.093	19.976	1.00	15.00	A	C
ATOM	4544	O	LYS	A	577	-3.929	-1.184	19.320	1.00	15.00	A	O
ATOM	4545	N	ASN	A	578	-2.120	-2.279	20.042	1.00	15.00	A	N
ATOM	4546	CA	ASN	A	578	-1.210	-1.385	19.340	1.00	15.00	A	C
ATOM	4547	CB	ASN	A	578	-0.529	-0.411	20.310	1.00	15.00	A	C
ATOM	4548	CG	ASN	A	578	-0.138	0.900	19.647	1.00	15.00	A	C
ATOM	4549	OD1	ASN	A	578	-0.212	1.040	18.425	1.00	15.00	A	O
ATOM	4550	ND2	ASN	A	578	0.289	1.864	20.445	1.00	15.00	A	N
ATOM	4551	C	ASN	A	578	-0.169	-2.151	18.535	1.00	15.00	A	C
ATOM	4552	O	ASN	A	578	0.039	-3.351	18.736	1.00	15.00	A	O
ATOM	4553	N	MET	A	579	0.473	-1.435	17.625	1.00	15.00	A	N
ATOM	4554	CA	MET	A	579	1.503	-1.986	16.760	1.00	15.00	A	C
ATOM	4555	CB	MET	A	579	1.915	-0.905	15.758	1.00	15.00	A	C
ATOM	4556	CG	MET	A	579	2.671	-1.405	14.543	1.00	15.00	A	C
ATOM	4557	SD	MET	A	579	2.885	-0.117	13.297	1.00	15.00	A	S
ATOM	4558	CE	MET	A	579	3.694	1.163	14.258	1.00	15.00	A	C
ATOM	4559	C	MET	A	579	2.712	-2.423	17.588	1.00	15.00	A	C
ATOM	4560	O	MET	A	579	2.977	-1.864	18.653	1.00	15.00	A	O
ATOM	4561	N	ASN	A	580	3.439	-3.423	17.108	1.00	15.00	A	N
ATOM	4562	CA	ASN	A	580	4.608	-3.922	17.826	1.00	15.00	A	C
ATOM	4563	CB	ASN	A	580	4.201	-5.038	18.790	1.00	15.00	A	C
ATOM	4564	CG	ASN	A	580	5.229	-5.266	19.876	1.00	15.00	A	C
ATOM	4565	OD1	ASN	A	580	6.133	-6.082	19.726	1.00	15.00	A	O
ATOM	4566	ND2	ASN	A	580	5.097	-4.546	20.977	1.00	15.00	A	N
ATOM	4567	C	ASN	A	580	5.680	-4.417	16.858	1.00	15.00	A	C
ATOM	4568	O	ASN	A	580	5.366	-5.018	15.833	1.00	15.00	A	O
ATOM	4569	N	VAL	A	581	6.944	-4.175	17.195	1.00	15.00	A	N
ATOM	4570	CA	VAL	A	581	8.060	-4.576	16.337	1.00	15.00	A	C
ATOM	4571	CB	VAL	A	581	9.180	-3.518	16.312	1.00	15.00	A	C
ATOM	4572	CG1	VAL	A	581	8.873	-2.432	15.301	1.00	15.00	A	C
ATOM	4573	CG2	VAL	A	581	9.382	-2.922	17.692	1.00	15.00	A	C
ATOM	4574	C	VAL	A	581	8.672	-5.921	16.719	1.00	15.00	A	C
ATOM	4575	O	VAL	A	581	9.477	-6.465	15.962	1.00	15.00	A	O
ATOM	4576	N	ARG	A	582	8.301	-6.464	17.876	1.00	15.00	A	N
ATOM	4577	CA	ARG	A	582	8.852	-7.747	18.316	1.00	15.00	A	C
ATOM	4578	CB	ARG	A	582	8.342	-8.175	19.695	1.00	15.00	A	C
ATOM	4579	CG	ARG	A	582	9.077	-9.379	20.268	1.00	15.00	A	C
ATOM	4580	CD	ARG	A	582	8.296	-10.041	21.396	1.00	15.00	A	C
ATOM	4581	NE	ARG	A	582	6.870	-10.183	21.086	1.00	15.00	A	N
ATOM	4582	CZ	ARG	A	582	6.376	-10.947	20.103	1.00	15.00	A	C
ATOM	4583	NH1	ARG	A	582	7.184	-11.655	19.319	1.00	15.00	A	N
ATOM	4584	NH2	ARG	A	582	5.069	-11.000	19.902	1.00	15.00	A	N
ATOM	4585	C	ARG	A	582	8.684	-8.861	17.272	1.00	15.00	A	C
ATOM	4586	O	ARG	A	582	9.659	-9.538	16.950	1.00	15.00	A	O
ATOM	4587	N	PRO	A	583	7.462	-9.084	16.726	1.00	15.00	A	N
ATOM	4588	CA	PRO	A	583	7.239	-10.119	15.701	1.00	15.00	A	C
ATOM	4589	CB	PRO	A	583	5.764	-9.946	15.328	1.00	15.00	A	C
ATOM	4590	CG	PRO	A	583	5.151	-9.301	16.520	1.00	15.00	A	C
ATOM	4591	CD	PRO	A	583	6.206	-8.391	17.076	1.00	15.00	A	C
ATOM	4592	C	PRO	A	583	8.121	-9.889	14.472	1.00	15.00	A	C
ATOM	4593	O	PRO	A	583	8.582	-10.838	13.835	1.00	15.00	A	O
ATOM	4594	N	LEU	A	584	8.358	-8.618	14.154	1.00	15.00	A	N
ATOM	4595	CA	LEU	A	584	9.196	-8.246	13.019	1.00	15.00	A	C
ATOM	4596	CB	LEU	A	584	9.124	-6.730	12.785	1.00	15.00	A	C
ATOM	4597	CG	LEU	A	584	10.303	-6.079	12.052	1.00	15.00	A	C
ATOM	4598	CD1	LEU	A	584	10.286	-6.422	10.571	1.00	15.00	A	C
ATOM	4599	CD2	LEU	A	584	10.292	-4.572	12.254	1.00	15.00	A	C
ATOM	4600	C	LEU	A	584	10.636	-8.683	13.273	1.00	15.00	A	C
ATOM	4601	O	LEU	A	584	11.273	-9.297	12.417	1.00	15.00	A	O
ATOM	4602	N	LEU	A	585	11.134	-8.372	14.465	1.00	15.00	A	N
ATOM	4603	CA	LEU	A	585	12.491	-8.739	14.848	1.00	15.00	A	C
ATOM	4604	CB	LEU	A	585	12.867	-8.079	16.179	1.00	15.00	A	C

ATOM	4605	CG	LEU	A	585	12.811	-6.549	16.215	1.00	15.00	A	C
ATOM	4606	CD1	LEU	A	585	13.049	-6.036	17.627	1.00	15.00	A	C
ATOM	4607	CD2	LEU	A	585	13.822	-5.947	15.248	1.00	15.00	A	C
ATOM	4608	C	LEU	A	585	12.614	-10.254	14.954	1.00	15.00	A	C
ATOM	4609	O	LEU	A	585	13.625	-10.831	14.569	1.00	15.00	A	O
ATOM	4610	N	ASN	A	586	11.558	-10.883	15.464	1.00	15.00	A	N
ATOM	4611	CA	ASN	A	586	11.507	-12.336	15.621	1.00	15.00	A	C
ATOM	4612	CB	ASN	A	586	10.184	-12.731	16.300	1.00	15.00	A	C
ATOM	4613	CG	ASN	A	586	9.993	-14.229	16.472	1.00	15.00	A	C
ATOM	4614	OD1	ASN	A	586	10.952	-14.993	16.546	1.00	15.00	A	O
ATOM	4615	ND2	ASN	A	586	8.741	-14.657	16.565	1.00	15.00	A	N
ATOM	4616	C	ASN	A	586	11.647	-13.028	14.264	1.00	15.00	A	C
ATOM	4617	O	ASN	A	586	12.305	-14.060	14.139	1.00	15.00	A	O
ATOM	4618	N	TYR	A	587	11.040	-12.437	13.242	1.00	15.00	A	N
ATOM	4619	CA	TYR	A	587	11.098	-12.982	11.891	1.00	15.00	A	C
ATOM	4620	CB	TYR	A	587	10.139	-12.212	10.971	1.00	15.00	A	C
ATOM	4621	CG	TYR	A	587	10.096	-12.700	9.536	1.00	15.00	A	C
ATOM	4622	CD1	TYR	A	587	10.878	-12.103	8.553	1.00	15.00	A	C
ATOM	4623	CD2	TYR	A	587	9.265	-13.749	9.160	1.00	15.00	A	C
ATOM	4624	CE1	TYR	A	587	10.834	-12.534	7.241	1.00	15.00	A	C
ATOM	4625	CE2	TYR	A	587	9.217	-14.188	7.849	1.00	15.00	A	C
ATOM	4626	CZ	TYR	A	587	10.003	-13.576	6.894	1.00	15.00	A	C
ATOM	4627	OH	TYR	A	587	9.957	-14.008	5.586	1.00	15.00	A	O
ATOM	4628	C	TYR	A	587	12.522	-12.929	11.332	1.00	15.00	A	C
ATOM	4629	O	TYR	A	587	12.944	-13.823	10.600	1.00	15.00	A	O
ATOM	4630	N	PHE	A	588	13.265	-11.891	11.699	1.00	15.00	A	N
ATOM	4631	CA	PHE	A	588	14.629	-11.718	11.208	1.00	15.00	A	C
ATOM	4632	CB	PHE	A	588	14.852	-10.278	10.740	1.00	15.00	A	C
ATOM	4633	CG	PHE	A	588	14.101	-9.917	9.493	1.00	15.00	A	C
ATOM	4634	CD1	PHE	A	588	14.498	-10.413	8.263	1.00	15.00	A	C
ATOM	4635	CD2	PHE	A	588	13.000	-9.081	9.551	1.00	15.00	A	C
ATOM	4636	CE1	PHE	A	588	13.811	-10.082	7.112	1.00	15.00	A	C
ATOM	4637	CE2	PHE	A	588	12.307	-8.745	8.404	1.00	15.00	A	C
ATOM	4638	CZ	PHE	A	588	12.713	-9.247	7.183	1.00	15.00	A	C
ATOM	4639	C	PHE	A	588	15.682	-12.088	12.248	1.00	15.00	A	C
ATOM	4640	O	PHE	A	588	16.857	-11.764	12.077	1.00	15.00	A	O
ATOM	4641	N	GLU	A	589	15.269	-12.777	13.305	1.00	15.00	A	N
ATOM	4642	CA	GLU	A	589	16.192	-13.179	14.370	1.00	15.00	A	C
ATOM	4643	CB	GLU	A	589	15.475	-13.926	15.504	1.00	15.00	A	C
ATOM	4644	CG	GLU	A	589	16.379	-14.340	16.661	1.00	15.00	A	C
ATOM	4645	CD	GLU	A	589	17.046	-13.165	17.364	1.00	15.00	A	C
ATOM	4646	OE1	GLU	A	589	16.704	-12.900	18.534	1.00	15.00	A	O
ATOM	4647	OE2	GLU	A	589	17.930	-12.520	16.753	1.00	15.00	A	O
ATOM	4648	C	GLU	A	589	17.411	-13.961	13.846	1.00	15.00	A	C
ATOM	4649	O	GLU	A	589	18.552	-13.587	14.128	1.00	15.00	A	O
ATOM	4650	N	PRO	A	590	17.209	-15.046	13.059	1.00	15.00	A	N
ATOM	4651	CA	PRO	A	590	18.328	-15.835	12.518	1.00	15.00	A	C
ATOM	4652	CB	PRO	A	590	17.641	-16.923	11.686	1.00	15.00	A	C
ATOM	4653	CG	PRO	A	590	16.262	-17.006	12.240	1.00	15.00	A	C
ATOM	4654	CD	PRO	A	590	15.906	-15.610	12.658	1.00	15.00	A	C
ATOM	4655	C	PRO	A	590	19.259	-15.001	11.637	1.00	15.00	A	C
ATOM	4656	O	PRO	A	590	20.428	-15.341	11.457	1.00	15.00	A	O
ATOM	4657	N	LEU	A	591	18.741	-13.910	11.090	1.00	15.00	A	N
ATOM	4658	CA	LEU	A	591	19.542	-13.043	10.240	1.00	15.00	A	C
ATOM	4659	CB	LEU	A	591	18.672	-12.341	9.196	1.00	15.00	A	C
ATOM	4660	CG	LEU	A	591	19.427	-11.601	8.089	1.00	15.00	A	C
ATOM	4661	CD1	LEU	A	591	20.145	-12.585	7.176	1.00	15.00	A	C
ATOM	4662	CD2	LEU	A	591	18.484	-10.713	7.295	1.00	15.00	A	C
ATOM	4663	C	LEU	A	591	20.287	-12.017	11.084	1.00	15.00	A	C
ATOM	4664	O	LEU	A	591	21.435	-11.679	10.798	1.00	15.00	A	O
ATOM	4665	N	PHE	A	592	19.630	-11.541	12.136	1.00	15.00	A	N
ATOM	4666	CA	PHE	A	592	20.219	-10.555	13.032	1.00	15.00	A	C
ATOM	4667	CB	PHE	A	592	19.192	-10.090	14.069	1.00	15.00	A	C
ATOM	4668	CG	PHE	A	592	19.631	-8.905	14.883	1.00	15.00	A	C
ATOM	4669	CD1	PHE	A	592	20.366	-7.880	14.306	1.00	15.00	A	C
ATOM	4670	CD2	PHE	A	592	19.307	-8.815	16.226	1.00	15.00	A	C
ATOM	4671	CE1	PHE	A	592	20.769	-6.791	15.053	1.00	15.00	A	C
ATOM	4672	CE2	PHE	A	592	19.706	-7.728	16.980	1.00	15.00	A	C
ATOM	4673	CZ	PHE	A	592	20.439	-6.715	16.393	1.00	15.00	A	C
ATOM	4674	C	PHE	A	592	21.461	-11.118	13.716	1.00	15.00	A	C
ATOM	4675	O	PHE	A	592	22.437	-10.403	13.946	1.00	15.00	A	O

ATOM	4676	N	THR	A	593	21.420	-12.400	14.048	1.00	15.00	A	N
ATOM	4677	CA	THR	A	593	22.551	-13.052	14.683	1.00	15.00	A	C
ATOM	4678	CB	THR	A	593	22.146	-14.416	15.272	1.00	15.00	A	C
ATOM	4679	OG1	THR	A	593	21.087	-14.975	14.486	1.00	15.00	A	O
ATOM	4680	CG2	THR	A	593	21.665	-14.248	16.706	1.00	15.00	A	C
ATOM	4681	C	THR	A	593	23.697	-13.225	13.684	1.00	15.00	A	C
ATOM	4682	O	THR	A	593	24.864	-13.033	14.026	1.00	15.00	A	O
ATOM	4683	N	TRP	A	594	23.345	-13.556	12.443	1.00	15.00	A	N
ATOM	4684	CA	TRP	A	594	24.330	-13.748	11.381	1.00	15.00	A	C
ATOM	4685	CB	TRP	A	594	23.664	-14.357	10.142	1.00	15.00	A	C
ATOM	4686	CG	TRP	A	594	24.628	-14.735	9.055	1.00	15.00	A	C
ATOM	4687	CD1	TRP	A	594	25.390	-15.865	8.994	1.00	15.00	A	C
ATOM	4688	CD2	TRP	A	594	24.929	-13.984	7.872	1.00	15.00	A	C
ATOM	4689	NE1	TRP	A	594	26.150	-15.863	7.849	1.00	15.00	A	N
ATOM	4690	CE2	TRP	A	594	25.884	-14.720	7.143	1.00	15.00	A	C
ATOM	4691	CE3	TRP	A	594	24.485	-12.762	7.360	1.00	15.00	A	C
ATOM	4692	CZ2	TRP	A	594	26.401	-14.274	5.930	1.00	15.00	A	C
ATOM	4693	CZ3	TRP	A	594	24.999	-12.321	6.157	1.00	15.00	A	C
ATOM	4694	CH2	TRP	A	594	25.947	-13.075	5.456	1.00	15.00	A	C
ATOM	4695	C	TRP	A	594	25.015	-12.429	11.021	1.00	15.00	A	C
ATOM	4696	O	TRP	A	594	26.237	-12.371	10.887	1.00	15.00	A	O
ATOM	4697	N	LEU	A	595	24.220	-11.372	10.865	1.00	15.00	A	N
ATOM	4698	CA	LEU	A	595	24.756	-10.053	10.530	1.00	15.00	A	C
ATOM	4699	CB	LEU	A	595	23.626	-9.034	10.345	1.00	15.00	A	C
ATOM	4700	CG	LEU	A	595	22.726	-9.233	9.121	1.00	15.00	A	C
ATOM	4701	CD1	LEU	A	595	21.565	-8.251	9.141	1.00	15.00	A	C
ATOM	4702	CD2	LEU	A	595	23.523	-9.093	7.833	1.00	15.00	A	C
ATOM	4703	C	LEU	A	595	25.728	-9.579	11.607	1.00	15.00	A	C
ATOM	4704	O	LEU	A	595	26.740	-8.941	11.312	1.00	15.00	A	O
ATOM	4705	N	LYS	A	596	25.418	-9.921	12.856	1.00	15.00	A	N
ATOM	4706	CA	LYS	A	596	26.253	-9.549	13.992	1.00	15.00	A	C
ATOM	4707	CB	LYS	A	596	25.573	-9.947	15.308	1.00	15.00	A	C
ATOM	4708	CG	LYS	A	596	25.072	-8.776	16.147	1.00	15.00	A	C
ATOM	4709	CD	LYS	A	596	23.599	-8.928	16.520	1.00	15.00	A	C
ATOM	4710	CE	LYS	A	596	23.353	-10.175	17.361	1.00	15.00	A	C
ATOM	4711	NZ	LYS	A	596	21.911	-10.362	17.700	1.00	15.00	A	N
ATOM	4712	C	LYS	A	596	27.635	-10.196	13.902	1.00	15.00	A	C
ATOM	4713	O	LYS	A	596	28.631	-9.600	14.304	1.00	15.00	A	O
ATOM	4714	N	ASP	A	597	27.691	-11.417	13.377	1.00	15.00	A	N
ATOM	4715	CA	ASP	A	597	28.963	-12.127	13.238	1.00	15.00	A	C
ATOM	4716	CB	ASP	A	597	28.759	-13.641	13.105	1.00	15.00	A	C
ATOM	4717	CG	ASP	A	597	30.018	-14.348	12.617	1.00	15.00	A	C
ATOM	4718	OD1	ASP	A	597	30.035	-14.794	11.454	1.00	15.00	A	O
ATOM	4719	OD2	ASP	A	597	31.006	-14.430	13.389	1.00	15.00	A	O
ATOM	4720	C	ASP	A	597	29.758	-11.608	12.044	1.00	15.00	A	C
ATOM	4721	O	ASP	A	597	30.982	-11.476	12.104	1.00	15.00	A	O
ATOM	4722	N	GLN	A	598	29.062	-11.292	10.964	1.00	15.00	A	N
ATOM	4723	CA	GLN	A	598	29.719	-10.806	9.758	1.00	15.00	A	C
ATOM	4724	CB	GLN	A	598	28.758	-10.816	8.568	1.00	15.00	A	C
ATOM	4725	CG	GLN	A	598	28.318	-12.208	8.151	1.00	15.00	A	C
ATOM	4726	CD	GLN	A	598	29.449	-13.041	7.580	1.00	15.00	A	C
ATOM	4727	OE1	GLN	A	598	30.303	-12.541	6.850	1.00	15.00	A	O
ATOM	4728	NE2	GLN	A	598	29.470	-14.316	7.928	1.00	15.00	A	N
ATOM	4729	C	GLN	A	598	30.297	-9.415	9.953	1.00	15.00	A	C
ATOM	4730	O	GLN	A	598	31.364	-9.098	9.430	1.00	15.00	A	O
ATOM	4731	N	ASN	A	599	29.617	-8.609	10.751	1.00	15.00	A	N
ATOM	4732	CA	ASN	A	599	30.048	-7.239	10.987	1.00	15.00	A	C
ATOM	4733	CB	ASN	A	599	28.855	-6.336	11.309	1.00	15.00	A	C
ATOM	4734	CG	ASN	A	599	28.043	-5.964	10.083	1.00	15.00	A	C
ATOM	4735	OD1	ASN	A	599	28.586	-5.689	9.014	1.00	15.00	A	O
ATOM	4736	ND2	ASN	A	599	26.730	-5.959	10.232	1.00	15.00	A	N
ATOM	4737	C	ASN	A	599	31.104	-7.131	12.077	1.00	15.00	A	C
ATOM	4738	O	ASN	A	599	31.500	-6.028	12.443	1.00	15.00	A	O
ATOM	4739	N	LYS	A	600	31.571	-8.264	12.594	1.00	15.00	A	N
ATOM	4740	CA	LYS	A	600	32.589	-8.238	13.641	1.00	15.00	A	C
ATOM	4741	CB	LYS	A	600	32.846	-9.631	14.222	1.00	15.00	A	C
ATOM	4742	CG	LYS	A	600	33.604	-10.576	13.304	1.00	15.00	A	C
ATOM	4743	CD	LYS	A	600	33.953	-11.870	14.019	1.00	15.00	A	C
ATOM	4744	CE	LYS	A	600	34.225	-12.990	13.032	1.00	15.00	A	C
ATOM	4745	NZ	LYS	A	600	33.001	-13.365	12.280	1.00	15.00	A	N
ATOM	4746	C	LYS	A	600	33.883	-7.606	13.133	1.00	15.00	A	C

ATOM	4747	O	LYS	A	600	34.631	-7.002	13.896	1.00	15.00	A	O
ATOM	4748	N	ASN	A	601	34.125	-7.734	11.833	1.00	15.00	A	N
ATOM	4749	CA	ASN	A	601	35.324	-7.171	11.215	1.00	15.00	A	C
ATOM	4750	CB	ASN	A	601	36.033	-8.206	10.328	1.00	15.00	A	C
ATOM	4751	CG	ASN	A	601	36.676	-9.344	11.104	1.00	15.00	A	C
ATOM	4752	OD1	ASN	A	601	36.743	-9.326	12.330	1.00	15.00	A	O
ATOM	4753	ND2	ASN	A	601	37.168	-10.344	10.387	1.00	15.00	A	N
ATOM	4754	C	ASN	A	601	34.970	-5.941	10.385	1.00	15.00	A	C
ATOM	4755	O	ASN	A	601	35.651	-5.614	9.413	1.00	15.00	A	O
ATOM	4756	N	SER	A	602	33.897	-5.266	10.770	1.00	15.00	A	N
ATOM	4757	CA	SER	A	602	33.444	-4.077	10.068	1.00	15.00	A	C
ATOM	4758	CB	SER	A	602	32.270	-4.424	9.150	1.00	15.00	A	C
ATOM	4759	OG	SER	A	602	32.700	-5.236	8.071	1.00	15.00	A	O
ATOM	4760	C	SER	A	602	33.041	-2.983	11.053	1.00	15.00	A	C
ATOM	4761	O	SER	A	602	32.915	-3.231	12.251	1.00	15.00	A	O
ATOM	4762	N	PHE	A	603	32.851	-1.773	10.544	1.00	15.00	A	N
ATOM	4763	CA	PHE	A	603	32.459	-0.652	11.380	1.00	15.00	A	C
ATOM	4764	CB	PHE	A	603	33.163	0.631	10.927	1.00	15.00	A	C
ATOM	4765	CG	PHE	A	603	32.716	1.858	11.670	1.00	15.00	A	C
ATOM	4766	CD1	PHE	A	603	33.151	2.097	12.963	1.00	15.00	A	C
ATOM	4767	CD2	PHE	A	603	31.851	2.765	11.079	1.00	15.00	A	C
ATOM	4768	CE1	PHE	A	603	32.732	3.218	13.653	1.00	15.00	A	C
ATOM	4769	CE2	PHE	A	603	31.429	3.888	11.762	1.00	15.00	A	C
ATOM	4770	CZ	PHE	A	603	31.870	4.115	13.051	1.00	15.00	A	C
ATOM	4771	C	PHE	A	603	30.947	-0.464	11.359	1.00	15.00	A	C
ATOM	4772	O	PHE	A	603	30.342	-0.311	10.295	1.00	15.00	A	O
ATOM	4773	N	VAL	A	604	30.344	-0.491	12.536	1.00	15.00	A	N
ATOM	4774	CA	VAL	A	604	28.912	-0.305	12.668	1.00	15.00	A	C
ATOM	4775	CB	VAL	A	604	28.358	-1.068	13.888	1.00	15.00	A	C
ATOM	4776	CG1	VAL	A	604	26.838	-1.014	13.922	1.00	15.00	A	C
ATOM	4777	CG2	VAL	A	604	28.841	-2.511	13.868	1.00	15.00	A	C
ATOM	4778	C	VAL	A	604	28.615	1.182	12.804	1.00	15.00	A	C
ATOM	4779	O	VAL	A	604	29.302	1.893	13.537	1.00	15.00	A	O
ATOM	4780	N	GLY	A	605	27.605	1.642	12.088	1.00	15.00	A	N
ATOM	4781	CA	GLY	A	605	27.252	3.045	12.119	1.00	15.00	A	C
ATOM	4782	C	GLY	A	605	27.665	3.735	10.833	1.00	15.00	A	C
ATOM	4783	O	GLY	A	605	28.138	3.079	9.900	1.00	15.00	A	O
ATOM	4784	N	TRP	A	606	27.487	5.046	10.776	1.00	15.00	A	N
ATOM	4785	CA	TRP	A	606	27.843	5.819	9.591	1.00	15.00	A	C
ATOM	4786	CB	TRP	A	606	26.742	5.725	8.519	1.00	15.00	A	C
ATOM	4787	CG	TRP	A	606	25.355	5.962	9.041	1.00	15.00	A	C
ATOM	4788	CD1	TRP	A	606	24.764	7.168	9.284	1.00	15.00	A	C
ATOM	4789	CD2	TRP	A	606	24.385	4.964	9.384	1.00	15.00	A	C
ATOM	4790	NE1	TRP	A	606	23.490	6.982	9.765	1.00	15.00	A	N
ATOM	4791	CE2	TRP	A	606	23.234	5.639	9.833	1.00	15.00	A	C
ATOM	4792	CE3	TRP	A	606	24.378	3.566	9.357	1.00	15.00	A	C
ATOM	4793	CZ2	TRP	A	606	22.089	4.965	10.252	1.00	15.00	A	C
ATOM	4794	CZ3	TRP	A	606	23.242	2.899	9.772	1.00	15.00	A	C
ATOM	4795	CH2	TRP	A	606	22.113	3.598	10.213	1.00	15.00	A	C
ATOM	4796	C	TRP	A	606	28.103	7.277	9.950	1.00	15.00	A	C
ATOM	4797	O	TRP	A	606	27.634	7.763	10.980	1.00	15.00	A	O
ATOM	4798	N	SER	A	607	28.866	7.962	9.113	1.00	15.00	A	N
ATOM	4799	CA	SER	A	607	29.162	9.367	9.328	1.00	15.00	A	C
ATOM	4800	CB	SER	A	607	30.655	9.642	9.131	1.00	15.00	A	C
ATOM	4801	OG	SER	A	607	31.174	8.929	8.020	1.00	15.00	A	O
ATOM	4802	C	SER	A	607	28.306	10.216	8.392	1.00	15.00	A	C
ATOM	4803	O	SER	A	607	28.108	9.860	7.228	1.00	15.00	A	O
ATOM	4804	N	THR	A	608	27.791	11.324	8.902	1.00	15.00	A	N
ATOM	4805	CA	THR	A	608	26.926	12.201	8.126	1.00	15.00	A	C
ATOM	4806	CB	THR	A	608	25.876	12.847	9.048	1.00	15.00	A	C
ATOM	4807	OG1	THR	A	608	26.500	13.209	10.288	1.00	15.00	A	O
ATOM	4808	CG2	THR	A	608	24.760	11.859	9.338	1.00	15.00	A	C
ATOM	4809	C	THR	A	608	27.695	13.298	7.387	1.00	15.00	A	C
ATOM	4810	O	THR	A	608	27.100	14.104	6.666	1.00	15.00	A	O
ATOM	4811	N	ASP	A	609	29.009	13.315	7.548	1.00	15.00	A	N
ATOM	4812	CA	ASP	A	609	29.849	14.326	6.914	1.00	15.00	A	C
ATOM	4813	CB	ASP	A	609	31.044	14.681	7.812	1.00	15.00	A	C
ATOM	4814	CG	ASP	A	609	32.050	13.551	7.955	1.00	15.00	A	C
ATOM	4815	OD1	ASP	A	609	31.617	12.382	8.099	1.00	15.00	A	O
ATOM	4816	OD2	ASP	A	609	33.263	13.827	7.916	1.00	15.00	A	O
ATOM	4817	C	ASP	A	609	30.324	13.888	5.529	1.00	15.00	A	C

ATOM	4818	O	ASP	A	609	30.224	14.651	4.561	1.00	15.00	A	O
ATOM	4819	N	TRP	A	610	30.823	12.660	5.438	1.00	15.00	A	N
ATOM	4820	CA	TRP	A	610	31.322	12.120	4.182	1.00	15.00	A	C
ATOM	4821	CB	TRP	A	610	31.902	10.711	4.366	1.00	15.00	A	C
ATOM	4822	CG	TRP	A	610	32.508	10.157	3.107	1.00	15.00	A	C
ATOM	4823	CD1	TRP	A	610	33.760	10.406	2.625	1.00	15.00	A	C
ATOM	4824	CD2	TRP	A	610	31.883	9.275	2.163	1.00	15.00	A	C
ATOM	4825	NE1	TRP	A	610	33.955	9.735	1.442	1.00	15.00	A	N
ATOM	4826	CE2	TRP	A	610	32.818	9.033	1.137	1.00	15.00	A	C
ATOM	4827	CE3	TRP	A	610	30.626	8.665	2.087	1.00	15.00	A	C
ATOM	4828	CZ2	TRP	A	610	32.536	8.210	0.049	1.00	15.00	A	C
ATOM	4829	CZ3	TRP	A	610	30.349	7.849	1.007	1.00	15.00	A	C
ATOM	4830	CH2	TRP	A	610	31.299	7.628	0.003	1.00	15.00	A	C
ATOM	4831	C	TRP	A	610	30.258	12.111	3.092	1.00	15.00	A	C
ATOM	4832	O	TRP	A	610	29.095	11.785	3.333	1.00	15.00	A	O
ATOM	4833	N	SER	A	611	30.681	12.470	1.892	1.00	15.00	A	N
ATOM	4834	CA	SER	A	611	29.813	12.505	0.734	1.00	15.00	A	C
ATOM	4835	CB	SER	A	611	29.171	13.888	0.618	1.00	15.00	A	C
ATOM	4836	OG	SER	A	611	30.112	14.908	0.908	1.00	15.00	A	O
ATOM	4837	C	SER	A	611	30.649	12.204	-0.504	1.00	15.00	A	C
ATOM	4838	O	SER	A	611	31.855	12.458	-0.504	1.00	15.00	A	O
ATOM	4839	N	PRO	A	612	30.043	11.652	-1.567	1.00	15.00	A	N
ATOM	4840	CA	PRO	A	612	30.756	11.326	-2.811	1.00	15.00	A	C
ATOM	4841	CB	PRO	A	612	29.752	10.459	-3.591	1.00	15.00	A	C
ATOM	4842	CG	PRO	A	612	28.667	10.131	-2.621	1.00	15.00	A	C
ATOM	4843	CD	PRO	A	612	28.631	11.269	-1.646	1.00	15.00	A	C
ATOM	4844	C	PRO	A	612	31.124	12.569	-3.630	1.00	15.00	A	C
ATOM	4845	O	PRO	A	612	31.197	12.509	-4.855	1.00	15.00	A	O
ATOM	4846	N	TYR	A	613	31.355	13.690	-2.952	1.00	15.00	A	N
ATOM	4847	CA	TYR	A	613	31.721	14.930	-3.627	1.00	15.00	A	C
ATOM	4848	CB	TYR	A	613	30.481	15.677	-4.145	1.00	15.00	A	C
ATOM	4849	CG	TYR	A	613	29.521	16.170	-3.077	1.00	15.00	A	C
ATOM	4850	CD1	TYR	A	613	29.721	17.392	-2.443	1.00	15.00	A	C
ATOM	4851	CD2	TYR	A	613	28.405	15.425	-2.720	1.00	15.00	A	C
ATOM	4852	CE1	TYR	A	613	28.840	17.855	-1.485	1.00	15.00	A	C
ATOM	4853	CE2	TYR	A	613	27.519	15.881	-1.761	1.00	15.00	A	C
ATOM	4854	CZ	TYR	A	613	27.741	17.095	-1.147	1.00	15.00	A	C
ATOM	4855	OH	TYR	A	613	26.859	17.558	-0.198	1.00	15.00	A	O
ATOM	4856	C	TYR	A	613	32.571	15.834	-2.734	1.00	15.00	A	C
ATOM	4857	O	TYR	A	613	32.804	16.998	-3.056	1.00	15.00	A	O
ATOM	4858	N	ALA	A	614	33.043	15.295	-1.619	1.00	15.00	A	N
ATOM	4859	CA	ALA	A	614	33.858	16.068	-0.692	1.00	15.00	A	C
ATOM	4860	CB	ALA	A	614	33.179	16.175	0.666	1.00	15.00	A	C
ATOM	4861	C	ALA	A	614	35.251	15.466	-0.558	1.00	15.00	A	C
ATOM	4862	O	ALA	A	614	35.581	14.850	0.457	1.00	15.00	A	O
ATOM	4863	N	ASP	A	615	36.053	15.629	-1.599	1.00	15.00	A	N
ATOM	4864	CA	ASP	A	615	37.417	15.109	-1.609	1.00	15.00	A	C
ATOM	4865	CB	ASP	A	615	38.011	15.169	-3.017	1.00	15.00	A	C
ATOM	4866	CG	ASP	A	615	37.763	13.904	-3.809	1.00	15.00	A	C
ATOM	4867	OD1	ASP	A	615	36.858	13.910	-4.669	1.00	15.00	A	O
ATOM	4868	OD2	ASP	A	615	38.474	12.907	-3.568	1.00	15.00	A	O
ATOM	4869	C	ASP	A	615	38.306	15.879	-0.642	1.00	15.00	A	C
ATOM	4870	O	ASP	A	615	38.131	17.085	-0.449	1.00	15.00	A	O
TER	4871		ASP	A	615							
ATOM	4871	N	PHE	B	1	-44.607	1.251	-3.988	1.00	15.00	B	N
ATOM	4872	CA	PHE	B	1	-44.881	0.552	-5.235	1.00	15.00	B	C
ATOM	4873	CB	PHE	B	1	-43.915	1.027	-6.330	1.00	15.00	B	C
ATOM	4874	CG	PHE	B	1	-44.183	0.436	-7.687	1.00	15.00	B	C
ATOM	4875	CD1	PHE	B	1	-45.253	0.876	-8.451	1.00	15.00	B	C
ATOM	4876	CD2	PHE	B	1	-43.367	-0.561	-8.199	1.00	15.00	B	C
ATOM	4877	CE1	PHE	B	1	-45.505	0.334	-9.697	1.00	15.00	B	C
ATOM	4878	CE2	PHE	B	1	-43.613	-1.107	-9.444	1.00	15.00	B	C
ATOM	4879	CZ	PHE	B	1	-44.684	-0.660	-10.195	1.00	15.00	B	C
ATOM	4880	C	PHE	B	1	-44.754	-0.956	-5.034	1.00	15.00	B	C
ATOM	4881	O	PHE	B	1	-43.811	-1.419	-4.390	1.00	15.00	B	O
ATOM	4882	N	PRO	B	2	-45.719	-1.735	-5.552	1.00	15.00	B	N
ATOM	4883	CA	PRO	B	2	-45.702	-3.196	-5.440	1.00	15.00	B	C
ATOM	4884	CB	PRO	B	2	-47.009	-3.631	-6.115	1.00	15.00	B	C
ATOM	4885	CG	PRO	B	2	-47.393	-2.483	-6.984	1.00	15.00	B	C
ATOM	4886	CD	PRO	B	2	-46.909	-1.253	-6.274	1.00	15.00	B	C
ATOM	4887	C	PRO	B	2	-44.504	-3.806	-6.162	1.00	15.00	B	C

ATOM	4888	O	PRO	B	2	-44.441	-3.805	-7.392	1.00	15.00	B	O
ATOM	4889	N	ILE	B	3	-43.549	-4.302	-5.384	1.00	15.00	B	N
ATOM	4890	CA	ILE	B	3	-42.350	-4.920	-5.933	1.00	15.00	B	C
ATOM	4891	CB	ILE	B	3	-41.336	-5.260	-4.817	1.00	15.00	B	C
ATOM	4892	CG1	ILE	B	3	-41.026	-4.013	-3.979	1.00	15.00	B	C
ATOM	4893	CG2	ILE	B	3	-40.056	-5.839	-5.406	1.00	15.00	B	C
ATOM	4894	CD1	ILE	B	3	-40.328	-4.312	-2.670	1.00	15.00	B	C
ATOM	4895	C	ILE	B	3	-42.707	-6.197	-6.691	1.00	15.00	B	C
ATOM	4896	O	ILE	B	3	-43.246	-7.140	-6.111	1.00	15.00	B	O
ATOM	4897	N	PRO	B	4	-42.431	-6.230	-8.005	1.00	15.00	B	N
ATOM	4898	CA	PRO	B	4	-42.716	-7.397	-8.839	1.00	15.00	B	C
ATOM	4899	CB	PRO	B	4	-42.506	-6.874	-10.262	1.00	15.00	B	C
ATOM	4900	CG	PRO	B	4	-41.535	-5.755	-10.110	1.00	15.00	B	C
ATOM	4901	CD	PRO	B	4	-41.827	-5.129	-8.776	1.00	15.00	B	C
ATOM	4902	C	PRO	B	4	-41.746	-8.538	-8.547	1.00	15.00	B	C
ATOM	4903	O	PRO	B	4	-40.845	-8.406	-7.717	1.00	15.00	B	O
ATOM	4904	N	LEU	B	5	-41.919	-9.655	-9.239	1.00	15.00	B	N
ATOM	4905	CA	LEU	B	5	-41.057	-10.806	-9.033	1.00	15.00	B	C
ATOM	4906	CB	LEU	B	5	-41.882	-12.026	-8.613	1.00	15.00	B	C
ATOM	4907	CG	LEU	B	5	-42.686	-11.888	-7.318	1.00	15.00	B	C
ATOM	4908	CD1	LEU	B	5	-43.506	-13.144	-7.065	1.00	15.00	B	C
ATOM	4909	CD2	LEU	B	5	-41.768	-11.601	-6.138	1.00	15.00	B	C
ATOM	4910	C	LEU	B	5	-40.221	-11.128	-10.272	1.00	15.00	B	C
ATOM	4911	O	LEU	B	5	-40.528	-12.062	-11.014	1.00	15.00	B	O
ATOM	4912	N	PRO	B	6	-39.141	-10.368	-10.511	1.00	15.00	B	N
ATOM	4913	CA	PRO	B	6	-38.258	-10.581	-11.646	1.00	15.00	B	C
ATOM	4914	CB	PRO	B	6	-37.833	-9.148	-11.973	1.00	15.00	B	C
ATOM	4915	CG	PRO	B	6	-37.825	-8.434	-10.653	1.00	15.00	B	C
ATOM	4916	CD	PRO	B	6	-38.679	-9.235	-9.696	1.00	15.00	B	C
ATOM	4917	C	PRO	B	6	-37.044	-11.411	-11.229	1.00	15.00	B	C
ATOM	4918	O	PRO	B	6	-37.151	-12.288	-10.374	1.00	15.00	B	O
ATOM	4919	N	TYR	B	7	-35.895	-11.132	-11.829	1.00	15.00	B	N
ATOM	4920	CA	TYR	B	7	-34.673	-11.851	-11.497	1.00	15.00	B	C
ATOM	4921	CB	TYR	B	7	-33.786	-12.011	-12.733	1.00	15.00	B	C
ATOM	4922	CG	TYR	B	7	-34.351	-12.971	-13.757	1.00	15.00	B	C
ATOM	4923	CD1	TYR	B	7	-34.474	-14.324	-13.469	1.00	15.00	B	C
ATOM	4924	CD2	TYR	B	7	-34.765	-12.526	-15.007	1.00	15.00	B	C
ATOM	4925	CE1	TYR	B	7	-34.993	-15.208	-14.395	1.00	15.00	B	C
ATOM	4926	CE2	TYR	B	7	-35.285	-13.403	-15.940	1.00	15.00	B	C
ATOM	4927	CZ	TYR	B	7	-35.397	-14.742	-15.628	1.00	15.00	B	C
ATOM	4928	OH	TYR	B	7	-35.915	-15.619	-16.552	1.00	15.00	B	O
ATOM	4929	C	TYR	B	7	-33.927	-11.132	-10.376	1.00	15.00	B	C
ATOM	4930	O	TYR	B	7	-34.244	-9.990	-10.057	1.00	15.00	B	O
ATOM	4931	N	CYS	B	8	-32.934	-11.794	-9.789	1.00	15.00	B	N
ATOM	4932	CA	CYS	B	8	-32.167	-11.208	-8.689	1.00	15.00	B	C
ATOM	4933	C	CYS	B	8	-31.527	-9.867	-9.050	1.00	15.00	B	C
ATOM	4934	O	CYS	B	8	-31.673	-8.890	-8.316	1.00	15.00	B	O
ATOM	4935	CB	CYS	B	8	-31.118	-12.181	-8.146	1.00	15.00	B	C
ATOM	4936	SG	CYS	B	8	-30.144	-11.511	-6.759	1.00	15.00	B	S
ATOM	4937	N	TRP	B	9	-30.825	-9.812	-10.178	1.00	15.00	B	N
ATOM	4938	CA	TRP	B	9	-30.172	-8.575	-10.600	1.00	15.00	B	C
ATOM	4939	CB	TRP	B	9	-29.227	-8.798	-11.786	1.00	15.00	B	C
ATOM	4940	CG	TRP	B	9	-29.792	-9.648	-12.883	1.00	15.00	B	C
ATOM	4941	CD1	TRP	B	9	-30.433	-9.217	-14.009	1.00	15.00	B	C
ATOM	4942	CD2	TRP	B	9	-29.756	-11.078	-12.967	1.00	15.00	B	C
ATOM	4943	NE1	TRP	B	9	-30.804	-10.288	-14.782	1.00	15.00	B	N
ATOM	4944	CE2	TRP	B	9	-30.397	-11.443	-14.167	1.00	15.00	B	C
ATOM	4945	CE3	TRP	B	9	-29.243	-12.085	-12.143	1.00	15.00	B	C
ATOM	4946	CZ2	TRP	B	9	-30.540	-12.771	-14.562	1.00	15.00	B	C
ATOM	4947	CZ3	TRP	B	9	-29.386	-13.402	-12.537	1.00	15.00	B	C
ATOM	4948	CH2	TRP	B	9	-30.028	-13.733	-13.736	1.00	15.00	B	C
ATOM	4949	C	TRP	B	9	-31.190	-7.478	-10.892	1.00	15.00	B	C
ATOM	4950	O	TRP	B	9	-30.950	-6.304	-10.608	1.00	15.00	B	O
ATOM	4951	N	LEU	B	10	-32.335	-7.874	-11.434	1.00	15.00	B	N
ATOM	4952	CA	LEU	B	10	-33.397	-6.931	-11.752	1.00	15.00	B	C
ATOM	4953	CB	LEU	B	10	-34.438	-7.577	-12.668	1.00	15.00	B	C
ATOM	4954	CG	LEU	B	10	-33.940	-8.017	-14.047	1.00	15.00	B	C
ATOM	4955	CD1	LEU	B	10	-35.015	-8.804	-14.777	1.00	15.00	B	C
ATOM	4956	CD2	LEU	B	10	-33.504	-6.814	-14.872	1.00	15.00	B	C
ATOM	4957	C	LEU	B	10	-34.054	-6.426	-10.474	1.00	15.00	B	C
ATOM	4958	O	LEU	B	10	-34.425	-5.262	-10.379	1.00	15.00	B	O

ATOM	4959	N	CYS	B	11	-34.187	-7.314	-9.494	1.00	15.00	B	N
ATOM	4960	CA	CYS	B	11	-34.780	-6.964	-8.207	1.00	15.00	B	C
ATOM	4961	C	CYS	B	11	-33.947	-5.885	-7.526	1.00	15.00	B	C
ATOM	4962	O	CYS	B	11	-34.482	-4.897	-7.020	1.00	15.00	B	O
ATOM	4963	CB	CYS	B	11	-34.869	-8.209	-7.313	1.00	15.00	B	C
ATOM	4964	SG	CYS	B	11	-35.310	-7.884	-5.573	1.00	15.00	B	S
ATOM	4965	N	ARG	B	12	-32.628	-6.068	-7.557	1.00	15.00	B	N
ATOM	4966	CA	ARG	B	12	-31.702	-5.119	-6.950	1.00	15.00	B	C
ATOM	4967	CB	ARG	B	12	-30.267	-5.661	-6.982	1.00	15.00	B	C
ATOM	4968	CG	ARG	B	12	-29.942	-6.619	-5.844	1.00	15.00	B	C
ATOM	4969	CD	ARG	B	12	-29.419	-7.951	-6.361	1.00	15.00	B	C
ATOM	4970	NE	ARG	B	12	-27.962	-8.072	-6.244	1.00	15.00	B	N
ATOM	4971	CZ	ARG	B	12	-27.196	-8.715	-7.129	1.00	15.00	B	C
ATOM	4972	NH1	ARG	B	12	-27.748	-9.286	-8.194	1.00	15.00	B	N
ATOM	4973	NH2	ARG	B	12	-25.882	-8.803	-6.942	1.00	15.00	B	N
ATOM	4974	C	ARG	B	12	-31.777	-3.756	-7.631	1.00	15.00	B	C
ATOM	4975	O	ARG	B	12	-31.503	-2.730	-7.015	1.00	15.00	B	O
ATOM	4976	N	ALA	B	13	-32.150	-3.754	-8.905	1.00	15.00	B	N
ATOM	4977	CA	ALA	B	13	-32.273	-2.517	-9.663	1.00	15.00	B	C
ATOM	4978	CB	ALA	B	13	-31.907	-2.748	-11.121	1.00	15.00	B	C
ATOM	4979	C	ALA	B	13	-33.689	-1.956	-9.548	1.00	15.00	B	C
ATOM	4980	O	ALA	B	13	-33.922	-0.766	-9.781	1.00	15.00	B	O
ATOM	4981	N	LEU	B	14	-34.630	-2.821	-9.190	1.00	15.00	B	N
ATOM	4982	CA	LEU	B	14	-36.023	-2.431	-9.035	1.00	15.00	B	C
ATOM	4983	CB	LEU	B	14	-36.931	-3.669	-9.023	1.00	15.00	B	C
ATOM	4984	CG	LEU	B	14	-38.440	-3.438	-9.196	1.00	15.00	B	C
ATOM	4985	CD1	LEU	B	14	-39.099	-3.076	-7.874	1.00	15.00	B	C
ATOM	4986	CD2	LEU	B	14	-38.718	-2.375	-10.251	1.00	15.00	B	C
ATOM	4987	C	LEU	B	14	-36.198	-1.619	-7.761	1.00	15.00	B	C
ATOM	4988	O	LEU	B	14	-36.651	-0.477	-7.803	1.00	15.00	B	O
ATOM	4989	N	ILE	B	15	-35.820	-2.201	-6.630	1.00	15.00	B	N
ATOM	4990	CA	ILE	B	15	-35.934	-1.506	-5.355	1.00	15.00	B	C
ATOM	4991	CB	ILE	B	15	-35.616	-2.424	-4.151	1.00	15.00	B	C
ATOM	4992	CG1	ILE	B	15	-35.743	-1.651	-2.832	1.00	15.00	B	C
ATOM	4993	CG2	ILE	B	15	-34.238	-3.060	-4.289	1.00	15.00	B	C
ATOM	4994	CD1	ILE	B	15	-35.656	-2.516	-1.596	1.00	15.00	B	C
ATOM	4995	C	ILE	B	15	-35.027	-0.276	-5.354	1.00	15.00	B	C
ATOM	4996	O	ILE	B	15	-35.294	0.715	-4.674	1.00	15.00	B	O
ATOM	4997	N	LYS	B	16	-33.980	-0.342	-6.169	1.00	15.00	B	N
ATOM	4998	CA	LYS	B	16	-33.026	0.744	-6.301	1.00	15.00	B	C
ATOM	4999	CB	LYS	B	16	-31.907	0.346	-7.266	1.00	15.00	B	C
ATOM	5000	CG	LYS	B	16	-30.894	1.442	-7.534	1.00	15.00	B	C
ATOM	5001	CD	LYS	B	16	-30.189	1.864	-6.257	1.00	15.00	B	C
ATOM	5002	CE	LYS	B	16	-29.679	3.287	-6.369	1.00	15.00	B	C
ATOM	5003	NZ	LYS	B	16	-30.794	4.266	-6.384	1.00	15.00	B	N
ATOM	5004	C	LYS	B	16	-33.703	2.027	-6.780	1.00	15.00	B	C
ATOM	5005	O	LYS	B	16	-33.330	3.125	-6.367	1.00	15.00	B	O
ATOM	5006	N	ARG	B	17	-34.697	1.896	-7.652	1.00	15.00	B	N
ATOM	5007	CA	ARG	B	17	-35.400	3.069	-8.159	1.00	15.00	B	C
ATOM	5008	CB	ARG	B	17	-35.962	2.860	-9.571	1.00	15.00	B	C
ATOM	5009	CG	ARG	B	17	-36.995	1.755	-9.702	1.00	15.00	B	C
ATOM	5010	CD	ARG	B	17	-36.949	1.129	-11.086	1.00	15.00	B	C
ATOM	5011	NE	ARG	B	17	-35.611	0.627	-11.411	1.00	15.00	B	N
ATOM	5012	CZ	ARG	B	17	-35.041	0.728	-12.611	1.00	15.00	B	C
ATOM	5013	NH1	ARG	B	17	-35.690	1.312	-13.614	1.00	15.00	B	N
ATOM	5014	NH2	ARG	B	17	-33.813	0.256	-12.800	1.00	15.00	B	N
ATOM	5015	C	ARG	B	17	-36.465	3.541	-7.177	1.00	15.00	B	C
ATOM	5016	O	ARG	B	17	-36.757	4.731	-7.094	1.00	15.00	B	O
ATOM	5017	N	ILE	B	18	-37.030	2.603	-6.422	1.00	15.00	B	N
ATOM	5018	CA	ILE	B	18	-38.039	2.940	-5.425	1.00	15.00	B	C
ATOM	5019	CB	ILE	B	18	-38.709	1.678	-4.839	1.00	15.00	B	C
ATOM	5020	CG1	ILE	B	18	-39.365	0.856	-5.953	1.00	15.00	B	C
ATOM	5021	CG2	ILE	B	18	-39.735	2.057	-3.777	1.00	15.00	B	C
ATOM	5022	CD1	ILE	B	18	-40.002	-0.431	-5.474	1.00	15.00	B	C
ATOM	5023	C	ILE	B	18	-37.373	3.732	-4.305	1.00	15.00	B	C
ATOM	5024	O	ILE	B	18	-37.885	4.760	-3.858	1.00	15.00	B	O
ATOM	5025	N	GLN	B	19	-36.211	3.248	-3.878	1.00	15.00	B	N
ATOM	5026	CA	GLN	B	19	-35.442	3.904	-2.834	1.00	15.00	B	C
ATOM	5027	CB	GLN	B	19	-34.259	3.026	-2.410	1.00	15.00	B	C
ATOM	5028	CG	GLN	B	19	-33.192	3.760	-1.610	1.00	15.00	B	C
ATOM	5029	CD	GLN	B	19	-33.564	3.947	-0.153	1.00	15.00	B	C

ATOM	5030	OE1	GLN	B	19	-34.264	4.892	0.207	1.00	15.00	B	O
ATOM	5031	NE2	GLN	B	19	-33.082	3.056	0.694	1.00	15.00	B	N
ATOM	5032	C	GLN	B	19	-34.937	5.252	-3.333	1.00	15.00	B	C
ATOM	5033	O	GLN	B	19	-34.926	6.225	-2.593	1.00	15.00	B	O
ATOM	5034	N	ALA	B	20	-34.548	5.301	-4.606	1.00	15.00	B	N
ATOM	5035	CA	ALA	B	20	-34.038	6.528	-5.215	1.00	15.00	B	C
ATOM	5036	CB	ALA	B	20	-33.672	6.293	-6.673	1.00	15.00	B	C
ATOM	5037	C	ALA	B	20	-35.041	7.669	-5.099	1.00	15.00	B	C
ATOM	5038	O	ALA	B	20	-34.662	8.832	-4.946	1.00	15.00	B	O
ATOM	5039	N	LEU	B	21	-36.316	7.330	-5.147	1.00	15.00	B	N
ATOM	5040	CA	LEU	B	21	-37.370	8.323	-5.048	1.00	15.00	B	C
ATOM	5041	CB	LEU	B	21	-38.648	7.813	-5.721	1.00	15.00	B	C
ATOM	5042	CG	LEU	B	21	-38.548	7.492	-7.214	1.00	15.00	B	C
ATOM	5043	CD1	LEU	B	21	-39.837	6.854	-7.710	1.00	15.00	B	C
ATOM	5044	CD2	LEU	B	21	-38.226	8.744	-8.015	1.00	15.00	B	C
ATOM	5045	C	LEU	B	21	-37.652	8.673	-3.593	1.00	15.00	B	C
ATOM	5046	O	LEU	B	21	-37.665	9.845	-3.219	1.00	15.00	B	O
ATOM	5047	N	ILE	B	22	-37.858	7.650	-2.775	1.00	15.00	B	N
ATOM	5048	CA	ILE	B	22	-38.162	7.845	-1.362	1.00	15.00	B	C
ATOM	5049	CB	ILE	B	22	-38.772	6.571	-0.733	1.00	15.00	B	C
ATOM	5050	CG1	ILE	B	22	-40.007	6.113	-1.520	1.00	15.00	B	C
ATOM	5051	CG2	ILE	B	22	-39.122	6.799	0.733	1.00	15.00	B	C
ATOM	5052	CD1	ILE	B	22	-41.112	7.147	-1.603	1.00	15.00	B	C
ATOM	5053	C	ILE	B	22	-36.924	8.267	-0.570	1.00	15.00	B	C
ATOM	5054	O	ILE	B	22	-35.953	7.525	-0.469	1.00	15.00	B	O
ATOM	5055	N	PRO	B	23	-36.934	9.483	-0.016	1.00	15.00	B	N
ATOM	5056	CA	PRO	B	23	-35.833	9.990	0.779	1.00	15.00	B	C
ATOM	5057	CB	PRO	B	23	-35.907	11.501	0.535	1.00	15.00	B	C
ATOM	5058	CG	PRO	B	23	-37.318	11.786	0.106	1.00	15.00	B	C
ATOM	5059	CD	PRO	B	23	-38.017	10.466	-0.119	1.00	15.00	B	C
ATOM	5060	C	PRO	B	23	-36.038	9.675	2.257	1.00	15.00	B	C
ATOM	5061	O	PRO	B	23	-36.941	10.224	2.890	1.00	15.00	B	O
ATOM	5062	N	LYS	B	24	-35.200	8.782	2.791	1.00	15.00	B	N
ATOM	5063	CA	LYS	B	24	-35.281	8.368	4.194	1.00	15.00	B	C
ATOM	5064	CB	LYS	B	24	-35.300	9.577	5.147	1.00	15.00	B	C
ATOM	5065	CG	LYS	B	24	-34.037	10.429	5.105	1.00	15.00	B	C
ATOM	5066	CD	LYS	B	24	-34.276	11.834	5.643	1.00	15.00	B	C
ATOM	5067	CE	LYS	B	24	-34.508	11.842	7.148	1.00	15.00	B	C
ATOM	5068	NZ	LYS	B	24	-34.409	13.218	7.714	1.00	15.00	B	N
ATOM	5069	C	LYS	B	24	-36.503	7.477	4.406	1.00	15.00	B	C
ATOM	5070	O	LYS	B	24	-37.527	7.921	4.926	1.00	15.00	B	O
ATOM	5071	N	GLY	B	25	-36.387	6.217	3.998	1.00	15.00	B	N
ATOM	5072	CA	GLY	B	25	-37.501	5.297	4.121	1.00	15.00	B	C
ATOM	5073	C	GLY	B	25	-37.218	4.114	5.026	1.00	15.00	B	C
ATOM	5074	O	GLY	B	25	-38.134	3.577	5.654	1.00	15.00	B	O
ATOM	5075	N	GLY	B	26	-35.963	3.694	5.087	1.00	15.00	B	N
ATOM	5076	CA	GLY	B	26	-35.599	2.569	5.927	1.00	15.00	B	C
ATOM	5077	C	GLY	B	26	-35.565	1.255	5.168	1.00	15.00	B	C
ATOM	5078	O	GLY	B	26	-36.394	0.371	5.398	1.00	15.00	B	O
ATOM	5079	N	ARG	B	27	-34.631	1.135	4.239	1.00	15.00	B	N
ATOM	5080	CA	ARG	B	27	-34.473	-0.075	3.450	1.00	15.00	B	C
ATOM	5081	CB	ARG	B	27	-34.779	0.196	1.970	1.00	15.00	B	C
ATOM	5082	CG	ARG	B	27	-36.112	0.885	1.710	1.00	15.00	B	C
ATOM	5083	CD	ARG	B	27	-37.284	0.036	2.181	1.00	15.00	B	C
ATOM	5084	NE	ARG	B	27	-38.561	0.738	2.038	1.00	15.00	B	N
ATOM	5085	CZ	ARG	B	27	-39.249	1.250	3.062	1.00	15.00	B	C
ATOM	5086	NH1	ARG	B	27	-38.788	1.136	4.302	1.00	15.00	B	N
ATOM	5087	NH2	ARG	B	27	-40.401	1.878	2.844	1.00	15.00	B	N
ATOM	5088	C	ARG	B	27	-33.048	-0.596	3.593	1.00	15.00	B	C
ATOM	5089	O	ARG	B	27	-32.100	0.182	3.660	1.00	15.00	B	O
ATOM	5090	N	LEU	B	28	-32.898	-1.911	3.648	1.00	15.00	B	N
ATOM	5091	CA	LEU	B	28	-31.579	-2.518	3.781	1.00	15.00	B	C
ATOM	5092	CB	LEU	B	28	-31.684	-3.918	4.403	1.00	15.00	B	C
ATOM	5093	CG	LEU	B	28	-31.799	-3.991	5.931	1.00	15.00	B	C
ATOM	5094	CD1	LEU	B	28	-30.712	-3.164	6.602	1.00	15.00	B	C
ATOM	5095	CD2	LEU	B	28	-33.182	-3.566	6.404	1.00	15.00	B	C
ATOM	5096	C	LEU	B	28	-30.882	-2.595	2.425	1.00	15.00	B	C
ATOM	5097	O	LEU	B	28	-31.385	-2.067	1.430	1.00	15.00	B	O
ATOM	5098	N	LEU	B	29	-29.722	-3.248	2.392	1.00	15.00	B	N
ATOM	5099	CA	LEU	B	29	-28.968	-3.404	1.153	1.00	15.00	B	C
ATOM	5100	CB	LEU	B	29	-27.645	-4.148	1.397	1.00	15.00	B	C

ATOM	5101	CG	LEU	B	29	-26.499	-3.343	2.033	1.00	15.00	B	C
ATOM	5102	CD1	LEU	B	29	-26.351	-1.979	1.376	1.00	15.00	B	C
ATOM	5103	CD2	LEU	B	29	-26.682	-3.209	3.537	1.00	15.00	B	C
ATOM	5104	C	LEU	B	29	-29.816	-4.143	0.118	1.00	15.00	B	C
ATOM	5105	O	LEU	B	29	-30.585	-5.039	0.472	1.00	15.00	B	O
ATOM	5106	N	PRO	B	30	-29.681	-3.778	-1.169	1.00	15.00	B	N
ATOM	5107	CA	PRO	B	30	-30.453	-4.375	-2.268	1.00	15.00	B	C
ATOM	5108	CB	PRO	B	30	-29.729	-3.896	-3.537	1.00	15.00	B	C
ATOM	5109	CG	PRO	B	30	-28.487	-3.214	-3.064	1.00	15.00	B	C
ATOM	5110	CD	PRO	B	30	-28.763	-2.747	-1.666	1.00	15.00	B	C
ATOM	5111	C	PRO	B	30	-30.516	-5.903	-2.225	1.00	15.00	B	C
ATOM	5112	O	PRO	B	30	-31.596	-6.488	-2.305	1.00	15.00	B	O
ATOM	5113	N	GLN	B	31	-29.363	-6.548	-2.078	1.00	15.00	B	N
ATOM	5114	CA	GLN	B	31	-29.314	-8.006	-2.040	1.00	15.00	B	C
ATOM	5115	CB	GLN	B	31	-27.870	-8.518	-2.103	1.00	15.00	B	C
ATOM	5116	CG	GLN	B	31	-27.742	-9.955	-2.600	1.00	15.00	B	C
ATOM	5117	CD	GLN	B	31	-27.927	-10.990	-1.502	1.00	15.00	B	C
ATOM	5118	OE1	GLN	B	31	-28.524	-12.044	-1.720	1.00	15.00	B	O
ATOM	5119	NE2	GLN	B	31	-27.403	-10.705	-0.319	1.00	15.00	B	N
ATOM	5120	C	GLN	B	31	-30.033	-8.556	-0.812	1.00	15.00	B	C
ATOM	5121	O	GLN	B	31	-30.699	-9.585	-0.887	1.00	15.00	B	O
ATOM	5122	N	LEU	B	32	-29.914	-7.853	0.308	1.00	15.00	B	N
ATOM	5123	CA	LEU	B	32	-30.549	-8.283	1.547	1.00	15.00	B	C
ATOM	5124	CB	LEU	B	32	-30.126	-7.396	2.722	1.00	15.00	B	C
ATOM	5125	CG	LEU	B	32	-28.643	-7.436	3.103	1.00	15.00	B	C
ATOM	5126	CD1	LEU	B	32	-28.379	-6.545	4.307	1.00	15.00	B	C
ATOM	5127	CD2	LEU	B	32	-28.195	-8.863	3.383	1.00	15.00	B	C
ATOM	5128	C	LEU	B	32	-32.067	-8.291	1.407	1.00	15.00	B	C
ATOM	5129	O	LEU	B	32	-32.727	-9.253	1.793	1.00	15.00	B	O
ATOM	5130	N	VAL	B	33	-32.613	-7.227	0.831	1.00	15.00	B	N
ATOM	5131	CA	VAL	B	33	-34.056	-7.124	0.642	1.00	15.00	B	C
ATOM	5132	CB	VAL	B	33	-34.489	-5.713	0.196	1.00	15.00	B	C
ATOM	5133	CG1	VAL	B	33	-36.008	-5.611	0.159	1.00	15.00	B	C
ATOM	5134	CG2	VAL	B	33	-33.910	-4.660	1.129	1.00	15.00	B	C
ATOM	5135	C	VAL	B	33	-34.545	-8.166	-0.363	1.00	15.00	B	C
ATOM	5136	O	VAL	B	33	-35.557	-8.833	-0.139	1.00	15.00	B	O
ATOM	5137	N	CYS	B	34	-33.807	-8.324	-1.458	1.00	15.00	B	N
ATOM	5138	CA	CYS	B	34	-34.161	-9.301	-2.485	1.00	15.00	B	C
ATOM	5139	C	CYS	B	34	-34.080	-10.723	-1.932	1.00	15.00	B	C
ATOM	5140	O	CYS	B	34	-34.697	-11.650	-2.465	1.00	15.00	B	O
ATOM	5141	CB	CYS	B	34	-33.263	-9.152	-3.713	1.00	15.00	B	C
ATOM	5142	SG	CYS	B	34	-33.518	-7.612	-4.652	1.00	15.00	B	S
ATOM	5143	N	ARG	B	35	-33.311	-10.884	-0.863	1.00	15.00	B	N
ATOM	5144	CA	ARG	B	35	-33.153	-12.173	-0.213	1.00	15.00	B	C
ATOM	5145	CB	ARG	B	35	-31.830	-12.213	0.559	1.00	15.00	B	C
ATOM	5146	CG	ARG	B	35	-31.417	-13.598	1.026	1.00	15.00	B	C
ATOM	5147	CD	ARG	B	35	-30.038	-13.963	0.502	1.00	15.00	B	C
ATOM	5148	NE	ARG	B	35	-30.014	-15.313	-0.058	1.00	15.00	B	N
ATOM	5149	CZ	ARG	B	35	-29.813	-15.580	-1.352	1.00	15.00	B	C
ATOM	5150	NH1	ARG	B	35	-29.611	-14.594	-2.217	1.00	15.00	B	N
ATOM	5151	NH2	ARG	B	35	-29.808	-16.839	-1.778	1.00	15.00	B	N
ATOM	5152	C	ARG	B	35	-34.317	-12.419	0.741	1.00	15.00	B	C
ATOM	5153	O	ARG	B	35	-34.774	-13.547	0.899	1.00	15.00	B	O
ATOM	5154	N	LEU	B	36	-34.799	-11.343	1.356	1.00	15.00	B	N
ATOM	5155	CA	LEU	B	36	-35.911	-11.422	2.299	1.00	15.00	B	C
ATOM	5156	CB	LEU	B	36	-36.082	-10.096	3.043	1.00	15.00	B	C
ATOM	5157	CG	LEU	B	36	-34.926	-9.679	3.954	1.00	15.00	B	C
ATOM	5158	CD1	LEU	B	36	-35.154	-8.278	4.502	1.00	15.00	B	C
ATOM	5159	CD2	LEU	B	36	-34.746	-10.679	5.086	1.00	15.00	B	C
ATOM	5160	C	LEU	B	36	-37.204	-11.798	1.592	1.00	15.00	B	C
ATOM	5161	O	LEU	B	36	-38.024	-12.538	2.131	1.00	15.00	B	O
ATOM	5162	N	VAL	B	37	-37.381	-11.288	0.379	1.00	15.00	B	N
ATOM	5163	CA	VAL	B	37	-38.574	-11.591	-0.404	1.00	15.00	B	C
ATOM	5164	CB	VAL	B	37	-38.882	-10.503	-1.457	1.00	15.00	B	C
ATOM	5165	CG1	VAL	B	37	-39.207	-9.180	-0.780	1.00	15.00	B	C
ATOM	5166	CG2	VAL	B	37	-37.729	-10.339	-2.433	1.00	15.00	B	C
ATOM	5167	C	VAL	B	37	-38.432	-12.954	-1.084	1.00	15.00	B	C
ATOM	5168	O	VAL	B	37	-39.347	-13.423	-1.760	1.00	15.00	B	O
ATOM	5169	N	LEU	B	38	-37.260	-13.564	-0.898	1.00	15.00	B	N
ATOM	5170	CA	LEU	B	38	-36.936	-14.878	-1.456	1.00	15.00	B	C
ATOM	5171	CB	LEU	B	38	-37.866	-15.966	-0.902	1.00	15.00	B	C

```
ATOM   5172  CG  LEU B  38     -37.290 -17.385  -0.832  1.00 15.00      B    C
ATOM   5173  CD1 LEU B  38     -36.012 -17.408  -0.004  1.00 15.00      B    C
ATOM   5174  CD2 LEU B  38     -38.317 -18.350  -0.257  1.00 15.00      B    C
ATOM   5175  C   LEU B  38     -36.910 -14.891  -2.987  1.00 15.00      B    C
ATOM   5176  O   LEU B  38     -36.816 -15.951  -3.605  1.00 15.00      B    O
ATOM   5177  N   ARG B  39     -36.964 -13.715  -3.604  1.00 15.00      B    N
ATOM   5178  CA  ARG B  39     -36.946 -13.638  -5.058  1.00 15.00      B    C
ATOM   5179  CB  ARG B  39     -37.555 -12.332  -5.578  1.00 15.00      B    C
ATOM   5180  CG  ARG B  39     -37.976 -12.386  -7.043  1.00 15.00      B    C
ATOM   5181  CD  ARG B  39     -38.668 -13.704  -7.387  1.00 15.00      B    C
ATOM   5182  NE  ARG B  39     -39.061 -13.763  -8.799  1.00 15.00      B    N
ATOM   5183  CZ  ARG B  39     -39.394 -14.881  -9.450  1.00 15.00      B    C
ATOM   5184  NH1 ARG B  39     -39.377 -16.056  -8.826  1.00 15.00      B    N
ATOM   5185  NH2 ARG B  39     -39.771 -14.814 -10.724  1.00 15.00      B    N
ATOM   5186  C   ARG B  39     -35.543 -13.871  -5.612  1.00 15.00      B    C
ATOM   5187  O   ARG B  39     -35.380 -14.384  -6.716  1.00 15.00      B    O
ATOM   5188  N   CYS B  40     -34.527 -13.499  -4.846  1.00 15.00      B    N
ATOM   5189  CA  CYS B  40     -33.152 -13.709  -5.274  1.00 15.00      B    C
ATOM   5190  C   CYS B  40     -32.667 -15.069  -4.784  1.00 15.00      B    C
ATOM   5191  O   CYS B  40     -31.910 -15.164  -3.826  1.00 15.00      B    O
ATOM   5192  CB  CYS B  40     -32.221 -12.590  -4.789  1.00 15.00      B    C
ATOM   5193  SG  CYS B  40     -30.466 -12.820  -5.241  1.00 15.00      B    S
ATOM   5194  N   SER B  41     -33.143 -16.121  -5.432  1.00 15.00      B    N
ATOM   5195  CA  SER B  41     -32.772 -17.480  -5.069  1.00 15.00      B    C
ATOM   5196  CB  SER B  41     -33.660 -18.469  -5.818  1.00 15.00      B    C
ATOM   5197  OG  SER B  41     -34.988 -17.980  -5.894  1.00 15.00      B    O
ATOM   5198  C   SER B  41     -31.298 -17.759  -5.361  1.00 15.00      B    C
ATOM   5199  O   SER B  41     -30.431 -17.572  -4.496  1.00 15.00      B    O
TER    5200      SER B  41
END
```

Prodigy Webserver

[https://wenmr.science.uu.nl › prodigy](https://wenmr.science.uu.nl)

Prodigy (protein-protein)

Prodigy Protein- Protein Binding-Affinity Predictions for ACE2 – SMB

[+] No. of intermolecular contacts: 53
[+] No. of charged-charged contacts: 10
[+] No. of charged-polar contacts: 7
[+] No. of charged-apolar contacts: 27
[+] No. of polar-polar contacts: 0
[+] No. of apolar-polar contacts: 6
[+] No. of apolar-apolar contacts: 3
[+] Percentage of apolar NIS residues: 35.85
[+] Percentage of charged NIS residues: 27.43
[++] Predicted binding affinity (kcal.mol-1):   -10.5
[++] Predicted dissociation constant (M) at 37.0˚C: 4.2e-08

ACE2 (A chain)			SMB (B chain)		
THR	27	A	PRO	23	B
TYR	83	A	LYS	24	B
LYS	353	A	PRO	30	B
LYS	31	A	PRO	23	B
ARG	393	A	LEU	29	B
GLY	354	A	ARG	12	B
ALA	25	A	LYS	24	B
LYS	31	A	ALA	20	B
ASP	30	A	GLY	25	B
LYS	31	A	LEU	21	B
HIS	34	A	LEU	28	B
PHE	28	A	LYS	24	B
PRO	389	A	LEU	29	B
LYS	353	A	GLN	31	B
LEU	39	A	LYS	16	B
HIS	34	A	VAL	33	B
LYS	31	A	GLN	19	B
HIS	34	A	ARG	12	B
ALA	387	A	LEU	29	B
GLU	35	A	ILE	22	B
GLN	24	A	LYS	24	B
GLN	388	A	LEU	29	B
THR	27	A	GLY	26	B
ASP	30	A	GLN	19	B
GLU	35	A	ALA	20	B
HIS	34	A	PRO	30	B
ASN	33	A	PRO	30	B
GLU	37	A	PRO	30	B
THR	27	A	LYS	24	B
ASP	30	A	ARG	27	B
GLU	35	A	GLN	19	B
PHE	390	A	LEU	29	B
LYS	353	A	ALA	13	B
ASP	30	A	LEU	28	B
GLU	35	A	LYS	16	B
LYS	31	A	LYS	24	B
ASP	30	A	GLY	26	B
LYS	31	A	ILE	22	B
HIS	34	A	GLN	19	B
ALA	36	A	LYS	16	B
ASP	38	A	LYS	16	B
HIS	34	A	LEU	29	B
ASN	33	A	LEU	29	B
HIS	34	A	LYS	16	B
GLU	37	A	LEU	29	B
GLU	35	A	LEU	21	B
HIS	34	A	ARG	27	B
ASP	30	A	LYS	24	B

LYS	353	A	ARG	12	B
HIS	34	A	ILE	15	B
ASP	38	A	ARG	12	B
THR	27	A	GLY	25	B
ASP	38	A	ALA	13	B

Binding data of SMB surfactant peptide with hACE2 receptor protein derived from experimentally determined peptide-protein interaction data using Surface Plasmon Resonance.

	ka (1/Ms)	kd (1/s)	Rmax (RU)	RI (RU)	Conc of analyte	KA (1/M)	KD (M)	Req (RU)	kobs (1/s)
0.5µg/ml SMB ox to hACE2	6.2e3	6.12e-5	6.48e3	5.89	1.05e-7	1.01e8	9.87e-9	5.93e3	7.12e-4

Surface Plasmon Resonance metrics derived from the time course of binding of the ACE2 to the SMB peptide. Association and dissociation kinetic rate constants (k_{on}, k_{off}) and equilibrium dissociation constants (K_D), calculated from surface plasmon resonance (SPR) kinetic measurements for the hACE2 was attached to the Biacore sensor chip while SMB and BYL peptides were flowed in the anylate. Peptide was dissolved in running buffer (10 mM HEPES, 150 mM NaCl, 3 mM EDTA, 0.005% Surfactant P20, pH 7.4) and was flowed past the recombinant protein constructs on the CSM sensor chip with a Biacore system (Methods). Kinetic rate constants and equilibrium dissociation constants were determined from curve fitting analysis of SPR traces.
