# Supplementary material for: Lung Surfactant Protein B Peptide Mimics Interact with the Human ACE2 Receptor
Source: Int J Mol Sci. 2023 Jun 29;24(13):10837. doi: 10.3390/ijms241310837 (PMC10341807; doi:10.3390/ijms241310837)
Supplement: Supplementary file 1 [file ijms-24-10837-s001.zip › File S2_B-YL_ACE2_complex.pdf]

S2. Supplement file - ACE2 – B-YL Haddock Docked Molecular Coordinates and Prodigy Binding Analysis of the predicted complex

Haddock Webserver (version 2.4)

https://wenmr.science.uu.nl/haddock2.4/submit/1

Haddock Predicted Molecular Coordinates of the ACE2 – B-YL Complex based on docking the B-YL helical domains and N-terminal insertion sequences with the ACE2 contact residues of the Covid Receptor Binding Domain.

Summary

HADDOCK clustered **150** structures in **19** cluster(s), which represents **75 %** of the water-refined models HADDOCK generated. Note that currently the maximum number of models considered for clustering is 200.

The top cluster detailed below is the most reliable according to HADDOCK. Its Z-score indicates how many standard deviations from the average this cluster is located in terms of score (the more negative the better).

Best Docked Complex Structure

HADDOCK score	-108.4 +/- 9.5
Cluster size	5
RMSD from the overall lowest-energy structure	0.3 +/- 0.2
Van der Waals energy	-82.6 +/- 6.2
Electrostatic energy	-79.2 +/- 7.1
Desolvation energy	-49.7 +/- 0.7
Restraints violation energy	397.9 +/- 56.1
Buried Surface Area	2496.9 +/- 71.7
Z-Score	-1.5

Docked ACE2 - BYL Complex PDB Coordinates

ACE2 (Chain A) B-YL (Chain B)

ATOM	1	N	SER	A	19	-38.849	-9.297	-10.388	1.00	15.00	A	N
ATOM	2	CA	SER	A	19	-39.329	-10.426	-11.170	1.00	15.00	A	C
ATOM	3	CB	SER	A	19	-40.161	-9.917	-12.348	1.00	15.00	A	C
ATOM	4	OG	SER	A	19	-40.284	-8.504	-12.298	1.00	15.00	A	O
ATOM	5	C	SER	A	19	-38.167	-11.286	-11.666	1.00	15.00	A	C
ATOM	6	O	SER	A	19	-38.039	-12.453	-11.287	1.00	15.00	A	O
ATOM	7	N	THR	A	20	-37.316	-10.704	-12.501	1.00	15.00	A	N
ATOM	8	CA	THR	A	20	-36.168	-11.417	-13.043	1.00	15.00	A	C
ATOM	9	CB	THR	A	20	-35.635	-10.736	-14.319	1.00	15.00	A	C
ATOM	10	OG1	THR	A	20	-35.385	-9.350	-14.054	1.00	15.00	A	O
ATOM	11	CG2	THR	A	20	-36.642	-10.859	-15.452	1.00	15.00	A	C
ATOM	12	C	THR	A	20	-35.052	-11.496	-12.006	1.00	15.00	A	C
ATOM	13	O	THR	A	20	-35.106	-10.816	-10.981	1.00	15.00	A	O
ATOM	14	N	ILE	A	21	-34.040	-12.316	-12.277	1.00	15.00	A	N
ATOM	15	CA	ILE	A	21	-32.917	-12.484	-11.357	1.00	15.00	A	C
ATOM	16	CB	ILE	A	21	-31.867	-13.474	-11.907	1.00	15.00	A	C
ATOM	17	CG1	ILE	A	21	-32.449	-14.891	-11.953	1.00	15.00	A	C
ATOM	18	CG2	ILE	A	21	-30.588	-13.445	-11.080	1.00	15.00	A	C
ATOM	19	CD1	ILE	A	21	-32.932	-15.409	-10.614	1.00	15.00	A	C
ATOM	20	C	ILE	A	21	-32.263	-11.147	-11.008	1.00	15.00	A	C
ATOM	21	O	ILE	A	21	-31.974	-10.879	-9.841	1.00	15.00	A	O
ATOM	22	N	GLU	A	22	-32.060	-10.297	-12.011	1.00	15.00	A	N
ATOM	23	CA	GLU	A	22	-31.448	-8.993	-11.784	1.00	15.00	A	C
ATOM	24	CB	GLU	A	22	-31.206	-8.254	-13.100	1.00	15.00	A	C
ATOM	25	CG	GLU	A	22	-30.311	-7.034	-12.950	1.00	15.00	A	C
ATOM	26	CD	GLU	A	22	-30.370	-6.106	-14.143	1.00	15.00	A	C
ATOM	27	OE1	GLU	A	22	-29.517	-6.238	-15.040	1.00	15.00	A	O
ATOM	28	OE2	GLU	A	22	-31.265	-5.234	-14.173	1.00	15.00	A	O
ATOM	29	C	GLU	A	22	-32.313	-8.145	-10.855	1.00	15.00	A	C
ATOM	30	O	GLU	A	22	-31.803	-7.449	-9.979	1.00	15.00	A	O
ATOM	31	N	GLU	A	23	-33.624	-8.214	-11.048	1.00	15.00	A	N
ATOM	32	CA	GLU	A	23	-34.557	-7.461	-10.223	1.00	15.00	A	C
ATOM	33	CB	GLU	A	23	-35.953	-7.482	-10.837	1.00	15.00	A	C
ATOM	34	CG	GLU	A	23	-36.023	-6.844	-12.213	1.00	15.00	A	C
ATOM	35	CD	GLU	A	23	-37.370	-7.042	-12.872	1.00	15.00	A	C
ATOM	36	OE1	GLU	A	23	-38.168	-6.087	-12.893	1.00	15.00	A	O
ATOM	37	OE2	GLU	A	23	-37.629	-8.161	-13.362	1.00	15.00	A	O
ATOM	38	C	GLU	A	23	-34.584	-8.018	-8.806	1.00	15.00	A	C
ATOM	39	O	GLU	A	23	-34.604	-7.266	-7.833	1.00	15.00	A	O
ATOM	40	N	GLN	A	24	-34.563	-9.341	-8.702	1.00	15.00	A	N
ATOM	41	CA	GLN	A	24	-34.566	-10.011	-7.410	1.00	15.00	A	C
ATOM	42	CB	GLN	A	24	-34.671	-11.525	-7.595	1.00	15.00	A	C
ATOM	43	CG	GLN	A	24	-35.969	-12.000	-8.225	1.00	15.00	A	C
ATOM	44	CD	GLN	A	24	-36.015	-13.508	-8.367	1.00	15.00	A	C
ATOM	45	OE1	GLN	A	24	-35.461	-14.234	-7.544	1.00	15.00	A	O
ATOM	46	NE2	GLN	A	24	-36.668	-13.988	-9.412	1.00	15.00	A	N
ATOM	47	C	GLN	A	24	-33.294	-9.678	-6.639	1.00	15.00	A	C
ATOM	48	O	GLN	A	24	-33.312	-9.536	-5.414	1.00	15.00	A	O
ATOM	49	N	ALA	A	25	-32.190	-9.560	-7.371	1.00	15.00	A	N
ATOM	50	CA	ALA	A	25	-30.902	-9.234	-6.778	1.00	15.00	A	C
ATOM	51	CB	ALA	A	25	-29.787	-9.427	-7.793	1.00	15.00	A	C
ATOM	52	C	ALA	A	25	-30.899	-7.806	-6.248	1.00	15.00	A	C
ATOM	53	O	ALA	A	25	-30.197	-7.494	-5.286	1.00	15.00	A	O
ATOM	54	N	LYS	A	26	-31.694	-6.946	-6.879	1.00	15.00	A	N
ATOM	55	CA	LYS	A	26	-31.794	-5.553	-6.463	1.00	15.00	A	C
ATOM	56	CB	LYS	A	26	-32.648	-4.746	-7.445	1.00	15.00	A	C
ATOM	57	CG	LYS	A	26	-31.930	-4.422	-8.747	1.00	15.00	A	C
ATOM	58	CD	LYS	A	26	-32.894	-4.330	-9.918	1.00	15.00	A	C
ATOM	59	CE	LYS	A	26	-32.145	-4.239	-11.237	1.00	15.00	A	C
ATOM	60	NZ	LYS	A	26	-33.012	-4.561	-12.404	1.00	15.00	A	N
ATOM	61	C	LYS	A	26	-32.340	-5.460	-5.044	1.00	15.00	A	C
ATOM	62	O	LYS	A	26	-31.806	-4.723	-4.219	1.00	15.00	A	O
ATOM	63	N	THR	A	27	-33.389	-6.231	-4.762	1.00	15.00	A	N
ATOM	64	CA	THR	A	27	-33.986	-6.260	-3.431	1.00	15.00	A	C

ATOM	65	CB	THR	A	27	-35.200	-7.223	-3.390	1.00	15.00	A	C
ATOM	66	OG1	THR	A	27	-36.276	-6.697	-4.181	1.00	15.00	A	O
ATOM	67	CG2	THR	A	27	-35.687	-7.449	-1.964	1.00	15.00	A	C
ATOM	68	C	THR	A	27	-32.944	-6.719	-2.408	1.00	15.00	A	C
ATOM	69	O	THR	A	27	-32.866	-6.195	-1.295	1.00	15.00	A	O
ATOM	70	N	PHE	A	28	-32.128	-7.684	-2.815	1.00	15.00	A	N
ATOM	71	CA	PHE	A	28	-31.092	-8.234	-1.955	1.00	15.00	A	C
ATOM	72	CB	PHE	A	28	-30.507	-9.504	-2.586	1.00	15.00	A	C
ATOM	73	CG	PHE	A	28	-29.355	-10.100	-1.828	1.00	15.00	A	C
ATOM	74	CD1	PHE	A	28	-29.562	-10.746	-0.622	1.00	15.00	A	C
ATOM	75	CD2	PHE	A	28	-28.064	-10.013	-2.325	1.00	15.00	A	C
ATOM	76	CE1	PHE	A	28	-28.504	-11.295	0.077	1.00	15.00	A	C
ATOM	77	CE2	PHE	A	28	-27.002	-10.559	-1.632	1.00	15.00	A	C
ATOM	78	CZ	PHE	A	28	-27.222	-11.200	-0.429	1.00	15.00	A	C
ATOM	79	C	PHE	A	28	-29.986	-7.216	-1.679	1.00	15.00	A	C
ATOM	80	O	PHE	A	28	-29.672	-6.928	-0.525	1.00	15.00	A	O
ATOM	81	N	LEU	A	29	-29.414	-6.660	-2.742	1.00	15.00	A	N
ATOM	82	CA	LEU	A	29	-28.329	-5.692	-2.612	1.00	15.00	A	C
ATOM	83	CB	LEU	A	29	-27.709	-5.368	-3.972	1.00	15.00	A	C
ATOM	84	CG	LEU	A	29	-26.873	-6.480	-4.610	1.00	15.00	A	C
ATOM	85	CD1	LEU	A	29	-26.303	-6.018	-5.940	1.00	15.00	A	C
ATOM	86	CD2	LEU	A	29	-25.757	-6.923	-3.674	1.00	15.00	A	C
ATOM	87	C	LEU	A	29	-28.757	-4.417	-1.894	1.00	15.00	A	C
ATOM	88	O	LEU	A	29	-27.992	-3.864	-1.102	1.00	15.00	A	O
ATOM	89	N	ASP	A	30	-29.970	-3.951	-2.171	1.00	15.00	A	N
ATOM	90	CA	ASP	A	30	-30.485	-2.740	-1.535	1.00	15.00	A	C
ATOM	91	CB	ASP	A	30	-31.853	-2.363	-2.110	1.00	15.00	A	C
ATOM	92	CG	ASP	A	30	-32.416	-1.093	-1.500	1.00	15.00	A	C
ATOM	93	OD1	ASP	A	30	-32.863	-1.142	-0.333	1.00	15.00	A	O
ATOM	94	OD2	ASP	A	30	-32.414	-0.052	-2.188	1.00	15.00	A	O
ATOM	95	C	ASP	A	30	-30.591	-2.939	-0.028	1.00	15.00	A	C
ATOM	96	O	ASP	A	30	-30.086	-2.132	0.760	1.00	15.00	A	O
ATOM	97	N	LYS	A	31	-31.227	-4.035	0.368	1.00	15.00	A	N
ATOM	98	CA	LYS	A	31	-31.397	-4.346	1.777	1.00	15.00	A	C
ATOM	99	CB	LYS	A	31	-32.298	-5.569	1.962	1.00	15.00	A	C
ATOM	100	CG	LYS	A	31	-33.178	-5.498	3.200	1.00	15.00	A	C
ATOM	101	CD	LYS	A	31	-34.083	-4.275	3.164	1.00	15.00	A	C
ATOM	102	CE	LYS	A	31	-34.254	-3.669	4.548	1.00	15.00	A	C
ATOM	103	NZ	LYS	A	31	-32.971	-3.147	5.087	1.00	15.00	A	N
ATOM	104	C	LYS	A	31	-30.043	-4.560	2.447	1.00	15.00	A	C
ATOM	105	O	LYS	A	31	-29.808	-4.075	3.556	1.00	15.00	A	O
ATOM	106	N	PHE	A	32	-29.153	-5.263	1.752	1.00	15.00	A	N
ATOM	107	CA	PHE	A	32	-27.812	-5.534	2.260	1.00	15.00	A	C
ATOM	108	CB	PHE	A	32	-27.033	-6.406	1.265	1.00	15.00	A	C
ATOM	109	CG	PHE	A	32	-25.548	-6.447	1.505	1.00	15.00	A	C
ATOM	110	CD1	PHE	A	32	-25.017	-7.215	2.526	1.00	15.00	A	C
ATOM	111	CD2	PHE	A	32	-24.685	-5.713	0.709	1.00	15.00	A	C
ATOM	112	CE1	PHE	A	32	-23.655	-7.252	2.750	1.00	15.00	A	C
ATOM	113	CE2	PHE	A	32	-23.322	-5.745	0.928	1.00	15.00	A	C
ATOM	114	CZ	PHE	A	32	-22.805	-6.516	1.950	1.00	15.00	A	C
ATOM	115	C	PHE	A	32	-27.060	-4.233	2.522	1.00	15.00	A	C
ATOM	116	O	PHE	A	32	-26.406	-4.081	3.552	1.00	15.00	A	O
ATOM	117	N	ASN	A	33	-27.171	-3.294	1.585	1.00	15.00	A	N
ATOM	118	CA	ASN	A	33	-26.502	-2.000	1.699	1.00	15.00	A	C
ATOM	119	CB	ASN	A	33	-26.826	-1.121	0.483	1.00	15.00	A	C
ATOM	120	CG	ASN	A	33	-25.745	-1.150	-0.582	1.00	15.00	A	C
ATOM	121	OD1	ASN	A	33	-24.847	-0.309	-0.596	1.00	15.00	A	O
ATOM	122	ND2	ASN	A	33	-25.826	-2.115	-1.487	1.00	15.00	A	N
ATOM	123	C	ASN	A	33	-26.913	-1.280	2.977	1.00	15.00	A	C
ATOM	124	O	ASN	A	33	-26.083	-0.682	3.660	1.00	15.00	A	O
ATOM	125	N	HIS	A	34	-28.195	-1.368	3.300	1.00	15.00	A	N
ATOM	126	CA	HIS	A	34	-28.743	-0.725	4.488	1.00	15.00	A	C
ATOM	127	CB	HIS	A	34	-30.271	-0.831	4.484	1.00	15.00	A	C
ATOM	128	CG	HIS	A	34	-30.967	0.223	3.676	1.00	15.00	A	C
ATOM	129	ND1	HIS	A	34	-31.289	0.095	2.340	1.00	15.00	A	N
ATOM	130	CD2	HIS	A	34	-31.429	1.442	4.053	1.00	15.00	A	C
ATOM	131	CE1	HIS	A	34	-31.925	1.212	1.961	1.00	15.00	A	C
ATOM	132	NE2	HIS	A	34	-32.036	2.060	2.967	1.00	15.00	A	N
ATOM	133	C	HIS	A	34	-28.187	-1.328	5.778	1.00	15.00	A	C
ATOM	134	O	HIS	A	34	-27.624	-0.621	6.613	1.00	15.00	A	O
ATOM	135	N	GLU	A	35	-28.334	-2.640	5.926	1.00	15.00	A	N

ATOM	136	CA	GLU	A	35	-27.875	-3.338	7.124	1.00	15.00	A	C
ATOM	137	CB	GLU	A	35	-28.386	-4.782	7.141	1.00	15.00	A	C
ATOM	138	CG	GLU	A	35	-29.782	-4.951	7.732	1.00	15.00	A	C
ATOM	139	CD	GLU	A	35	-30.881	-4.365	6.864	1.00	15.00	A	C
ATOM	140	OE1	GLU	A	35	-31.215	-3.174	7.033	1.00	15.00	A	O
ATOM	141	OE2	GLU	A	35	-31.428	-5.093	6.008	1.00	15.00	A	O
ATOM	142	C	GLU	A	35	-26.354	-3.297	7.289	1.00	15.00	A	C
ATOM	143	O	GLU	A	35	-25.843	-3.217	8.410	1.00	15.00	A	O
ATOM	144	N	ALA	A	36	-25.632	-3.341	6.175	1.00	15.00	A	N
ATOM	145	CA	ALA	A	36	-24.173	-3.312	6.210	1.00	15.00	A	C
ATOM	146	CB	ALA	A	36	-23.594	-3.581	4.830	1.00	15.00	A	C
ATOM	147	C	ALA	A	36	-23.652	-1.989	6.761	1.00	15.00	A	C
ATOM	148	O	ALA	A	36	-22.623	-1.956	7.435	1.00	15.00	A	O
ATOM	149	N	GLU	A	37	-24.378	-0.909	6.480	1.00	15.00	A	N
ATOM	150	CA	GLU	A	37	-23.997	0.425	6.938	1.00	15.00	A	C
ATOM	151	CB	GLU	A	37	-25.022	1.462	6.465	1.00	15.00	A	C
ATOM	152	CG	GLU	A	37	-24.592	2.911	6.647	1.00	15.00	A	C
ATOM	153	CD	GLU	A	37	-23.658	3.390	5.552	1.00	15.00	A	C
ATOM	154	OE1	GLU	A	37	-22.538	3.846	5.868	1.00	15.00	A	O
ATOM	155	OE2	GLU	A	37	-24.039	3.313	4.369	1.00	15.00	A	O
ATOM	156	C	GLU	A	37	-23.868	0.472	8.461	1.00	15.00	A	C
ATOM	157	O	GLU	A	37	-22.967	1.115	8.999	1.00	15.00	A	O
ATOM	158	N	ASP	A	38	-24.765	-0.219	9.155	1.00	15.00	A	N
ATOM	159	CA	ASP	A	38	-24.734	-0.238	10.612	1.00	15.00	A	C
ATOM	160	CB	ASP	A	38	-26.109	-0.550	11.200	1.00	15.00	A	C
ATOM	161	CG	ASP	A	38	-26.149	-0.326	12.700	1.00	15.00	A	C
ATOM	162	OD1	ASP	A	38	-26.287	0.843	13.121	1.00	15.00	A	O
ATOM	163	OD2	ASP	A	38	-26.027	-1.307	13.458	1.00	15.00	A	O
ATOM	164	C	ASP	A	38	-23.714	-1.238	11.132	1.00	15.00	A	C
ATOM	165	O	ASP	A	38	-22.823	-0.882	11.900	1.00	15.00	A	O
ATOM	166	N	LEU	A	39	-23.838	-2.482	10.680	1.00	15.00	A	N
ATOM	167	CA	LEU	A	39	-22.948	-3.560	11.105	1.00	15.00	A	C
ATOM	168	CB	LEU	A	39	-23.294	-4.860	10.379	1.00	15.00	A	C
ATOM	169	CG	LEU	A	39	-24.537	-5.590	10.890	1.00	15.00	A	C
ATOM	170	CD1	LEU	A	39	-25.140	-6.452	9.796	1.00	15.00	A	C
ATOM	171	CD2	LEU	A	39	-24.196	-6.433	12.109	1.00	15.00	A	C
ATOM	172	C	LEU	A	39	-21.470	-3.213	10.934	1.00	15.00	A	C
ATOM	173	O	LEU	A	39	-20.652	-3.507	11.810	1.00	15.00	A	O
ATOM	174	N	PHE	A	40	-21.128	-2.583	9.815	1.00	15.00	A	N
ATOM	175	CA	PHE	A	40	-19.747	-2.197	9.560	1.00	15.00	A	C
ATOM	176	CB	PHE	A	40	-19.573	-1.710	8.119	1.00	15.00	A	C
ATOM	177	CG	PHE	A	40	-18.156	-1.365	7.755	1.00	15.00	A	C
ATOM	178	CD1	PHE	A	40	-17.197	-2.356	7.626	1.00	15.00	A	C
ATOM	179	CD2	PHE	A	40	-17.785	-0.048	7.538	1.00	15.00	A	C
ATOM	180	CE1	PHE	A	40	-15.894	-2.041	7.289	1.00	15.00	A	C
ATOM	181	CE2	PHE	A	40	-16.485	0.275	7.201	1.00	15.00	A	C
ATOM	182	CZ	PHE	A	40	-15.538	-0.723	7.077	1.00	15.00	A	C
ATOM	183	C	PHE	A	40	-19.315	-1.116	10.545	1.00	15.00	A	C
ATOM	184	O	PHE	A	40	-18.192	-1.138	11.052	1.00	15.00	A	O
ATOM	185	N	TYR	A	41	-20.223	-0.188	10.826	1.00	15.00	A	N
ATOM	186	CA	TYR	A	41	-19.949	0.899	11.757	1.00	15.00	A	C
ATOM	187	CB	TYR	A	41	-21.129	1.878	11.811	1.00	15.00	A	C
ATOM	188	CG	TYR	A	41	-20.908	3.055	12.738	1.00	15.00	A	C
ATOM	189	CD1	TYR	A	41	-19.847	3.925	12.541	1.00	15.00	A	C
ATOM	190	CD2	TYR	A	41	-21.758	3.295	13.811	1.00	15.00	A	C
ATOM	191	CE1	TYR	A	41	-19.632	4.998	13.380	1.00	15.00	A	C
ATOM	192	CE2	TYR	A	41	-21.551	4.370	14.658	1.00	15.00	A	C
ATOM	193	CZ	TYR	A	41	-20.484	5.217	14.437	1.00	15.00	A	C
ATOM	194	OH	TYR	A	41	-20.271	6.289	15.273	1.00	15.00	A	O
ATOM	195	C	TYR	A	41	-19.661	0.351	13.148	1.00	15.00	A	C
ATOM	196	O	TYR	A	41	-18.685	0.740	13.785	1.00	15.00	A	O
ATOM	197	N	GLN	A	42	-20.506	-0.569	13.601	1.00	15.00	A	N
ATOM	198	CA	GLN	A	42	-20.353	-1.179	14.920	1.00	15.00	A	C
ATOM	199	CB	GLN	A	42	-21.488	-2.174	15.185	1.00	15.00	A	C
ATOM	200	CG	GLN	A	42	-22.865	-1.661	14.797	1.00	15.00	A	C
ATOM	201	CD	GLN	A	42	-23.442	-0.671	15.790	1.00	15.00	A	C
ATOM	202	OE1	GLN	A	42	-22.733	-0.133	16.641	1.00	15.00	A	O
ATOM	203	NE2	GLN	A	42	-24.730	-0.398	15.664	1.00	15.00	A	N
ATOM	204	C	GLN	A	42	-19.005	-1.882	15.041	1.00	15.00	A	C
ATOM	205	O	GLN	A	42	-18.277	-1.692	16.018	1.00	15.00	A	O
ATOM	206	N	SER	A	43	-18.673	-2.682	14.034	1.00	15.00	A	N

ATOM	207	CA	SER	A	43	-17.415	-3.415	14.019	1.00	15.00	A	C
ATOM	208	CB	SER	A	43	-17.349	-4.342	12.800	1.00	15.00	A	C
ATOM	209	OG	SER	A	43	-16.274	-5.259	12.908	1.00	15.00	A	O
ATOM	210	C	SER	A	43	-16.226	-2.456	14.029	1.00	15.00	A	C
ATOM	211	O	SER	A	43	-15.260	-2.655	14.770	1.00	15.00	A	O
ATOM	212	N	SER	A	44	-16.307	-1.412	13.215	1.00	15.00	A	N
ATOM	213	CA	SER	A	44	-15.241	-0.424	13.133	1.00	15.00	A	C
ATOM	214	CB	SER	A	44	-15.458	0.490	11.929	1.00	15.00	A	C
ATOM	215	OG	SER	A	44	-15.643	-0.276	10.751	1.00	15.00	A	O
ATOM	216	C	SER	A	44	-15.138	0.386	14.423	1.00	15.00	A	C
ATOM	217	O	SER	A	44	-14.039	0.690	14.886	1.00	15.00	A	O
ATOM	218	N	LEU	A	45	-16.287	0.719	15.006	1.00	15.00	A	N
ATOM	219	CA	LEU	A	45	-16.329	1.481	16.248	1.00	15.00	A	C
ATOM	220	CB	LEU	A	45	-17.776	1.786	16.642	1.00	15.00	A	C
ATOM	221	CG	LEU	A	45	-17.970	2.703	17.852	1.00	15.00	A	C
ATOM	222	CD1	LEU	A	45	-17.508	4.118	17.536	1.00	15.00	A	C
ATOM	223	CD2	LEU	A	45	-19.424	2.698	18.298	1.00	15.00	A	C
ATOM	224	C	LEU	A	45	-15.632	0.714	17.362	1.00	15.00	A	C
ATOM	225	O	LEU	A	45	-14.819	1.271	18.100	1.00	15.00	A	O
ATOM	226	N	ALA	A	46	-15.951	-0.572	17.470	1.00	15.00	A	N
ATOM	227	CA	ALA	A	46	-15.342	-1.426	18.481	1.00	15.00	A	C
ATOM	228	CB	ALA	A	46	-15.967	-2.811	18.453	1.00	15.00	A	C
ATOM	229	C	ALA	A	46	-13.838	-1.512	18.250	1.00	15.00	A	C
ATOM	230	O	ALA	A	46	-13.048	-1.493	19.195	1.00	15.00	A	O
ATOM	231	N	SER	A	47	-13.452	-1.593	16.980	1.00	15.00	A	N
ATOM	232	CA	SER	A	47	-12.050	-1.660	16.608	1.00	15.00	A	C
ATOM	233	CB	SER	A	47	-11.909	-1.901	15.101	1.00	15.00	A	C
ATOM	234	OG	SER	A	47	-10.567	-2.189	14.744	1.00	15.00	A	O
ATOM	235	C	SER	A	47	-11.337	-0.373	17.019	1.00	15.00	A	C
ATOM	236	O	SER	A	47	-10.228	-0.414	17.551	1.00	15.00	A	O
ATOM	237	N	TRP	A	48	-11.986	0.766	16.779	1.00	15.00	A	N
ATOM	238	CA	TRP	A	48	-11.419	2.060	17.139	1.00	15.00	A	C
ATOM	239	CB	TRP	A	48	-12.322	3.212	16.670	1.00	15.00	A	C
ATOM	240	CG	TRP	A	48	-11.871	4.563	17.152	1.00	15.00	A	C
ATOM	241	CD1	TRP	A	48	-12.137	5.134	18.366	1.00	15.00	A	C
ATOM	242	CD2	TRP	A	48	-11.072	5.512	16.435	1.00	15.00	A	C
ATOM	243	NE1	TRP	A	48	-11.545	6.369	18.452	1.00	15.00	A	N
ATOM	244	CE2	TRP	A	48	-10.889	6.627	17.278	1.00	15.00	A	C
ATOM	245	CE3	TRP	A	48	-10.494	5.528	15.164	1.00	15.00	A	C
ATOM	246	CZ2	TRP	A	48	-10.148	7.739	16.892	1.00	15.00	A	C
ATOM	247	CZ3	TRP	A	48	-9.761	6.635	14.783	1.00	15.00	A	C
ATOM	248	CH2	TRP	A	48	-9.596	7.725	15.643	1.00	15.00	A	C
ATOM	249	C	TRP	A	48	-11.210	2.143	18.644	1.00	15.00	A	C
ATOM	250	O	TRP	A	48	-10.142	2.541	19.108	1.00	15.00	A	O
ATOM	251	N	ASN	A	49	-12.237	1.754	19.396	1.00	15.00	A	N
ATOM	252	CA	ASN	A	49	-12.181	1.783	20.854	1.00	15.00	A	C
ATOM	253	CB	ASN	A	49	-13.446	1.177	21.471	1.00	15.00	A	C
ATOM	254	CG	ASN	A	49	-14.636	2.118	21.421	1.00	15.00	A	C
ATOM	255	OD1	ASN	A	49	-14.481	3.337	21.357	1.00	15.00	A	O
ATOM	256	ND2	ASN	A	49	-15.837	1.559	21.460	1.00	15.00	A	N
ATOM	257	C	ASN	A	49	-10.944	1.061	21.365	1.00	15.00	A	C
ATOM	258	O	ASN	A	49	-10.174	1.614	22.149	1.00	15.00	A	O
ATOM	259	N	TYR	A	50	-10.745	-0.164	20.894	1.00	15.00	A	N
ATOM	260	CA	TYR	A	50	-9.592	-0.958	21.294	1.00	15.00	A	C
ATOM	261	CB	TYR	A	50	-9.721	-2.397	20.778	1.00	15.00	A	C
ATOM	262	CG	TYR	A	50	-8.420	-3.171	20.798	1.00	15.00	A	C
ATOM	263	CD1	TYR	A	50	-7.838	-3.560	21.996	1.00	15.00	A	C
ATOM	264	CD2	TYR	A	50	-7.768	-3.500	19.616	1.00	15.00	A	C
ATOM	265	CE1	TYR	A	50	-6.644	-4.254	22.015	1.00	15.00	A	C
ATOM	266	CE2	TYR	A	50	-6.576	-4.196	19.625	1.00	15.00	A	C
ATOM	267	CZ	TYR	A	50	-6.018	-4.570	20.828	1.00	15.00	A	C
ATOM	268	OH	TYR	A	50	-4.828	-5.260	20.845	1.00	15.00	A	O
ATOM	269	C	TYR	A	50	-8.292	-0.335	20.790	1.00	15.00	A	C
ATOM	270	O	TYR	A	50	-7.284	-0.322	21.492	1.00	15.00	A	O
ATOM	271	N	ASN	A	51	-8.327	0.195	19.576	1.00	15.00	A	N
ATOM	272	CA	ASN	A	51	-7.148	0.802	18.970	1.00	15.00	A	C
ATOM	273	CB	ASN	A	51	-7.383	1.116	17.492	1.00	15.00	A	C
ATOM	274	CG	ASN	A	51	-6.921	-0.004	16.579	1.00	15.00	A	C
ATOM	275	OD1	ASN	A	51	-5.776	-0.029	16.138	1.00	15.00	A	O
ATOM	276	ND2	ASN	A	51	-7.812	-0.939	16.288	1.00	15.00	A	N
ATOM	277	C	ASN	A	51	-6.681	2.046	19.720	1.00	15.00	A	C

ATOM	278	O	ASN	A	51	-5.490	2.355	19.733	1.00	15.00	A	O
ATOM	279	N	THR	A	52	-7.613	2.756	20.343	1.00	15.00	A	N
ATOM	280	CA	THR	A	52	-7.271	3.960	21.094	1.00	15.00	A	C
ATOM	281	CB	THR	A	52	-8.278	5.101	20.844	1.00	15.00	A	C
ATOM	282	OG1	THR	A	52	-9.620	4.631	21.037	1.00	15.00	A	O
ATOM	283	CG2	THR	A	52	-8.132	5.638	19.430	1.00	15.00	A	C
ATOM	284	C	THR	A	52	-7.169	3.681	22.593	1.00	15.00	A	C
ATOM	285	O	THR	A	52	-6.807	4.559	23.378	1.00	15.00	A	O
ATOM	286	N	ASN	A	53	-7.486	2.455	22.982	1.00	15.00	A	N
ATOM	287	CA	ASN	A	53	-7.440	2.053	24.382	1.00	15.00	A	C
ATOM	288	CB	ASN	A	53	-8.686	2.548	25.126	1.00	15.00	A	C
ATOM	289	CG	ASN	A	53	-8.651	2.264	26.616	1.00	15.00	A	C
ATOM	290	OD1	ASN	A	53	-7.593	2.042	27.198	1.00	15.00	A	O
ATOM	291	ND2	ASN	A	53	-9.815	2.289	27.248	1.00	15.00	A	N
ATOM	292	C	ASN	A	53	-7.315	0.536	24.483	1.00	15.00	A	C
ATOM	293	O	ASN	A	53	-8.317	-0.183	24.529	1.00	15.00	A	O
ATOM	294	N	ILE	A	54	-6.079	0.063	24.490	1.00	15.00	A	N
ATOM	295	CA	ILE	A	54	-5.788	-1.362	24.559	1.00	15.00	A	C
ATOM	296	CB	ILE	A	54	-4.319	-1.664	24.188	1.00	15.00	A	C
ATOM	297	CG1	ILE	A	54	-3.990	-1.075	22.812	1.00	15.00	A	C
ATOM	298	CG2	ILE	A	54	-4.055	-3.165	24.204	1.00	15.00	A	C
ATOM	299	CD1	ILE	A	54	-2.521	-1.140	22.449	1.00	15.00	A	C
ATOM	300	C	ILE	A	54	-6.107	-1.950	25.933	1.00	15.00	A	C
ATOM	301	O	ILE	A	54	-5.312	-1.855	26.868	1.00	15.00	A	O
ATOM	302	N	THR	A	55	-7.286	-2.540	26.045	1.00	15.00	A	N
ATOM	303	CA	THR	A	55	-7.724	-3.176	27.276	1.00	15.00	A	C
ATOM	304	CB	THR	A	55	-8.752	-2.305	28.031	1.00	15.00	A	C
ATOM	305	OG1	THR	A	55	-9.803	-1.909	27.139	1.00	15.00	A	O
ATOM	306	CG2	THR	A	55	-8.093	-1.069	28.624	1.00	15.00	A	C
ATOM	307	C	THR	A	55	-8.362	-4.518	26.937	1.00	15.00	A	C
ATOM	308	O	THR	A	55	-8.924	-4.672	25.852	1.00	15.00	A	O
ATOM	309	N	GLU	A	56	-8.272	-5.480	27.854	1.00	15.00	A	N
ATOM	310	CA	GLU	A	56	-8.845	-6.811	27.640	1.00	15.00	A	C
ATOM	311	CB	GLU	A	56	-8.677	-7.670	28.897	1.00	15.00	A	C
ATOM	312	CG	GLU	A	56	-8.989	-9.143	28.692	1.00	15.00	A	C
ATOM	313	CD	GLU	A	56	-8.193	-9.747	27.553	1.00	15.00	A	C
ATOM	314	OE1	GLU	A	56	-6.947	-9.741	27.633	1.00	15.00	A	O
ATOM	315	OE2	GLU	A	56	-8.814	-10.219	26.580	1.00	15.00	A	O
ATOM	316	C	GLU	A	56	-10.323	-6.717	27.260	1.00	15.00	A	C
ATOM	317	O	GLU	A	56	-10.795	-7.401	26.351	1.00	15.00	A	O
ATOM	318	N	GLU	A	57	-11.040	-5.839	27.951	1.00	15.00	A	N
ATOM	319	CA	GLU	A	57	-12.457	-5.631	27.693	1.00	15.00	A	C
ATOM	320	CB	GLU	A	57	-13.015	-4.597	28.671	1.00	15.00	A	C
ATOM	321	CG	GLU	A	57	-14.476	-4.246	28.452	1.00	15.00	A	C
ATOM	322	CD	GLU	A	57	-14.919	-3.084	29.313	1.00	15.00	A	C
ATOM	323	OE1	GLU	A	57	-16.025	-3.157	29.884	1.00	15.00	A	O
ATOM	324	OE2	GLU	A	57	-14.152	-2.102	29.419	1.00	15.00	A	O
ATOM	325	C	GLU	A	57	-12.679	-5.174	26.252	1.00	15.00	A	C
ATOM	326	O	GLU	A	57	-13.581	-5.658	25.566	1.00	15.00	A	O
ATOM	327	N	ASN	A	58	-11.832	-4.259	25.793	1.00	15.00	A	N
ATOM	328	CA	ASN	A	58	-11.934	-3.740	24.435	1.00	15.00	A	C
ATOM	329	CB	ASN	A	58	-11.053	-2.506	24.238	1.00	15.00	A	C
ATOM	330	CG	ASN	A	58	-11.835	-1.212	24.338	1.00	15.00	A	C
ATOM	331	OD1	ASN	A	58	-13.035	-1.173	24.074	1.00	15.00	A	O
ATOM	332	ND2	ASN	A	58	-11.155	-0.138	24.706	1.00	15.00	A	N
ATOM	333	C	ASN	A	58	-11.589	-4.809	23.412	1.00	15.00	A	C
ATOM	334	O	ASN	A	58	-12.186	-4.856	22.338	1.00	15.00	A	O
ATOM	335	N	VAL	A	59	-10.627	-5.667	23.750	1.00	15.00	A	N
ATOM	336	CA	VAL	A	59	-10.227	-6.754	22.862	1.00	15.00	A	C
ATOM	337	CB	VAL	A	59	-9.134	-7.649	23.496	1.00	15.00	A	C
ATOM	338	CG1	VAL	A	59	-8.746	-8.779	22.553	1.00	15.00	A	C
ATOM	339	CG2	VAL	A	59	-7.910	-6.830	23.871	1.00	15.00	A	C
ATOM	340	C	VAL	A	59	-11.444	-7.615	22.556	1.00	15.00	A	C
ATOM	341	O	VAL	A	59	-11.798	-7.826	21.396	1.00	15.00	A	O
ATOM	342	N	GLN	A	60	-12.098	-8.079	23.614	1.00	15.00	A	N
ATOM	343	CA	GLN	A	60	-13.286	-8.905	23.478	1.00	15.00	A	C
ATOM	344	CB	GLN	A	60	-13.757	-9.413	24.842	1.00	15.00	A	C
ATOM	345	CG	GLN	A	60	-13.231	-10.795	25.192	1.00	15.00	A	C
ATOM	346	CD	GLN	A	60	-13.759	-11.864	24.256	1.00	15.00	A	C
ATOM	347	OE1	GLN	A	60	-13.185	-12.120	23.198	1.00	15.00	A	O
ATOM	348	NE2	GLN	A	60	-14.855	-12.501	24.641	1.00	15.00	A	N

ATOM	349	C	GLN	A	60	-14.404	-8.151	22.769	1.00	15.00	A	C
ATOM	350	O	GLN	A	60	-15.121	-8.721	21.952	1.00	15.00	A	O
ATOM	351	N	ASN	A	61	-14.542	-6.865	23.077	1.00	15.00	A	N
ATOM	352	CA	ASN	A	61	-15.570	-6.035	22.451	1.00	15.00	A	C
ATOM	353	CB	ASN	A	61	-15.589	-4.628	23.054	1.00	15.00	A	C
ATOM	354	CG	ASN	A	61	-16.666	-4.458	24.108	1.00	15.00	A	C
ATOM	355	OD1	ASN	A	61	-17.856	-4.445	23.799	1.00	15.00	A	O
ATOM	356	ND2	ASN	A	61	-16.258	-4.319	25.360	1.00	15.00	A	N
ATOM	357	C	ASN	A	61	-15.352	-5.959	20.945	1.00	15.00	A	C
ATOM	358	O	ASN	A	61	-16.296	-6.065	20.160	1.00	15.00	A	O
ATOM	359	N	MET	A	62	-14.096	-5.788	20.551	1.00	15.00	A	N
ATOM	360	CA	MET	A	62	-13.732	-5.712	19.143	1.00	15.00	A	C
ATOM	361	CB	MET	A	62	-12.262	-5.304	19.001	1.00	15.00	A	C
ATOM	362	CG	MET	A	62	-11.727	-5.352	17.579	1.00	15.00	A	C
ATOM	363	SD	MET	A	62	-10.009	-4.817	17.472	1.00	15.00	A	S
ATOM	364	CE	MET	A	62	-9.622	-5.253	15.780	1.00	15.00	A	C
ATOM	365	C	MET	A	62	-13.980	-7.053	18.461	1.00	15.00	A	C
ATOM	366	O	MET	A	62	-14.509	-7.107	17.349	1.00	15.00	A	O
ATOM	367	N	ASN	A	63	-13.610	-8.131	19.146	1.00	15.00	A	N
ATOM	368	CA	ASN	A	63	-13.795	-9.481	18.619	1.00	15.00	A	C
ATOM	369	CB	ASN	A	63	-13.161	-10.528	19.545	1.00	15.00	A	C
ATOM	370	CG	ASN	A	63	-11.649	-10.418	19.639	1.00	15.00	A	C
ATOM	371	OD1	ASN	A	63	-10.992	-9.898	18.738	1.00	15.00	A	O
ATOM	372	ND2	ASN	A	63	-11.084	-10.918	20.730	1.00	15.00	A	N
ATOM	373	C	ASN	A	63	-15.277	-9.785	18.437	1.00	15.00	A	C
ATOM	374	O	ASN	A	63	-15.697	-10.276	17.392	1.00	15.00	A	O
ATOM	375	N	ASN	A	64	-16.066	-9.450	19.456	1.00	15.00	A	N
ATOM	376	CA	ASN	A	64	-17.510	-9.683	19.438	1.00	15.00	A	C
ATOM	377	CB	ASN	A	64	-18.143	-9.235	20.760	1.00	15.00	A	C
ATOM	378	CG	ASN	A	64	-17.979	-10.251	21.875	1.00	15.00	A	C
ATOM	379	OD1	ASN	A	64	-18.016	-11.457	21.647	1.00	15.00	A	O
ATOM	380	ND2	ASN	A	64	-17.797	-9.767	23.095	1.00	15.00	A	N
ATOM	381	C	ASN	A	64	-18.186	-8.965	18.275	1.00	15.00	A	C
ATOM	382	O	ASN	A	64	-19.200	-9.427	17.749	1.00	15.00	A	O
ATOM	383	N	ALA	A	65	-17.634	-7.824	17.886	1.00	15.00	A	N
ATOM	384	CA	ALA	A	65	-18.184	-7.054	16.781	1.00	15.00	A	C
ATOM	385	CB	ALA	A	65	-17.810	-5.587	16.917	1.00	15.00	A	C
ATOM	386	C	ALA	A	65	-17.702	-7.610	15.446	1.00	15.00	A	C
ATOM	387	O	ALA	A	65	-18.485	-7.760	14.505	1.00	15.00	A	O
ATOM	388	N	GLY	A	66	-16.413	-7.933	15.385	1.00	15.00	A	N
ATOM	389	CA	GLY	A	66	-15.828	-8.467	14.169	1.00	15.00	A	C
ATOM	390	C	GLY	A	66	-16.413	-9.808	13.772	1.00	15.00	A	C
ATOM	391	O	GLY	A	66	-16.653	-10.059	12.589	1.00	15.00	A	O
ATOM	392	N	ASP	A	67	-16.643	-10.669	14.757	1.00	15.00	A	N
ATOM	393	CA	ASP	A	67	-17.208	-11.993	14.503	1.00	15.00	A	C
ATOM	394	CB	ASP	A	67	-17.283	-12.816	15.792	1.00	15.00	A	C
ATOM	395	CG	ASP	A	67	-16.214	-13.891	15.862	1.00	15.00	A	C
ATOM	396	OD1	ASP	A	67	-15.536	-14.122	14.838	1.00	15.00	A	O
ATOM	397	OD2	ASP	A	67	-16.057	-14.504	16.938	1.00	15.00	A	O
ATOM	398	C	ASP	A	67	-18.586	-11.886	13.863	1.00	15.00	A	C
ATOM	399	O	ASP	A	67	-18.905	-12.610	12.918	1.00	15.00	A	O
ATOM	400	N	LYS	A	68	-19.394	-10.958	14.366	1.00	15.00	A	N
ATOM	401	CA	LYS	A	68	-20.736	-10.745	13.836	1.00	15.00	A	C
ATOM	402	CB	LYS	A	68	-21.502	-9.729	14.685	1.00	15.00	A	C
ATOM	403	CG	LYS	A	68	-21.883	-10.234	16.066	1.00	15.00	A	C
ATOM	404	CD	LYS	A	68	-22.708	-9.206	16.821	1.00	15.00	A	C
ATOM	405	CE	LYS	A	68	-23.036	-9.686	18.225	1.00	15.00	A	C
ATOM	406	NZ	LYS	A	68	-23.916	-8.728	18.949	1.00	15.00	A	N
ATOM	407	C	LYS	A	68	-20.670	-10.274	12.388	1.00	15.00	A	C
ATOM	408	O	LYS	A	68	-21.444	-10.723	11.542	1.00	15.00	A	O
ATOM	409	N	TRP	A	69	-19.733	-9.374	12.114	1.00	15.00	A	N
ATOM	410	CA	TRP	A	69	-19.545	-8.842	10.771	1.00	15.00	A	C
ATOM	411	CB	TRP	A	69	-18.519	-7.702	10.804	1.00	15.00	A	C
ATOM	412	CG	TRP	A	69	-18.161	-7.142	9.460	1.00	15.00	A	C
ATOM	413	CD1	TRP	A	69	-16.927	-7.153	8.877	1.00	15.00	A	C
ATOM	414	CD2	TRP	A	69	-19.036	-6.484	8.534	1.00	15.00	A	C
ATOM	415	NE1	TRP	A	69	-16.979	-6.544	7.648	1.00	15.00	A	N
ATOM	416	CE2	TRP	A	69	-18.262	-6.125	7.413	1.00	15.00	A	C
ATOM	417	CE3	TRP	A	69	-20.397	-6.164	8.543	1.00	15.00	A	C
ATOM	418	CZ2	TRP	A	69	-18.803	-5.464	6.313	1.00	15.00	A	C
ATOM	419	CZ3	TRP	A	69	-20.932	-5.508	7.450	1.00	15.00	A	C

ATOM	420	CH2	TRP	A	69	-20.137	-5.166	6.351	1.00	15.00	A	C
ATOM	421	C	TRP	A	69	-19.096	-9.951	9.822	1.00	15.00	A	C
ATOM	422	O	TRP	A	69	-19.604	-10.074	8.705	1.00	15.00	A	O
ATOM	423	N	SER	A	70	-18.161	-10.771	10.290	1.00	15.00	A	N
ATOM	424	CA	SER	A	70	-17.643	-11.880	9.502	1.00	15.00	A	C
ATOM	425	CB	SER	A	70	-16.482	-12.552	10.238	1.00	15.00	A	C
ATOM	426	OG	SER	A	70	-15.488	-11.600	10.580	1.00	15.00	A	O
ATOM	427	C	SER	A	70	-18.747	-12.894	9.195	1.00	15.00	A	C
ATOM	428	O	SER	A	70	-18.828	-13.425	8.082	1.00	15.00	A	O
ATOM	429	N	ALA	A	71	-19.596	-13.156	10.185	1.00	15.00	A	N
ATOM	430	CA	ALA	A	71	-20.702	-14.087	10.014	1.00	15.00	A	C
ATOM	431	CB	ALA	A	71	-21.363	-14.386	11.352	1.00	15.00	A	C
ATOM	432	C	ALA	A	71	-21.718	-13.523	9.027	1.00	15.00	A	C
ATOM	433	O	ALA	A	71	-22.218	-14.239	8.159	1.00	15.00	A	O
ATOM	434	N	PHE	A	72	-21.999	-12.229	9.160	1.00	15.00	A	N
ATOM	435	CA	PHE	A	72	-22.943	-11.548	8.281	1.00	15.00	A	C
ATOM	436	CB	PHE	A	72	-23.091	-10.078	8.693	1.00	15.00	A	C
ATOM	437	CG	PHE	A	72	-23.979	-9.265	7.790	1.00	15.00	A	C
ATOM	438	CD1	PHE	A	72	-25.339	-9.523	7.713	1.00	15.00	A	C
ATOM	439	CD2	PHE	A	72	-23.453	-8.237	7.024	1.00	15.00	A	C
ATOM	440	CE1	PHE	A	72	-26.156	-8.772	6.889	1.00	15.00	A	C
ATOM	441	CE2	PHE	A	72	-24.264	-7.483	6.197	1.00	15.00	A	C
ATOM	442	CZ	PHE	A	72	-25.618	-7.751	6.129	1.00	15.00	A	C
ATOM	443	C	PHE	A	72	-22.495	-11.648	6.828	1.00	15.00	A	C
ATOM	444	O	PHE	A	72	-23.287	-11.980	5.945	1.00	15.00	A	O
ATOM	445	N	LEU	A	73	-21.216	-11.375	6.589	1.00	15.00	A	N
ATOM	446	CA	LEU	A	73	-20.658	-11.440	5.246	1.00	15.00	A	C
ATOM	447	CB	LEU	A	73	-19.202	-10.973	5.241	1.00	15.00	A	C
ATOM	448	CG	LEU	A	73	-18.962	-9.486	5.502	1.00	15.00	A	C
ATOM	449	CD1	LEU	A	73	-17.472	-9.190	5.525	1.00	15.00	A	C
ATOM	450	CD2	LEU	A	73	-19.663	-8.637	4.451	1.00	15.00	A	C
ATOM	451	C	LEU	A	73	-20.746	-12.855	4.692	1.00	15.00	A	C
ATOM	452	O	LEU	A	73	-21.032	-13.053	3.513	1.00	15.00	A	O
ATOM	453	N	LYS	A	74	-20.507	-13.836	5.555	1.00	15.00	A	N
ATOM	454	CA	LYS	A	74	-20.560	-15.233	5.155	1.00	15.00	A	C
ATOM	455	CB	LYS	A	74	-20.122	-16.144	6.305	1.00	15.00	A	C
ATOM	456	CG	LYS	A	74	-19.933	-17.600	5.908	1.00	15.00	A	C
ATOM	457	CD	LYS	A	74	-19.057	-18.342	6.907	1.00	15.00	A	C
ATOM	458	CE	LYS	A	74	-17.624	-17.828	6.882	1.00	15.00	A	C
ATOM	459	NZ	LYS	A	74	-17.022	-17.920	5.522	1.00	15.00	A	N
ATOM	460	C	LYS	A	74	-21.958	-15.606	4.672	1.00	15.00	A	C
ATOM	461	O	LYS	A	74	-22.113	-16.203	3.608	1.00	15.00	A	O
ATOM	462	N	GLU	A	75	-22.966	-15.233	5.450	1.00	15.00	A	N
ATOM	463	CA	GLU	A	75	-24.352	-15.517	5.100	1.00	15.00	A	C
ATOM	464	CB	GLU	A	75	-25.283	-15.094	6.243	1.00	15.00	A	C
ATOM	465	CG	GLU	A	75	-26.768	-15.269	5.955	1.00	15.00	A	C
ATOM	466	CD	GLU	A	75	-27.131	-16.676	5.515	1.00	15.00	A	C
ATOM	467	OE1	GLU	A	75	-26.369	-17.615	5.827	1.00	15.00	A	O
ATOM	468	OE2	GLU	A	75	-28.178	-16.837	4.857	1.00	15.00	A	O
ATOM	469	C	GLU	A	75	-24.734	-14.804	3.804	1.00	15.00	A	C
ATOM	470	O	GLU	A	75	-25.286	-15.410	2.884	1.00	15.00	A	O
ATOM	471	N	GLN	A	76	-24.405	-13.517	3.729	1.00	15.00	A	N
ATOM	472	CA	GLN	A	76	-24.710	-12.717	2.550	1.00	15.00	A	C
ATOM	473	CB	GLN	A	76	-24.283	-11.263	2.751	1.00	15.00	A	C
ATOM	474	CG	GLN	A	76	-25.101	-10.516	3.790	1.00	15.00	A	C
ATOM	475	CD	GLN	A	76	-26.585	-10.517	3.481	1.00	15.00	A	C
ATOM	476	OE1	GLN	A	76	-27.083	-9.650	2.770	1.00	15.00	A	O
ATOM	477	NE2	GLN	A	76	-27.302	-11.490	4.020	1.00	15.00	A	N
ATOM	478	C	GLN	A	76	-24.053	-13.291	1.301	1.00	15.00	A	C
ATOM	479	O	GLN	A	76	-24.641	-13.275	0.223	1.00	15.00	A	O
ATOM	480	N	SER	A	77	-22.838	-13.803	1.456	1.00	15.00	A	N
ATOM	481	CA	SER	A	77	-22.107	-14.388	0.343	1.00	15.00	A	C
ATOM	482	CB	SER	A	77	-20.715	-14.844	0.793	1.00	15.00	A	C
ATOM	483	OG	SER	A	77	-19.851	-15.031	-0.316	1.00	15.00	A	O
ATOM	484	C	SER	A	77	-22.883	-15.560	-0.253	1.00	15.00	A	C
ATOM	485	O	SER	A	77	-23.081	-15.630	-1.466	1.00	15.00	A	O
ATOM	486	N	THR	A	78	-23.343	-16.458	0.611	1.00	15.00	A	N
ATOM	487	CA	THR	A	78	-24.099	-17.626	0.179	1.00	15.00	A	C
ATOM	488	CB	THR	A	78	-24.445	-18.527	1.377	1.00	15.00	A	C
ATOM	489	OG1	THR	A	78	-23.390	-18.445	2.346	1.00	15.00	A	O
ATOM	490	CG2	THR	A	78	-24.599	-19.973	0.929	1.00	15.00	A	C

ATOM	491	C	THR	A	78	-25.385	-17.213	-0.536	1.00	15.00	A	C
ATOM	492	O	THR	A	78	-25.716	-17.746	-1.597	1.00	15.00	A	O
ATOM	493	N	LEU	A	79	-26.094	-16.249	0.044	1.00	15.00	A	N
ATOM	494	CA	LEU	A	79	-27.339	-15.757	-0.536	1.00	15.00	A	C
ATOM	495	CB	LEU	A	79	-28.037	-14.797	0.430	1.00	15.00	A	C
ATOM	496	CG	LEU	A	79	-28.471	-15.379	1.777	1.00	15.00	A	C
ATOM	497	CD1	LEU	A	79	-28.975	-14.276	2.693	1.00	15.00	A	C
ATOM	498	CD2	LEU	A	79	-29.540	-16.444	1.586	1.00	15.00	A	C
ATOM	499	C	LEU	A	79	-27.084	-15.058	-1.868	1.00	15.00	A	C
ATOM	500	O	LEU	A	79	-27.896	-15.136	-2.790	1.00	15.00	A	O
ATOM	501	N	ALA	A	80	-25.950	-14.375	-1.964	1.00	15.00	A	N
ATOM	502	CA	ALA	A	80	-25.583	-13.664	-3.182	1.00	15.00	A	C
ATOM	503	CB	ALA	A	80	-24.378	-12.769	-2.937	1.00	15.00	A	C
ATOM	504	C	ALA	A	80	-25.296	-14.637	-4.317	1.00	15.00	A	C
ATOM	505	O	ALA	A	80	-25.648	-14.381	-5.466	1.00	15.00	A	O
ATOM	506	N	GLN	A	81	-24.679	-15.766	-3.981	1.00	15.00	A	N
ATOM	507	CA	GLN	A	81	-24.330	-16.785	-4.971	1.00	15.00	A	C
ATOM	508	CB	GLN	A	81	-23.364	-17.812	-4.375	1.00	15.00	A	C
ATOM	509	CG	GLN	A	81	-22.032	-17.219	-3.939	1.00	15.00	A	C
ATOM	510	CD	GLN	A	81	-21.212	-18.162	-3.079	1.00	15.00	A	C
ATOM	511	OE1	GLN	A	81	-21.214	-19.375	-3.281	1.00	15.00	A	O
ATOM	512	NE2	GLN	A	81	-20.507	-17.609	-2.104	1.00	15.00	A	N
ATOM	513	C	GLN	A	81	-25.566	-17.475	-5.551	1.00	15.00	A	C
ATOM	514	O	GLN	A	81	-25.457	-18.312	-6.444	1.00	15.00	A	O
ATOM	515	N	MET	A	82	-26.741	-17.116	-5.043	1.00	15.00	A	N
ATOM	516	CA	MET	A	82	-27.990	-17.694	-5.522	1.00	15.00	A	C
ATOM	517	CB	MET	A	82	-29.060	-17.665	-4.427	1.00	15.00	A	C
ATOM	518	CG	MET	A	82	-28.738	-18.528	-3.218	1.00	15.00	A	C
ATOM	519	SD	MET	A	82	-30.025	-18.471	-1.955	1.00	15.00	A	S
ATOM	520	CE	MET	A	82	-29.291	-19.496	-0.682	1.00	15.00	A	C
ATOM	521	C	MET	A	82	-28.486	-16.953	-6.763	1.00	15.00	A	C
ATOM	522	O	MET	A	82	-29.437	-17.381	-7.416	1.00	15.00	A	O
ATOM	523	N	TYR	A	83	-27.836	-15.840	-7.079	1.00	15.00	A	N
ATOM	524	CA	TYR	A	83	-28.204	-15.038	-8.239	1.00	15.00	A	C
ATOM	525	CB	TYR	A	83	-28.453	-13.582	-7.827	1.00	15.00	A	C
ATOM	526	CG	TYR	A	83	-29.589	-13.399	-6.840	1.00	15.00	A	C
ATOM	527	CD1	TYR	A	83	-29.338	-13.133	-5.499	1.00	15.00	A	C
ATOM	528	CD2	TYR	A	83	-30.914	-13.489	-7.250	1.00	15.00	A	C
ATOM	529	CE1	TYR	A	83	-30.372	-12.962	-4.597	1.00	15.00	A	C
ATOM	530	CE2	TYR	A	83	-31.954	-13.320	-6.355	1.00	15.00	A	C
ATOM	531	CZ	TYR	A	83	-31.679	-13.058	-5.030	1.00	15.00	A	C
ATOM	532	OH	TYR	A	83	-32.715	-12.882	-4.133	1.00	15.00	A	O
ATOM	533	C	TYR	A	83	-27.111	-15.109	-9.308	1.00	15.00	A	C
ATOM	534	O	TYR	A	83	-26.040	-14.526	-9.147	1.00	15.00	A	O
ATOM	535	N	PRO	A	84	-27.364	-15.836	-10.409	1.00	15.00	A	N
ATOM	536	CA	PRO	A	84	-26.393	-15.991	-11.502	1.00	15.00	A	C
ATOM	537	CB	PRO	A	84	-27.102	-16.925	-12.491	1.00	15.00	A	C
ATOM	538	CG	PRO	A	84	-28.550	-16.806	-12.160	1.00	15.00	A	C
ATOM	539	CD	PRO	A	84	-28.616	-16.562	-10.680	1.00	15.00	A	C
ATOM	540	C	PRO	A	84	-26.047	-14.663	-12.178	1.00	15.00	A	C
ATOM	541	O	PRO	A	84	-26.930	-13.918	-12.603	1.00	15.00	A	O
ATOM	542	N	LEU	A	85	-24.749	-14.389	-12.302	1.00	15.00	A	N
ATOM	543	CA	LEU	A	85	-24.270	-13.151	-12.918	1.00	15.00	A	C
ATOM	544	CB	LEU	A	85	-22.783	-12.937	-12.626	1.00	15.00	A	C
ATOM	545	CG	LEU	A	85	-22.425	-12.454	-11.222	1.00	15.00	A	C
ATOM	546	CD1	LEU	A	85	-20.928	-12.217	-11.115	1.00	15.00	A	C
ATOM	547	CD2	LEU	A	85	-23.193	-11.188	-10.873	1.00	15.00	A	C
ATOM	548	C	LEU	A	85	-24.506	-13.108	-14.425	1.00	15.00	A	C
ATOM	549	O	LEU	A	85	-24.177	-12.117	-15.082	1.00	15.00	A	O
ATOM	550	N	GLN	A	86	-25.065	-14.173	-14.977	1.00	15.00	A	N
ATOM	551	CA	GLN	A	86	-25.332	-14.225	-16.406	1.00	15.00	A	C
ATOM	552	CB	GLN	A	86	-25.464	-15.669	-16.892	1.00	15.00	A	C
ATOM	553	CG	GLN	A	86	-25.579	-15.793	-18.403	1.00	15.00	A	C
ATOM	554	CD	GLN	A	86	-26.646	-16.779	-18.832	1.00	15.00	A	C
ATOM	555	OE1	GLN	A	86	-26.846	-17.814	-18.201	1.00	15.00	A	O
ATOM	556	NE2	GLN	A	86	-27.353	-16.451	-19.902	1.00	15.00	A	N
ATOM	557	C	GLN	A	86	-26.586	-13.427	-16.752	1.00	15.00	A	C
ATOM	558	O	GLN	A	86	-26.680	-12.840	-17.827	1.00	15.00	A	O
ATOM	559	N	GLU	A	87	-27.532	-13.385	-15.821	1.00	15.00	A	N
ATOM	560	CA	GLU	A	87	-28.784	-12.666	-16.030	1.00	15.00	A	C
ATOM	561	CB	GLU	A	87	-29.937	-13.401	-15.351	1.00	15.00	A	C

ATOM	562	CG	GLU	A	87	-30.252	-14.759	-15.950	1.00	15.00	A	C
ATOM	563	CD	GLU	A	87	-31.371	-15.460	-15.211	1.00	15.00	A	C
ATOM	564	OE1	GLU	A	87	-31.093	-16.474	-14.539	1.00	15.00	A	O
ATOM	565	OE2	GLU	A	87	-32.525	-14.982	-15.287	1.00	15.00	A	O
ATOM	566	C	GLU	A	87	-28.700	-11.240	-15.496	1.00	15.00	A	C
ATOM	567	O	GLU	A	87	-29.715	-10.556	-15.360	1.00	15.00	A	O
ATOM	568	N	ILE	A	88	-27.490	-10.792	-15.200	1.00	15.00	A	N
ATOM	569	CA	ILE	A	88	-27.287	-9.450	-14.675	1.00	15.00	A	C
ATOM	570	CB	ILE	A	88	-26.384	-9.448	-13.421	1.00	15.00	A	C
ATOM	571	CG1	ILE	A	88	-26.720	-10.619	-12.486	1.00	15.00	A	C
ATOM	572	CG2	ILE	A	88	-26.497	-8.122	-12.684	1.00	15.00	A	C
ATOM	573	CD1	ILE	A	88	-28.071	-10.514	-11.807	1.00	15.00	A	C
ATOM	574	C	ILE	A	88	-26.663	-8.548	-15.738	1.00	15.00	A	C
ATOM	575	O	ILE	A	88	-25.523	-8.768	-16.168	1.00	15.00	A	O
ATOM	576	N	GLN	A	89	-27.412	-7.538	-16.156	1.00	15.00	A	N
ATOM	577	CA	GLN	A	89	-26.941	-6.597	-17.162	1.00	15.00	A	C
ATOM	578	CB	GLN	A	89	-28.047	-6.283	-18.175	1.00	15.00	A	C
ATOM	579	CG	GLN	A	89	-28.030	-7.165	-19.416	1.00	15.00	A	C
ATOM	580	CD	GLN	A	89	-28.392	-8.612	-19.130	1.00	15.00	A	C
ATOM	581	OE1	GLN	A	89	-27.865	-9.529	-19.757	1.00	15.00	A	O
ATOM	582	NE2	GLN	A	89	-29.302	-8.827	-18.191	1.00	15.00	A	N
ATOM	583	C	GLN	A	89	-26.462	-5.315	-16.495	1.00	15.00	A	C
ATOM	584	O	GLN	A	89	-25.452	-4.735	-16.895	1.00	15.00	A	O
ATOM	585	N	ASN	A	90	-27.194	-4.885	-15.475	1.00	15.00	A	N
ATOM	586	CA	ASN	A	90	-26.854	-3.676	-14.733	1.00	15.00	A	C
ATOM	587	CB	ASN	A	90	-27.944	-3.351	-13.708	1.00	15.00	A	C
ATOM	588	CG	ASN	A	90	-27.773	-1.980	-13.084	1.00	15.00	A	C
ATOM	589	OD1	ASN	A	90	-27.067	-1.820	-12.090	1.00	15.00	A	O
ATOM	590	ND2	ASN	A	90	-28.424	-0.980	-13.659	1.00	15.00	A	N
ATOM	591	C	ASN	A	90	-25.500	-3.823	-14.042	1.00	15.00	A	C
ATOM	592	O	ASN	A	90	-25.354	-4.598	-13.095	1.00	15.00	A	O
ATOM	593	N	LEU	A	91	-24.517	-3.069	-14.525	1.00	15.00	A	N
ATOM	594	CA	LEU	A	91	-23.158	-3.108	-13.989	1.00	15.00	A	C
ATOM	595	CB	LEU	A	91	-22.250	-2.137	-14.748	1.00	15.00	A	C
ATOM	596	CG	LEU	A	91	-22.074	-2.406	-16.246	1.00	15.00	A	C
ATOM	597	CD1	LEU	A	91	-21.269	-1.293	-16.898	1.00	15.00	A	C
ATOM	598	CD2	LEU	A	91	-21.408	-3.756	-16.476	1.00	15.00	A	C
ATOM	599	C	LEU	A	91	-23.098	-2.827	-12.489	1.00	15.00	A	C
ATOM	600	O	LEU	A	91	-22.401	-3.525	-11.755	1.00	15.00	A	O
ATOM	601	N	THR	A	92	-23.836	-1.818	-12.036	1.00	15.00	A	N
ATOM	602	CA	THR	A	92	-23.851	-1.446	-10.623	1.00	15.00	A	C
ATOM	603	CB	THR	A	92	-24.752	-0.223	-10.385	1.00	15.00	A	C
ATOM	604	OG1	THR	A	92	-25.384	0.136	-11.622	1.00	15.00	A	O
ATOM	605	CG2	THR	A	92	-23.927	0.952	-9.884	1.00	15.00	A	C
ATOM	606	C	THR	A	92	-24.296	-2.606	-9.727	1.00	15.00	A	C
ATOM	607	O	THR	A	92	-23.888	-2.701	-8.568	1.00	15.00	A	O
ATOM	608	N	VAL	A	93	-25.135	-3.480	-10.269	1.00	15.00	A	N
ATOM	609	CA	VAL	A	93	-25.616	-4.640	-9.529	1.00	15.00	A	C
ATOM	610	CB	VAL	A	93	-27.006	-5.089	-10.038	1.00	15.00	A	C
ATOM	611	CG1	VAL	A	93	-27.465	-6.361	-9.340	1.00	15.00	A	C
ATOM	612	CG2	VAL	A	93	-28.030	-3.982	-9.845	1.00	15.00	A	C
ATOM	613	C	VAL	A	93	-24.624	-5.789	-9.689	1.00	15.00	A	C
ATOM	614	O	VAL	A	93	-24.246	-6.443	-8.719	1.00	15.00	A	O
ATOM	615	N	LYS	A	94	-24.192	-5.996	-10.927	1.00	15.00	A	N
ATOM	616	CA	LYS	A	94	-23.250	-7.055	-11.271	1.00	15.00	A	C
ATOM	617	CB	LYS	A	94	-22.939	-6.984	-12.768	1.00	15.00	A	C
ATOM	618	CG	LYS	A	94	-22.359	-8.256	-13.360	1.00	15.00	A	C
ATOM	619	CD	LYS	A	94	-22.278	-8.155	-14.874	1.00	15.00	A	C
ATOM	620	CE	LYS	A	94	-22.131	-9.523	-15.517	1.00	15.00	A	C
ATOM	621	NZ	LYS	A	94	-22.574	-9.514	-16.937	1.00	15.00	A	N
ATOM	622	C	LYS	A	94	-21.954	-6.951	-10.470	1.00	15.00	A	C
ATOM	623	O	LYS	A	94	-21.482	-7.939	-9.909	1.00	15.00	A	O
ATOM	624	N	LEU	A	95	-21.391	-5.750	-10.416	1.00	15.00	A	N
ATOM	625	CA	LEU	A	95	-20.141	-5.517	-9.699	1.00	15.00	A	C
ATOM	626	CB	LEU	A	95	-19.640	-4.093	-9.937	1.00	15.00	A	C
ATOM	627	CG	LEU	A	95	-19.254	-3.756	-11.379	1.00	15.00	A	C
ATOM	628	CD1	LEU	A	95	-18.984	-2.269	-11.528	1.00	15.00	A	C
ATOM	629	CD2	LEU	A	95	-18.045	-4.569	-11.817	1.00	15.00	A	C
ATOM	630	C	LEU	A	95	-20.279	-5.799	-8.208	1.00	15.00	A	C
ATOM	631	O	LEU	A	95	-19.425	-6.459	-7.614	1.00	15.00	A	O
ATOM	632	N	GLN	A	96	-21.360	-5.311	-7.611	1.00	15.00	A	N

ATOM	633	CA	GLN	A	96	-21.600	-5.519	-6.189	1.00	15.00	A	C
ATOM	634	CB	GLN	A	96	-22.782	-4.681	-5.701	1.00	15.00	A	C
ATOM	635	CG	GLN	A	96	-22.458	-3.210	-5.500	1.00	15.00	A	C
ATOM	636	CD	GLN	A	96	-23.638	-2.427	-4.964	1.00	15.00	A	C
ATOM	637	OE1	GLN	A	96	-23.806	-2.275	-3.752	1.00	15.00	A	O
ATOM	638	NE2	GLN	A	96	-24.465	-1.928	-5.865	1.00	15.00	A	N
ATOM	639	C	GLN	A	96	-21.828	-6.995	-5.887	1.00	15.00	A	C
ATOM	640	O	GLN	A	96	-21.280	-7.533	-4.926	1.00	15.00	A	O
ATOM	641	N	LEU	A	97	-22.630	-7.648	-6.721	1.00	15.00	A	N
ATOM	642	CA	LEU	A	97	-22.917	-9.065	-6.549	1.00	15.00	A	C
ATOM	643	CB	LEU	A	97	-23.904	-9.553	-7.612	1.00	15.00	A	C
ATOM	644	CG	LEU	A	97	-25.388	-9.477	-7.251	1.00	15.00	A	C
ATOM	645	CD1	LEU	A	97	-26.241	-9.876	-8.444	1.00	15.00	A	C
ATOM	646	CD2	LEU	A	97	-25.694	-10.369	-6.056	1.00	15.00	A	C
ATOM	647	C	LEU	A	97	-21.637	-9.886	-6.620	1.00	15.00	A	C
ATOM	648	O	LEU	A	97	-21.378	-10.716	-5.755	1.00	15.00	A	O
ATOM	649	N	GLN	A	98	-20.828	-9.621	-7.640	1.00	15.00	A	N
ATOM	650	CA	GLN	A	98	-19.573	-10.338	-7.840	1.00	15.00	A	C
ATOM	651	CB	GLN	A	98	-18.882	-9.849	-9.114	1.00	15.00	A	C
ATOM	652	CG	GLN	A	98	-17.859	-10.821	-9.681	1.00	15.00	A	C
ATOM	653	CD	GLN	A	98	-17.248	-10.338	-10.983	1.00	15.00	A	C
ATOM	654	OE1	GLN	A	98	-16.935	-11.131	-11.866	1.00	15.00	A	O
ATOM	655	NE2	GLN	A	98	-17.069	-9.031	-11.109	1.00	15.00	A	N
ATOM	656	C	GLN	A	98	-18.635	-10.181	-6.644	1.00	15.00	A	C
ATOM	657	O	GLN	A	98	-17.957	-11.129	-6.248	1.00	15.00	A	O
ATOM	658	N	ALA	A	99	-18.620	-8.989	-6.059	1.00	15.00	A	N
ATOM	659	CA	ALA	A	99	-17.759	-8.706	-4.915	1.00	15.00	A	C
ATOM	660	CB	ALA	A	99	-17.654	-7.206	-4.687	1.00	15.00	A	C
ATOM	661	C	ALA	A	99	-18.244	-9.409	-3.650	1.00	15.00	A	C
ATOM	662	O	ALA	A	99	-17.491	-9.570	-2.693	1.00	15.00	A	O
ATOM	663	N	LEU	A	100	-19.500	-9.828	-3.646	1.00	15.00	A	N
ATOM	664	CA	LEU	A	100	-20.064	-10.507	-2.488	1.00	15.00	A	C
ATOM	665	CB	LEU	A	100	-21.439	-9.923	-2.148	1.00	15.00	A	C
ATOM	666	CG	LEU	A	100	-22.030	-10.316	-0.791	1.00	15.00	A	C
ATOM	667	CD1	LEU	A	100	-21.084	-9.937	0.340	1.00	15.00	A	C
ATOM	668	CD2	LEU	A	100	-23.388	-9.663	-0.593	1.00	15.00	A	C
ATOM	669	C	LEU	A	100	-20.164	-12.012	-2.721	1.00	15.00	A	C
ATOM	670	O	LEU	A	100	-20.318	-12.789	-1.779	1.00	15.00	A	O
ATOM	671	N	GLN	A	101	-20.061	-12.424	-3.977	1.00	15.00	A	N
ATOM	672	CA	GLN	A	101	-20.157	-13.837	-4.326	1.00	15.00	A	C
ATOM	673	CB	GLN	A	101	-20.682	-14.016	-5.748	1.00	15.00	A	C
ATOM	674	CG	GLN	A	101	-22.150	-13.667	-5.904	1.00	15.00	A	C
ATOM	675	CD	GLN	A	101	-22.635	-13.792	-7.331	1.00	15.00	A	C
ATOM	676	OE1	GLN	A	101	-21.866	-13.651	-8.278	1.00	15.00	A	O
ATOM	677	NE2	GLN	A	101	-23.919	-14.049	-7.492	1.00	15.00	A	N
ATOM	678	C	GLN	A	101	-18.834	-14.569	-4.153	1.00	15.00	A	C
ATOM	679	O	GLN	A	101	-18.805	-15.798	-4.106	1.00	15.00	A	O
ATOM	680	N	GLN	A	102	-17.743	-13.821	-4.066	1.00	15.00	A	N
ATOM	681	CA	GLN	A	102	-16.426	-14.422	-3.897	1.00	15.00	A	C
ATOM	682	CB	GLN	A	102	-15.300	-13.412	-4.150	1.00	15.00	A	C
ATOM	683	CG	GLN	A	102	-15.470	-12.069	-3.454	1.00	15.00	A	C
ATOM	684	CD	GLN	A	102	-14.309	-11.126	-3.715	1.00	15.00	A	C
ATOM	685	OE1	GLN	A	102	-14.474	-9.909	-3.757	1.00	15.00	A	O
ATOM	686	NE2	GLN	A	102	-13.119	-11.683	-3.893	1.00	15.00	A	N
ATOM	687	C	GLN	A	102	-16.278	-15.098	-2.532	1.00	15.00	A	C
ATOM	688	O	GLN	A	102	-15.899	-14.467	-1.545	1.00	15.00	A	O
ATOM	689	N	ASN	A	103	-16.592	-16.391	-2.490	1.00	15.00	A	N
ATOM	690	CA	ASN	A	103	-16.493	-17.168	-1.262	1.00	15.00	A	C
ATOM	691	CB	ASN	A	103	-17.175	-18.543	-1.403	1.00	15.00	A	C
ATOM	692	CG	ASN	A	103	-16.626	-19.398	-2.532	1.00	15.00	A	C
ATOM	693	OD1	ASN	A	103	-15.420	-19.581	-2.657	1.00	15.00	A	O
ATOM	694	ND2	ASN	A	103	-17.512	-19.924	-3.370	1.00	15.00	A	N
ATOM	695	C	ASN	A	103	-15.039	-17.279	-0.792	1.00	15.00	A	C
ATOM	696	O	ASN	A	103	-14.773	-17.500	0.392	1.00	15.00	A	O
ATOM	697	N	GLY	A	104	-14.108	-17.131	-1.735	1.00	15.00	A	N
ATOM	698	CA	GLY	A	104	-12.687	-17.169	-1.419	1.00	15.00	A	C
ATOM	699	C	GLY	A	104	-12.243	-18.499	-0.852	1.00	15.00	A	C
ATOM	700	O	GLY	A	104	-12.073	-19.466	-1.587	1.00	15.00	A	O
ATOM	701	N	SER	A	105	-12.033	-18.546	0.454	1.00	15.00	A	N
ATOM	702	CA	SER	A	105	-11.622	-19.774	1.108	1.00	15.00	A	C
ATOM	703	CB	SER	A	105	-10.902	-19.454	2.417	1.00	15.00	A	C

ATOM	704	OG	SER	A	105	-9.764	-20.279	2.589	1.00	15.00	A	O
ATOM	705	C	SER	A	105	-12.824	-20.675	1.377	1.00	15.00	A	C
ATOM	706	O	SER	A	105	-12.670	-21.853	1.687	1.00	15.00	A	O
ATOM	707	N	SER	A	106	-14.023	-20.124	1.223	1.00	15.00	A	N
ATOM	708	CA	SER	A	106	-15.247	-20.889	1.460	1.00	15.00	A	C
ATOM	709	CB	SER	A	106	-16.450	-19.962	1.686	1.00	15.00	A	C
ATOM	710	OG	SER	A	106	-16.219	-19.064	2.768	1.00	15.00	A	O
ATOM	711	C	SER	A	106	-15.518	-21.890	0.327	1.00	15.00	A	C
ATOM	712	O	SER	A	106	-16.577	-22.521	0.280	1.00	15.00	A	O
ATOM	713	N	VAL	A	107	-14.572	-22.003	-0.601	1.00	15.00	A	N
ATOM	714	CA	VAL	A	107	-14.700	-22.943	-1.700	1.00	15.00	A	C
ATOM	715	CB	VAL	A	107	-14.119	-22.375	-3.017	1.00	15.00	A	C
ATOM	716	CG1	VAL	A	107	-12.597	-22.456	-3.046	1.00	15.00	A	C
ATOM	717	CG2	VAL	A	107	-14.737	-23.065	-4.226	1.00	15.00	A	C
ATOM	718	C	VAL	A	107	-14.028	-24.278	-1.357	1.00	15.00	A	C
ATOM	719	O	VAL	A	107	-14.128	-25.244	-2.112	1.00	15.00	A	O
ATOM	720	N	LEU	A	108	-13.348	-24.337	-0.217	1.00	15.00	A	N
ATOM	721	CA	LEU	A	108	-12.672	-25.563	0.187	1.00	15.00	A	C
ATOM	722	CB	LEU	A	108	-11.239	-25.277	0.651	1.00	15.00	A	C
ATOM	723	CG	LEU	A	108	-10.294	-24.665	-0.386	1.00	15.00	A	C
ATOM	724	CD1	LEU	A	108	-8.980	-24.262	0.268	1.00	15.00	A	C
ATOM	725	CD2	LEU	A	108	-10.049	-25.638	-1.531	1.00	15.00	A	C
ATOM	726	C	LEU	A	108	-13.442	-26.274	1.285	1.00	15.00	A	C
ATOM	727	O	LEU	A	108	-14.398	-25.733	1.838	1.00	15.00	A	O
ATOM	728	N	SER	A	109	-13.028	-27.493	1.591	1.00	15.00	A	N
ATOM	729	CA	SER	A	109	-13.664	-28.267	2.633	1.00	15.00	A	C
ATOM	730	CB	SER	A	109	-13.158	-29.712	2.579	1.00	15.00	A	C
ATOM	731	OG	SER	A	109	-11.839	-29.764	2.052	1.00	15.00	A	O
ATOM	732	C	SER	A	109	-13.332	-27.638	3.978	1.00	15.00	A	C
ATOM	733	O	SER	A	109	-12.287	-26.994	4.115	1.00	15.00	A	O
ATOM	734	N	GLU	A	110	-14.214	-27.806	4.959	1.00	15.00	A	N
ATOM	735	CA	GLU	A	110	-13.997	-27.243	6.288	1.00	15.00	A	C
ATOM	736	CB	GLU	A	110	-15.053	-27.750	7.261	1.00	15.00	A	C
ATOM	737	CG	GLU	A	110	-16.482	-27.500	6.826	1.00	15.00	A	C
ATOM	738	CD	GLU	A	110	-17.461	-28.281	7.671	1.00	15.00	A	C
ATOM	739	OE1	GLU	A	110	-17.194	-28.441	8.882	1.00	15.00	A	O
ATOM	740	OE2	GLU	A	110	-18.479	-28.747	7.123	1.00	15.00	A	O
ATOM	741	C	GLU	A	110	-12.622	-27.629	6.807	1.00	15.00	A	C
ATOM	742	O	GLU	A	110	-11.934	-26.826	7.436	1.00	15.00	A	O
ATOM	743	N	ASP	A	111	-12.234	-28.869	6.530	1.00	15.00	A	N
ATOM	744	CA	ASP	A	111	-10.937	-29.389	6.944	1.00	15.00	A	C
ATOM	745	CB	ASP	A	111	-10.751	-30.825	6.450	1.00	15.00	A	C
ATOM	746	CG	ASP	A	111	-9.294	-31.249	6.442	1.00	15.00	A	C
ATOM	747	OD1	ASP	A	111	-8.687	-31.326	7.534	1.00	15.00	A	O
ATOM	748	OD2	ASP	A	111	-8.751	-31.488	5.345	1.00	15.00	A	O
ATOM	749	C	ASP	A	111	-9.799	-28.513	6.433	1.00	15.00	A	C
ATOM	750	O	ASP	A	111	-9.056	-27.927	7.222	1.00	15.00	A	O
ATOM	751	N	LYS	A	112	-9.686	-28.403	5.114	1.00	15.00	A	N
ATOM	752	CA	LYS	A	112	-8.630	-27.606	4.506	1.00	15.00	A	C
ATOM	753	CB	LYS	A	112	-8.571	-27.807	2.992	1.00	15.00	A	C
ATOM	754	CG	LYS	A	112	-8.191	-29.220	2.583	1.00	15.00	A	C
ATOM	755	CD	LYS	A	112	-7.805	-29.303	1.116	1.00	15.00	A	C
ATOM	756	CE	LYS	A	112	-7.393	-30.717	0.736	1.00	15.00	A	C
ATOM	757	NZ	LYS	A	112	-6.803	-30.777	-0.626	1.00	15.00	A	N
ATOM	758	C	LYS	A	112	-8.742	-26.131	4.874	1.00	15.00	A	C
ATOM	759	O	LYS	A	112	-7.734	-25.453	5.053	1.00	15.00	A	O
ATOM	760	N	SER	A	113	-9.972	-25.646	5.008	1.00	15.00	A	N
ATOM	761	CA	SER	A	113	-10.206	-24.257	5.380	1.00	15.00	A	C
ATOM	762	CB	SER	A	113	-11.705	-23.945	5.379	1.00	15.00	A	C
ATOM	763	OG	SER	A	113	-12.289	-24.249	4.125	1.00	15.00	A	O
ATOM	764	C	SER	A	113	-9.621	-23.980	6.756	1.00	15.00	A	C
ATOM	765	O	SER	A	113	-8.955	-22.972	6.970	1.00	15.00	A	O
ATOM	766	N	LYS	A	114	-9.862	-24.892	7.690	1.00	15.00	A	N
ATOM	767	CA	LYS	A	114	-9.345	-24.742	9.041	1.00	15.00	A	C
ATOM	768	CB	LYS	A	114	-9.945	-25.797	9.976	1.00	15.00	A	C
ATOM	769	CG	LYS	A	114	-11.426	-25.611	10.255	1.00	15.00	A	C
ATOM	770	CD	LYS	A	114	-11.978	-26.732	11.127	1.00	15.00	A	C
ATOM	771	CE	LYS	A	114	-11.827	-28.090	10.460	1.00	15.00	A	C
ATOM	772	NZ	LYS	A	114	-12.236	-29.205	11.358	1.00	15.00	A	N
ATOM	773	C	LYS	A	114	-7.834	-24.883	9.041	1.00	15.00	A	C
ATOM	774	O	LYS	A	114	-7.125	-24.125	9.702	1.00	15.00	A	O

ATOM	775	N	ARG	A	115	-7.356	-25.862	8.284	1.00	15.00	A	N
ATOM	776	CA	ARG	A	115	-5.935	-26.142	8.179	1.00	15.00	A	C
ATOM	777	CB	ARG	A	115	-5.692	-27.356	7.284	1.00	15.00	A	C
ATOM	778	CG	ARG	A	115	-4.362	-28.061	7.521	1.00	15.00	A	C
ATOM	779	CD	ARG	A	115	-4.258	-29.290	6.641	1.00	15.00	A	C
ATOM	780	NE	ARG	A	115	-5.367	-30.211	6.871	1.00	15.00	A	N
ATOM	781	CZ	ARG	A	115	-5.254	-31.533	6.784	1.00	15.00	A	C
ATOM	782	NH1	ARG	A	115	-4.083	-32.077	6.455	1.00	15.00	A	N
ATOM	783	NH2	ARG	A	115	-6.292	-32.319	7.063	1.00	15.00	A	N
ATOM	784	C	ARG	A	115	-5.185	-24.929	7.655	1.00	15.00	A	C
ATOM	785	O	ARG	A	115	-4.186	-24.514	8.243	1.00	15.00	A	O
ATOM	786	N	LEU	A	116	-5.696	-24.342	6.572	1.00	15.00	A	N
ATOM	787	CA	LEU	A	116	-5.083	-23.161	5.971	1.00	15.00	A	C
ATOM	788	CB	LEU	A	116	-5.842	-22.741	4.701	1.00	15.00	A	C
ATOM	789	CG	LEU	A	116	-5.314	-21.509	3.958	1.00	15.00	A	C
ATOM	790	CD1	LEU	A	116	-3.887	-21.735	3.490	1.00	15.00	A	C
ATOM	791	CD2	LEU	A	116	-6.212	-21.153	2.780	1.00	15.00	A	C
ATOM	792	C	LEU	A	116	-5.049	-22.009	6.973	1.00	15.00	A	C
ATOM	793	O	LEU	A	116	-4.024	-21.342	7.135	1.00	15.00	A	O
ATOM	794	N	ASN	A	117	-6.174	-21.796	7.662	1.00	15.00	A	N
ATOM	795	CA	ASN	A	117	-6.277	-20.737	8.664	1.00	15.00	A	C
ATOM	796	CB	ASN	A	117	-7.687	-20.689	9.260	1.00	15.00	A	C
ATOM	797	CG	ASN	A	117	-8.613	-19.781	8.476	1.00	15.00	A	C
ATOM	798	OD1	ASN	A	117	-8.198	-18.741	7.973	1.00	15.00	A	O
ATOM	799	ND2	ASN	A	117	-9.871	-20.174	8.356	1.00	15.00	A	N
ATOM	800	C	ASN	A	117	-5.249	-20.941	9.769	1.00	15.00	A	C
ATOM	801	O	ASN	A	117	-4.660	-19.984	10.275	1.00	15.00	A	O
ATOM	802	N	THR	A	118	-5.029	-22.200	10.125	1.00	15.00	A	N
ATOM	803	CA	THR	A	118	-4.062	-22.545	11.153	1.00	15.00	A	C
ATOM	804	CB	THR	A	118	-4.126	-24.049	11.491	1.00	15.00	A	C
ATOM	805	OG1	THR	A	118	-5.484	-24.410	11.779	1.00	15.00	A	O
ATOM	806	CG2	THR	A	118	-3.260	-24.372	12.699	1.00	15.00	A	C
ATOM	807	C	THR	A	118	-2.651	-22.175	10.695	1.00	15.00	A	C
ATOM	808	O	THR	A	118	-1.885	-21.564	11.443	1.00	15.00	A	O
ATOM	809	N	ILE	A	119	-2.324	-22.527	9.452	1.00	15.00	A	N
ATOM	810	CA	ILE	A	119	-1.012	-22.225	8.887	1.00	15.00	A	C
ATOM	811	CB	ILE	A	119	-0.874	-22.764	7.448	1.00	15.00	A	C
ATOM	812	CG1	ILE	A	119	-1.065	-24.281	7.424	1.00	15.00	A	C
ATOM	813	CG2	ILE	A	119	0.482	-22.392	6.862	1.00	15.00	A	C
ATOM	814	CD1	ILE	A	119	-1.285	-24.845	6.039	1.00	15.00	A	C
ATOM	815	C	ILE	A	119	-0.774	-20.718	8.880	1.00	15.00	A	C
ATOM	816	O	ILE	A	119	0.279	-20.245	9.308	1.00	15.00	A	O
ATOM	817	N	LEU	A	120	-1.772	-19.973	8.407	1.00	15.00	A	N
ATOM	818	CA	LEU	A	120	-1.687	-18.519	8.346	1.00	15.00	A	C
ATOM	819	CB	LEU	A	120	-2.975	-17.932	7.762	1.00	15.00	A	C
ATOM	820	CG	LEU	A	120	-3.300	-18.315	6.316	1.00	15.00	A	C
ATOM	821	CD1	LEU	A	120	-4.674	-17.795	5.922	1.00	15.00	A	C
ATOM	822	CD2	LEU	A	120	-2.234	-17.786	5.370	1.00	15.00	A	C
ATOM	823	C	LEU	A	120	-1.430	-17.934	9.729	1.00	15.00	A	C
ATOM	824	O	LEU	A	120	-0.543	-17.099	9.907	1.00	15.00	A	O
ATOM	825	N	ASN	A	121	-2.202	-18.397	10.707	1.00	15.00	A	N
ATOM	826	CA	ASN	A	121	-2.060	-17.930	12.082	1.00	15.00	A	C
ATOM	827	CB	ASN	A	121	-3.114	-18.574	12.988	1.00	15.00	A	C
ATOM	828	CG	ASN	A	121	-4.412	-17.791	13.034	1.00	15.00	A	C
ATOM	829	OD1	ASN	A	121	-4.590	-16.911	13.873	1.00	15.00	A	O
ATOM	830	ND2	ASN	A	121	-5.331	-18.108	12.135	1.00	15.00	A	N
ATOM	831	C	ASN	A	121	-0.665	-18.228	12.610	1.00	15.00	A	C
ATOM	832	O	ASN	A	121	0.001	-17.348	13.149	1.00	15.00	A	O
ATOM	833	N	THR	A	122	-0.220	-19.468	12.420	1.00	15.00	A	N
ATOM	834	CA	THR	A	122	1.097	-19.895	12.878	1.00	15.00	A	C
ATOM	835	CB	THR	A	122	1.339	-21.392	12.592	1.00	15.00	A	C
ATOM	836	OG1	THR	A	122	0.193	-22.148	13.008	1.00	15.00	A	O
ATOM	837	CG2	THR	A	122	2.559	-21.890	13.352	1.00	15.00	A	C
ATOM	838	C	THR	A	122	2.213	-19.057	12.248	1.00	15.00	A	C
ATOM	839	O	THR	A	122	3.150	-18.642	12.934	1.00	15.00	A	O
ATOM	840	N	MET	A	123	2.106	-18.800	10.947	1.00	15.00	A	N
ATOM	841	CA	MET	A	123	3.108	-18.003	10.246	1.00	15.00	A	C
ATOM	842	CB	MET	A	123	2.837	-17.968	8.738	1.00	15.00	A	C
ATOM	843	CG	MET	A	123	3.115	-19.282	8.026	1.00	15.00	A	C
ATOM	844	SD	MET	A	123	3.155	-19.118	6.229	1.00	15.00	A	S
ATOM	845	CE	MET	A	123	1.506	-18.503	5.900	1.00	15.00	A	C

ATOM	846	C	MET	A	123	3.151	-16.588	10.810	1.00	15.00	A	C
ATOM	847	O	MET	A	123	4.223	-16.011	10.993	1.00	15.00	A	O
ATOM	848	N	SER	A	124	1.976	-16.048	11.104	1.00	15.00	A	N
ATOM	849	CA	SER	A	124	1.865	-14.711	11.660	1.00	15.00	A	C
ATOM	850	CB	SER	A	124	0.394	-14.280	11.714	1.00	15.00	A	C
ATOM	851	OG	SER	A	124	0.270	-12.891	11.965	1.00	15.00	A	O
ATOM	852	C	SER	A	124	2.492	-14.649	13.054	1.00	15.00	A	C
ATOM	853	O	SER	A	124	3.286	-13.749	13.345	1.00	15.00	A	O
ATOM	854	N	THR	A	125	2.157	-15.622	13.903	1.00	15.00	A	N
ATOM	855	CA	THR	A	125	2.680	-15.668	15.263	1.00	15.00	A	C
ATOM	856	CB	THR	A	125	2.038	-16.794	16.100	1.00	15.00	A	C
ATOM	857	OG1	THR	A	125	1.750	-17.922	15.265	1.00	15.00	A	O
ATOM	858	CG2	THR	A	125	0.754	-16.309	16.754	1.00	15.00	A	C
ATOM	859	C	THR	A	125	4.201	-15.800	15.299	1.00	15.00	A	C
ATOM	860	O	THR	A	125	4.868	-15.100	16.059	1.00	15.00	A	O
ATOM	861	N	ILE	A	126	4.749	-16.681	14.468	1.00	15.00	A	N
ATOM	862	CA	ILE	A	126	6.194	-16.891	14.428	1.00	15.00	A	C
ATOM	863	CB	ILE	A	126	6.581	-18.068	13.501	1.00	15.00	A	C
ATOM	864	CG1	ILE	A	126	6.031	-19.388	14.051	1.00	15.00	A	C
ATOM	865	CG2	ILE	A	126	8.092	-18.155	13.331	1.00	15.00	A	C
ATOM	866	CD1	ILE	A	126	6.326	-20.587	13.175	1.00	15.00	A	C
ATOM	867	C	ILE	A	126	6.922	-15.621	13.989	1.00	15.00	A	C
ATOM	868	O	ILE	A	126	7.971	-15.274	14.527	1.00	15.00	A	O
ATOM	869	N	TYR	A	127	6.339	-14.915	13.031	1.00	15.00	A	N
ATOM	870	CA	TYR	A	127	6.930	-13.688	12.511	1.00	15.00	A	C
ATOM	871	CB	TYR	A	127	6.187	-13.243	11.248	1.00	15.00	A	C
ATOM	872	CG	TYR	A	127	6.748	-11.992	10.611	1.00	15.00	A	C
ATOM	873	CD1	TYR	A	127	7.837	-12.059	9.753	1.00	15.00	A	C
ATOM	874	CD2	TYR	A	127	6.193	-10.745	10.871	1.00	15.00	A	C
ATOM	875	CE1	TYR	A	127	8.357	-10.919	9.174	1.00	15.00	A	C
ATOM	876	CE2	TYR	A	127	6.706	-9.601	10.294	1.00	15.00	A	C
ATOM	877	CZ	TYR	A	127	7.789	-9.694	9.448	1.00	15.00	A	C
ATOM	878	OH	TYR	A	127	8.304	-8.556	8.867	1.00	15.00	A	O
ATOM	879	C	TYR	A	127	6.928	-12.561	13.547	1.00	15.00	A	C
ATOM	880	O	TYR	A	127	7.807	-11.698	13.539	1.00	15.00	A	O
ATOM	881	N	SER	A	128	5.952	-12.575	14.442	1.00	15.00	A	N
ATOM	882	CA	SER	A	128	5.839	-11.533	15.451	1.00	15.00	A	C
ATOM	883	CB	SER	A	128	4.367	-11.253	15.757	1.00	15.00	A	C
ATOM	884	OG	SER	A	128	3.597	-12.439	15.663	1.00	15.00	A	O
ATOM	885	C	SER	A	128	6.601	-11.877	16.726	1.00	15.00	A	C
ATOM	886	O	SER	A	128	7.251	-11.019	17.323	1.00	15.00	A	O
ATOM	887	N	THR	A	129	6.529	-13.133	17.141	1.00	15.00	A	N
ATOM	888	CA	THR	A	129	7.210	-13.558	18.352	1.00	15.00	A	C
ATOM	889	CB	THR	A	129	6.434	-14.654	19.110	1.00	15.00	A	C
ATOM	890	OG1	THR	A	129	6.169	-15.761	18.242	1.00	15.00	A	O
ATOM	891	CG2	THR	A	129	5.121	-14.107	19.648	1.00	15.00	A	C
ATOM	892	C	THR	A	129	8.639	-14.015	18.073	1.00	15.00	A	C
ATOM	893	O	THR	A	129	9.356	-14.417	18.989	1.00	15.00	A	O
ATOM	894	N	GLY	A	130	9.048	-13.954	16.812	1.00	15.00	A	N
ATOM	895	CA	GLY	A	130	10.395	-14.350	16.446	1.00	15.00	A	C
ATOM	896	C	GLY	A	130	11.441	-13.511	17.153	1.00	15.00	A	C
ATOM	897	O	GLY	A	130	11.350	-12.283	17.177	1.00	15.00	A	O
ATOM	898	N	LYS	A	131	12.428	-14.170	17.741	1.00	15.00	A	N
ATOM	899	CA	LYS	A	131	13.485	-13.480	18.463	1.00	15.00	A	C
ATOM	900	CB	LYS	A	131	13.102	-13.301	19.938	1.00	15.00	A	C
ATOM	901	CG	LYS	A	131	12.798	-14.603	20.669	1.00	15.00	A	C
ATOM	902	CD	LYS	A	131	12.372	-14.357	22.108	1.00	15.00	A	C
ATOM	903	CE	LYS	A	131	10.975	-13.761	22.184	1.00	15.00	A	C
ATOM	904	NZ	LYS	A	131	9.979	-14.588	21.453	1.00	15.00	A	N
ATOM	905	C	LYS	A	131	14.787	-14.256	18.352	1.00	15.00	A	C
ATOM	906	O	LYS	A	131	14.798	-15.478	18.468	1.00	15.00	A	O
ATOM	907	N	VAL	A	132	15.878	-13.548	18.124	1.00	15.00	A	N
ATOM	908	CA	VAL	A	132	17.174	-14.189	17.997	1.00	15.00	A	C
ATOM	909	CB	VAL	A	132	17.811	-13.958	16.611	1.00	15.00	A	C
ATOM	910	CG1	VAL	A	132	16.986	-14.645	15.534	1.00	15.00	A	C
ATOM	911	CG2	VAL	A	132	17.945	-12.472	16.313	1.00	15.00	A	C
ATOM	912	C	VAL	A	132	18.119	-13.741	19.103	1.00	15.00	A	C
ATOM	913	O	VAL	A	132	18.292	-12.546	19.351	1.00	15.00	A	O
ATOM	914	N	CYS	A	133	18.695	-14.708	19.791	1.00	15.00	A	N
ATOM	915	CA	CYS	A	133	19.617	-14.425	20.876	1.00	15.00	A	C
ATOM	916	C	CYS	A	133	21.058	-14.491	20.385	1.00	15.00	A	C

ATOM	917	O	CYS	A	133	21.353	-15.143	19.380	1.00	15.00	A	O
ATOM	918	CB	CYS	A	133	19.395	-15.421	22.019	1.00	15.00	A	C
ATOM	919	SG	CYS	A	133	17.706	-16.115	22.078	1.00	15.00	A	S
ATOM	920	N	ASN	A	134	21.947	-13.798	21.082	1.00	15.00	A	N
ATOM	921	CA	ASN	A	134	23.360	-13.787	20.726	1.00	15.00	A	C
ATOM	922	CB	ASN	A	134	24.054	-12.516	21.239	1.00	15.00	A	C
ATOM	923	CG	ASN	A	134	23.985	-12.363	22.745	1.00	15.00	A	C
ATOM	924	OD1	ASN	A	134	22.937	-12.554	23.349	1.00	15.00	A	O
ATOM	925	ND2	ASN	A	134	25.107	-12.042	23.363	1.00	15.00	A	N
ATOM	926	C	ASN	A	134	24.046	-15.032	21.280	1.00	15.00	A	C
ATOM	927	O	ASN	A	134	23.618	-15.579	22.295	1.00	15.00	A	O
ATOM	928	N	PRO	A	135	25.122	-15.496	20.627	1.00	15.00	A	N
ATOM	929	CA	PRO	A	135	25.847	-16.700	21.054	1.00	15.00	A	C
ATOM	930	CB	PRO	A	135	26.857	-16.927	19.924	1.00	15.00	A	C
ATOM	931	CG	PRO	A	135	27.053	-15.580	19.319	1.00	15.00	A	C
ATOM	932	CD	PRO	A	135	25.726	-14.884	19.428	1.00	15.00	A	C
ATOM	933	C	PRO	A	135	26.572	-16.524	22.387	1.00	15.00	A	C
ATOM	934	O	PRO	A	135	26.961	-17.504	23.022	1.00	15.00	A	O
ATOM	935	N	ASP	A	136	26.742	-15.278	22.808	1.00	15.00	A	N
ATOM	936	CA	ASP	A	136	27.434	-14.978	24.057	1.00	15.00	A	C
ATOM	937	CB	ASP	A	136	28.114	-13.603	23.994	1.00	15.00	A	C
ATOM	938	CG	ASP	A	136	28.285	-13.084	22.579	1.00	15.00	A	C
ATOM	939	OD1	ASP	A	136	29.365	-13.291	21.994	1.00	15.00	A	O
ATOM	940	OD2	ASP	A	136	27.326	-12.478	22.048	1.00	15.00	A	O
ATOM	941	C	ASP	A	136	26.497	-15.042	25.261	1.00	15.00	A	C
ATOM	942	O	ASP	A	136	26.913	-15.418	26.357	1.00	15.00	A	O
ATOM	943	N	ASN	A	137	25.234	-14.680	25.066	1.00	15.00	A	N
ATOM	944	CA	ASN	A	137	24.267	-14.695	26.160	1.00	15.00	A	C
ATOM	945	CB	ASN	A	137	24.080	-13.294	26.752	1.00	15.00	A	C
ATOM	946	CG	ASN	A	137	23.218	-13.296	28.003	1.00	15.00	A	C
ATOM	947	OD1	ASN	A	137	23.027	-14.332	28.643	1.00	15.00	A	O
ATOM	948	ND2	ASN	A	137	22.705	-12.134	28.373	1.00	15.00	A	N
ATOM	949	C	ASN	A	137	22.931	-15.285	25.718	1.00	15.00	A	C
ATOM	950	O	ASN	A	137	22.181	-14.667	24.970	1.00	15.00	A	O
ATOM	951	N	PRO	A	138	22.612	-16.492	26.204	1.00	15.00	A	N
ATOM	952	CA	PRO	A	138	21.372	-17.197	25.850	1.00	15.00	A	C
ATOM	953	CB	PRO	A	138	21.493	-18.540	26.582	1.00	15.00	A	C
ATOM	954	CG	PRO	A	138	22.941	-18.675	26.909	1.00	15.00	A	C
ATOM	955	CD	PRO	A	138	23.440	-17.278	27.130	1.00	15.00	A	C
ATOM	956	C	PRO	A	138	20.097	-16.471	26.287	1.00	15.00	A	C
ATOM	957	O	PRO	A	138	19.016	-16.767	25.785	1.00	15.00	A	O
ATOM	958	N	GLN	A	139	20.213	-15.528	27.216	1.00	15.00	A	N
ATOM	959	CA	GLN	A	139	19.038	-14.799	27.694	1.00	15.00	A	C
ATOM	960	CB	GLN	A	139	19.082	-14.584	29.212	1.00	15.00	A	C
ATOM	961	CG	GLN	A	139	20.206	-13.678	29.686	1.00	15.00	A	C
ATOM	962	CD	GLN	A	139	20.058	-13.247	31.132	1.00	15.00	A	C
ATOM	963	OE1	GLN	A	139	18.950	-13.078	31.635	1.00	15.00	A	O
ATOM	964	NE2	GLN	A	139	21.180	-13.069	31.814	1.00	15.00	A	N
ATOM	965	C	GLN	A	139	18.833	-13.471	26.963	1.00	15.00	A	C
ATOM	966	O	GLN	A	139	17.826	-12.794	27.167	1.00	15.00	A	O
ATOM	967	N	GLU	A	140	19.775	-13.107	26.103	1.00	15.00	A	N
ATOM	968	CA	GLU	A	140	19.678	-11.854	25.363	1.00	15.00	A	C
ATOM	969	CB	GLU	A	140	21.035	-11.153	25.298	1.00	15.00	A	C
ATOM	970	CG	GLU	A	140	21.049	-9.770	25.927	1.00	15.00	A	C
ATOM	971	CD	GLU	A	140	22.449	-9.301	26.268	1.00	15.00	A	C
ATOM	972	OE1	GLU	A	140	22.966	-8.408	25.568	1.00	15.00	A	O
ATOM	973	OE2	GLU	A	140	23.030	-9.830	27.241	1.00	15.00	A	O
ATOM	974	C	GLU	A	140	19.116	-12.088	23.964	1.00	15.00	A	C
ATOM	975	O	GLU	A	140	19.854	-12.153	22.981	1.00	15.00	A	O
ATOM	976	N	CYS	A	141	17.804	-12.234	23.885	1.00	15.00	A	N
ATOM	977	CA	CYS	A	141	17.138	-12.465	22.613	1.00	15.00	A	C
ATOM	978	C	CYS	A	141	16.444	-11.200	22.121	1.00	15.00	A	C
ATOM	979	O	CYS	A	141	15.614	-10.622	22.823	1.00	15.00	A	O
ATOM	980	CB	CYS	A	141	16.131	-13.606	22.748	1.00	15.00	A	C
ATOM	981	SG	CYS	A	141	16.793	-15.089	23.574	1.00	15.00	A	S
ATOM	982	N	LEU	A	142	16.782	-10.777	20.910	1.00	15.00	A	N
ATOM	983	CA	LEU	A	142	16.199	-9.578	20.328	1.00	15.00	A	C
ATOM	984	CB	LEU	A	142	17.290	-8.687	19.725	1.00	15.00	A	C
ATOM	985	CG	LEU	A	142	18.296	-8.084	20.708	1.00	15.00	A	C
ATOM	986	CD1	LEU	A	142	19.416	-7.379	19.959	1.00	15.00	A	C
ATOM	987	CD2	LEU	A	142	17.603	-7.125	21.664	1.00	15.00	A	C

ATOM	988	C	LEU	A	142	15.169	-9.926	19.261	1.00	15.00	A	C
ATOM	989	O	LEU	A	142	15.397	-10.797	18.420	1.00	15.00	A	O
ATOM	990	N	LEU	A	143	14.030	-9.249	19.313	1.00	15.00	A	N
ATOM	991	CA	LEU	A	143	12.962	-9.456	18.345	1.00	15.00	A	C
ATOM	992	CB	LEU	A	143	11.620	-9.037	18.954	1.00	15.00	A	C
ATOM	993	CG	LEU	A	143	11.196	-9.749	20.239	1.00	15.00	A	C
ATOM	994	CD1	LEU	A	143	10.550	-8.765	21.202	1.00	15.00	A	C
ATOM	995	CD2	LEU	A	143	10.239	-10.886	19.926	1.00	15.00	A	C
ATOM	996	C	LEU	A	143	13.234	-8.607	17.111	1.00	15.00	A	C
ATOM	997	O	LEU	A	143	14.126	-7.755	17.129	1.00	15.00	A	O
ATOM	998	N	LEU	A	144	12.477	-8.837	16.042	1.00	15.00	A	N
ATOM	999	CA	LEU	A	144	12.644	-8.060	14.821	1.00	15.00	A	C
ATOM	1000	CB	LEU	A	144	11.689	-8.546	13.727	1.00	15.00	A	C
ATOM	1001	CG	LEU	A	144	11.705	-7.747	12.418	1.00	15.00	A	C
ATOM	1002	CD1	LEU	A	144	12.988	-8.000	11.643	1.00	15.00	A	C
ATOM	1003	CD2	LEU	A	144	10.485	-8.072	11.571	1.00	15.00	A	C
ATOM	1004	C	LEU	A	144	12.392	-6.587	15.110	1.00	15.00	A	C
ATOM	1005	O	LEU	A	144	13.242	-5.736	14.855	1.00	15.00	A	O
ATOM	1006	N	GLU	A	145	11.231	-6.307	15.674	1.00	15.00	A	N
ATOM	1007	CA	GLU	A	145	10.853	-4.948	16.013	1.00	15.00	A	C
ATOM	1008	CB	GLU	A	145	9.428	-4.667	15.522	1.00	15.00	A	C
ATOM	1009	CG	GLU	A	145	8.922	-3.258	15.804	1.00	15.00	A	C
ATOM	1010	CD	GLU	A	145	9.799	-2.181	15.194	1.00	15.00	A	C
ATOM	1011	OE1	GLU	A	145	10.565	-2.494	14.258	1.00	15.00	A	O
ATOM	1012	OE2	GLU	A	145	9.727	-1.026	15.655	1.00	15.00	A	O
ATOM	1013	C	GLU	A	145	10.940	-4.739	17.520	1.00	15.00	A	C
ATOM	1014	O	GLU	A	145	10.325	-5.474	18.291	1.00	15.00	A	O
ATOM	1015	N	PRO	A	146	11.748	-3.768	17.965	1.00	15.00	A	N
ATOM	1016	CA	PRO	A	146	12.563	-2.931	17.094	1.00	15.00	A	C
ATOM	1017	CB	PRO	A	146	12.368	-1.570	17.758	1.00	15.00	A	C
ATOM	1018	CG	PRO	A	146	12.270	-1.876	19.227	1.00	15.00	A	C
ATOM	1019	CD	PRO	A	146	11.871	-3.331	19.361	1.00	15.00	A	C
ATOM	1020	C	PRO	A	146	14.037	-3.321	17.189	1.00	15.00	A	C
ATOM	1021	O	PRO	A	146	14.912	-2.584	16.747	1.00	15.00	A	O
ATOM	1022	N	GLY	A	147	14.294	-4.491	17.764	1.00	15.00	A	N
ATOM	1023	CA	GLY	A	147	15.656	-4.958	17.951	1.00	15.00	A	C
ATOM	1024	C	GLY	A	147	16.431	-5.126	16.660	1.00	15.00	A	C
ATOM	1025	O	GLY	A	147	17.345	-4.357	16.371	1.00	15.00	A	O
ATOM	1026	N	LEU	A	148	16.064	-6.130	15.883	1.00	15.00	A	N
ATOM	1027	CA	LEU	A	148	16.745	-6.413	14.628	1.00	15.00	A	C
ATOM	1028	CB	LEU	A	148	16.246	-7.725	14.028	1.00	15.00	A	C
ATOM	1029	CG	LEU	A	148	16.511	-8.980	14.860	1.00	15.00	A	C
ATOM	1030	CD1	LEU	A	148	16.037	-10.221	14.122	1.00	15.00	A	C
ATOM	1031	CD2	LEU	A	148	17.987	-9.088	15.211	1.00	15.00	A	C
ATOM	1032	C	LEU	A	148	16.605	-5.274	13.623	1.00	15.00	A	C
ATOM	1033	O	LEU	A	148	17.531	-4.997	12.860	1.00	15.00	A	O
ATOM	1034	N	ASN	A	149	15.454	-4.615	13.632	1.00	15.00	A	N
ATOM	1035	CA	ASN	A	149	15.204	-3.508	12.716	1.00	15.00	A	C
ATOM	1036	CB	ASN	A	149	13.752	-3.030	12.791	1.00	15.00	A	C
ATOM	1037	CG	ASN	A	149	12.820	-3.871	11.938	1.00	15.00	A	C
ATOM	1038	OD1	ASN	A	149	13.253	-4.545	11.002	1.00	15.00	A	O
ATOM	1039	ND2	ASN	A	149	11.536	-3.833	12.246	1.00	15.00	A	N
ATOM	1040	C	ASN	A	149	16.167	-2.355	12.957	1.00	15.00	A	C
ATOM	1041	O	ASN	A	149	16.697	-1.780	12.010	1.00	15.00	A	O
ATOM	1042	N	GLU	A	150	16.402	-2.034	14.226	1.00	15.00	A	N
ATOM	1043	CA	GLU	A	150	17.312	-0.954	14.590	1.00	15.00	A	C
ATOM	1044	CB	GLU	A	150	17.292	-0.736	16.108	1.00	15.00	A	C
ATOM	1045	CG	GLU	A	150	17.953	0.550	16.582	1.00	15.00	A	C
ATOM	1046	CD	GLU	A	150	19.461	0.427	16.701	1.00	15.00	A	C
ATOM	1047	OE1	GLU	A	150	20.164	1.417	16.414	1.00	15.00	A	O
ATOM	1048	OE2	GLU	A	150	19.939	-0.665	17.070	1.00	15.00	A	O
ATOM	1049	C	GLU	A	150	18.724	-1.275	14.109	1.00	15.00	A	C
ATOM	1050	O	GLU	A	150	19.361	-0.466	13.435	1.00	15.00	A	O
ATOM	1051	N	ILE	A	151	19.189	-2.476	14.430	1.00	15.00	A	N
ATOM	1052	CA	ILE	A	151	20.523	-2.918	14.036	1.00	15.00	A	C
ATOM	1053	CB	ILE	A	151	20.810	-4.352	14.537	1.00	15.00	A	C
ATOM	1054	CG1	ILE	A	151	20.720	-4.409	16.065	1.00	15.00	A	C
ATOM	1055	CG2	ILE	A	151	22.177	-4.831	14.061	1.00	15.00	A	C
ATOM	1056	CD1	ILE	A	151	20.864	-5.803	16.638	1.00	15.00	A	C
ATOM	1057	C	ILE	A	151	20.697	-2.863	12.516	1.00	15.00	A	C
ATOM	1058	O	ILE	A	151	21.729	-2.426	12.008	1.00	15.00	A	O

ATOM	1059	N	MET	A	152	19.668	-3.273	11.791	1.00	15.00	A	N
ATOM	1060	CA	MET	A	152	19.722	-3.279	10.336	1.00	15.00	A	C
ATOM	1061	CB	MET	A	152	18.764	-4.322	9.759	1.00	15.00	A	C
ATOM	1062	CG	MET	A	152	19.148	-5.757	10.085	1.00	15.00	A	C
ATOM	1063	SD	MET	A	152	20.754	-6.221	9.412	1.00	15.00	A	S
ATOM	1064	CE	MET	A	152	20.338	-6.472	7.691	1.00	15.00	A	C
ATOM	1065	C	MET	A	152	19.438	-1.902	9.742	1.00	15.00	A	C
ATOM	1066	O	MET	A	152	19.373	-1.751	8.523	1.00	15.00	A	O
ATOM	1067	N	ALA	A	153	19.284	-0.898	10.592	1.00	15.00	A	N
ATOM	1068	CA	ALA	A	153	19.007	0.451	10.122	1.00	15.00	A	C
ATOM	1069	CB	ALA	A	153	17.624	0.902	10.571	1.00	15.00	A	C
ATOM	1070	C	ALA	A	153	20.067	1.453	10.570	1.00	15.00	A	C
ATOM	1071	O	ALA	A	153	20.552	2.249	9.766	1.00	15.00	A	O
ATOM	1072	N	ASN	A	154	20.439	1.400	11.843	1.00	15.00	A	N
ATOM	1073	CA	ASN	A	154	21.411	2.339	12.394	1.00	15.00	A	C
ATOM	1074	CB	ASN	A	154	20.941	2.881	13.746	1.00	15.00	A	C
ATOM	1075	CG	ASN	A	154	19.695	3.738	13.627	1.00	15.00	A	C
ATOM	1076	OD1	ASN	A	154	18.572	3.236	13.643	1.00	15.00	A	O
ATOM	1077	ND2	ASN	A	154	19.881	5.045	13.503	1.00	15.00	A	N
ATOM	1078	C	ASN	A	154	22.826	1.774	12.501	1.00	15.00	A	C
ATOM	1079	O	ASN	A	154	23.798	2.514	12.336	1.00	15.00	A	O
ATOM	1080	N	SER	A	155	22.956	0.481	12.788	1.00	15.00	A	N
ATOM	1081	CA	SER	A	155	24.279	-0.131	12.908	1.00	15.00	A	C
ATOM	1082	CB	SER	A	155	24.204	-1.564	13.439	1.00	15.00	A	C
ATOM	1083	OG	SER	A	155	25.466	-2.207	13.341	1.00	15.00	A	O
ATOM	1084	C	SER	A	155	25.024	-0.085	11.579	1.00	15.00	A	C
ATOM	1085	O	SER	A	155	24.435	-0.285	10.513	1.00	15.00	A	O
ATOM	1086	N	LEU	A	156	26.323	0.171	11.649	1.00	15.00	A	N
ATOM	1087	CA	LEU	A	156	27.148	0.265	10.456	1.00	15.00	A	C
ATOM	1088	CB	LEU	A	156	27.765	1.663	10.349	1.00	15.00	A	C
ATOM	1089	CG	LEU	A	156	26.784	2.827	10.181	1.00	15.00	A	C
ATOM	1090	CD1	LEU	A	156	27.491	4.155	10.401	1.00	15.00	A	C
ATOM	1091	CD2	LEU	A	156	26.133	2.788	8.807	1.00	15.00	A	C
ATOM	1092	C	LEU	A	156	28.247	-0.790	10.444	1.00	15.00	A	C
ATOM	1093	O	LEU	A	156	29.163	-0.728	9.622	1.00	15.00	A	O
ATOM	1094	N	ASP	A	157	28.164	-1.760	11.348	1.00	15.00	A	N
ATOM	1095	CA	ASP	A	157	29.173	-2.812	11.398	1.00	15.00	A	C
ATOM	1096	CB	ASP	A	157	29.393	-3.353	12.809	1.00	15.00	A	C
ATOM	1097	CG	ASP	A	157	30.456	-4.437	12.827	1.00	15.00	A	C
ATOM	1098	OD1	ASP	A	157	31.652	-4.104	12.706	1.00	15.00	A	O
ATOM	1099	OD2	ASP	A	157	30.093	-5.625	12.923	1.00	15.00	A	O
ATOM	1100	C	ASP	A	157	28.826	-3.939	10.438	1.00	15.00	A	C
ATOM	1101	O	ASP	A	157	27.673	-4.365	10.350	1.00	15.00	A	O
ATOM	1102	N	TYR	A	158	29.829	-4.419	9.722	1.00	15.00	A	N
ATOM	1103	CA	TYR	A	158	29.638	-5.478	8.744	1.00	15.00	A	C
ATOM	1104	CB	TYR	A	158	30.890	-5.636	7.874	1.00	15.00	A	C
ATOM	1105	CG	TYR	A	158	30.724	-6.614	6.732	1.00	15.00	A	C
ATOM	1106	CD1	TYR	A	158	31.095	-7.947	6.871	1.00	15.00	A	C
ATOM	1107	CD2	TYR	A	158	30.191	-6.207	5.516	1.00	15.00	A	C
ATOM	1108	CE1	TYR	A	158	30.940	-8.844	5.833	1.00	15.00	A	C
ATOM	1109	CE2	TYR	A	158	30.033	-7.098	4.472	1.00	15.00	A	C
ATOM	1110	CZ	TYR	A	158	30.408	-8.415	4.635	1.00	15.00	A	C
ATOM	1111	OH	TYR	A	158	30.249	-9.304	3.599	1.00	15.00	A	O
ATOM	1112	C	TYR	A	158	29.274	-6.810	9.395	1.00	15.00	A	C
ATOM	1113	O	TYR	A	158	28.464	-7.564	8.858	1.00	15.00	A	O
ATOM	1114	N	ASN	A	159	29.850	-7.084	10.556	1.00	15.00	A	N
ATOM	1115	CA	ASN	A	159	29.604	-8.344	11.251	1.00	15.00	A	C
ATOM	1116	CB	ASN	A	159	30.741	-8.663	12.226	1.00	15.00	A	C
ATOM	1117	CG	ASN	A	159	31.989	-9.166	11.526	1.00	15.00	A	C
ATOM	1118	OD1	ASN	A	159	31.914	-9.866	10.518	1.00	15.00	A	O
ATOM	1119	ND2	ASN	A	159	33.149	-8.804	12.053	1.00	15.00	A	N
ATOM	1120	C	ASN	A	159	28.267	-8.336	11.979	1.00	15.00	A	C
ATOM	1121	O	ASN	A	159	27.484	-9.280	11.863	1.00	15.00	A	O
ATOM	1122	N	GLU	A	160	28.012	-7.268	12.725	1.00	15.00	A	N
ATOM	1123	CA	GLU	A	160	26.769	-7.129	13.477	1.00	15.00	A	C
ATOM	1124	CB	GLU	A	160	26.759	-5.809	14.248	1.00	15.00	A	C
ATOM	1125	CG	GLU	A	160	25.647	-5.693	15.277	1.00	15.00	A	C
ATOM	1126	CD	GLU	A	160	25.761	-4.434	16.113	1.00	15.00	A	C
ATOM	1127	OE1	GLU	A	160	25.529	-4.509	17.336	1.00	15.00	A	O
ATOM	1128	OE2	GLU	A	160	26.086	-3.370	15.541	1.00	15.00	A	O
ATOM	1129	C	GLU	A	160	25.555	-7.225	12.555	1.00	15.00	A	C

ATOM	1130	O	GLU	A	160	24.610	-7.969	12.829	1.00	15.00	A	O
ATOM	1131	N	ARG	A	161	25.594	-6.487	11.449	1.00	15.00	A	N
ATOM	1132	CA	ARG	A	161	24.498	-6.502	10.485	1.00	15.00	A	C
ATOM	1133	CB	ARG	A	161	24.687	-5.418	9.422	1.00	15.00	A	C
ATOM	1134	CG	ARG	A	161	24.615	-4.000	9.968	1.00	15.00	A	C
ATOM	1135	CD	ARG	A	161	24.746	-2.971	8.857	1.00	15.00	A	C
ATOM	1136	NE	ARG	A	161	23.639	-3.059	7.907	1.00	15.00	A	N
ATOM	1137	CZ	ARG	A	161	22.607	-2.219	7.882	1.00	15.00	A	C
ATOM	1138	NH1	ARG	A	161	22.538	-1.207	8.743	1.00	15.00	A	N
ATOM	1139	NH2	ARG	A	161	21.635	-2.401	6.998	1.00	15.00	A	N
ATOM	1140	C	ARG	A	161	24.368	-7.877	9.837	1.00	15.00	A	C
ATOM	1141	O	ARG	A	161	23.264	-8.338	9.551	1.00	15.00	A	O
ATOM	1142	N	LEU	A	162	25.504	-8.532	9.618	1.00	15.00	A	N
ATOM	1143	CA	LEU	A	162	25.521	-9.863	9.025	1.00	15.00	A	C
ATOM	1144	CB	LEU	A	162	26.966	-10.307	8.764	1.00	15.00	A	C
ATOM	1145	CG	LEU	A	162	27.177	-11.765	8.349	1.00	15.00	A	C
ATOM	1146	CD1	LEU	A	162	26.566	-12.031	6.983	1.00	15.00	A	C
ATOM	1147	CD2	LEU	A	162	28.658	-12.112	8.354	1.00	15.00	A	C
ATOM	1148	C	LEU	A	162	24.825	-10.853	9.952	1.00	15.00	A	C
ATOM	1149	O	LEU	A	162	24.038	-11.691	9.510	1.00	15.00	A	O
ATOM	1150	N	TRP	A	163	25.114	-10.731	11.244	1.00	15.00	A	N
ATOM	1151	CA	TRP	A	163	24.526	-11.595	12.257	1.00	15.00	A	C
ATOM	1152	CB	TRP	A	163	25.092	-11.255	13.642	1.00	15.00	A	C
ATOM	1153	CG	TRP	A	163	24.382	-11.933	14.777	1.00	15.00	A	C
ATOM	1154	CD1	TRP	A	163	24.496	-13.241	15.150	1.00	15.00	A	C
ATOM	1155	CD2	TRP	A	163	23.445	-11.337	15.687	1.00	15.00	A	C
ATOM	1156	NE1	TRP	A	163	23.689	-13.497	16.233	1.00	15.00	A	N
ATOM	1157	CE2	TRP	A	163	23.035	-12.345	16.582	1.00	15.00	A	C
ATOM	1158	CE3	TRP	A	163	22.916	-10.050	15.833	1.00	15.00	A	C
ATOM	1159	CZ2	TRP	A	163	22.119	-12.106	17.605	1.00	15.00	A	C
ATOM	1160	CZ3	TRP	A	163	22.008	-9.816	16.849	1.00	15.00	A	C
ATOM	1161	CH2	TRP	A	163	21.620	-10.838	17.723	1.00	15.00	A	C
ATOM	1162	C	TRP	A	163	23.007	-11.468	12.266	1.00	15.00	A	C
ATOM	1163	O	TRP	A	163	22.297	-12.468	12.191	1.00	15.00	A	O
ATOM	1164	N	ALA	A	164	22.522	-10.233	12.350	1.00	15.00	A	N
ATOM	1165	CA	ALA	A	164	21.086	-9.968	12.377	1.00	15.00	A	C
ATOM	1166	CB	ALA	A	164	20.826	-8.484	12.590	1.00	15.00	A	C
ATOM	1167	C	ALA	A	164	20.406	-10.455	11.102	1.00	15.00	A	C
ATOM	1168	O	ALA	A	164	19.311	-11.024	11.144	1.00	15.00	A	O
ATOM	1169	N	TRP	A	165	21.066	-10.233	9.972	1.00	15.00	A	N
ATOM	1170	CA	TRP	A	165	20.542	-10.643	8.678	1.00	15.00	A	C
ATOM	1171	CB	TRP	A	165	21.482	-10.173	7.561	1.00	15.00	A	C
ATOM	1172	CG	TRP	A	165	20.921	-10.335	6.179	1.00	15.00	A	C
ATOM	1173	CD1	TRP	A	165	20.199	-9.414	5.481	1.00	15.00	A	C
ATOM	1174	CD2	TRP	A	165	21.040	-11.482	5.325	1.00	15.00	A	C
ATOM	1175	NE1	TRP	A	165	19.851	-9.915	4.253	1.00	15.00	A	N
ATOM	1176	CE2	TRP	A	165	20.356	-11.183	4.132	1.00	15.00	A	C
ATOM	1177	CE3	TRP	A	165	21.654	-12.732	5.454	1.00	15.00	A	C
ATOM	1178	CZ2	TRP	A	165	20.270	-12.087	3.077	1.00	15.00	A	C
ATOM	1179	CZ3	TRP	A	165	21.568	-13.627	4.405	1.00	15.00	A	C
ATOM	1180	CH2	TRP	A	165	20.881	-13.300	3.231	1.00	15.00	A	C
ATOM	1181	C	TRP	A	165	20.387	-12.157	8.608	1.00	15.00	A	C
ATOM	1182	O	TRP	A	165	19.367	-12.666	8.131	1.00	15.00	A	O
ATOM	1183	N	GLU	A	166	21.400	-12.866	9.083	1.00	15.00	A	N
ATOM	1184	CA	GLU	A	166	21.392	-14.318	9.058	1.00	15.00	A	C
ATOM	1185	CB	GLU	A	166	22.811	-14.878	9.200	1.00	15.00	A	C
ATOM	1186	CG	GLU	A	166	22.911	-16.390	9.030	1.00	15.00	A	C
ATOM	1187	CD	GLU	A	166	22.270	-16.882	7.745	1.00	15.00	A	C
ATOM	1188	OE1	GLU	A	166	22.256	-16.125	6.753	1.00	15.00	A	O
ATOM	1189	OE2	GLU	A	166	21.774	-18.028	7.721	1.00	15.00	A	O
ATOM	1190	C	GLU	A	166	20.474	-14.913	10.116	1.00	15.00	A	C
ATOM	1191	O	GLU	A	166	19.687	-15.805	9.811	1.00	15.00	A	O
ATOM	1192	N	SER	A	167	20.548	-14.403	11.343	1.00	15.00	A	N
ATOM	1193	CA	SER	A	167	19.742	-14.933	12.442	1.00	15.00	A	C
ATOM	1194	CB	SER	A	167	20.063	-14.233	13.769	1.00	15.00	A	C
ATOM	1195	OG	SER	A	167	19.909	-12.829	13.671	1.00	15.00	A	O
ATOM	1196	C	SER	A	167	18.243	-14.928	12.142	1.00	15.00	A	C
ATOM	1197	O	SER	A	167	17.549	-15.915	12.404	1.00	15.00	A	O
ATOM	1198	N	TRP	A	168	17.751	-13.834	11.568	1.00	15.00	A	N
ATOM	1199	CA	TRP	A	168	16.336	-13.710	11.239	1.00	15.00	A	C
ATOM	1200	CB	TRP	A	168	16.040	-12.310	10.683	1.00	15.00	A	C

ATOM	1201	CG	TRP	A	168	14.585	-12.046	10.430	1.00	15.00	A	C
ATOM	1202	CD1	TRP	A	168	14.013	-11.698	9.243	1.00	15.00	A	C
ATOM	1203	CD2	TRP	A	168	13.519	-12.109	11.388	1.00	15.00	A	C
ATOM	1204	NE1	TRP	A	168	12.657	-11.539	9.401	1.00	15.00	A	N
ATOM	1205	CE2	TRP	A	168	12.329	-11.788	10.708	1.00	15.00	A	C
ATOM	1206	CE3	TRP	A	168	13.455	-12.408	12.753	1.00	15.00	A	C
ATOM	1207	CZ2	TRP	A	168	11.089	-11.756	11.348	1.00	15.00	A	C
ATOM	1208	CZ3	TRP	A	168	12.225	-12.375	13.383	1.00	15.00	A	C
ATOM	1209	CH2	TRP	A	168	11.060	-12.054	12.682	1.00	15.00	A	C
ATOM	1210	C	TRP	A	168	15.881	-14.787	10.250	1.00	15.00	A	C
ATOM	1211	O	TRP	A	168	14.721	-15.200	10.249	1.00	15.00	A	O
ATOM	1212	N	ARG	A	169	16.795	-15.268	9.427	1.00	15.00	A	N
ATOM	1213	CA	ARG	A	169	16.445	-16.269	8.431	1.00	15.00	A	C
ATOM	1214	CB	ARG	A	169	17.031	-15.912	7.065	1.00	15.00	A	C
ATOM	1215	CG	ARG	A	169	16.130	-14.998	6.245	1.00	15.00	A	C
ATOM	1216	CD	ARG	A	169	16.920	-14.089	5.318	1.00	15.00	A	C
ATOM	1217	NE	ARG	A	169	17.473	-12.940	6.029	1.00	15.00	A	N
ATOM	1218	CZ	ARG	A	169	17.038	-11.684	5.871	1.00	15.00	A	C
ATOM	1219	NH1	ARG	A	169	16.046	-11.414	5.029	1.00	15.00	A	N
ATOM	1220	NH2	ARG	A	169	17.589	-10.702	6.563	1.00	15.00	A	N
ATOM	1221	C	ARG	A	169	16.812	-17.688	8.850	1.00	15.00	A	C
ATOM	1222	O	ARG	A	169	16.112	-18.633	8.502	1.00	15.00	A	O
ATOM	1223	N	SER	A	170	17.892	-17.838	9.604	1.00	15.00	A	N
ATOM	1224	CA	SER	A	170	18.329	-19.158	10.062	1.00	15.00	A	C
ATOM	1225	CB	SER	A	170	19.758	-19.105	10.622	1.00	15.00	A	C
ATOM	1226	OG	SER	A	170	20.001	-17.883	11.293	1.00	15.00	A	O
ATOM	1227	C	SER	A	170	17.372	-19.740	11.100	1.00	15.00	A	C
ATOM	1228	O	SER	A	170	17.143	-20.950	11.144	1.00	15.00	A	O
ATOM	1229	N	GLU	A	171	16.806	-18.861	11.912	1.00	15.00	A	N
ATOM	1230	CA	GLU	A	171	15.881	-19.266	12.957	1.00	15.00	A	C
ATOM	1231	CB	GLU	A	171	16.040	-18.349	14.174	1.00	15.00	A	C
ATOM	1232	CG	GLU	A	171	16.109	-19.069	15.512	1.00	15.00	A	C
ATOM	1233	CD	GLU	A	171	14.762	-19.591	15.970	1.00	15.00	A	C
ATOM	1234	OE1	GLU	A	171	14.591	-20.828	16.024	1.00	15.00	A	O
ATOM	1235	OE2	GLU	A	171	13.872	-18.764	16.266	1.00	15.00	A	O
ATOM	1236	C	GLU	A	171	14.441	-19.242	12.452	1.00	15.00	A	C
ATOM	1237	O	GLU	A	171	13.911	-20.261	12.005	1.00	15.00	A	O
ATOM	1238	N	VAL	A	172	13.830	-18.063	12.506	1.00	15.00	A	N
ATOM	1239	CA	VAL	A	172	12.450	-17.867	12.073	1.00	15.00	A	C
ATOM	1240	CB	VAL	A	172	12.045	-16.385	12.241	1.00	15.00	A	C
ATOM	1241	CG1	VAL	A	172	10.609	-16.143	11.798	1.00	15.00	A	C
ATOM	1242	CG2	VAL	A	172	12.250	-15.936	13.679	1.00	15.00	A	C
ATOM	1243	C	VAL	A	172	12.233	-18.291	10.618	1.00	15.00	A	C
ATOM	1244	O	VAL	A	172	11.239	-18.944	10.297	1.00	15.00	A	O
ATOM	1245	N	GLY	A	173	13.174	-17.931	9.750	1.00	15.00	A	N
ATOM	1246	CA	GLY	A	173	13.065	-18.255	8.334	1.00	15.00	A	C
ATOM	1247	C	GLY	A	173	12.844	-19.733	8.042	1.00	15.00	A	C
ATOM	1248	O	GLY	A	173	11.978	-20.088	7.241	1.00	15.00	A	O
ATOM	1249	N	LYS	A	174	13.621	-20.598	8.691	1.00	15.00	A	N
ATOM	1250	CA	LYS	A	174	13.504	-22.037	8.475	1.00	15.00	A	C
ATOM	1251	CB	LYS	A	174	14.683	-22.798	9.096	1.00	15.00	A	C
ATOM	1252	CG	LYS	A	174	16.038	-22.483	8.474	1.00	15.00	A	C
ATOM	1253	CD	LYS	A	174	17.084	-23.524	8.855	1.00	15.00	A	C
ATOM	1254	CE	LYS	A	174	18.482	-23.078	8.457	1.00	15.00	A	C
ATOM	1255	NZ	LYS	A	174	19.165	-24.065	7.574	1.00	15.00	A	N
ATOM	1256	C	LYS	A	174	12.180	-22.589	9.000	1.00	15.00	A	C
ATOM	1257	O	LYS	A	174	11.619	-23.525	8.435	1.00	15.00	A	O
ATOM	1258	N	GLN	A	175	11.677	-22.000	10.072	1.00	15.00	A	N
ATOM	1259	CA	GLN	A	175	10.419	-22.451	10.660	1.00	15.00	A	C
ATOM	1260	CB	GLN	A	175	10.175	-21.755	11.996	1.00	15.00	A	C
ATOM	1261	CG	GLN	A	175	11.249	-21.986	13.039	1.00	15.00	A	C
ATOM	1262	CD	GLN	A	175	11.027	-21.116	14.252	1.00	15.00	A	C
ATOM	1263	OE1	GLN	A	175	9.890	-20.808	14.605	1.00	15.00	A	O
ATOM	1264	NE2	GLN	A	175	12.104	-20.709	14.889	1.00	15.00	A	N
ATOM	1265	C	GLN	A	175	9.237	-22.170	9.738	1.00	15.00	A	C
ATOM	1266	O	GLN	A	175	8.204	-22.837	9.813	1.00	15.00	A	O
ATOM	1267	N	LEU	A	176	9.409	-21.193	8.855	1.00	15.00	A	N
ATOM	1268	CA	LEU	A	176	8.355	-20.786	7.941	1.00	15.00	A	C
ATOM	1269	CB	LEU	A	176	8.485	-19.293	7.633	1.00	15.00	A	C
ATOM	1270	CG	LEU	A	176	8.266	-18.354	8.821	1.00	15.00	A	C
ATOM	1271	CD1	LEU	A	176	8.701	-16.937	8.480	1.00	15.00	A	C

```
ATOM   1272  CD2 LEU A 176       6.811 -18.383   9.262  1.00 15.00       A    C
ATOM   1273  C   LEU A 176       8.330 -21.605   6.649  1.00 15.00       A    C
ATOM   1274  O   LEU A 176       7.370 -21.536   5.884  1.00 15.00       A    O
ATOM   1275  N   ARG A 177       9.376 -22.389   6.412  1.00 15.00       A    N
ATOM   1276  CA  ARG A 177       9.457 -23.207   5.190  1.00 15.00       A    C
ATOM   1277  CB  ARG A 177      10.850 -23.836   4.978  1.00 15.00       A    C
ATOM   1278  CG  ARG A 177      10.878 -25.016   4.003  1.00 15.00       A    C
ATOM   1279  CD  ARG A 177      10.554 -24.580   2.581  1.00 15.00       A    C
ATOM   1280  NE  ARG A 177      11.686 -23.966   1.928  1.00 15.00       A    N
ATOM   1281  CZ  ARG A 177      12.217 -24.405   0.783  1.00 15.00       A    C
ATOM   1282  NH1 ARG A 177      11.709 -25.454   0.146  1.00 15.00       A    N
ATOM   1283  NH2 ARG A 177      13.216 -23.731   0.231  1.00 15.00       A    N
ATOM   1284  C   ARG A 177       8.336 -24.254   5.080  1.00 15.00       A    C
ATOM   1285  O   ARG A 177       7.570 -24.219   4.122  1.00 15.00       A    O
ATOM   1286  N   PRO A 178       8.219 -25.201   6.038  1.00 15.00       A    N
ATOM   1287  CA  PRO A 178       7.180 -26.231   5.987  1.00 15.00       A    C
ATOM   1288  CB  PRO A 178       7.357 -27.025   7.288  1.00 15.00       A    C
ATOM   1289  CG  PRO A 178       8.274 -26.206   8.141  1.00 15.00       A    C
ATOM   1290  CD  PRO A 178       9.089 -25.355   7.214  1.00 15.00       A    C
ATOM   1291  C   PRO A 178       5.786 -25.626   5.912  1.00 15.00       A    C
ATOM   1292  O   PRO A 178       4.906 -26.148   5.229  1.00 15.00       A    O
ATOM   1293  N   LEU A 179       5.609 -24.505   6.594  1.00 15.00       A    N
ATOM   1294  CA  LEU A 179       4.330 -23.818   6.623  1.00 15.00       A    C
ATOM   1295  CB  LEU A 179       4.327 -22.744   7.710  1.00 15.00       A    C
ATOM   1296  CG  LEU A 179       4.445 -23.250   9.149  1.00 15.00       A    C
ATOM   1297  CD1 LEU A 179       4.538 -22.080  10.118  1.00 15.00       A    C
ATOM   1298  CD2 LEU A 179       3.266 -24.147   9.506  1.00 15.00       A    C
ATOM   1299  C   LEU A 179       3.996 -23.196   5.274  1.00 15.00       A    C
ATOM   1300  O   LEU A 179       2.901 -23.387   4.746  1.00 15.00       A    O
ATOM   1301  N   TYR A 180       4.952 -22.475   4.705  1.00 15.00       A    N
ATOM   1302  CA  TYR A 180       4.749 -21.811   3.422  1.00 15.00       A    C
ATOM   1303  CB  TYR A 180       5.963 -20.954   3.052  1.00 15.00       A    C
ATOM   1304  CG  TYR A 180       5.649 -19.854   2.062  1.00 15.00       A    C
ATOM   1305  CD1 TYR A 180       5.060 -18.666   2.479  1.00 15.00       A    C
ATOM   1306  CD2 TYR A 180       5.932 -20.005   0.711  1.00 15.00       A    C
ATOM   1307  CE1 TYR A 180       4.762 -17.662   1.578  1.00 15.00       A    C
ATOM   1308  CE2 TYR A 180       5.639 -19.005  -0.197  1.00 15.00       A    C
ATOM   1309  CZ  TYR A 180       5.054 -17.835   0.241  1.00 15.00       A    C
ATOM   1310  OH  TYR A 180       4.756 -16.838  -0.662  1.00 15.00       A    O
ATOM   1311  C   TYR A 180       4.444 -22.815   2.313  1.00 15.00       A    C
ATOM   1312  O   TYR A 180       3.648 -22.537   1.416  1.00 15.00       A    O
ATOM   1313  N   GLU A 181       5.055 -23.989   2.402  1.00 15.00       A    N
ATOM   1314  CA  GLU A 181       4.856 -25.029   1.405  1.00 15.00       A    C
ATOM   1315  CB  GLU A 181       5.828 -26.195   1.610  1.00 15.00       A    C
ATOM   1316  CG  GLU A 181       7.280 -25.838   1.314  1.00 15.00       A    C
ATOM   1317  CD  GLU A 181       8.208 -27.037   1.311  1.00 15.00       A    C
ATOM   1318  OE1 GLU A 181       7.792 -28.114   1.789  1.00 15.00       A    O
ATOM   1319  OE2 GLU A 181       9.348 -26.914   0.810  1.00 15.00       A    O
ATOM   1320  C   GLU A 181       3.407 -25.508   1.356  1.00 15.00       A    C
ATOM   1321  O   GLU A 181       2.800 -25.551   0.284  1.00 15.00       A    O
ATOM   1322  N   GLU A 182       2.843 -25.847   2.513  1.00 15.00       A    N
ATOM   1323  CA  GLU A 182       1.456 -26.302   2.562  1.00 15.00       A    C
ATOM   1324  CB  GLU A 182       1.105 -26.922   3.915  1.00 15.00       A    C
ATOM   1325  CG  GLU A 182      -0.115 -27.830   3.858  1.00 15.00       A    C
ATOM   1326  CD  GLU A 182      -0.523 -28.376   5.211  1.00 15.00       A    C
ATOM   1327  OE1 GLU A 182       0.363 -28.804   5.976  1.00 15.00       A    O
ATOM   1328  OE2 GLU A 182      -1.737 -28.388   5.499  1.00 15.00       A    O
ATOM   1329  C   GLU A 182       0.520 -25.142   2.242  1.00 15.00       A    C
ATOM   1330  O   GLU A 182      -0.541 -25.323   1.639  1.00 15.00       A    O
ATOM   1331  N   TYR A 183       0.940 -23.942   2.635  1.00 15.00       A    N
ATOM   1332  CA  TYR A 183       0.176 -22.731   2.379  1.00 15.00       A    C
ATOM   1333  CB  TYR A 183       0.895 -21.519   2.996  1.00 15.00       A    C
ATOM   1334  CG  TYR A 183       0.693 -20.202   2.273  1.00 15.00       A    C
ATOM   1335  CD1 TYR A 183       1.626 -19.746   1.349  1.00 15.00       A    C
ATOM   1336  CD2 TYR A 183      -0.424 -19.414   2.515  1.00 15.00       A    C
ATOM   1337  CE1 TYR A 183       1.452 -18.547   0.686  1.00 15.00       A    C
ATOM   1338  CE2 TYR A 183      -0.606 -18.212   1.856  1.00 15.00       A    C
ATOM   1339  CZ  TYR A 183       0.335 -17.784   0.943  1.00 15.00       A    C
ATOM   1340  OH  TYR A 183       0.156 -16.592   0.279  1.00 15.00       A    O
ATOM   1341  C   TYR A 183      -0.027 -22.546   0.876  1.00 15.00       A    C
ATOM   1342  O   TYR A 183      -1.127 -22.234   0.424  1.00 15.00       A    O
```

ATOM	1343	N	VAL	A	184	1.039	-22.767	0.110	1.00	15.00	A	N
ATOM	1344	CA	VAL	A	184	0.988	-22.629	-1.343	1.00	15.00	A	C
ATOM	1345	CB	VAL	A	184	2.389	-22.795	-1.979	1.00	15.00	A	C
ATOM	1346	CG1	VAL	A	184	2.300	-22.897	-3.497	1.00	15.00	A	C
ATOM	1347	CG2	VAL	A	184	3.292	-21.638	-1.584	1.00	15.00	A	C
ATOM	1348	C	VAL	A	184	0.023	-23.643	-1.952	1.00	15.00	A	C
ATOM	1349	O	VAL	A	184	-0.751	-23.313	-2.852	1.00	15.00	A	O
ATOM	1350	N	VAL	A	185	0.056	-24.866	-1.439	1.00	15.00	A	N
ATOM	1351	CA	VAL	A	185	-0.806	-25.930	-1.937	1.00	15.00	A	C
ATOM	1352	CB	VAL	A	185	-0.449	-27.295	-1.308	1.00	15.00	A	C
ATOM	1353	CG1	VAL	A	185	-1.371	-28.391	-1.825	1.00	15.00	A	C
ATOM	1354	CG2	VAL	A	185	1.005	-27.647	-1.587	1.00	15.00	A	C
ATOM	1355	C	VAL	A	185	-2.279	-25.618	-1.686	1.00	15.00	A	C
ATOM	1356	O	VAL	A	185	-3.081	-25.582	-2.621	1.00	15.00	A	O
ATOM	1357	N	LEU	A	186	-2.620	-25.361	-0.430	1.00	15.00	A	N
ATOM	1358	CA	LEU	A	186	-3.998	-25.070	-0.053	1.00	15.00	A	C
ATOM	1359	CB	LEU	A	186	-4.141	-24.985	1.467	1.00	15.00	A	C
ATOM	1360	CG	LEU	A	186	-3.785	-26.259	2.238	1.00	15.00	A	C
ATOM	1361	CD1	LEU	A	186	-3.892	-26.030	3.736	1.00	15.00	A	C
ATOM	1362	CD2	LEU	A	186	-4.672	-27.418	1.807	1.00	15.00	A	C
ATOM	1363	C	LEU	A	186	-4.533	-23.809	-0.731	1.00	15.00	A	C
ATOM	1364	O	LEU	A	186	-5.693	-23.763	-1.141	1.00	15.00	A	O
ATOM	1365	N	LYS	A	187	-3.688	-22.791	-0.858	1.00	15.00	A	N
ATOM	1366	CA	LYS	A	187	-4.098	-21.549	-1.504	1.00	15.00	A	C
ATOM	1367	CB	LYS	A	187	-3.071	-20.442	-1.268	1.00	15.00	A	C
ATOM	1368	CG	LYS	A	187	-3.158	-19.800	0.106	1.00	15.00	A	C
ATOM	1369	CD	LYS	A	187	-4.399	-18.933	0.239	1.00	15.00	A	C
ATOM	1370	CE	LYS	A	187	-4.333	-18.066	1.485	1.00	15.00	A	C
ATOM	1371	NZ	LYS	A	187	-5.085	-16.797	1.316	1.00	15.00	A	N
ATOM	1372	C	LYS	A	187	-4.312	-21.765	-2.996	1.00	15.00	A	C
ATOM	1373	O	LYS	A	187	-5.197	-21.161	-3.602	1.00	15.00	A	O
ATOM	1374	N	ASN	A	188	-3.498	-22.637	-3.577	1.00	15.00	A	N
ATOM	1375	CA	ASN	A	188	-3.598	-22.956	-4.994	1.00	15.00	A	C
ATOM	1376	CB	ASN	A	188	-2.422	-23.823	-5.431	1.00	15.00	A	C
ATOM	1377	CG	ASN	A	188	-1.653	-23.220	-6.583	1.00	15.00	A	C
ATOM	1378	OD1	ASN	A	188	-2.223	-22.569	-7.457	1.00	15.00	A	O
ATOM	1379	ND2	ASN	A	188	-0.349	-23.427	-6.584	1.00	15.00	A	N
ATOM	1380	C	ASN	A	188	-4.900	-23.687	-5.270	1.00	15.00	A	C
ATOM	1381	O	ASN	A	188	-5.581	-23.414	-6.260	1.00	15.00	A	O
ATOM	1382	N	GLU	A	189	-5.238	-24.622	-4.383	1.00	15.00	A	N
ATOM	1383	CA	GLU	A	189	-6.475	-25.378	-4.504	1.00	15.00	A	C
ATOM	1384	CB	GLU	A	189	-6.609	-26.371	-3.350	1.00	15.00	A	C
ATOM	1385	CG	GLU	A	189	-5.653	-27.547	-3.415	1.00	15.00	A	C
ATOM	1386	CD	GLU	A	189	-6.064	-28.663	-2.481	1.00	15.00	A	C
ATOM	1387	OE1	GLU	A	189	-5.226	-29.104	-1.663	1.00	15.00	A	O
ATOM	1388	OE2	GLU	A	189	-7.234	-29.104	-2.549	1.00	15.00	A	O
ATOM	1389	C	GLU	A	189	-7.636	-24.404	-4.468	1.00	15.00	A	C
ATOM	1390	O	GLU	A	189	-8.567	-24.483	-5.273	1.00	15.00	A	O
ATOM	1391	N	MET	A	190	-7.549	-23.472	-3.527	1.00	15.00	A	N
ATOM	1392	CA	MET	A	190	-8.553	-22.432	-3.355	1.00	15.00	A	C
ATOM	1393	CB	MET	A	190	-8.137	-21.508	-2.203	1.00	15.00	A	C
ATOM	1394	CG	MET	A	190	-8.898	-20.196	-2.143	1.00	15.00	A	C
ATOM	1395	SD	MET	A	190	-7.845	-18.800	-1.691	1.00	15.00	A	S
ATOM	1396	CE	MET	A	190	-8.922	-17.438	-2.126	1.00	15.00	A	C
ATOM	1397	C	MET	A	190	-8.732	-21.624	-4.644	1.00	15.00	A	C
ATOM	1398	O	MET	A	190	-9.852	-21.409	-5.105	1.00	15.00	A	O
ATOM	1399	N	ALA	A	191	-7.613	-21.202	-5.226	1.00	15.00	A	N
ATOM	1400	CA	ALA	A	191	-7.619	-20.419	-6.455	1.00	15.00	A	C
ATOM	1401	CB	ALA	A	191	-6.212	-19.943	-6.778	1.00	15.00	A	C
ATOM	1402	C	ALA	A	191	-8.197	-21.217	-7.623	1.00	15.00	A	C
ATOM	1403	O	ALA	A	191	-9.018	-20.709	-8.386	1.00	15.00	A	O
ATOM	1404	N	ARG	A	192	-7.773	-22.475	-7.752	1.00	15.00	A	N
ATOM	1405	CA	ARG	A	192	-8.259	-23.346	-8.827	1.00	15.00	A	C
ATOM	1406	CB	ARG	A	192	-7.498	-24.682	-8.858	1.00	15.00	A	C
ATOM	1407	CG	ARG	A	192	-6.389	-24.743	-9.906	1.00	15.00	A	C
ATOM	1408	CD	ARG	A	192	-5.008	-24.917	-9.283	1.00	15.00	A	C
ATOM	1409	NE	ARG	A	192	-3.994	-24.133	-9.997	1.00	15.00	A	N
ATOM	1410	CZ	ARG	A	192	-2.678	-24.350	-9.929	1.00	15.00	A	C
ATOM	1411	NH1	ARG	A	192	-2.194	-25.325	-9.175	1.00	15.00	A	N
ATOM	1412	NH2	ARG	A	192	-1.854	-23.566	-10.594	1.00	15.00	A	N
ATOM	1413	C	ARG	A	192	-9.765	-23.570	-8.711	1.00	15.00	A	C

```
ATOM   1414  O   ARG A 192     -10.491 -23.555  -9.708  1.00 15.00       A    O
ATOM   1415  N   ALA A 193     -10.231 -23.736  -7.479  1.00 15.00       A    N
ATOM   1416  CA  ALA A 193     -11.645 -23.946  -7.215  1.00 15.00       A    C
ATOM   1417  CB  ALA A 193     -11.848 -24.486  -5.805  1.00 15.00       A    C
ATOM   1418  C   ALA A 193     -12.441 -22.661  -7.447  1.00 15.00       A    C
ATOM   1419  O   ALA A 193     -13.664 -22.686  -7.541  1.00 15.00       A    O
ATOM   1420  N   ASN A 194     -11.730 -21.544  -7.552  1.00 15.00       A    N
ATOM   1421  CA  ASN A 194     -12.350 -20.250  -7.800  1.00 15.00       A    C
ATOM   1422  CB  ASN A 194     -11.639 -19.156  -6.987  1.00 15.00       A    C
ATOM   1423  CG  ASN A 194     -12.290 -18.867  -5.647  1.00 15.00       A    C
ATOM   1424  OD1 ASN A 194     -13.041 -17.904  -5.502  1.00 15.00       A    O
ATOM   1425  ND2 ASN A 194     -12.000 -19.693  -4.652  1.00 15.00       A    N
ATOM   1426  C   ASN A 194     -12.253 -19.924  -9.289  1.00 15.00       A    C
ATOM   1427  O   ASN A 194     -12.566 -18.810  -9.707  1.00 15.00       A    O
ATOM   1428  N   HIS A 195     -11.790 -20.908 -10.071  1.00 15.00       A    N
ATOM   1429  CA  HIS A 195     -11.646 -20.778 -11.528  1.00 15.00       A    C
ATOM   1430  CB  HIS A 195     -12.954 -20.333 -12.204  1.00 15.00       A    C
ATOM   1431  CG  HIS A 195     -14.095 -21.291 -12.021  1.00 15.00       A    C
ATOM   1432  ND1 HIS A 195     -15.364 -20.849 -11.725  1.00 15.00       A    N
ATOM   1433  CD2 HIS A 195     -14.104 -22.647 -12.092  1.00 15.00       A    C
ATOM   1434  CE1 HIS A 195     -16.111 -21.931 -11.622  1.00 15.00       A    C
ATOM   1435  NE2 HIS A 195     -15.393 -23.041 -11.836  1.00 15.00       A    N
ATOM   1436  C   HIS A 195     -10.458 -19.907 -11.963  1.00 15.00       A    C
ATOM   1437  O   HIS A 195     -10.500 -19.268 -13.016  1.00 15.00       A    O
ATOM   1438  N   TYR A 196      -9.395 -19.896 -11.168  1.00 15.00       A    N
ATOM   1439  CA  TYR A 196      -8.196 -19.121 -11.496  1.00 15.00       A    C
ATOM   1440  CB  TYR A 196      -7.765 -18.262 -10.306  1.00 15.00       A    C
ATOM   1441  CG  TYR A 196      -8.663 -17.070 -10.065  1.00 15.00       A    C
ATOM   1442  CD1 TYR A 196      -9.832 -17.189  -9.325  1.00 15.00       A    C
ATOM   1443  CD2 TYR A 196      -8.344 -15.824 -10.583  1.00 15.00       A    C
ATOM   1444  CE1 TYR A 196     -10.659 -16.102  -9.110  1.00 15.00       A    C
ATOM   1445  CE2 TYR A 196      -9.162 -14.731 -10.372  1.00 15.00       A    C
ATOM   1446  CZ  TYR A 196     -10.317 -14.875  -9.635  1.00 15.00       A    C
ATOM   1447  OH  TYR A 196     -11.135 -13.789  -9.425  1.00 15.00       A    O
ATOM   1448  C   TYR A 196      -7.061 -20.053 -11.921  1.00 15.00       A    C
ATOM   1449  O   TYR A 196      -7.090 -21.244 -11.607  1.00 15.00       A    O
ATOM   1450  N   GLU A 197      -6.061 -19.521 -12.624  1.00 15.00       A    N
ATOM   1451  CA  GLU A 197      -4.944 -20.350 -13.080  1.00 15.00       A    C
ATOM   1452  CB  GLU A 197      -4.241 -19.774 -14.321  1.00 15.00       A    C
ATOM   1453  CG  GLU A 197      -3.341 -18.578 -14.058  1.00 15.00       A    C
ATOM   1454  CD  GLU A 197      -2.407 -18.275 -15.215  1.00 15.00       A    C
ATOM   1455  OE1 GLU A 197      -2.543 -18.909 -16.281  1.00 15.00       A    O
ATOM   1456  OE2 GLU A 197      -1.522 -17.406 -15.054  1.00 15.00       A    O
ATOM   1457  C   GLU A 197      -3.958 -20.648 -11.949  1.00 15.00       A    C
ATOM   1458  O   GLU A 197      -3.486 -21.780 -11.809  1.00 15.00       A    O
ATOM   1459  N   ASP A 198      -3.655 -19.637 -11.141  1.00 15.00       A    N
ATOM   1460  CA  ASP A 198      -2.743 -19.797 -10.008  1.00 15.00       A    C
ATOM   1461  CB  ASP A 198      -1.270 -19.593 -10.416  1.00 15.00       A    C
ATOM   1462  CG  ASP A 198      -0.843 -18.142 -10.567  1.00 15.00       A    C
ATOM   1463  OD1 ASP A 198      -0.286 -17.584  -9.603  1.00 15.00       A    O
ATOM   1464  OD2 ASP A 198      -1.038 -17.565 -11.654  1.00 15.00       A    O
ATOM   1465  C   ASP A 198      -3.162 -18.874  -8.865  1.00 15.00       A    C
ATOM   1466  O   ASP A 198      -4.077 -18.067  -9.033  1.00 15.00       A    O
ATOM   1467  N   TYR A 199      -2.525 -18.997  -7.701  1.00 15.00       A    N
ATOM   1468  CA  TYR A 199      -2.886 -18.149  -6.563  1.00 15.00       A    C
ATOM   1469  CB  TYR A 199      -2.239 -18.604  -5.250  1.00 15.00       A    C
ATOM   1470  CG  TYR A 199      -2.709 -17.803  -4.050  1.00 15.00       A    C
ATOM   1471  CD1 TYR A 199      -4.060 -17.530  -3.860  1.00 15.00       A    C
ATOM   1472  CD2 TYR A 199      -1.808 -17.307  -3.118  1.00 15.00       A    C
ATOM   1473  CE1 TYR A 199      -4.496 -16.787  -2.781  1.00 15.00       A    C
ATOM   1474  CE2 TYR A 199      -2.236 -16.565  -2.033  1.00 15.00       A    C
ATOM   1475  CZ  TYR A 199      -3.580 -16.307  -1.871  1.00 15.00       A    C
ATOM   1476  OH  TYR A 199      -4.012 -15.568  -0.792  1.00 15.00       A    O
ATOM   1477  C   TYR A 199      -2.589 -16.682  -6.848  1.00 15.00       A    C
ATOM   1478  O   TYR A 199      -3.333 -15.793  -6.432  1.00 15.00       A    O
ATOM   1479  N   GLY A 200      -1.512 -16.434  -7.576  1.00 15.00       A    N
ATOM   1480  CA  GLY A 200      -1.155 -15.081  -7.927  1.00 15.00       A    C
ATOM   1481  C   GLY A 200      -2.178 -14.508  -8.879  1.00 15.00       A    C
ATOM   1482  O   GLY A 200      -2.438 -13.309  -8.873  1.00 15.00       A    O
ATOM   1483  N   ASP A 201      -2.767 -15.385  -9.691  1.00 15.00       A    N
ATOM   1484  CA  ASP A 201      -3.797 -14.988 -10.648  1.00 15.00       A    C
```

```
ATOM   1485  CB  ASP A 201      -4.245 -16.191 -11.482  1.00 15.00      A    C
ATOM   1486  CG  ASP A 201      -5.158 -15.814 -12.631  1.00 15.00      A    C
ATOM   1487  OD1 ASP A 201      -4.972 -14.723 -13.215  1.00 15.00      A    O
ATOM   1488  OD2 ASP A 201      -6.063 -16.614 -12.960  1.00 15.00      A    O
ATOM   1489  C   ASP A 201      -4.980 -14.407  -9.897  1.00 15.00      A    C
ATOM   1490  O   ASP A 201      -5.621 -13.460 -10.347  1.00 15.00      A    O
ATOM   1491  N   TYR A 202      -5.240 -14.977  -8.726  1.00 15.00      A    N
ATOM   1492  CA  TYR A 202      -6.322 -14.522  -7.869  1.00 15.00      A    C
ATOM   1493  CB  TYR A 202      -6.483 -15.480  -6.683  1.00 15.00      A    C
ATOM   1494  CG  TYR A 202      -7.590 -15.112  -5.723  1.00 15.00      A    C
ATOM   1495  CD1 TYR A 202      -7.304 -14.533  -4.493  1.00 15.00      A    C
ATOM   1496  CD2 TYR A 202      -8.921 -15.347  -6.043  1.00 15.00      A    C
ATOM   1497  CE1 TYR A 202      -8.311 -14.197  -3.610  1.00 15.00      A    C
ATOM   1498  CE2 TYR A 202      -9.936 -15.013  -5.166  1.00 15.00      A    C
ATOM   1499  CZ  TYR A 202      -9.626 -14.439  -3.951  1.00 15.00      A    C
ATOM   1500  OH  TYR A 202     -10.632 -14.109  -3.070  1.00 15.00      A    O
ATOM   1501  C   TYR A 202      -6.034 -13.104  -7.381  1.00 15.00      A    C
ATOM   1502  O   TYR A 202      -6.942 -12.289  -7.223  1.00 15.00      A    O
ATOM   1503  N   TRP A 203      -4.756 -12.811  -7.160  1.00 15.00      A    N
ATOM   1504  CA  TRP A 203      -4.338 -11.488  -6.709  1.00 15.00      A    C
ATOM   1505  CB  TRP A 203      -2.925 -11.538  -6.119  1.00 15.00      A    C
ATOM   1506  CG  TRP A 203      -2.860 -12.045  -4.709  1.00 15.00      A    C
ATOM   1507  CD1 TRP A 203      -3.891 -12.143  -3.820  1.00 15.00      A    C
ATOM   1508  CD2 TRP A 203      -1.693 -12.511  -4.022  1.00 15.00      A    C
ATOM   1509  NE1 TRP A 203      -3.438 -12.645  -2.624  1.00 15.00      A    N
ATOM   1510  CE2 TRP A 203      -2.092 -12.878  -2.722  1.00 15.00      A    C
ATOM   1511  CE3 TRP A 203      -0.350 -12.657  -4.381  1.00 15.00      A    C
ATOM   1512  CZ2 TRP A 203      -1.196 -13.378  -1.780  1.00 15.00      A    C
ATOM   1513  CZ3 TRP A 203       0.537 -13.153  -3.445  1.00 15.00      A    C
ATOM   1514  CH2 TRP A 203       0.110 -13.509  -2.161  1.00 15.00      A    C
ATOM   1515  C   TRP A 203      -4.375 -10.503  -7.873  1.00 15.00      A    C
ATOM   1516  O   TRP A 203      -4.591  -9.309  -7.691  1.00 15.00      A    O
ATOM   1517  N   ARG A 204      -4.185 -11.025  -9.079  1.00 15.00      A    N
ATOM   1518  CA  ARG A 204      -4.194 -10.206 -10.283  1.00 15.00      A    C
ATOM   1519  CB  ARG A 204      -3.431 -10.913 -11.420  1.00 15.00      A    C
ATOM   1520  CG  ARG A 204      -2.002 -11.307 -11.062  1.00 15.00      A    C
ATOM   1521  CD  ARG A 204      -1.218 -11.828 -12.265  1.00 15.00      A    C
ATOM   1522  NE  ARG A 204      -1.704 -13.122 -12.757  1.00 15.00      A    N
ATOM   1523  CZ  ARG A 204      -1.145 -14.305 -12.468  1.00 15.00      A    C
ATOM   1524  NH1 ARG A 204      -0.076 -14.380 -11.678  1.00 15.00      A    N
ATOM   1525  NH2 ARG A 204      -1.641 -15.413 -12.996  1.00 15.00      A    N
ATOM   1526  C   ARG A 204      -5.627  -9.897 -10.719  1.00 15.00      A    C
ATOM   1527  O   ARG A 204      -5.849  -9.227 -11.727  1.00 15.00      A    O
ATOM   1528  N   GLY A 205      -6.593 -10.373  -9.936  1.00 15.00      A    N
ATOM   1529  CA  GLY A 205      -7.995 -10.168 -10.256  1.00 15.00      A    C
ATOM   1530  C   GLY A 205      -8.531  -8.801  -9.877  1.00 15.00      A    C
ATOM   1531  O   GLY A 205      -9.548  -8.368 -10.413  1.00 15.00      A    O
ATOM   1532  N   ASP A 206      -7.852  -8.112  -8.967  1.00 15.00      A    N
ATOM   1533  CA  ASP A 206      -8.296  -6.785  -8.525  1.00 15.00      A    C
ATOM   1534  CB  ASP A 206      -7.491  -6.322  -7.306  1.00 15.00      A    C
ATOM   1535  CG  ASP A 206      -7.979  -4.997  -6.740  1.00 15.00      A    C
ATOM   1536  OD1 ASP A 206      -9.131  -4.942  -6.248  1.00 15.00      A    O
ATOM   1537  OD2 ASP A 206      -7.206  -4.015  -6.777  1.00 15.00      A    O
ATOM   1538  C   ASP A 206      -8.194  -5.758  -9.653  1.00 15.00      A    C
ATOM   1539  O   ASP A 206      -8.910  -4.758  -9.673  1.00 15.00      A    O
ATOM   1540  N   TYR A 207      -7.317  -6.031 -10.607  1.00 15.00      A    N
ATOM   1541  CA  TYR A 207      -7.114  -5.136 -11.736  1.00 15.00      A    C
ATOM   1542  CB  TYR A 207      -5.626  -5.070 -12.101  1.00 15.00      A    C
ATOM   1543  CG  TYR A 207      -4.700  -4.660 -10.973  1.00 15.00      A    C
ATOM   1544  CD1 TYR A 207      -4.288  -5.577 -10.013  1.00 15.00      A    C
ATOM   1545  CD2 TYR A 207      -4.223  -3.359 -10.879  1.00 15.00      A    C
ATOM   1546  CE1 TYR A 207      -3.429  -5.211  -8.992  1.00 15.00      A    C
ATOM   1547  CE2 TYR A 207      -3.365  -2.983  -9.861  1.00 15.00      A    C
ATOM   1548  CZ  TYR A 207      -2.971  -3.912  -8.920  1.00 15.00      A    C
ATOM   1549  OH  TYR A 207      -2.108  -3.541  -7.910  1.00 15.00      A    O
ATOM   1550  C   TYR A 207      -7.903  -5.610 -12.953  1.00 15.00      A    C
ATOM   1551  O   TYR A 207      -7.847  -4.991 -14.017  1.00 15.00      A    O
ATOM   1552  N   GLU A 208      -8.648  -6.696 -12.794  1.00 15.00      A    N
ATOM   1553  CA  GLU A 208      -9.414  -7.258 -13.897  1.00 15.00      A    C
ATOM   1554  CB  GLU A 208      -9.721  -8.736 -13.664  1.00 15.00      A    C
ATOM   1555  CG  GLU A 208     -10.395  -9.405 -14.849  1.00 15.00      A    C
```

ATOM	1556	CD	GLU	A	208	-10.538	-10.902	-14.688	1.00	15.00	A	C
ATOM	1557	OE1	GLU	A	208	-10.428	-11.398	-13.549	1.00	15.00	A	O
ATOM	1558	OE2	GLU	A	208	-10.757	-11.587	-15.707	1.00	15.00	A	O
ATOM	1559	C	GLU	A	208	-10.695	-6.480	-14.174	1.00	15.00	A	C
ATOM	1560	O	GLU	A	208	-11.441	-6.126	-13.260	1.00	15.00	A	O
ATOM	1561	N	VAL	A	209	-10.938	-6.222	-15.451	1.00	15.00	A	N
ATOM	1562	CA	VAL	A	209	-12.119	-5.497	-15.892	1.00	15.00	A	C
ATOM	1563	CB	VAL	A	209	-11.769	-4.049	-16.309	1.00	15.00	A	C
ATOM	1564	CG1	VAL	A	209	-13.017	-3.298	-16.740	1.00	15.00	A	C
ATOM	1565	CG2	VAL	A	209	-11.061	-3.307	-15.185	1.00	15.00	A	C
ATOM	1566	C	VAL	A	209	-12.726	-6.219	-17.090	1.00	15.00	A	C
ATOM	1567	O	VAL	A	209	-12.062	-6.381	-18.112	1.00	15.00	A	O
ATOM	1568	N	ASN	A	210	-13.970	-6.669	-16.954	1.00	15.00	A	N
ATOM	1569	CA	ASN	A	210	-14.649	-7.378	-18.038	1.00	15.00	A	C
ATOM	1570	CB	ASN	A	210	-14.754	-8.879	-17.737	1.00	15.00	A	C
ATOM	1571	CG	ASN	A	210	-13.435	-9.618	-17.791	1.00	15.00	A	C
ATOM	1572	OD1	ASN	A	210	-12.890	-9.866	-18.864	1.00	15.00	A	O
ATOM	1573	ND2	ASN	A	210	-12.932	-10.002	-16.631	1.00	15.00	A	N
ATOM	1574	C	ASN	A	210	-16.056	-6.841	-18.263	1.00	15.00	A	C
ATOM	1575	O	ASN	A	210	-16.953	-7.076	-17.447	1.00	15.00	A	O
ATOM	1576	N	GLY	A	211	-16.251	-6.113	-19.352	1.00	15.00	A	N
ATOM	1577	CA	GLY	A	211	-17.571	-5.600	-19.663	1.00	15.00	A	C
ATOM	1578	C	GLY	A	211	-17.599	-4.115	-19.950	1.00	15.00	A	C
ATOM	1579	O	GLY	A	211	-18.663	-3.493	-19.910	1.00	15.00	A	O
ATOM	1580	N	VAL	A	212	-16.443	-3.534	-20.228	1.00	15.00	A	N
ATOM	1581	CA	VAL	A	212	-16.362	-2.112	-20.522	1.00	15.00	A	C
ATOM	1582	CB	VAL	A	212	-15.725	-1.308	-19.365	1.00	15.00	A	C
ATOM	1583	CG1	VAL	A	212	-16.180	0.143	-19.414	1.00	15.00	A	C
ATOM	1584	CG2	VAL	A	212	-16.066	-1.929	-18.018	1.00	15.00	A	C
ATOM	1585	C	VAL	A	212	-15.569	-1.891	-21.804	1.00	15.00	A	C
ATOM	1586	O	VAL	A	212	-14.338	-1.882	-21.787	1.00	15.00	A	O
ATOM	1587	N	ASP	A	213	-16.293	-1.722	-22.906	1.00	15.00	A	N
ATOM	1588	CA	ASP	A	213	-15.697	-1.521	-24.228	1.00	15.00	A	C
ATOM	1589	CB	ASP	A	213	-16.771	-1.149	-25.253	1.00	15.00	A	C
ATOM	1590	CG	ASP	A	213	-17.134	-2.300	-26.169	1.00	15.00	A	C
ATOM	1591	OD1	ASP	A	213	-16.429	-2.499	-27.181	1.00	15.00	A	O
ATOM	1592	OD2	ASP	A	213	-18.129	-2.998	-25.878	1.00	15.00	A	O
ATOM	1593	C	ASP	A	213	-14.593	-0.470	-24.233	1.00	15.00	A	C
ATOM	1594	O	ASP	A	213	-14.839	0.718	-24.009	1.00	15.00	A	O
ATOM	1595	N	GLY	A	214	-13.368	-0.921	-24.475	1.00	15.00	A	N
ATOM	1596	CA	GLY	A	214	-12.235	-0.018	-24.531	1.00	15.00	A	C
ATOM	1597	C	GLY	A	214	-11.707	0.368	-23.165	1.00	15.00	A	C
ATOM	1598	O	GLY	A	214	-10.973	1.353	-23.036	1.00	15.00	A	O
ATOM	1599	N	TYR	A	215	-12.100	-0.382	-22.145	1.00	15.00	A	N
ATOM	1600	CA	TYR	A	215	-11.653	-0.129	-20.778	1.00	15.00	A	C
ATOM	1601	CB	TYR	A	215	-12.696	0.686	-20.005	1.00	15.00	A	C
ATOM	1602	CG	TYR	A	215	-12.798	2.130	-20.436	1.00	15.00	A	C
ATOM	1603	CD1	TYR	A	215	-11.998	3.104	-19.856	1.00	15.00	A	C
ATOM	1604	CD2	TYR	A	215	-13.690	2.519	-21.427	1.00	15.00	A	C
ATOM	1605	CE1	TYR	A	215	-12.081	4.424	-20.253	1.00	15.00	A	C
ATOM	1606	CE2	TYR	A	215	-13.781	3.836	-21.829	1.00	15.00	A	C
ATOM	1607	CZ	TYR	A	215	-12.975	4.785	-21.239	1.00	15.00	A	C
ATOM	1608	OH	TYR	A	215	-13.054	6.095	-21.649	1.00	15.00	A	O
ATOM	1609	C	TYR	A	215	-11.375	-1.437	-20.045	1.00	15.00	A	C
ATOM	1610	O	TYR	A	215	-11.010	-1.430	-18.867	1.00	15.00	A	O
ATOM	1611	N	ASP	A	216	-11.553	-2.551	-20.747	1.00	15.00	A	N
ATOM	1612	CA	ASP	A	216	-11.328	-3.873	-20.170	1.00	15.00	A	C
ATOM	1613	CB	ASP	A	216	-11.827	-4.977	-21.110	1.00	15.00	A	C
ATOM	1614	CG	ASP	A	216	-13.329	-4.955	-21.317	1.00	15.00	A	C
ATOM	1615	OD1	ASP	A	216	-14.077	-4.938	-20.315	1.00	15.00	A	O
ATOM	1616	OD2	ASP	A	216	-13.763	-4.956	-22.484	1.00	15.00	A	O
ATOM	1617	C	ASP	A	216	-9.855	-4.095	-19.853	1.00	15.00	A	C
ATOM	1618	O	ASP	A	216	-8.976	-3.454	-20.434	1.00	15.00	A	O
ATOM	1619	N	TYR	A	217	-9.592	-5.015	-18.938	1.00	15.00	A	N
ATOM	1620	CA	TYR	A	217	-8.232	-5.332	-18.530	1.00	15.00	A	C
ATOM	1621	CB	TYR	A	217	-7.777	-4.376	-17.421	1.00	15.00	A	C
ATOM	1622	CG	TYR	A	217	-6.279	-4.336	-17.203	1.00	15.00	A	C
ATOM	1623	CD1	TYR	A	217	-5.681	-5.092	-16.203	1.00	15.00	A	C
ATOM	1624	CD2	TYR	A	217	-5.464	-3.533	-17.991	1.00	15.00	A	C
ATOM	1625	CE1	TYR	A	217	-4.317	-5.051	-15.993	1.00	15.00	A	C
ATOM	1626	CE2	TYR	A	217	-4.097	-3.488	-17.789	1.00	15.00	A	C

ATOM	1627	CZ	TYR	A	217	-3.529	-4.248	-16.787	1.00	15.00	A	C
ATOM	1628	OH	TYR	A	217	-2.171	-4.208	-16.580	1.00	15.00	A	O
ATOM	1629	C	TYR	A	217	-8.169	-6.772	-18.040	1.00	15.00	A	C
ATOM	1630	O	TYR	A	217	-8.978	-7.185	-17.212	1.00	15.00	A	O
ATOM	1631	N	SER	A	218	-7.217	-7.534	-18.555	1.00	15.00	A	N
ATOM	1632	CA	SER	A	218	-7.073	-8.925	-18.166	1.00	15.00	A	C
ATOM	1633	CB	SER	A	218	-6.683	-9.776	-19.379	1.00	15.00	A	C
ATOM	1634	OG	SER	A	218	-5.416	-9.390	-19.885	1.00	15.00	A	O
ATOM	1635	C	SER	A	218	-6.032	-9.072	-17.061	1.00	15.00	A	C
ATOM	1636	O	SER	A	218	-5.132	-8.241	-16.929	1.00	15.00	A	O
ATOM	1637	N	ARG	A	219	-6.155	-10.134	-16.275	1.00	15.00	A	N
ATOM	1638	CA	ARG	A	219	-5.222	-10.396	-15.184	1.00	15.00	A	C
ATOM	1639	CB	ARG	A	219	-5.748	-11.522	-14.295	1.00	15.00	A	C
ATOM	1640	CG	ARG	A	219	-7.191	-11.324	-13.868	1.00	15.00	A	C
ATOM	1641	CD	ARG	A	219	-7.612	-12.324	-12.808	1.00	15.00	A	C
ATOM	1642	NE	ARG	A	219	-7.482	-13.705	-13.258	1.00	15.00	A	N
ATOM	1643	CZ	ARG	A	219	-8.470	-14.416	-13.800	1.00	15.00	A	C
ATOM	1644	NH1	ARG	A	219	-9.670	-13.877	-13.984	1.00	15.00	A	N
ATOM	1645	NH2	ARG	A	219	-8.259	-15.683	-14.123	1.00	15.00	A	N
ATOM	1646	C	ARG	A	219	-3.836	-10.743	-15.720	1.00	15.00	A	C
ATOM	1647	O	ARG	A	219	-2.835	-10.625	-15.012	1.00	15.00	A	O
ATOM	1648	N	GLY	A	220	-3.788	-11.165	-16.978	1.00	15.00	A	N
ATOM	1649	CA	GLY	A	220	-2.526	-11.513	-17.600	1.00	15.00	A	C
ATOM	1650	C	GLY	A	220	-1.761	-10.283	-18.041	1.00	15.00	A	C
ATOM	1651	O	GLY	A	220	-0.538	-10.230	-17.910	1.00	15.00	A	O
ATOM	1652	N	GLN	A	221	-2.495	-9.284	-18.531	1.00	15.00	A	N
ATOM	1653	CA	GLN	A	221	-1.901	-8.032	-19.000	1.00	15.00	A	C
ATOM	1654	CB	GLN	A	221	-2.999	-7.080	-19.490	1.00	15.00	A	C
ATOM	1655	CG	GLN	A	221	-2.484	-5.759	-20.042	1.00	15.00	A	C
ATOM	1656	CD	GLN	A	221	-2.025	-5.857	-21.482	1.00	15.00	A	C
ATOM	1657	OE1	GLN	A	221	-2.610	-6.584	-22.285	1.00	15.00	A	O
ATOM	1658	NE2	GLN	A	221	-0.976	-5.125	-21.819	1.00	15.00	A	N
ATOM	1659	C	GLN	A	221	-1.090	-7.363	-17.894	1.00	15.00	A	C
ATOM	1660	O	GLN	A	221	-0.076	-6.718	-18.159	1.00	15.00	A	O
ATOM	1661	N	LEU	A	222	-1.534	-7.543	-16.652	1.00	15.00	A	N
ATOM	1662	CA	LEU	A	222	-0.855	-6.963	-15.499	1.00	15.00	A	C
ATOM	1663	CB	LEU	A	222	-1.561	-7.355	-14.197	1.00	15.00	A	C
ATOM	1664	CG	LEU	A	222	-0.940	-6.811	-12.906	1.00	15.00	A	C
ATOM	1665	CD1	LEU	A	222	-1.128	-5.305	-12.807	1.00	15.00	A	C
ATOM	1666	CD2	LEU	A	222	-1.520	-7.513	-11.689	1.00	15.00	A	C
ATOM	1667	C	LEU	A	222	0.606	-7.394	-15.451	1.00	15.00	A	C
ATOM	1668	O	LEU	A	222	1.479	-6.620	-15.066	1.00	15.00	A	O
ATOM	1669	N	ILE	A	223	0.868	-8.627	-15.859	1.00	15.00	A	N
ATOM	1670	CA	ILE	A	223	2.225	-9.151	-15.861	1.00	15.00	A	C
ATOM	1671	CB	ILE	A	223	2.245	-10.673	-16.124	1.00	15.00	A	C
ATOM	1672	CG1	ILE	A	223	1.278	-11.393	-15.179	1.00	15.00	A	C
ATOM	1673	CG2	ILE	A	223	3.656	-11.223	-15.965	1.00	15.00	A	C
ATOM	1674	CD1	ILE	A	223	1.041	-12.846	-15.532	1.00	15.00	A	C
ATOM	1675	C	ILE	A	223	3.044	-8.447	-16.935	1.00	15.00	A	C
ATOM	1676	O	ILE	A	223	4.108	-7.896	-16.656	1.00	15.00	A	O
ATOM	1677	N	GLU	A	224	2.510	-8.438	-18.150	1.00	15.00	A	N
ATOM	1678	CA	GLU	A	224	3.174	-7.815	-19.286	1.00	15.00	A	C
ATOM	1679	CB	GLU	A	224	2.336	-7.997	-20.552	1.00	15.00	A	C
ATOM	1680	CG	GLU	A	224	2.059	-9.454	-20.890	1.00	15.00	A	C
ATOM	1681	CD	GLU	A	224	1.141	-9.621	-22.082	1.00	15.00	A	C
ATOM	1682	OE1	GLU	A	224	-0.078	-9.805	-21.870	1.00	15.00	A	O
ATOM	1683	OE2	GLU	A	224	1.642	-9.576	-23.228	1.00	15.00	A	O
ATOM	1684	C	GLU	A	224	3.459	-6.337	-19.031	1.00	15.00	A	C
ATOM	1685	O	GLU	A	224	4.598	-5.892	-19.159	1.00	15.00	A	O
ATOM	1686	N	ASP	A	225	2.431	-5.592	-18.640	1.00	15.00	A	N
ATOM	1687	CA	ASP	A	225	2.570	-4.162	-18.367	1.00	15.00	A	C
ATOM	1688	CB	ASP	A	225	1.244	-3.566	-17.888	1.00	15.00	A	C
ATOM	1689	CG	ASP	A	225	0.204	-3.459	-18.986	1.00	15.00	A	C
ATOM	1690	OD1	ASP	A	225	0.560	-3.602	-20.170	1.00	15.00	A	O
ATOM	1691	OD2	ASP	A	225	-0.978	-3.227	-18.664	1.00	15.00	A	O
ATOM	1692	C	ASP	A	225	3.655	-3.891	-17.332	1.00	15.00	A	C
ATOM	1693	O	ASP	A	225	4.491	-3.001	-17.507	1.00	15.00	A	O
ATOM	1694	N	VAL	A	226	3.646	-4.673	-16.258	1.00	15.00	A	N
ATOM	1695	CA	VAL	A	226	4.627	-4.521	-15.189	1.00	15.00	A	C
ATOM	1696	CB	VAL	A	226	4.295	-5.418	-13.971	1.00	15.00	A	C
ATOM	1697	CG1	VAL	A	226	5.500	-5.579	-13.054	1.00	15.00	A	C

ATOM	1698	CG2	VAL	A	226	3.122	-4.838	-13.195	1.00	15.00	A	C
ATOM	1699	C	VAL	A	226	6.044	-4.803	-15.685	1.00	15.00	A	C
ATOM	1700	O	VAL	A	226	6.954	-4.005	-15.462	1.00	15.00	A	O
ATOM	1701	N	GLU	A	227	6.221	-5.919	-16.381	1.00	15.00	A	N
ATOM	1702	CA	GLU	A	227	7.534	-6.297	-16.888	1.00	15.00	A	C
ATOM	1703	CB	GLU	A	227	7.556	-7.763	-17.341	1.00	15.00	A	C
ATOM	1704	CG	GLU	A	227	7.040	-8.726	-16.276	1.00	15.00	A	C
ATOM	1705	CD	GLU	A	227	7.436	-10.174	-16.500	1.00	15.00	A	C
ATOM	1706	OE1	GLU	A	227	7.171	-10.711	-17.591	1.00	15.00	A	O
ATOM	1707	OE2	GLU	A	227	8.000	-10.785	-15.565	1.00	15.00	A	O
ATOM	1708	C	GLU	A	227	8.028	-5.341	-17.978	1.00	15.00	A	C
ATOM	1709	O	GLU	A	227	9.222	-5.061	-18.068	1.00	15.00	A	O
ATOM	1710	N	HIS	A	228	7.106	-4.823	-18.788	1.00	15.00	A	N
ATOM	1711	CA	HIS	A	228	7.466	-3.886	-19.854	1.00	15.00	A	C
ATOM	1712	CB	HIS	A	228	6.275	-3.606	-20.779	1.00	15.00	A	C
ATOM	1713	CG	HIS	A	228	5.904	-4.732	-21.694	1.00	15.00	A	C
ATOM	1714	ND1	HIS	A	228	4.835	-4.627	-22.552	1.00	15.00	A	N
ATOM	1715	CD2	HIS	A	228	6.465	-5.960	-21.830	1.00	15.00	A	C
ATOM	1716	CE1	HIS	A	228	4.762	-5.780	-23.185	1.00	15.00	A	C
ATOM	1717	NE2	HIS	A	228	5.726	-6.617	-22.781	1.00	15.00	A	N
ATOM	1718	C	HIS	A	228	7.963	-2.567	-19.275	1.00	15.00	A	C
ATOM	1719	O	HIS	A	228	9.038	-2.091	-19.628	1.00	15.00	A	O
ATOM	1720	N	THR	A	229	7.177	-1.992	-18.369	1.00	15.00	A	N
ATOM	1721	CA	THR	A	229	7.528	-0.720	-17.748	1.00	15.00	A	C
ATOM	1722	CB	THR	A	229	6.380	-0.166	-16.880	1.00	15.00	A	C
ATOM	1723	OG1	THR	A	229	5.841	-1.205	-16.049	1.00	15.00	A	O
ATOM	1724	CG2	THR	A	229	5.272	0.404	-17.754	1.00	15.00	A	C
ATOM	1725	C	THR	A	229	8.807	-0.826	-16.919	1.00	15.00	A	C
ATOM	1726	O	THR	A	229	9.550	0.147	-16.779	1.00	15.00	A	O
ATOM	1727	N	PHE	A	230	9.064	-2.016	-16.382	1.00	15.00	A	N
ATOM	1728	CA	PHE	A	230	10.252	-2.250	-15.572	1.00	15.00	A	C
ATOM	1729	CB	PHE	A	230	10.219	-3.652	-14.956	1.00	15.00	A	C
ATOM	1730	CG	PHE	A	230	11.318	-3.906	-13.964	1.00	15.00	A	C
ATOM	1731	CD1	PHE	A	230	11.292	-3.308	-12.714	1.00	15.00	A	C
ATOM	1732	CD2	PHE	A	230	12.376	-4.740	-14.282	1.00	15.00	A	C
ATOM	1733	CE1	PHE	A	230	12.302	-3.537	-11.801	1.00	15.00	A	C
ATOM	1734	CE2	PHE	A	230	13.390	-4.975	-13.373	1.00	15.00	A	C
ATOM	1735	CZ	PHE	A	230	13.353	-4.372	-12.131	1.00	15.00	A	C
ATOM	1736	C	PHE	A	230	11.521	-2.056	-16.399	1.00	15.00	A	C
ATOM	1737	O	PHE	A	230	12.553	-1.620	-15.881	1.00	15.00	A	O
ATOM	1738	N	GLU	A	231	11.431	-2.366	-17.689	1.00	15.00	A	N
ATOM	1739	CA	GLU	A	231	12.561	-2.219	-18.599	1.00	15.00	A	C
ATOM	1740	CB	GLU	A	231	12.208	-2.754	-19.991	1.00	15.00	A	C
ATOM	1741	CG	GLU	A	231	11.991	-4.257	-20.049	1.00	15.00	A	C
ATOM	1742	CD	GLU	A	231	13.144	-5.029	-19.444	1.00	15.00	A	C
ATOM	1743	OE1	GLU	A	231	14.278	-4.926	-19.957	1.00	15.00	A	O
ATOM	1744	OE2	GLU	A	231	12.934	-5.736	-18.438	1.00	15.00	A	O
ATOM	1745	C	GLU	A	231	12.984	-0.757	-18.703	1.00	15.00	A	C
ATOM	1746	O	GLU	A	231	14.164	-0.449	-18.860	1.00	15.00	A	O
ATOM	1747	N	GLU	A	232	12.012	0.139	-18.594	1.00	15.00	A	N
ATOM	1748	CA	GLU	A	232	12.270	1.569	-18.681	1.00	15.00	A	C
ATOM	1749	CB	GLU	A	232	10.986	2.321	-19.042	1.00	15.00	A	C
ATOM	1750	CG	GLU	A	232	10.428	1.959	-20.412	1.00	15.00	A	C
ATOM	1751	CD	GLU	A	232	8.993	2.409	-20.602	1.00	15.00	A	C
ATOM	1752	OE1	GLU	A	232	8.771	3.615	-20.845	1.00	15.00	A	O
ATOM	1753	OE2	GLU	A	232	8.084	1.557	-20.500	1.00	15.00	A	O
ATOM	1754	C	GLU	A	232	12.876	2.110	-17.384	1.00	15.00	A	C
ATOM	1755	O	GLU	A	232	13.564	3.131	-17.386	1.00	15.00	A	O
ATOM	1756	N	ILE	A	233	12.633	1.412	-16.279	1.00	15.00	A	N
ATOM	1757	CA	ILE	A	233	13.160	1.827	-14.981	1.00	15.00	A	C
ATOM	1758	CB	ILE	A	233	12.281	1.314	-13.818	1.00	15.00	A	C
ATOM	1759	CG1	ILE	A	233	10.805	1.608	-14.094	1.00	15.00	A	C
ATOM	1760	CG2	ILE	A	233	12.706	1.951	-12.501	1.00	15.00	A	C
ATOM	1761	CD1	ILE	A	233	9.855	0.929	-13.133	1.00	15.00	A	C
ATOM	1762	C	ILE	A	233	14.591	1.319	-14.794	1.00	15.00	A	C
ATOM	1763	O	ILE	A	233	15.362	1.873	-14.006	1.00	15.00	A	O
ATOM	1764	N	LYS	A	234	14.935	0.271	-15.537	1.00	15.00	A	N
ATOM	1765	CA	LYS	A	234	16.265	-0.340	-15.468	1.00	15.00	A	C
ATOM	1766	CB	LYS	A	234	16.433	-1.451	-16.505	1.00	15.00	A	C
ATOM	1767	CG	LYS	A	234	15.979	-2.818	-16.034	1.00	15.00	A	C
ATOM	1768	CD	LYS	A	234	16.096	-3.836	-17.151	1.00	15.00	A	C

ATOM	1769	CE	LYS	A	234	15.692	-5.220	-16.679	1.00	15.00	A	C
ATOM	1770	NZ	LYS	A	234	15.449	-6.136	-17.822	1.00	15.00	A	N
ATOM	1771	C	LYS	A	234	17.414	0.671	-15.561	1.00	15.00	A	C
ATOM	1772	O	LYS	A	234	18.223	0.749	-14.641	1.00	15.00	A	O
ATOM	1773	N	PRO	A	235	17.496	1.475	-16.649	1.00	15.00	A	N
ATOM	1774	CA	PRO	A	235	18.576	2.463	-16.819	1.00	15.00	A	C
ATOM	1775	CB	PRO	A	235	18.138	3.260	-18.051	1.00	15.00	A	C
ATOM	1776	CG	PRO	A	235	17.289	2.313	-18.822	1.00	15.00	A	C
ATOM	1777	CD	PRO	A	235	16.568	1.484	-17.798	1.00	15.00	A	C
ATOM	1778	C	PRO	A	235	18.715	3.393	-15.614	1.00	15.00	A	C
ATOM	1779	O	PRO	A	235	19.821	3.794	-15.248	1.00	15.00	A	O
ATOM	1780	N	LEU	A	236	17.587	3.725	-15.000	1.00	15.00	A	N
ATOM	1781	CA	LEU	A	236	17.578	4.603	-13.837	1.00	15.00	A	C
ATOM	1782	CB	LEU	A	236	16.160	5.127	-13.588	1.00	15.00	A	C
ATOM	1783	CG	LEU	A	236	15.949	5.971	-12.329	1.00	15.00	A	C
ATOM	1784	CD1	LEU	A	236	16.736	7.271	-12.404	1.00	15.00	A	C
ATOM	1785	CD2	LEU	A	236	14.468	6.252	-12.119	1.00	15.00	A	C
ATOM	1786	C	LEU	A	236	18.096	3.862	-12.607	1.00	15.00	A	C
ATOM	1787	O	LEU	A	236	18.911	4.386	-11.846	1.00	15.00	A	O
ATOM	1788	N	TYR	A	237	17.633	2.631	-12.433	1.00	15.00	A	N
ATOM	1789	CA	TYR	A	237	18.036	1.814	-11.298	1.00	15.00	A	C
ATOM	1790	CB	TYR	A	237	17.157	0.567	-11.183	1.00	15.00	A	C
ATOM	1791	CG	TYR	A	237	17.517	-0.321	-10.013	1.00	15.00	A	C
ATOM	1792	CD1	TYR	A	237	17.423	0.147	-8.709	1.00	15.00	A	C
ATOM	1793	CD2	TYR	A	237	17.953	-1.625	-10.213	1.00	15.00	A	C
ATOM	1794	CE1	TYR	A	237	17.754	-0.658	-7.638	1.00	15.00	A	C
ATOM	1795	CE2	TYR	A	237	18.286	-2.438	-9.147	1.00	15.00	A	C
ATOM	1796	CZ	TYR	A	237	18.184	-1.949	-7.861	1.00	15.00	A	C
ATOM	1797	OH	TYR	A	237	18.508	-2.755	-6.796	1.00	15.00	A	O
ATOM	1798	C	TYR	A	237	19.505	1.419	-11.388	1.00	15.00	A	C
ATOM	1799	O	TYR	A	237	20.225	1.466	-10.392	1.00	15.00	A	O
ATOM	1800	N	GLU	A	238	19.944	1.037	-12.583	1.00	15.00	A	N
ATOM	1801	CA	GLU	A	238	21.329	0.631	-12.804	1.00	15.00	A	C
ATOM	1802	CB	GLU	A	238	21.554	0.222	-14.260	1.00	15.00	A	C
ATOM	1803	CG	GLU	A	238	20.939	-1.119	-14.626	1.00	15.00	A	C
ATOM	1804	CD	GLU	A	238	21.330	-1.581	-16.013	1.00	15.00	A	C
ATOM	1805	OE1	GLU	A	238	22.546	-1.686	-16.282	1.00	15.00	A	O
ATOM	1806	OE2	GLU	A	238	20.421	-1.844	-16.830	1.00	15.00	A	O
ATOM	1807	C	GLU	A	238	22.304	1.730	-12.399	1.00	15.00	A	C
ATOM	1808	O	GLU	A	238	23.304	1.468	-11.731	1.00	15.00	A	O
ATOM	1809	N	HIS	A	239	22.000	2.964	-12.788	1.00	15.00	A	N
ATOM	1810	CA	HIS	A	239	22.857	4.093	-12.451	1.00	15.00	A	C
ATOM	1811	CB	HIS	A	239	22.508	5.330	-13.275	1.00	15.00	A	C
ATOM	1812	CG	HIS	A	239	23.191	5.349	-14.607	1.00	15.00	A	C
ATOM	1813	ND1	HIS	A	239	24.424	5.936	-14.770	1.00	15.00	A	N
ATOM	1814	CD2	HIS	A	239	22.792	4.816	-15.788	1.00	15.00	A	C
ATOM	1815	CE1	HIS	A	239	24.751	5.747	-16.035	1.00	15.00	A	C
ATOM	1816	NE2	HIS	A	239	23.794	5.073	-16.688	1.00	15.00	A	N
ATOM	1817	C	HIS	A	239	22.818	4.384	-10.955	1.00	15.00	A	C
ATOM	1818	O	HIS	A	239	23.829	4.760	-10.359	1.00	15.00	A	O
ATOM	1819	N	LEU	A	240	21.652	4.202	-10.350	1.00	15.00	A	N
ATOM	1820	CA	LEU	A	240	21.501	4.416	-8.920	1.00	15.00	A	C
ATOM	1821	CB	LEU	A	240	20.025	4.327	-8.518	1.00	15.00	A	C
ATOM	1822	CG	LEU	A	240	19.711	4.548	-7.035	1.00	15.00	A	C
ATOM	1823	CD1	LEU	A	240	19.964	5.995	-6.638	1.00	15.00	A	C
ATOM	1824	CD2	LEU	A	240	18.277	4.144	-6.726	1.00	15.00	A	C
ATOM	1825	C	LEU	A	240	22.314	3.366	-8.170	1.00	15.00	A	C
ATOM	1826	O	LEU	A	240	23.061	3.684	-7.245	1.00	15.00	A	O
ATOM	1827	N	HIS	A	241	22.174	2.117	-8.606	1.00	15.00	A	N
ATOM	1828	CA	HIS	A	241	22.887	0.989	-8.014	1.00	15.00	A	C
ATOM	1829	CB	HIS	A	241	22.511	-0.296	-8.768	1.00	15.00	A	C
ATOM	1830	CG	HIS	A	241	23.015	-1.571	-8.157	1.00	15.00	A	C
ATOM	1831	ND1	HIS	A	241	24.300	-2.015	-8.374	1.00	15.00	A	N
ATOM	1832	CD2	HIS	A	241	22.359	-2.471	-7.383	1.00	15.00	A	C
ATOM	1833	CE1	HIS	A	241	24.397	-3.166	-7.736	1.00	15.00	A	C
ATOM	1834	NE2	HIS	A	241	23.249	-3.482	-7.124	1.00	15.00	A	N
ATOM	1835	C	HIS	A	241	24.393	1.215	-8.097	1.00	15.00	A	C
ATOM	1836	O	HIS	A	241	25.116	1.027	-7.118	1.00	15.00	A	O
ATOM	1837	N	ALA	A	242	24.848	1.648	-9.269	1.00	15.00	A	N
ATOM	1838	CA	ALA	A	242	26.264	1.903	-9.503	1.00	15.00	A	C
ATOM	1839	CB	ALA	A	242	26.505	2.275	-10.957	1.00	15.00	A	C

```
ATOM   1840  C   ALA A 242      26.807   2.980  -8.569  1.00 15.00      A    C
ATOM   1841  O   ALA A 242      27.869   2.808  -7.965  1.00 15.00      A    O
ATOM   1842  N   TYR A 243      26.066   4.076  -8.431  1.00 15.00      A    N
ATOM   1843  CA  TYR A 243      26.487   5.169  -7.568  1.00 15.00      A    C
ATOM   1844  CB  TYR A 243      25.588   6.398  -7.737  1.00 15.00      A    C
ATOM   1845  CG  TYR A 243      25.980   7.560  -6.851  1.00 15.00      A    C
ATOM   1846  CD1 TYR A 243      27.084   8.351  -7.150  1.00 15.00      A    C
ATOM   1847  CD2 TYR A 243      25.252   7.860  -5.707  1.00 15.00      A    C
ATOM   1848  CE1 TYR A 243      27.449   9.405  -6.335  1.00 15.00      A    C
ATOM   1849  CE2 TYR A 243      25.609   8.914  -4.887  1.00 15.00      A    C
ATOM   1850  CZ  TYR A 243      26.708   9.683  -5.204  1.00 15.00      A    C
ATOM   1851  OH  TYR A 243      27.073  10.731  -4.385  1.00 15.00      A    O
ATOM   1852  C   TYR A 243      26.521   4.718  -6.113  1.00 15.00      A    C
ATOM   1853  O   TYR A 243      27.471   5.017  -5.384  1.00 15.00      A    O
ATOM   1854  N   VAL A 244      25.484   4.000  -5.691  1.00 15.00      A    N
ATOM   1855  CA  VAL A 244      25.420   3.493  -4.325  1.00 15.00      A    C
ATOM   1856  CB  VAL A 244      24.085   2.767  -4.031  1.00 15.00      A    C
ATOM   1857  CG1 VAL A 244      24.122   2.100  -2.665  1.00 15.00      A    C
ATOM   1858  CG2 VAL A 244      22.922   3.739  -4.097  1.00 15.00      A    C
ATOM   1859  C   VAL A 244      26.596   2.551  -4.080  1.00 15.00      A    C
ATOM   1860  O   VAL A 244      27.239   2.603  -3.027  1.00 15.00      A    O
ATOM   1861  N   ARG A 245      26.890   1.718  -5.079  1.00 15.00      A    N
ATOM   1862  CA  ARG A 245      27.999   0.784  -4.998  1.00 15.00      A    C
ATOM   1863  CB  ARG A 245      28.067  -0.100  -6.242  1.00 15.00      A    C
ATOM   1864  CG  ARG A 245      29.357  -0.897  -6.363  1.00 15.00      A    C
ATOM   1865  CD  ARG A 245      29.599  -1.340  -7.792  1.00 15.00      A    C
ATOM   1866  NE  ARG A 245      28.900  -2.573  -8.142  1.00 15.00      A    N
ATOM   1867  CZ  ARG A 245      28.342  -2.771  -9.337  1.00 15.00      A    C
ATOM   1868  NH1 ARG A 245      28.426  -1.823 -10.265  1.00 15.00      A    N
ATOM   1869  NH2 ARG A 245      27.711  -3.897  -9.612  1.00 15.00      A    N
ATOM   1870  C   ARG A 245      29.318   1.527  -4.801  1.00 15.00      A    C
ATOM   1871  O   ARG A 245      30.117   1.153  -3.954  1.00 15.00      A    O
ATOM   1872  N   ALA A 246      29.526   2.590  -5.574  1.00 15.00      A    N
ATOM   1873  CA  ALA A 246      30.748   3.388  -5.471  1.00 15.00      A    C
ATOM   1874  CB  ALA A 246      30.807   4.437  -6.568  1.00 15.00      A    C
ATOM   1875  C   ALA A 246      30.885   4.048  -4.099  1.00 15.00      A    C
ATOM   1876  O   ALA A 246      31.971   4.083  -3.520  1.00 15.00      A    O
ATOM   1877  N   LYS A 247      29.779   4.562  -3.578  1.00 15.00      A    N
ATOM   1878  CA  LYS A 247      29.783   5.222  -2.279  1.00 15.00      A    C
ATOM   1879  CB  LYS A 247      28.474   5.981  -2.042  1.00 15.00      A    C
ATOM   1880  CG  LYS A 247      28.209   7.101  -3.039  1.00 15.00      A    C
ATOM   1881  CD  LYS A 247      29.042   8.342  -2.744  1.00 15.00      A    C
ATOM   1882  CE  LYS A 247      28.424   9.174  -1.632  1.00 15.00      A    C
ATOM   1883  NZ  LYS A 247      28.825  10.604  -1.706  1.00 15.00      A    N
ATOM   1884  C   LYS A 247      30.038   4.230  -1.150  1.00 15.00      A    C
ATOM   1885  O   LYS A 247      30.833   4.497  -0.250  1.00 15.00      A    O
ATOM   1886  N   LEU A 248      29.367   3.086  -1.207  1.00 15.00      A    N
ATOM   1887  CA  LEU A 248      29.524   2.056  -0.188  1.00 15.00      A    C
ATOM   1888  CB  LEU A 248      28.405   1.017  -0.289  1.00 15.00      A    C
ATOM   1889  CG  LEU A 248      27.012   1.466   0.154  1.00 15.00      A    C
ATOM   1890  CD1 LEU A 248      26.017   0.324   0.016  1.00 15.00      A    C
ATOM   1891  CD2 LEU A 248      27.042   1.978   1.586  1.00 15.00      A    C
ATOM   1892  C   LEU A 248      30.888   1.375  -0.285  1.00 15.00      A    C
ATOM   1893  O   LEU A 248      31.464   0.976   0.727  1.00 15.00      A    O
ATOM   1894  N   MET A 249      31.401   1.252  -1.505  1.00 15.00      A    N
ATOM   1895  CA  MET A 249      32.698   0.619  -1.743  1.00 15.00      A    C
ATOM   1896  CB  MET A 249      32.970   0.512  -3.244  1.00 15.00      A    C
ATOM   1897  CG  MET A 249      34.163  -0.356  -3.614  1.00 15.00      A    C
ATOM   1898  SD  MET A 249      34.530  -0.308  -5.379  1.00 15.00      A    S
ATOM   1899  CE  MET A 249      35.050   1.396  -5.571  1.00 15.00      A    C
ATOM   1900  C   MET A 249      33.822   1.395  -1.065  1.00 15.00      A    C
ATOM   1901  O   MET A 249      34.847   0.827  -0.700  1.00 15.00      A    O
ATOM   1902  N   ASN A 250      33.617   2.693  -0.893  1.00 15.00      A    N
ATOM   1903  CA  ASN A 250      34.615   3.542  -0.254  1.00 15.00      A    C
ATOM   1904  CB  ASN A 250      34.378   5.015  -0.598  1.00 15.00      A    C
ATOM   1905  CG  ASN A 250      34.861   5.374  -1.992  1.00 15.00      A    C
ATOM   1906  OD1 ASN A 250      35.868   4.855  -2.468  1.00 15.00      A    O
ATOM   1907  ND2 ASN A 250      34.140   6.261  -2.659  1.00 15.00      A    N
ATOM   1908  C   ASN A 250      34.614   3.338   1.257  1.00 15.00      A    C
ATOM   1909  O   ASN A 250      35.593   3.641   1.937  1.00 15.00      A    O
ATOM   1910  N   ALA A 251      33.508   2.817   1.774  1.00 15.00      A    N
```

ATOM	1911	CA	ALA	A	251	33.377	2.560	3.202	1.00	15.00	A	C
ATOM	1912	CB	ALA	A	251	31.957	2.850	3.666	1.00	15.00	A	C
ATOM	1913	C	ALA	A	251	33.762	1.118	3.517	1.00	15.00	A	C
ATOM	1914	O	ALA	A	251	34.344	0.829	4.561	1.00	15.00	A	O
ATOM	1915	N	TYR	A	252	33.434	0.216	2.599	1.00	15.00	A	N
ATOM	1916	CA	TYR	A	252	33.749	-1.199	2.757	1.00	15.00	A	C
ATOM	1917	CB	TYR	A	252	32.465	-2.023	2.922	1.00	15.00	A	C
ATOM	1918	CG	TYR	A	252	31.510	-1.521	3.985	1.00	15.00	A	C
ATOM	1919	CD1	TYR	A	252	31.702	-1.830	5.326	1.00	15.00	A	C
ATOM	1920	CD2	TYR	A	252	30.406	-0.750	3.643	1.00	15.00	A	C
ATOM	1921	CE1	TYR	A	252	30.822	-1.385	6.296	1.00	15.00	A	C
ATOM	1922	CE2	TYR	A	252	29.523	-0.298	4.605	1.00	15.00	A	C
ATOM	1923	CZ	TYR	A	252	29.734	-0.619	5.929	1.00	15.00	A	C
ATOM	1924	OH	TYR	A	252	28.854	-0.174	6.889	1.00	15.00	A	O
ATOM	1925	C	TYR	A	252	34.514	-1.702	1.531	1.00	15.00	A	C
ATOM	1926	O	TYR	A	252	33.993	-2.507	0.757	1.00	15.00	A	O
ATOM	1927	N	PRO	A	253	35.771	-1.256	1.347	1.00	15.00	A	N
ATOM	1928	CA	PRO	A	253	36.588	-1.644	0.187	1.00	15.00	A	C
ATOM	1929	CB	PRO	A	253	37.866	-0.814	0.349	1.00	15.00	A	C
ATOM	1930	CG	PRO	A	253	37.928	-0.491	1.801	1.00	15.00	A	C
ATOM	1931	CD	PRO	A	253	36.503	-0.350	2.252	1.00	15.00	A	C
ATOM	1932	C	PRO	A	253	36.924	-3.131	0.167	1.00	15.00	A	C
ATOM	1933	O	PRO	A	253	37.200	-3.702	-0.885	1.00	15.00	A	O
ATOM	1934	N	SER	A	254	36.892	-3.756	1.330	1.00	15.00	A	N
ATOM	1935	CA	SER	A	254	37.205	-5.167	1.441	1.00	15.00	A	C
ATOM	1936	CB	SER	A	254	38.089	-5.383	2.670	1.00	15.00	A	C
ATOM	1937	OG	SER	A	254	37.852	-4.368	3.638	1.00	15.00	A	O
ATOM	1938	C	SER	A	254	35.943	-6.019	1.547	1.00	15.00	A	C
ATOM	1939	O	SER	A	254	36.015	-7.200	1.877	1.00	15.00	A	O
ATOM	1940	N	TYR	A	255	34.786	-5.427	1.263	1.00	15.00	A	N
ATOM	1941	CA	TYR	A	255	33.531	-6.162	1.360	1.00	15.00	A	C
ATOM	1942	CB	TYR	A	255	32.776	-5.767	2.632	1.00	15.00	A	C
ATOM	1943	CG	TYR	A	255	33.519	-6.071	3.915	1.00	15.00	A	C
ATOM	1944	CD1	TYR	A	255	33.843	-7.377	4.260	1.00	15.00	A	C
ATOM	1945	CD2	TYR	A	255	33.895	-5.053	4.781	1.00	15.00	A	C
ATOM	1946	CE1	TYR	A	255	34.518	-7.660	5.431	1.00	15.00	A	C
ATOM	1947	CE2	TYR	A	255	34.572	-5.326	5.955	1.00	15.00	A	C
ATOM	1948	CZ	TYR	A	255	34.881	-6.632	6.274	1.00	15.00	A	C
ATOM	1949	OH	TYR	A	255	35.553	-6.911	7.441	1.00	15.00	A	O
ATOM	1950	C	TYR	A	255	32.627	-5.974	0.143	1.00	15.00	A	C
ATOM	1951	O	TYR	A	255	31.621	-6.669	0.006	1.00	15.00	A	O
ATOM	1952	N	ILE	A	256	32.971	-5.041	-0.735	1.00	15.00	A	N
ATOM	1953	CA	ILE	A	256	32.153	-4.783	-1.915	1.00	15.00	A	C
ATOM	1954	CB	ILE	A	256	31.373	-3.454	-1.787	1.00	15.00	A	C
ATOM	1955	CG1	ILE	A	256	30.508	-3.472	-0.523	1.00	15.00	A	C
ATOM	1956	CG2	ILE	A	256	30.508	-3.209	-3.018	1.00	15.00	A	C
ATOM	1957	CD1	ILE	A	256	29.795	-2.170	-0.243	1.00	15.00	A	C
ATOM	1958	C	ILE	A	256	32.990	-4.776	-3.189	1.00	15.00	A	C
ATOM	1959	O	ILE	A	256	34.095	-4.236	-3.218	1.00	15.00	A	O
ATOM	1960	N	SER	A	257	32.455	-5.388	-4.235	1.00	15.00	A	N
ATOM	1961	CA	SER	A	257	33.128	-5.454	-5.519	1.00	15.00	A	C
ATOM	1962	CB	SER	A	257	32.879	-6.821	-6.168	1.00	15.00	A	C
ATOM	1963	OG	SER	A	257	33.359	-6.865	-7.503	1.00	15.00	A	O
ATOM	1964	C	SER	A	257	32.620	-4.337	-6.426	1.00	15.00	A	C
ATOM	1965	O	SER	A	257	31.417	-4.073	-6.477	1.00	15.00	A	O
ATOM	1966	N	PRO	A	258	33.530	-3.669	-7.155	1.00	15.00	A	N
ATOM	1967	CA	PRO	A	258	33.175	-2.572	-8.066	1.00	15.00	A	C
ATOM	1968	CB	PRO	A	258	34.533	-2.062	-8.563	1.00	15.00	A	C
ATOM	1969	CG	PRO	A	258	35.461	-3.210	-8.370	1.00	15.00	A	C
ATOM	1970	CD	PRO	A	258	34.983	-3.919	-7.137	1.00	15.00	A	C
ATOM	1971	C	PRO	A	258	32.327	-3.045	-9.246	1.00	15.00	A	C
ATOM	1972	O	PRO	A	258	31.746	-2.234	-9.969	1.00	15.00	A	O
ATOM	1973	N	ILE	A	259	32.266	-4.357	-9.440	1.00	15.00	A	N
ATOM	1974	CA	ILE	A	259	31.486	-4.933	-10.530	1.00	15.00	A	C
ATOM	1975	CB	ILE	A	259	32.389	-5.614	-11.585	1.00	15.00	A	C
ATOM	1976	CG1	ILE	A	259	33.168	-6.779	-10.966	1.00	15.00	A	C
ATOM	1977	CG2	ILE	A	259	33.343	-4.605	-12.210	1.00	15.00	A	C
ATOM	1978	CD1	ILE	A	259	33.853	-7.668	-11.982	1.00	15.00	A	C
ATOM	1979	C	ILE	A	259	30.469	-5.946	-10.005	1.00	15.00	A	C
ATOM	1980	O	ILE	A	259	29.535	-6.326	-10.710	1.00	15.00	A	O
ATOM	1981	N	GLY	A	260	30.644	-6.364	-8.758	1.00	15.00	A	N

ATOM	1982	CA	GLY	A	260	29.750	-7.342	-8.167	1.00	15.00	A	C
ATOM	1983	C	GLY	A	260	28.485	-6.730	-7.599	1.00	15.00	A	C
ATOM	1984	O	GLY	A	260	28.353	-5.507	-7.524	1.00	15.00	A	O
ATOM	1985	N	CYS	A	261	27.551	-7.586	-7.208	1.00	15.00	A	N
ATOM	1986	CA	CYS	A	261	26.286	-7.143	-6.638	1.00	15.00	A	C
ATOM	1987	CB	CYS	A	261	25.315	-8.319	-6.551	1.00	15.00	A	C
ATOM	1988	SG	CYS	A	261	24.934	-9.083	-8.142	1.00	15.00	A	S
ATOM	1989	C	CYS	A	261	26.488	-6.523	-5.256	1.00	15.00	A	C
ATOM	1990	O	CYS	A	261	27.583	-6.571	-4.693	1.00	15.00	A	O
ATOM	1991	N	LEU	A	262	25.426	-5.947	-4.710	1.00	15.00	A	N
ATOM	1992	CA	LEU	A	262	25.490	-5.317	-3.399	1.00	15.00	A	C
ATOM	1993	CB	LEU	A	262	24.615	-4.061	-3.363	1.00	15.00	A	C
ATOM	1994	CG	LEU	A	262	25.141	-2.845	-4.130	1.00	15.00	A	C
ATOM	1995	CD1	LEU	A	262	24.170	-1.680	-4.015	1.00	15.00	A	C
ATOM	1996	CD2	LEU	A	262	26.519	-2.448	-3.624	1.00	15.00	A	C
ATOM	1997	C	LEU	A	262	25.056	-6.286	-2.307	1.00	15.00	A	C
ATOM	1998	O	LEU	A	262	24.147	-7.091	-2.514	1.00	15.00	A	O
ATOM	1999	N	PRO	A	263	25.716	-6.234	-1.139	1.00	15.00	A	N
ATOM	2000	CA	PRO	A	263	25.392	-7.102	-0.001	1.00	15.00	A	C
ATOM	2001	CB	PRO	A	263	26.393	-6.676	1.079	1.00	15.00	A	C
ATOM	2002	CG	PRO	A	263	27.495	-6.007	0.333	1.00	15.00	A	C
ATOM	2003	CD	PRO	A	263	26.844	-5.337	-0.840	1.00	15.00	A	C
ATOM	2004	C	PRO	A	263	23.964	-6.876	0.490	1.00	15.00	A	C
ATOM	2005	O	PRO	A	263	23.555	-5.744	0.749	1.00	15.00	A	O
ATOM	2006	N	ALA	A	264	23.219	-7.966	0.624	1.00	15.00	A	N
ATOM	2007	CA	ALA	A	264	21.827	-7.915	1.065	1.00	15.00	A	C
ATOM	2008	CB	ALA	A	264	21.223	-9.311	1.056	1.00	15.00	A	C
ATOM	2009	C	ALA	A	264	21.652	-7.266	2.437	1.00	15.00	A	C
ATOM	2010	O	ALA	A	264	20.620	-6.656	2.713	1.00	15.00	A	O
ATOM	2011	N	HIS	A	265	22.657	-7.389	3.293	1.00	15.00	A	N
ATOM	2012	CA	HIS	A	265	22.585	-6.822	4.635	1.00	15.00	A	C
ATOM	2013	CB	HIS	A	265	23.352	-7.689	5.644	1.00	15.00	A	C
ATOM	2014	CG	HIS	A	265	24.821	-7.838	5.373	1.00	15.00	A	C
ATOM	2015	ND1	HIS	A	265	25.288	-8.335	4.177	1.00	15.00	A	N
ATOM	2016	CD2	HIS	A	265	25.873	-7.562	6.182	1.00	15.00	A	C
ATOM	2017	CE1	HIS	A	265	26.604	-8.354	4.285	1.00	15.00	A	C
ATOM	2018	NE2	HIS	A	265	27.000	-7.897	5.480	1.00	15.00	A	N
ATOM	2019	C	HIS	A	265	23.064	-5.373	4.685	1.00	15.00	A	C
ATOM	2020	O	HIS	A	265	23.053	-4.743	5.744	1.00	15.00	A	O
ATOM	2021	N	LEU	A	266	23.458	-4.835	3.541	1.00	15.00	A	N
ATOM	2022	CA	LEU	A	266	23.948	-3.462	3.474	1.00	15.00	A	C
ATOM	2023	CB	LEU	A	266	25.411	-3.434	3.019	1.00	15.00	A	C
ATOM	2024	CG	LEU	A	266	26.441	-4.011	3.995	1.00	15.00	A	C
ATOM	2025	CD1	LEU	A	266	27.846	-3.873	3.430	1.00	15.00	A	C
ATOM	2026	CD2	LEU	A	266	26.342	-3.329	5.351	1.00	15.00	A	C
ATOM	2027	C	LEU	A	266	23.093	-2.620	2.535	1.00	15.00	A	C
ATOM	2028	O	LEU	A	266	23.568	-1.651	1.949	1.00	15.00	A	O
ATOM	2029	N	LEU	A	267	21.826	-2.990	2.405	1.00	15.00	A	N
ATOM	2030	CA	LEU	A	267	20.913	-2.269	1.526	1.00	15.00	A	C
ATOM	2031	CB	LEU	A	267	19.910	-3.227	0.889	1.00	15.00	A	C
ATOM	2032	CG	LEU	A	267	20.501	-4.319	-0.002	1.00	15.00	A	C
ATOM	2033	CD1	LEU	A	267	19.411	-5.264	-0.476	1.00	15.00	A	C
ATOM	2034	CD2	LEU	A	267	21.237	-3.709	-1.187	1.00	15.00	A	C
ATOM	2035	C	LEU	A	267	20.185	-1.140	2.248	1.00	15.00	A	C
ATOM	2036	O	LEU	A	267	19.719	-0.195	1.622	1.00	15.00	A	O
ATOM	2037	N	GLY	A	268	20.081	-1.235	3.564	1.00	15.00	A	N
ATOM	2038	CA	GLY	A	268	19.405	-0.195	4.314	1.00	15.00	A	C
ATOM	2039	C	GLY	A	268	18.458	-0.748	5.351	1.00	15.00	A	C
ATOM	2040	O	GLY	A	268	18.243	-0.128	6.389	1.00	15.00	A	O
ATOM	2041	N	ASP	A	269	17.885	-1.909	5.074	1.00	15.00	A	N
ATOM	2042	CA	ASP	A	269	16.964	-2.538	6.013	1.00	15.00	A	C
ATOM	2043	CB	ASP	A	269	15.486	-2.288	5.660	1.00	15.00	A	C
ATOM	2044	CG	ASP	A	269	15.053	-2.876	4.333	1.00	15.00	A	C
ATOM	2045	OD1	ASP	A	269	14.675	-4.068	4.301	1.00	15.00	A	O
ATOM	2046	OD2	ASP	A	269	15.076	-2.150	3.321	1.00	15.00	A	O
ATOM	2047	C	ASP	A	269	17.283	-4.019	6.214	1.00	15.00	A	C
ATOM	2048	O	ASP	A	269	18.424	-4.442	6.011	1.00	15.00	A	O
ATOM	2049	N	MET	A	270	16.281	-4.795	6.607	1.00	15.00	A	N
ATOM	2050	CA	MET	A	270	16.459	-6.218	6.878	1.00	15.00	A	C
ATOM	2051	CB	MET	A	270	15.512	-6.650	8.007	1.00	15.00	A	C
ATOM	2052	CG	MET	A	270	15.427	-8.152	8.233	1.00	15.00	A	C

ATOM	2053	SD	MET	A	270	16.941	-8.846	8.923	1.00	15.00	A	S
ATOM	2054	CE	MET	A	270	16.751	-8.419	10.651	1.00	15.00	A	C
ATOM	2055	C	MET	A	270	16.244	-7.081	5.632	1.00	15.00	A	C
ATOM	2056	O	MET	A	270	16.742	-8.209	5.550	1.00	15.00	A	O
ATOM	2057	N	TRP	A	271	15.509	-6.562	4.661	1.00	15.00	A	N
ATOM	2058	CA	TRP	A	271	15.239	-7.311	3.437	1.00	15.00	A	C
ATOM	2059	CB	TRP	A	271	13.728	-7.479	3.231	1.00	15.00	A	C
ATOM	2060	CG	TRP	A	271	12.997	-8.021	4.426	1.00	15.00	A	C
ATOM	2061	CD1	TRP	A	271	12.763	-9.334	4.721	1.00	15.00	A	C
ATOM	2062	CD2	TRP	A	271	12.397	-7.261	5.483	1.00	15.00	A	C
ATOM	2063	NE1	TRP	A	271	12.058	-9.435	5.896	1.00	15.00	A	N
ATOM	2064	CE2	TRP	A	271	11.822	-8.178	6.384	1.00	15.00	A	C
ATOM	2065	CE3	TRP	A	271	12.292	-5.895	5.755	1.00	15.00	A	C
ATOM	2066	CZ2	TRP	A	271	11.154	-7.773	7.537	1.00	15.00	A	C
ATOM	2067	CZ3	TRP	A	271	11.629	-5.496	6.900	1.00	15.00	A	C
ATOM	2068	CH2	TRP	A	271	11.070	-6.431	7.777	1.00	15.00	A	C
ATOM	2069	C	TRP	A	271	15.853	-6.619	2.223	1.00	15.00	A	C
ATOM	2070	O	TRP	A	271	16.195	-7.264	1.222	1.00	15.00	A	O
ATOM	2071	N	GLY	A	272	15.991	-5.308	2.321	1.00	15.00	A	N
ATOM	2072	CA	GLY	A	272	16.537	-4.532	1.237	1.00	15.00	A	C
ATOM	2073	C	GLY	A	272	15.424	-3.989	0.374	1.00	15.00	A	C
ATOM	2074	O	GLY	A	272	15.400	-4.211	-0.834	1.00	15.00	A	O
ATOM	2075	N	ARG	A	273	14.510	-3.269	1.008	1.00	15.00	A	N
ATOM	2076	CA	ARG	A	273	13.360	-2.695	0.340	1.00	15.00	A	C
ATOM	2077	CB	ARG	A	273	12.080	-3.097	1.086	1.00	15.00	A	C
ATOM	2078	CG	ARG	A	273	10.843	-3.204	0.204	1.00	15.00	A	C
ATOM	2079	CD	ARG	A	273	9.813	-2.139	0.531	1.00	15.00	A	C
ATOM	2080	NE	ARG	A	273	9.068	-2.426	1.755	1.00	15.00	A	N
ATOM	2081	CZ	ARG	A	273	7.763	-2.199	1.891	1.00	15.00	A	C
ATOM	2082	NH1	ARG	A	273	7.064	-1.703	0.875	1.00	15.00	A	N
ATOM	2083	NH2	ARG	A	273	7.162	-2.432	3.049	1.00	15.00	A	N
ATOM	2084	C	ARG	A	273	13.474	-1.174	0.290	1.00	15.00	A	C
ATOM	2085	O	ARG	A	273	12.924	-0.533	-0.607	1.00	15.00	A	O
ATOM	2086	N	PHE	A	274	14.162	-0.598	1.271	1.00	15.00	A	N
ATOM	2087	CA	PHE	A	274	14.347	0.846	1.327	1.00	15.00	A	C
ATOM	2088	CB	PHE	A	274	13.448	1.491	2.384	1.00	15.00	A	C
ATOM	2089	CG	PHE	A	274	11.985	1.457	2.044	1.00	15.00	A	C
ATOM	2090	CD1	PHE	A	274	11.531	1.931	0.824	1.00	15.00	A	C
ATOM	2091	CD2	PHE	A	274	11.063	0.948	2.944	1.00	15.00	A	C
ATOM	2092	CE1	PHE	A	274	10.186	1.897	0.505	1.00	15.00	A	C
ATOM	2093	CE2	PHE	A	274	9.715	0.910	2.631	1.00	15.00	A	C
ATOM	2094	CZ	PHE	A	274	9.276	1.386	1.411	1.00	15.00	A	C
ATOM	2095	C	PHE	A	274	15.808	1.198	1.581	1.00	15.00	A	C
ATOM	2096	O	PHE	A	274	16.365	0.893	2.636	1.00	15.00	A	O
ATOM	2097	N	TRP	A	275	16.423	1.851	0.606	1.00	15.00	A	N
ATOM	2098	CA	TRP	A	275	17.821	2.248	0.707	1.00	15.00	A	C
ATOM	2099	CB	TRP	A	275	18.469	2.284	-0.682	1.00	15.00	A	C
ATOM	2100	CG	TRP	A	275	18.567	0.957	-1.378	1.00	15.00	A	C
ATOM	2101	CD1	TRP	A	275	18.049	-0.240	-0.969	1.00	15.00	A	C
ATOM	2102	CD2	TRP	A	275	19.231	0.698	-2.620	1.00	15.00	A	C
ATOM	2103	NE1	TRP	A	275	18.354	-1.225	-1.879	1.00	15.00	A	N
ATOM	2104	CE2	TRP	A	275	19.079	-0.673	-2.901	1.00	15.00	A	C
ATOM	2105	CE3	TRP	A	275	19.942	1.495	-3.521	1.00	15.00	A	C
ATOM	2106	CZ2	TRP	A	275	19.611	-1.263	-4.047	1.00	15.00	A	C
ATOM	2107	CZ3	TRP	A	275	20.468	0.910	-4.657	1.00	15.00	A	C
ATOM	2108	CH2	TRP	A	275	20.301	-0.456	-4.910	1.00	15.00	A	C
ATOM	2109	C	TRP	A	275	17.934	3.627	1.346	1.00	15.00	A	C
ATOM	2110	O	TRP	A	275	19.001	4.235	1.346	1.00	15.00	A	O
ATOM	2111	N	THR	A	276	16.825	4.116	1.891	1.00	15.00	A	N
ATOM	2112	CA	THR	A	276	16.783	5.426	2.528	1.00	15.00	A	C
ATOM	2113	CB	THR	A	276	15.375	5.715	3.079	1.00	15.00	A	C
ATOM	2114	OG1	THR	A	276	14.403	5.085	2.232	1.00	15.00	A	O
ATOM	2115	CG2	THR	A	276	15.116	7.215	3.122	1.00	15.00	A	C
ATOM	2116	C	THR	A	276	17.800	5.555	3.664	1.00	15.00	A	C
ATOM	2117	O	THR	A	276	18.308	6.646	3.930	1.00	15.00	A	O
ATOM	2118	N	ASN	A	277	18.118	4.439	4.310	1.00	15.00	A	N
ATOM	2119	CA	ASN	A	277	19.066	4.449	5.422	1.00	15.00	A	C
ATOM	2120	CB	ASN	A	277	18.863	3.242	6.338	1.00	15.00	A	C
ATOM	2121	CG	ASN	A	277	17.566	3.315	7.119	1.00	15.00	A	C
ATOM	2122	OD1	ASN	A	277	17.062	4.398	7.412	1.00	15.00	A	O
ATOM	2123	ND2	ASN	A	277	17.016	2.162	7.460	1.00	15.00	A	N

ATOM	2124	C	ASN	A	277	20.506	4.515	4.934	1.00	15.00	A	C
ATOM	2125	O	ASN	A	277	21.438	4.629	5.727	1.00	15.00	A	O
ATOM	2126	N	LEU	A	278	20.685	4.448	3.623	1.00	15.00	A	N
ATOM	2127	CA	LEU	A	278	22.012	4.515	3.036	1.00	15.00	A	C
ATOM	2128	CB	LEU	A	278	22.104	3.640	1.784	1.00	15.00	A	C
ATOM	2129	CG	LEU	A	278	22.087	2.126	1.999	1.00	15.00	A	C
ATOM	2130	CD1	LEU	A	278	22.339	1.407	0.683	1.00	15.00	A	C
ATOM	2131	CD2	LEU	A	278	23.116	1.715	3.042	1.00	15.00	A	C
ATOM	2132	C	LEU	A	278	22.361	5.949	2.678	1.00	15.00	A	C
ATOM	2133	O	LEU	A	278	23.485	6.233	2.268	1.00	15.00	A	O
ATOM	2134	N	TYR	A	279	21.394	6.851	2.846	1.00	15.00	A	N
ATOM	2135	CA	TYR	A	279	21.577	8.269	2.529	1.00	15.00	A	C
ATOM	2136	CB	TYR	A	279	20.296	9.052	2.842	1.00	15.00	A	C
ATOM	2137	CG	TYR	A	279	20.372	10.537	2.548	1.00	15.00	A	C
ATOM	2138	CD1	TYR	A	279	20.329	11.014	1.243	1.00	15.00	A	C
ATOM	2139	CD2	TYR	A	279	20.480	11.462	3.580	1.00	15.00	A	C
ATOM	2140	CE1	TYR	A	279	20.390	12.370	0.975	1.00	15.00	A	C
ATOM	2141	CE2	TYR	A	279	20.544	12.818	3.321	1.00	15.00	A	C
ATOM	2142	CZ	TYR	A	279	20.498	13.267	2.018	1.00	15.00	A	C
ATOM	2143	OH	TYR	A	279	20.556	14.618	1.761	1.00	15.00	A	O
ATOM	2144	C	TYR	A	279	22.764	8.869	3.278	1.00	15.00	A	C
ATOM	2145	O	TYR	A	279	23.395	9.812	2.807	1.00	15.00	A	O
ATOM	2146	N	SER	A	280	23.072	8.301	4.432	1.00	15.00	A	N
ATOM	2147	CA	SER	A	280	24.174	8.769	5.254	1.00	15.00	A	C
ATOM	2148	CB	SER	A	280	24.141	8.007	6.576	1.00	15.00	A	C
ATOM	2149	OG	SER	A	280	23.243	6.911	6.478	1.00	15.00	A	O
ATOM	2150	C	SER	A	280	25.517	8.548	4.559	1.00	15.00	A	C
ATOM	2151	O	SER	A	280	26.472	9.288	4.783	1.00	15.00	A	O
ATOM	2152	N	LEU	A	281	25.578	7.537	3.700	1.00	15.00	A	N
ATOM	2153	CA	LEU	A	281	26.809	7.215	2.995	1.00	15.00	A	C
ATOM	2154	CB	LEU	A	281	27.194	5.752	3.237	1.00	15.00	A	C
ATOM	2155	CG	LEU	A	281	27.403	5.341	4.698	1.00	15.00	A	C
ATOM	2156	CD1	LEU	A	281	27.636	3.843	4.806	1.00	15.00	A	C
ATOM	2157	CD2	LEU	A	281	28.562	6.110	5.315	1.00	15.00	A	C
ATOM	2158	C	LEU	A	281	26.713	7.496	1.498	1.00	15.00	A	C
ATOM	2159	O	LEU	A	281	27.732	7.658	0.831	1.00	15.00	A	O
ATOM	2160	N	THR	A	282	25.497	7.559	0.969	1.00	15.00	A	N
ATOM	2161	CA	THR	A	282	25.301	7.807	-0.455	1.00	15.00	A	C
ATOM	2162	CB	THR	A	282	24.154	6.957	-1.034	1.00	15.00	A	C
ATOM	2163	OG1	THR	A	282	23.003	7.063	-0.190	1.00	15.00	A	O
ATOM	2164	CG2	THR	A	282	24.570	5.498	-1.130	1.00	15.00	A	C
ATOM	2165	C	THR	A	282	25.048	9.282	-0.755	1.00	15.00	A	C
ATOM	2166	O	THR	A	282	24.902	9.669	-1.913	1.00	15.00	A	O
ATOM	2167	N	VAL	A	283	25.013	10.100	0.292	1.00	15.00	A	N
ATOM	2168	CA	VAL	A	283	24.776	11.534	0.145	1.00	15.00	A	C
ATOM	2169	CB	VAL	A	283	24.749	12.256	1.515	1.00	15.00	A	C
ATOM	2170	CG1	VAL	A	283	26.051	12.054	2.280	1.00	15.00	A	C
ATOM	2171	CG2	VAL	A	283	24.436	13.736	1.347	1.00	15.00	A	C
ATOM	2172	C	VAL	A	283	25.809	12.191	-0.774	1.00	15.00	A	C
ATOM	2173	O	VAL	A	283	27.017	12.080	-0.552	1.00	15.00	A	O
ATOM	2174	N	PRO	A	284	25.344	12.857	-1.839	1.00	15.00	A	N
ATOM	2175	CA	PRO	A	284	26.223	13.543	-2.788	1.00	15.00	A	C
ATOM	2176	CB	PRO	A	284	25.267	14.039	-3.882	1.00	15.00	A	C
ATOM	2177	CG	PRO	A	284	24.007	13.270	-3.683	1.00	15.00	A	C
ATOM	2178	CD	PRO	A	284	23.928	12.978	-2.215	1.00	15.00	A	C
ATOM	2179	C	PRO	A	284	26.923	14.729	-2.136	1.00	15.00	A	C
ATOM	2180	O	PRO	A	284	28.145	14.740	-1.987	1.00	15.00	A	O
ATOM	2181	N	PHE	A	285	26.136	15.712	-1.727	1.00	15.00	A	N
ATOM	2182	CA	PHE	A	285	26.668	16.908	-1.099	1.00	15.00	A	C
ATOM	2183	CB	PHE	A	285	26.142	18.156	-1.813	1.00	15.00	A	C
ATOM	2184	CG	PHE	A	285	26.287	18.093	-3.309	1.00	15.00	A	C
ATOM	2185	CD1	PHE	A	285	27.511	18.332	-3.910	1.00	15.00	A	C
ATOM	2186	CD2	PHE	A	285	25.199	17.789	-4.112	1.00	15.00	A	C
ATOM	2187	CE1	PHE	A	285	27.650	18.270	-5.284	1.00	15.00	A	C
ATOM	2188	CE2	PHE	A	285	25.329	17.726	-5.488	1.00	15.00	A	C
ATOM	2189	CZ	PHE	A	285	26.557	17.967	-6.075	1.00	15.00	A	C
ATOM	2190	C	PHE	A	285	26.301	16.934	0.379	1.00	15.00	A	C
ATOM	2191	O	PHE	A	285	25.249	17.446	0.761	1.00	15.00	A	O
ATOM	2192	N	GLY	A	286	27.176	16.373	1.204	1.00	15.00	A	N
ATOM	2193	CA	GLY	A	286	26.929	16.317	2.636	1.00	15.00	A	C
ATOM	2194	C	GLY	A	286	27.120	17.651	3.331	1.00	15.00	A	C

ATOM	2195	O	GLY	A	286	26.864	17.777	4.525	1.00	15.00	A	O
ATOM	2196	N	GLN	A	287	27.563	18.651	2.585	1.00	15.00	A	N
ATOM	2197	CA	GLN	A	287	27.788	19.974	3.145	1.00	15.00	A	C
ATOM	2198	CB	GLN	A	287	29.117	20.544	2.654	1.00	15.00	A	C
ATOM	2199	CG	GLN	A	287	30.316	19.721	3.094	1.00	15.00	A	C
ATOM	2200	CD	GLN	A	287	31.483	20.576	3.541	1.00	15.00	A	C
ATOM	2201	OE1	GLN	A	287	32.404	20.841	2.775	1.00	15.00	A	O
ATOM	2202	NE2	GLN	A	287	31.444	21.021	4.788	1.00	15.00	A	N
ATOM	2203	C	GLN	A	287	26.629	20.912	2.822	1.00	15.00	A	C
ATOM	2204	O	GLN	A	287	26.756	22.133	2.901	1.00	15.00	A	O
ATOM	2205	N	LYS	A	288	25.495	20.326	2.468	1.00	15.00	A	N
ATOM	2206	CA	LYS	A	288	24.308	21.095	2.138	1.00	15.00	A	C
ATOM	2207	CB	LYS	A	288	23.769	20.699	0.764	1.00	15.00	A	C
ATOM	2208	CG	LYS	A	288	24.524	21.321	-0.394	1.00	15.00	A	C
ATOM	2209	CD	LYS	A	288	24.264	22.817	-0.487	1.00	15.00	A	C
ATOM	2210	CE	LYS	A	288	25.230	23.470	-1.456	1.00	15.00	A	C
ATOM	2211	NZ	LYS	A	288	24.777	24.808	-1.913	1.00	15.00	A	N
ATOM	2212	C	LYS	A	288	23.229	20.908	3.195	1.00	15.00	A	C
ATOM	2213	O	LYS	A	288	22.821	19.782	3.481	1.00	15.00	A	O
ATOM	2214	N	PRO	A	289	22.768	22.016	3.794	1.00	15.00	A	N
ATOM	2215	CA	PRO	A	289	21.726	21.987	4.824	1.00	15.00	A	C
ATOM	2216	CB	PRO	A	289	21.507	23.468	5.167	1.00	15.00	A	C
ATOM	2217	CG	PRO	A	289	22.095	24.225	4.024	1.00	15.00	A	C
ATOM	2218	CD	PRO	A	289	23.227	23.384	3.515	1.00	15.00	A	C
ATOM	2219	C	PRO	A	289	20.431	21.369	4.305	1.00	15.00	A	C
ATOM	2220	O	PRO	A	289	19.927	21.751	3.248	1.00	15.00	A	O
ATOM	2221	N	ASN	A	290	19.909	20.400	5.044	1.00	15.00	A	N
ATOM	2222	CA	ASN	A	290	18.674	19.732	4.659	1.00	15.00	A	C
ATOM	2223	CB	ASN	A	290	18.557	18.369	5.346	1.00	15.00	A	C
ATOM	2224	CG	ASN	A	290	18.094	17.278	4.397	1.00	15.00	A	C
ATOM	2225	OD1	ASN	A	290	16.957	16.816	4.463	1.00	15.00	A	O
ATOM	2226	ND2	ASN	A	290	18.976	16.864	3.499	1.00	15.00	A	N
ATOM	2227	C	ASN	A	290	17.473	20.609	4.987	1.00	15.00	A	C
ATOM	2228	O	ASN	A	290	17.594	21.588	5.725	1.00	15.00	A	O
ATOM	2229	N	ILE	A	291	16.318	20.260	4.446	1.00	15.00	A	N
ATOM	2230	CA	ILE	A	291	15.111	21.037	4.673	1.00	15.00	A	C
ATOM	2231	CB	ILE	A	291	14.259	21.153	3.390	1.00	15.00	A	C
ATOM	2232	CG1	ILE	A	291	15.150	21.442	2.177	1.00	15.00	A	C
ATOM	2233	CG2	ILE	A	291	13.209	22.244	3.550	1.00	15.00	A	C
ATOM	2234	CD1	ILE	A	291	14.432	21.344	0.847	1.00	15.00	A	C
ATOM	2235	C	ILE	A	291	14.264	20.432	5.786	1.00	15.00	A	C
ATOM	2236	O	ILE	A	291	13.529	19.471	5.563	1.00	15.00	A	O
ATOM	2237	N	ASP	A	292	14.396	20.985	6.984	1.00	15.00	A	N
ATOM	2238	CA	ASP	A	292	13.626	20.527	8.134	1.00	15.00	A	C
ATOM	2239	CB	ASP	A	292	14.516	19.801	9.152	1.00	15.00	A	C
ATOM	2240	CG	ASP	A	292	13.725	19.121	10.262	1.00	15.00	A	C
ATOM	2241	OD1	ASP	A	292	12.488	19.303	10.329	1.00	15.00	A	O
ATOM	2242	OD2	ASP	A	292	14.336	18.403	11.074	1.00	15.00	A	O
ATOM	2243	C	ASP	A	292	12.939	21.720	8.783	1.00	15.00	A	C
ATOM	2244	O	ASP	A	292	13.562	22.760	9.007	1.00	15.00	A	O
ATOM	2245	N	VAL	A	293	11.655	21.578	9.063	1.00	15.00	A	N
ATOM	2246	CA	VAL	A	293	10.884	22.650	9.672	1.00	15.00	A	C
ATOM	2247	CB	VAL	A	293	9.626	22.994	8.843	1.00	15.00	A	C
ATOM	2248	CG1	VAL	A	293	10.015	23.464	7.449	1.00	15.00	A	C
ATOM	2249	CG2	VAL	A	293	8.682	21.802	8.766	1.00	15.00	A	C
ATOM	2250	C	VAL	A	293	10.474	22.306	11.099	1.00	15.00	A	C
ATOM	2251	O	VAL	A	293	9.731	23.057	11.731	1.00	15.00	A	O
ATOM	2252	N	THR	A	294	10.969	21.179	11.605	1.00	15.00	A	N
ATOM	2253	CA	THR	A	294	10.650	20.733	12.958	1.00	15.00	A	C
ATOM	2254	CB	THR	A	294	11.398	19.431	13.309	1.00	15.00	A	C
ATOM	2255	OG1	THR	A	294	11.089	18.428	12.329	1.00	15.00	A	O
ATOM	2256	CG2	THR	A	294	10.996	18.927	14.687	1.00	15.00	A	C
ATOM	2257	C	THR	A	294	10.964	21.817	13.991	1.00	15.00	A	C
ATOM	2258	O	THR	A	294	10.113	22.164	14.814	1.00	15.00	A	O
ATOM	2259	N	ASP	A	295	12.174	22.365	13.925	1.00	15.00	A	N
ATOM	2260	CA	ASP	A	295	12.592	23.417	14.850	1.00	15.00	A	C
ATOM	2261	CB	ASP	A	295	14.041	23.837	14.585	1.00	15.00	A	C
ATOM	2262	CG	ASP	A	295	15.054	22.913	15.232	1.00	15.00	A	C
ATOM	2263	OD1	ASP	A	295	14.784	22.417	16.346	1.00	15.00	A	O
ATOM	2264	OD2	ASP	A	295	16.120	22.686	14.623	1.00	15.00	A	O
ATOM	2265	C	ASP	A	295	11.675	24.629	14.736	1.00	15.00	A	C

ATOM	2266	O	ASP	A	295	11.196	25.157	15.739	1.00	15.00	A	O
ATOM	2267	N	ALA	A	296	11.403	25.037	13.499	1.00	15.00	A	N
ATOM	2268	CA	ALA	A	296	10.541	26.186	13.235	1.00	15.00	A	C
ATOM	2269	CB	ALA	A	296	10.482	26.476	11.743	1.00	15.00	A	C
ATOM	2270	C	ALA	A	296	9.139	25.959	13.794	1.00	15.00	A	C
ATOM	2271	O	ALA	A	296	8.481	26.893	14.254	1.00	15.00	A	O
ATOM	2272	N	MET	A	297	8.687	24.710	13.748	1.00	15.00	A	N
ATOM	2273	CA	MET	A	297	7.375	24.354	14.267	1.00	15.00	A	C
ATOM	2274	CB	MET	A	297	7.022	22.907	13.907	1.00	15.00	A	C
ATOM	2275	CG	MET	A	297	6.659	22.701	12.444	1.00	15.00	A	C
ATOM	2276	SD	MET	A	297	6.660	20.965	11.956	1.00	15.00	A	S
ATOM	2277	CE	MET	A	297	5.334	20.326	12.975	1.00	15.00	A	C
ATOM	2278	C	MET	A	297	7.341	24.547	15.777	1.00	15.00	A	C
ATOM	2279	O	MET	A	297	6.362	25.045	16.327	1.00	15.00	A	O
ATOM	2280	N	VAL	A	298	8.425	24.166	16.440	1.00	15.00	A	N
ATOM	2281	CA	VAL	A	298	8.523	24.311	17.887	1.00	15.00	A	C
ATOM	2282	CB	VAL	A	298	9.762	23.582	18.454	1.00	15.00	A	C
ATOM	2283	CG1	VAL	A	298	9.852	23.755	19.964	1.00	15.00	A	C
ATOM	2284	CG2	VAL	A	298	9.724	22.105	18.092	1.00	15.00	A	C
ATOM	2285	C	VAL	A	298	8.577	25.791	18.264	1.00	15.00	A	C
ATOM	2286	O	VAL	A	298	7.923	26.222	19.214	1.00	15.00	A	O
ATOM	2287	N	ASP	A	299	9.340	26.560	17.491	1.00	15.00	A	N
ATOM	2288	CA	ASP	A	299	9.489	28.000	17.718	1.00	15.00	A	C
ATOM	2289	CB	ASP	A	299	10.423	28.613	16.669	1.00	15.00	A	C
ATOM	2290	CG	ASP	A	299	11.863	28.717	17.130	1.00	15.00	A	C
ATOM	2291	OD1	ASP	A	299	12.091	28.887	18.346	1.00	15.00	A	O
ATOM	2292	OD2	ASP	A	299	12.765	28.641	16.269	1.00	15.00	A	O
ATOM	2293	C	ASP	A	299	8.146	28.718	17.666	1.00	15.00	A	C
ATOM	2294	O	ASP	A	299	7.861	29.592	18.484	1.00	15.00	A	O
ATOM	2295	N	GLN	A	300	7.315	28.340	16.701	1.00	15.00	A	N
ATOM	2296	CA	GLN	A	300	6.003	28.956	16.538	1.00	15.00	A	C
ATOM	2297	CB	GLN	A	300	5.563	28.912	15.077	1.00	15.00	A	C
ATOM	2298	CG	GLN	A	300	6.450	29.686	14.120	1.00	15.00	A	C
ATOM	2299	CD	GLN	A	300	5.953	29.605	12.691	1.00	15.00	A	C
ATOM	2300	OE1	GLN	A	300	4.751	29.475	12.443	1.00	15.00	A	O
ATOM	2301	NE2	GLN	A	300	6.872	29.671	11.743	1.00	15.00	A	N
ATOM	2302	C	GLN	A	300	4.948	28.282	17.412	1.00	15.00	A	C
ATOM	2303	O	GLN	A	300	3.760	28.589	17.302	1.00	15.00	A	O
ATOM	2304	N	ALA	A	301	5.394	27.360	18.265	1.00	15.00	A	N
ATOM	2305	CA	ALA	A	301	4.509	26.629	19.172	1.00	15.00	A	C
ATOM	2306	CB	ALA	A	301	3.890	27.559	20.209	1.00	15.00	A	C
ATOM	2307	C	ALA	A	301	3.436	25.839	18.420	1.00	15.00	A	C
ATOM	2308	O	ALA	A	301	2.245	25.953	18.707	1.00	15.00	A	O
ATOM	2309	N	TRP	A	302	3.867	25.042	17.453	1.00	15.00	A	N
ATOM	2310	CA	TRP	A	302	2.950	24.228	16.663	1.00	15.00	A	C
ATOM	2311	CB	TRP	A	302	3.600	23.816	15.337	1.00	15.00	A	C
ATOM	2312	CG	TRP	A	302	3.543	24.864	14.265	1.00	15.00	A	C
ATOM	2313	CD1	TRP	A	302	3.924	26.169	14.369	1.00	15.00	A	C
ATOM	2314	CD2	TRP	A	302	3.082	24.689	12.920	1.00	15.00	A	C
ATOM	2315	NE1	TRP	A	302	3.725	26.819	13.175	1.00	15.00	A	N
ATOM	2316	CE2	TRP	A	302	3.210	25.932	12.269	1.00	15.00	A	C
ATOM	2317	CE3	TRP	A	302	2.575	23.602	12.204	1.00	15.00	A	C
ATOM	2318	CZ2	TRP	A	302	2.847	26.117	10.939	1.00	15.00	A	C
ATOM	2319	CZ3	TRP	A	302	2.216	23.788	10.883	1.00	15.00	A	C
ATOM	2320	CH2	TRP	A	302	2.355	25.035	10.264	1.00	15.00	A	C
ATOM	2321	C	TRP	A	302	2.525	22.978	17.429	1.00	15.00	A	C
ATOM	2322	O	TRP	A	302	3.366	22.233	17.933	1.00	15.00	A	O
ATOM	2323	N	ASP	A	303	1.221	22.758	17.520	1.00	15.00	A	N
ATOM	2324	CA	ASP	A	303	0.689	21.585	18.205	1.00	15.00	A	C
ATOM	2325	CB	ASP	A	303	-0.282	21.992	19.326	1.00	15.00	A	C
ATOM	2326	CG	ASP	A	303	-1.739	21.937	18.908	1.00	15.00	A	C
ATOM	2327	OD1	ASP	A	303	-2.138	22.735	18.032	1.00	15.00	A	O
ATOM	2328	OD2	ASP	A	303	-2.477	21.073	19.432	1.00	15.00	A	O
ATOM	2329	C	ASP	A	303	0.010	20.658	17.195	1.00	15.00	A	C
ATOM	2330	O	ASP	A	303	-0.005	20.950	15.995	1.00	15.00	A	O
ATOM	2331	N	ALA	A	304	-0.552	19.555	17.681	1.00	15.00	A	N
ATOM	2332	CA	ALA	A	304	-1.224	18.584	16.823	1.00	15.00	A	C
ATOM	2333	CB	ALA	A	304	-1.722	17.410	17.649	1.00	15.00	A	C
ATOM	2334	C	ALA	A	304	-2.377	19.229	16.064	1.00	15.00	A	C
ATOM	2335	O	ALA	A	304	-2.538	19.014	14.858	1.00	15.00	A	O
ATOM	2336	N	GLN	A	305	-3.160	20.034	16.774	1.00	15.00	A	N

ATOM	2337	CA	GLN	A	305	-4.295	20.727	16.182	1.00	15.00	A	C
ATOM	2338	CB	GLN	A	305	-5.009	21.557	17.254	1.00	15.00	A	C
ATOM	2339	CG	GLN	A	305	-6.122	22.458	16.744	1.00	15.00	A	C
ATOM	2340	CD	GLN	A	305	-7.413	21.715	16.464	1.00	15.00	A	C
ATOM	2341	OE1	GLN	A	305	-7.425	20.672	15.817	1.00	15.00	A	O
ATOM	2342	NE2	GLN	A	305	-8.514	22.247	16.967	1.00	15.00	A	N
ATOM	2343	C	GLN	A	305	-3.817	21.620	15.044	1.00	15.00	A	C
ATOM	2344	O	GLN	A	305	-4.373	21.593	13.946	1.00	15.00	A	O
ATOM	2345	N	ARG	A	306	-2.773	22.397	15.314	1.00	15.00	A	N
ATOM	2346	CA	ARG	A	306	-2.197	23.288	14.316	1.00	15.00	A	C
ATOM	2347	CB	ARG	A	306	-1.030	24.081	14.911	1.00	15.00	A	C
ATOM	2348	CG	ARG	A	306	-0.386	25.059	13.940	1.00	15.00	A	C
ATOM	2349	CD	ARG	A	306	-1.257	26.287	13.733	1.00	15.00	A	C
ATOM	2350	NE	ARG	A	306	-0.871	27.045	12.542	1.00	15.00	A	N
ATOM	2351	CZ	ARG	A	306	-0.001	28.055	12.542	1.00	15.00	A	C
ATOM	2352	NH1	ARG	A	306	0.589	28.438	13.673	1.00	15.00	A	N
ATOM	2353	NH2	ARG	A	306	0.272	28.683	11.404	1.00	15.00	A	N
ATOM	2354	C	ARG	A	306	-1.724	22.508	13.093	1.00	15.00	A	C
ATOM	2355	O	ARG	A	306	-1.960	22.921	11.959	1.00	15.00	A	O
ATOM	2356	N	ILE	A	307	-1.067	21.377	13.329	1.00	15.00	A	N
ATOM	2357	CA	ILE	A	307	-0.564	20.544	12.242	1.00	15.00	A	C
ATOM	2358	CB	ILE	A	307	0.222	19.319	12.777	1.00	15.00	A	C
ATOM	2359	CG1	ILE	A	307	1.499	19.779	13.490	1.00	15.00	A	C
ATOM	2360	CG2	ILE	A	307	0.559	18.348	11.651	1.00	15.00	A	C
ATOM	2361	CD1	ILE	A	307	2.208	18.684	14.260	1.00	15.00	A	C
ATOM	2362	C	ILE	A	307	-1.709	20.079	11.337	1.00	15.00	A	C
ATOM	2363	O	ILE	A	307	-1.659	20.242	10.117	1.00	15.00	A	O
ATOM	2364	N	PHE	A	308	-2.753	19.532	11.947	1.00	15.00	A	N
ATOM	2365	CA	PHE	A	308	-3.908	19.030	11.198	1.00	15.00	A	C
ATOM	2366	CB	PHE	A	308	-4.761	18.110	12.070	1.00	15.00	A	C
ATOM	2367	CG	PHE	A	308	-4.261	16.695	12.121	1.00	15.00	A	C
ATOM	2368	CD1	PHE	A	308	-3.272	16.324	13.017	1.00	15.00	A	C
ATOM	2369	CD2	PHE	A	308	-4.776	15.735	11.265	1.00	15.00	A	C
ATOM	2370	CE1	PHE	A	308	-2.811	15.024	13.062	1.00	15.00	A	C
ATOM	2371	CE2	PHE	A	308	-4.319	14.432	11.305	1.00	15.00	A	C
ATOM	2372	CZ	PHE	A	308	-3.333	14.076	12.203	1.00	15.00	A	C
ATOM	2373	C	PHE	A	308	-4.756	20.143	10.569	1.00	15.00	A	C
ATOM	2374	O	PHE	A	308	-5.381	19.947	9.524	1.00	15.00	A	O
ATOM	2375	N	LYS	A	309	-4.780	21.301	11.211	1.00	15.00	A	N
ATOM	2376	CA	LYS	A	309	-5.535	22.445	10.710	1.00	15.00	A	C
ATOM	2377	CB	LYS	A	309	-5.634	23.532	11.780	1.00	15.00	A	C
ATOM	2378	CG	LYS	A	309	-6.893	23.439	12.621	1.00	15.00	A	C
ATOM	2379	CD	LYS	A	309	-8.132	23.492	11.748	1.00	15.00	A	C
ATOM	2380	CE	LYS	A	309	-9.319	22.894	12.472	1.00	15.00	A	C
ATOM	2381	NZ	LYS	A	309	-10.525	22.847	11.613	1.00	15.00	A	N
ATOM	2382	C	LYS	A	309	-4.917	22.998	9.429	1.00	15.00	A	C
ATOM	2383	O	LYS	A	309	-5.619	23.412	8.508	1.00	15.00	A	O
ATOM	2384	N	GLU	A	310	-3.601	22.983	9.367	1.00	15.00	A	N
ATOM	2385	CA	GLU	A	310	-2.898	23.462	8.194	1.00	15.00	A	C
ATOM	2386	CB	GLU	A	310	-1.411	23.541	8.498	1.00	15.00	A	C
ATOM	2387	CG	GLU	A	310	-1.043	24.636	9.482	1.00	15.00	A	C
ATOM	2388	CD	GLU	A	310	-1.565	25.986	9.063	1.00	15.00	A	C
ATOM	2389	OE1	GLU	A	310	-1.703	26.220	7.850	1.00	15.00	A	O
ATOM	2390	OE2	GLU	A	310	-1.845	26.828	9.946	1.00	15.00	A	O
ATOM	2391	C	GLU	A	310	-3.135	22.506	7.037	1.00	15.00	A	C
ATOM	2392	O	GLU	A	310	-3.278	22.917	5.883	1.00	15.00	A	O
ATOM	2393	N	ALA	A	311	-3.192	21.225	7.369	1.00	15.00	A	N
ATOM	2394	CA	ALA	A	311	-3.430	20.192	6.379	1.00	15.00	A	C
ATOM	2395	CB	ALA	A	311	-3.256	18.809	6.986	1.00	15.00	A	C
ATOM	2396	C	ALA	A	311	-4.822	20.349	5.801	1.00	15.00	A	C
ATOM	2397	O	ALA	A	311	-5.013	20.239	4.592	1.00	15.00	A	O
ATOM	2398	N	GLU	A	312	-5.790	20.613	6.678	1.00	15.00	A	N
ATOM	2399	CA	GLU	A	312	-7.170	20.813	6.264	1.00	15.00	A	C
ATOM	2400	CB	GLU	A	312	-8.039	21.056	7.500	1.00	15.00	A	C
ATOM	2401	CG	GLU	A	312	-9.512	21.274	7.207	1.00	15.00	A	C
ATOM	2402	CD	GLU	A	312	-10.288	21.693	8.436	1.00	15.00	A	C
ATOM	2403	OE1	GLU	A	312	-10.026	22.795	8.969	1.00	15.00	A	O
ATOM	2404	OE2	GLU	A	312	-11.165	20.924	8.881	1.00	15.00	A	O
ATOM	2405	C	GLU	A	312	-7.254	22.006	5.317	1.00	15.00	A	C
ATOM	2406	O	GLU	A	312	-7.914	21.955	4.278	1.00	15.00	A	O
ATOM	2407	N	LYS	A	313	-6.562	23.075	5.690	1.00	15.00	A	N

ATOM	2408	CA	LYS	A	313	-6.517	24.293	4.892	1.00	15.00	A	C
ATOM	2409	CB	LYS	A	313	-5.650	25.328	5.607	1.00	15.00	A	C
ATOM	2410	CG	LYS	A	313	-5.625	26.702	4.959	1.00	15.00	A	C
ATOM	2411	CD	LYS	A	313	-4.568	27.583	5.606	1.00	15.00	A	C
ATOM	2412	CE	LYS	A	313	-4.732	27.620	7.119	1.00	15.00	A	C
ATOM	2413	NZ	LYS	A	313	-3.495	28.077	7.801	1.00	15.00	A	N
ATOM	2414	C	LYS	A	313	-5.934	24.002	3.511	1.00	15.00	A	C
ATOM	2415	O	LYS	A	313	-6.355	24.583	2.508	1.00	15.00	A	O
ATOM	2416	N	PHE	A	314	-4.959	23.098	3.475	1.00	15.00	A	N
ATOM	2417	CA	PHE	A	314	-4.310	22.703	2.231	1.00	15.00	A	C
ATOM	2418	CB	PHE	A	314	-3.175	21.705	2.513	1.00	15.00	A	C
ATOM	2419	CG	PHE	A	314	-2.723	20.896	1.324	1.00	15.00	A	C
ATOM	2420	CD1	PHE	A	314	-1.961	21.473	0.321	1.00	15.00	A	C
ATOM	2421	CD2	PHE	A	314	-3.054	19.554	1.218	1.00	15.00	A	C
ATOM	2422	CE1	PHE	A	314	-1.540	20.727	-0.765	1.00	15.00	A	C
ATOM	2423	CE2	PHE	A	314	-2.637	18.803	0.135	1.00	15.00	A	C
ATOM	2424	CZ	PHE	A	314	-1.879	19.391	-0.859	1.00	15.00	A	C
ATOM	2425	C	PHE	A	314	-5.326	22.111	1.261	1.00	15.00	A	C
ATOM	2426	O	PHE	A	314	-5.355	22.471	0.084	1.00	15.00	A	O
ATOM	2427	N	PHE	A	315	-6.175	21.225	1.765	1.00	15.00	A	N
ATOM	2428	CA	PHE	A	315	-7.193	20.591	0.937	1.00	15.00	A	C
ATOM	2429	CB	PHE	A	315	-7.856	19.427	1.676	1.00	15.00	A	C
ATOM	2430	CG	PHE	A	315	-6.991	18.202	1.755	1.00	15.00	A	C
ATOM	2431	CD1	PHE	A	315	-7.082	17.212	0.792	1.00	15.00	A	C
ATOM	2432	CD2	PHE	A	315	-6.082	18.044	2.786	1.00	15.00	A	C
ATOM	2433	CE1	PHE	A	315	-6.283	16.087	0.855	1.00	15.00	A	C
ATOM	2434	CE2	PHE	A	315	-5.280	16.923	2.857	1.00	15.00	A	C
ATOM	2435	CZ	PHE	A	315	-5.380	15.943	1.890	1.00	15.00	A	C
ATOM	2436	C	PHE	A	315	-8.235	21.603	0.478	1.00	15.00	A	C
ATOM	2437	O	PHE	A	315	-8.649	21.599	-0.679	1.00	15.00	A	O
ATOM	2438	N	VAL	A	316	-8.636	22.483	1.384	1.00	15.00	A	N
ATOM	2439	CA	VAL	A	316	-9.629	23.506	1.072	1.00	15.00	A	C
ATOM	2440	CB	VAL	A	316	-10.003	24.333	2.325	1.00	15.00	A	C
ATOM	2441	CG1	VAL	A	316	-10.987	25.442	1.978	1.00	15.00	A	C
ATOM	2442	CG2	VAL	A	316	-10.584	23.430	3.402	1.00	15.00	A	C
ATOM	2443	C	VAL	A	316	-9.145	24.439	-0.044	1.00	15.00	A	C
ATOM	2444	O	VAL	A	316	-9.930	24.866	-0.891	1.00	15.00	A	O
ATOM	2445	N	SER	A	317	-7.844	24.720	-0.062	1.00	15.00	A	N
ATOM	2446	CA	SER	A	317	-7.266	25.614	-1.063	1.00	15.00	A	C
ATOM	2447	CB	SER	A	317	-5.795	25.917	-0.751	1.00	15.00	A	C
ATOM	2448	OG	SER	A	317	-4.946	24.841	-1.122	1.00	15.00	A	O
ATOM	2449	C	SER	A	317	-7.420	25.089	-2.493	1.00	15.00	A	C
ATOM	2450	O	SER	A	317	-7.431	25.867	-3.444	1.00	15.00	A	O
ATOM	2451	N	VAL	A	318	-7.550	23.774	-2.649	1.00	15.00	A	N
ATOM	2452	CA	VAL	A	318	-7.691	23.190	-3.979	1.00	15.00	A	C
ATOM	2453	CB	VAL	A	318	-6.902	21.871	-4.148	1.00	15.00	A	C
ATOM	2454	CG1	VAL	A	318	-5.450	22.055	-3.733	1.00	15.00	A	C
ATOM	2455	CG2	VAL	A	318	-7.543	20.732	-3.374	1.00	15.00	A	C
ATOM	2456	C	VAL	A	318	-9.158	22.998	-4.367	1.00	15.00	A	C
ATOM	2457	O	VAL	A	318	-9.464	22.443	-5.422	1.00	15.00	A	O
ATOM	2458	N	GLY	A	319	-10.059	23.463	-3.509	1.00	15.00	A	N
ATOM	2459	CA	GLY	A	319	-11.478	23.345	-3.789	1.00	15.00	A	C
ATOM	2460	C	GLY	A	319	-12.126	22.166	-3.095	1.00	15.00	A	C
ATOM	2461	O	GLY	A	319	-13.299	21.874	-3.324	1.00	15.00	A	O
ATOM	2462	N	LEU	A	320	-11.371	21.486	-2.247	1.00	15.00	A	N
ATOM	2463	CA	LEU	A	320	-11.894	20.336	-1.526	1.00	15.00	A	C
ATOM	2464	CB	LEU	A	320	-10.796	19.293	-1.300	1.00	15.00	A	C
ATOM	2465	CG	LEU	A	320	-10.304	18.560	-2.552	1.00	15.00	A	C
ATOM	2466	CD1	LEU	A	320	-9.117	17.668	-2.224	1.00	15.00	A	C
ATOM	2467	CD2	LEU	A	320	-11.429	17.748	-3.177	1.00	15.00	A	C
ATOM	2468	C	LEU	A	320	-12.518	20.770	-0.203	1.00	15.00	A	C
ATOM	2469	O	LEU	A	320	-12.101	21.766	0.389	1.00	15.00	A	O
ATOM	2470	N	PRO	A	321	-13.542	20.040	0.266	1.00	15.00	A	N
ATOM	2471	CA	PRO	A	321	-14.228	20.360	1.521	1.00	15.00	A	C
ATOM	2472	CB	PRO	A	321	-15.397	19.370	1.556	1.00	15.00	A	C
ATOM	2473	CG	PRO	A	321	-14.963	18.240	0.690	1.00	15.00	A	C
ATOM	2474	CD	PRO	A	321	-14.113	18.850	-0.388	1.00	15.00	A	C
ATOM	2475	C	PRO	A	321	-13.334	20.165	2.744	1.00	15.00	A	C
ATOM	2476	O	PRO	A	321	-12.305	19.489	2.683	1.00	15.00	A	O
ATOM	2477	N	ASN	A	322	-13.742	20.765	3.854	1.00	15.00	A	N
ATOM	2478	CA	ASN	A	322	-12.998	20.665	5.102	1.00	15.00	A	C

ATOM	2479	CB	ASN	A	322	-13.249	21.888	5.999	1.00	15.00	A	C
ATOM	2480	CG	ASN	A	322	-14.718	22.263	6.138	1.00	15.00	A	C
ATOM	2481	OD1	ASN	A	322	-15.614	21.459	5.888	1.00	15.00	A	O
ATOM	2482	ND2	ASN	A	322	-14.975	23.500	6.538	1.00	15.00	A	N
ATOM	2483	C	ASN	A	322	-13.361	19.382	5.838	1.00	15.00	A	C
ATOM	2484	O	ASN	A	322	-14.115	18.553	5.322	1.00	15.00	A	O
ATOM	2485	N	MET	A	323	-12.827	19.214	7.037	1.00	15.00	A	N
ATOM	2486	CA	MET	A	323	-13.107	18.025	7.824	1.00	15.00	A	C
ATOM	2487	CB	MET	A	323	-11.955	17.725	8.784	1.00	15.00	A	C
ATOM	2488	CG	MET	A	323	-10.620	17.443	8.110	1.00	15.00	A	C
ATOM	2489	SD	MET	A	323	-10.538	15.808	7.346	1.00	15.00	A	S
ATOM	2490	CE	MET	A	323	-11.175	16.159	5.709	1.00	15.00	A	C
ATOM	2491	C	MET	A	323	-14.409	18.195	8.592	1.00	15.00	A	C
ATOM	2492	O	MET	A	323	-14.720	19.288	9.069	1.00	15.00	A	O
ATOM	2493	N	THR	A	324	-15.169	17.116	8.702	1.00	15.00	A	N
ATOM	2494	CA	THR	A	324	-16.440	17.142	9.408	1.00	15.00	A	C
ATOM	2495	CB	THR	A	324	-17.188	15.808	9.226	1.00	15.00	A	C
ATOM	2496	OG1	THR	A	324	-16.240	14.732	9.196	1.00	15.00	A	O
ATOM	2497	CG2	THR	A	324	-17.981	15.814	7.928	1.00	15.00	A	C
ATOM	2498	C	THR	A	324	-16.234	17.414	10.898	1.00	15.00	A	C
ATOM	2499	O	THR	A	324	-15.161	17.145	11.441	1.00	15.00	A	O
ATOM	2500	N	GLN	A	325	-17.262	17.947	11.554	1.00	15.00	A	N
ATOM	2501	CA	GLN	A	325	-17.186	18.251	12.980	1.00	15.00	A	C
ATOM	2502	CB	GLN	A	325	-18.512	18.831	13.481	1.00	15.00	A	C
ATOM	2503	CG	GLN	A	325	-18.445	19.382	14.896	1.00	15.00	A	C
ATOM	2504	CD	GLN	A	325	-19.807	19.742	15.456	1.00	15.00	A	C
ATOM	2505	OE1	GLN	A	325	-20.710	20.152	14.723	1.00	15.00	A	O
ATOM	2506	NE2	GLN	A	325	-19.971	19.573	16.758	1.00	15.00	A	N
ATOM	2507	C	GLN	A	325	-16.820	17.003	13.778	1.00	15.00	A	C
ATOM	2508	O	GLN	A	325	-15.902	17.028	14.597	1.00	15.00	A	O
ATOM	2509	N	GLY	A	326	-17.514	15.903	13.483	1.00	15.00	A	N
ATOM	2510	CA	GLY	A	326	-17.269	14.646	14.169	1.00	15.00	A	C
ATOM	2511	C	GLY	A	326	-15.836	14.170	14.036	1.00	15.00	A	C
ATOM	2512	O	GLY	A	326	-15.339	13.436	14.883	1.00	15.00	A	O
ATOM	2513	N	PHE	A	327	-15.164	14.596	12.975	1.00	15.00	A	N
ATOM	2514	CA	PHE	A	327	-13.782	14.211	12.755	1.00	15.00	A	C
ATOM	2515	CB	PHE	A	327	-13.323	14.625	11.351	1.00	15.00	A	C
ATOM	2516	CG	PHE	A	327	-11.837	14.521	11.136	1.00	15.00	A	C
ATOM	2517	CD1	PHE	A	327	-11.012	15.617	11.343	1.00	15.00	A	C
ATOM	2518	CD2	PHE	A	327	-11.266	13.328	10.730	1.00	15.00	A	C
ATOM	2519	CE1	PHE	A	327	-9.648	15.523	11.151	1.00	15.00	A	C
ATOM	2520	CE2	PHE	A	327	-9.903	13.228	10.535	1.00	15.00	A	C
ATOM	2521	CZ	PHE	A	327	-9.093	14.326	10.747	1.00	15.00	A	C
ATOM	2522	C	PHE	A	327	-12.893	14.863	13.803	1.00	15.00	A	C
ATOM	2523	O	PHE	A	327	-11.993	14.232	14.348	1.00	15.00	A	O
ATOM	2524	N	TRP	A	328	-13.173	16.121	14.100	1.00	15.00	A	N
ATOM	2525	CA	TRP	A	328	-12.385	16.870	15.066	1.00	15.00	A	C
ATOM	2526	CB	TRP	A	328	-12.537	18.380	14.830	1.00	15.00	A	C
ATOM	2527	CG	TRP	A	328	-11.905	18.856	13.549	1.00	15.00	A	C
ATOM	2528	CD1	TRP	A	328	-12.543	19.351	12.444	1.00	15.00	A	C
ATOM	2529	CD2	TRP	A	328	-10.505	18.877	13.241	1.00	15.00	A	C
ATOM	2530	NE1	TRP	A	328	-11.623	19.682	11.473	1.00	15.00	A	N
ATOM	2531	CE2	TRP	A	328	-10.368	19.394	11.937	1.00	15.00	A	C
ATOM	2532	CE3	TRP	A	328	-9.352	18.507	13.940	1.00	15.00	A	C
ATOM	2533	CZ2	TRP	A	328	-9.128	19.551	11.321	1.00	15.00	A	C
ATOM	2534	CZ3	TRP	A	328	-8.124	18.664	13.326	1.00	15.00	A	C
ATOM	2535	CH2	TRP	A	328	-8.022	19.181	12.030	1.00	15.00	A	C
ATOM	2536	C	TRP	A	328	-12.724	16.493	16.510	1.00	15.00	A	C
ATOM	2537	O	TRP	A	328	-11.970	16.805	17.430	1.00	15.00	A	O
ATOM	2538	N	GLU	A	329	-13.839	15.796	16.700	1.00	15.00	A	N
ATOM	2539	CA	GLU	A	329	-14.272	15.402	18.036	1.00	15.00	A	C
ATOM	2540	CB	GLU	A	329	-15.753	15.748	18.231	1.00	15.00	A	C
ATOM	2541	CG	GLU	A	329	-16.084	17.223	18.041	1.00	15.00	A	C
ATOM	2542	CD	GLU	A	329	-17.544	17.463	17.706	1.00	15.00	A	C
ATOM	2543	OE1	GLU	A	329	-18.173	18.324	18.361	1.00	15.00	A	O
ATOM	2544	OE2	GLU	A	329	-18.060	16.804	16.779	1.00	15.00	A	O
ATOM	2545	C	GLU	A	329	-14.054	13.911	18.318	1.00	15.00	A	C
ATOM	2546	O	GLU	A	329	-13.895	13.513	19.471	1.00	15.00	A	O
ATOM	2547	N	ASN	A	330	-14.020	13.091	17.272	1.00	15.00	A	N
ATOM	2548	CA	ASN	A	330	-13.872	11.643	17.448	1.00	15.00	A	C
ATOM	2549	CB	ASN	A	330	-14.992	10.905	16.706	1.00	15.00	A	C

ATOM	2550	CG	ASN	A	330	-16.309	10.920	17.454	1.00	15.00	A	C
ATOM	2551	OD1	ASN	A	330	-16.339	10.850	18.682	1.00	15.00	A	O
ATOM	2552	ND2	ASN	A	330	-17.409	11.003	16.721	1.00	15.00	A	N
ATOM	2553	C	ASN	A	330	-12.516	11.094	17.012	1.00	15.00	A	C
ATOM	2554	O	ASN	A	330	-12.331	9.876	16.953	1.00	15.00	A	O
ATOM	2555	N	SER	A	331	-11.566	11.965	16.720	1.00	15.00	A	N
ATOM	2556	CA	SER	A	331	-10.247	11.515	16.284	1.00	15.00	A	C
ATOM	2557	CB	SER	A	331	-9.809	12.235	15.009	1.00	15.00	A	C
ATOM	2558	OG	SER	A	331	-10.659	11.912	13.924	1.00	15.00	A	O
ATOM	2559	C	SER	A	331	-9.188	11.675	17.367	1.00	15.00	A	C
ATOM	2560	O	SER	A	331	-9.316	12.506	18.264	1.00	15.00	A	O
ATOM	2561	N	MET	A	332	-8.142	10.864	17.273	1.00	15.00	A	N
ATOM	2562	CA	MET	A	332	-7.042	10.913	18.221	1.00	15.00	A	C
ATOM	2563	CB	MET	A	332	-6.830	9.549	18.887	1.00	15.00	A	C
ATOM	2564	CG	MET	A	332	-5.763	9.545	19.973	1.00	15.00	A	C
ATOM	2565	SD	MET	A	332	-5.835	8.082	21.028	1.00	15.00	A	S
ATOM	2566	CE	MET	A	332	-7.205	8.504	22.102	1.00	15.00	A	C
ATOM	2567	C	MET	A	332	-5.786	11.352	17.485	1.00	15.00	A	C
ATOM	2568	O	MET	A	332	-5.105	10.539	16.860	1.00	15.00	A	O
ATOM	2569	N	LEU	A	333	-5.506	12.645	17.542	1.00	15.00	A	N
ATOM	2570	CA	LEU	A	333	-4.354	13.214	16.858	1.00	15.00	A	C
ATOM	2571	CB	LEU	A	333	-4.775	14.517	16.177	1.00	15.00	A	C
ATOM	2572	CG	LEU	A	333	-6.200	14.538	15.611	1.00	15.00	A	C
ATOM	2573	CD1	LEU	A	333	-6.626	15.956	15.272	1.00	15.00	A	C
ATOM	2574	CD2	LEU	A	333	-6.316	13.634	14.395	1.00	15.00	A	C
ATOM	2575	C	LEU	A	333	-3.220	13.476	17.842	1.00	15.00	A	C
ATOM	2576	O	LEU	A	333	-2.261	14.179	17.533	1.00	15.00	A	O
ATOM	2577	N	THR	A	334	-3.342	12.889	19.021	1.00	15.00	A	N
ATOM	2578	CA	THR	A	334	-2.356	13.047	20.074	1.00	15.00	A	C
ATOM	2579	CB	THR	A	334	-2.773	14.173	21.047	1.00	15.00	A	C
ATOM	2580	OG1	THR	A	334	-4.041	14.719	20.648	1.00	15.00	A	O
ATOM	2581	CG2	THR	A	334	-1.730	15.276	21.078	1.00	15.00	A	C
ATOM	2582	C	THR	A	334	-2.242	11.746	20.860	1.00	15.00	A	C
ATOM	2583	O	THR	A	334	-2.993	10.806	20.614	1.00	15.00	A	O
ATOM	2584	N	ASP	A	335	-1.306	11.696	21.798	1.00	15.00	A	N
ATOM	2585	CA	ASP	A	335	-1.116	10.511	22.627	1.00	15.00	A	C
ATOM	2586	CB	ASP	A	335	0.264	10.538	23.281	1.00	15.00	A	C
ATOM	2587	CG	ASP	A	335	1.189	9.475	22.732	1.00	15.00	A	C
ATOM	2588	OD1	ASP	A	335	1.263	8.384	23.330	1.00	15.00	A	O
ATOM	2589	OD2	ASP	A	335	1.854	9.743	21.703	1.00	15.00	A	O
ATOM	2590	C	ASP	A	335	-2.176	10.439	23.717	1.00	15.00	A	C
ATOM	2591	O	ASP	A	335	-2.613	11.470	24.235	1.00	15.00	A	O
ATOM	2592	N	PRO	A	336	-2.608	9.219	24.073	1.00	15.00	A	N
ATOM	2593	CA	PRO	A	336	-3.606	9.001	25.123	1.00	15.00	A	C
ATOM	2594	CB	PRO	A	336	-4.171	7.622	24.778	1.00	15.00	A	C
ATOM	2595	CG	PRO	A	336	-3.050	6.907	24.095	1.00	15.00	A	C
ATOM	2596	CD	PRO	A	336	-2.172	7.952	23.454	1.00	15.00	A	C
ATOM	2597	C	PRO	A	336	-2.964	8.981	26.512	1.00	15.00	A	C
ATOM	2598	O	PRO	A	336	-3.631	8.765	27.528	1.00	15.00	A	O
ATOM	2599	N	GLY	A	337	-1.658	9.201	26.544	1.00	15.00	A	N
ATOM	2600	CA	GLY	A	337	-0.935	9.216	27.794	1.00	15.00	A	C
ATOM	2601	C	GLY	A	337	-0.372	7.855	28.137	1.00	15.00	A	C
ATOM	2602	O	GLY	A	337	-0.421	6.934	27.328	1.00	15.00	A	O
ATOM	2603	N	ASN	A	338	0.153	7.720	29.345	1.00	15.00	A	N
ATOM	2604	CA	ASN	A	338	0.728	6.452	29.777	1.00	15.00	A	C
ATOM	2605	CB	ASN	A	338	1.966	6.680	30.653	1.00	15.00	A	C
ATOM	2606	CG	ASN	A	338	2.841	5.442	30.783	1.00	15.00	A	C
ATOM	2607	OD1	ASN	A	338	2.911	4.609	29.877	1.00	15.00	A	O
ATOM	2608	ND2	ASN	A	338	3.523	5.315	31.911	1.00	15.00	A	N
ATOM	2609	C	ASN	A	338	-0.303	5.598	30.509	1.00	15.00	A	C
ATOM	2610	O	ASN	A	338	-0.013	4.478	30.925	1.00	15.00	A	O
ATOM	2611	N	VAL	A	339	-1.511	6.130	30.655	1.00	15.00	A	N
ATOM	2612	CA	VAL	A	339	-2.583	5.409	31.334	1.00	15.00	A	C
ATOM	2613	CB	VAL	A	339	-3.674	6.370	31.856	1.00	15.00	A	C
ATOM	2614	CG1	VAL	A	339	-4.647	5.635	32.767	1.00	15.00	A	C
ATOM	2615	CG2	VAL	A	339	-3.047	7.551	32.582	1.00	15.00	A	C
ATOM	2616	C	VAL	A	339	-3.205	4.398	30.377	1.00	15.00	A	C
ATOM	2617	O	VAL	A	339	-3.150	3.191	30.606	1.00	15.00	A	O
ATOM	2618	N	GLN	A	340	-3.780	4.902	29.297	1.00	15.00	A	N
ATOM	2619	CA	GLN	A	340	-4.391	4.051	28.291	1.00	15.00	A	C
ATOM	2620	CB	GLN	A	340	-5.759	4.592	27.859	1.00	15.00	A	C

```
ATOM   2621  CG  GLN A 340      -5.863   6.109  27.822  1.00 15.00       A    C
ATOM   2622  CD  GLN A 340      -7.075   6.596  27.048  1.00 15.00       A    C
ATOM   2623  OE1 GLN A 340      -7.728   7.561  27.439  1.00 15.00       A    O
ATOM   2624  NE2 GLN A 340      -7.371   5.949  25.930  1.00 15.00       A    N
ATOM   2625  C   GLN A 340      -3.462   3.943  27.093  1.00 15.00       A    C
ATOM   2626  O   GLN A 340      -3.002   4.953  26.569  1.00 15.00       A    O
ATOM   2627  N   LYS A 341      -3.164   2.725  26.679  1.00 15.00       A    N
ATOM   2628  CA  LYS A 341      -2.279   2.516  25.546  1.00 15.00       A    C
ATOM   2629  CB  LYS A 341      -1.429   1.257  25.736  1.00 15.00       A    C
ATOM   2630  CG  LYS A 341      -0.465   1.342  26.911  1.00 15.00       A    C
ATOM   2631  CD  LYS A 341       0.327   2.641  26.878  1.00 15.00       A    C
ATOM   2632  CE  LYS A 341       1.011   2.909  28.206  1.00 15.00       A    C
ATOM   2633  NZ  LYS A 341       2.350   2.266  28.291  1.00 15.00       A    N
ATOM   2634  C   LYS A 341      -3.066   2.452  24.248  1.00 15.00       A    C
ATOM   2635  O   LYS A 341      -4.191   1.962  24.223  1.00 15.00       A    O
ATOM   2636  N   ALA A 342      -2.475   2.955  23.179  1.00 15.00       A    N
ATOM   2637  CA  ALA A 342      -3.125   2.958  21.881  1.00 15.00       A    C
ATOM   2638  CB  ALA A 342      -3.717   4.331  21.599  1.00 15.00       A    C
ATOM   2639  C   ALA A 342      -2.129   2.590  20.794  1.00 15.00       A    C
ATOM   2640  O   ALA A 342      -0.920   2.554  21.033  1.00 15.00       A    O
ATOM   2641  N   VAL A 343      -2.638   2.295  19.612  1.00 15.00       A    N
ATOM   2642  CA  VAL A 343      -1.791   1.950  18.485  1.00 15.00       A    C
ATOM   2643  CB  VAL A 343      -2.529   1.042  17.476  1.00 15.00       A    C
ATOM   2644  CG1 VAL A 343      -1.600   0.623  16.345  1.00 15.00       A    C
ATOM   2645  CG2 VAL A 343      -3.104  -0.181  18.177  1.00 15.00       A    C
ATOM   2646  C   VAL A 343      -1.340   3.228  17.786  1.00 15.00       A    C
ATOM   2647  O   VAL A 343      -2.113   3.854  17.063  1.00 15.00       A    O
ATOM   2648  N   CYS A 344      -0.094   3.622  18.016  1.00 15.00       A    N
ATOM   2649  CA  CYS A 344       0.447   4.835  17.409  1.00 15.00       A    C
ATOM   2650  C   CYS A 344       0.837   4.629  15.946  1.00 15.00       A    C
ATOM   2651  O   CYS A 344       1.911   5.045  15.512  1.00 15.00       A    O
ATOM   2652  CB  CYS A 344       1.631   5.374  18.216  1.00 15.00       A    C
ATOM   2653  SG  CYS A 344       1.165   6.174  19.785  1.00 15.00       A    S
ATOM   2654  N   HIS A 345      -0.046   3.998  15.188  1.00 15.00       A    N
ATOM   2655  CA  HIS A 345       0.191   3.754  13.776  1.00 15.00       A    C
ATOM   2656  CB  HIS A 345       0.064   2.259  13.449  1.00 15.00       A    C
ATOM   2657  CG  HIS A 345       0.388   1.911  12.023  1.00 15.00       A    C
ATOM   2658  ND1 HIS A 345      -0.497   1.214  11.236  1.00 15.00       A    N
ATOM   2659  CD2 HIS A 345       1.502   2.191  11.297  1.00 15.00       A    C
ATOM   2660  CE1 HIS A 345       0.084   1.086  10.059  1.00 15.00       A    C
ATOM   2661  NE2 HIS A 345       1.293   1.660  10.049  1.00 15.00       A    N
ATOM   2662  C   HIS A 345      -0.800   4.575  12.961  1.00 15.00       A    C
ATOM   2663  O   HIS A 345      -2.010   4.357  13.047  1.00 15.00       A    O
ATOM   2664  N   PRO A 346      -0.294   5.554  12.193  1.00 15.00       A    N
ATOM   2665  CA  PRO A 346      -1.130   6.431  11.364  1.00 15.00       A    C
ATOM   2666  CB  PRO A 346      -0.109   7.199  10.522  1.00 15.00       A    C
ATOM   2667  CG  PRO A 346       1.132   7.191  11.344  1.00 15.00       A    C
ATOM   2668  CD  PRO A 346       1.139   5.875  12.066  1.00 15.00       A    C
ATOM   2669  C   PRO A 346      -2.061   5.639  10.458  1.00 15.00       A    C
ATOM   2670  O   PRO A 346      -1.608   4.885   9.597  1.00 15.00       A    O
ATOM   2671  N   THR A 347      -3.360   5.790  10.681  1.00 15.00       A    N
ATOM   2672  CA  THR A 347      -4.351   5.090   9.885  1.00 15.00       A    C
ATOM   2673  CB  THR A 347      -4.729   3.738  10.530  1.00 15.00       A    C
ATOM   2674  OG1 THR A 347      -4.650   3.838  11.963  1.00 15.00       A    O
ATOM   2675  CG2 THR A 347      -3.815   2.621  10.043  1.00 15.00       A    C
ATOM   2676  C   THR A 347      -5.623   5.917   9.710  1.00 15.00       A    C
ATOM   2677  O   THR A 347      -6.089   6.571  10.651  1.00 15.00       A    O
ATOM   2678  N   ALA A 348      -6.154   5.914   8.493  1.00 15.00       A    N
ATOM   2679  CA  ALA A 348      -7.399   6.605   8.191  1.00 15.00       A    C
ATOM   2680  CB  ALA A 348      -7.471   6.940   6.711  1.00 15.00       A    C
ATOM   2681  C   ALA A 348      -8.542   5.680   8.583  1.00 15.00       A    C
ATOM   2682  O   ALA A 348      -8.489   4.479   8.309  1.00 15.00       A    O
ATOM   2683  N   TRP A 349      -9.568   6.213   9.223  1.00 15.00       A    N
ATOM   2684  CA  TRP A 349     -10.667   5.379   9.672  1.00 15.00       A    C
ATOM   2685  CB  TRP A 349     -10.687   5.303  11.203  1.00 15.00       A    C
ATOM   2686  CG  TRP A 349      -9.614   4.441  11.791  1.00 15.00       A    C
ATOM   2687  CD1 TRP A 349      -8.281   4.717  11.850  1.00 15.00       A    C
ATOM   2688  CD2 TRP A 349      -9.788   3.166  12.417  1.00 15.00       A    C
ATOM   2689  NE1 TRP A 349      -7.610   3.689  12.462  1.00 15.00       A    N
ATOM   2690  CE2 TRP A 349      -8.513   2.725  12.822  1.00 15.00       A    C
ATOM   2691  CE3 TRP A 349     -10.897   2.354  12.670  1.00 15.00       A    C
```

```
ATOM   2692  CZ2 TRP A 349      -8.318   1.508  13.468  1.00 15.00      A    C
ATOM   2693  CZ3 TRP A 349     -10.702   1.148  13.311  1.00 15.00      A    C
ATOM   2694  CH2 TRP A 349      -9.423   0.736  13.703  1.00 15.00      A    C
ATOM   2695  C   TRP A 349     -12.036   5.818   9.179  1.00 15.00      A    C
ATOM   2696  O   TRP A 349     -12.581   6.823   9.640  1.00 15.00      A    O
ATOM   2697  N   ASP A 350     -12.578   5.067   8.233  1.00 15.00      A    N
ATOM   2698  CA  ASP A 350     -13.926   5.308   7.748  1.00 15.00      A    C
ATOM   2699  CB  ASP A 350     -14.046   5.211   6.225  1.00 15.00      A    C
ATOM   2700  CG  ASP A 350     -15.505   5.142   5.781  1.00 15.00      A    C
ATOM   2701  OD1 ASP A 350     -16.349   5.856   6.378  1.00 15.00      A    O
ATOM   2702  OD2 ASP A 350     -15.815   4.366   4.853  1.00 15.00      A    O
ATOM   2703  C   ASP A 350     -14.804   4.255   8.394  1.00 15.00      A    C
ATOM   2704  O   ASP A 350     -14.650   3.061   8.135  1.00 15.00      A    O
ATOM   2705  N   LEU A 351     -15.683   4.687   9.268  1.00 15.00      A    N
ATOM   2706  CA  LEU A 351     -16.554   3.768   9.971  1.00 15.00      A    C
ATOM   2707  CB  LEU A 351     -16.704   4.198  11.436  1.00 15.00      A    C
ATOM   2708  CG  LEU A 351     -15.466   4.075  12.334  1.00 15.00      A    C
ATOM   2709  CD1 LEU A 351     -14.477   5.199  12.070  1.00 15.00      A    C
ATOM   2710  CD2 LEU A 351     -15.875   4.061  13.797  1.00 15.00      A    C
ATOM   2711  C   LEU A 351     -17.920   3.709   9.312  1.00 15.00      A    C
ATOM   2712  O   LEU A 351     -18.814   3.006   9.781  1.00 15.00      A    O
ATOM   2713  N   GLY A 352     -18.071   4.423   8.208  1.00 15.00      A    N
ATOM   2714  CA  GLY A 352     -19.348   4.460   7.540  1.00 15.00      A    C
ATOM   2715  C   GLY A 352     -20.275   5.426   8.247  1.00 15.00      A    C
ATOM   2716  O   GLY A 352     -19.888   6.036   9.247  1.00 15.00      A    O
ATOM   2717  N   LYS A 353     -21.488   5.585   7.732  1.00 15.00      A    N
ATOM   2718  CA  LYS A 353     -22.471   6.498   8.323  1.00 15.00      A    C
ATOM   2719  CB  LYS A 353     -22.887   6.053   9.728  1.00 15.00      A    C
ATOM   2720  CG  LYS A 353     -23.767   4.817   9.757  1.00 15.00      A    C
ATOM   2721  CD  LYS A 353     -24.422   4.645  11.116  1.00 15.00      A    C
ATOM   2722  CE  LYS A 353     -25.249   3.374  11.173  1.00 15.00      A    C
ATOM   2723  NZ  LYS A 353     -26.025   3.280  12.437  1.00 15.00      A    N
ATOM   2724  C   LYS A 353     -21.988   7.952   8.340  1.00 15.00      A    C
ATOM   2725  O   LYS A 353     -22.644   8.825   8.912  1.00 15.00      A    O
ATOM   2726  N   GLY A 354     -20.858   8.210   7.693  1.00 15.00      A    N
ATOM   2727  CA  GLY A 354     -20.319   9.555   7.640  1.00 15.00      A    C
ATOM   2728  C   GLY A 354     -19.333   9.832   8.752  1.00 15.00      A    C
ATOM   2729  O   GLY A 354     -18.892  10.968   8.932  1.00 15.00      A    O
ATOM   2730  N   ASP A 355     -18.990   8.797   9.502  1.00 15.00      A    N
ATOM   2731  CA  ASP A 355     -18.048   8.934  10.603  1.00 15.00      A    C
ATOM   2732  CB  ASP A 355     -18.424   7.996  11.755  1.00 15.00      A    C
ATOM   2733  CG  ASP A 355     -17.679   8.293  13.042  1.00 15.00      A    C
ATOM   2734  OD1 ASP A 355     -17.510   9.482  13.381  1.00 15.00      A    O
ATOM   2735  OD2 ASP A 355     -17.274   7.331  13.731  1.00 15.00      A    O
ATOM   2736  C   ASP A 355     -16.619   8.679  10.132  1.00 15.00      A    C
ATOM   2737  O   ASP A 355     -16.171   7.535  10.021  1.00 15.00      A    O
ATOM   2738  N   PHE A 356     -15.926   9.765   9.821  1.00 15.00      A    N
ATOM   2739  CA  PHE A 356     -14.547   9.705   9.361  1.00 15.00      A    C
ATOM   2740  CB  PHE A 356     -14.362  10.571   8.108  1.00 15.00      A    C
ATOM   2741  CG  PHE A 356     -15.319  10.247   6.998  1.00 15.00      A    C
ATOM   2742  CD1 PHE A 356     -15.083   9.179   6.150  1.00 15.00      A    C
ATOM   2743  CD2 PHE A 356     -16.457  11.011   6.804  1.00 15.00      A    C
ATOM   2744  CE1 PHE A 356     -15.965   8.877   5.129  1.00 15.00      A    C
ATOM   2745  CE2 PHE A 356     -17.345  10.717   5.786  1.00 15.00      A    C
ATOM   2746  CZ  PHE A 356     -17.099   9.647   4.946  1.00 15.00      A    C
ATOM   2747  C   PHE A 356     -13.614  10.183  10.463  1.00 15.00      A    C
ATOM   2748  O   PHE A 356     -13.762  11.292  10.970  1.00 15.00      A    O
ATOM   2749  N   ARG A 357     -12.653   9.348  10.831  1.00 15.00      A    N
ATOM   2750  CA  ARG A 357     -11.703   9.688  11.893  1.00 15.00      A    C
ATOM   2751  CB  ARG A 357     -12.100   8.991  13.200  1.00 15.00      A    C
ATOM   2752  CG  ARG A 357     -13.520   9.283  13.651  1.00 15.00      A    C
ATOM   2753  CD  ARG A 357     -14.215   8.039  14.169  1.00 15.00      A    C
ATOM   2754  NE  ARG A 357     -13.974   7.846  15.594  1.00 15.00      A    N
ATOM   2755  CZ  ARG A 357     -14.893   7.451  16.463  1.00 15.00      A    C
ATOM   2756  NH1 ARG A 357     -16.128   7.198  16.070  1.00 15.00      A    N
ATOM   2757  NH2 ARG A 357     -14.574   7.312  17.739  1.00 15.00      A    N
ATOM   2758  C   ARG A 357     -10.285   9.280  11.519  1.00 15.00      A    C
ATOM   2759  O   ARG A 357     -10.089   8.508  10.586  1.00 15.00      A    O
ATOM   2760  N   ILE A 358      -9.308   9.806  12.252  1.00 15.00      A    N
ATOM   2761  CA  ILE A 358      -7.904   9.487  12.024  1.00 15.00      A    C
ATOM   2762  CB  ILE A 358      -7.154  10.668  11.371  1.00 15.00      A    C
```

ATOM	2763	CG1	ILE	A	358	-7.461	10.736	9.879	1.00	15.00	A	C
ATOM	2764	CG2	ILE	A	358	-5.651	10.587	11.610	1.00	15.00	A	C
ATOM	2765	CD1	ILE	A	358	-6.811	11.905	9.175	1.00	15.00	A	C
ATOM	2766	C	ILE	A	358	-7.221	9.108	13.332	1.00	15.00	A	C
ATOM	2767	O	ILE	A	358	-7.444	9.740	14.365	1.00	15.00	A	O
ATOM	2768	N	LEU	A	359	-6.394	8.076	13.282	1.00	15.00	A	N
ATOM	2769	CA	LEU	A	359	-5.674	7.614	14.455	1.00	15.00	A	C
ATOM	2770	CB	LEU	A	359	-5.964	6.136	14.688	1.00	15.00	A	C
ATOM	2771	CG	LEU	A	359	-6.214	5.709	16.133	1.00	15.00	A	C
ATOM	2772	CD1	LEU	A	359	-6.681	4.265	16.172	1.00	15.00	A	C
ATOM	2773	CD2	LEU	A	359	-4.964	5.891	16.980	1.00	15.00	A	C
ATOM	2774	C	LEU	A	359	-4.177	7.807	14.260	1.00	15.00	A	C
ATOM	2775	O	LEU	A	359	-3.571	7.198	13.373	1.00	15.00	A	O
ATOM	2776	N	MET	A	360	-3.580	8.652	15.086	1.00	15.00	A	N
ATOM	2777	CA	MET	A	360	-2.158	8.914	14.995	1.00	15.00	A	C
ATOM	2778	CB	MET	A	360	-1.839	9.666	13.700	1.00	15.00	A	C
ATOM	2779	CG	MET	A	360	-0.357	9.896	13.455	1.00	15.00	A	C
ATOM	2780	SD	MET	A	360	-0.042	11.268	12.327	1.00	15.00	A	S
ATOM	2781	CE	MET	A	360	-1.070	10.811	10.935	1.00	15.00	A	C
ATOM	2782	C	MET	A	360	-1.687	9.742	16.173	1.00	15.00	A	C
ATOM	2783	O	MET	A	360	-2.266	10.780	16.479	1.00	15.00	A	O
ATOM	2784	N	CYS	A	361	-0.645	9.276	16.837	1.00	15.00	A	N
ATOM	2785	CA	CYS	A	361	-0.074	10.003	17.958	1.00	15.00	A	C
ATOM	2786	C	CYS	A	361	0.905	11.033	17.401	1.00	15.00	A	C
ATOM	2787	O	CYS	A	361	2.111	10.958	17.639	1.00	15.00	A	O
ATOM	2788	CB	CYS	A	361	0.648	9.036	18.899	1.00	15.00	A	C
ATOM	2789	SG	CYS	A	361	-0.274	7.502	19.245	1.00	15.00	A	S
ATOM	2790	N	THR	A	362	0.359	11.964	16.630	1.00	15.00	A	N
ATOM	2791	CA	THR	A	362	1.125	13.017	15.976	1.00	15.00	A	C
ATOM	2792	CB	THR	A	362	0.172	13.978	15.245	1.00	15.00	A	C
ATOM	2793	OG1	THR	A	362	-0.855	13.202	14.608	1.00	15.00	A	O
ATOM	2794	CG2	THR	A	362	0.917	14.778	14.188	1.00	15.00	A	C
ATOM	2795	C	THR	A	362	2.035	13.804	16.924	1.00	15.00	A	C
ATOM	2796	O	THR	A	362	1.616	14.257	17.996	1.00	15.00	A	O
ATOM	2797	N	LYS	A	363	3.293	13.937	16.518	1.00	15.00	A	N
ATOM	2798	CA	LYS	A	363	4.290	14.676	17.274	1.00	15.00	A	C
ATOM	2799	CB	LYS	A	363	5.579	13.852	17.391	1.00	15.00	A	C
ATOM	2800	CG	LYS	A	363	5.367	12.404	17.792	1.00	15.00	A	C
ATOM	2801	CD	LYS	A	363	4.974	12.296	19.250	1.00	15.00	A	C
ATOM	2802	CE	LYS	A	363	4.518	10.893	19.598	1.00	15.00	A	C
ATOM	2803	NZ	LYS	A	363	3.941	10.847	20.962	1.00	15.00	A	N
ATOM	2804	C	LYS	A	363	4.599	15.981	16.550	1.00	15.00	A	C
ATOM	2805	O	LYS	A	363	4.106	16.217	15.447	1.00	15.00	A	O
ATOM	2806	N	VAL	A	364	5.420	16.823	17.161	1.00	15.00	A	N
ATOM	2807	CA	VAL	A	364	5.794	18.095	16.558	1.00	15.00	A	C
ATOM	2808	CB	VAL	A	364	6.032	19.180	17.634	1.00	15.00	A	C
ATOM	2809	CG1	VAL	A	364	6.201	20.553	16.998	1.00	15.00	A	C
ATOM	2810	CG2	VAL	A	364	4.882	19.193	18.631	1.00	15.00	A	C
ATOM	2811	C	VAL	A	364	7.051	17.891	15.719	1.00	15.00	A	C
ATOM	2812	O	VAL	A	364	8.143	18.335	16.076	1.00	15.00	A	O
ATOM	2813	N	THR	A	365	6.882	17.179	14.617	1.00	15.00	A	N
ATOM	2814	CA	THR	A	365	7.979	16.862	13.724	1.00	15.00	A	C
ATOM	2815	CB	THR	A	365	8.387	15.389	13.934	1.00	15.00	A	C
ATOM	2816	OG1	THR	A	365	7.275	14.681	14.513	1.00	15.00	A	O
ATOM	2817	CG2	THR	A	365	9.574	15.303	14.883	1.00	15.00	A	C
ATOM	2818	C	THR	A	365	7.571	17.067	12.266	1.00	15.00	A	C
ATOM	2819	O	THR	A	365	6.384	17.006	11.931	1.00	15.00	A	O
ATOM	2820	N	MET	A	366	8.555	17.323	11.404	1.00	15.00	A	N
ATOM	2821	CA	MET	A	366	8.295	17.527	9.981	1.00	15.00	A	C
ATOM	2822	CB	MET	A	366	9.577	17.928	9.240	1.00	15.00	A	C
ATOM	2823	CG	MET	A	366	9.395	18.123	7.742	1.00	15.00	A	C
ATOM	2824	SD	MET	A	366	10.587	19.277	7.037	1.00	15.00	A	S
ATOM	2825	CE	MET	A	366	10.152	19.194	5.301	1.00	15.00	A	C
ATOM	2826	C	MET	A	366	7.676	16.282	9.350	1.00	15.00	A	C
ATOM	2827	O	MET	A	366	6.756	16.381	8.536	1.00	15.00	A	O
ATOM	2828	N	ASP	A	367	8.175	15.113	9.741	1.00	15.00	A	N
ATOM	2829	CA	ASP	A	367	7.662	13.848	9.220	1.00	15.00	A	C
ATOM	2830	CB	ASP	A	367	8.472	12.662	9.749	1.00	15.00	A	C
ATOM	2831	CG	ASP	A	367	9.880	12.616	9.187	1.00	15.00	A	C
ATOM	2832	OD1	ASP	A	367	10.027	12.485	7.953	1.00	15.00	A	O
ATOM	2833	OD2	ASP	A	367	10.838	12.720	9.981	1.00	15.00	A	O

ATOM	2834	C	ASP	A	367	6.185	13.675	9.556	1.00	15.00	A	C
ATOM	2835	O	ASP	A	367	5.376	13.341	8.689	1.00	15.00	A	O
ATOM	2836	N	ASP	A	368	5.832	13.924	10.815	1.00	15.00	A	N
ATOM	2837	CA	ASP	A	368	4.444	13.808	11.263	1.00	15.00	A	C
ATOM	2838	CB	ASP	A	368	4.330	14.067	12.765	1.00	15.00	A	C
ATOM	2839	CG	ASP	A	368	4.727	12.868	13.596	1.00	15.00	A	C
ATOM	2840	OD1	ASP	A	368	5.896	12.439	13.490	1.00	15.00	A	O
ATOM	2841	OD2	ASP	A	368	3.879	12.371	14.367	1.00	15.00	A	O
ATOM	2842	C	ASP	A	368	3.561	14.786	10.507	1.00	15.00	A	C
ATOM	2843	O	ASP	A	368	2.422	14.477	10.157	1.00	15.00	A	O
ATOM	2844	N	PHE	A	369	4.108	15.967	10.249	1.00	15.00	A	N
ATOM	2845	CA	PHE	A	369	3.399	17.002	9.514	1.00	15.00	A	C
ATOM	2846	CB	PHE	A	369	4.271	18.262	9.434	1.00	15.00	A	C
ATOM	2847	CG	PHE	A	369	3.737	19.341	8.540	1.00	15.00	A	C
ATOM	2848	CD1	PHE	A	369	2.660	20.118	8.932	1.00	15.00	A	C
ATOM	2849	CD2	PHE	A	369	4.320	19.583	7.306	1.00	15.00	A	C
ATOM	2850	CE1	PHE	A	369	2.173	21.115	8.110	1.00	15.00	A	C
ATOM	2851	CE2	PHE	A	369	3.838	20.577	6.478	1.00	15.00	A	C
ATOM	2852	CZ	PHE	A	369	2.763	21.345	6.881	1.00	15.00	A	C
ATOM	2853	C	PHE	A	369	3.040	16.498	8.115	1.00	15.00	A	C
ATOM	2854	O	PHE	A	369	1.942	16.744	7.611	1.00	15.00	A	O
ATOM	2855	N	LEU	A	370	3.968	15.770	7.502	1.00	15.00	A	N
ATOM	2856	CA	LEU	A	370	3.751	15.218	6.171	1.00	15.00	A	C
ATOM	2857	CB	LEU	A	370	5.077	14.787	5.540	1.00	15.00	A	C
ATOM	2858	CG	LEU	A	370	6.090	15.902	5.267	1.00	15.00	A	C
ATOM	2859	CD1	LEU	A	370	7.362	15.331	4.660	1.00	15.00	A	C
ATOM	2860	CD2	LEU	A	370	5.489	16.964	4.359	1.00	15.00	A	C
ATOM	2861	C	LEU	A	370	2.787	14.038	6.231	1.00	15.00	A	C
ATOM	2862	O	LEU	A	370	1.894	13.913	5.394	1.00	15.00	A	O
ATOM	2863	N	THR	A	371	2.962	13.189	7.238	1.00	15.00	A	N
ATOM	2864	CA	THR	A	371	2.108	12.020	7.417	1.00	15.00	A	C
ATOM	2865	CB	THR	A	371	2.589	11.149	8.595	1.00	15.00	A	C
ATOM	2866	OG1	THR	A	371	4.007	10.956	8.487	1.00	15.00	A	O
ATOM	2867	CG2	THR	A	371	1.905	9.791	8.569	1.00	15.00	A	C
ATOM	2868	C	THR	A	371	0.650	12.432	7.628	1.00	15.00	A	C
ATOM	2869	O	THR	A	371	-0.272	11.741	7.194	1.00	15.00	A	O
ATOM	2870	N	ALA	A	372	0.447	13.571	8.287	1.00	15.00	A	N
ATOM	2871	CA	ALA	A	372	-0.896	14.084	8.531	1.00	15.00	A	C
ATOM	2872	CB	ALA	A	372	-0.836	15.359	9.358	1.00	15.00	A	C
ATOM	2873	C	ALA	A	372	-1.612	14.339	7.207	1.00	15.00	A	C
ATOM	2874	O	ALA	A	372	-2.821	14.138	7.091	1.00	15.00	A	O
ATOM	2875	N	HIS	A	373	-0.846	14.762	6.207	1.00	15.00	A	N
ATOM	2876	CA	HIS	A	373	-1.388	15.038	4.882	1.00	15.00	A	C
ATOM	2877	CB	HIS	A	373	-0.396	15.862	4.057	1.00	15.00	A	C
ATOM	2878	CG	HIS	A	373	-0.362	17.314	4.420	1.00	15.00	A	C
ATOM	2879	ND1	HIS	A	373	0.418	17.843	5.428	1.00	15.00	A	N
ATOM	2880	CD2	HIS	A	373	-1.037	18.360	3.885	1.00	15.00	A	C
ATOM	2881	CE1	HIS	A	373	0.196	19.164	5.469	1.00	15.00	A	C
ATOM	2882	NE2	HIS	A	373	-0.680	19.527	4.552	1.00	15.00	A	N
ATOM	2883	C	HIS	A	373	-1.722	13.737	4.161	1.00	15.00	A	C
ATOM	2884	O	HIS	A	373	-2.712	13.656	3.435	1.00	15.00	A	O
ATOM	2885	N	HIS	A	374	-0.891	12.721	4.377	1.00	15.00	A	N
ATOM	2886	CA	HIS	A	374	-1.092	11.410	3.766	1.00	15.00	A	C
ATOM	2887	CB	HIS	A	374	0.087	10.489	4.119	1.00	15.00	A	C
ATOM	2888	CG	HIS	A	374	0.157	9.205	3.338	1.00	15.00	A	C
ATOM	2889	ND1	HIS	A	374	0.972	9.083	2.236	1.00	15.00	A	N
ATOM	2890	CD2	HIS	A	374	-0.473	8.021	3.559	1.00	15.00	A	C
ATOM	2891	CE1	HIS	A	374	0.826	7.837	1.815	1.00	15.00	A	C
ATOM	2892	NE2	HIS	A	374	-0.036	7.160	2.582	1.00	15.00	A	N
ATOM	2893	C	HIS	A	374	-2.401	10.806	4.266	1.00	15.00	A	C
ATOM	2894	O	HIS	A	374	-3.259	10.407	3.475	1.00	15.00	A	O
ATOM	2895	N	GLU	A	375	-2.559	10.784	5.585	1.00	15.00	A	N
ATOM	2896	CA	GLU	A	375	-3.755	10.236	6.212	1.00	15.00	A	C
ATOM	2897	CB	GLU	A	375	-3.597	10.184	7.732	1.00	15.00	A	C
ATOM	2898	CG	GLU	A	375	-2.548	9.193	8.202	1.00	15.00	A	C
ATOM	2899	CD	GLU	A	375	-2.611	7.887	7.438	1.00	15.00	A	C
ATOM	2900	OE1	GLU	A	375	-3.635	7.182	7.553	1.00	15.00	A	O
ATOM	2901	OE2	GLU	A	375	-1.642	7.576	6.722	1.00	15.00	A	O
ATOM	2902	C	GLU	A	375	-5.004	11.022	5.834	1.00	15.00	A	C
ATOM	2903	O	GLU	A	375	-6.047	10.441	5.533	1.00	15.00	A	O
ATOM	2904	N	MET	A	376	-4.900	12.348	5.842	1.00	15.00	A	N

```
ATOM   2905  CA  MET A 376      -6.035  13.188   5.483  1.00 15.00       A    C
ATOM   2906  CB  MET A 376      -5.770  14.662   5.798  1.00 15.00       A    C
ATOM   2907  CG  MET A 376      -7.012  15.537   5.711  1.00 15.00       A    C
ATOM   2908  SD  MET A 376      -6.742  17.204   6.339  1.00 15.00       A    S
ATOM   2909  CE  MET A 376      -6.559  16.883   8.092  1.00 15.00       A    C
ATOM   2910  C   MET A 376      -6.391  12.997   4.013  1.00 15.00       A    C
ATOM   2911  O   MET A 376      -7.544  13.170   3.613  1.00 15.00       A    O
ATOM   2912  N   GLY A 377      -5.390  12.631   3.215  1.00 15.00       A    N
ATOM   2913  CA  GLY A 377      -5.609  12.386   1.805  1.00 15.00       A    C
ATOM   2914  C   GLY A 377      -6.540  11.211   1.602  1.00 15.00       A    C
ATOM   2915  O   GLY A 377      -7.387  11.225   0.708  1.00 15.00       A    O
ATOM   2916  N   HIS A 378      -6.386  10.197   2.447  1.00 15.00       A    N
ATOM   2917  CA  HIS A 378      -7.232   9.014   2.384  1.00 15.00       A    C
ATOM   2918  CB  HIS A 378      -6.709   7.913   3.312  1.00 15.00       A    C
ATOM   2919  CG  HIS A 378      -5.501   7.193   2.796  1.00 15.00       A    C
ATOM   2920  ND1 HIS A 378      -5.485   6.626   1.543  1.00 15.00       A    N
ATOM   2921  CD2 HIS A 378      -4.306   6.971   3.402  1.00 15.00       A    C
ATOM   2922  CE1 HIS A 378      -4.292   6.074   1.414  1.00 15.00       A    C
ATOM   2923  NE2 HIS A 378      -3.548   6.257   2.509  1.00 15.00       A    N
ATOM   2924  C   HIS A 378      -8.659   9.383   2.763  1.00 15.00       A    C
ATOM   2925  O   HIS A 378      -9.603   9.032   2.064  1.00 15.00       A    O
ATOM   2926  N   ILE A 379      -8.800  10.132   3.857  1.00 15.00       A    N
ATOM   2927  CA  ILE A 379     -10.114  10.572   4.329  1.00 15.00       A    C
ATOM   2928  CB  ILE A 379      -9.998  11.406   5.627  1.00 15.00       A    C
ATOM   2929  CG1 ILE A 379      -9.449  10.547   6.771  1.00 15.00       A    C
ATOM   2930  CG2 ILE A 379     -11.337  12.025   6.015  1.00 15.00       A    C
ATOM   2931  CD1 ILE A 379     -10.343   9.386   7.157  1.00 15.00       A    C
ATOM   2932  C   ILE A 379     -10.823  11.386   3.247  1.00 15.00       A    C
ATOM   2933  O   ILE A 379     -12.042  11.307   3.088  1.00 15.00       A    O
ATOM   2934  N   GLN A 380     -10.047  12.154   2.489  1.00 15.00       A    N
ATOM   2935  CA  GLN A 380     -10.595  12.964   1.411  1.00 15.00       A    C
ATOM   2936  CB  GLN A 380      -9.516  13.877   0.826  1.00 15.00       A    C
ATOM   2937  CG  GLN A 380     -10.070  15.022  -0.003  1.00 15.00       A    C
ATOM   2938  CD  GLN A 380     -10.880  16.008   0.820  1.00 15.00       A    C
ATOM   2939  OE1 GLN A 380     -11.879  16.547   0.350  1.00 15.00       A    O
ATOM   2940  NE2 GLN A 380     -10.448  16.261   2.046  1.00 15.00       A    N
ATOM   2941  C   GLN A 380     -11.179  12.063   0.325  1.00 15.00       A    C
ATOM   2942  O   GLN A 380     -12.171  12.406  -0.317  1.00 15.00       A    O
ATOM   2943  N   TYR A 381     -10.555  10.907   0.138  1.00 15.00       A    N
ATOM   2944  CA  TYR A 381     -11.008   9.930  -0.842  1.00 15.00       A    C
ATOM   2945  CB  TYR A 381      -9.940   8.835  -0.990  1.00 15.00       A    C
ATOM   2946  CG  TYR A 381      -9.950   8.060  -2.296  1.00 15.00       A    C
ATOM   2947  CD1 TYR A 381     -11.099   7.943  -3.071  1.00 15.00       A    C
ATOM   2948  CD2 TYR A 381      -8.796   7.432  -2.746  1.00 15.00       A    C
ATOM   2949  CE1 TYR A 381     -11.093   7.226  -4.255  1.00 15.00       A    C
ATOM   2950  CE2 TYR A 381      -8.781   6.716  -3.927  1.00 15.00       A    C
ATOM   2951  CZ  TYR A 381      -9.930   6.615  -4.678  1.00 15.00       A    C
ATOM   2952  OH  TYR A 381      -9.916   5.899  -5.856  1.00 15.00       A    O
ATOM   2953  C   TYR A 381     -12.308   9.315  -0.336  1.00 15.00       A    C
ATOM   2954  O   TYR A 381     -13.300   9.238  -1.062  1.00 15.00       A    O
ATOM   2955  N   ASP A 382     -12.288   8.912   0.930  1.00 15.00       A    N
ATOM   2956  CA  ASP A 382     -13.443   8.301   1.583  1.00 15.00       A    C
ATOM   2957  CB  ASP A 382     -13.116   7.949   3.041  1.00 15.00       A    C
ATOM   2958  CG  ASP A 382     -11.836   7.148   3.193  1.00 15.00       A    C
ATOM   2959  OD1 ASP A 382     -11.518   6.350   2.284  1.00 15.00       A    O
ATOM   2960  OD2 ASP A 382     -11.139   7.331   4.216  1.00 15.00       A    O
ATOM   2961  C   ASP A 382     -14.657   9.222   1.537  1.00 15.00       A    C
ATOM   2962  O   ASP A 382     -15.752   8.808   1.156  1.00 15.00       A    O
ATOM   2963  N   MET A 383     -14.451  10.480   1.910  1.00 15.00       A    N
ATOM   2964  CA  MET A 383     -15.525  11.470   1.921  1.00 15.00       A    C
ATOM   2965  CB  MET A 383     -15.053  12.770   2.578  1.00 15.00       A    C
ATOM   2966  CG  MET A 383     -14.943  12.699   4.092  1.00 15.00       A    C
ATOM   2967  SD  MET A 383     -14.212  14.179   4.820  1.00 15.00       A    S
ATOM   2968  CE  MET A 383     -15.316  15.451   4.208  1.00 15.00       A    C
ATOM   2969  C   MET A 383     -16.043  11.760   0.516  1.00 15.00       A    C
ATOM   2970  O   MET A 383     -17.193  12.165   0.341  1.00 15.00       A    O
ATOM   2971  N   ALA A 384     -15.194  11.548  -0.479  1.00 15.00       A    N
ATOM   2972  CA  ALA A 384     -15.563  11.800  -1.863  1.00 15.00       A    C
ATOM   2973  CB  ALA A 384     -14.331  11.850  -2.748  1.00 15.00       A    C
ATOM   2974  C   ALA A 384     -16.566  10.774  -2.383  1.00 15.00       A    C
ATOM   2975  O   ALA A 384     -17.510  11.127  -3.093  1.00 15.00       A    O
```

ATOM	2976	N	TYR	A	385	-16.378	9.507	-2.024	1.00	15.00	A	N
ATOM	2977	CA	TYR	A	385	-17.291	8.463	-2.478	1.00	15.00	A	C
ATOM	2978	CB	TYR	A	385	-16.590	7.142	-2.850	1.00	15.00	A	C
ATOM	2979	CG	TYR	A	385	-15.661	6.527	-1.816	1.00	15.00	A	C
ATOM	2980	CD1	TYR	A	385	-16.149	5.984	-0.633	1.00	15.00	A	C
ATOM	2981	CD2	TYR	A	385	-14.293	6.449	-2.050	1.00	15.00	A	C
ATOM	2982	CE1	TYR	A	385	-15.303	5.383	0.283	1.00	15.00	A	C
ATOM	2983	CE2	TYR	A	385	-13.439	5.858	-1.136	1.00	15.00	A	C
ATOM	2984	CZ	TYR	A	385	-13.949	5.325	0.028	1.00	15.00	A	C
ATOM	2985	OH	TYR	A	385	-13.107	4.715	0.930	1.00	15.00	A	O
ATOM	2986	C	TYR	A	385	-18.485	8.271	-1.541	1.00	15.00	A	C
ATOM	2987	O	TYR	A	385	-18.963	7.155	-1.335	1.00	15.00	A	O
ATOM	2988	N	ALA	A	386	-18.979	9.383	-1.001	1.00	15.00	A	N
ATOM	2989	CA	ALA	A	386	-20.121	9.365	-0.094	1.00	15.00	A	C
ATOM	2990	CB	ALA	A	386	-20.316	10.731	0.545	1.00	15.00	A	C
ATOM	2991	C	ALA	A	386	-21.394	8.923	-0.813	1.00	15.00	A	C
ATOM	2992	O	ALA	A	386	-22.175	8.130	-0.287	1.00	15.00	A	O
ATOM	2993	N	ALA	A	387	-21.594	9.425	-2.025	1.00	15.00	A	N
ATOM	2994	CA	ALA	A	387	-22.771	9.072	-2.811	1.00	15.00	A	C
ATOM	2995	CB	ALA	A	387	-23.202	10.238	-3.691	1.00	15.00	A	C
ATOM	2996	C	ALA	A	387	-22.501	7.817	-3.643	1.00	15.00	A	C
ATOM	2997	O	ALA	A	387	-22.764	7.780	-4.845	1.00	15.00	A	O
ATOM	2998	N	GLN	A	388	-21.958	6.798	-2.987	1.00	15.00	A	N
ATOM	2999	CA	GLN	A	388	-21.640	5.531	-3.632	1.00	15.00	A	C
ATOM	3000	CB	GLN	A	388	-20.124	5.403	-3.834	1.00	15.00	A	C
ATOM	3001	CG	GLN	A	388	-19.550	6.237	-4.970	1.00	15.00	A	C
ATOM	3002	CD	GLN	A	388	-19.871	5.672	-6.340	1.00	15.00	A	C
ATOM	3003	OE1	GLN	A	388	-20.149	4.483	-6.488	1.00	15.00	A	O
ATOM	3004	NE2	GLN	A	388	-19.809	6.517	-7.357	1.00	15.00	A	N
ATOM	3005	C	GLN	A	388	-22.125	4.370	-2.766	1.00	15.00	A	C
ATOM	3006	O	GLN	A	388	-22.104	4.459	-1.534	1.00	15.00	A	O
ATOM	3007	N	PRO	A	389	-22.586	3.273	-3.394	1.00	15.00	A	N
ATOM	3008	CA	PRO	A	389	-23.059	2.085	-2.672	1.00	15.00	A	C
ATOM	3009	CB	PRO	A	389	-23.477	1.114	-3.781	1.00	15.00	A	C
ATOM	3010	CG	PRO	A	389	-22.826	1.628	-5.019	1.00	15.00	A	C
ATOM	3011	CD	PRO	A	389	-22.701	3.114	-4.850	1.00	15.00	A	C
ATOM	3012	C	PRO	A	389	-21.955	1.476	-1.804	1.00	15.00	A	C
ATOM	3013	O	PRO	A	389	-20.770	1.629	-2.096	1.00	15.00	A	O
ATOM	3014	N	PHE	A	390	-22.359	0.766	-0.758	1.00	15.00	A	N
ATOM	3015	CA	PHE	A	390	-21.429	0.159	0.194	1.00	15.00	A	C
ATOM	3016	CB	PHE	A	390	-22.190	-0.629	1.264	1.00	15.00	A	C
ATOM	3017	CG	PHE	A	390	-21.317	-1.150	2.370	1.00	15.00	A	C
ATOM	3018	CD1	PHE	A	390	-20.933	-2.481	2.401	1.00	15.00	A	C
ATOM	3019	CD2	PHE	A	390	-20.879	-0.307	3.377	1.00	15.00	A	C
ATOM	3020	CE1	PHE	A	390	-20.127	-2.961	3.414	1.00	15.00	A	C
ATOM	3021	CE2	PHE	A	390	-20.072	-0.780	4.394	1.00	15.00	A	C
ATOM	3022	CZ	PHE	A	390	-19.696	-2.109	4.413	1.00	15.00	A	C
ATOM	3023	C	PHE	A	390	-20.359	-0.719	-0.457	1.00	15.00	A	C
ATOM	3024	O	PHE	A	390	-19.169	-0.544	-0.198	1.00	15.00	A	O
ATOM	3025	N	LEU	A	391	-20.774	-1.646	-1.312	1.00	15.00	A	N
ATOM	3026	CA	LEU	A	391	-19.832	-2.560	-1.960	1.00	15.00	A	C
ATOM	3027	CB	LEU	A	391	-20.555	-3.765	-2.562	1.00	15.00	A	C
ATOM	3028	CG	LEU	A	391	-21.047	-4.816	-1.564	1.00	15.00	A	C
ATOM	3029	CD1	LEU	A	391	-21.896	-5.865	-2.262	1.00	15.00	A	C
ATOM	3030	CD2	LEU	A	391	-19.873	-5.467	-0.847	1.00	15.00	A	C
ATOM	3031	C	LEU	A	391	-18.940	-1.879	-2.996	1.00	15.00	A	C
ATOM	3032	O	LEU	A	391	-18.050	-2.509	-3.569	1.00	15.00	A	O
ATOM	3033	N	LEU	A	392	-19.168	-0.597	-3.234	1.00	15.00	A	N
ATOM	3034	CA	LEU	A	392	-18.368	0.145	-4.194	1.00	15.00	A	C
ATOM	3035	CB	LEU	A	392	-19.227	0.661	-5.352	1.00	15.00	A	C
ATOM	3036	CG	LEU	A	392	-19.830	-0.416	-6.264	1.00	15.00	A	C
ATOM	3037	CD1	LEU	A	392	-20.680	0.211	-7.356	1.00	15.00	A	C
ATOM	3038	CD2	LEU	A	392	-18.739	-1.288	-6.868	1.00	15.00	A	C
ATOM	3039	C	LEU	A	392	-17.608	1.276	-3.509	1.00	15.00	A	C
ATOM	3040	O	LEU	A	392	-17.000	2.123	-4.165	1.00	15.00	A	O
ATOM	3041	N	ARG	A	393	-17.641	1.273	-2.179	1.00	15.00	A	N
ATOM	3042	CA	ARG	A	393	-16.948	2.283	-1.391	1.00	15.00	A	C
ATOM	3043	CB	ARG	A	393	-17.691	2.576	-0.087	1.00	15.00	A	C
ATOM	3044	CG	ARG	A	393	-18.968	3.377	-0.259	1.00	15.00	A	C
ATOM	3045	CD	ARG	A	393	-19.590	3.699	1.087	1.00	15.00	A	C
ATOM	3046	NE	ARG	A	393	-21.050	3.741	1.021	1.00	15.00	A	N

ATOM	3047	CZ	ARG	A	393	-21.842	3.650	2.090	1.00	15.00	A	C
ATOM	3048	NH1	ARG	A	393	-21.316	3.516	3.303	1.00	15.00	A	N
ATOM	3049	NH2	ARG	A	393	-23.160	3.695	1.953	1.00	15.00	A	N
ATOM	3050	C	ARG	A	393	-15.531	1.824	-1.085	1.00	15.00	A	C
ATOM	3051	O	ARG	A	393	-15.297	1.092	-0.123	1.00	15.00	A	O
ATOM	3052	N	ASN	A	394	-14.604	2.238	-1.931	1.00	15.00	A	N
ATOM	3053	CA	ASN	A	394	-13.198	1.889	-1.790	1.00	15.00	A	C
ATOM	3054	CB	ASN	A	394	-13.019	0.365	-1.869	1.00	15.00	A	C
ATOM	3055	CG	ASN	A	394	-11.814	-0.136	-1.095	1.00	15.00	A	C
ATOM	3056	OD1	ASN	A	394	-10.712	-0.234	-1.632	1.00	15.00	A	O
ATOM	3057	ND2	ASN	A	394	-12.017	-0.461	0.173	1.00	15.00	A	N
ATOM	3058	C	ASN	A	394	-12.437	2.550	-2.927	1.00	15.00	A	C
ATOM	3059	O	ASN	A	394	-13.001	3.378	-3.642	1.00	15.00	A	O
ATOM	3060	N	GLY	A	395	-11.175	2.194	-3.100	1.00	15.00	A	N
ATOM	3061	CA	GLY	A	395	-10.396	2.761	-4.177	1.00	15.00	A	C
ATOM	3062	C	GLY	A	395	-10.546	1.923	-5.426	1.00	15.00	A	C
ATOM	3063	O	GLY	A	395	-11.031	0.793	-5.350	1.00	15.00	A	O
ATOM	3064	N	ALA	A	396	-10.140	2.467	-6.569	1.00	15.00	A	N
ATOM	3065	CA	ALA	A	396	-10.239	1.745	-7.836	1.00	15.00	A	C
ATOM	3066	CB	ALA	A	396	-9.681	2.578	-8.977	1.00	15.00	A	C
ATOM	3067	C	ALA	A	396	-9.549	0.384	-7.760	1.00	15.00	A	C
ATOM	3068	O	ALA	A	396	-10.069	-0.611	-8.261	1.00	15.00	A	O
ATOM	3069	N	ASN	A	397	-8.383	0.351	-7.123	1.00	15.00	A	N
ATOM	3070	CA	ASN	A	397	-7.630	-0.887	-6.960	1.00	15.00	A	C
ATOM	3071	CB	ASN	A	397	-6.836	-1.272	-8.223	1.00	15.00	A	C
ATOM	3072	CG	ASN	A	397	-5.739	-0.297	-8.611	1.00	15.00	A	C
ATOM	3073	OD1	ASN	A	397	-5.128	0.357	-7.768	1.00	15.00	A	O
ATOM	3074	ND2	ASN	A	397	-5.474	-0.201	-9.906	1.00	15.00	A	N
ATOM	3075	C	ASN	A	397	-6.745	-0.834	-5.717	1.00	15.00	A	C
ATOM	3076	O	ASN	A	397	-6.633	0.216	-5.069	1.00	15.00	A	O
ATOM	3077	N	GLU	A	398	-6.115	-1.960	-5.400	1.00	15.00	A	N
ATOM	3078	CA	GLU	A	398	-5.252	-2.086	-4.225	1.00	15.00	A	C
ATOM	3079	CB	GLU	A	398	-4.767	-3.528	-4.045	1.00	15.00	A	C
ATOM	3080	CG	GLU	A	398	-4.215	-4.177	-5.303	1.00	15.00	A	C
ATOM	3081	CD	GLU	A	398	-3.166	-5.230	-5.004	1.00	15.00	A	C
ATOM	3082	OE1	GLU	A	398	-3.336	-5.991	-4.024	1.00	15.00	A	O
ATOM	3083	OE2	GLU	A	398	-2.159	-5.286	-5.744	1.00	15.00	A	O
ATOM	3084	C	GLU	A	398	-4.075	-1.102	-4.180	1.00	15.00	A	C
ATOM	3085	O	GLU	A	398	-3.417	-0.974	-3.143	1.00	15.00	A	O
ATOM	3086	N	GLY	A	399	-3.801	-0.414	-5.281	1.00	15.00	A	N
ATOM	3087	CA	GLY	A	399	-2.699	0.532	-5.301	1.00	15.00	A	C
ATOM	3088	C	GLY	A	399	-3.136	1.954	-5.613	1.00	15.00	A	C
ATOM	3089	O	GLY	A	399	-2.301	2.841	-5.780	1.00	15.00	A	O
ATOM	3090	N	PHE	A	400	-4.442	2.183	-5.664	1.00	15.00	A	N
ATOM	3091	CA	PHE	A	400	-4.972	3.507	-5.978	1.00	15.00	A	C
ATOM	3092	CB	PHE	A	400	-6.377	3.396	-6.578	1.00	15.00	A	C
ATOM	3093	CG	PHE	A	400	-6.489	3.904	-7.988	1.00	15.00	A	C
ATOM	3094	CD1	PHE	A	400	-5.946	3.189	-9.044	1.00	15.00	A	C
ATOM	3095	CD2	PHE	A	400	-7.149	5.091	-8.260	1.00	15.00	A	C
ATOM	3096	CE1	PHE	A	400	-6.057	3.648	-10.343	1.00	15.00	A	C
ATOM	3097	CE2	PHE	A	400	-7.263	5.557	-9.557	1.00	15.00	A	C
ATOM	3098	CZ	PHE	A	400	-6.717	4.834	-10.600	1.00	15.00	A	C
ATOM	3099	C	PHE	A	400	-4.995	4.430	-4.761	1.00	15.00	A	C
ATOM	3100	O	PHE	A	400	-4.544	5.572	-4.834	1.00	15.00	A	O
ATOM	3101	N	HIS	A	401	-5.513	3.923	-3.646	1.00	15.00	A	N
ATOM	3102	CA	HIS	A	401	-5.622	4.704	-2.411	1.00	15.00	A	C
ATOM	3103	CB	HIS	A	401	-6.180	3.846	-1.273	1.00	15.00	A	C
ATOM	3104	CG	HIS	A	401	-7.558	4.239	-0.841	1.00	15.00	A	C
ATOM	3105	ND1	HIS	A	401	-7.831	5.011	0.269	1.00	15.00	A	N
ATOM	3106	CD2	HIS	A	401	-8.758	3.948	-1.396	1.00	15.00	A	C
ATOM	3107	CE1	HIS	A	401	-9.163	5.157	0.350	1.00	15.00	A	C
ATOM	3108	NE2	HIS	A	401	-9.769	4.529	-0.642	1.00	15.00	A	N
ATOM	3109	C	HIS	A	401	-4.297	5.329	-1.985	1.00	15.00	A	C
ATOM	3110	O	HIS	A	401	-4.203	6.542	-1.811	1.00	15.00	A	O
ATOM	3111	N	GLU	A	402	-3.278	4.495	-1.835	1.00	15.00	A	N
ATOM	3112	CA	GLU	A	402	-1.959	4.953	-1.408	1.00	15.00	A	C
ATOM	3113	CB	GLU	A	402	-1.016	3.764	-1.236	1.00	15.00	A	C
ATOM	3114	CG	GLU	A	402	-1.135	3.077	0.112	1.00	15.00	A	C
ATOM	3115	CD	GLU	A	402	-0.546	3.916	1.227	1.00	15.00	A	C
ATOM	3116	OE1	GLU	A	402	-1.280	4.750	1.802	1.00	15.00	A	O
ATOM	3117	OE2	GLU	A	402	0.656	3.753	1.513	1.00	15.00	A	O

ATOM	3118	C	GLU	A	402	-1.358	5.995	-2.353	1.00	15.00	A	C
ATOM	3119	O	GLU	A	402	-0.583	6.853	-1.932	1.00	15.00	A	O
ATOM	3120	N	ALA	A	403	-1.736	5.934	-3.625	1.00	15.00	A	N
ATOM	3121	CA	ALA	A	403	-1.228	6.876	-4.616	1.00	15.00	A	C
ATOM	3122	CB	ALA	A	403	-1.587	6.416	-6.019	1.00	15.00	A	C
ATOM	3123	C	ALA	A	403	-1.764	8.281	-4.354	1.00	15.00	A	C
ATOM	3124	O	ALA	A	403	-1.077	9.276	-4.588	1.00	15.00	A	O
ATOM	3125	N	VAL	A	404	-2.990	8.348	-3.850	1.00	15.00	A	N
ATOM	3126	CA	VAL	A	404	-3.631	9.622	-3.543	1.00	15.00	A	C
ATOM	3127	CB	VAL	A	404	-5.154	9.444	-3.333	1.00	15.00	A	C
ATOM	3128	CG1	VAL	A	404	-5.847	10.785	-3.144	1.00	15.00	A	C
ATOM	3129	CG2	VAL	A	404	-5.768	8.688	-4.502	1.00	15.00	A	C
ATOM	3130	C	VAL	A	404	-3.010	10.242	-2.291	1.00	15.00	A	C
ATOM	3131	O	VAL	A	404	-3.062	11.454	-2.088	1.00	15.00	A	O
ATOM	3132	N	GLY	A	405	-2.398	9.404	-1.466	1.00	15.00	A	N
ATOM	3133	CA	GLY	A	405	-1.782	9.885	-0.247	1.00	15.00	A	C
ATOM	3134	C	GLY	A	405	-0.391	10.446	-0.472	1.00	15.00	A	C
ATOM	3135	O	GLY	A	405	-0.071	11.538	0.006	1.00	15.00	A	O
ATOM	3136	N	GLU	A	406	0.427	9.715	-1.224	1.00	15.00	A	N
ATOM	3137	CA	GLU	A	406	1.803	10.126	-1.495	1.00	15.00	A	C
ATOM	3138	CB	GLU	A	406	2.577	9.032	-2.232	1.00	15.00	A	C
ATOM	3139	CG	GLU	A	406	2.966	7.851	-1.350	1.00	15.00	A	C
ATOM	3140	CD	GLU	A	406	4.471	7.655	-1.247	1.00	15.00	A	C
ATOM	3141	OE1	GLU	A	406	5.220	8.413	-1.899	1.00	15.00	A	O
ATOM	3142	OE2	GLU	A	406	4.906	6.723	-0.532	1.00	15.00	A	O
ATOM	3143	C	GLU	A	406	1.902	11.469	-2.223	1.00	15.00	A	C
ATOM	3144	O	GLU	A	406	2.810	12.255	-1.954	1.00	15.00	A	O
ATOM	3145	N	ILE	A	407	0.969	11.742	-3.135	1.00	15.00	A	N
ATOM	3146	CA	ILE	A	407	0.986	13.007	-3.875	1.00	15.00	A	C
ATOM	3147	CB	ILE	A	407	-0.066	13.064	-5.003	1.00	15.00	A	C
ATOM	3148	CG1	ILE	A	407	-1.472	12.790	-4.462	1.00	15.00	A	C
ATOM	3149	CG2	ILE	A	407	0.290	12.086	-6.112	1.00	15.00	A	C
ATOM	3150	CD1	ILE	A	407	-2.573	12.984	-5.483	1.00	15.00	A	C
ATOM	3151	C	ILE	A	407	0.822	14.209	-2.943	1.00	15.00	A	C
ATOM	3152	O	ILE	A	407	1.421	15.263	-3.164	1.00	15.00	A	O
ATOM	3153	N	MET	A	408	0.028	14.033	-1.888	1.00	15.00	A	N
ATOM	3154	CA	MET	A	408	-0.204	15.100	-0.919	1.00	15.00	A	C
ATOM	3155	CB	MET	A	408	-1.275	14.690	0.096	1.00	15.00	A	C
ATOM	3156	CG	MET	A	408	-2.588	14.236	-0.519	1.00	15.00	A	C
ATOM	3157	SD	MET	A	408	-3.366	15.510	-1.528	1.00	15.00	A	S
ATOM	3158	CE	MET	A	408	-4.751	14.596	-2.201	1.00	15.00	A	C
ATOM	3159	C	MET	A	408	1.088	15.421	-0.187	1.00	15.00	A	C
ATOM	3160	O	MET	A	408	1.460	16.585	-0.031	1.00	15.00	A	O
ATOM	3161	N	SER	A	409	1.777	14.371	0.238	1.00	15.00	A	N
ATOM	3162	CA	SER	A	409	3.033	14.511	0.951	1.00	15.00	A	C
ATOM	3163	CB	SER	A	409	3.486	13.143	1.469	1.00	15.00	A	C
ATOM	3164	OG	SER	A	409	2.418	12.486	2.137	1.00	15.00	A	O
ATOM	3165	C	SER	A	409	4.106	15.138	0.060	1.00	15.00	A	C
ATOM	3166	O	SER	A	409	4.998	15.831	0.545	1.00	15.00	A	O
ATOM	3167	N	LEU	A	410	3.999	14.902	-1.246	1.00	15.00	A	N
ATOM	3168	CA	LEU	A	410	4.956	15.443	-2.207	1.00	15.00	A	C
ATOM	3169	CB	LEU	A	410	4.706	14.875	-3.609	1.00	15.00	A	C
ATOM	3170	CG	LEU	A	410	5.013	13.388	-3.815	1.00	15.00	A	C
ATOM	3171	CD1	LEU	A	410	4.592	12.944	-5.208	1.00	15.00	A	C
ATOM	3172	CD2	LEU	A	410	6.489	13.105	-3.585	1.00	15.00	A	C
ATOM	3173	C	LEU	A	410	4.915	16.969	-2.249	1.00	15.00	A	C
ATOM	3174	O	LEU	A	410	5.909	17.631	-1.955	1.00	15.00	A	O
ATOM	3175	N	SER	A	411	3.760	17.523	-2.602	1.00	15.00	A	N
ATOM	3176	CA	SER	A	411	3.604	18.972	-2.700	1.00	15.00	A	C
ATOM	3177	CB	SER	A	411	2.244	19.328	-3.304	1.00	15.00	A	C
ATOM	3178	OG	SER	A	411	2.164	18.889	-4.650	1.00	15.00	A	O
ATOM	3179	C	SER	A	411	3.804	19.680	-1.361	1.00	15.00	A	C
ATOM	3180	O	SER	A	411	4.376	20.769	-1.311	1.00	15.00	A	O
ATOM	3181	N	ALA	A	412	3.366	19.048	-0.279	1.00	15.00	A	N
ATOM	3182	CA	ALA	A	412	3.486	19.635	1.054	1.00	15.00	A	C
ATOM	3183	CB	ALA	A	412	2.585	18.906	2.039	1.00	15.00	A	C
ATOM	3184	C	ALA	A	412	4.928	19.656	1.562	1.00	15.00	A	C
ATOM	3185	O	ALA	A	412	5.229	20.306	2.562	1.00	15.00	A	O
ATOM	3186	N	ALA	A	413	5.819	18.954	0.875	1.00	15.00	A	N
ATOM	3187	CA	ALA	A	413	7.218	18.901	1.284	1.00	15.00	A	C
ATOM	3188	CB	ALA	A	413	7.731	17.470	1.243	1.00	15.00	A	C

ATOM	3189	C	ALA	A	413	8.089	19.802	0.418	1.00	15.00	A	C
ATOM	3190	O	ALA	A	413	9.312	19.824	0.560	1.00	15.00	A	O
ATOM	3191	N	THR	A	414	7.461	20.555	-0.472	1.00	15.00	A	N
ATOM	3192	CA	THR	A	414	8.200	21.440	-1.355	1.00	15.00	A	C
ATOM	3193	CB	THR	A	414	7.425	21.749	-2.652	1.00	15.00	A	C
ATOM	3194	OG1	THR	A	414	6.200	22.434	-2.350	1.00	15.00	A	O
ATOM	3195	CG2	THR	A	414	7.121	20.466	-3.409	1.00	15.00	A	C
ATOM	3196	C	THR	A	414	8.560	22.743	-0.650	1.00	15.00	A	C
ATOM	3197	O	THR	A	414	7.785	23.254	0.164	1.00	15.00	A	O
ATOM	3198	N	PRO	A	415	9.751	23.293	-0.946	1.00	15.00	A	N
ATOM	3199	CA	PRO	A	415	10.212	24.551	-0.350	1.00	15.00	A	C
ATOM	3200	CB	PRO	A	415	11.522	24.838	-1.089	1.00	15.00	A	C
ATOM	3201	CG	PRO	A	415	11.989	23.504	-1.555	1.00	15.00	A	C
ATOM	3202	CD	PRO	A	415	10.747	22.721	-1.870	1.00	15.00	A	C
ATOM	3203	C	PRO	A	415	9.213	25.676	-0.595	1.00	15.00	A	C
ATOM	3204	O	PRO	A	415	9.067	26.577	0.228	1.00	15.00	A	O
ATOM	3205	N	LYS	A	416	8.515	25.598	-1.725	1.00	15.00	A	N
ATOM	3206	CA	LYS	A	416	7.518	26.597	-2.088	1.00	15.00	A	C
ATOM	3207	CB	LYS	A	416	6.958	26.311	-3.487	1.00	15.00	A	C
ATOM	3208	CG	LYS	A	416	6.088	27.428	-4.050	1.00	15.00	A	C
ATOM	3209	CD	LYS	A	416	4.841	26.885	-4.736	1.00	15.00	A	C
ATOM	3210	CE	LYS	A	416	5.172	26.174	-6.040	1.00	15.00	A	C
ATOM	3211	NZ	LYS	A	416	3.944	25.684	-6.727	1.00	15.00	A	N
ATOM	3212	C	LYS	A	416	6.383	26.611	-1.069	1.00	15.00	A	C
ATOM	3213	O	LYS	A	416	6.061	27.654	-0.498	1.00	15.00	A	O
ATOM	3214	N	HIS	A	417	5.798	25.441	-0.832	1.00	15.00	A	N
ATOM	3215	CA	HIS	A	417	4.700	25.306	0.117	1.00	15.00	A	C
ATOM	3216	CB	HIS	A	417	4.158	23.869	0.091	1.00	15.00	A	C
ATOM	3217	CG	HIS	A	417	2.926	23.651	0.921	1.00	15.00	A	C
ATOM	3218	ND1	HIS	A	417	1.640	23.708	0.426	1.00	15.00	A	N
ATOM	3219	CD2	HIS	A	417	2.802	23.342	2.237	1.00	15.00	A	C
ATOM	3220	CE1	HIS	A	417	0.798	23.457	1.437	1.00	15.00	A	C
ATOM	3221	NE2	HIS	A	417	1.454	23.224	2.557	1.00	15.00	A	N
ATOM	3222	C	HIS	A	417	5.163	25.680	1.522	1.00	15.00	A	C
ATOM	3223	O	HIS	A	417	4.482	26.414	2.238	1.00	15.00	A	O
ATOM	3224	N	LEU	A	418	6.344	25.197	1.894	1.00	15.00	A	N
ATOM	3225	CA	LEU	A	418	6.910	25.467	3.212	1.00	15.00	A	C
ATOM	3226	CB	LEU	A	418	8.220	24.702	3.399	1.00	15.00	A	C
ATOM	3227	CG	LEU	A	418	8.106	23.176	3.397	1.00	15.00	A	C
ATOM	3228	CD1	LEU	A	418	9.482	22.537	3.321	1.00	15.00	A	C
ATOM	3229	CD2	LEU	A	418	7.355	22.691	4.627	1.00	15.00	A	C
ATOM	3230	C	LEU	A	418	7.122	26.962	3.447	1.00	15.00	A	C
ATOM	3231	O	LEU	A	418	6.931	27.458	4.560	1.00	15.00	A	O
ATOM	3232	N	LYS	A	419	7.513	27.676	2.402	1.00	15.00	A	N
ATOM	3233	CA	LYS	A	419	7.736	29.113	2.506	1.00	15.00	A	C
ATOM	3234	CB	LYS	A	419	8.583	29.617	1.334	1.00	15.00	A	C
ATOM	3235	CG	LYS	A	419	10.059	29.272	1.443	1.00	15.00	A	C
ATOM	3236	CD	LYS	A	419	10.793	29.541	0.139	1.00	15.00	A	C
ATOM	3237	CE	LYS	A	419	12.275	29.227	0.269	1.00	15.00	A	C
ATOM	3238	NZ	LYS	A	419	13.017	29.481	-0.994	1.00	15.00	A	N
ATOM	3239	C	LYS	A	419	6.409	29.859	2.559	1.00	15.00	A	C
ATOM	3240	O	LYS	A	419	6.288	30.885	3.225	1.00	15.00	A	O
ATOM	3241	N	SER	A	420	5.412	29.323	1.867	1.00	15.00	A	N
ATOM	3242	CA	SER	A	420	4.093	29.933	1.822	1.00	15.00	A	C
ATOM	3243	CB	SER	A	420	3.275	29.351	0.665	1.00	15.00	A	C
ATOM	3244	OG	SER	A	420	2.064	30.063	0.482	1.00	15.00	A	O
ATOM	3245	C	SER	A	420	3.342	29.767	3.143	1.00	15.00	A	C
ATOM	3246	O	SER	A	420	2.551	30.630	3.524	1.00	15.00	A	O
ATOM	3247	N	ILE	A	421	3.582	28.659	3.839	1.00	15.00	A	N
ATOM	3248	CA	ILE	A	421	2.911	28.406	5.112	1.00	15.00	A	C
ATOM	3249	CB	ILE	A	421	2.687	26.903	5.382	1.00	15.00	A	C
ATOM	3250	CG1	ILE	A	421	4.014	26.137	5.362	1.00	15.00	A	C
ATOM	3251	CG2	ILE	A	421	1.707	26.325	4.369	1.00	15.00	A	C
ATOM	3252	CD1	ILE	A	421	3.867	24.642	5.540	1.00	15.00	A	C
ATOM	3253	C	ILE	A	421	3.642	29.060	6.282	1.00	15.00	A	C
ATOM	3254	O	ILE	A	421	3.178	29.014	7.421	1.00	15.00	A	O
ATOM	3255	N	GLY	A	422	4.788	29.665	5.991	1.00	15.00	A	N
ATOM	3256	CA	GLY	A	422	5.558	30.341	7.018	1.00	15.00	A	C
ATOM	3257	C	GLY	A	422	6.397	29.404	7.866	1.00	15.00	A	C
ATOM	3258	O	GLY	A	422	6.767	29.744	8.988	1.00	15.00	A	O
ATOM	3259	N	LEU	A	423	6.702	28.228	7.340	1.00	15.00	A	N

```
ATOM   3260  CA  LEU A 423       7.512  27.265   8.074  1.00 15.00      A    C
ATOM   3261  CB  LEU A 423       6.980  25.841   7.898  1.00 15.00      A    C
ATOM   3262  CG  LEU A 423       5.907  25.400   8.896  1.00 15.00      A    C
ATOM   3263  CD1 LEU A 423       5.532  23.943   8.673  1.00 15.00      A    C
ATOM   3264  CD2 LEU A 423       6.384  25.619  10.324  1.00 15.00      A    C
ATOM   3265  C   LEU A 423       8.974  27.348   7.657  1.00 15.00      A    C
ATOM   3266  O   LEU A 423       9.875  27.059   8.442  1.00 15.00      A    O
ATOM   3267  N   LEU A 424       9.201  27.751   6.418  1.00 15.00      A    N
ATOM   3268  CA  LEU A 424      10.547  27.878   5.890  1.00 15.00      A    C
ATOM   3269  CB  LEU A 424      10.711  26.974   4.665  1.00 15.00      A    C
ATOM   3270  CG  LEU A 424      12.122  26.842   4.091  1.00 15.00      A    C
ATOM   3271  CD1 LEU A 424      13.052  26.183   5.098  1.00 15.00      A    C
ATOM   3272  CD2 LEU A 424      12.092  26.053   2.791  1.00 15.00      A    C
ATOM   3273  C   LEU A 424      10.819  29.326   5.511  1.00 15.00      A    C
ATOM   3274  O   LEU A 424       9.995  29.963   4.855  1.00 15.00      A    O
ATOM   3275  N   SER A 425      11.965  29.845   5.935  1.00 15.00      A    N
ATOM   3276  CA  SER A 425      12.336  31.217   5.628  1.00 15.00      A    C
ATOM   3277  CB  SER A 425      13.649  31.593   6.323  1.00 15.00      A    C
ATOM   3278  OG  SER A 425      13.551  32.868   6.933  1.00 15.00      A    O
ATOM   3279  C   SER A 425      12.451  31.408   4.118  1.00 15.00      A    C
ATOM   3280  O   SER A 425      13.012  30.563   3.416  1.00 15.00      A    O
ATOM   3281  N   PRO A 426      11.900  32.513   3.598  1.00 15.00      A    N
ATOM   3282  CA  PRO A 426      11.930  32.823   2.162  1.00 15.00      A    C
ATOM   3283  CB  PRO A 426      11.220  34.181   2.075  1.00 15.00      A    C
ATOM   3284  CG  PRO A 426      10.401  34.261   3.317  1.00 15.00      A    C
ATOM   3285  CD  PRO A 426      11.195  33.550   4.371  1.00 15.00      A    C
ATOM   3286  C   PRO A 426      13.357  32.945   1.638  1.00 15.00      A    C
ATOM   3287  O   PRO A 426      13.632  32.663   0.471  1.00 15.00      A    O
ATOM   3288  N   ASP A 427      14.254  33.335   2.528  1.00 15.00      A    N
ATOM   3289  CA  ASP A 427      15.664  33.526   2.217  1.00 15.00      A    C
ATOM   3290  CB  ASP A 427      16.337  34.289   3.364  1.00 15.00      A    C
ATOM   3291  CG  ASP A 427      15.331  35.039   4.225  1.00 15.00      A    C
ATOM   3292  OD1 ASP A 427      15.364  36.287   4.232  1.00 15.00      A    O
ATOM   3293  OD2 ASP A 427      14.487  34.369   4.881  1.00 15.00      A    O
ATOM   3294  C   ASP A 427      16.376  32.198   1.971  1.00 15.00      A    C
ATOM   3295  O   ASP A 427      17.532  32.178   1.547  1.00 15.00      A    O
ATOM   3296  N   PHE A 428      15.693  31.090   2.256  1.00 15.00      A    N
ATOM   3297  CA  PHE A 428      16.263  29.767   2.035  1.00 15.00      A    C
ATOM   3298  CB  PHE A 428      15.311  28.671   2.524  1.00 15.00      A    C
ATOM   3299  CG  PHE A 428      15.891  27.285   2.459  1.00 15.00      A    C
ATOM   3300  CD1 PHE A 428      15.559  26.425   1.424  1.00 15.00      A    C
ATOM   3301  CD2 PHE A 428      16.772  26.845   3.432  1.00 15.00      A    C
ATOM   3302  CE1 PHE A 428      16.092  25.151   1.363  1.00 15.00      A    C
ATOM   3303  CE2 PHE A 428      17.309  25.573   3.378  1.00 15.00      A    C
ATOM   3304  CZ  PHE A 428      16.970  24.725   2.342  1.00 15.00      A    C
ATOM   3305  C   PHE A 428      16.560  29.579   0.551  1.00 15.00      A    C
ATOM   3306  O   PHE A 428      15.645  29.520  -0.274  1.00 15.00      A    O
ATOM   3307  N   GLN A 429      17.840  29.502   0.224  1.00 15.00      A    N
ATOM   3308  CA  GLN A 429      18.280  29.344  -1.151  1.00 15.00      A    C
ATOM   3309  CB  GLN A 429      19.756  29.743  -1.276  1.00 15.00      A    C
ATOM   3310  CG  GLN A 429      20.330  29.650  -2.684  1.00 15.00      A    C
ATOM   3311  CD  GLN A 429      19.737  30.668  -3.641  1.00 15.00      A    C
ATOM   3312  OE1 GLN A 429      18.571  31.044  -3.536  1.00 15.00      A    O
ATOM   3313  NE2 GLN A 429      20.542  31.121  -4.588  1.00 15.00      A    N
ATOM   3314  C   GLN A 429      18.065  27.921  -1.657  1.00 15.00      A    C
ATOM   3315  O   GLN A 429      18.319  26.952  -0.943  1.00 15.00      A    O
ATOM   3316  N   GLU A 430      17.584  27.815  -2.889  1.00 15.00      A    N
ATOM   3317  CA  GLU A 430      17.342  26.529  -3.527  1.00 15.00      A    C
ATOM   3318  CB  GLU A 430      15.889  26.449  -4.021  1.00 15.00      A    C
ATOM   3319  CG  GLU A 430      14.852  26.530  -2.907  1.00 15.00      A    C
ATOM   3320  CD  GLU A 430      13.440  26.804  -3.400  1.00 15.00      A    C
ATOM   3321  OE1 GLU A 430      12.945  26.050  -4.259  1.00 15.00      A    O
ATOM   3322  OE2 GLU A 430      12.812  27.761  -2.905  1.00 15.00      A    O
ATOM   3323  C   GLU A 430      18.306  26.377  -4.701  1.00 15.00      A    C
ATOM   3324  O   GLU A 430      18.150  27.038  -5.728  1.00 15.00      A    O
ATOM   3325  N   ASP A 431      19.319  25.535  -4.540  1.00 15.00      A    N
ATOM   3326  CA  ASP A 431      20.304  25.334  -5.594  1.00 15.00      A    C
ATOM   3327  CB  ASP A 431      21.698  25.824  -5.170  1.00 15.00      A    C
ATOM   3328  CG  ASP A 431      22.259  25.107  -3.953  1.00 15.00      A    C
ATOM   3329  OD1 ASP A 431      22.407  23.869  -3.995  1.00 15.00      A    O
ATOM   3330  OD2 ASP A 431      22.581  25.786  -2.956  1.00 15.00      A    O
```

ATOM	3331	C	ASP	A	431	20.359	23.888	-6.070	1.00	15.00	A	C
ATOM	3332	O	ASP	A	431	19.721	23.002	-5.493	1.00	15.00	A	O
ATOM	3333	N	ASN	A	432	21.136	23.672	-7.130	1.00	15.00	A	N
ATOM	3334	CA	ASN	A	432	21.316	22.352	-7.735	1.00	15.00	A	C
ATOM	3335	CB	ASN	A	432	22.428	22.403	-8.791	1.00	15.00	A	C
ATOM	3336	CG	ASN	A	432	21.948	22.811	-10.172	1.00	15.00	A	C
ATOM	3337	OD1	ASN	A	432	20.967	23.534	-10.318	1.00	15.00	A	O
ATOM	3338	ND2	ASN	A	432	22.654	22.357	-11.199	1.00	15.00	A	N
ATOM	3339	C	ASN	A	432	21.676	21.291	-6.701	1.00	15.00	A	C
ATOM	3340	O	ASN	A	432	21.082	20.215	-6.676	1.00	15.00	A	O
ATOM	3341	N	GLU	A	433	22.640	21.608	-5.846	1.00	15.00	A	N
ATOM	3342	CA	GLU	A	433	23.103	20.676	-4.826	1.00	15.00	A	C
ATOM	3343	CB	GLU	A	433	24.242	21.288	-4.012	1.00	15.00	A	C
ATOM	3344	CG	GLU	A	433	25.533	21.498	-4.786	1.00	15.00	A	C
ATOM	3345	CD	GLU	A	433	26.580	22.214	-3.958	1.00	15.00	A	C
ATOM	3346	OE1	GLU	A	433	27.247	21.548	-3.138	1.00	15.00	A	O
ATOM	3347	OE2	GLU	A	433	26.707	23.452	-4.100	1.00	15.00	A	O
ATOM	3348	C	GLU	A	433	21.972	20.247	-3.899	1.00	15.00	A	C
ATOM	3349	O	GLU	A	433	21.742	19.052	-3.695	1.00	15.00	A	O
ATOM	3350	N	THR	A	434	21.260	21.222	-3.350	1.00	15.00	A	N
ATOM	3351	CA	THR	A	434	20.160	20.942	-2.439	1.00	15.00	A	C
ATOM	3352	CB	THR	A	434	19.558	22.236	-1.864	1.00	15.00	A	C
ATOM	3353	OG1	THR	A	434	19.674	23.291	-2.829	1.00	15.00	A	O
ATOM	3354	CG2	THR	A	434	20.294	22.639	-0.595	1.00	15.00	A	C
ATOM	3355	C	THR	A	434	19.065	20.093	-3.088	1.00	15.00	A	C
ATOM	3356	O	THR	A	434	18.500	19.203	-2.449	1.00	15.00	A	O
ATOM	3357	N	GLU	A	435	18.783	20.352	-4.359	1.00	15.00	A	N
ATOM	3358	CA	GLU	A	435	17.755	19.603	-5.069	1.00	15.00	A	C
ATOM	3359	CB	GLU	A	435	17.293	20.351	-6.316	1.00	15.00	A	C
ATOM	3360	CG	GLU	A	435	16.715	21.720	-5.996	1.00	15.00	A	C
ATOM	3361	CD	GLU	A	435	15.815	22.261	-7.081	1.00	15.00	A	C
ATOM	3362	OE1	GLU	A	435	15.327	21.468	-7.908	1.00	15.00	A	O
ATOM	3363	OE2	GLU	A	435	15.571	23.483	-7.089	1.00	15.00	A	O
ATOM	3364	C	GLU	A	435	18.212	18.182	-5.388	1.00	15.00	A	C
ATOM	3365	O	GLU	A	435	17.426	17.235	-5.301	1.00	15.00	A	O
ATOM	3366	N	ILE	A	436	19.486	18.031	-5.740	1.00	15.00	A	N
ATOM	3367	CA	ILE	A	436	20.043	16.715	-6.041	1.00	15.00	A	C
ATOM	3368	CB	ILE	A	436	21.492	16.810	-6.578	1.00	15.00	A	C
ATOM	3369	CG1	ILE	A	436	21.505	17.416	-7.987	1.00	15.00	A	C
ATOM	3370	CG2	ILE	A	436	22.181	15.450	-6.571	1.00	15.00	A	C
ATOM	3371	CD1	ILE	A	436	20.678	16.649	-8.999	1.00	15.00	A	C
ATOM	3372	C	ILE	A	436	19.996	15.823	-4.799	1.00	15.00	A	C
ATOM	3373	O	ILE	A	436	19.644	14.644	-4.882	1.00	15.00	A	O
ATOM	3374	N	ASN	A	437	20.326	16.402	-3.644	1.00	15.00	A	N
ATOM	3375	CA	ASN	A	437	20.310	15.665	-2.380	1.00	15.00	A	C
ATOM	3376	CB	ASN	A	437	20.740	16.566	-1.217	1.00	15.00	A	C
ATOM	3377	CG	ASN	A	437	22.243	16.670	-1.042	1.00	15.00	A	C
ATOM	3378	OD1	ASN	A	437	23.016	15.936	-1.657	1.00	15.00	A	O
ATOM	3379	ND2	ASN	A	437	22.666	17.582	-0.182	1.00	15.00	A	N
ATOM	3380	C	ASN	A	437	18.912	15.131	-2.099	1.00	15.00	A	C
ATOM	3381	O	ASN	A	437	18.740	13.995	-1.651	1.00	15.00	A	O
ATOM	3382	N	PHE	A	438	17.918	15.965	-2.375	1.00	15.00	A	N
ATOM	3383	CA	PHE	A	438	16.524	15.605	-2.167	1.00	15.00	A	C
ATOM	3384	CB	PHE	A	438	15.627	16.817	-2.439	1.00	15.00	A	C
ATOM	3385	CG	PHE	A	438	14.154	16.522	-2.399	1.00	15.00	A	C
ATOM	3386	CD1	PHE	A	438	13.527	16.209	-1.204	1.00	15.00	A	C
ATOM	3387	CD2	PHE	A	438	13.396	16.562	-3.558	1.00	15.00	A	C
ATOM	3388	CE1	PHE	A	438	12.173	15.940	-1.166	1.00	15.00	A	C
ATOM	3389	CE2	PHE	A	438	12.041	16.294	-3.528	1.00	15.00	A	C
ATOM	3390	CZ	PHE	A	438	11.429	15.982	-2.330	1.00	15.00	A	C
ATOM	3391	C	PHE	A	438	16.130	14.435	-3.062	1.00	15.00	A	C
ATOM	3392	O	PHE	A	438	15.591	13.432	-2.590	1.00	15.00	A	O
ATOM	3393	N	LEU	A	439	16.421	14.561	-4.352	1.00	15.00	A	N
ATOM	3394	CA	LEU	A	439	16.096	13.520	-5.320	1.00	15.00	A	C
ATOM	3395	CB	LEU	A	439	16.447	13.970	-6.739	1.00	15.00	A	C
ATOM	3396	CG	LEU	A	439	15.529	15.028	-7.354	1.00	15.00	A	C
ATOM	3397	CD1	LEU	A	439	16.075	15.490	-8.695	1.00	15.00	A	C
ATOM	3398	CD2	LEU	A	439	14.116	14.485	-7.509	1.00	15.00	A	C
ATOM	3399	C	LEU	A	439	16.799	12.207	-4.989	1.00	15.00	A	C
ATOM	3400	O	LEU	A	439	16.240	11.128	-5.189	1.00	15.00	A	O
ATOM	3401	N	LEU	A	440	18.022	12.306	-4.477	1.00	15.00	A	N

ATOM	3402	CA	LEU	A	440	18.803	11.130	-4.109	1.00	15.00	A	C
ATOM	3403	CB	LEU	A	440	20.196	11.554	-3.623	1.00	15.00	A	C
ATOM	3404	CG	LEU	A	440	21.336	10.538	-3.784	1.00	15.00	A	C
ATOM	3405	CD1	LEU	A	440	21.360	9.547	-2.630	1.00	15.00	A	C
ATOM	3406	CD2	LEU	A	440	21.245	9.815	-5.120	1.00	15.00	A	C
ATOM	3407	C	LEU	A	440	18.077	10.328	-3.031	1.00	15.00	A	C
ATOM	3408	O	LEU	A	440	17.893	9.117	-3.162	1.00	15.00	A	O
ATOM	3409	N	LYS	A	441	17.642	11.020	-1.982	1.00	15.00	A	N
ATOM	3410	CA	LYS	A	441	16.929	10.382	-0.881	1.00	15.00	A	C
ATOM	3411	CB	LYS	A	441	16.657	11.403	0.230	1.00	15.00	A	C
ATOM	3412	CG	LYS	A	441	16.368	10.796	1.594	1.00	15.00	A	C
ATOM	3413	CD	LYS	A	441	16.284	11.872	2.667	1.00	15.00	A	C
ATOM	3414	CE	LYS	A	441	16.019	11.274	4.041	1.00	15.00	A	C
ATOM	3415	NZ	LYS	A	441	15.905	12.323	5.095	1.00	15.00	A	N
ATOM	3416	C	LYS	A	441	15.618	9.772	-1.376	1.00	15.00	A	C
ATOM	3417	O	LYS	A	441	15.200	8.702	-0.921	1.00	15.00	A	O
ATOM	3418	N	GLN	A	442	14.982	10.460	-2.317	1.00	15.00	A	N
ATOM	3419	CA	GLN	A	442	13.727	9.998	-2.896	1.00	15.00	A	C
ATOM	3420	CB	GLN	A	442	13.126	11.082	-3.796	1.00	15.00	A	C
ATOM	3421	CG	GLN	A	442	12.595	12.292	-3.047	1.00	15.00	A	C
ATOM	3422	CD	GLN	A	442	11.337	11.982	-2.263	1.00	15.00	A	C
ATOM	3423	OE1	GLN	A	442	11.398	11.534	-1.120	1.00	15.00	A	O
ATOM	3424	NE2	GLN	A	442	10.186	12.226	-2.870	1.00	15.00	A	N
ATOM	3425	C	GLN	A	442	13.934	8.716	-3.697	1.00	15.00	A	C
ATOM	3426	O	GLN	A	442	13.171	7.760	-3.562	1.00	15.00	A	O
ATOM	3427	N	ALA	A	443	14.978	8.697	-4.518	1.00	15.00	A	N
ATOM	3428	CA	ALA	A	443	15.288	7.539	-5.352	1.00	15.00	A	C
ATOM	3429	CB	ALA	A	443	16.449	7.845	-6.282	1.00	15.00	A	C
ATOM	3430	C	ALA	A	443	15.588	6.300	-4.515	1.00	15.00	A	C
ATOM	3431	O	ALA	A	443	15.179	5.194	-4.864	1.00	15.00	A	O
ATOM	3432	N	LEU	A	444	16.291	6.494	-3.407	1.00	15.00	A	N
ATOM	3433	CA	LEU	A	444	16.650	5.392	-2.518	1.00	15.00	A	C
ATOM	3434	CB	LEU	A	444	17.538	5.901	-1.381	1.00	15.00	A	C
ATOM	3435	CG	LEU	A	444	18.882	6.509	-1.782	1.00	15.00	A	C
ATOM	3436	CD1	LEU	A	444	19.580	7.093	-0.565	1.00	15.00	A	C
ATOM	3437	CD2	LEU	A	444	19.763	5.471	-2.461	1.00	15.00	A	C
ATOM	3438	C	LEU	A	444	15.411	4.724	-1.931	1.00	15.00	A	C
ATOM	3439	O	LEU	A	444	15.421	3.534	-1.617	1.00	15.00	A	O
ATOM	3440	N	THR	A	445	14.347	5.495	-1.793	1.00	15.00	A	N
ATOM	3441	CA	THR	A	445	13.106	4.991	-1.231	1.00	15.00	A	C
ATOM	3442	CB	THR	A	445	12.421	6.089	-0.393	1.00	15.00	A	C
ATOM	3443	OG1	THR	A	445	13.416	6.791	0.370	1.00	15.00	A	O
ATOM	3444	CG2	THR	A	445	11.393	5.488	0.552	1.00	15.00	A	C
ATOM	3445	C	THR	A	445	12.142	4.519	-2.320	1.00	15.00	A	C
ATOM	3446	O	THR	A	445	11.617	3.409	-2.264	1.00	15.00	A	O
ATOM	3447	N	ILE	A	446	11.938	5.360	-3.320	1.00	15.00	A	N
ATOM	3448	CA	ILE	A	446	11.010	5.062	-4.402	1.00	15.00	A	C
ATOM	3449	CB	ILE	A	446	10.546	6.361	-5.093	1.00	15.00	A	C
ATOM	3450	CG1	ILE	A	446	9.782	7.239	-4.096	1.00	15.00	A	C
ATOM	3451	CG2	ILE	A	446	9.686	6.055	-6.308	1.00	15.00	A	C
ATOM	3452	CD1	ILE	A	446	9.713	8.699	-4.486	1.00	15.00	A	C
ATOM	3453	C	ILE	A	446	11.580	4.088	-5.438	1.00	15.00	A	C
ATOM	3454	O	ILE	A	446	11.051	2.995	-5.625	1.00	15.00	A	O
ATOM	3455	N	VAL	A	447	12.662	4.481	-6.096	1.00	15.00	A	N
ATOM	3456	CA	VAL	A	447	13.270	3.650	-7.135	1.00	15.00	A	C
ATOM	3457	CB	VAL	A	447	14.376	4.409	-7.903	1.00	15.00	A	C
ATOM	3458	CG1	VAL	A	447	14.913	3.569	-9.055	1.00	15.00	A	C
ATOM	3459	CG2	VAL	A	447	13.847	5.739	-8.417	1.00	15.00	A	C
ATOM	3460	C	VAL	A	447	13.827	2.340	-6.578	1.00	15.00	A	C
ATOM	3461	O	VAL	A	447	13.742	1.300	-7.227	1.00	15.00	A	O
ATOM	3462	N	GLY	A	448	14.374	2.394	-5.371	1.00	15.00	A	N
ATOM	3463	CA	GLY	A	448	14.949	1.208	-4.757	1.00	15.00	A	C
ATOM	3464	C	GLY	A	448	13.940	0.109	-4.464	1.00	15.00	A	C
ATOM	3465	O	GLY	A	448	14.262	-1.076	-4.558	1.00	15.00	A	O
ATOM	3466	N	THR	A	449	12.714	0.489	-4.132	1.00	15.00	A	N
ATOM	3467	CA	THR	A	449	11.684	-0.489	-3.806	1.00	15.00	A	C
ATOM	3468	CB	THR	A	449	10.597	0.100	-2.874	1.00	15.00	A	C
ATOM	3469	OG1	THR	A	449	9.913	-0.963	-2.203	1.00	15.00	A	O
ATOM	3470	CG2	THR	A	449	9.589	0.938	-3.649	1.00	15.00	A	C
ATOM	3471	C	THR	A	449	11.045	-1.122	-5.049	1.00	15.00	A	C
ATOM	3472	O	THR	A	449	10.419	-2.183	-4.959	1.00	15.00	A	O

ATOM	3473	N	LEU	A	450	11.225	-0.489	-6.204	1.00	15.00	A	N
ATOM	3474	CA	LEU	A	450	10.646	-0.982	-7.455	1.00	15.00	A	C
ATOM	3475	CB	LEU	A	450	10.825	0.025	-8.596	1.00	15.00	A	C
ATOM	3476	CG	LEU	A	450	10.127	1.376	-8.411	1.00	15.00	A	C
ATOM	3477	CD1	LEU	A	450	10.541	2.350	-9.501	1.00	15.00	A	C
ATOM	3478	CD2	LEU	A	450	8.615	1.201	-8.393	1.00	15.00	A	C
ATOM	3479	C	LEU	A	450	11.130	-2.388	-7.850	1.00	15.00	A	C
ATOM	3480	O	LEU	A	450	10.311	-3.298	-8.002	1.00	15.00	A	O
ATOM	3481	N	PRO	A	451	12.456	-2.605	-8.018	1.00	15.00	A	N
ATOM	3482	CA	PRO	A	451	12.994	-3.919	-8.401	1.00	15.00	A	C
ATOM	3483	CB	PRO	A	451	14.505	-3.686	-8.502	1.00	15.00	A	C
ATOM	3484	CG	PRO	A	451	14.644	-2.214	-8.667	1.00	15.00	A	C
ATOM	3485	CD	PRO	A	451	13.532	-1.614	-7.864	1.00	15.00	A	C
ATOM	3486	C	PRO	A	451	12.695	-4.987	-7.357	1.00	15.00	A	C
ATOM	3487	O	PRO	A	451	12.397	-6.133	-7.697	1.00	15.00	A	O
ATOM	3488	N	PHE	A	452	12.770	-4.602	-6.086	1.00	15.00	A	N
ATOM	3489	CA	PHE	A	452	12.500	-5.523	-4.992	1.00	15.00	A	C
ATOM	3490	CB	PHE	A	452	12.742	-4.845	-3.639	1.00	15.00	A	C
ATOM	3491	CG	PHE	A	452	12.582	-5.762	-2.457	1.00	15.00	A	C
ATOM	3492	CD1	PHE	A	452	11.374	-5.844	-1.782	1.00	15.00	A	C
ATOM	3493	CD2	PHE	A	452	13.639	-6.541	-2.019	1.00	15.00	A	C
ATOM	3494	CE1	PHE	A	452	11.223	-6.683	-0.695	1.00	15.00	A	C
ATOM	3495	CE2	PHE	A	452	13.495	-7.383	-0.933	1.00	15.00	A	C
ATOM	3496	CZ	PHE	A	452	12.286	-7.454	-0.269	1.00	15.00	A	C
ATOM	3497	C	PHE	A	452	11.068	-6.040	-5.072	1.00	15.00	A	C
ATOM	3498	O	PHE	A	452	10.823	-7.237	-4.933	1.00	15.00	A	O
ATOM	3499	N	THR	A	453	10.136	-5.128	-5.316	1.00	15.00	A	N
ATOM	3500	CA	THR	A	453	8.727	-5.476	-5.415	1.00	15.00	A	C
ATOM	3501	CB	THR	A	453	7.855	-4.210	-5.541	1.00	15.00	A	C
ATOM	3502	OG1	THR	A	453	8.041	-3.386	-4.380	1.00	15.00	A	O
ATOM	3503	CG2	THR	A	453	6.386	-4.571	-5.674	1.00	15.00	A	C
ATOM	3504	C	THR	A	453	8.467	-6.408	-6.598	1.00	15.00	A	C
ATOM	3505	O	THR	A	453	7.855	-7.466	-6.441	1.00	15.00	A	O
ATOM	3506	N	TYR	A	454	8.953	-6.018	-7.773	1.00	15.00	A	N
ATOM	3507	CA	TYR	A	454	8.768	-6.817	-8.978	1.00	15.00	A	C
ATOM	3508	CB	TYR	A	454	9.372	-6.110	-10.198	1.00	15.00	A	C
ATOM	3509	CG	TYR	A	454	9.596	-7.025	-11.383	1.00	15.00	A	C
ATOM	3510	CD1	TYR	A	454	8.524	-7.584	-12.068	1.00	15.00	A	C
ATOM	3511	CD2	TYR	A	454	10.880	-7.344	-11.805	1.00	15.00	A	C
ATOM	3512	CE1	TYR	A	454	8.726	-8.436	-13.137	1.00	15.00	A	C
ATOM	3513	CE2	TYR	A	454	11.091	-8.190	-12.875	1.00	15.00	A	C
ATOM	3514	CZ	TYR	A	454	10.012	-8.735	-13.536	1.00	15.00	A	C
ATOM	3515	OH	TYR	A	454	10.222	-9.587	-14.595	1.00	15.00	A	O
ATOM	3516	C	TYR	A	454	9.360	-8.218	-8.825	1.00	15.00	A	C
ATOM	3517	O	TYR	A	454	8.707	-9.213	-9.134	1.00	15.00	A	O
ATOM	3518	N	MET	A	455	10.592	-8.285	-8.338	1.00	15.00	A	N
ATOM	3519	CA	MET	A	455	11.283	-9.558	-8.164	1.00	15.00	A	C
ATOM	3520	CB	MET	A	455	12.739	-9.316	-7.753	1.00	15.00	A	C
ATOM	3521	CG	MET	A	455	13.556	-10.575	-7.489	1.00	15.00	A	C
ATOM	3522	SD	MET	A	455	13.557	-11.073	-5.752	1.00	15.00	A	S
ATOM	3523	CE	MET	A	455	13.568	-9.484	-4.924	1.00	15.00	A	C
ATOM	3524	C	MET	A	455	10.576	-10.461	-7.157	1.00	15.00	A	C
ATOM	3525	O	MET	A	455	10.369	-11.648	-7.420	1.00	15.00	A	O
ATOM	3526	N	LEU	A	456	10.188	-9.892	-6.020	1.00	15.00	A	N
ATOM	3527	CA	LEU	A	456	9.520	-10.655	-4.974	1.00	15.00	A	C
ATOM	3528	CB	LEU	A	456	9.320	-9.802	-3.718	1.00	15.00	A	C
ATOM	3529	CG	LEU	A	456	8.889	-10.552	-2.453	1.00	15.00	A	C
ATOM	3530	CD1	LEU	A	456	9.761	-10.154	-1.273	1.00	15.00	A	C
ATOM	3531	CD2	LEU	A	456	7.423	-10.289	-2.145	1.00	15.00	A	C
ATOM	3532	C	LEU	A	456	8.190	-11.222	-5.455	1.00	15.00	A	C
ATOM	3533	O	LEU	A	456	7.904	-12.405	-5.258	1.00	15.00	A	O
ATOM	3534	N	GLU	A	457	7.386	-10.384	-6.098	1.00	15.00	A	N
ATOM	3535	CA	GLU	A	457	6.088	-10.818	-6.594	1.00	15.00	A	C
ATOM	3536	CB	GLU	A	457	5.226	-9.629	-7.024	1.00	15.00	A	C
ATOM	3537	CG	GLU	A	457	3.731	-9.899	-6.940	1.00	15.00	A	C
ATOM	3538	CD	GLU	A	457	3.211	-9.933	-5.512	1.00	15.00	A	C
ATOM	3539	OE1	GLU	A	457	2.198	-9.263	-5.232	1.00	15.00	A	O
ATOM	3540	OE2	GLU	A	457	3.816	-10.618	-4.662	1.00	15.00	A	O
ATOM	3541	C	GLU	A	457	6.236	-11.825	-7.730	1.00	15.00	A	C
ATOM	3542	O	GLU	A	457	5.495	-12.808	-7.798	1.00	15.00	A	O
ATOM	3543	N	LYS	A	458	7.203	-11.587	-8.615	1.00	15.00	A	N

ATOM	3544	CA	LYS	A	458	7.450	-12.488	-9.736	1.00	15.00	A	C
ATOM	3545	CB	LYS	A	458	8.608	-11.981	-10.603	1.00	15.00	A	C
ATOM	3546	CG	LYS	A	458	8.521	-12.394	-12.065	1.00	15.00	A	C
ATOM	3547	CD	LYS	A	458	9.860	-12.901	-12.583	1.00	15.00	A	C
ATOM	3548	CE	LYS	A	458	9.959	-12.782	-14.099	1.00	15.00	A	C
ATOM	3549	NZ	LYS	A	458	8.708	-13.205	-14.789	1.00	15.00	A	N
ATOM	3550	C	LYS	A	458	7.763	-13.887	-9.222	1.00	15.00	A	C
ATOM	3551	O	LYS	A	458	7.259	-14.877	-9.750	1.00	15.00	A	O
ATOM	3552	N	TRP	A	459	8.588	-13.952	-8.179	1.00	15.00	A	N
ATOM	3553	CA	TRP	A	459	8.967	-15.222	-7.570	1.00	15.00	A	C
ATOM	3554	CB	TRP	A	459	9.906	-14.977	-6.381	1.00	15.00	A	C
ATOM	3555	CG	TRP	A	459	10.407	-16.228	-5.719	1.00	15.00	A	C
ATOM	3556	CD1	TRP	A	459	11.476	-16.986	-6.100	1.00	15.00	A	C
ATOM	3557	CD2	TRP	A	459	9.863	-16.863	-4.553	1.00	15.00	A	C
ATOM	3558	NE1	TRP	A	459	11.629	-18.054	-5.249	1.00	15.00	A	N
ATOM	3559	CE2	TRP	A	459	10.652	-18.000	-4.291	1.00	15.00	A	C
ATOM	3560	CE3	TRP	A	459	8.786	-16.581	-3.707	1.00	15.00	A	C
ATOM	3561	CZ2	TRP	A	459	10.398	-18.854	-3.219	1.00	15.00	A	C
ATOM	3562	CZ3	TRP	A	459	8.535	-17.429	-2.645	1.00	15.00	A	C
ATOM	3563	CH2	TRP	A	459	9.337	-18.552	-2.410	1.00	15.00	A	C
ATOM	3564	C	TRP	A	459	7.727	-15.978	-7.111	1.00	15.00	A	C
ATOM	3565	O	TRP	A	459	7.562	-17.162	-7.403	1.00	15.00	A	O
ATOM	3566	N	ARG	A	460	6.844	-15.270	-6.418	1.00	15.00	A	N
ATOM	3567	CA	ARG	A	460	5.614	-15.859	-5.907	1.00	15.00	A	C
ATOM	3568	CB	ARG	A	460	4.875	-14.858	-5.024	1.00	15.00	A	C
ATOM	3569	CG	ARG	A	460	5.736	-14.284	-3.916	1.00	15.00	A	C
ATOM	3570	CD	ARG	A	460	5.128	-13.020	-3.348	1.00	15.00	A	C
ATOM	3571	NE	ARG	A	460	4.473	-13.259	-2.067	1.00	15.00	A	N
ATOM	3572	CZ	ARG	A	460	3.571	-12.440	-1.534	1.00	15.00	A	C
ATOM	3573	NH1	ARG	A	460	3.209	-11.332	-2.170	1.00	15.00	A	N
ATOM	3574	NH2	ARG	A	460	3.020	-12.734	-0.365	1.00	15.00	A	N
ATOM	3575	C	ARG	A	460	4.715	-16.331	-7.041	1.00	15.00	A	C
ATOM	3576	O	ARG	A	460	4.186	-17.436	-6.992	1.00	15.00	A	O
ATOM	3577	N	TRP	A	461	4.557	-15.494	-8.061	1.00	15.00	A	N
ATOM	3578	CA	TRP	A	461	3.723	-15.833	-9.212	1.00	15.00	A	C
ATOM	3579	CB	TRP	A	461	3.740	-14.703	-10.244	1.00	15.00	A	C
ATOM	3580	CG	TRP	A	461	2.901	-13.517	-9.877	1.00	15.00	A	C
ATOM	3581	CD1	TRP	A	461	1.959	-13.444	-8.891	1.00	15.00	A	C
ATOM	3582	CD2	TRP	A	461	2.926	-12.230	-10.504	1.00	15.00	A	C
ATOM	3583	NE1	TRP	A	461	1.394	-12.191	-8.868	1.00	15.00	A	N
ATOM	3584	CE2	TRP	A	461	1.974	-11.426	-9.847	1.00	15.00	A	C
ATOM	3585	CE3	TRP	A	461	3.664	-11.680	-11.557	1.00	15.00	A	C
ATOM	3586	CZ2	TRP	A	461	1.741	-10.101	-10.209	1.00	15.00	A	C
ATOM	3587	CZ3	TRP	A	461	3.431	-10.366	-11.914	1.00	15.00	A	C
ATOM	3588	CH2	TRP	A	461	2.478	-9.591	-11.243	1.00	15.00	A	C
ATOM	3589	C	TRP	A	461	4.197	-17.127	-9.864	1.00	15.00	A	C
ATOM	3590	O	TRP	A	461	3.403	-18.031	-10.129	1.00	15.00	A	O
ATOM	3591	N	MET	A	462	5.499	-17.213	-10.102	1.00	15.00	A	N
ATOM	3592	CA	MET	A	462	6.085	-18.394	-10.719	1.00	15.00	A	C
ATOM	3593	CB	MET	A	462	7.557	-18.145	-11.066	1.00	15.00	A	C
ATOM	3594	CG	MET	A	462	7.763	-17.016	-12.068	1.00	15.00	A	C
ATOM	3595	SD	MET	A	462	9.501	-16.638	-12.377	1.00	15.00	A	S
ATOM	3596	CE	MET	A	462	10.079	-16.358	-10.711	1.00	15.00	A	C
ATOM	3597	C	MET	A	462	5.932	-19.608	-9.807	1.00	15.00	A	C
ATOM	3598	O	MET	A	462	5.629	-20.712	-10.265	1.00	15.00	A	O
ATOM	3599	N	VAL	A	463	6.119	-19.394	-8.509	1.00	15.00	A	N
ATOM	3600	CA	VAL	A	463	5.984	-20.464	-7.525	1.00	15.00	A	C
ATOM	3601	CB	VAL	A	463	6.446	-20.001	-6.119	1.00	15.00	A	C
ATOM	3602	CG1	VAL	A	463	5.844	-20.863	-5.017	1.00	15.00	A	C
ATOM	3603	CG2	VAL	A	463	7.964	-20.024	-6.028	1.00	15.00	A	C
ATOM	3604	C	VAL	A	463	4.541	-20.967	-7.466	1.00	15.00	A	C
ATOM	3605	O	VAL	A	463	4.297	-22.173	-7.410	1.00	15.00	A	O
ATOM	3606	N	PHE	A	464	3.591	-20.038	-7.506	1.00	15.00	A	N
ATOM	3607	CA	PHE	A	464	2.175	-20.383	-7.444	1.00	15.00	A	C
ATOM	3608	CB	PHE	A	464	1.305	-19.139	-7.253	1.00	15.00	A	C
ATOM	3609	CG	PHE	A	464	1.403	-18.544	-5.876	1.00	15.00	A	C
ATOM	3610	CD1	PHE	A	464	1.517	-19.361	-4.763	1.00	15.00	A	C
ATOM	3611	CD2	PHE	A	464	1.386	-17.171	-5.694	1.00	15.00	A	C
ATOM	3612	CE1	PHE	A	464	1.611	-18.821	-3.495	1.00	15.00	A	C
ATOM	3613	CE2	PHE	A	464	1.479	-16.624	-4.428	1.00	15.00	A	C
ATOM	3614	CZ	PHE	A	464	1.594	-17.451	-3.327	1.00	15.00	A	C

ATOM	3615	C	PHE	A	464	1.723	-21.185	-8.658	1.00	15.00	A	C
ATOM	3616	O	PHE	A	464	0.914	-22.104	-8.536	1.00	15.00	A	O
ATOM	3617	N	LYS	A	465	2.236	-20.844	-9.829	1.00	15.00	A	N
ATOM	3618	CA	LYS	A	465	1.869	-21.566	-11.036	1.00	15.00	A	C
ATOM	3619	CB	LYS	A	465	2.241	-20.776	-12.285	1.00	15.00	A	C
ATOM	3620	CG	LYS	A	465	1.619	-21.328	-13.559	1.00	15.00	A	C
ATOM	3621	CD	LYS	A	465	1.634	-20.303	-14.677	1.00	15.00	A	C
ATOM	3622	CE	LYS	A	465	1.194	-18.937	-14.177	1.00	15.00	A	C
ATOM	3623	NZ	LYS	A	465	0.991	-17.983	-15.292	1.00	15.00	A	N
ATOM	3624	C	LYS	A	465	2.531	-22.936	-11.064	1.00	15.00	A	C
ATOM	3625	O	LYS	A	465	1.905	-23.933	-11.427	1.00	15.00	A	O
ATOM	3626	N	GLY	A	466	3.791	-22.976	-10.654	1.00	15.00	A	N
ATOM	3627	CA	GLY	A	466	4.534	-24.219	-10.643	1.00	15.00	A	C
ATOM	3628	C	GLY	A	466	5.778	-24.133	-11.502	1.00	15.00	A	C
ATOM	3629	O	GLY	A	466	6.350	-25.150	-11.888	1.00	15.00	A	O
ATOM	3630	N	GLU	A	467	6.197	-22.907	-11.789	1.00	15.00	A	N
ATOM	3631	CA	GLU	A	467	7.374	-22.660	-12.609	1.00	15.00	A	C
ATOM	3632	CB	GLU	A	467	7.297	-21.255	-13.223	1.00	15.00	A	C
ATOM	3633	CG	GLU	A	467	6.066	-21.036	-14.102	1.00	15.00	A	C
ATOM	3634	CD	GLU	A	467	5.732	-19.570	-14.336	1.00	15.00	A	C
ATOM	3635	OE1	GLU	A	467	4.543	-19.259	-14.581	1.00	15.00	A	O
ATOM	3636	OE2	GLU	A	467	6.647	-18.729	-14.273	1.00	15.00	A	O
ATOM	3637	C	GLU	A	467	8.657	-22.837	-11.792	1.00	15.00	A	C
ATOM	3638	O	GLU	A	467	9.749	-22.972	-12.345	1.00	15.00	A	O
ATOM	3639	N	ILE	A	468	8.518	-22.844	-10.469	1.00	15.00	A	N
ATOM	3640	CA	ILE	A	468	9.662	-23.014	-9.578	1.00	15.00	A	C
ATOM	3641	CB	ILE	A	468	9.887	-21.780	-8.675	1.00	15.00	A	C
ATOM	3642	CG1	ILE	A	468	10.178	-20.541	-9.528	1.00	15.00	A	C
ATOM	3643	CG2	ILE	A	468	11.026	-22.030	-7.693	1.00	15.00	A	C
ATOM	3644	CD1	ILE	A	468	10.440	-19.288	-8.722	1.00	15.00	A	C
ATOM	3645	C	ILE	A	468	9.497	-24.264	-8.711	1.00	15.00	A	C
ATOM	3646	O	ILE	A	468	8.556	-24.364	-7.921	1.00	15.00	A	O
ATOM	3647	N	PRO	A	469	10.409	-25.239	-8.865	1.00	15.00	A	N
ATOM	3648	CA	PRO	A	469	10.383	-26.490	-8.098	1.00	15.00	A	C
ATOM	3649	CB	PRO	A	469	11.491	-27.338	-8.741	1.00	15.00	A	C
ATOM	3650	CG	PRO	A	469	11.812	-26.659	-10.029	1.00	15.00	A	C
ATOM	3651	CD	PRO	A	469	11.536	-25.204	-9.806	1.00	15.00	A	C
ATOM	3652	C	PRO	A	469	10.696	-26.254	-6.621	1.00	15.00	A	C
ATOM	3653	O	PRO	A	469	11.414	-25.317	-6.273	1.00	15.00	A	O
ATOM	3654	N	LYS	A	470	10.166	-27.121	-5.762	1.00	15.00	A	N
ATOM	3655	CA	LYS	A	470	10.370	-27.009	-4.316	1.00	15.00	A	C
ATOM	3656	CB	LYS	A	470	9.552	-28.075	-3.587	1.00	15.00	A	C
ATOM	3657	CG	LYS	A	470	8.175	-27.613	-3.137	1.00	15.00	A	C
ATOM	3658	CD	LYS	A	470	7.442	-28.708	-2.373	1.00	15.00	A	C
ATOM	3659	CE	LYS	A	470	8.349	-29.380	-1.352	1.00	15.00	A	C
ATOM	3660	NZ	LYS	A	470	7.605	-29.831	-0.147	1.00	15.00	A	N
ATOM	3661	C	LYS	A	470	11.842	-27.144	-3.940	1.00	15.00	A	C
ATOM	3662	O	LYS	A	470	12.299	-26.572	-2.951	1.00	15.00	A	O
ATOM	3663	N	ASP	A	471	12.563	-27.909	-4.743	1.00	15.00	A	N
ATOM	3664	CA	ASP	A	471	13.987	-28.154	-4.543	1.00	15.00	A	C
ATOM	3665	CB	ASP	A	471	14.391	-29.378	-5.365	1.00	15.00	A	C
ATOM	3666	CG	ASP	A	471	13.803	-29.323	-6.764	1.00	15.00	A	C
ATOM	3667	OD1	ASP	A	471	12.618	-29.705	-6.919	1.00	15.00	A	O
ATOM	3668	OD2	ASP	A	471	14.500	-28.867	-7.699	1.00	15.00	A	O
ATOM	3669	C	ASP	A	471	14.826	-26.966	-5.000	1.00	15.00	A	C
ATOM	3670	O	ASP	A	471	16.044	-26.954	-4.827	1.00	15.00	A	O
ATOM	3671	N	GLN	A	472	14.177	-25.969	-5.587	1.00	15.00	A	N
ATOM	3672	CA	GLN	A	472	14.880	-24.794	-6.077	1.00	15.00	A	C
ATOM	3673	CB	GLN	A	472	15.027	-24.859	-7.600	1.00	15.00	A	C
ATOM	3674	CG	GLN	A	472	16.025	-25.898	-8.086	1.00	15.00	A	C
ATOM	3675	CD	GLN	A	472	16.085	-25.980	-9.596	1.00	15.00	A	C
ATOM	3676	OE1	GLN	A	472	16.873	-25.280	-10.240	1.00	15.00	A	O
ATOM	3677	NE2	GLN	A	472	15.264	-26.845	-10.171	1.00	15.00	A	N
ATOM	3678	C	GLN	A	472	14.165	-23.510	-5.678	1.00	15.00	A	C
ATOM	3679	O	GLN	A	472	14.238	-22.503	-6.385	1.00	15.00	A	O
ATOM	3680	N	TRP	A	473	13.496	-23.540	-4.535	1.00	15.00	A	N
ATOM	3681	CA	TRP	A	473	12.765	-22.379	-4.047	1.00	15.00	A	C
ATOM	3682	CB	TRP	A	473	11.872	-22.765	-2.868	1.00	15.00	A	C
ATOM	3683	CG	TRP	A	473	10.449	-23.033	-3.245	1.00	15.00	A	C
ATOM	3684	CD1	TRP	A	473	9.964	-23.298	-4.492	1.00	15.00	A	C
ATOM	3685	CD2	TRP	A	473	9.323	-23.058	-2.364	1.00	15.00	A	C

ATOM	3686	NE1	TRP	A	473	8.607	-23.492	-4.442	1.00	15.00	A	N
ATOM	3687	CE2	TRP	A	473	8.188	-23.349	-3.145	1.00	15.00	A	C
ATOM	3688	CE3	TRP	A	473	9.166	-22.864	-0.989	1.00	15.00	A	C
ATOM	3689	CZ2	TRP	A	473	6.912	-23.450	-2.595	1.00	15.00	A	C
ATOM	3690	CZ3	TRP	A	473	7.900	-22.964	-0.446	1.00	15.00	A	C
ATOM	3691	CH2	TRP	A	473	6.790	-23.255	-1.248	1.00	15.00	A	C
ATOM	3692	C	TRP	A	473	13.723	-21.275	-3.625	1.00	15.00	A	C
ATOM	3693	O	TRP	A	473	13.620	-20.134	-4.084	1.00	15.00	A	O
ATOM	3694	N	MET	A	474	14.665	-21.629	-2.764	1.00	15.00	A	N
ATOM	3695	CA	MET	A	474	15.642	-20.672	-2.269	1.00	15.00	A	C
ATOM	3696	CB	MET	A	474	16.343	-21.195	-1.014	1.00	15.00	A	C
ATOM	3697	CG	MET	A	474	15.456	-21.252	0.218	1.00	15.00	A	C
ATOM	3698	SD	MET	A	474	14.433	-19.778	0.421	1.00	15.00	A	S
ATOM	3699	CE	MET	A	474	15.651	-18.489	0.174	1.00	15.00	A	C
ATOM	3700	C	MET	A	474	16.666	-20.348	-3.338	1.00	15.00	A	C
ATOM	3701	O	MET	A	474	17.100	-19.202	-3.470	1.00	15.00	A	O
ATOM	3702	N	LYS	A	475	17.045	-21.365	-4.096	1.00	15.00	A	N
ATOM	3703	CA	LYS	A	475	18.013	-21.205	-5.165	1.00	15.00	A	C
ATOM	3704	CB	LYS	A	475	18.246	-22.542	-5.868	1.00	15.00	A	C
ATOM	3705	CG	LYS	A	475	19.361	-22.513	-6.895	1.00	15.00	A	C
ATOM	3706	CD	LYS	A	475	19.245	-23.678	-7.858	1.00	15.00	A	C
ATOM	3707	CE	LYS	A	475	19.969	-23.381	-9.156	1.00	15.00	A	C
ATOM	3708	NZ	LYS	A	475	19.460	-24.208	-10.279	1.00	15.00	A	N
ATOM	3709	C	LYS	A	475	17.550	-20.155	-6.173	1.00	15.00	A	C
ATOM	3710	O	LYS	A	475	18.244	-19.167	-6.411	1.00	15.00	A	O
ATOM	3711	N	LYS	A	476	16.359	-20.359	-6.732	1.00	15.00	A	N
ATOM	3712	CA	LYS	A	476	15.805	-19.438	-7.722	1.00	15.00	A	C
ATOM	3713	CB	LYS	A	476	14.470	-19.961	-8.259	1.00	15.00	A	C
ATOM	3714	CG	LYS	A	476	14.465	-20.224	-9.756	1.00	15.00	A	C
ATOM	3715	CD	LYS	A	476	14.671	-18.935	-10.536	1.00	15.00	A	C
ATOM	3716	CE	LYS	A	476	15.017	-19.206	-11.990	1.00	15.00	A	C
ATOM	3717	NZ	LYS	A	476	15.399	-17.956	-12.701	1.00	15.00	A	N
ATOM	3718	C	LYS	A	476	15.634	-18.036	-7.147	1.00	15.00	A	C
ATOM	3719	O	LYS	A	476	15.790	-17.038	-7.856	1.00	15.00	A	O
ATOM	3720	N	TRP	A	477	15.322	-17.973	-5.859	1.00	15.00	A	N
ATOM	3721	CA	TRP	A	477	15.135	-16.705	-5.170	1.00	15.00	A	C
ATOM	3722	CB	TRP	A	477	14.717	-16.957	-3.717	1.00	15.00	A	C
ATOM	3723	CG	TRP	A	477	14.727	-15.735	-2.849	1.00	15.00	A	C
ATOM	3724	CD1	TRP	A	477	15.611	-15.450	-1.851	1.00	15.00	A	C
ATOM	3725	CD2	TRP	A	477	13.808	-14.638	-2.896	1.00	15.00	A	C
ATOM	3726	NE1	TRP	A	477	15.301	-14.246	-1.271	1.00	15.00	A	N
ATOM	3727	CE2	TRP	A	477	14.197	-13.726	-1.895	1.00	15.00	A	C
ATOM	3728	CE3	TRP	A	477	12.695	-14.340	-3.686	1.00	15.00	A	C
ATOM	3729	CZ2	TRP	A	477	13.511	-12.537	-1.665	1.00	15.00	A	C
ATOM	3730	CZ3	TRP	A	477	12.016	-13.160	-3.456	1.00	15.00	A	C
ATOM	3731	CH2	TRP	A	477	12.426	-12.272	-2.455	1.00	15.00	A	C
ATOM	3732	C	TRP	A	477	16.407	-15.864	-5.219	1.00	15.00	A	C
ATOM	3733	O	TRP	A	477	16.373	-14.685	-5.577	1.00	15.00	A	O
ATOM	3734	N	TRP	A	478	17.533	-16.478	-4.884	1.00	15.00	A	N
ATOM	3735	CA	TRP	A	478	18.800	-15.766	-4.887	1.00	15.00	A	C
ATOM	3736	CB	TRP	A	478	19.803	-16.364	-3.903	1.00	15.00	A	C
ATOM	3737	CG	TRP	A	478	19.431	-16.027	-2.492	1.00	15.00	A	C
ATOM	3738	CD1	TRP	A	478	18.959	-16.877	-1.534	1.00	15.00	A	C
ATOM	3739	CD2	TRP	A	478	19.465	-14.726	-1.893	1.00	15.00	A	C
ATOM	3740	NE1	TRP	A	478	18.695	-16.181	-0.377	1.00	15.00	A	N
ATOM	3741	CE2	TRP	A	478	18.998	-14.861	-0.574	1.00	15.00	A	C
ATOM	3742	CE3	TRP	A	478	19.841	-13.459	-2.348	1.00	15.00	A	C
ATOM	3743	CZ2	TRP	A	478	18.905	-13.779	0.298	1.00	15.00	A	C
ATOM	3744	CZ3	TRP	A	478	19.749	-12.387	-1.483	1.00	15.00	A	C
ATOM	3745	CH2	TRP	A	478	19.279	-12.552	-0.176	1.00	15.00	A	C
ATOM	3746	C	TRP	A	478	19.353	-15.571	-6.291	1.00	15.00	A	C
ATOM	3747	O	TRP	A	478	20.123	-14.640	-6.540	1.00	15.00	A	O
ATOM	3748	N	GLU	A	479	18.943	-16.438	-7.213	1.00	15.00	A	N
ATOM	3749	CA	GLU	A	479	19.359	-16.313	-8.602	1.00	15.00	A	C
ATOM	3750	CB	GLU	A	479	18.788	-17.458	-9.449	1.00	15.00	A	C
ATOM	3751	CG	GLU	A	479	19.467	-18.808	-9.267	1.00	15.00	A	C
ATOM	3752	CD	GLU	A	479	18.778	-19.904	-10.061	1.00	15.00	A	C
ATOM	3753	OE1	GLU	A	479	17.562	-19.788	-10.302	1.00	15.00	A	O
ATOM	3754	OE2	GLU	A	479	19.449	-20.885	-10.447	1.00	15.00	A	O
ATOM	3755	C	GLU	A	479	18.826	-14.990	-9.140	1.00	15.00	A	C
ATOM	3756	O	GLU	A	479	19.567	-14.181	-9.696	1.00	15.00	A	O

ATOM	3757	N	MET	A	480	17.535	-14.762	-8.914	1.00	15.00	A	N
ATOM	3758	CA	MET	A	480	16.869	-13.549	-9.373	1.00	15.00	A	C
ATOM	3759	CB	MET	A	480	15.355	-13.738	-9.372	1.00	15.00	A	C
ATOM	3760	CG	MET	A	480	14.819	-14.448	-10.601	1.00	15.00	A	C
ATOM	3761	SD	MET	A	480	13.296	-15.352	-10.265	1.00	15.00	A	S
ATOM	3762	CE	MET	A	480	12.408	-14.156	-9.266	1.00	15.00	A	C
ATOM	3763	C	MET	A	480	17.244	-12.334	-8.531	1.00	15.00	A	C
ATOM	3764	O	MET	A	480	17.205	-11.204	-9.014	1.00	15.00	A	O
ATOM	3765	N	LYS	A	481	17.596	-12.561	-7.270	1.00	15.00	A	N
ATOM	3766	CA	LYS	A	481	17.985	-11.469	-6.384	1.00	15.00	A	C
ATOM	3767	CB	LYS	A	481	18.221	-11.973	-4.960	1.00	15.00	A	C
ATOM	3768	CG	LYS	A	481	17.082	-11.682	-3.992	1.00	15.00	A	C
ATOM	3769	CD	LYS	A	481	17.164	-10.264	-3.439	1.00	15.00	A	C
ATOM	3770	CE	LYS	A	481	16.242	-10.086	-2.241	1.00	15.00	A	C
ATOM	3771	NZ	LYS	A	481	16.386	-8.741	-1.613	1.00	15.00	A	N
ATOM	3772	C	LYS	A	481	19.232	-10.771	-6.912	1.00	15.00	A	C
ATOM	3773	O	LYS	A	481	19.310	-9.542	-6.924	1.00	15.00	A	O
ATOM	3774	N	ARG	A	482	20.199	-11.563	-7.358	1.00	15.00	A	N
ATOM	3775	CA	ARG	A	482	21.438	-11.022	-7.904	1.00	15.00	A	C
ATOM	3776	CB	ARG	A	482	22.523	-12.105	-7.946	1.00	15.00	A	C
ATOM	3777	CG	ARG	A	482	22.815	-12.826	-6.638	1.00	15.00	A	C
ATOM	3778	CD	ARG	A	482	23.706	-14.029	-6.913	1.00	15.00	A	C
ATOM	3779	NE	ARG	A	482	24.177	-14.712	-5.709	1.00	15.00	A	N
ATOM	3780	CZ	ARG	A	482	25.320	-15.401	-5.656	1.00	15.00	A	C
ATOM	3781	NH1	ARG	A	482	26.100	-15.487	-6.732	1.00	15.00	A	N
ATOM	3782	NH2	ARG	A	482	25.658	-16.041	-4.548	1.00	15.00	A	N
ATOM	3783	C	ARG	A	482	21.206	-10.531	-9.330	1.00	15.00	A	C
ATOM	3784	O	ARG	A	482	21.803	-9.556	-9.772	1.00	15.00	A	O
ATOM	3785	N	GLU	A	483	20.322	-11.225	-10.033	1.00	15.00	A	N
ATOM	3786	CA	GLU	A	483	20.005	-10.927	-11.426	1.00	15.00	A	C
ATOM	3787	CB	GLU	A	483	19.267	-12.132	-12.024	1.00	15.00	A	C
ATOM	3788	CG	GLU	A	483	18.714	-11.948	-13.426	1.00	15.00	A	C
ATOM	3789	CD	GLU	A	483	17.888	-13.144	-13.861	1.00	15.00	A	C
ATOM	3790	OE1	GLU	A	483	16.757	-13.305	-13.348	1.00	15.00	A	O
ATOM	3791	OE2	GLU	A	483	18.374	-13.931	-14.698	1.00	15.00	A	O
ATOM	3792	C	GLU	A	483	19.187	-9.646	-11.611	1.00	15.00	A	C
ATOM	3793	O	GLU	A	483	19.675	-8.662	-12.169	1.00	15.00	A	O
ATOM	3794	N	ILE	A	484	17.954	-9.663	-11.128	1.00	15.00	A	N
ATOM	3795	CA	ILE	A	484	17.041	-8.537	-11.284	1.00	15.00	A	C
ATOM	3796	CB	ILE	A	484	15.578	-8.984	-11.055	1.00	15.00	A	C
ATOM	3797	CG1	ILE	A	484	15.236	-10.178	-11.953	1.00	15.00	A	C
ATOM	3798	CG2	ILE	A	484	14.612	-7.833	-11.305	1.00	15.00	A	C
ATOM	3799	CD1	ILE	A	484	13.923	-10.849	-11.611	1.00	15.00	A	C
ATOM	3800	C	ILE	A	484	17.353	-7.357	-10.363	1.00	15.00	A	C
ATOM	3801	O	ILE	A	484	17.374	-6.208	-10.804	1.00	15.00	A	O
ATOM	3802	N	VAL	A	485	17.599	-7.636	-9.091	1.00	15.00	A	N
ATOM	3803	CA	VAL	A	485	17.849	-6.571	-8.121	1.00	15.00	A	C
ATOM	3804	CB	VAL	A	485	17.262	-6.918	-6.734	1.00	15.00	A	C
ATOM	3805	CG1	VAL	A	485	16.936	-5.653	-5.952	1.00	15.00	A	C
ATOM	3806	CG2	VAL	A	485	16.024	-7.789	-6.875	1.00	15.00	A	C
ATOM	3807	C	VAL	A	485	19.330	-6.222	-7.976	1.00	15.00	A	C
ATOM	3808	O	VAL	A	485	19.673	-5.126	-7.528	1.00	15.00	A	O
ATOM	3809	N	GLY	A	486	20.205	-7.139	-8.353	1.00	15.00	A	N
ATOM	3810	CA	GLY	A	486	21.627	-6.887	-8.224	1.00	15.00	A	C
ATOM	3811	C	GLY	A	486	22.059	-6.932	-6.772	1.00	15.00	A	C
ATOM	3812	O	GLY	A	486	22.957	-6.197	-6.348	1.00	15.00	A	O
ATOM	3813	N	VAL	A	487	21.404	-7.794	-6.009	1.00	15.00	A	N
ATOM	3814	CA	VAL	A	487	21.693	-7.956	-4.594	1.00	15.00	A	C
ATOM	3815	CB	VAL	A	487	20.431	-7.701	-3.744	1.00	15.00	A	C
ATOM	3816	CG1	VAL	A	487	20.697	-7.989	-2.276	1.00	15.00	A	C
ATOM	3817	CG2	VAL	A	487	19.945	-6.272	-3.929	1.00	15.00	A	C
ATOM	3818	C	VAL	A	487	22.199	-9.369	-4.335	1.00	15.00	A	C
ATOM	3819	O	VAL	A	487	21.557	-10.342	-4.725	1.00	15.00	A	O
ATOM	3820	N	VAL	A	488	23.335	-9.477	-3.667	1.00	15.00	A	N
ATOM	3821	CA	VAL	A	488	23.928	-10.774	-3.388	1.00	15.00	A	C
ATOM	3822	CB	VAL	A	488	25.381	-10.850	-3.911	1.00	15.00	A	C
ATOM	3823	CG1	VAL	A	488	26.294	-9.882	-3.171	1.00	15.00	A	C
ATOM	3824	CG2	VAL	A	488	25.912	-12.270	-3.847	1.00	15.00	A	C
ATOM	3825	C	VAL	A	488	23.868	-11.122	-1.899	1.00	15.00	A	C
ATOM	3826	O	VAL	A	488	23.992	-10.248	-1.033	1.00	15.00	A	O
ATOM	3827	N	GLU	A	489	23.662	-12.399	-1.613	1.00	15.00	A	N

ATOM	3828	CA	GLU	A	489	23.583	-12.882	-0.247	1.00	15.00	A	C
ATOM	3829	CB	GLU	A	489	22.679	-14.125	-0.163	1.00	15.00	A	C
ATOM	3830	CG	GLU	A	489	23.321	-15.449	-0.568	1.00	15.00	A	C
ATOM	3831	CD	GLU	A	489	23.628	-15.582	-2.053	1.00	15.00	A	C
ATOM	3832	OE1	GLU	A	489	24.243	-16.597	-2.434	1.00	15.00	A	O
ATOM	3833	OE2	GLU	A	489	23.275	-14.680	-2.845	1.00	15.00	A	O
ATOM	3834	C	GLU	A	489	24.977	-13.163	0.314	1.00	15.00	A	C
ATOM	3835	O	GLU	A	489	25.836	-13.712	-0.375	1.00	15.00	A	O
ATOM	3836	N	PRO	A	490	25.230	-12.756	1.566	1.00	15.00	A	N
ATOM	3837	CA	PRO	A	490	26.527	-12.967	2.216	1.00	15.00	A	C
ATOM	3838	CB	PRO	A	490	26.485	-11.988	3.389	1.00	15.00	A	C
ATOM	3839	CG	PRO	A	490	25.037	-11.871	3.727	1.00	15.00	A	C
ATOM	3840	CD	PRO	A	490	24.283	-12.033	2.435	1.00	15.00	A	C
ATOM	3841	C	PRO	A	490	26.706	-14.398	2.722	1.00	15.00	A	C
ATOM	3842	O	PRO	A	490	27.829	-14.870	2.888	1.00	15.00	A	O
ATOM	3843	N	VAL	A	491	25.597	-15.083	2.964	1.00	15.00	A	N
ATOM	3844	CA	VAL	A	491	25.634	-16.452	3.456	1.00	15.00	A	C
ATOM	3845	CB	VAL	A	491	25.012	-16.563	4.868	1.00	15.00	A	C
ATOM	3846	CG1	VAL	A	491	25.131	-17.984	5.406	1.00	15.00	A	C
ATOM	3847	CG2	VAL	A	491	25.660	-15.574	5.827	1.00	15.00	A	C
ATOM	3848	C	VAL	A	491	24.880	-17.379	2.507	1.00	15.00	A	C
ATOM	3849	O	VAL	A	491	23.712	-17.138	2.201	1.00	15.00	A	O
ATOM	3850	N	PRO	A	492	25.544	-18.443	2.021	1.00	15.00	A	N
ATOM	3851	CA	PRO	A	492	24.925	-19.417	1.111	1.00	15.00	A	C
ATOM	3852	CB	PRO	A	492	26.048	-20.424	0.831	1.00	15.00	A	C
ATOM	3853	CG	PRO	A	492	27.031	-20.229	1.937	1.00	15.00	A	C
ATOM	3854	CD	PRO	A	492	26.944	-18.780	2.319	1.00	15.00	A	C
ATOM	3855	C	PRO	A	492	23.741	-20.113	1.778	1.00	15.00	A	C
ATOM	3856	O	PRO	A	492	23.909	-20.881	2.725	1.00	15.00	A	O
ATOM	3857	N	HIS	A	493	22.544	-19.833	1.288	1.00	15.00	A	N
ATOM	3858	CA	HIS	A	493	21.337	-20.415	1.855	1.00	15.00	A	C
ATOM	3859	CB	HIS	A	493	20.222	-19.375	1.945	1.00	15.00	A	C
ATOM	3860	CG	HIS	A	493	20.355	-18.446	3.109	1.00	15.00	A	C
ATOM	3861	ND1	HIS	A	493	19.397	-17.516	3.437	1.00	15.00	A	N
ATOM	3862	CD2	HIS	A	493	21.341	-18.304	4.027	1.00	15.00	A	C
ATOM	3863	CE1	HIS	A	493	19.785	-16.844	4.500	1.00	15.00	A	C
ATOM	3864	NE2	HIS	A	493	20.957	-17.302	4.875	1.00	15.00	A	N
ATOM	3865	C	HIS	A	493	20.864	-21.619	1.062	1.00	15.00	A	C
ATOM	3866	O	HIS	A	493	20.755	-21.566	-0.162	1.00	15.00	A	O
ATOM	3867	N	ASP	A	494	20.580	-22.698	1.773	1.00	15.00	A	N
ATOM	3868	CA	ASP	A	494	20.109	-23.925	1.155	1.00	15.00	A	C
ATOM	3869	CB	ASP	A	494	20.660	-25.141	1.900	1.00	15.00	A	C
ATOM	3870	CG	ASP	A	494	19.997	-25.351	3.243	1.00	15.00	A	C
ATOM	3871	OD1	ASP	A	494	20.051	-24.435	4.093	1.00	15.00	A	O
ATOM	3872	OD2	ASP	A	494	19.403	-26.423	3.448	1.00	15.00	A	O
ATOM	3873	C	ASP	A	494	18.580	-23.954	1.128	1.00	15.00	A	C
ATOM	3874	O	ASP	A	494	17.926	-22.976	1.500	1.00	15.00	A	O
ATOM	3875	N	GLU	A	495	18.015	-25.083	0.724	1.00	15.00	A	N
ATOM	3876	CA	GLU	A	495	16.568	-25.221	0.614	1.00	15.00	A	C
ATOM	3877	CB	GLU	A	495	16.185	-26.292	-0.406	1.00	15.00	A	C
ATOM	3878	CG	GLU	A	495	16.490	-25.905	-1.846	1.00	15.00	A	C
ATOM	3879	CD	GLU	A	495	15.807	-24.616	-2.272	1.00	15.00	A	C
ATOM	3880	OE1	GLU	A	495	14.653	-24.374	-1.848	1.00	15.00	A	O
ATOM	3881	OE2	GLU	A	495	16.417	-23.835	-3.032	1.00	15.00	A	O
ATOM	3882	C	GLU	A	495	15.847	-25.432	1.948	1.00	15.00	A	C
ATOM	3883	O	GLU	A	495	14.642	-25.655	1.968	1.00	15.00	A	O
ATOM	3884	N	THR	A	496	16.564	-25.380	3.065	1.00	15.00	A	N
ATOM	3885	CA	THR	A	496	15.909	-25.527	4.362	1.00	15.00	A	C
ATOM	3886	CB	THR	A	496	16.818	-26.172	5.430	1.00	15.00	A	C
ATOM	3887	OG1	THR	A	496	18.015	-25.401	5.595	1.00	15.00	A	O
ATOM	3888	CG2	THR	A	496	17.172	-27.602	5.051	1.00	15.00	A	C
ATOM	3889	C	THR	A	496	15.438	-24.160	4.848	1.00	15.00	A	C
ATOM	3890	O	THR	A	496	14.736	-24.044	5.854	1.00	15.00	A	O
ATOM	3891	N	TYR	A	497	15.833	-23.130	4.109	1.00	15.00	A	N
ATOM	3892	CA	TYR	A	497	15.479	-21.760	4.433	1.00	15.00	A	C
ATOM	3893	CB	TYR	A	497	16.593	-20.806	3.984	1.00	15.00	A	C
ATOM	3894	CG	TYR	A	497	17.775	-20.728	4.914	1.00	15.00	A	C
ATOM	3895	CD1	TYR	A	497	18.905	-21.501	4.694	1.00	15.00	A	C
ATOM	3896	CD2	TYR	A	497	17.763	-19.880	6.010	1.00	15.00	A	C
ATOM	3897	CE1	TYR	A	497	19.990	-21.435	5.543	1.00	15.00	A	C
ATOM	3898	CE2	TYR	A	497	18.844	-19.804	6.863	1.00	15.00	A	C

```
ATOM   3899  CZ  TYR A 497      19.955 -20.584   6.626  1.00 15.00      A    C
ATOM   3900  OH  TYR A 497      21.028 -20.527   7.482  1.00 15.00      A    O
ATOM   3901  C   TYR A 497      14.190 -21.340   3.745  1.00 15.00      A    C
ATOM   3902  O   TYR A 497      13.613 -22.080   2.943  1.00 15.00      A    O
ATOM   3903  N   CYS A 498      13.761 -20.135   4.066  1.00 15.00      A    N
ATOM   3904  CA  CYS A 498      12.572 -19.539   3.488  1.00 15.00      A    C
ATOM   3905  CB  CYS A 498      11.298 -20.087   4.133  1.00 15.00      A    C
ATOM   3906  SG  CYS A 498       9.911 -20.273   2.985  1.00 15.00      A    S
ATOM   3907  C   CYS A 498      12.671 -18.032   3.659  1.00 15.00      A    C
ATOM   3908  O   CYS A 498      11.825 -17.399   4.289  1.00 15.00      A    O
ATOM   3909  N   ASP A 499      13.738 -17.483   3.093  1.00 15.00      A    N
ATOM   3910  CA  ASP A 499      14.035 -16.050   3.161  1.00 15.00      A    C
ATOM   3911  CB  ASP A 499      15.290 -15.690   2.357  1.00 15.00      A    C
ATOM   3912  CG  ASP A 499      16.507 -16.506   2.752  1.00 15.00      A    C
ATOM   3913  OD1 ASP A 499      16.523 -17.069   3.862  1.00 15.00      A    O
ATOM   3914  OD2 ASP A 499      17.454 -16.593   1.947  1.00 15.00      A    O
ATOM   3915  C   ASP A 499      12.858 -15.155   2.763  1.00 15.00      A    C
ATOM   3916  O   ASP A 499      12.557 -14.203   3.474  1.00 15.00      A    O
ATOM   3917  N   PRO A 500      12.181 -15.417   1.622  1.00 15.00      A    N
ATOM   3918  CA  PRO A 500      11.038 -14.598   1.200  1.00 15.00      A    C
ATOM   3919  CB  PRO A 500      10.514 -15.314  -0.049  1.00 15.00      A    C
ATOM   3920  CG  PRO A 500      11.669 -16.102  -0.554  1.00 15.00      A    C
ATOM   3921  CD  PRO A 500      12.474 -16.489   0.650  1.00 15.00      A    C
ATOM   3922  C   PRO A 500       9.945 -14.554   2.266  1.00 15.00      A    C
ATOM   3923  O   PRO A 500       9.277 -13.540   2.440  1.00 15.00      A    O
ATOM   3924  N   ALA A 501       9.800 -15.649   3.003  1.00 15.00      A    N
ATOM   3925  CA  ALA A 501       8.780 -15.744   4.038  1.00 15.00      A    C
ATOM   3926  CB  ALA A 501       8.511 -17.197   4.397  1.00 15.00      A    C
ATOM   3927  C   ALA A 501       9.143 -14.936   5.280  1.00 15.00      A    C
ATOM   3928  O   ALA A 501       8.299 -14.710   6.145  1.00 15.00      A    O
ATOM   3929  N   SER A 502      10.386 -14.469   5.361  1.00 15.00      A    N
ATOM   3930  CA  SER A 502      10.820 -13.689   6.511  1.00 15.00      A    C
ATOM   3931  CB  SER A 502      12.344 -13.757   6.701  1.00 15.00      A    C
ATOM   3932  OG  SER A 502      13.026 -12.823   5.878  1.00 15.00      A    O
ATOM   3933  C   SER A 502      10.323 -12.245   6.418  1.00 15.00      A    C
ATOM   3934  O   SER A 502      10.716 -11.387   7.213  1.00 15.00      A    O
ATOM   3935  N   LEU A 503       9.467 -11.985   5.432  1.00 15.00      A    N
ATOM   3936  CA  LEU A 503       8.888 -10.665   5.228  1.00 15.00      A    C
ATOM   3937  CB  LEU A 503       9.100 -10.193   3.784  1.00 15.00      A    C
ATOM   3938  CG  LEU A 503       8.372  -8.906   3.374  1.00 15.00      A    C
ATOM   3939  CD1 LEU A 503       8.874  -7.713   4.177  1.00 15.00      A    C
ATOM   3940  CD2 LEU A 503       8.527  -8.652   1.884  1.00 15.00      A    C
ATOM   3941  C   LEU A 503       7.398 -10.694   5.560  1.00 15.00      A    C
ATOM   3942  O   LEU A 503       6.679 -11.596   5.131  1.00 15.00      A    O
ATOM   3943  N   PHE A 504       6.956  -9.692   6.315  1.00 15.00      A    N
ATOM   3944  CA  PHE A 504       5.562  -9.560   6.749  1.00 15.00      A    C
ATOM   3945  CB  PHE A 504       5.327  -8.146   7.300  1.00 15.00      A    C
ATOM   3946  CG  PHE A 504       3.912  -7.866   7.723  1.00 15.00      A    C
ATOM   3947  CD1 PHE A 504       3.445  -8.296   8.954  1.00 15.00      A    C
ATOM   3948  CD2 PHE A 504       3.051  -7.168   6.891  1.00 15.00      A    C
ATOM   3949  CE1 PHE A 504       2.146  -8.039   9.347  1.00 15.00      A    C
ATOM   3950  CE2 PHE A 504       1.751  -6.907   7.277  1.00 15.00      A    C
ATOM   3951  CZ  PHE A 504       1.297  -7.343   8.508  1.00 15.00      A    C
ATOM   3952  C   PHE A 504       4.542  -9.864   5.652  1.00 15.00      A    C
ATOM   3953  O   PHE A 504       3.675 -10.717   5.822  1.00 15.00      A    O
ATOM   3954  N   HIS A 505       4.666  -9.175   4.526  1.00 15.00      A    N
ATOM   3955  CA  HIS A 505       3.732  -9.325   3.408  1.00 15.00      A    C
ATOM   3956  CB  HIS A 505       4.046  -8.306   2.311  1.00 15.00      A    C
ATOM   3957  CG  HIS A 505       3.965  -6.884   2.765  1.00 15.00      A    C
ATOM   3958  ND1 HIS A 505       5.045  -6.162   3.224  1.00 15.00      A    N
ATOM   3959  CD2 HIS A 505       2.902  -6.046   2.828  1.00 15.00      A    C
ATOM   3960  CE1 HIS A 505       4.613  -4.938   3.549  1.00 15.00      A    C
ATOM   3961  NE2 HIS A 505       3.320  -4.815   3.325  1.00 15.00      A    N
ATOM   3962  C   HIS A 505       3.707 -10.729   2.811  1.00 15.00      A    C
ATOM   3963  O   HIS A 505       2.753 -11.098   2.127  1.00 15.00      A    O
ATOM   3964  N   VAL A 506       4.735 -11.514   3.071  1.00 15.00      A    N
ATOM   3965  CA  VAL A 506       4.806 -12.854   2.516  1.00 15.00      A    C
ATOM   3966  CB  VAL A 506       6.239 -13.212   2.078  1.00 15.00      A    C
ATOM   3967  CG1 VAL A 506       6.258 -14.530   1.320  1.00 15.00      A    C
ATOM   3968  CG2 VAL A 506       6.815 -12.099   1.215  1.00 15.00      A    C
ATOM   3969  C   VAL A 506       4.255 -13.907   3.476  1.00 15.00      A    C
```

ATOM	3970	O	VAL	A	506	3.490	-14.779	3.069	1.00	15.00	A	O
ATOM	3971	N	SER	A	507	4.618	-13.808	4.748	1.00	15.00	A	N
ATOM	3972	CA	SER	A	507	4.160	-14.771	5.744	1.00	15.00	A	C
ATOM	3973	CB	SER	A	507	5.148	-14.834	6.909	1.00	15.00	A	C
ATOM	3974	OG	SER	A	507	5.934	-13.654	6.968	1.00	15.00	A	O
ATOM	3975	C	SER	A	507	2.756	-14.457	6.256	1.00	15.00	A	C
ATOM	3976	O	SER	A	507	2.117	-15.296	6.890	1.00	15.00	A	O
ATOM	3977	N	ASN	A	508	2.271	-13.253	5.978	1.00	15.00	A	N
ATOM	3978	CA	ASN	A	508	0.943	-12.855	6.434	1.00	15.00	A	C
ATOM	3979	CB	ASN	A	508	0.976	-11.502	7.149	1.00	15.00	A	C
ATOM	3980	CG	ASN	A	508	1.581	-11.598	8.538	1.00	15.00	A	C
ATOM	3981	OD1	ASN	A	508	0.870	-11.744	9.531	1.00	15.00	A	O
ATOM	3982	ND2	ASN	A	508	2.902	-11.524	8.618	1.00	15.00	A	N
ATOM	3983	C	ASN	A	508	-0.086	-12.870	5.309	1.00	15.00	A	C
ATOM	3984	O	ASN	A	508	-1.203	-12.387	5.480	1.00	15.00	A	O
ATOM	3985	N	ASP	A	509	0.310	-13.423	4.161	1.00	15.00	A	N
ATOM	3986	CA	ASP	A	509	-0.575	-13.550	2.995	1.00	15.00	A	C
ATOM	3987	CB	ASP	A	509	-1.740	-14.499	3.315	1.00	15.00	A	C
ATOM	3988	CG	ASP	A	509	-2.594	-14.853	2.114	1.00	15.00	A	C
ATOM	3989	OD1	ASP	A	509	-2.034	-15.264	1.074	1.00	15.00	A	O
ATOM	3990	OD2	ASP	A	509	-3.836	-14.769	2.220	1.00	15.00	A	O
ATOM	3991	C	ASP	A	509	-1.083	-12.201	2.467	1.00	15.00	A	C
ATOM	3992	O	ASP	A	509	-2.248	-11.846	2.640	1.00	15.00	A	O
ATOM	3993	N	TYR	A	510	-0.197	-11.450	1.823	1.00	15.00	A	N
ATOM	3994	CA	TYR	A	510	-0.553	-10.152	1.258	1.00	15.00	A	C
ATOM	3995	CB	TYR	A	510	-0.132	-9.016	2.199	1.00	15.00	A	C
ATOM	3996	CG	TYR	A	510	-1.060	-8.776	3.367	1.00	15.00	A	C
ATOM	3997	CD1	TYR	A	510	-2.298	-8.171	3.185	1.00	15.00	A	C
ATOM	3998	CD2	TYR	A	510	-0.692	-9.141	4.654	1.00	15.00	A	C
ATOM	3999	CE1	TYR	A	510	-3.141	-7.937	4.255	1.00	15.00	A	C
ATOM	4000	CE2	TYR	A	510	-1.529	-8.914	5.728	1.00	15.00	A	C
ATOM	4001	CZ	TYR	A	510	-2.751	-8.311	5.524	1.00	15.00	A	C
ATOM	4002	OH	TYR	A	510	-3.585	-8.079	6.594	1.00	15.00	A	O
ATOM	4003	C	TYR	A	510	0.134	-9.950	-0.086	1.00	15.00	A	C
ATOM	4004	O	TYR	A	510	1.231	-10.469	-0.313	1.00	15.00	A	O
ATOM	4005	N	SER	A	511	-0.509	-9.206	-0.975	1.00	15.00	A	N
ATOM	4006	CA	SER	A	511	0.062	-8.914	-2.280	1.00	15.00	A	C
ATOM	4007	CB	SER	A	511	-1.038	-8.453	-3.238	1.00	15.00	A	C
ATOM	4008	OG	SER	A	511	-2.032	-7.716	-2.542	1.00	15.00	A	O
ATOM	4009	C	SER	A	511	1.131	-7.836	-2.132	1.00	15.00	A	C
ATOM	4010	O	SER	A	511	1.011	-6.943	-1.288	1.00	15.00	A	O
ATOM	4011	N	PHE	A	512	2.172	-7.920	-2.937	1.00	15.00	A	N
ATOM	4012	CA	PHE	A	512	3.257	-6.959	-2.868	1.00	15.00	A	C
ATOM	4013	CB	PHE	A	512	4.594	-7.684	-2.693	1.00	15.00	A	C
ATOM	4014	CG	PHE	A	512	5.627	-6.886	-1.952	1.00	15.00	A	C
ATOM	4015	CD1	PHE	A	512	5.439	-6.547	-0.622	1.00	15.00	A	C
ATOM	4016	CD2	PHE	A	512	6.787	-6.476	-2.584	1.00	15.00	A	C
ATOM	4017	CE1	PHE	A	512	6.389	-5.814	0.063	1.00	15.00	A	C
ATOM	4018	CE2	PHE	A	512	7.741	-5.743	-1.906	1.00	15.00	A	C
ATOM	4019	CZ	PHE	A	512	7.542	-5.411	-0.580	1.00	15.00	A	C
ATOM	4020	C	PHE	A	512	3.285	-6.078	-4.110	1.00	15.00	A	C
ATOM	4021	O	PHE	A	512	3.753	-4.940	-4.062	1.00	15.00	A	O
ATOM	4022	N	ILE	A	513	2.753	-6.604	-5.211	1.00	15.00	A	N
ATOM	4023	CA	ILE	A	513	2.713	-5.891	-6.489	1.00	15.00	A	C
ATOM	4024	CB	ILE	A	513	2.006	-6.728	-7.584	1.00	15.00	A	C
ATOM	4025	CG1	ILE	A	513	2.240	-6.133	-8.976	1.00	15.00	A	C
ATOM	4026	CG2	ILE	A	513	0.516	-6.877	-7.300	1.00	15.00	A	C
ATOM	4027	CD1	ILE	A	513	3.685	-6.178	-9.425	1.00	15.00	A	C
ATOM	4028	C	ILE	A	513	2.064	-4.506	-6.375	1.00	15.00	A	C
ATOM	4029	O	ILE	A	513	2.402	-3.592	-7.129	1.00	15.00	A	O
ATOM	4030	N	ARG	A	514	1.161	-4.353	-5.409	1.00	15.00	A	N
ATOM	4031	CA	ARG	A	514	0.454	-3.091	-5.179	1.00	15.00	A	C
ATOM	4032	CB	ARG	A	514	-0.500	-3.228	-3.991	1.00	15.00	A	C
ATOM	4033	CG	ARG	A	514	0.192	-3.275	-2.639	1.00	15.00	A	C
ATOM	4034	CD	ARG	A	514	-0.587	-4.117	-1.648	1.00	15.00	A	C
ATOM	4035	NE	ARG	A	514	-1.975	-3.680	-1.518	1.00	15.00	A	N
ATOM	4036	CZ	ARG	A	514	-2.932	-4.428	-0.976	1.00	15.00	A	C
ATOM	4037	NH1	ARG	A	514	-2.646	-5.644	-0.525	1.00	15.00	A	N
ATOM	4038	NH2	ARG	A	514	-4.171	-3.959	-0.884	1.00	15.00	A	N
ATOM	4039	C	ARG	A	514	1.410	-1.928	-4.929	1.00	15.00	A	C
ATOM	4040	O	ARG	A	514	1.051	-0.768	-5.119	1.00	15.00	A	O

ATOM	4041	N	TYR	A	515	2.627	-2.239	-4.499	1.00	15.00	A	N
ATOM	4042	CA	TYR	A	515	3.613	-1.211	-4.221	1.00	15.00	A	C
ATOM	4043	CB	TYR	A	515	4.634	-1.701	-3.192	1.00	15.00	A	C
ATOM	4044	CG	TYR	A	515	4.025	-1.953	-1.829	1.00	15.00	A	C
ATOM	4045	CD1	TYR	A	515	3.664	-0.894	-1.007	1.00	15.00	A	C
ATOM	4046	CD2	TYR	A	515	3.800	-3.245	-1.367	1.00	15.00	A	C
ATOM	4047	CE1	TYR	A	515	3.098	-1.111	0.234	1.00	15.00	A	C
ATOM	4048	CE2	TYR	A	515	3.235	-3.473	-0.125	1.00	15.00	A	C
ATOM	4049	CZ	TYR	A	515	2.885	-2.401	0.671	1.00	15.00	A	C
ATOM	4050	OH	TYR	A	515	2.316	-2.614	1.903	1.00	15.00	A	O
ATOM	4051	C	TYR	A	515	4.286	-0.740	-5.503	1.00	15.00	A	C
ATOM	4052	O	TYR	A	515	4.841	0.354	-5.560	1.00	15.00	A	O
ATOM	4053	N	TYR	A	516	4.220	-1.566	-6.537	1.00	15.00	A	N
ATOM	4054	CA	TYR	A	516	4.805	-1.217	-7.822	1.00	15.00	A	C
ATOM	4055	CB	TYR	A	516	5.163	-2.482	-8.615	1.00	15.00	A	C
ATOM	4056	CG	TYR	A	516	5.776	-2.211	-9.974	1.00	15.00	A	C
ATOM	4057	CD1	TYR	A	516	5.013	-2.303	-11.132	1.00	15.00	A	C
ATOM	4058	CD2	TYR	A	516	7.115	-1.858	-10.101	1.00	15.00	A	C
ATOM	4059	CE1	TYR	A	516	5.563	-2.052	-12.374	1.00	15.00	A	C
ATOM	4060	CE2	TYR	A	516	7.674	-1.605	-11.341	1.00	15.00	A	C
ATOM	4061	CZ	TYR	A	516	6.892	-1.704	-12.474	1.00	15.00	A	C
ATOM	4062	OH	TYR	A	516	7.440	-1.448	-13.710	1.00	15.00	A	O
ATOM	4063	C	TYR	A	516	3.818	-0.360	-8.608	1.00	15.00	A	C
ATOM	4064	O	TYR	A	516	4.189	0.647	-9.211	1.00	15.00	A	O
ATOM	4065	N	THR	A	517	2.557	-0.759	-8.571	1.00	15.00	A	N
ATOM	4066	CA	THR	A	517	1.499	-0.048	-9.269	1.00	15.00	A	C
ATOM	4067	CB	THR	A	517	0.203	-0.874	-9.232	1.00	15.00	A	C
ATOM	4068	OG1	THR	A	517	0.244	-1.773	-8.112	1.00	15.00	A	O
ATOM	4069	CG2	THR	A	517	0.061	-1.678	-10.512	1.00	15.00	A	C
ATOM	4070	C	THR	A	517	1.250	1.325	-8.644	1.00	15.00	A	C
ATOM	4071	O	THR	A	517	1.186	2.340	-9.345	1.00	15.00	A	O
ATOM	4072	N	ARG	A	518	1.139	1.334	-7.318	1.00	15.00	A	N
ATOM	4073	CA	ARG	A	518	0.895	2.553	-6.547	1.00	15.00	A	C
ATOM	4074	CB	ARG	A	518	0.883	2.240	-5.048	1.00	15.00	A	C
ATOM	4075	CG	ARG	A	518	0.787	3.458	-4.150	1.00	15.00	A	C
ATOM	4076	CD	ARG	A	518	1.881	3.433	-3.099	1.00	15.00	A	C
ATOM	4077	NE	ARG	A	518	3.064	4.180	-3.510	1.00	15.00	A	N
ATOM	4078	CZ	ARG	A	518	4.042	4.528	-2.685	1.00	15.00	A	C
ATOM	4079	NH1	ARG	A	518	3.995	4.203	-1.397	1.00	15.00	A	N
ATOM	4080	NH2	ARG	A	518	5.075	5.195	-3.152	1.00	15.00	A	N
ATOM	4081	C	ARG	A	518	1.934	3.628	-6.847	1.00	15.00	A	C
ATOM	4082	O	ARG	A	518	1.584	4.788	-7.065	1.00	15.00	A	O
ATOM	4083	N	THR	A	519	3.207	3.239	-6.844	1.00	15.00	A	N
ATOM	4084	CA	THR	A	519	4.298	4.167	-7.115	1.00	15.00	A	C
ATOM	4085	CB	THR	A	519	5.658	3.445	-7.043	1.00	15.00	A	C
ATOM	4086	OG1	THR	A	519	5.802	2.841	-5.754	1.00	15.00	A	O
ATOM	4087	CG2	THR	A	519	6.802	4.419	-7.264	1.00	15.00	A	C
ATOM	4088	C	THR	A	519	4.139	4.856	-8.475	1.00	15.00	A	C
ATOM	4089	O	THR	A	519	4.290	6.074	-8.586	1.00	15.00	A	O
ATOM	4090	N	LEU	A	520	3.818	4.078	-9.501	1.00	15.00	A	N
ATOM	4091	CA	LEU	A	520	3.646	4.623	-10.842	1.00	15.00	A	C
ATOM	4092	CB	LEU	A	520	3.635	3.507	-11.889	1.00	15.00	A	C
ATOM	4093	CG	LEU	A	520	5.001	3.068	-12.439	1.00	15.00	A	C
ATOM	4094	CD1	LEU	A	520	5.962	2.697	-11.319	1.00	15.00	A	C
ATOM	4095	CD2	LEU	A	520	4.838	1.902	-13.400	1.00	15.00	A	C
ATOM	4096	C	LEU	A	520	2.387	5.481	-10.939	1.00	15.00	A	C
ATOM	4097	O	LEU	A	520	2.398	6.542	-11.568	1.00	15.00	A	O
ATOM	4098	N	TYR	A	521	1.310	5.026	-10.302	1.00	15.00	A	N
ATOM	4099	CA	TYR	A	521	0.047	5.760	-10.308	1.00	15.00	A	C
ATOM	4100	CB	TYR	A	521	-1.037	4.995	-9.537	1.00	15.00	A	C
ATOM	4101	CG	TYR	A	521	-1.511	3.713	-10.183	1.00	15.00	A	C
ATOM	4102	CD1	TYR	A	521	-1.529	3.564	-11.565	1.00	15.00	A	C
ATOM	4103	CD2	TYR	A	521	-1.949	2.649	-9.404	1.00	15.00	A	C
ATOM	4104	CE1	TYR	A	521	-1.968	2.391	-12.150	1.00	15.00	A	C
ATOM	4105	CE2	TYR	A	521	-2.387	1.475	-9.980	1.00	15.00	A	C
ATOM	4106	CZ	TYR	A	521	-2.395	1.351	-11.352	1.00	15.00	A	C
ATOM	4107	OH	TYR	A	521	-2.834	0.180	-11.926	1.00	15.00	A	O
ATOM	4108	C	TYR	A	521	0.221	7.136	-9.677	1.00	15.00	A	C
ATOM	4109	O	TYR	A	521	-0.257	8.140	-10.207	1.00	15.00	A	O
ATOM	4110	N	GLN	A	522	0.922	7.167	-8.549	1.00	15.00	A	N
ATOM	4111	CA	GLN	A	522	1.161	8.404	-7.813	1.00	15.00	A	C

ATOM	4112	CB	GLN	A	522	1.925	8.114	-6.509	1.00	15.00	A	C
ATOM	4113	CG	GLN	A	522	3.392	8.527	-6.505	1.00	15.00	A	C
ATOM	4114	CD	GLN	A	522	4.196	7.828	-5.431	1.00	15.00	A	C
ATOM	4115	OE1	GLN	A	522	3.980	6.651	-5.145	1.00	15.00	A	O
ATOM	4116	NE2	GLN	A	522	5.127	8.551	-4.833	1.00	15.00	A	N
ATOM	4117	C	GLN	A	522	1.884	9.446	-8.665	1.00	15.00	A	C
ATOM	4118	O	GLN	A	522	1.516	10.622	-8.668	1.00	15.00	A	O
ATOM	4119	N	PHE	A	523	2.894	9.010	-9.409	1.00	15.00	A	N
ATOM	4120	CA	PHE	A	523	3.655	9.921	-10.248	1.00	15.00	A	C
ATOM	4121	CB	PHE	A	523	4.973	9.297	-10.701	1.00	15.00	A	C
ATOM	4122	CG	PHE	A	523	6.004	9.256	-9.609	1.00	15.00	A	C
ATOM	4123	CD1	PHE	A	523	6.446	10.427	-9.015	1.00	15.00	A	C
ATOM	4124	CD2	PHE	A	523	6.522	8.053	-9.168	1.00	15.00	A	C
ATOM	4125	CE1	PHE	A	523	7.387	10.398	-8.004	1.00	15.00	A	C
ATOM	4126	CE2	PHE	A	523	7.464	8.016	-8.159	1.00	15.00	A	C
ATOM	4127	CZ	PHE	A	523	7.896	9.190	-7.575	1.00	15.00	A	C
ATOM	4128	C	PHE	A	523	2.817	10.394	-11.421	1.00	15.00	A	C
ATOM	4129	O	PHE	A	523	2.944	11.532	-11.871	1.00	15.00	A	O
ATOM	4130	N	GLN	A	524	1.944	9.515	-11.897	1.00	15.00	A	N
ATOM	4131	CA	GLN	A	524	1.055	9.846	-12.996	1.00	15.00	A	C
ATOM	4132	CB	GLN	A	524	0.303	8.599	-13.474	1.00	15.00	A	C
ATOM	4133	CG	GLN	A	524	1.087	7.719	-14.435	1.00	15.00	A	C
ATOM	4134	CD	GLN	A	524	0.308	6.492	-14.872	1.00	15.00	A	C
ATOM	4135	OE1	GLN	A	524	0.434	5.418	-14.287	1.00	15.00	A	O
ATOM	4136	NE2	GLN	A	524	-0.505	6.643	-15.908	1.00	15.00	A	N
ATOM	4137	C	GLN	A	524	0.065	10.911	-12.540	1.00	15.00	A	C
ATOM	4138	O	GLN	A	524	-0.171	11.896	-13.240	1.00	15.00	A	O
ATOM	4139	N	PHE	A	525	-0.497	10.708	-11.348	1.00	15.00	A	N
ATOM	4140	CA	PHE	A	525	-1.460	11.643	-10.777	1.00	15.00	A	C
ATOM	4141	CB	PHE	A	525	-1.973	11.145	-9.421	1.00	15.00	A	C
ATOM	4142	CG	PHE	A	525	-2.888	9.957	-9.493	1.00	15.00	A	C
ATOM	4143	CD1	PHE	A	525	-3.820	9.839	-10.511	1.00	15.00	A	C
ATOM	4144	CD2	PHE	A	525	-2.819	8.959	-8.536	1.00	15.00	A	C
ATOM	4145	CE1	PHE	A	525	-4.664	8.748	-10.573	1.00	15.00	A	C
ATOM	4146	CE2	PHE	A	525	-3.660	7.865	-8.591	1.00	15.00	A	C
ATOM	4147	CZ	PHE	A	525	-4.583	7.759	-9.611	1.00	15.00	A	C
ATOM	4148	C	PHE	A	525	-0.845	13.023	-10.602	1.00	15.00	A	C
ATOM	4149	O	PHE	A	525	-1.384	14.016	-11.090	1.00	15.00	A	O
ATOM	4150	N	GLN	A	526	0.290	13.070	-9.910	1.00	15.00	A	N
ATOM	4151	CA	GLN	A	526	0.982	14.327	-9.650	1.00	15.00	A	C
ATOM	4152	CB	GLN	A	526	2.279	14.085	-8.869	1.00	15.00	A	C
ATOM	4153	CG	GLN	A	526	2.952	15.353	-8.356	1.00	15.00	A	C
ATOM	4154	CD	GLN	A	526	2.144	16.077	-7.291	1.00	15.00	A	C
ATOM	4155	OE1	GLN	A	526	0.917	16.023	-7.271	1.00	15.00	A	O
ATOM	4156	NE2	GLN	A	526	2.834	16.765	-6.394	1.00	15.00	A	N
ATOM	4157	C	GLN	A	526	1.259	15.101	-10.937	1.00	15.00	A	C
ATOM	4158	O	GLN	A	526	0.946	16.285	-11.029	1.00	15.00	A	O
ATOM	4159	N	GLU	A	527	1.812	14.422	-11.936	1.00	15.00	A	N
ATOM	4160	CA	GLU	A	527	2.128	15.062	-13.209	1.00	15.00	A	C
ATOM	4161	CB	GLU	A	527	2.799	14.073	-14.163	1.00	15.00	A	C
ATOM	4162	CG	GLU	A	527	3.472	14.728	-15.359	1.00	15.00	A	C
ATOM	4163	CD	GLU	A	527	3.998	13.718	-16.354	1.00	15.00	A	C
ATOM	4164	OE1	GLU	A	527	3.181	13.034	-17.000	1.00	15.00	A	O
ATOM	4165	OE2	GLU	A	527	5.236	13.604	-16.501	1.00	15.00	A	O
ATOM	4166	C	GLU	A	527	0.883	15.668	-13.858	1.00	15.00	A	C
ATOM	4167	O	GLU	A	527	0.897	16.824	-14.292	1.00	15.00	A	O
ATOM	4168	N	ALA	A	528	-0.198	14.895	-13.892	1.00	15.00	A	N
ATOM	4169	CA	ALA	A	528	-1.449	15.350	-14.488	1.00	15.00	A	C
ATOM	4170	CB	ALA	A	528	-2.468	14.223	-14.511	1.00	15.00	A	C
ATOM	4171	C	ALA	A	528	-2.009	16.561	-13.749	1.00	15.00	A	C
ATOM	4172	O	ALA	A	528	-2.530	17.494	-14.366	1.00	15.00	A	O
ATOM	4173	N	LEU	A	529	-1.898	16.545	-12.428	1.00	15.00	A	N
ATOM	4174	CA	LEU	A	529	-2.388	17.646	-11.613	1.00	15.00	A	C
ATOM	4175	CB	LEU	A	529	-2.454	17.247	-10.137	1.00	15.00	A	C
ATOM	4176	CG	LEU	A	529	-3.313	16.023	-9.805	1.00	15.00	A	C
ATOM	4177	CD1	LEU	A	529	-3.283	15.733	-8.313	1.00	15.00	A	C
ATOM	4178	CD2	LEU	A	529	-4.743	16.211	-10.293	1.00	15.00	A	C
ATOM	4179	C	LEU	A	529	-1.518	18.884	-11.801	1.00	15.00	A	C
ATOM	4180	O	LEU	A	529	-2.030	19.995	-11.927	1.00	15.00	A	O
ATOM	4181	N	CYS	A	530	-0.203	18.681	-11.838	1.00	15.00	A	N
ATOM	4182	CA	CYS	A	530	0.743	19.777	-12.025	1.00	15.00	A	C

ATOM	4183	C	CYS	A	530	0.506	20.488	-13.354	1.00	15.00	A	C
ATOM	4184	O	CYS	A	530	0.632	21.709	-13.446	1.00	15.00	A	O
ATOM	4185	CB	CYS	A	530	2.183	19.270	-11.949	1.00	15.00	A	C
ATOM	4186	SG	CYS	A	530	2.648	18.583	-10.329	1.00	15.00	A	S
ATOM	4187	N	GLN	A	531	0.160	19.720	-14.384	1.00	15.00	A	N
ATOM	4188	CA	GLN	A	531	-0.112	20.297	-15.694	1.00	15.00	A	C
ATOM	4189	CB	GLN	A	531	-0.306	19.197	-16.746	1.00	15.00	A	C
ATOM	4190	CG	GLN	A	531	-0.367	19.707	-18.180	1.00	15.00	A	C
ATOM	4191	CD	GLN	A	531	-0.388	18.584	-19.201	1.00	15.00	A	C
ATOM	4192	OE1	GLN	A	531	0.654	18.135	-19.670	1.00	15.00	A	O
ATOM	4193	NE2	GLN	A	531	-1.577	18.128	-19.559	1.00	15.00	A	N
ATOM	4194	C	GLN	A	531	-1.343	21.198	-15.622	1.00	15.00	A	C
ATOM	4195	O	GLN	A	531	-1.383	22.267	-16.231	1.00	15.00	A	O
ATOM	4196	N	ALA	A	532	-2.336	20.767	-14.849	1.00	15.00	A	N
ATOM	4197	CA	ALA	A	532	-3.570	21.526	-14.687	1.00	15.00	A	C
ATOM	4198	CB	ALA	A	532	-4.665	20.641	-14.109	1.00	15.00	A	C
ATOM	4199	C	ALA	A	532	-3.357	22.759	-13.810	1.00	15.00	A	C
ATOM	4200	O	ALA	A	532	-3.867	23.839	-14.112	1.00	15.00	A	O
ATOM	4201	N	ALA	A	533	-2.577	22.592	-12.742	1.00	15.00	A	N
ATOM	4202	CA	ALA	A	533	-2.289	23.677	-11.803	1.00	15.00	A	C
ATOM	4203	CB	ALA	A	533	-1.700	23.116	-10.517	1.00	15.00	A	C
ATOM	4204	C	ALA	A	533	-1.344	24.712	-12.405	1.00	15.00	A	C
ATOM	4205	O	ALA	A	533	-1.079	25.747	-11.794	1.00	15.00	A	O
ATOM	4206	N	LYS	A	534	-0.836	24.412	-13.599	1.00	15.00	A	N
ATOM	4207	CA	LYS	A	534	0.076	25.303	-14.311	1.00	15.00	A	C
ATOM	4208	CB	LYS	A	534	-0.574	26.663	-14.586	1.00	15.00	A	C
ATOM	4209	CG	LYS	A	534	-1.935	26.559	-15.253	1.00	15.00	A	C
ATOM	4210	CD	LYS	A	534	-2.757	27.820	-15.052	1.00	15.00	A	C
ATOM	4211	CE	LYS	A	534	-4.227	27.558	-15.338	1.00	15.00	A	C
ATOM	4212	NZ	LYS	A	534	-4.418	26.826	-16.620	1.00	15.00	A	N
ATOM	4213	C	LYS	A	534	1.393	25.458	-13.563	1.00	15.00	A	C
ATOM	4214	O	LYS	A	534	1.744	26.544	-13.098	1.00	15.00	A	O
ATOM	4215	N	HIS	A	535	2.109	24.354	-13.432	1.00	15.00	A	N
ATOM	4216	CA	HIS	A	535	3.385	24.363	-12.749	1.00	15.00	A	C
ATOM	4217	CB	HIS	A	535	3.535	23.144	-11.831	1.00	15.00	A	C
ATOM	4218	CG	HIS	A	535	4.914	23.010	-11.262	1.00	15.00	A	C
ATOM	4219	ND1	HIS	A	535	5.740	21.978	-11.631	1.00	15.00	A	N
ATOM	4220	CD2	HIS	A	535	5.575	23.830	-10.408	1.00	15.00	A	C
ATOM	4221	CE1	HIS	A	535	6.880	22.191	-11.009	1.00	15.00	A	C
ATOM	4222	NE2	HIS	A	535	6.827	23.298	-10.258	1.00	15.00	A	N
ATOM	4223	C	HIS	A	535	4.539	24.428	-13.741	1.00	15.00	A	C
ATOM	4224	O	HIS	A	535	4.741	23.512	-14.539	1.00	15.00	A	O
ATOM	4225	N	GLU	A	536	5.287	25.515	-13.677	1.00	15.00	A	N
ATOM	4226	CA	GLU	A	536	6.433	25.711	-14.546	1.00	15.00	A	C
ATOM	4227	CB	GLU	A	536	6.537	27.184	-14.953	1.00	15.00	A	C
ATOM	4228	CG	GLU	A	536	5.292	27.742	-15.626	1.00	15.00	A	C
ATOM	4229	CD	GLU	A	536	5.070	27.175	-17.014	1.00	15.00	A	C
ATOM	4230	OE1	GLU	A	536	6.069	26.855	-17.693	1.00	15.00	A	O
ATOM	4231	OE2	GLU	A	536	3.897	27.061	-17.428	1.00	15.00	A	O
ATOM	4232	C	GLU	A	536	7.706	25.297	-13.818	1.00	15.00	A	C
ATOM	4233	O	GLU	A	536	8.017	25.823	-12.745	1.00	15.00	A	O
ATOM	4234	N	GLY	A	537	8.424	24.338	-14.380	1.00	15.00	A	N
ATOM	4235	CA	GLY	A	537	9.657	23.890	-13.769	1.00	15.00	A	C
ATOM	4236	C	GLY	A	537	9.719	22.386	-13.592	1.00	15.00	A	C
ATOM	4237	O	GLY	A	537	8.952	21.653	-14.213	1.00	15.00	A	O
ATOM	4238	N	PRO	A	538	10.640	21.899	-12.744	1.00	15.00	A	N
ATOM	4239	CA	PRO	A	538	10.808	20.468	-12.483	1.00	15.00	A	C
ATOM	4240	CB	PRO	A	538	12.143	20.387	-11.728	1.00	15.00	A	C
ATOM	4241	CG	PRO	A	538	12.739	21.755	-11.805	1.00	15.00	A	C
ATOM	4242	CD	PRO	A	538	11.591	22.702	-11.972	1.00	15.00	A	C
ATOM	4243	C	PRO	A	538	9.680	19.916	-11.616	1.00	15.00	A	C
ATOM	4244	O	PRO	A	538	9.295	20.530	-10.618	1.00	15.00	A	O
ATOM	4245	N	LEU	A	539	9.185	18.741	-11.992	1.00	15.00	A	N
ATOM	4246	CA	LEU	A	539	8.084	18.081	-11.292	1.00	15.00	A	C
ATOM	4247	CB	LEU	A	539	7.823	16.702	-11.902	1.00	15.00	A	C
ATOM	4248	CG	LEU	A	539	6.448	16.089	-11.627	1.00	15.00	A	C
ATOM	4249	CD1	LEU	A	539	5.350	16.943	-12.244	1.00	15.00	A	C
ATOM	4250	CD2	LEU	A	539	6.379	14.664	-12.155	1.00	15.00	A	C
ATOM	4251	C	LEU	A	539	8.303	17.954	-9.782	1.00	15.00	A	C
ATOM	4252	O	LEU	A	539	7.347	18.001	-9.009	1.00	15.00	A	O
ATOM	4253	N	HIS	A	540	9.553	17.807	-9.356	1.00	15.00	A	N

```
ATOM   4254  CA  HIS A 540       9.853  17.656  -7.932  1.00 15.00       A    C
ATOM   4255  CB  HIS A 540      11.257  17.088  -7.690  1.00 15.00       A    C
ATOM   4256  CG  HIS A 540      12.382  17.929  -8.207  1.00 15.00       A    C
ATOM   4257  ND1 HIS A 540      13.018  17.628  -9.388  1.00 15.00       A    N
ATOM   4258  CD2 HIS A 540      12.960  19.021  -7.655  1.00 15.00       A    C
ATOM   4259  CE1 HIS A 540      13.965  18.536  -9.527  1.00 15.00       A    C
ATOM   4260  NE2 HIS A 540      13.964  19.395  -8.505  1.00 15.00       A    N
ATOM   4261  C   HIS A 540       9.594  18.928  -7.118  1.00 15.00       A    C
ATOM   4262  O   HIS A 540       9.497  18.877  -5.895  1.00 15.00       A    O
ATOM   4263  N   LYS A 541       9.474  20.064  -7.798  1.00 15.00       A    N
ATOM   4264  CA  LYS A 541       9.211  21.332  -7.122  1.00 15.00       A    C
ATOM   4265  CB  LYS A 541      10.133  22.432  -7.655  1.00 15.00       A    C
ATOM   4266  CG  LYS A 541      11.609  22.209  -7.389  1.00 15.00       A    C
ATOM   4267  CD  LYS A 541      12.444  23.304  -8.031  1.00 15.00       A    C
ATOM   4268  CE  LYS A 541      12.490  24.546  -7.158  1.00 15.00       A    C
ATOM   4269  NZ  LYS A 541      13.485  24.409  -6.068  1.00 15.00       A    N
ATOM   4270  C   LYS A 541       7.758  21.747  -7.331  1.00 15.00       A    C
ATOM   4271  O   LYS A 541       7.391  22.907  -7.120  1.00 15.00       A    O
ATOM   4272  N   CYS A 542       6.940  20.796  -7.757  1.00 15.00       A    N
ATOM   4273  CA  CYS A 542       5.534  21.054  -8.015  1.00 15.00       A    C
ATOM   4274  C   CYS A 542       4.706  21.143  -6.740  1.00 15.00       A    C
ATOM   4275  O   CYS A 542       5.006  20.508  -5.731  1.00 15.00       A    O
ATOM   4276  CB  CYS A 542       4.938  19.999  -8.946  1.00 15.00       A    C
ATOM   4277  SG  CYS A 542       3.154  20.217  -9.249  1.00 15.00       A    S
ATOM   4278  N   ASP A 543       3.642  21.927  -6.819  1.00 15.00       A    N
ATOM   4279  CA  ASP A 543       2.724  22.130  -5.713  1.00 15.00       A    C
ATOM   4280  CB  ASP A 543       3.237  23.232  -4.787  1.00 15.00       A    C
ATOM   4281  CG  ASP A 543       2.197  23.692  -3.794  1.00 15.00       A    C
ATOM   4282  OD1 ASP A 543       1.422  22.844  -3.308  1.00 15.00       A    O
ATOM   4283  OD2 ASP A 543       2.152  24.901  -3.509  1.00 15.00       A    O
ATOM   4284  C   ASP A 543       1.362  22.495  -6.288  1.00 15.00       A    C
ATOM   4285  O   ASP A 543       1.260  23.415  -7.103  1.00 15.00       A    O
ATOM   4286  N   ILE A 544       0.332  21.770  -5.872  1.00 15.00       A    N
ATOM   4287  CA  ILE A 544      -1.019  21.983  -6.382  1.00 15.00       A    C
ATOM   4288  CB  ILE A 544      -1.741  20.641  -6.628  1.00 15.00       A    C
ATOM   4289  CG1 ILE A 544      -1.722  19.780  -5.358  1.00 15.00       A    C
ATOM   4290  CG2 ILE A 544      -1.101  19.900  -7.794  1.00 15.00       A    C
ATOM   4291  CD1 ILE A 544      -2.519  18.498  -5.468  1.00 15.00       A    C
ATOM   4292  C   ILE A 544      -1.878  22.859  -5.473  1.00 15.00       A    C
ATOM   4293  O   ILE A 544      -3.072  23.029  -5.725  1.00 15.00       A    O
ATOM   4294  N   SER A 545      -1.282  23.408  -4.421  1.00 15.00       A    N
ATOM   4295  CA  SER A 545      -2.015  24.268  -3.497  1.00 15.00       A    C
ATOM   4296  CB  SER A 545      -1.102  24.738  -2.363  1.00 15.00       A    C
ATOM   4297  OG  SER A 545      -0.449  23.641  -1.748  1.00 15.00       A    O
ATOM   4298  C   SER A 545      -2.612  25.475  -4.221  1.00 15.00       A    C
ATOM   4299  O   SER A 545      -1.973  26.060  -5.099  1.00 15.00       A    O
ATOM   4300  N   ASN A 546      -3.850  25.817  -3.858  1.00 15.00       A    N
ATOM   4301  CA  ASN A 546      -4.568  26.959  -4.439  1.00 15.00       A    C
ATOM   4302  CB  ASN A 546      -3.749  28.256  -4.379  1.00 15.00       A    C
ATOM   4303  CG  ASN A 546      -3.698  28.865  -2.990  1.00 15.00       A    C
ATOM   4304  OD1 ASN A 546      -4.641  28.750  -2.210  1.00 15.00       A    O
ATOM   4305  ND2 ASN A 546      -2.590  29.520  -2.671  1.00 15.00       A    N
ATOM   4306  C   ASN A 546      -5.082  26.704  -5.857  1.00 15.00       A    C
ATOM   4307  O   ASN A 546      -5.625  27.608  -6.490  1.00 15.00       A    O
ATOM   4308  N   SER A 547      -4.919  25.486  -6.359  1.00 15.00       A    N
ATOM   4309  CA  SER A 547      -5.388  25.161  -7.700  1.00 15.00       A    C
ATOM   4310  CB  SER A 547      -4.324  24.411  -8.502  1.00 15.00       A    C
ATOM   4311  OG  SER A 547      -4.816  24.037  -9.783  1.00 15.00       A    O
ATOM   4312  C   SER A 547      -6.686  24.367  -7.661  1.00 15.00       A    C
ATOM   4313  O   SER A 547      -6.706  23.191  -7.280  1.00 15.00       A    O
ATOM   4314  N   THR A 548      -7.767  25.018  -8.058  1.00 15.00       A    N
ATOM   4315  CA  THR A 548      -9.077  24.394  -8.089  1.00 15.00       A    C
ATOM   4316  CB  THR A 548     -10.163  25.465  -8.280  1.00 15.00       A    C
ATOM   4317  OG1 THR A 548      -9.539  26.704  -8.654  1.00 15.00       A    O
ATOM   4318  CG2 THR A 548     -10.946  25.663  -6.992  1.00 15.00       A    C
ATOM   4319  C   THR A 548      -9.160  23.389  -9.234  1.00 15.00       A    C
ATOM   4320  O   THR A 548      -9.840  22.366  -9.136  1.00 15.00       A    O
ATOM   4321  N   GLU A 549      -8.455  23.697 -10.316  1.00 15.00       A    N
ATOM   4322  CA  GLU A 549      -8.420  22.847 -11.498  1.00 15.00       A    C
ATOM   4323  CB  GLU A 549      -7.541  23.473 -12.590  1.00 15.00       A    C
ATOM   4324  CG  GLU A 549      -7.751  24.967 -12.805  1.00 15.00       A    C
```

```
ATOM   4325  CD  GLU A 549      -7.032  25.810 -11.770  1.00 15.00      A    C
ATOM   4326  OE1 GLU A 549      -5.823  26.056 -11.940  1.00 15.00      A    O
ATOM   4327  OE2 GLU A 549      -7.677  26.208 -10.774  1.00 15.00      A    O
ATOM   4328  C   GLU A 549      -7.891  21.463 -11.144  1.00 15.00      A    C
ATOM   4329  O   GLU A 549      -8.487  20.445 -11.508  1.00 15.00      A    O
ATOM   4330  N   ALA A 550      -6.773  21.437 -10.425  1.00 15.00      A    N
ATOM   4331  CA  ALA A 550      -6.160  20.184 -10.009  1.00 15.00      A    C
ATOM   4332  CB  ALA A 550      -4.812  20.446  -9.355  1.00 15.00      A    C
ATOM   4333  C   ALA A 550      -7.078  19.429  -9.055  1.00 15.00      A    C
ATOM   4334  O   ALA A 550      -7.279  18.221  -9.197  1.00 15.00      A    O
ATOM   4335  N   GLY A 551      -7.645  20.155  -8.095  1.00 15.00      A    N
ATOM   4336  CA  GLY A 551      -8.541  19.549  -7.129  1.00 15.00      A    C
ATOM   4337  C   GLY A 551      -9.752  18.915  -7.784  1.00 15.00      A    C
ATOM   4338  O   GLY A 551     -10.094  17.769  -7.494  1.00 15.00      A    O
ATOM   4339  N   GLN A 552     -10.389  19.658  -8.684  1.00 15.00      A    N
ATOM   4340  CA  GLN A 552     -11.569  19.172  -9.392  1.00 15.00      A    C
ATOM   4341  CB  GLN A 552     -12.169  20.290 -10.254  1.00 15.00      A    C
ATOM   4342  CG  GLN A 552     -13.386  19.880 -11.072  1.00 15.00      A    C
ATOM   4343  CD  GLN A 552     -14.566  19.468 -10.212  1.00 15.00      A    C
ATOM   4344  OE1 GLN A 552     -15.376  20.299  -9.812  1.00 15.00      A    O
ATOM   4345  NE2 GLN A 552     -14.675  18.178  -9.931  1.00 15.00      A    N
ATOM   4346  C   GLN A 552     -11.233  17.959 -10.257  1.00 15.00      A    C
ATOM   4347  O   GLN A 552     -12.003  16.997 -10.321  1.00 15.00      A    O
ATOM   4348  N   LYS A 553     -10.079  18.007 -10.914  1.00 15.00      A    N
ATOM   4349  CA  LYS A 553      -9.641  16.915 -11.774  1.00 15.00      A    C
ATOM   4350  CB  LYS A 553      -8.357  17.296 -12.517  1.00 15.00      A    C
ATOM   4351  CG  LYS A 553      -7.978  16.342 -13.639  1.00 15.00      A    C
ATOM   4352  CD  LYS A 553      -7.698  17.095 -14.930  1.00 15.00      A    C
ATOM   4353  CE  LYS A 553      -7.213  16.160 -16.028  1.00 15.00      A    C
ATOM   4354  NZ  LYS A 553      -8.194  15.079 -16.323  1.00 15.00      A    N
ATOM   4355  C   LYS A 553      -9.436  15.636 -10.968  1.00 15.00      A    C
ATOM   4356  O   LYS A 553      -9.813  14.549 -11.407  1.00 15.00      A    O
ATOM   4357  N   LEU A 554      -8.849  15.776  -9.787  1.00 15.00      A    N
ATOM   4358  CA  LEU A 554      -8.604  14.635  -8.915  1.00 15.00      A    C
ATOM   4359  CB  LEU A 554      -7.614  15.011  -7.808  1.00 15.00      A    C
ATOM   4360  CG  LEU A 554      -7.154  13.872  -6.895  1.00 15.00      A    C
ATOM   4361  CD1 LEU A 554      -6.343  12.848  -7.675  1.00 15.00      A    C
ATOM   4362  CD2 LEU A 554      -6.352  14.415  -5.723  1.00 15.00      A    C
ATOM   4363  C   LEU A 554      -9.912  14.139  -8.306  1.00 15.00      A    C
ATOM   4364  O   LEU A 554     -10.158  12.932  -8.228  1.00 15.00      A    O
ATOM   4365  N   PHE A 555     -10.757  15.083  -7.897  1.00 15.00      A    N
ATOM   4366  CA  PHE A 555     -12.047  14.767  -7.292  1.00 15.00      A    C
ATOM   4367  CB  PHE A 555     -12.771  16.052  -6.873  1.00 15.00      A    C
ATOM   4368  CG  PHE A 555     -14.073  15.821  -6.158  1.00 15.00      A    C
ATOM   4369  CD1 PHE A 555     -14.092  15.308  -4.872  1.00 15.00      A    C
ATOM   4370  CD2 PHE A 555     -15.278  16.117  -6.774  1.00 15.00      A    C
ATOM   4371  CE1 PHE A 555     -15.288  15.093  -4.213  1.00 15.00      A    C
ATOM   4372  CE2 PHE A 555     -16.476  15.905  -6.122  1.00 15.00      A    C
ATOM   4373  CZ  PHE A 555     -16.482  15.392  -4.840  1.00 15.00      A    C
ATOM   4374  C   PHE A 555     -12.921  13.947  -8.238  1.00 15.00      A    C
ATOM   4375  O   PHE A 555     -13.737  13.136  -7.797  1.00 15.00      A    O
ATOM   4376  N   ASN A 556     -12.733  14.150  -9.538  1.00 15.00      A    N
ATOM   4377  CA  ASN A 556     -13.499  13.425 -10.549  1.00 15.00      A    C
ATOM   4378  CB  ASN A 556     -13.151  13.904 -11.959  1.00 15.00      A    C
ATOM   4379  CG  ASN A 556     -13.918  15.150 -12.351  1.00 15.00      A    C
ATOM   4380  OD1 ASN A 556     -15.022  15.392 -11.868  1.00 15.00      A    O
ATOM   4381  ND2 ASN A 556     -13.340  15.947 -13.235  1.00 15.00      A    N
ATOM   4382  C   ASN A 556     -13.288  11.921 -10.434  1.00 15.00      A    C
ATOM   4383  O   ASN A 556     -14.158  11.134 -10.798  1.00 15.00      A    O
ATOM   4384  N   MET A 557     -12.127  11.529  -9.930  1.00 15.00      A    N
ATOM   4385  CA  MET A 557     -11.814  10.120  -9.748  1.00 15.00      A    C
ATOM   4386  CB  MET A 557     -10.322   9.867  -9.994  1.00 15.00      A    C
ATOM   4387  CG  MET A 557      -9.873   8.434  -9.744  1.00 15.00      A    C
ATOM   4388  SD  MET A 557      -9.321   8.147  -8.049  1.00 15.00      A    S
ATOM   4389  CE  MET A 557      -7.809   9.107  -8.017  1.00 15.00      A    C
ATOM   4390  C   MET A 557     -12.198   9.684  -8.341  1.00 15.00      A    C
ATOM   4391  O   MET A 557     -12.733   8.594  -8.136  1.00 15.00      A    O
ATOM   4392  N   LEU A 558     -11.947  10.564  -7.378  1.00 15.00      A    N
ATOM   4393  CA  LEU A 558     -12.245  10.288  -5.978  1.00 15.00      A    C
ATOM   4394  CB  LEU A 558     -11.776  11.445  -5.090  1.00 15.00      A    C
ATOM   4395  CG  LEU A 558     -10.310  11.869  -5.222  1.00 15.00      A    C
```

ATOM	4396	CD1	LEU	A	558	-10.012	13.044	-4.305	1.00	15.00	A	C
ATOM	4397	CD2	LEU	A	558	-9.378	10.706	-4.925	1.00	15.00	A	C
ATOM	4398	C	LEU	A	558	-13.734	10.031	-5.748	1.00	15.00	A	C
ATOM	4399	O	LEU	A	558	-14.106	9.207	-4.917	1.00	15.00	A	O
ATOM	4400	N	ARG	A	559	-14.584	10.725	-6.499	1.00	15.00	A	N
ATOM	4401	CA	ARG	A	559	-16.029	10.573	-6.351	1.00	15.00	A	C
ATOM	4402	CB	ARG	A	559	-16.770	11.806	-6.877	1.00	15.00	A	C
ATOM	4403	CG	ARG	A	559	-16.804	11.921	-8.392	1.00	15.00	A	C
ATOM	4404	CD	ARG	A	559	-17.960	12.796	-8.851	1.00	15.00	A	C
ATOM	4405	NE	ARG	A	559	-19.231	12.380	-8.250	1.00	15.00	A	N
ATOM	4406	CZ	ARG	A	559	-20.210	11.763	-8.916	1.00	15.00	A	C
ATOM	4407	NH1	ARG	A	559	-20.066	11.490	-10.209	1.00	15.00	A	N
ATOM	4408	NH2	ARG	A	559	-21.333	11.421	-8.284	1.00	15.00	A	N
ATOM	4409	C	ARG	A	559	-16.559	9.304	-7.020	1.00	15.00	A	C
ATOM	4410	O	ARG	A	559	-17.761	9.027	-6.978	1.00	15.00	A	O
ATOM	4411	N	LEU	A	560	-15.671	8.548	-7.650	1.00	15.00	A	N
ATOM	4412	CA	LEU	A	560	-16.064	7.314	-8.313	1.00	15.00	A	C
ATOM	4413	CB	LEU	A	560	-15.308	7.138	-9.634	1.00	15.00	A	C
ATOM	4414	CG	LEU	A	560	-15.554	8.196	-10.712	1.00	15.00	A	C
ATOM	4415	CD1	LEU	A	560	-14.731	7.889	-11.954	1.00	15.00	A	C
ATOM	4416	CD2	LEU	A	560	-17.034	8.285	-11.056	1.00	15.00	A	C
ATOM	4417	C	LEU	A	560	-15.803	6.119	-7.410	1.00	15.00	A	C
ATOM	4418	O	LEU	A	560	-16.617	5.198	-7.326	1.00	15.00	A	O
ATOM	4419	N	GLY	A	561	-14.672	6.145	-6.722	1.00	15.00	A	N
ATOM	4420	CA	GLY	A	561	-14.318	5.045	-5.853	1.00	15.00	A	C
ATOM	4421	C	GLY	A	561	-13.929	3.836	-6.672	1.00	15.00	A	C
ATOM	4422	O	GLY	A	561	-12.901	3.848	-7.350	1.00	15.00	A	O
ATOM	4423	N	LYS	A	562	-14.753	2.799	-6.637	1.00	15.00	A	N
ATOM	4424	CA	LYS	A	562	-14.475	1.603	-7.412	1.00	15.00	A	C
ATOM	4425	CB	LYS	A	562	-13.887	0.475	-6.555	1.00	15.00	A	C
ATOM	4426	CG	LYS	A	562	-14.849	-0.183	-5.579	1.00	15.00	A	C
ATOM	4427	CD	LYS	A	562	-14.252	-1.468	-5.022	1.00	15.00	A	C
ATOM	4428	CE	LYS	A	562	-13.954	-2.466	-6.134	1.00	15.00	A	C
ATOM	4429	NZ	LYS	A	562	-12.931	-3.473	-5.734	1.00	15.00	A	N
ATOM	4430	C	LYS	A	562	-15.694	1.143	-8.202	1.00	15.00	A	C
ATOM	4431	O	LYS	A	562	-15.801	-0.026	-8.575	1.00	15.00	A	O
ATOM	4432	N	SER	A	563	-16.617	2.069	-8.449	1.00	15.00	A	N
ATOM	4433	CA	SER	A	563	-17.810	1.756	-9.222	1.00	15.00	A	C
ATOM	4434	CB	SER	A	563	-18.878	2.838	-9.032	1.00	15.00	A	C
ATOM	4435	OG	SER	A	563	-18.308	4.135	-9.069	1.00	15.00	A	O
ATOM	4436	C	SER	A	563	-17.426	1.635	-10.689	1.00	15.00	A	C
ATOM	4437	O	SER	A	563	-17.863	0.727	-11.393	1.00	15.00	A	O
ATOM	4438	N	GLU	A	564	-16.588	2.563	-11.123	1.00	15.00	A	N
ATOM	4439	CA	GLU	A	564	-16.091	2.585	-12.483	1.00	15.00	A	C
ATOM	4440	CB	GLU	A	564	-15.902	4.034	-12.945	1.00	15.00	A	C
ATOM	4441	CG	GLU	A	564	-17.198	4.812	-13.107	1.00	15.00	A	C
ATOM	4442	CD	GLU	A	564	-17.977	4.417	-14.348	1.00	15.00	A	C
ATOM	4443	OE1	GLU	A	564	-17.574	3.447	-15.025	1.00	15.00	A	O
ATOM	4444	OE2	GLU	A	564	-18.988	5.085	-14.646	1.00	15.00	A	O
ATOM	4445	C	GLU	A	564	-14.756	1.852	-12.532	1.00	15.00	A	C
ATOM	4446	O	GLU	A	564	-14.050	1.777	-11.523	1.00	15.00	A	O
ATOM	4447	N	PRO	A	565	-14.392	1.295	-13.695	1.00	15.00	A	N
ATOM	4448	CA	PRO	A	565	-13.130	0.570	-13.859	1.00	15.00	A	C
ATOM	4449	CB	PRO	A	565	-13.131	0.179	-15.340	1.00	15.00	A	C
ATOM	4450	CG	PRO	A	565	-14.561	0.230	-15.749	1.00	15.00	A	C
ATOM	4451	CD	PRO	A	565	-15.185	1.322	-14.934	1.00	15.00	A	C
ATOM	4452	C	PRO	A	565	-11.927	1.461	-13.562	1.00	15.00	A	C
ATOM	4453	O	PRO	A	565	-11.934	2.653	-13.882	1.00	15.00	A	O
ATOM	4454	N	TRP	A	566	-10.892	0.877	-12.960	1.00	15.00	A	N
ATOM	4455	CA	TRP	A	566	-9.680	1.621	-12.624	1.00	15.00	A	C
ATOM	4456	CB	TRP	A	566	-8.656	0.732	-11.900	1.00	15.00	A	C
ATOM	4457	CG	TRP	A	566	-7.987	-0.295	-12.766	1.00	15.00	A	C
ATOM	4458	CD1	TRP	A	566	-8.416	-1.566	-13.014	1.00	15.00	A	C
ATOM	4459	CD2	TRP	A	566	-6.760	-0.138	-13.490	1.00	15.00	A	C
ATOM	4460	NE1	TRP	A	566	-7.536	-2.208	-13.851	1.00	15.00	A	N
ATOM	4461	CE2	TRP	A	566	-6.510	-1.352	-14.157	1.00	15.00	A	C
ATOM	4462	CE3	TRP	A	566	-5.850	0.914	-13.641	1.00	15.00	A	C
ATOM	4463	CZ2	TRP	A	566	-5.390	-1.543	-14.961	1.00	15.00	A	C
ATOM	4464	CZ3	TRP	A	566	-4.740	0.722	-14.440	1.00	15.00	A	C
ATOM	4465	CH2	TRP	A	566	-4.518	-0.497	-15.090	1.00	15.00	A	C
ATOM	4466	C	TRP	A	566	-9.071	2.272	-13.865	1.00	15.00	A	C

ATOM	4467	O	TRP	A	566	-8.424	3.315	-13.778	1.00	15.00	A	O
ATOM	4468	N	THR	A	567	-9.306	1.652	-15.016	1.00	15.00	A	N
ATOM	4469	CA	THR	A	567	-8.811	2.154	-16.285	1.00	15.00	A	C
ATOM	4470	CB	THR	A	567	-9.180	1.172	-17.413	1.00	15.00	A	C
ATOM	4471	OG1	THR	A	567	-10.404	0.506	-17.067	1.00	15.00	A	O
ATOM	4472	CG2	THR	A	567	-8.088	0.127	-17.588	1.00	15.00	A	C
ATOM	4473	C	THR	A	567	-9.414	3.529	-16.582	1.00	15.00	A	C
ATOM	4474	O	THR	A	567	-8.721	4.444	-17.026	1.00	15.00	A	O
ATOM	4475	N	LEU	A	568	-10.709	3.670	-16.309	1.00	15.00	A	N
ATOM	4476	CA	LEU	A	568	-11.406	4.931	-16.529	1.00	15.00	A	C
ATOM	4477	CB	LEU	A	568	-12.922	4.709	-16.580	1.00	15.00	A	C
ATOM	4478	CG	LEU	A	568	-13.794	5.966	-16.697	1.00	15.00	A	C
ATOM	4479	CD1	LEU	A	568	-13.472	6.738	-17.968	1.00	15.00	A	C
ATOM	4480	CD2	LEU	A	568	-15.268	5.601	-16.651	1.00	15.00	A	C
ATOM	4481	C	LEU	A	568	-11.053	5.917	-15.423	1.00	15.00	A	C
ATOM	4482	O	LEU	A	568	-10.891	7.112	-15.667	1.00	15.00	A	O
ATOM	4483	N	ALA	A	569	-10.921	5.400	-14.205	1.00	15.00	A	N
ATOM	4484	CA	ALA	A	569	-10.572	6.225	-13.056	1.00	15.00	A	C
ATOM	4485	CB	ALA	A	569	-10.512	5.376	-11.794	1.00	15.00	A	C
ATOM	4486	C	ALA	A	569	-9.239	6.929	-13.293	1.00	15.00	A	C
ATOM	4487	O	ALA	A	569	-9.054	8.084	-12.909	1.00	15.00	A	O
ATOM	4488	N	LEU	A	570	-8.321	6.225	-13.943	1.00	15.00	A	N
ATOM	4489	CA	LEU	A	570	-7.012	6.774	-14.251	1.00	15.00	A	C
ATOM	4490	CB	LEU	A	570	-6.053	5.650	-14.654	1.00	15.00	A	C
ATOM	4491	CG	LEU	A	570	-4.558	5.959	-14.544	1.00	15.00	A	C
ATOM	4492	CD1	LEU	A	570	-4.188	6.322	-13.115	1.00	15.00	A	C
ATOM	4493	CD2	LEU	A	570	-3.735	4.776	-15.022	1.00	15.00	A	C
ATOM	4494	C	LEU	A	570	-7.121	7.809	-15.368	1.00	15.00	A	C
ATOM	4495	O	LEU	A	570	-6.521	8.885	-15.292	1.00	15.00	A	O
ATOM	4496	N	GLU	A	571	-7.910	7.483	-16.389	1.00	15.00	A	N
ATOM	4497	CA	GLU	A	571	-8.118	8.370	-17.532	1.00	15.00	A	C
ATOM	4498	CB	GLU	A	571	-9.020	7.693	-18.569	1.00	15.00	A	C
ATOM	4499	CG	GLU	A	571	-9.249	8.505	-19.834	1.00	15.00	A	C
ATOM	4500	CD	GLU	A	571	-9.563	7.633	-21.032	1.00	15.00	A	C
ATOM	4501	OE1	GLU	A	571	-8.655	7.408	-21.859	1.00	15.00	A	O
ATOM	4502	OE2	GLU	A	571	-10.714	7.154	-21.145	1.00	15.00	A	O
ATOM	4503	C	GLU	A	571	-8.719	9.702	-17.088	1.00	15.00	A	C
ATOM	4504	O	GLU	A	571	-8.359	10.763	-17.601	1.00	15.00	A	O
ATOM	4505	N	ASN	A	572	-9.611	9.638	-16.109	1.00	15.00	A	N
ATOM	4506	CA	ASN	A	572	-10.262	10.830	-15.576	1.00	15.00	A	C
ATOM	4507	CB	ASN	A	572	-11.233	10.454	-14.455	1.00	15.00	A	C
ATOM	4508	CG	ASN	A	572	-12.680	10.466	-14.906	1.00	15.00	A	C
ATOM	4509	OD1	ASN	A	572	-12.979	10.272	-16.080	1.00	15.00	A	O
ATOM	4510	ND2	ASN	A	572	-13.591	10.692	-13.972	1.00	15.00	A	N
ATOM	4511	C	ASN	A	572	-9.247	11.845	-15.060	1.00	15.00	A	C
ATOM	4512	O	ASN	A	572	-9.480	13.055	-15.115	1.00	15.00	A	O
ATOM	4513	N	VAL	A	573	-8.115	11.359	-14.572	1.00	15.00	A	N
ATOM	4514	CA	VAL	A	573	-7.087	12.239	-14.040	1.00	15.00	A	C
ATOM	4515	CB	VAL	A	573	-6.555	11.758	-12.672	1.00	15.00	A	C
ATOM	4516	CG1	VAL	A	573	-5.510	12.727	-12.130	1.00	15.00	A	C
ATOM	4517	CG2	VAL	A	573	-7.693	11.597	-11.678	1.00	15.00	A	C
ATOM	4518	C	VAL	A	573	-5.912	12.425	-14.998	1.00	15.00	A	C
ATOM	4519	O	VAL	A	573	-5.660	13.533	-15.469	1.00	15.00	A	O
ATOM	4520	N	VAL	A	574	-5.224	11.334	-15.314	1.00	15.00	A	N
ATOM	4521	CA	VAL	A	574	-4.033	11.398	-16.160	1.00	15.00	A	C
ATOM	4522	CB	VAL	A	574	-3.000	10.311	-15.783	1.00	15.00	A	C
ATOM	4523	CG1	VAL	A	574	-2.825	10.255	-14.274	1.00	15.00	A	C
ATOM	4524	CG2	VAL	A	574	-3.406	8.948	-16.320	1.00	15.00	A	C
ATOM	4525	C	VAL	A	574	-4.318	11.363	-17.662	1.00	15.00	A	C
ATOM	4526	O	VAL	A	574	-3.454	11.713	-18.463	1.00	15.00	A	O
ATOM	4527	N	GLY	A	575	-5.515	10.946	-18.046	1.00	15.00	A	N
ATOM	4528	CA	GLY	A	575	-5.851	10.881	-19.459	1.00	15.00	A	C
ATOM	4529	C	GLY	A	575	-5.320	9.622	-20.119	1.00	15.00	A	C
ATOM	4530	O	GLY	A	575	-5.260	9.527	-21.343	1.00	15.00	A	O
ATOM	4531	N	ALA	A	576	-4.923	8.661	-19.300	1.00	15.00	A	N
ATOM	4532	CA	ALA	A	576	-4.403	7.397	-19.788	1.00	15.00	A	C
ATOM	4533	CB	ALA	A	576	-2.898	7.314	-19.572	1.00	15.00	A	C
ATOM	4534	C	ALA	A	576	-5.109	6.254	-19.076	1.00	15.00	A	C
ATOM	4535	O	ALA	A	576	-5.256	6.275	-17.856	1.00	15.00	A	O
ATOM	4536	N	LYS	A	577	-5.546	5.266	-19.842	1.00	15.00	A	N
ATOM	4537	CA	LYS	A	577	-6.259	4.122	-19.288	1.00	15.00	A	C

ATOM	4538	CB	LYS	A	577	-7.129	3.465	-20.363	1.00	15.00	A	C
ATOM	4539	CG	LYS	A	577	-8.106	4.421	-21.022	1.00	15.00	A	C
ATOM	4540	CD	LYS	A	577	-8.907	3.746	-22.123	1.00	15.00	A	C
ATOM	4541	CE	LYS	A	577	-9.722	4.768	-22.900	1.00	15.00	A	C
ATOM	4542	NZ	LYS	A	577	-10.826	4.141	-23.670	1.00	15.00	A	N
ATOM	4543	C	LYS	A	577	-5.326	3.087	-18.668	1.00	15.00	A	C
ATOM	4544	O	LYS	A	577	-5.783	2.153	-18.015	1.00	15.00	A	O
ATOM	4545	N	ASN	A	578	-4.026	3.245	-18.862	1.00	15.00	A	N
ATOM	4546	CA	ASN	A	578	-3.075	2.288	-18.313	1.00	15.00	A	C
ATOM	4547	CB	ASN	A	578	-2.535	1.355	-19.404	1.00	15.00	A	C
ATOM	4548	CG	ASN	A	578	-2.101	0.004	-18.859	1.00	15.00	A	C
ATOM	4549	OD1	ASN	A	578	-2.077	-0.215	-17.647	1.00	15.00	A	O
ATOM	4550	ND2	ASN	A	578	-1.751	-0.909	-19.749	1.00	15.00	A	N
ATOM	4551	C	ASN	A	578	-1.929	2.978	-17.583	1.00	15.00	A	C
ATOM	4552	O	ASN	A	578	-1.713	4.185	-17.733	1.00	15.00	A	O
ATOM	4553	N	MET	A	579	-1.211	2.193	-16.796	1.00	15.00	A	N
ATOM	4554	CA	MET	A	579	-0.076	2.666	-16.020	1.00	15.00	A	C
ATOM	4555	CB	MET	A	579	0.407	1.519	-15.129	1.00	15.00	A	C
ATOM	4556	CG	MET	A	579	1.308	1.929	-13.982	1.00	15.00	A	C
ATOM	4557	SD	MET	A	579	1.624	0.567	-12.842	1.00	15.00	A	S
ATOM	4558	CE	MET	A	579	2.276	-0.676	-13.960	1.00	15.00	A	C
ATOM	4559	C	MET	A	579	1.050	3.119	-16.950	1.00	15.00	A	C
ATOM	4560	O	MET	A	579	1.166	2.633	-18.076	1.00	15.00	A	O
ATOM	4561	N	ASN	A	580	1.874	4.052	-16.489	1.00	15.00	A	N
ATOM	4562	CA	ASN	A	580	2.975	4.558	-17.303	1.00	15.00	A	C
ATOM	4563	CB	ASN	A	580	2.502	5.732	-18.163	1.00	15.00	A	C
ATOM	4564	CG	ASN	A	580	3.403	5.975	-19.354	1.00	15.00	A	C
ATOM	4565	OD1	ASN	A	580	4.355	6.745	-19.277	1.00	15.00	A	O
ATOM	4566	ND2	ASN	A	580	3.109	5.317	-20.463	1.00	15.00	A	N
ATOM	4567	C	ASN	A	580	4.158	4.977	-16.434	1.00	15.00	A	C
ATOM	4568	O	ASN	A	580	3.974	5.516	-15.344	1.00	15.00	A	O
ATOM	4569	N	VAL	A	581	5.371	4.741	-16.927	1.00	15.00	A	N
ATOM	4570	CA	VAL	A	581	6.585	5.074	-16.179	1.00	15.00	A	C
ATOM	4571	CB	VAL	A	581	7.670	3.990	-16.324	1.00	15.00	A	C
ATOM	4572	CG1	VAL	A	581	7.456	2.874	-15.322	1.00	15.00	A	C
ATOM	4573	CG2	VAL	A	581	7.690	3.441	-17.738	1.00	15.00	A	C
ATOM	4574	C	VAL	A	581	7.190	6.421	-16.567	1.00	15.00	A	C
ATOM	4575	O	VAL	A	581	8.073	6.918	-15.869	1.00	15.00	A	O
ATOM	4576	N	ARG	A	582	6.725	7.014	-17.663	1.00	15.00	A	N
ATOM	4577	CA	ARG	A	582	7.262	8.303	-18.107	1.00	15.00	A	C
ATOM	4578	CB	ARG	A	582	6.629	8.798	-19.410	1.00	15.00	A	C
ATOM	4579	CG	ARG	A	582	7.345	9.998	-20.016	1.00	15.00	A	C
ATOM	4580	CD	ARG	A	582	6.479	10.725	-21.037	1.00	15.00	A	C
ATOM	4581	NE	ARG	A	582	5.095	10.896	-20.583	1.00	15.00	A	N
ATOM	4582	CZ	ARG	A	582	4.727	11.633	-19.527	1.00	15.00	A	C
ATOM	4583	NH1	ARG	A	582	5.633	12.279	-18.798	1.00	15.00	A	N
ATOM	4584	NH2	ARG	A	582	3.449	11.715	-19.195	1.00	15.00	A	N
ATOM	4585	C	ARG	A	582	7.226	9.378	-17.008	1.00	15.00	A	C
ATOM	4586	O	ARG	A	582	8.244	10.019	-16.758	1.00	15.00	A	O
ATOM	4587	N	PRO	A	583	6.068	9.603	-16.338	1.00	15.00	A	N
ATOM	4588	CA	PRO	A	583	5.975	10.600	-15.255	1.00	15.00	A	C
ATOM	4589	CB	PRO	A	583	4.538	10.446	-14.746	1.00	15.00	A	C
ATOM	4590	CG	PRO	A	583	3.795	9.864	-15.896	1.00	15.00	A	C
ATOM	4591	CD	PRO	A	583	4.767	8.955	-16.589	1.00	15.00	A	C
ATOM	4592	C	PRO	A	583	6.964	10.297	-14.128	1.00	15.00	A	C
ATOM	4593	O	PRO	A	583	7.506	11.206	-13.498	1.00	15.00	A	O
ATOM	4594	N	LEU	A	584	7.196	9.008	-13.889	1.00	15.00	A	N
ATOM	4595	CA	LEU	A	584	8.129	8.567	-12.857	1.00	15.00	A	C
ATOM	4596	CB	LEU	A	584	8.034	7.044	-12.676	1.00	15.00	A	C
ATOM	4597	CG	LEU	A	584	9.267	6.329	-12.109	1.00	15.00	A	C
ATOM	4598	CD1	LEU	A	584	9.427	6.604	-10.622	1.00	15.00	A	C
ATOM	4599	CD2	LEU	A	584	9.187	4.834	-12.373	1.00	15.00	A	C
ATOM	4600	C	LEU	A	584	9.550	8.975	-13.234	1.00	15.00	A	C
ATOM	4601	O	LEU	A	584	10.285	9.538	-12.422	1.00	15.00	A	O
ATOM	4602	N	LEU	A	585	9.920	8.700	-14.480	1.00	15.00	A	N
ATOM	4603	CA	LEU	A	585	11.244	9.046	-14.980	1.00	15.00	A	C
ATOM	4604	CB	LEU	A	585	11.467	8.440	-16.369	1.00	15.00	A	C
ATOM	4605	CG	LEU	A	585	11.356	6.916	-16.468	1.00	15.00	A	C
ATOM	4606	CD1	LEU	A	585	11.444	6.465	-17.918	1.00	15.00	A	C
ATOM	4607	CD2	LEU	A	585	12.432	6.239	-15.630	1.00	15.00	A	C
ATOM	4608	C	LEU	A	585	11.402	10.561	-15.032	1.00	15.00	A	C

ATOM	4609	O	LEU	A	585	12.463	11.093	-14.725	1.00	15.00	A	O
ATOM	4610	N	ASN	A	586	10.321	11.239	-15.406	1.00	15.00	A	N
ATOM	4611	CA	ASN	A	586	10.297	12.699	-15.496	1.00	15.00	A	C
ATOM	4612	CB	ASN	A	586	8.925	13.157	-16.020	1.00	15.00	A	C
ATOM	4613	CG	ASN	A	586	8.759	14.666	-16.102	1.00	15.00	A	C
ATOM	4614	OD1	ASN	A	586	9.727	15.411	-16.231	1.00	15.00	A	O
ATOM	4615	ND2	ASN	A	586	7.515	15.125	-16.055	1.00	15.00	A	N
ATOM	4616	C	ASN	A	586	10.594	13.326	-14.132	1.00	15.00	A	C
ATOM	4617	O	ASN	A	586	11.290	14.337	-14.034	1.00	15.00	A	O
ATOM	4618	N	TYR	A	587	10.079	12.704	-13.078	1.00	15.00	A	N
ATOM	4619	CA	TYR	A	587	10.292	13.190	-11.719	1.00	15.00	A	C
ATOM	4620	CB	TYR	A	587	9.410	12.406	-10.738	1.00	15.00	A	C
ATOM	4621	CG	TYR	A	587	9.529	12.832	-9.287	1.00	15.00	A	C
ATOM	4622	CD1	TYR	A	587	10.392	12.175	-8.417	1.00	15.00	A	C
ATOM	4623	CD2	TYR	A	587	8.769	13.881	-8.783	1.00	15.00	A	C
ATOM	4624	CE1	TYR	A	587	10.496	12.550	-7.091	1.00	15.00	A	C
ATOM	4625	CE2	TYR	A	587	8.869	14.264	-7.457	1.00	15.00	A	C
ATOM	4626	CZ	TYR	A	587	9.733	13.594	-6.615	1.00	15.00	A	C
ATOM	4627	OH	TYR	A	587	9.834	13.968	-5.293	1.00	15.00	A	O
ATOM	4628	C	TYR	A	587	11.763	13.078	-11.314	1.00	15.00	A	C
ATOM	4629	O	TYR	A	587	12.282	13.928	-10.592	1.00	15.00	A	O
ATOM	4630	N	PHE	A	588	12.435	12.042	-11.801	1.00	15.00	A	N
ATOM	4631	CA	PHE	A	588	13.837	11.815	-11.463	1.00	15.00	A	C
ATOM	4632	CB	PHE	A	588	14.066	10.352	-11.077	1.00	15.00	A	C
ATOM	4633	CG	PHE	A	588	13.431	9.954	-9.778	1.00	15.00	A	C
ATOM	4634	CD1	PHE	A	588	13.955	10.393	-8.573	1.00	15.00	A	C
ATOM	4635	CD2	PHE	A	588	12.311	9.141	-9.761	1.00	15.00	A	C
ATOM	4636	CE1	PHE	A	588	13.374	10.027	-7.375	1.00	15.00	A	C
ATOM	4637	CE2	PHE	A	588	11.724	8.771	-8.566	1.00	15.00	A	C
ATOM	4638	CZ	PHE	A	588	12.256	9.215	-7.371	1.00	15.00	A	C
ATOM	4639	C	PHE	A	588	14.786	12.202	-12.593	1.00	15.00	A	C
ATOM	4640	O	PHE	A	588	15.963	11.841	-12.561	1.00	15.00	A	O
ATOM	4641	N	GLU	A	589	14.285	12.945	-13.572	1.00	15.00	A	N
ATOM	4642	CA	GLU	A	589	15.101	13.369	-14.713	1.00	15.00	A	C
ATOM	4643	CB	GLU	A	589	14.289	14.188	-15.727	1.00	15.00	A	C
ATOM	4644	CG	GLU	A	589	15.073	14.628	-16.960	1.00	15.00	A	C
ATOM	4645	CD	GLU	A	589	15.604	13.465	-17.791	1.00	15.00	A	C
ATOM	4646	OE1	GLU	A	589	15.122	13.277	-18.927	1.00	15.00	A	O
ATOM	4647	OE2	GLU	A	589	16.518	12.749	-17.314	1.00	15.00	A	O
ATOM	4648	C	GLU	A	589	16.394	14.091	-14.292	1.00	15.00	A	C
ATOM	4649	O	GLU	A	589	17.485	13.695	-14.709	1.00	15.00	A	O
ATOM	4650	N	PRO	A	590	16.313	15.148	-13.447	1.00	15.00	A	N
ATOM	4651	CA	PRO	A	590	17.507	15.881	-12.998	1.00	15.00	A	C
ATOM	4652	CB	PRO	A	590	16.946	16.952	-12.056	1.00	15.00	A	C
ATOM	4653	CG	PRO	A	590	15.521	17.098	-12.460	1.00	15.00	A	C
ATOM	4654	CD	PRO	A	590	15.078	15.730	-12.887	1.00	15.00	A	C
ATOM	4655	C	PRO	A	590	18.499	14.986	-12.253	1.00	15.00	A	C
ATOM	4656	O	PRO	A	590	19.690	15.287	-12.184	1.00	15.00	A	O
ATOM	4657	N	LEU	A	591	18.009	13.886	-11.697	1.00	15.00	A	N
ATOM	4658	CA	LEU	A	591	18.868	12.964	-10.973	1.00	15.00	A	C
ATOM	4659	CB	LEU	A	591	18.093	12.240	-9.870	1.00	15.00	A	C
ATOM	4660	CG	LEU	A	591	18.938	11.433	-8.882	1.00	15.00	A	C
ATOM	4661	CD1	LEU	A	591	19.775	12.357	-8.009	1.00	15.00	A	C
ATOM	4662	CD2	LEU	A	591	18.059	10.532	-8.033	1.00	15.00	A	C
ATOM	4663	C	LEU	A	591	19.487	11.956	-11.933	1.00	15.00	A	C
ATOM	4664	O	LEU	A	591	20.648	11.577	-11.788	1.00	15.00	A	O
ATOM	4665	N	PHE	A	592	18.705	11.543	-12.924	1.00	15.00	A	N
ATOM	4666	CA	PHE	A	592	19.163	10.582	-13.918	1.00	15.00	A	C
ATOM	4667	CB	PHE	A	592	18.015	10.194	-14.856	1.00	15.00	A	C
ATOM	4668	CG	PHE	A	592	18.329	9.046	-15.775	1.00	15.00	A	C
ATOM	4669	CD1	PHE	A	592	19.103	7.980	-15.342	1.00	15.00	A	C
ATOM	4670	CD2	PHE	A	592	17.847	9.032	-17.073	1.00	15.00	A	C
ATOM	4671	CE1	PHE	A	592	19.390	6.925	-16.186	1.00	15.00	A	C
ATOM	4672	CE2	PHE	A	592	18.129	7.980	-17.922	1.00	15.00	A	C
ATOM	4673	CZ	PHE	A	592	18.903	6.925	-17.478	1.00	15.00	A	C
ATOM	4674	C	PHE	A	592	20.342	11.143	-14.708	1.00	15.00	A	C
ATOM	4675	O	PHE	A	592	21.266	10.412	-15.071	1.00	15.00	A	O
ATOM	4676	N	THR	A	593	20.307	12.439	-14.981	1.00	15.00	A	N
ATOM	4677	CA	THR	A	593	21.385	13.087	-15.707	1.00	15.00	A	C
ATOM	4678	CB	THR	A	593	20.967	14.487	-16.193	1.00	15.00	A	C
ATOM	4679	OG1	THR	A	593	20.020	15.044	-15.275	1.00	15.00	A	O

ATOM	4680	CG2	THR	A	593	20.331	14.398	-17.573	1.00	15.00	A	C
ATOM	4681	C	THR	A	593	22.637	13.182	-14.831	1.00	15.00	A	C
ATOM	4682	O	THR	A	593	23.754	12.972	-15.305	1.00	15.00	A	O
ATOM	4683	N	TRP	A	594	22.431	13.467	-13.546	1.00	15.00	A	N
ATOM	4684	CA	TRP	A	594	23.529	13.584	-12.589	1.00	15.00	A	C
ATOM	4685	CB	TRP	A	594	23.016	14.158	-11.263	1.00	15.00	A	C
ATOM	4686	CG	TRP	A	594	24.098	14.460	-10.269	1.00	15.00	A	C
ATOM	4687	CD1	TRP	A	594	24.896	15.568	-10.238	1.00	15.00	A	C
ATOM	4688	CD2	TRP	A	594	24.501	13.647	-9.160	1.00	15.00	A	C
ATOM	4689	NE1	TRP	A	594	25.770	15.493	-9.181	1.00	15.00	A	N
ATOM	4690	CE2	TRP	A	594	25.546	14.324	-8.503	1.00	15.00	A	C
ATOM	4691	CE3	TRP	A	594	24.076	12.413	-8.658	1.00	15.00	A	C
ATOM	4692	CZ2	TRP	A	594	26.175	13.808	-7.374	1.00	15.00	A	C
ATOM	4693	CZ3	TRP	A	594	24.702	11.903	-7.537	1.00	15.00	A	C
ATOM	4694	CH2	TRP	A	594	25.738	12.600	-6.907	1.00	15.00	A	C
ATOM	4695	C	TRP	A	594	24.204	12.233	-12.356	1.00	15.00	A	C
ATOM	4696	O	TRP	A	594	25.432	12.136	-12.353	1.00	15.00	A	O
ATOM	4697	N	LEU	A	595	23.397	11.192	-12.158	1.00	15.00	A	N
ATOM	4698	CA	LEU	A	595	23.923	9.847	-11.934	1.00	15.00	A	C
ATOM	4699	CB	LEU	A	595	22.786	8.851	-11.673	1.00	15.00	A	C
ATOM	4700	CG	LEU	A	595	22.021	9.027	-10.356	1.00	15.00	A	C
ATOM	4701	CD1	LEU	A	595	20.828	8.086	-10.296	1.00	15.00	A	C
ATOM	4702	CD2	LEU	A	595	22.938	8.805	-9.162	1.00	15.00	A	C
ATOM	4703	C	LEU	A	595	24.766	9.397	-13.124	1.00	15.00	A	C
ATOM	4704	O	LEU	A	595	25.791	8.732	-12.961	1.00	15.00	A	O
ATOM	4705	N	LYS	A	596	24.331	9.792	-14.319	1.00	15.00	A	N
ATOM	4706	CA	LYS	A	596	25.032	9.452	-15.552	1.00	15.00	A	C
ATOM	4707	CB	LYS	A	596	24.228	9.926	-16.770	1.00	15.00	A	C
ATOM	4708	CG	LYS	A	596	23.608	8.807	-17.600	1.00	15.00	A	C
ATOM	4709	CD	LYS	A	596	22.109	9.012	-17.808	1.00	15.00	A	C
ATOM	4710	CE	LYS	A	596	21.813	10.306	-18.556	1.00	15.00	A	C
ATOM	4711	NZ	LYS	A	596	20.349	10.546	-18.733	1.00	15.00	A	N
ATOM	4712	C	LYS	A	596	26.436	10.058	-15.583	1.00	15.00	A	C
ATOM	4713	O	LYS	A	596	27.364	9.453	-16.114	1.00	15.00	A	O
ATOM	4714	N	ASP	A	597	26.588	11.254	-15.021	1.00	15.00	A	N
ATOM	4715	CA	ASP	A	597	27.892	11.919	-14.993	1.00	15.00	A	C
ATOM	4716	CB	ASP	A	597	27.752	13.430	-14.773	1.00	15.00	A	C
ATOM	4717	CG	ASP	A	597	29.074	14.074	-14.378	1.00	15.00	A	C
ATOM	4718	OD1	ASP	A	597	29.214	14.472	-13.203	1.00	15.00	A	O
ATOM	4719	OD2	ASP	A	597	29.993	14.149	-15.233	1.00	15.00	A	O
ATOM	4720	C	ASP	A	597	28.792	11.324	-13.914	1.00	15.00	A	C
ATOM	4721	O	ASP	A	597	29.997	11.158	-14.111	1.00	15.00	A	O
ATOM	4722	N	GLN	A	598	28.208	10.986	-12.777	1.00	15.00	A	N
ATOM	4723	CA	GLN	A	598	28.972	10.428	-11.670	1.00	15.00	A	C
ATOM	4724	CB	GLN	A	598	28.142	10.406	-10.385	1.00	15.00	A	C
ATOM	4725	CG	GLN	A	598	27.786	11.785	-9.862	1.00	15.00	A	C
ATOM	4726	CD	GLN	A	598	28.991	12.562	-9.364	1.00	15.00	A	C
ATOM	4727	OE1	GLN	A	598	29.898	12.008	-8.743	1.00	15.00	A	O
ATOM	4728	NE2	GLN	A	598	29.014	13.852	-9.653	1.00	15.00	A	N
ATOM	4729	C	GLN	A	598	29.485	9.034	-11.988	1.00	15.00	A	C
ATOM	4730	O	GLN	A	598	30.591	8.666	-11.595	1.00	15.00	A	O
ATOM	4731	N	ASN	A	599	28.703	8.282	-12.745	1.00	15.00	A	N
ATOM	4732	CA	ASN	A	599	29.067	6.914	-13.083	1.00	15.00	A	C
ATOM	4733	CB	ASN	A	599	27.823	6.053	-13.310	1.00	15.00	A	C
ATOM	4734	CG	ASN	A	599	27.123	5.665	-12.020	1.00	15.00	A	C
ATOM	4735	OD1	ASN	A	599	27.760	5.331	-11.021	1.00	15.00	A	O
ATOM	4736	ND2	ASN	A	599	25.804	5.713	-12.035	1.00	15.00	A	N
ATOM	4737	C	ASN	A	599	29.997	6.829	-14.283	1.00	15.00	A	C
ATOM	4738	O	ASN	A	599	30.309	5.735	-14.745	1.00	15.00	A	O
ATOM	4739	N	LYS	A	600	30.455	7.972	-14.789	1.00	15.00	A	N
ATOM	4740	CA	LYS	A	600	31.355	7.967	-15.938	1.00	15.00	A	C
ATOM	4741	CB	LYS	A	600	31.602	9.379	-16.479	1.00	15.00	A	C
ATOM	4742	CG	LYS	A	600	32.467	10.260	-15.594	1.00	15.00	A	C
ATOM	4743	CD	LYS	A	600	32.789	11.574	-16.282	1.00	15.00	A	C
ATOM	4744	CE	LYS	A	600	33.183	12.639	-15.275	1.00	15.00	A	C
ATOM	4745	NZ	LYS	A	600	32.049	12.998	-14.390	1.00	15.00	A	N
ATOM	4746	C	LYS	A	600	32.672	7.269	-15.601	1.00	15.00	A	C
ATOM	4747	O	LYS	A	600	33.317	6.688	-16.469	1.00	15.00	A	O
ATOM	4748	N	ASN	A	601	33.049	7.318	-14.327	1.00	15.00	A	N
ATOM	4749	CA	ASN	A	601	34.284	6.687	-13.866	1.00	15.00	A	C
ATOM	4750	CB	ASN	A	601	35.124	7.654	-13.016	1.00	15.00	A	C

```
ATOM   4751  CG  ASN A 601      35.702   8.823 -13.796  1.00 15.00      A    C
ATOM   4752  OD1 ASN A 601      35.644   8.867 -15.021  1.00 15.00      A    O
ATOM   4753  ND2 ASN A 601      36.281   9.779 -13.083  1.00 15.00      A    N
ATOM   4754  C   ASN A 601      33.971   5.434 -13.053  1.00 15.00      A    C
ATOM   4755  O   ASN A 601      34.747   5.031 -12.186  1.00 15.00      A    O
ATOM   4756  N   SER A 602      32.828   4.827 -13.334  1.00 15.00      A    N
ATOM   4757  CA  SER A 602      32.402   3.627 -12.633  1.00 15.00      A    C
ATOM   4758  CB  SER A 602      31.335   3.980 -11.597  1.00 15.00      A    C
ATOM   4759  OG  SER A 602      31.885   4.775 -10.562  1.00 15.00      A    O
ATOM   4760  C   SER A 602      31.869   2.587 -13.614  1.00 15.00      A    C
ATOM   4761  O   SER A 602      31.628   2.888 -14.782  1.00 15.00      A    O
ATOM   4762  N   PHE A 603      31.698   1.361 -13.137  1.00 15.00      A    N
ATOM   4763  CA  PHE A 603      31.193   0.287 -13.971  1.00 15.00      A    C
ATOM   4764  CB  PHE A 603      31.900  -1.033 -13.642  1.00 15.00      A    C
ATOM   4765  CG  PHE A 603      31.333  -2.216 -14.374  1.00 15.00      A    C
ATOM   4766  CD1 PHE A 603      31.605  -2.410 -15.718  1.00 15.00      A    C
ATOM   4767  CD2 PHE A 603      30.518  -3.125 -13.720  1.00 15.00      A    C
ATOM   4768  CE1 PHE A 603      31.074  -3.489 -16.396  1.00 15.00      A    C
ATOM   4769  CE2 PHE A 603      29.985  -4.207 -14.391  1.00 15.00      A    C
ATOM   4770  CZ  PHE A 603      30.263  -4.389 -15.732  1.00 15.00      A    C
ATOM   4771  C   PHE A 603      29.685   0.136 -13.805  1.00 15.00      A    C
ATOM   4772  O   PHE A 603      29.185  -0.043 -12.691  1.00 15.00      A    O
ATOM   4773  N   VAL A 604      28.968   0.225 -14.913  1.00 15.00      A    N
ATOM   4774  CA  VAL A 604      27.525   0.079 -14.905  1.00 15.00      A    C
ATOM   4775  CB  VAL A 604      26.874   0.895 -16.039  1.00 15.00      A    C
ATOM   4776  CG1 VAL A 604      25.357   0.887 -15.914  1.00 15.00      A    C
ATOM   4777  CG2 VAL A 604      27.405   2.321 -16.032  1.00 15.00      A    C
ATOM   4778  C   VAL A 604      27.176  -1.395 -15.058  1.00 15.00      A    C
ATOM   4779  O   VAL A 604      27.769  -2.097 -15.876  1.00 15.00      A    O
ATOM   4780  N   GLY A 605      26.230  -1.856 -14.261  1.00 15.00      A    N
ATOM   4781  CA  GLY A 605      25.836  -3.247 -14.304  1.00 15.00      A    C
ATOM   4782  C   GLY A 605      26.347  -3.994 -13.088  1.00 15.00      A    C
ATOM   4783  O   GLY A 605      26.924  -3.385 -12.182  1.00 15.00      A    O
ATOM   4784  N   TRP A 606      26.141  -5.303 -13.058  1.00 15.00      A    N
ATOM   4785  CA  TRP A 606      26.585  -6.128 -11.939  1.00 15.00      A    C
ATOM   4786  CB  TRP A 606      25.589  -6.044 -10.768  1.00 15.00      A    C
ATOM   4787  CG  TRP A 606      24.152  -6.217 -11.168  1.00 15.00      A    C
ATOM   4788  CD1 TRP A 606      23.502  -7.393 -11.410  1.00 15.00      A    C
ATOM   4789  CD2 TRP A 606      23.185  -5.177 -11.374  1.00 15.00      A    C
ATOM   4790  NE1 TRP A 606      22.196  -7.150 -11.758  1.00 15.00      A    N
ATOM   4791  CE2 TRP A 606      21.976  -5.799 -11.742  1.00 15.00      A    C
ATOM   4792  CE3 TRP A 606      23.224  -3.783 -11.284  1.00 15.00      A    C
ATOM   4793  CZ2 TRP A 606      20.819  -5.076 -12.020  1.00 15.00      A    C
ATOM   4794  CZ3 TRP A 606      22.074  -3.068 -11.560  1.00 15.00      A    C
ATOM   4795  CH2 TRP A 606      20.888  -3.715 -11.923  1.00 15.00      A    C
ATOM   4796  C   TRP A 606      26.765  -7.579 -12.370  1.00 15.00      A    C
ATOM   4797  O   TRP A 606      26.172  -8.017 -13.356  1.00 15.00      A    O
ATOM   4798  N   SER A 607      27.600  -8.311 -11.649  1.00 15.00      A    N
ATOM   4799  CA  SER A 607      27.833  -9.714 -11.946  1.00 15.00      A    C
ATOM   4800  CB  SER A 607      29.328 -10.034 -11.908  1.00 15.00      A    C
ATOM   4801  OG  SER A 607      29.966  -9.396 -10.815  1.00 15.00      A    O
ATOM   4802  C   SER A 607      27.045 -10.586 -10.970  1.00 15.00      A    C
ATOM   4803  O   SER A 607      26.980 -10.289  -9.775  1.00 15.00      A    O
ATOM   4804  N   THR A 608      26.441 -11.649 -11.480  1.00 15.00      A    N
ATOM   4805  CA  THR A 608      25.625 -12.539 -10.667  1.00 15.00      A    C
ATOM   4806  CB  THR A 608      24.478 -13.111 -11.517  1.00 15.00      A    C
ATOM   4807  OG1 THR A 608      24.968 -13.393 -12.838  1.00 15.00      A    O
ATOM   4808  CG2 THR A 608      23.351 -12.098 -11.625  1.00 15.00      A    C
ATOM   4809  C   THR A 608      26.424 -13.692 -10.057  1.00 15.00      A    C
ATOM   4810  O   THR A 608      25.875 -14.512  -9.315  1.00 15.00      A    O
ATOM   4811  N   ASP A 609      27.713 -13.741 -10.350  1.00 15.00      A    N
ATOM   4812  CA  ASP A 609      28.577 -14.804  -9.849  1.00 15.00      A    C
ATOM   4813  CB  ASP A 609      29.659 -15.152 -10.882  1.00 15.00      A    C
ATOM   4814  CG  ASP A 609      30.673 -14.040 -11.089  1.00 15.00      A    C
ATOM   4815  OD1 ASP A 609      30.257 -12.859 -11.160  1.00 15.00      A    O
ATOM   4816  OD2 ASP A 609      31.879 -14.342 -11.172  1.00 15.00      A    O
ATOM   4817  C   ASP A 609      29.210 -14.441  -8.507  1.00 15.00      A    C
ATOM   4818  O   ASP A 609      29.191 -15.241  -7.564  1.00 15.00      A    O
ATOM   4819  N   TRP A 610      29.753 -13.231  -8.422  1.00 15.00      A    N
ATOM   4820  CA  TRP A 610      30.401 -12.758  -7.207  1.00 15.00      A    C
ATOM   4821  CB  TRP A 610      31.002 -11.360  -7.399  1.00 15.00      A    C
```

ATOM	4822	CG	TRP	A	610	31.754	-10.869	-6.194	1.00	15.00	A	C
ATOM	4823	CD1	TRP	A	610	33.040	-11.172	-5.853	1.00	15.00	A	C
ATOM	4824	CD2	TRP	A	610	31.260	-10.001	-5.163	1.00	15.00	A	C
ATOM	4825	NE1	TRP	A	610	33.378	-10.547	-4.677	1.00	15.00	A	N
ATOM	4826	CE2	TRP	A	610	32.303	-9.823	-4.233	1.00	15.00	A	C
ATOM	4827	CE3	TRP	A	610	30.038	-9.359	-4.936	1.00	15.00	A	C
ATOM	4828	CZ2	TRP	A	610	32.162	-9.029	-3.097	1.00	15.00	A	C
ATOM	4829	CZ3	TRP	A	610	29.901	-8.572	-3.808	1.00	15.00	A	C
ATOM	4830	CH2	TRP	A	610	30.956	-8.414	-2.903	1.00	15.00	A	C
ATOM	4831	C	TRP	A	610	29.459	-12.765	-6.009	1.00	15.00	A	C
ATOM	4832	O	TRP	A	610	28.290	-12.392	-6.111	1.00	15.00	A	O
ATOM	4833	N	SER	A	611	29.993	-13.191	-4.877	1.00	15.00	A	N
ATOM	4834	CA	SER	A	611	29.251	-13.249	-3.636	1.00	15.00	A	C
ATOM	4835	CB	SER	A	611	28.580	-14.617	-3.505	1.00	15.00	A	C
ATOM	4836	OG	SER	A	611	29.467	-15.656	-3.885	1.00	15.00	A	O
ATOM	4837	C	SER	A	611	30.222	-13.026	-2.483	1.00	15.00	A	C
ATOM	4838	O	SER	A	611	31.412	-13.317	-2.620	1.00	15.00	A	O
ATOM	4839	N	PRO	A	612	29.750	-12.500	-1.341	1.00	15.00	A	N
ATOM	4840	CA	PRO	A	612	30.599	-12.249	-0.168	1.00	15.00	A	C
ATOM	4841	CB	PRO	A	612	29.711	-11.389	0.749	1.00	15.00	A	C
ATOM	4842	CG	PRO	A	612	28.543	-10.986	-0.088	1.00	15.00	A	C
ATOM	4843	CD	PRO	A	612	28.367	-12.077	-1.100	1.00	15.00	A	C
ATOM	4844	C	PRO	A	612	31.009	-13.536	0.557	1.00	15.00	A	C
ATOM	4845	O	PRO	A	612	31.222	-13.529	1.767	1.00	15.00	A	O
ATOM	4846	N	TYR	A	613	31.120	-14.635	-0.184	1.00	15.00	A	N
ATOM	4847	CA	TYR	A	613	31.511	-15.914	0.399	1.00	15.00	A	C
ATOM	4848	CB	TYR	A	613	30.308	-16.642	1.021	1.00	15.00	A	C
ATOM	4849	CG	TYR	A	613	29.226	-17.070	0.046	1.00	15.00	A	C
ATOM	4850	CD1	TYR	A	613	29.316	-18.276	-0.641	1.00	15.00	A	C
ATOM	4851	CD2	TYR	A	613	28.105	-16.279	-0.170	1.00	15.00	A	C
ATOM	4852	CE1	TYR	A	613	28.324	-18.679	-1.514	1.00	15.00	A	C
ATOM	4853	CE2	TYR	A	613	27.109	-16.675	-1.044	1.00	15.00	A	C
ATOM	4854	CZ	TYR	A	613	27.223	-17.876	-1.712	1.00	15.00	A	C
ATOM	4855	OH	TYR	A	613	26.231	-18.279	-2.576	1.00	15.00	A	O
ATOM	4856	C	TYR	A	613	32.229	-16.806	-0.613	1.00	15.00	A	C
ATOM	4857	O	TYR	A	613	32.453	-17.990	-0.362	1.00	15.00	A	O
ATOM	4858	N	ALA	A	614	32.599	-16.237	-1.752	1.00	15.00	A	N
ATOM	4859	CA	ALA	A	614	33.284	-16.997	-2.788	1.00	15.00	A	C
ATOM	4860	CB	ALA	A	614	32.465	-17.029	-4.070	1.00	15.00	A	C
ATOM	4861	C	ALA	A	614	34.677	-16.435	-3.045	1.00	15.00	A	C
ATOM	4862	O	ALA	A	614	34.925	-15.799	-4.071	1.00	15.00	A	O
ATOM	4863	N	ASP	A	615	35.573	-16.656	-2.094	1.00	15.00	A	N
ATOM	4864	CA	ASP	A	615	36.949	-16.180	-2.208	1.00	15.00	A	C
ATOM	4865	CB	ASP	A	615	37.684	-16.323	-0.874	1.00	15.00	A	C
ATOM	4866	CG	ASP	A	615	37.575	-15.083	-0.014	1.00	15.00	A	C
ATOM	4867	OD1	ASP	A	615	36.774	-15.094	0.944	1.00	15.00	A	O
ATOM	4868	OD2	ASP	A	615	38.290	-14.100	-0.299	1.00	15.00	A	O
ATOM	4869	C	ASP	A	615	37.704	-16.935	-3.294	1.00	15.00	A	C
ATOM	4870	O	ASP	A	615	37.471	-18.127	-3.513	1.00	15.00	A	O
TER	4871		ASP	A	615							
ATOM	4871	N	PHE	B	1	-19.955	20.147	-7.538	1.00	15.00	B	N
ATOM	4872	CA	PHE	B	1	-19.767	19.112	-6.533	1.00	15.00	B	C
ATOM	4873	CB	PHE	B	1	-19.216	19.714	-5.236	1.00	15.00	B	C
ATOM	4874	CG	PHE	B	1	-17.723	19.601	-5.092	1.00	15.00	B	C
ATOM	4875	CD1	PHE	B	1	-17.168	18.830	-4.083	1.00	15.00	B	C
ATOM	4876	CD2	PHE	B	1	-16.875	20.264	-5.965	1.00	15.00	B	C
ATOM	4877	CE1	PHE	B	1	-15.797	18.723	-3.946	1.00	15.00	B	C
ATOM	4878	CE2	PHE	B	1	-15.503	20.161	-5.834	1.00	15.00	B	C
ATOM	4879	CZ	PHE	B	1	-14.963	19.389	-4.823	1.00	15.00	B	C
ATOM	4880	C	PHE	B	1	-21.078	18.383	-6.258	1.00	15.00	B	C
ATOM	4881	O	PHE	B	1	-22.103	19.013	-5.994	1.00	15.00	B	O
ATOM	4882	N	PRO	B	2	-21.066	17.046	-6.344	1.00	15.00	B	N
ATOM	4883	CA	PRO	B	2	-22.255	16.231	-6.091	1.00	15.00	B	C
ATOM	4884	CB	PRO	B	2	-21.819	14.824	-6.510	1.00	15.00	B	C
ATOM	4885	CG	PRO	B	2	-20.337	14.830	-6.364	1.00	15.00	B	C
ATOM	4886	CD	PRO	B	2	-19.894	16.227	-6.693	1.00	15.00	B	C
ATOM	4887	C	PRO	B	2	-22.624	16.250	-4.613	1.00	15.00	B	C
ATOM	4888	O	PRO	B	2	-21.785	15.973	-3.754	1.00	15.00	B	O
ATOM	4889	N	ILE	B	3	-23.872	16.594	-4.327	1.00	15.00	B	N
ATOM	4890	CA	ILE	B	3	-24.358	16.655	-2.956	1.00	15.00	B	C
ATOM	4891	CB	ILE	B	3	-25.812	17.172	-2.892	1.00	15.00	B	C

ATOM	4892	CG1	ILE	B	3	-25.930	18.518	-3.617	1.00	15.00	B	C
ATOM	4893	CG2	ILE	B	3	-26.275	17.301	-1.446	1.00	15.00	B	C
ATOM	4894	CD1	ILE	B	3	-27.352	19.017	-3.763	1.00	15.00	B	C
ATOM	4895	C	ILE	B	3	-24.277	15.282	-2.294	1.00	15.00	B	C
ATOM	4896	O	ILE	B	3	-24.938	14.336	-2.724	1.00	15.00	B	O
ATOM	4897	N	PRO	B	4	-23.443	15.154	-1.253	1.00	15.00	B	N
ATOM	4898	CA	PRO	B	4	-23.285	13.896	-0.532	1.00	15.00	B	C
ATOM	4899	CB	PRO	B	4	-22.072	14.146	0.369	1.00	15.00	B	C
ATOM	4900	CG	PRO	B	4	-22.036	15.624	0.559	1.00	15.00	B	C
ATOM	4901	CD	PRO	B	4	-22.594	16.226	-0.701	1.00	15.00	B	C
ATOM	4902	C	PRO	B	4	-24.519	13.595	0.304	1.00	15.00	B	C
ATOM	4903	O	PRO	B	4	-24.855	14.342	1.226	1.00	15.00	B	O
ATOM	4904	N	LEU	B	5	-25.207	12.515	-0.038	1.00	15.00	B	N
ATOM	4905	CA	LEU	B	5	-26.407	12.123	0.682	1.00	15.00	B	C
ATOM	4906	CB	LEU	B	5	-27.045	10.889	0.039	1.00	15.00	B	C
ATOM	4907	CG	LEU	B	5	-27.579	11.070	-1.384	1.00	15.00	B	C
ATOM	4908	CD1	LEU	B	5	-28.111	9.753	-1.927	1.00	15.00	B	C
ATOM	4909	CD2	LEU	B	5	-28.659	12.142	-1.423	1.00	15.00	B	C
ATOM	4910	C	LEU	B	5	-26.081	11.850	2.144	1.00	15.00	B	C
ATOM	4911	O	LEU	B	5	-25.193	11.049	2.443	1.00	15.00	B	O
ATOM	4912	N	PRO	B	6	-26.773	12.542	3.071	1.00	15.00	B	N
ATOM	4913	CA	PRO	B	6	-26.559	12.370	4.509	1.00	15.00	B	C
ATOM	4914	CB	PRO	B	6	-27.715	13.152	5.137	1.00	15.00	B	C
ATOM	4915	CG	PRO	B	6	-28.058	14.185	4.121	1.00	15.00	B	C
ATOM	4916	CD	PRO	B	6	-27.813	13.545	2.782	1.00	15.00	B	C
ATOM	4917	C	PRO	B	6	-26.642	10.900	4.893	1.00	15.00	B	C
ATOM	4918	O	PRO	B	6	-27.709	10.294	4.837	1.00	15.00	B	O
ATOM	4919	N	TYR	B	7	-25.507	10.337	5.271	1.00	15.00	B	N
ATOM	4920	CA	TYR	B	7	-25.421	8.930	5.636	1.00	15.00	B	C
ATOM	4921	CB	TYR	B	7	-24.005	8.569	6.043	1.00	15.00	B	C
ATOM	4922	CG	TYR	B	7	-23.129	8.148	4.891	1.00	15.00	B	C
ATOM	4923	CD1	TYR	B	7	-23.491	7.083	4.083	1.00	15.00	B	C
ATOM	4924	CD2	TYR	B	7	-21.939	8.807	4.617	1.00	15.00	B	C
ATOM	4925	CE1	TYR	B	7	-22.693	6.685	3.034	1.00	15.00	B	C
ATOM	4926	CE2	TYR	B	7	-21.133	8.415	3.567	1.00	15.00	B	C
ATOM	4927	CZ	TYR	B	7	-21.517	7.352	2.779	1.00	15.00	B	C
ATOM	4928	OH	TYR	B	7	-20.722	6.952	1.734	1.00	15.00	B	O
ATOM	4929	C	TYR	B	7	-26.418	8.519	6.713	1.00	15.00	B	C
ATOM	4930	O	TYR	B	7	-26.993	7.435	6.646	1.00	15.00	B	O
ATOM	4931	N	TYR	B	8	-26.633	9.373	7.704	1.00	15.00	B	N
ATOM	4932	CA	TYR	B	8	-27.584	9.051	8.759	1.00	15.00	B	C
ATOM	4933	CB	TYR	B	8	-27.409	9.931	9.995	1.00	15.00	B	C
ATOM	4934	CG	TYR	B	8	-26.727	9.194	11.125	1.00	15.00	B	C
ATOM	4935	CD1	TYR	B	8	-27.467	8.611	12.145	1.00	15.00	B	C
ATOM	4936	CD2	TYR	B	8	-25.345	9.056	11.158	1.00	15.00	B	C
ATOM	4937	CE1	TYR	B	8	-26.851	7.911	13.164	1.00	15.00	B	C
ATOM	4938	CE2	TYR	B	8	-24.719	8.362	12.176	1.00	15.00	B	C
ATOM	4939	CZ	TYR	B	8	-25.477	7.790	13.176	1.00	15.00	B	C
ATOM	4940	OH	TYR	B	8	-24.864	7.083	14.184	1.00	15.00	B	O
ATOM	4941	C	TYR	B	8	-29.014	9.052	8.231	1.00	15.00	B	C
ATOM	4942	O	TYR	B	8	-29.902	8.420	8.801	1.00	15.00	B	O
ATOM	4943	N	TRP	B	9	-29.221	9.749	7.123	1.00	15.00	B	N
ATOM	4944	CA	TRP	B	9	-30.523	9.805	6.482	1.00	15.00	B	C
ATOM	4945	CB	TRP	B	9	-30.663	11.103	5.682	1.00	15.00	B	C
ATOM	4946	CG	TRP	B	9	-31.924	11.862	5.962	1.00	15.00	B	C
ATOM	4947	CD1	TRP	B	9	-32.403	12.235	7.185	1.00	15.00	B	C
ATOM	4948	CD2	TRP	B	9	-32.864	12.349	4.997	1.00	15.00	B	C
ATOM	4949	NE1	TRP	B	9	-33.586	12.917	7.041	1.00	15.00	B	N
ATOM	4950	CE2	TRP	B	9	-33.891	13.002	5.708	1.00	15.00	B	C
ATOM	4951	CE3	TRP	B	9	-32.939	12.294	3.602	1.00	15.00	B	C
ATOM	4952	CZ2	TRP	B	9	-34.978	13.597	5.070	1.00	15.00	B	C
ATOM	4953	CZ3	TRP	B	9	-34.018	12.885	2.972	1.00	15.00	B	C
ATOM	4954	CH2	TRP	B	9	-35.023	13.528	3.705	1.00	15.00	B	C
ATOM	4955	C	TRP	B	9	-30.643	8.601	5.554	1.00	15.00	B	C
ATOM	4956	O	TRP	B	9	-31.728	8.073	5.329	1.00	15.00	B	O
ATOM	4957	N	LEU	B	10	-29.493	8.163	5.049	1.00	15.00	B	N
ATOM	4958	CA	LEU	B	10	-29.408	7.011	4.159	1.00	15.00	B	C
ATOM	4959	CB	LEU	B	10	-28.006	6.950	3.533	1.00	15.00	B	C
ATOM	4960	CG	LEU	B	10	-27.775	5.891	2.452	1.00	15.00	B	C
ATOM	4961	CD1	LEU	B	10	-27.769	6.529	1.071	1.00	15.00	B	C
ATOM	4962	CD2	LEU	B	10	-26.476	5.138	2.705	1.00	15.00	B	C

ATOM	4963	C	LEU	B	10	-29.684	5.728	4.938	1.00	15.00	B	C
ATOM	4964	O	LEU	B	10	-29.984	4.686	4.364	1.00	15.00	B	O
ATOM	4965	N	TYR	B	11	-29.574	5.814	6.255	1.00	15.00	B	N
ATOM	4966	CA	TYR	B	11	-29.821	4.667	7.110	1.00	15.00	B	C
ATOM	4967	CB	TYR	B	11	-28.671	4.479	8.106	1.00	15.00	B	C
ATOM	4968	CG	TYR	B	11	-28.862	3.304	9.040	1.00	15.00	B	C
ATOM	4969	CD1	TYR	B	11	-29.320	3.491	10.338	1.00	15.00	B	C
ATOM	4970	CD2	TYR	B	11	-28.597	2.008	8.619	1.00	15.00	B	C
ATOM	4971	CE1	TYR	B	11	-29.507	2.421	11.190	1.00	15.00	B	C
ATOM	4972	CE2	TYR	B	11	-28.781	0.932	9.465	1.00	15.00	B	C
ATOM	4973	CZ	TYR	B	11	-29.237	1.144	10.748	1.00	15.00	B	C
ATOM	4974	OH	TYR	B	11	-29.419	0.077	11.594	1.00	15.00	B	O
ATOM	4975	C	TYR	B	11	-31.150	4.800	7.852	1.00	15.00	B	C
ATOM	4976	O	TYR	B	11	-31.808	3.808	8.145	1.00	15.00	B	O
ATOM	4977	N	ARG	B	12	-31.547	6.028	8.157	1.00	15.00	B	N
ATOM	4978	CA	ARG	B	12	-32.789	6.256	8.883	1.00	15.00	B	C
ATOM	4979	CB	ARG	B	12	-32.619	7.382	9.907	1.00	15.00	B	C
ATOM	4980	CG	ARG	B	12	-33.779	7.528	10.880	1.00	15.00	B	C
ATOM	4981	CD	ARG	B	12	-33.447	8.517	11.986	1.00	15.00	B	C
ATOM	4982	NE	ARG	B	12	-32.294	8.085	12.781	1.00	15.00	B	N
ATOM	4983	CZ	ARG	B	12	-31.689	8.843	13.698	1.00	15.00	B	C
ATOM	4984	NH1	ARG	B	12	-32.127	10.075	13.943	1.00	15.00	B	N
ATOM	4985	NH2	ARG	B	12	-30.644	8.368	14.369	1.00	15.00	B	N
ATOM	4986	C	ARG	B	12	-33.961	6.551	7.950	1.00	15.00	B	C
ATOM	4987	O	ARG	B	12	-34.956	5.827	7.942	1.00	15.00	B	O
ATOM	4988	N	ALA	B	13	-33.840	7.610	7.163	1.00	15.00	B	N
ATOM	4989	CA	ALA	B	13	-34.904	8.003	6.249	1.00	15.00	B	C
ATOM	4990	CB	ALA	B	13	-34.693	9.418	5.742	1.00	15.00	B	C
ATOM	4991	C	ALA	B	13	-35.039	7.028	5.089	1.00	15.00	B	C
ATOM	4992	O	ALA	B	13	-36.148	6.745	4.639	1.00	15.00	B	O
ATOM	4993	N	LEU	B	14	-33.915	6.520	4.601	1.00	15.00	B	N
ATOM	4994	CA	LEU	B	14	-33.937	5.571	3.498	1.00	15.00	B	C
ATOM	4995	CB	LEU	B	14	-32.542	5.338	2.926	1.00	15.00	B	C
ATOM	4996	CG	LEU	B	14	-32.434	5.343	1.399	1.00	15.00	B	C
ATOM	4997	CD1	LEU	B	14	-32.692	6.736	0.846	1.00	15.00	B	C
ATOM	4998	CD2	LEU	B	14	-31.076	4.828	0.952	1.00	15.00	B	C
ATOM	4999	C	LEU	B	14	-34.582	4.264	3.940	1.00	15.00	B	C
ATOM	5000	O	LEU	B	14	-35.336	3.655	3.186	1.00	15.00	B	O
ATOM	5001	N	ILE	B	15	-34.300	3.852	5.176	1.00	15.00	B	N
ATOM	5002	CA	ILE	B	15	-34.890	2.635	5.724	1.00	15.00	B	C
ATOM	5003	CB	ILE	B	15	-34.261	2.248	7.091	1.00	15.00	B	C
ATOM	5004	CG1	ILE	B	15	-33.196	1.160	6.911	1.00	15.00	B	C
ATOM	5005	CG2	ILE	B	15	-35.306	1.812	8.115	1.00	15.00	B	C
ATOM	5006	CD1	ILE	B	15	-33.735	-0.157	6.391	1.00	15.00	B	C
ATOM	5007	C	ILE	B	15	-36.399	2.829	5.848	1.00	15.00	B	C
ATOM	5008	O	ILE	B	15	-37.182	1.919	5.570	1.00	15.00	B	O
ATOM	5009	N	LYS	B	16	-36.793	4.037	6.242	1.00	15.00	B	N
ATOM	5010	CA	LYS	B	16	-38.201	4.379	6.372	1.00	15.00	B	C
ATOM	5011	CB	LYS	B	16	-38.364	5.758	7.010	1.00	15.00	B	C
ATOM	5012	CG	LYS	B	16	-38.713	5.725	8.486	1.00	15.00	B	C
ATOM	5013	CD	LYS	B	16	-39.288	7.056	8.934	1.00	15.00	B	C
ATOM	5014	CE	LYS	B	16	-39.873	6.967	10.332	1.00	15.00	B	C
ATOM	5015	NZ	LYS	B	16	-40.674	8.175	10.670	1.00	15.00	B	N
ATOM	5016	C	LYS	B	16	-38.854	4.370	4.999	1.00	15.00	B	C
ATOM	5017	O	LYS	B	16	-39.969	3.879	4.830	1.00	15.00	B	O
ATOM	5018	N	ARG	B	17	-38.143	4.922	4.019	1.00	15.00	B	N
ATOM	5019	CA	ARG	B	17	-38.627	4.956	2.648	1.00	15.00	B	C
ATOM	5020	CB	ARG	B	17	-37.678	5.755	1.754	1.00	15.00	B	C
ATOM	5021	CG	ARG	B	17	-37.839	7.259	1.870	1.00	15.00	B	C
ATOM	5022	CD	ARG	B	17	-36.897	7.985	0.926	1.00	15.00	B	C
ATOM	5023	NE	ARG	B	17	-37.147	9.427	0.900	1.00	15.00	B	N
ATOM	5024	CZ	ARG	B	17	-36.474	10.286	0.135	1.00	15.00	B	C
ATOM	5025	NH1	ARG	B	17	-35.507	9.848	-0.665	1.00	15.00	B	N
ATOM	5026	NH2	ARG	B	17	-36.769	11.583	0.171	1.00	15.00	B	N
ATOM	5027	C	ARG	B	17	-38.751	3.534	2.132	1.00	15.00	B	C
ATOM	5028	O	ARG	B	17	-39.670	3.210	1.385	1.00	15.00	B	O
ATOM	5029	N	ILE	B	18	-37.807	2.696	2.543	1.00	15.00	B	N
ATOM	5030	CA	ILE	B	18	-37.808	1.296	2.171	1.00	15.00	B	C
ATOM	5031	CB	ILE	B	18	-36.527	0.574	2.665	1.00	15.00	B	C
ATOM	5032	CG1	ILE	B	18	-35.441	0.620	1.588	1.00	15.00	B	C
ATOM	5033	CG2	ILE	B	18	-36.797	-0.866	3.093	1.00	15.00	B	C

ATOM	5034	CD1	ILE	B	18	-35.801	-0.141	0.331	1.00	15.00	B	C
ATOM	5035	C	ILE	B	18	-39.053	0.628	2.734	1.00	15.00	B	C
ATOM	5036	O	ILE	B	18	-39.775	-0.033	2.010	1.00	15.00	B	O
ATOM	5037	N	GLN	B	19	-39.315	0.852	4.020	1.00	15.00	B	N
ATOM	5038	CA	GLN	B	19	-40.479	0.273	4.685	1.00	15.00	B	C
ATOM	5039	CB	GLN	B	19	-40.510	0.678	6.159	1.00	15.00	B	C
ATOM	5040	CG	GLN	B	19	-39.520	-0.073	7.030	1.00	15.00	B	C
ATOM	5041	CD	GLN	B	19	-39.428	0.500	8.429	1.00	15.00	B	C
ATOM	5042	OE1	GLN	B	19	-39.555	1.707	8.633	1.00	15.00	B	O
ATOM	5043	NE2	GLN	B	19	-39.216	-0.366	9.407	1.00	15.00	B	N
ATOM	5044	C	GLN	B	19	-41.777	0.688	4.004	1.00	15.00	B	C
ATOM	5045	O	GLN	B	19	-42.713	-0.102	3.897	1.00	15.00	B	O
ATOM	5046	N	ALA	B	20	-41.819	1.927	3.535	1.00	15.00	B	N
ATOM	5047	CA	ALA	B	20	-42.998	2.446	2.862	1.00	15.00	B	C
ATOM	5048	CB	ALA	B	20	-42.953	3.967	2.814	1.00	15.00	B	C
ATOM	5049	C	ALA	B	20	-43.128	1.876	1.452	1.00	15.00	B	C
ATOM	5050	O	ALA	B	20	-44.180	1.987	0.824	1.00	15.00	B	O
ATOM	5051	N	LEU	B	21	-42.058	1.268	0.959	1.00	15.00	B	N
ATOM	5052	CA	LEU	B	21	-42.061	0.703	-0.383	1.00	15.00	B	C
ATOM	5053	CB	LEU	B	21	-41.201	1.561	-1.319	1.00	15.00	B	C
ATOM	5054	CG	LEU	B	21	-41.613	3.031	-1.458	1.00	15.00	B	C
ATOM	5055	CD1	LEU	B	21	-40.555	3.811	-2.220	1.00	15.00	B	C
ATOM	5056	CD2	LEU	B	21	-42.966	3.153	-2.147	1.00	15.00	B	C
ATOM	5057	C	LEU	B	21	-41.576	-0.746	-0.396	1.00	15.00	B	C
ATOM	5058	O	LEU	B	21	-41.068	-1.223	-1.409	1.00	15.00	B	O
ATOM	5059	N	ILE	B	22	-41.727	-1.450	0.720	1.00	15.00	B	N
ATOM	5060	CA	ILE	B	22	-41.299	-2.843	0.784	1.00	15.00	B	C
ATOM	5061	CB	ILE	B	22	-39.904	-3.062	1.438	1.00	15.00	B	C
ATOM	5062	CG1	ILE	B	22	-39.372	-4.457	1.081	1.00	15.00	B	C
ATOM	5063	CG2	ILE	B	22	-39.946	-2.878	2.954	1.00	15.00	B	C
ATOM	5064	CD1	ILE	B	22	-38.066	-4.823	1.758	1.00	15.00	B	C
ATOM	5065	C	ILE	B	22	-42.353	-3.771	1.390	1.00	15.00	B	C
ATOM	5066	O	ILE	B	22	-42.663	-3.710	2.580	1.00	15.00	B	O
ATOM	5067	N	PRO	B	23	-42.971	-4.602	0.547	1.00	15.00	B	N
ATOM	5068	CA	PRO	B	23	-43.956	-5.586	0.986	1.00	15.00	B	C
ATOM	5069	CB	PRO	B	23	-44.726	-5.928	-0.302	1.00	15.00	B	C
ATOM	5070	CG	PRO	B	23	-44.167	-5.037	-1.371	1.00	15.00	B	C
ATOM	5071	CD	PRO	B	23	-42.806	-4.612	-0.903	1.00	15.00	B	C
ATOM	5072	C	PRO	B	23	-43.253	-6.836	1.516	1.00	15.00	B	C
ATOM	5073	O	PRO	B	23	-42.034	-6.973	1.373	1.00	15.00	B	O
ATOM	5074	N	LYS	B	24	-44.018	-7.762	2.090	1.00	15.00	B	N
ATOM	5075	CA	LYS	B	24	-43.447	-9.000	2.629	1.00	15.00	B	C
ATOM	5076	CB	LYS	B	24	-44.497	-9.810	3.394	1.00	15.00	B	C
ATOM	5077	CG	LYS	B	24	-44.863	-9.220	4.746	1.00	15.00	B	C
ATOM	5078	CD	LYS	B	24	-45.129	-10.309	5.772	1.00	15.00	B	C
ATOM	5079	CE	LYS	B	24	-45.066	-9.762	7.191	1.00	15.00	B	C
ATOM	5080	NZ	LYS	B	24	-45.044	-10.849	8.210	1.00	15.00	B	N
ATOM	5081	C	LYS	B	24	-42.811	-9.839	1.524	1.00	15.00	B	C
ATOM	5082	O	LYS	B	24	-41.970	-10.696	1.778	1.00	15.00	B	O
ATOM	5083	N	GLY	B	25	-43.234	-9.577	0.300	1.00	15.00	B	N
ATOM	5084	CA	GLY	B	25	-42.701	-10.267	-0.856	1.00	15.00	B	C
ATOM	5085	C	GLY	B	25	-42.298	-9.246	-1.895	1.00	15.00	B	C
ATOM	5086	O	GLY	B	25	-42.522	-9.426	-3.093	1.00	15.00	B	O
ATOM	5087	N	GLY	B	26	-41.713	-8.157	-1.412	1.00	15.00	B	N
ATOM	5088	CA	GLY	B	26	-41.304	-7.071	-2.272	1.00	15.00	B	C
ATOM	5089	C	GLY	B	26	-40.101	-7.363	-3.139	1.00	15.00	B	C
ATOM	5090	O	GLY	B	26	-38.966	-7.110	-2.749	1.00	15.00	B	O
ATOM	5091	N	ARG	B	27	-40.353	-7.891	-4.322	1.00	15.00	B	N
ATOM	5092	CA	ARG	B	27	-39.287	-8.178	-5.267	1.00	15.00	B	C
ATOM	5093	CB	ARG	B	27	-39.544	-9.500	-5.994	1.00	15.00	B	C
ATOM	5094	CG	ARG	B	27	-39.329	-10.733	-5.123	1.00	15.00	B	C
ATOM	5095	CD	ARG	B	27	-37.894	-10.808	-4.615	1.00	15.00	B	C
ATOM	5096	NE	ARG	B	27	-37.627	-12.032	-3.849	1.00	15.00	B	N
ATOM	5097	CZ	ARG	B	27	-36.432	-12.633	-3.802	1.00	15.00	B	C
ATOM	5098	NH1	ARG	B	27	-35.404	-12.127	-4.473	1.00	15.00	B	N
ATOM	5099	NH2	ARG	B	27	-36.261	-13.737	-3.079	1.00	15.00	B	N
ATOM	5100	C	ARG	B	27	-39.152	-7.018	-6.251	1.00	15.00	B	C
ATOM	5101	O	ARG	B	27	-38.152	-6.886	-6.950	1.00	15.00	B	O
ATOM	5102	N	LEU	B	28	-40.179	-6.175	-6.284	1.00	15.00	B	N
ATOM	5103	CA	LEU	B	28	-40.195	-5.005	-7.155	1.00	15.00	B	C
ATOM	5104	CB	LEU	B	28	-41.462	-5.000	-8.014	1.00	15.00	B	C

ATOM	5105	CG	LEU	B	28	-41.637	-6.179	-8.973	1.00	15.00	B	C
ATOM	5106	CD1	LEU	B	28	-43.012	-6.141	-9.620	1.00	15.00	B	C
ATOM	5107	CD2	LEU	B	28	-40.547	-6.172	-10.033	1.00	15.00	B	C
ATOM	5108	C	LEU	B	28	-40.136	-3.726	-6.325	1.00	15.00	B	C
ATOM	5109	O	LEU	B	28	-39.774	-2.665	-6.828	1.00	15.00	B	O
ATOM	5110	N	LEU	B	29	-40.490	-3.851	-5.044	1.00	15.00	B	N
ATOM	5111	CA	LEU	B	29	-40.499	-2.717	-4.112	1.00	15.00	B	C
ATOM	5112	CB	LEU	B	29	-41.562	-1.678	-4.501	1.00	15.00	B	C
ATOM	5113	CG	LEU	B	29	-43.007	-1.985	-4.096	1.00	15.00	B	C
ATOM	5114	CD1	LEU	B	29	-43.827	-0.705	-4.052	1.00	15.00	B	C
ATOM	5115	CD2	LEU	B	29	-43.636	-2.986	-5.053	1.00	15.00	B	C
ATOM	5116	C	LEU	B	29	-39.124	-2.059	-4.011	1.00	15.00	B	C
ATOM	5117	O	LEU	B	29	-38.928	-0.944	-4.493	1.00	15.00	B	O
ATOM	5118	N	PRO	B	30	-38.151	-2.743	-3.379	1.00	15.00	B	N
ATOM	5119	CA	PRO	B	30	-36.781	-2.228	-3.236	1.00	15.00	B	C
ATOM	5120	CB	PRO	B	30	-36.025	-3.399	-2.606	1.00	15.00	B	C
ATOM	5121	CG	PRO	B	30	-37.075	-4.158	-1.877	1.00	15.00	B	C
ATOM	5122	CD	PRO	B	30	-38.313	-4.048	-2.719	1.00	15.00	B	C
ATOM	5123	C	PRO	B	30	-36.168	-1.866	-4.586	1.00	15.00	B	C
ATOM	5124	O	PRO	B	30	-35.250	-1.060	-4.662	1.00	15.00	B	O
ATOM	5125	N	GLN	B	31	-36.698	-2.459	-5.652	1.00	15.00	B	N
ATOM	5126	CA	GLN	B	31	-36.219	-2.186	-6.997	1.00	15.00	B	C
ATOM	5127	CB	GLN	B	31	-36.905	-3.110	-7.999	1.00	15.00	B	C
ATOM	5128	CG	GLN	B	31	-36.111	-3.346	-9.267	1.00	15.00	B	C
ATOM	5129	CD	GLN	B	31	-36.981	-3.369	-10.504	1.00	15.00	B	C
ATOM	5130	OE1	GLN	B	31	-37.065	-2.382	-11.235	1.00	15.00	B	O
ATOM	5131	NE2	GLN	B	31	-37.646	-4.484	-10.731	1.00	15.00	B	N
ATOM	5132	C	GLN	B	31	-36.501	-0.732	-7.361	1.00	15.00	B	C
ATOM	5133	O	GLN	B	31	-35.695	-0.077	-8.022	1.00	15.00	B	O
ATOM	5134	N	LEU	B	32	-37.654	-0.237	-6.921	1.00	15.00	B	N
ATOM	5135	CA	LEU	B	32	-38.047	1.141	-7.177	1.00	15.00	B	C
ATOM	5136	CB	LEU	B	32	-39.513	1.358	-6.789	1.00	15.00	B	C
ATOM	5137	CG	LEU	B	32	-40.089	2.751	-7.055	1.00	15.00	B	C
ATOM	5138	CD1	LEU	B	32	-40.114	3.049	-8.548	1.00	15.00	B	C
ATOM	5139	CD2	LEU	B	32	-41.481	2.878	-6.457	1.00	15.00	B	C
ATOM	5140	C	LEU	B	32	-37.147	2.081	-6.387	1.00	15.00	B	C
ATOM	5141	O	LEU	B	32	-36.712	3.117	-6.892	1.00	15.00	B	O
ATOM	5142	N	VAL	B	33	-36.861	1.698	-5.147	1.00	15.00	B	N
ATOM	5143	CA	VAL	B	33	-35.995	2.487	-4.281	1.00	15.00	B	C
ATOM	5144	CB	VAL	B	33	-35.962	1.916	-2.848	1.00	15.00	B	C
ATOM	5145	CG1	VAL	B	33	-35.169	2.827	-1.921	1.00	15.00	B	C
ATOM	5146	CG2	VAL	B	33	-37.375	1.713	-2.321	1.00	15.00	B	C
ATOM	5147	C	VAL	B	33	-34.584	2.494	-4.855	1.00	15.00	B	C
ATOM	5148	O	VAL	B	33	-33.934	3.538	-4.927	1.00	15.00	B	O
ATOM	5149	N	TYR	B	34	-34.139	1.320	-5.287	1.00	15.00	B	N
ATOM	5150	CA	TYR	B	34	-32.822	1.149	-5.882	1.00	15.00	B	C
ATOM	5151	CB	TYR	B	34	-32.637	-0.310	-6.305	1.00	15.00	B	C
ATOM	5152	CG	TYR	B	34	-31.203	-0.783	-6.314	1.00	15.00	B	C
ATOM	5153	CD1	TYR	B	34	-30.611	-1.232	-7.485	1.00	15.00	B	C
ATOM	5154	CD2	TYR	B	34	-30.445	-0.788	-5.151	1.00	15.00	B	C
ATOM	5155	CE1	TYR	B	34	-29.304	-1.673	-7.498	1.00	15.00	B	C
ATOM	5156	CE2	TYR	B	34	-29.135	-1.226	-5.155	1.00	15.00	B	C
ATOM	5157	CZ	TYR	B	34	-28.571	-1.669	-6.332	1.00	15.00	B	C
ATOM	5158	OH	TYR	B	34	-27.268	-2.108	-6.345	1.00	15.00	B	O
ATOM	5159	C	TYR	B	34	-32.660	2.067	-7.090	1.00	15.00	B	C
ATOM	5160	O	TYR	B	34	-31.592	2.642	-7.305	1.00	15.00	B	O
ATOM	5161	N	ARG	B	35	-33.731	2.202	-7.873	1.00	15.00	B	N
ATOM	5162	CA	ARG	B	35	-33.721	3.067	-9.050	1.00	15.00	B	C
ATOM	5163	CB	ARG	B	35	-35.071	3.020	-9.775	1.00	15.00	B	C
ATOM	5164	CG	ARG	B	35	-35.368	1.715	-10.496	1.00	15.00	B	C
ATOM	5165	CD	ARG	B	35	-36.742	1.756	-11.149	1.00	15.00	B	C
ATOM	5166	NE	ARG	B	35	-37.139	0.458	-11.698	1.00	15.00	B	N
ATOM	5167	CZ	ARG	B	35	-37.754	0.297	-12.871	1.00	15.00	B	C
ATOM	5168	NH1	ARG	B	35	-38.044	1.352	-13.628	1.00	15.00	B	N
ATOM	5169	NH2	ARG	B	35	-38.080	-0.924	-13.282	1.00	15.00	B	N
ATOM	5170	C	ARG	B	35	-33.425	4.503	-8.635	1.00	15.00	B	C
ATOM	5171	O	ARG	B	35	-32.644	5.202	-9.282	1.00	15.00	B	O
ATOM	5172	N	LEU	B	36	-34.052	4.927	-7.545	1.00	15.00	B	N
ATOM	5173	CA	LEU	B	36	-33.862	6.272	-7.023	1.00	15.00	B	C
ATOM	5174	CB	LEU	B	36	-34.878	6.564	-5.914	1.00	15.00	B	C
ATOM	5175	CG	LEU	B	36	-36.355	6.420	-6.290	1.00	15.00	B	C

ATOM	5176	CD1	LEU	B	36	-37.235	6.557	-5.058	1.00	15.00	B	C
ATOM	5177	CD2	LEU	B	36	-36.745	7.445	-7.345	1.00	15.00	B	C
ATOM	5178	C	LEU	B	36	-32.446	6.431	-6.485	1.00	15.00	B	C
ATOM	5179	O	LEU	B	36	-31.789	7.441	-6.731	1.00	15.00	B	O
ATOM	5180	N	VAL	B	37	-31.982	5.417	-5.761	1.00	15.00	B	N
ATOM	5181	CA	VAL	B	37	-30.642	5.426	-5.185	1.00	15.00	B	C
ATOM	5182	CB	VAL	B	37	-30.375	4.155	-4.344	1.00	15.00	B	C
ATOM	5183	CG1	VAL	B	37	-28.926	4.103	-3.876	1.00	15.00	B	C
ATOM	5184	CG2	VAL	B	37	-31.317	4.097	-3.152	1.00	15.00	B	C
ATOM	5185	C	VAL	B	37	-29.580	5.554	-6.275	1.00	15.00	B	C
ATOM	5186	O	VAL	B	37	-28.700	6.411	-6.196	1.00	15.00	B	O
ATOM	5187	N	LEU	B	38	-29.686	4.719	-7.305	1.00	15.00	B	N
ATOM	5188	CA	LEU	B	38	-28.732	4.735	-8.411	1.00	15.00	B	C
ATOM	5189	CB	LEU	B	38	-29.038	3.615	-9.410	1.00	15.00	B	C
ATOM	5190	CG	LEU	B	38	-28.853	2.180	-8.907	1.00	15.00	B	C
ATOM	5191	CD1	LEU	B	38	-29.175	1.184	-10.011	1.00	15.00	B	C
ATOM	5192	CD2	LEU	B	38	-27.440	1.968	-8.386	1.00	15.00	B	C
ATOM	5193	C	LEU	B	38	-28.734	6.081	-9.129	1.00	15.00	B	C
ATOM	5194	O	LEU	B	38	-27.695	6.549	-9.593	1.00	15.00	B	O
ATOM	5195	N	ARG	B	39	-29.902	6.706	-9.191	1.00	15.00	B	N
ATOM	5196	CA	ARG	B	39	-30.056	7.993	-9.858	1.00	15.00	B	C
ATOM	5197	CB	ARG	B	39	-31.542	8.311	-10.051	1.00	15.00	B	C
ATOM	5198	CG	ARG	B	39	-31.833	9.324	-11.147	1.00	15.00	B	C
ATOM	5199	CD	ARG	B	39	-33.325	9.585	-11.274	1.00	15.00	B	C
ATOM	5200	NE	ARG	B	39	-33.648	10.373	-12.466	1.00	15.00	B	N
ATOM	5201	CZ	ARG	B	39	-34.834	10.942	-12.687	1.00	15.00	B	C
ATOM	5202	NH1	ARG	B	39	-35.814	10.811	-11.798	1.00	15.00	B	N
ATOM	5203	NH2	ARG	B	39	-35.041	11.640	-13.801	1.00	15.00	B	N
ATOM	5204	C	ARG	B	39	-29.369	9.122	-9.087	1.00	15.00	B	C
ATOM	5205	O	ARG	B	39	-29.127	10.195	-9.636	1.00	15.00	B	O
ATOM	5206	N	TYR	B	40	-29.053	8.878	-7.820	1.00	15.00	B	N
ATOM	5207	CA	TYR	B	40	-28.404	9.891	-6.991	1.00	15.00	B	C
ATOM	5208	CB	TYR	B	40	-29.306	10.289	-5.817	1.00	15.00	B	C
ATOM	5209	CG	TYR	B	40	-30.644	10.859	-6.244	1.00	15.00	B	C
ATOM	5210	CD1	TYR	B	40	-30.717	11.875	-7.189	1.00	15.00	B	C
ATOM	5211	CD2	TYR	B	40	-31.833	10.377	-5.710	1.00	15.00	B	C
ATOM	5212	CE1	TYR	B	40	-31.932	12.393	-7.592	1.00	15.00	B	C
ATOM	5213	CE2	TYR	B	40	-33.055	10.891	-6.107	1.00	15.00	B	C
ATOM	5214	CZ	TYR	B	40	-33.098	11.898	-7.049	1.00	15.00	B	C
ATOM	5215	OH	TYR	B	40	-34.312	12.409	-7.452	1.00	15.00	B	O
ATOM	5216	C	TYR	B	40	-27.033	9.427	-6.497	1.00	15.00	B	C
ATOM	5217	O	TYR	B	40	-26.463	10.006	-5.571	1.00	15.00	B	O
ATOM	5218	N	SER	B	41	-26.502	8.389	-7.127	1.00	15.00	B	N
ATOM	5219	CA	SER	B	41	-25.197	7.858	-6.749	1.00	15.00	B	C
ATOM	5220	CB	SER	B	41	-25.284	6.354	-6.481	1.00	15.00	B	C
ATOM	5221	OG	SER	B	41	-26.158	6.076	-5.401	1.00	15.00	B	O
ATOM	5222	C	SER	B	41	-24.143	8.146	-7.819	1.00	15.00	B	C
ATOM	5223	O	SER	B	41	-23.690	9.287	-7.986	1.00	15.00	B	O
TER	5224		SER	B	41							
END												

Prodigy Webserver

https://wenmr.science.uu.nl › prodigy

Prodigy (protein-protein option)

Prodigy Protein- Protein Binding-Affinity Predictions for ACE2 – BYL Complex

[+] No. of intermolecular contacts: 81
[+] No. of charged-charged contacts: 2
[+] No. of charged-polar contacts: 6
[+] No. of charged-apolar contacts: 32
[+] No. of polar-polar contacts: 3
[+] No. of apolar-polar contacts: 16
[+] No. of apolar-apolar contacts: 22
[+] Percentage of apolar NIS residues: 35.73
[+] Percentage of charged NIS residues: 27.45
[++] Predicted binding affinity (kcal.mol-1): -11.9
[++] Predicted dissociation constant (M) at 37.0˚C: 4.0e-09

Prodigy Predicted Contact Residues

ACE2 (Chain A) BYL(Chain B)

THR	27	A	ARG	27	B
HIS	34	A	LEU	14	B
THR	92	A	LEU	38	B
THR	27	A	LEU	28	B
GLN	552	A	PHE	1	B
VAL	93	A	TYR	34	B
HIS	34	A	ILE	15	B
GLU	23	A	ARG	27	B
ALA	387	A	ILE	3	B
THR	27	A	GLY	26	B
LYS	26	A	TYR	34	B
SER	19	A	LEU	28	B
GLU	35	A	ILE	15	B
ASP	30	A	VAL	33	B
ASP	30	A	TYR	34	B
ALA	387	A	PRO	4	B
LYS	26	A	GLN	31	B
LYS	31	A	ILE	22	B
THR	27	A	PRO	30	B
LYS	353	A	TYR	8	B
HIS	34	A	LEU	10	B
ALA	25	A	ARG	27	B
ARG	393	A	TYR	7	B
VAL	93	A	LEU	38	B
ALA	386	A	PRO	4	B
THR	92	A	TYR	34	B
LYS	31	A	ILE	18	B
GLU	23	A	LEU	28	B
HIS	34	A	VAL	33	B
ASP	38	A	TYR	11	B
ARG	393	A	LEU	10	B

ASP	382	A	TYR	7	B
PRO	389	A	SER	41	B
ASP	30	A	ILE	18	B
ASN	90	A	TYR	34	B
ARG	559	A	PRO	2	B
LEU	29	A	TYR	34	B
SER	19	A	GLN	31	B
ARG	559	A	SER	41	B
THR	27	A	ILE	22	B
HIS	34	A	TYR	11	B
ALA	387	A	TYR	7	B
LYS	26	A	PRO	30	B
ASP	30	A	PRO	30	B
GLN	388	A	SER	41	B
PHE	555	A	PRO	2	B
GLU	35	A	TYR	11	B
GLU	37	A	TYR	11	B
GLN	388	A	TYR	7	B
LYS	31	A	ILE	15	B
LYS	353	A	TYR	11	B
ALA	387	A	LEU	5	B
ASN	33	A	TYR	34	B
GLU	23	A	ARG	35	B
LEU	320	A	PHE	1	B
PRO	321	A	PHE	1	B
SER	19	A	ARG	27	B
THR	27	A	GLN	31	B
GLN	96	A	TYR	34	B
ALA	386	A	TYR	7	B
ALA	387	A	TYR	40	B
GLU	37	A	TYR	7	B
HIS	34	A	ILE	18	B
ASN	90	A	LEU	38	B
PHE	555	A	PHE	1	B
PHE	356	A	TYR	7	B
MET	383	A	PRO	4	B
LYS	353	A	TYR	7	B
GLU	23	A	GLN	31	B
ALA	386	A	LEU	5	B
THR	27	A	LEU	29	B
ALA	387	A	SER	41	B
GLY	354	A	TYR	7	B
GLY	319	A	PHE	1	B
ASP	30	A	VAL	37	B
GLN	24	A	ARG	27	B
TYR	385	A	TYR	7	B
TYR	83	A	ARG	27	B
GLY	352	A	TYR	7	B
GLU	37	A	LEU	10	B
TYR	41	A	TYR	8	B

Binding data o BYL surfactant peptide with hACE2 receptor protein derived from experimentally determined peptide-protein interaction data using Surface Plasmon Resonance.

	ka (1/Ms)	kd (1/s)	Rmax (RU)	RI (RU)	Conc of analyte	KA (1/M)	KD (M)	Req (RU)	kobs (1/s)
0.5µg/ml BYL to hACE2	2.19e4	2.79e-5	258	2.12	1.05e-7	7.85e8	1.27e-9	255	2.33e-3

Surface Plasmon Resonance metrics derived from the time course of binding of the ACE2 to the BYL peptide. Association and dissociation kinetic rate constants (k_{on}, k_{off}) and equilibrium dissociation constants (K_D), calculated from surface plasmon resonance (SPR) kinetic measurements for the hACE2 was attached to the Biacore sensor chip while SMB and BYL peptides were flowed in the anylate. Peptide was dissolved in running buffer (10 mM HEPES, 150 mM NaCl, 3 mM EDTA, 0.005% Surfactant P20, pH 7.4) and was flowed past the recombinant protein constructs on the CSM sensor chip with a Biacore system. Kinetic rate constants and equilibrium dissociation constants were determined from curve fitting analysis of SPR traces.
