# Supplementary material for: Lung Surfactant Protein B Peptide Mimics Interact with the Human ACE2 Receptor
Source: Int J Mol Sci. 2023 Jun 29;24(13):10837. doi: 10.3390/ijms241310837 (PMC10341807; doi:10.3390/ijms241310837)
Supplement: Supplementary file 1 [file ijms-24-10837-s001.zip › File S3_SPR_Data.pdf]

S3. Supplement file – ACE2- SMB and B-YL Surface Plasmon Resonance Data

0.5µg/ml SMB ox to hACE2_X0.5µg/ml SMB ox to hACE2_Y0.5µg/ml BYL to hACE2_X
0.5µg/ml BYL to hACE2_Y

-47	-0.338867	-47	-1.34668
-46	-0.365234	-46	-1.29883
-45	-0.368164	-45	-1.25684
-44	-0.375977	-44	-1.2207
-43	-0.375977	-43	-1.16895
-42	-0.387695	-42	-1.14648
-41	-0.37793	-41	-1.10449
-40	-0.396484	-40	-1.08203
-39	-0.423828	-39	-1.08008
-38	-0.436523	-38	-1.07227
-37	-0.460938	-37	-1.0625
-36	-0.410156	-36	-1.01953
-35	-0.560547	-35	-1.09766
-34	-0.524414	-34	-1.01758
-33	-0.376953	-33	-0.84082
-32	-0.347656	-32	-0.751953
-31	-0.355469	-31	-0.745117
-30	-0.354492	-30	-0.757813
-29	-0.356445	-29	-0.868164
-28	-0.261719	-28	-0.821289
-27	-0.134766	-27	-0.539063
-26	-0.121094	-26	-0.537109
-25	-0.118164	-25	-0.489258
-24	-0.0976563	-24	-0.432617
-23	-0.0664063	-23	-0.370117
-22	-0.0361328	-22	-0.341797
-21	-0.0136719	-21	-0.361328
-20	7.8125e-3	-20	-0.366211
-19	0.0195313	-19	-0.368164
-18	0.0351563	-18	-0.368164
-17	0.0400391	-17	-0.374023
-16	0.0488281	-16	-0.347656
-15	0.0488281	-15	-0.318359
-14	0.0537109	-14	-0.264648
-13	0.0439453	-13	-0.217773
-12	0.0488281	-12	-0.191406
-11	0.0449219	-11	-0.162109
-10	0.0253906	-10	-0.132813
-9	0.0400391	-9	-0.103516

-8	0.0429688	-8	-0.0742188
-7	0.0361328	-7	-0.0332031
-6	0.0302734	-6	1.95313e-3
-5	0.0175781	-5	0.0117188
-4	-9.76563e-4	-4	7.8125e-3
-3	-0.0136719	-3	6.83594e-3
-2	-0.0205078	-2	-0.0195313
-1	-0.0126953	-1	-8.78906e-3
0	-0.0449219	0	-0.0351563
1	-0.428711	1	-0.34668
2	0.734375	2	1.91992
3	-0.37207	3	0.118164
4	8.64063	4	10.0205
5	4.36621	5	1.6875
6	7.52734	6	2.22559
7	10.9844	7	2.89844
8	14.6738	8	3.4834
9	18.6504	9	4.03027
10	22.5557	10	4.60156
11	26.5898	11	5.13379
12	30.8271	12	5.75195
13	35.0059	13	6.27344
14	39.1514	14	6.76465
15	43.2217	15	7.29199
16	47.3418	16	7.80664
17	51.5244	17	8.34668
18	55.6318	18	8.91992
19	59.8994	19	9.57715
20	64.1719	20	10.2256
21	68.3809	21	10.7139
22	72.6143	22	11.2773
23	76.8213	23	11.8955
24	81.0625	24	12.5
25	85.335 25	13.0322	
26	89.5713	26	13.6016
27	93.832 27	14.2012	
28	98.0703	28	14.7754
29	102.394	29	15.3066
30	106.602	30	15.8965
31	110.851	31	16.5342
32	115.03 32	17.1143	
33	119.262	33	17.665
34	123.404	34	18.2168
35	127.497	35	18.7871

36	131.674	36	19.3281
37	135.693	37	19.9121
38	139.788	38	20.498
39	143.852	39	21.0469
40	147.912	40	21.5957
41	151.997	41	22.0957
42	156.058	42	22.6465
43	160.196	43	23.209
44	164.347	44	23.791
45	168.437	45	24.2939
46	172.519	46	24.8652
47	176.659	47	25.416
48	180.671	48	25.9277
49	184.77 49	26.4375	
50	188.83 50	26.9492	
51	192.925	51	27.4473
52	196.944	52	27.9492
53	201.035	53	28.4473
54	205.104	54	29.0195
55	209.198	55	29.5557
56	213.235	56	30.085
57	217.252	57	30.6035
58	221.402	58	31.1211
59	225.41 59	31.6455	
60	229.397	60	32.1963
61	233.432	61	32.7139
62	237.432	62	33.2236
63	241.487	63	33.7275
64	245.577	64	34.2285
65	249.688	65	34.7402
66	253.766	66	35.2188
67	257.801	67	35.7129
68	261.798	68	36.209
69	265.824	69	36.7383
70	269.844	70	37.2578
71	273.881	71	37.7383
72	277.898	72	38.2559
73	281.93 73	38.7578	
74	285.94 74	39.1992	
75	290.001	75	39.6309
76	294.041	76	40.0918
77	298.021	77	40.6113
78	302.088	78	41.1289
79	306.135	79	41.5801

80	310.156	80	42.0488
81	314.198	81	42.5273
82	318.201	82	43.0371
83	322.208	83	43.4922
84	326.239	84	43.9453
85	330.218	85	44.4688
86	334.236	86	44.9297
87	338.187	87	45.4004
88	342.237	88	45.873
89	346.247	89	46.3789
90	350.274	90	46.8262
91	354.298	91	47.2676
92	358.285	92	47.7168
93	362.283	93	48.2168
94	366.288	94	48.7129
95	370.347	95	49.1719
96	374.301	96	49.6289
97	378.288	97	50.0664
98	382.347	98	50.4785
99	386.397	99	50.9609
100	390.376	100	51.4375
101	394.366	101	51.9199
102	398.315	102	52.373
103	402.274	103	52.8242
104	406.296	104	53.291
105	410.376	105	53.7344
106	414.376	106	54.1504
107	418.285	107	54.6074
108	422.269	108	55.1387
109	426.235	109	55.6074
110	430.21 110	56.0391	
111	434.176	111	56.4873
112	438.146	112	56.9238
113	442.061	113	57.3496
114	445.959	114	57.8066
115	449.914	115	58.2461
116	453.912	116	58.6582
117	457.895	117	59.0684
118	461.846	118	59.5215
119	465.817	119	59.9922
120	469.843	120	60.4316
121	473.806	121	60.8613
122	477.798	122	61.2813
123	481.679	123	61.6914

124	485.657	124	62.0947
125	489.625	125	62.5146
126	493.641	126	62.9219
127	497.591	127	63.333
128	501.589	128	63.8125
129	505.526	129	64.2305
130	509.513	130	64.6504
131	513.506	131	65.0801
132	517.481	132	65.4805
133	521.466	133	65.9004
134	525.41	134	66.3652
135	529.355	135	66.8281
136	533.247	136	67.2207
137	537.159	137	67.6016
138	541.128	138	68.0098
139	545.002	139	68.4199
140	548.968	140	68.8398
141	552.907	141	69.25
142	556.833	142	69.6484
143	560.754	143	70.0625
144	564.673	144	70.5352
145	568.566	145	70.998
146	572.478	146	71.4004
147	576.44	147	71.7715
148	580.314	148	72.1816
149	584.251	149	72.6074
150	588.174	150	73.0146
151	592.043	151	73.4141
152	595.917	152	73.8203
153	599.816	153	74.2109
154	603.656	154	74.6309
155	607.417	155	75.0508
156	611.345	156	75.4492
157	615.193	157	75.8438
158	619.03	158	76.2266
159	622.862	159	76.6465
160	626.702	160	77.1113
161	630.562	161	77.5273
162	634.411	162	77.9375
163	638.184	163	78.3887
164	641.852	164	78.8164
165	645.603	165	79.2461
166	649.382	166	79.6465
167	653.219	167	80.0371

168	656.938	168	80.4297
169	660.603	169	80.7988
170	664.247	170	81.1777
171	667.813	171	81.5605
172	671.429	172	81.8955
173	675.076	173	82.2715
174	678.694	174	82.6641
175	682.187	175	83.1035
176	685.741	176	83.4961
177	689.324	177	83.7822
178	692.892	178	84.0352
179	696.375	179	84.2559
180	699.604	180	84.4443
181	702.764	181	84.6299
182	705.808	182	84.8057
183	714.996	183	96.2139
184	702.131	184	71.1768
185	711.183	185	84.2783
186	711.622	186	84.46
187	711.519	187	84.2959
188	711.613	188	84.2441
189	711.775	189	84.3047
190	711.839	190	84.3965
191	711.938	191	84.4697
192	712.102	192	84.7939
193	710.827	193	83.1211
194	710.552	194	83.0361
195	710.435	195	83.1719
196	710.334	196	83.2764
197	711.133	197	84.4346
198	712.636	198	86.1895
199	713.669	199	87.4863
200	717.124	200	91.373
201	716.384	201	90.7012
202	713.013	202	86.7324
203	711.875	203	85.3516
204	711.411	204	84.7246
205	711.118	205	84.3076
206	708.26 206	81.0527	
207	707.596	207	81.041
208	707.575	208	81.2373
209	707.647	209	81.4922
210	707.278	210	81.1553
211	704.314	211	78.1025

212	699.451	212	73.4287
213	695.656	213	69.9658
214	694.772	214	69.6084
215	697.907	215	72.5811
216	704.722	216	77.7129
217	713.711	217	88.3242
218	701.69 218	79.7197	
219	696.916	219	72.5918
220	697.167	220	72.1104
221	699.246	221	74.2939
222	699.805	222	75.3516
223	699.787	223	75.2871
224	699.369	224	74.8633
225	699.217	225	74.6748
226	699.104	226	74.5732
227	699.133	227	74.5449
228	699.057	228	74.5508
229	700.934	229	74.5752
230	703.923	230	74.6064
231	705.54 231	74.6328	
232	705.875	232	74.7197
233	705.876	233	74.7881
234	705.893	234	74.8613
235	705.933	235	74.9316
236	705.911	236	75.0098
237	705.872	237	75.0947
238	705.834	238	75.1475
239	705.766	239	75.2227
240	705.701	240	75.2744
241	705.665	241	75.3096
242	705.62 242	75.376	
243	705.575	243	75.4805
244	705.508	244	75.5654
245	705.458	245	75.6143
246	705.435	246	75.6396
247	705.386	247	75.665
248	705.353	248	75.6982
249	705.316	249	75.7256
250	705.27 250	75.749	
251	705.249	251	75.7666
252	705.194	252	75.791
253	705.149	253	75.8535
254	705.09 254	75.9072	
255	705.022	255	75.9453

256	704.999	256	75.9482
257	704.981	257	75.9658
258	704.929	258	75.9971
259	704.867	259	76.0342
260	704.813	260	76.0654
261	704.769	261	76.0898
262	704.738	262	76.126
263	704.679	263	76.1357
264	704.639	264	76.1787
265	704.589	265	76.2178
266	704.544	266	76.2393
267	704.505	267	76.2764
268	704.469	268	76.2783
269	704.449	269	76.2715
270	704.396	270	76.252
271	704.339	271	76.2598
272	704.314	272	76.2734
273	704.28 273	76.2783	
274	704.252	274	76.293
275	704.219	275	76.291
276	704.158	276	76.293
277	704.098	277	76.3057
278	704.05 278	76.3145	
279	704 279	76.3271	
280	703.954	280	76.3604
281	703.901	281	76.3779
282	703.859	282	76.4111
283	703.813	283	76.4053
284	703.809	284	76.3975
285	703.783	285	76.3936
286	703.74 286	76.3975	
287	703.678	287	76.4219
288	703.584	288	76.4414
289	703.498	289	76.4102
290	703.441	290	76.3691
291	703.427	291	76.3516
292	703.41 292	76.3447	
293	703.389	293	76.3594
294	703.357	294	76.3799
295	703.309	295	76.3848
296	703.273	296	76.3936
297	703.229	297	76.417
298	703.192	298	76.4199
299	703.175	299	76.4023

300	703.154	300	76.4121
301	703.114	301	76.4189
302	703.078	302	76.4287
303	703.018	303	76.4385
304	702.965	304	76.4395
305	702.917	305	76.4414
306	702.846	306	76.4463
307	702.799	307	76.4492
308	702.764	308	76.4424
309	702.713	309	76.4316
310	702.678	310	76.415
311	702.637	311	76.415
312	702.598	312	76.4307
313	702.544	313	76.4414
314	702.508	314	76.459
315	702.48 315	76.4971	
316	702.442	316	76.4912
317	702.406	317	76.4551
318	702.355	318	76.4473
319	702.32 319	76.416	
320	702.287	320	76.3887
321	702.258	321	76.3906
322	702.229	322	76.3906
323	702.203	323	76.3916
324	702.172	324	76.4102
325	702.134	325	76.4414
326	702.098	326	76.4619
327	702.08 327	76.4805	
328	702.021	328	76.4834
329	701.952	329	76.4854
330	701.886	330	76.4551
331	701.823	331	76.4463
332	701.774	332	76.4346
333	701.712	333	76.4102
334	701.664	334	76.4131
335	701.639	335	76.4131
336	701.617	336	76.4033
337	701.607	337	76.4131
338	701.599	338	76.4307
339	701.562	339	76.4443
340	701.516	340	76.4277
341	701.486	341	76.4443
342	701.449	342	76.4541
343	701.411	343	76.4717

344	701.389	344	76.4814
345	701.353	345	76.4951
346	701.31 346	76.4951	
347	701.259	347	76.457
348	701.198	348	76.4277
349	701.128	349	76.4199
350	701.067	350	76.4023
351	700.999	351	76.3809
352	700.942	352	76.3809
353	700.927	353	76.3789
354	700.901	354	76.3613
355	700.872	355	76.3652
356	700.841	356	76.377
357	700.802	357	76.3789
358	700.753	358	76.3877
359	700.714	359	76.4082
360	700.675	360	76.4082
361	700.628	361	76.3984
362	700.602	362	76.3965
363	700.562	363	76.3955
364	700.537	364	76.3848
365	700.489	365	76.377
366	700.46 366	76.3496	
367	700.413	367	76.3496
368	700.366	368	76.334
369	700.333	369	76.3105
370	700.302	370	76.2988
371	700.257	371	76.3203
372	700.246	372	76.3203
373	700.218	373	76.3125
374	700.19 374	76.3223	
375	700.142	375	76.3164
376	700.104	376	76.3252
377	700.048	377	76.3262
378	700.015	378	76.3379
379	699.981	379	76.3301
380	699.94 380	76.3271	
381	699.907	381	76.3145
382	699.878	382	76.3086
383	699.833	383	76.333
384	699.808	384	76.3418
385	699.753	385	76.3418
386	699.706	386	76.3477
387	699.657	387	76.3496

388	699.599	388	76.332
389	699.548	389	76.2988
390	699.501	390	76.2832
391	699.472	391	76.2539
392	699.448	392	76.2109
393	699.438	393	76.3721
394	699.409	394	77.5439
395	699.372	395	78.6328
396	699.335	396	78.7959
397	699.292	397	78.8076
398	699.231	398	78.8018
399	699.177	399	78.7715
400	699.122	400	78.7422
