# Supplementary material for: Lung Surfactant Protein B Peptide Mimics Interact with the Human ACE2 Receptor
Source: Int J Mol Sci. 2023 Jun 29;24(13):10837. doi: 10.3390/ijms241310837 (PMC10341807; doi:10.3390/ijms241310837)
Supplement: Supplementary file 1 [file ijms-24-10837-s001.zip › File S4_FTIR spectrum of Peptides in DMSO.pdf]

S4 Supplement file – Secondary Structure of B-YL and SMB Peptides in DMSO

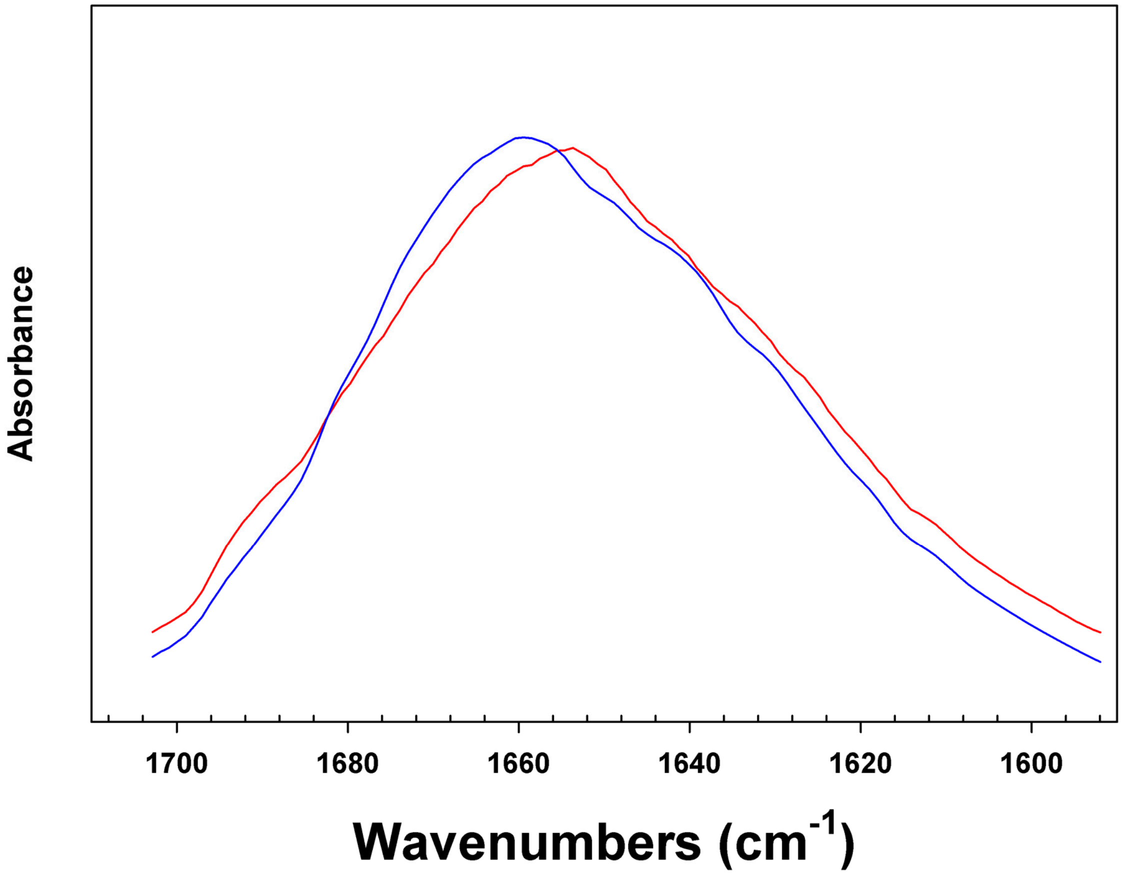

FTIR spectrum of B-YL (blue) and SMB (red) peptides amide I band in dimethyl sulfoxide (DMSO) solvent. Peptides were dissolved in DMSO at a concentration of 4 mM. Infrared spectra were recorded at 37°C using a Jasco FT/IR-4600 FTIR spectrometer (Jasco, Easton, MD) fitted with an ATR Pro One monolithic diamond ATR and a deuterium triglyceride sulfate (DLATGS) detector. The samples were averaged over 256 scans and at a resolution of 4 cm^{-1}. The spectra for peptide in DMSO were obtained by subtracting the peptide-solvent spectrum form a reference spectrum of

DMSO solvent. Relative amounts of α-helix, β-turn, β-sheet, or random (disordered) structures in either peptide self-films or lipid-peptide films were estimated using Fourier self-deconvolution (GRAMS/AI8, version 8.0, Themo Electron Corporation, Waltham, MA) and area of component peaks calculated using curve-fitting software (Igor Pro, version 1.6, Wavemetrics, Lake Oswego, OR) [30]. FTIR frequency limits were: α-helix (1662-1645 cm^{-1}), β-sheet (1637-1613 cm^{-1} and 1710-1682 cm^{-1}), turn/bend (1682-1662 cm^{-1}), and disordered or random (1650-1637 cm^{-1}) [31]. The relative contributions of the various conformations are shown in Table 1. The dominant conformation of the peptides in DMSO is alpha helix with absorbance between 1662-1645 cm^{-1} followed by turn, beta sheet and disordered structures.

Percent Conformation in DMSO

**Peptide**	**Alpha Helix**	**Beta Sheet**	**Turn**	**Disordered**
**B-YL**	**30.69**	**21.67**	**26.67**	**20.77**
**SMB**	**33.83**	**19.04**	**20.61**	**26.52**

References

30. Kauppinen, J.K.; Moffatt, D.J.; Mantsch, H.H.; Cameron, D.G. Fourier self-deconvolution: A method for resolving intrinsically overlapped bands. *Appl. Spectr.* 1981, 35, 271–276.
31. Byler, D.M.; Susi, H. Examination of the secondary structure of protein by deconvolved FTIR spectra. *Biopolymers* 1986, 25, 469–487.
